# Single‐Site and Cooperative Bond Activation Reactions with Ylide‐Functionalized Tetrylenes: A Computational Study

**DOI:** 10.1002/ejic.202100816

**Published:** 2021-10-26

**Authors:** Henning Steinert, Julian Löffler, Viktoria H. Gessner

**Affiliations:** ^1^ Faculty of Chemistry and Biochemistry Ruhr-Universität Bochum Universitätsstraße 150 44780 Bochum Germany

**Keywords:** Bond activation, Density functional calculations, Main group elements, Tetrylenes, Ylides

## Abstract

Due to their transition metal‐like behavior divalent group 14 compounds bear huge potential for their application in bond activation reactions and catalysis. Here we report on detailed computational studies on the use of ylide‐substituted tetrylenes in the activation of dihydrogen and phenol. A series of acyclic and cyclic ylidyltetrylenes featuring various α‐substituents with different *σ*‐ and *π*‐donating capabilities have been investigated which demonstrate that particularly *π*‐accepting boryl groups lead to beneficial properties and low barriers for single‐site activation reactions, above all in the case of silylenes. In contrast, for the thermodynamically more stable germylenes and stannylenes an alternative mechanism involving the active participation of the ylide ligand in the E−H bond (E=H or PhO) activation process by addition across the element carbon linkage was found to be energetically favored. Furthermore, the boryl substituted tetrylenes allowed for a further activation pathway involving the active participation of the boron element bond. These cooperative mechanisms are especially attractive for the heavier cyclic ylidyltetrylenes in which the loss of the protonated ylide group is prevented due to the cyclic framework. Overall, the present studies suggest that cyclic ylide‐substituted germylenes and stannylenes bear huge potential for cooperative bond activations at mild conditions which should be experimentally addressed in the future.

## Graphical abstract

Computational studies on the use of ylide‐functionalized heavier carbenes (with E=Si, Ge, Sn) in bond activation reactions revealed that these species can undergo both, single‐site as well as cooperative activation processes. The latter involve the active participation of the ylide moiety and are particularly attractive for the more stable germylenes and stannylenes, thus opening new perspectives for future catalyst design.

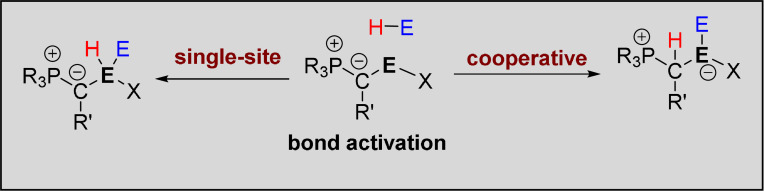

## Introduction

In the past 20 years, the application of main group element compounds in bond activation reactions have received intense research interests.^[1]^ Particularly, divalent group 14 compounds have been studied in great detail ever since the discovery by Bertrand and coworkers that singlet carbenes can activate dihydrogen under mild reaction conditions.^[2]^ In contrast to carbenes, the ground electronic state of their heavier congeners R_2_E (with E=Si, Ge and Sn) is usually a singlet state, which gives rise to a high energy lone pair (HOMO) and an energetically accessible vacant *p*‐orbital (LUMO).^[3]^ This orbital setup mimics the frontier *d*‐orbitals of transition metals and leads the dual donor and acceptor character necessary for bond activations.^[1]^ The reactivity of tetrylenes towards small molecules and strong bonds greatly depends on the nature of the R substituents which determine the orbital energies. Thus, many different substituents with varying steric and electronic properties have been employed until to date to tune the ability of the tetrylene to engage in bond activation reactions.[^4^, ^5^]

The singlet‐triplet gap and the HOMO‐LUMO separation were found to be a valuable measures for the ability of carbenes and related species to engange in bond activation reactions. In general, small HOMO‐LUMO gaps lead to a higher activity of tetrylenes towards small molecules as was already demonstrated by Bertrand and coworkers by means of the different behavior of N‐heterocyclic carbenes (NHCs) compared to cyclic alkyl(amino)carbenes (CAACs) towards dihydrogen.^[2]^ Two key factors influence this energy separation: the angle at the group 14 element and the donor/acceptor properties of the substituents.^[6]^ Large angles are associated with small HOMO‐LUMO gaps due to the higher *p*‐character of the lone pair leading to an elevation of the HOMO energy.^[7]^ Thus, acyclic tetrylenes are usually more reactive than there cyclic derivatives. Likewise, bulky groups are beneficial for small molecule activation since they also increase the R−E−R angle. For electronic manipulation a broad variety of substituents have been employed particularly for the heavier group 14 analogues of carbenes.^[5]^ Whereas amino groups owing to their −I and +M donor properties are privileged substituents to stabilize tetrylenes, they usually cause a large HOMO‐LUMO separation. More reactive species suitable for small molecule activation are thus generated with strongly *σ*‐donating (i. e. more electropositive) groups, which leave the empty *p*‐orbital unpopulated, hence lower in energy and available for interactions with bonding orbitals of further substrates. Thus, besides alkyl moieties, also silyl or more lately boryl groups have been used in this chemistry.^[5]^ From a thermodynamic perspective the heavier group 14 carbene analogues seem to be more attractive for establishing reversible and hence catalytic processes. While E−H activation reactions with carbenes are highly exothermic and hence irreversible, reversible bond activations have been reported for their heavier analogues, albeit catalytic applications are still scarce.[^8^, ^9^]

**Figure 1:**
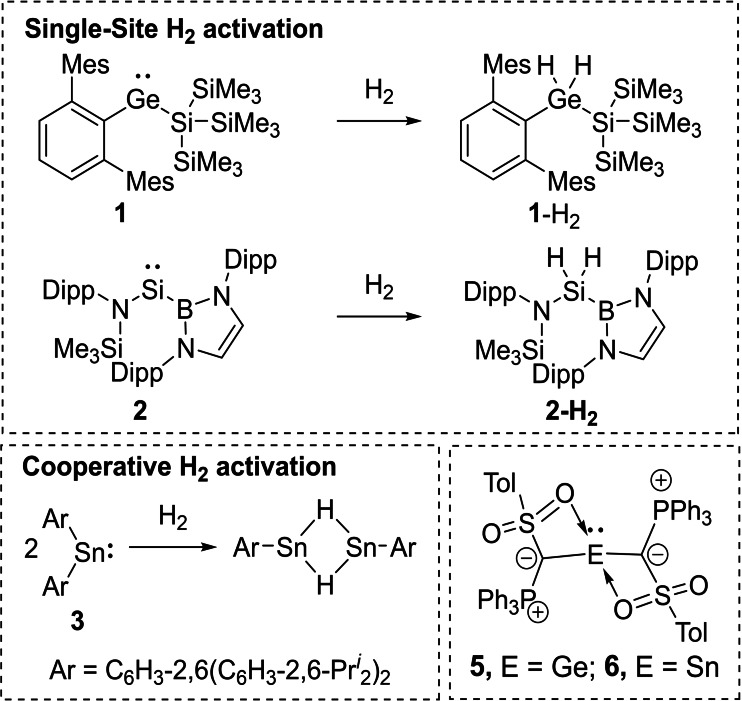
H_2_‐Activation on the germylene **1** and the silylene **2** by Aldridge (top) and Power (bottom left) as well as the recently reported tetrylenes by our group.

Due to the special electronic structure of tetrylenes and the presence of a filled and empty orbital at the group 14 element, bond activation reactions typically occur directly at the metal center (single site).[^4^, ^5^] These activation processes have been investigated in detail,^[10]^ and are exemplarily depicted in Figure 1 by means of the silyl‐substituted germylene **1** ^[5a]^ and the boryl‐functionalized silylene **2** ^[5b]^ reported by Aldridge and coworkers. Besides this single‐site reactivity, also E−H bond activation processes involving the active participation of the *α*‐substituent (cooperative) have been described in literature.^[11]^ For example, Power reported on dihydrogen activation with stannylene **3** via arene elimination (Figure 1).^[12]^ Similar reactivities have been reported with other substituents, e. g. with the *β*‐diketiminato ligand.^[13]^

Recently, our group has focused on the use of ylides as substituents in main group chemistry.[^14^, ^15^] Starting from *s*‐block metal yldiides we were able to isolate the diylide‐substituted germylene and stannylene **5** and **6** which exhibited unusual electronic properties.[^15a^, ^15b^, ^15c^, ^16^] While diamino tetrylenes typically show *π*‐donation from the amino group into the empty *p*‐orbital at the group 14 element, **5** and **6** exhibited an alignment of all three lone pairs in the C−E−C linkage thus leading to remarkably increased HOMO and LUMO levels and donor strengths. These properties led us to investigate the potential of ylide‐substituted tetrylenes in bond activation reactions. Since attempts to use **5** and **6** in the activation of dihydrogen remained unsuccessful so far, we set out to evaluate the properties of differently substituted ylidyltetrylenes of type YER (with Y=ylide, R=2^nd^ substituent, E=Si, Ge, Sn). Recent calculations by Phukan and coworkers showed that cyclic amino(ylide) substituted tetrylenes exhibit reasonable low barriers for the activation of small molecules, but usually higher LUMO energies than diaminotetrylenes.[^17^, ^18^]

Here, we show that particularly cyclic and acyclic *push‐pull* tetrylenes are promising candidates for bond activations. Most importantly, not only single‐site activations at the group 14 element but also bifunctional bond activation pathways were found to be viable processes.

## Results and Discussion

### Computational details

All calculations were performed without symmetry restrictions using the Gaussian16 Revision B.01^[19]^ or the Gaussian16 Revision C.01^[20]^ program packages. If possible starting coordinates were directly obtained from crystal structure analyses; otherwise GaussView 6.0^[21]^ was used. The structures of the tetrylenes, activation products and transition states were optimized using the PW6B95 functional^[22]^ with Grimme's D3 dispersion correction with Becke‐Johnson (BJ) damping^[23]^ and the def2svp basis set^[24]^ as well as the MWB46 ECP^[25]^ as implemented in Gaussian for Sn. For benchmarking, energy‐optimization of **5** was also performed with the PBE0^[26]^ and the BP86^[27]^ functional. However, comparison of the obtained structure parameters with those from the XRD analysis revealed the best agreement with the PW6B95 functional. All optimized structures have been confirmed as energetic minima on the potential energy surface by calculation of the harmonic frequencies on the same level of theory and showed only positive Hessian eigenvalues for the ground states, or a single imaginary frequency for the transition states corresponding to the H−H− or O−H bond elongation.^[28]^ Single point calculation on the PW6B95D3^[22]^/def2tzvp^[24]^ level of theory with the MWB46 ECP^[25]^ for Sn as implemented in Gaussian16 were performed to access the final energies. The gas‐phase energies (1 atm) are converted to 1 M standard state by adding 7.9259 kJ/mol to each species. Reaction energies are calculated as the difference of the sum of energies of all products and the sum of energies of all reagents: E=ΣE(products)‐ΣE(reactants). Natural bond orbital (NBO) analyses were performed with NBO Version 7.^[29]^

### Acyclic ylide tetrylenes (AYT)

**Figure 2:**
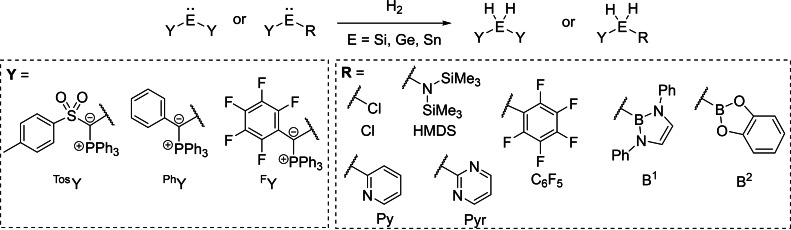
Structures of the different ylide‐substituted tetrylenes and their use in single site dihydrogen activation.

To study the propensity of ylide‐substituted tetrylenes to undergo bond activations we at first chose a set of tetrylenes with three different ylide groups (Figure 2). The sulfonyl ylide ^**Tos**^ **Y** was chosen due to the facile synthesis of these compounds via the corresponding metalated ylide, whereas the aryl substituted ylides ^**Ph**^ **Y** and ^**F**^ **Y** were selected due to their simple design. As second substituents R, a selection of commonly applied electron‐donating and electron‐withdrawing groups were used to study the impact of different electronic properties on the activity of the compounds towards E−H bond activation. Besides a second ylide group, a simple chloro substituent as well as the “classical“ bis(trimethylsilyl)amide group (HMDS) already employed by Lappert in the 1970s^[30]^ were used. Furthermore, the boryl substituents **B^1^** and **B^2^** were included in this study, since a **B^1^**‐like substituent was previously described to significantly lower the activation barrier for H_2_‐splitting with germylenes.^[5a]^ Also, the electron‐poor aryl groups pentafluorophenyl (C_6_F_5_), pyrimidine (Pyr) and pyridine (Py) were investigated to further examine *push‐pull*‐effects in ylidyltetrylenes.

**Figure 3:**
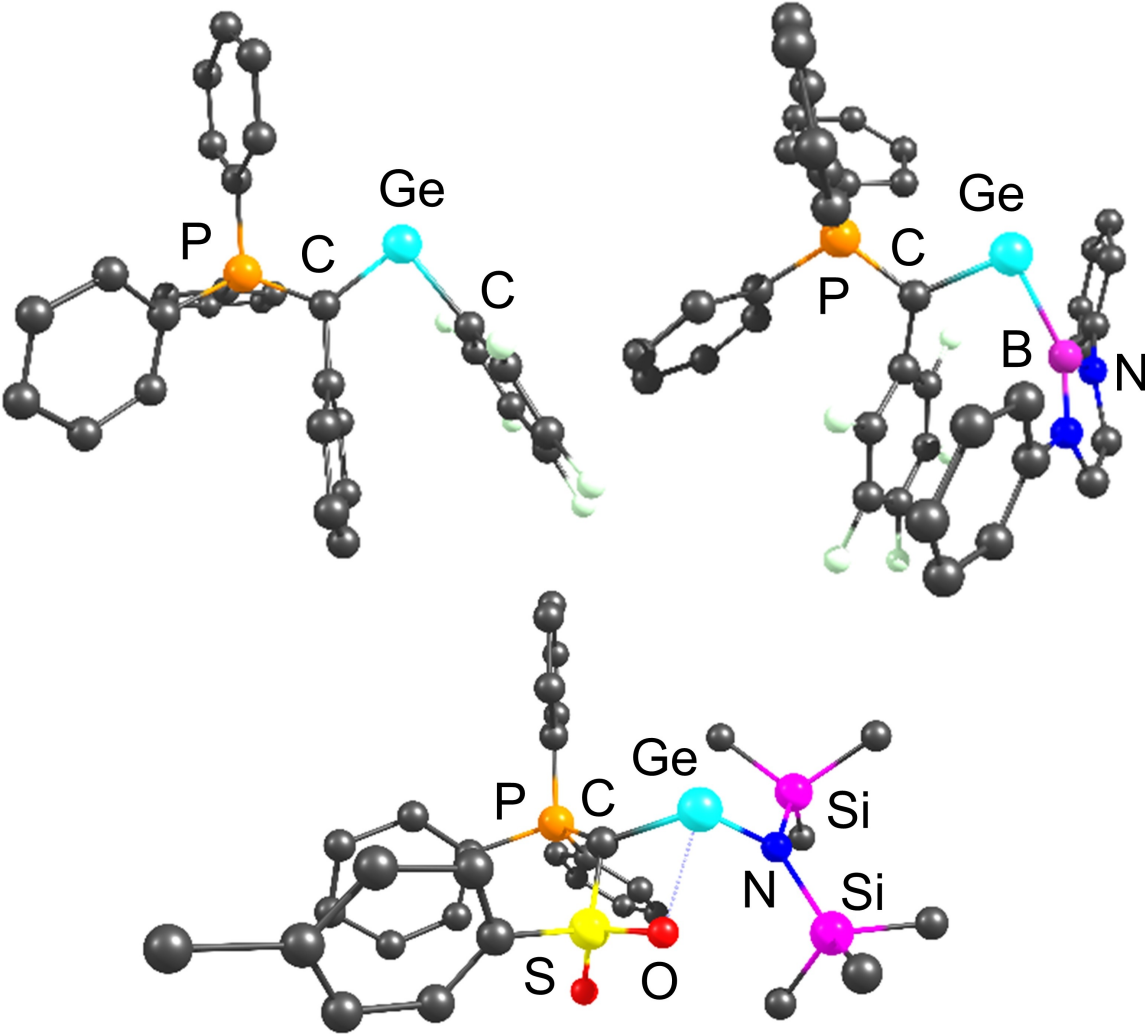
The optimized structures of ^**Tos**^ **YGeC_6_F_5_** (upper letft), ^**F**^ **YGeB^1^** (upper right), ^**Tos**^ **YGeHMDS** (bottom). Hydrogen atoms are omitted for clarity.

The energy optimized structures of three selected germylenes are shown in Figure 3. In all optimized structures of the ^**Tos**^ **Y**‐substituted tetrylenes, the tosyl group coordinates to the group 14 element thus thermodynamically stabilizing the tetrylene. This conformation results – in accordance with previous experimental observations^[15]^ – in a perpendicular arrangement of the C−E−C linkage relative to the ylide moiety (P−C−S plane). In contrast, the ^**Ph**^ **Y** and ^**F**^ **Y** substituted systems feature the expected co‐planar arrangement. This allows for a stabilizing *π‐*interaction between the ylide and the group 14 element and hence in shorter C−E distances compared to the corresponding ^**Tos**^ **YER** systems. However, the aryl groups in the ylide backbone of these tetrylenes are not in plane but rotated out of the E−P−C plane, thus preventing further delocalization of electron density into the aromatic substituent. This arrangement has also been observed in other ylide‐substituted main group compounds such as phosphenium cations^[31]^ and is probably the result of the steric demand of the PPh_3_ group. Instead, the aryl groups in the ylide backbone arrange parallel to aromatic groups of the R substituents (C_6_F_5_, Py, Pyr, B^1^ and B^2^), thus resulting in stabilizing *π*‐interactions (cf. Figure 3) and rather acute C^Ylide^−E−R‐angles (Table 1). It is interesting to note, that unlike in NHCs the amino group in the HMDS‐substituted compounds does not show ideal co‐planarity with the tetrylene centre probably also due to its high steric demand.

**Table 1:** C−E−R Angle [°] and NBO charges (given in [e]) at the tetrylene and the ylidic carbon centre as well as the *s*‐ and *p*‐character [%] of the lone pair at the group 14 element for selected examples of the investigated acyclic tetrylenes. The values for all tetrylenes can be found in Table S2.1 in the SI.

		Si					Ge					Sn				
Y	R	C−Si−R‐ angle	NBOanalysis				C−Ge−R‐ angle	NBOanalysis				C−Sn−R‐ angle	NBO‐Analysis			
			q(Si)	q(C)	*s*	*p*		q(Ge)	q(C)	*s*	*p*		q(Sn)	q(C)	*s*	*p*
^Tos^Y	Y	103.8	0.97	−1.49^[a]^	72	28	102.7	0.99	−1.49^[a]^	81	19	100.4	1.22	−1.54^[a]^	84	16
	Cl	100.6	0.91	−1.49	78	22	99.5	0.96	−1.50	86	14	97.5	1.16	−1.55	89	11
	HMDS	104.3	1.10	−1.46	73	27	103.5	1.12	−1.47	83	17	99.9	1.31	−1.53	86	14
	C_6_F_5_	96.5	1.00	−1.47	72	28	141.7	1.01	−1.47	81	19	90.9	1.21	−1.53	86	14
	Py	94.7	0.99	−1.46	72	28	93.1	1.00	−1.46	80	20	89.2	1.17	−1.52	85	15
	B^2^	89.2	0.64	−1.44	69	31	87.6	0.66	−1.44	78	22	86.6	0.92	−1.49	81	19
^Ph^Y	Y	107.4	0.73	−1.14^[a]^	68	32	105.1	0.72	−1.12^[a]^	76	24	101.7	0.95	−1.17^[a]^	84	16
	Cl	98.9	0.77	−1.21	78	22	96.8	0.79	−1.19	84	16	93.5	0.98	−1.23	88	12
	HMDS	103.6	0.97	−1.17	74	26	101.0	0.96	−1.14	81	19	97.8	1.16	−1.20	86	14
	C_6_F_5_	93.3	0.84	−1.17	74	26	91.3	0.84	−1.15	80	20	87.2	1.04	−1.21	85	15
	Py	95.4	0.84	−1.18	73	27	93.5	0.83	−1.16	79	21	89.9	1.00	−1.20	84	16
	B^2^	89.4	0.48	−1.15	70	30	87.3	0.48	−1.28	76	24	84.4	0.73	−1.18	81	19

[a] Average values.

**Figure 4:**
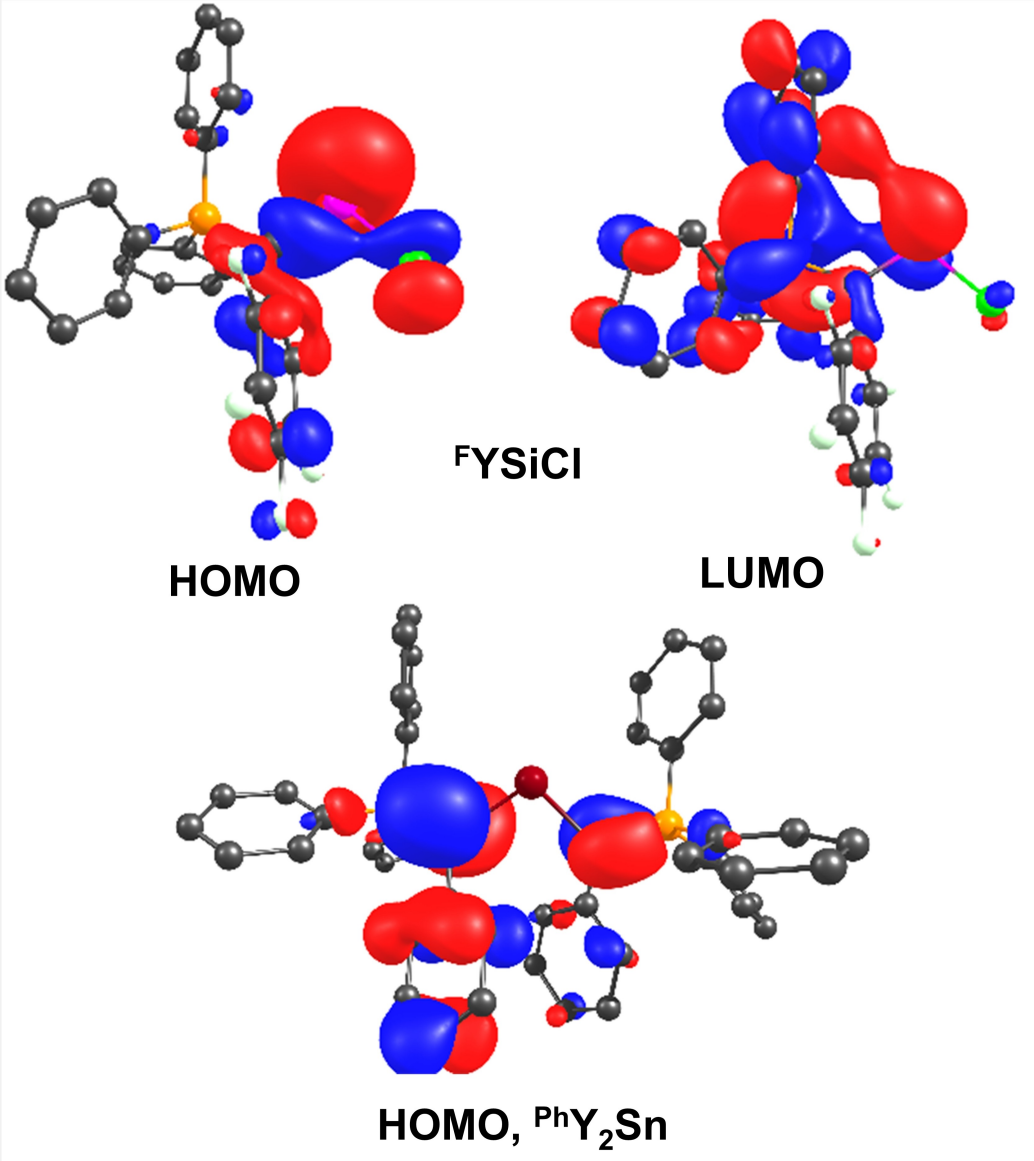
The HOMO (upper left) and LUMO (upper right) of ^**F**^ **YSiCl** and the HOMO of ^**Ph**^ **Y_2_Sn** (bottom). Hydrogen atoms are omitted for clarity.

Since the energies of the HOMO as well as the LUMO at the tetrylene centre were shown to correlate with the propensity of tetrylenes to undergo bond activations,[^2^, ^4^] we first calculated these values for all compounds. Whereas the frontier orbitals in divalent group 14 compounds are usually located at the group 14 element, this was not necessarily the case for all tetrylenes considered in this study. Overall, it was found that for the more electron‐rich tetrylenes – in particular the silylenes and the ^**Ph**^ **Y** substituted compounds – the HOMO is mostly located at the central group 14 element (Figure 4). In contrast, for the more electron‐poor tetrylenes – in particular the stannylenes – the HOMO is mostly located at the ylidic carbon atoms (e. g. ^**Ph**^ **Y_2_Sn**, Figure 3) and the HOMO‐1 is predominantly found at the tetrylene centre. It is also important to note, that for all ^**Tos**^ **Y** and most of the ^**Ph**^ **Y**‐substituted systems the LUMO is not located at the tetrylene centre, but in the ylide backbone.

**Figure 5:**
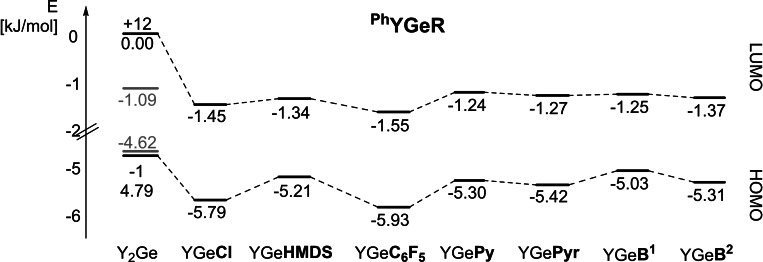
Plot of the energies of the frontier orbitals of the germylenes with the phenyl‐substituted ylide group ^**Ph**^ **Y**. Energies of the orbitals predominantly localized at the germanium center are given in black. In case that these orbitals are not the HOMO or LUMO, the HOMO and LUMO energies are given in grey.

In general, the highest HOMO and LUMO energies of all compounds (see SI for detailed information) were found for ^**Ph**^ **Y**, the lowest for the electron poor ylide ^**F**^ **Y**. Furthermore, some general trends can be deduced for the influence of the second substituent on the orbital energies. Figure 5 exemplarily depicts the energies of the frontier orbitals of the germylenes ^**Ph**^ **YGeR** with the phenyl‐substituted ylide group. Firstly, due to the strong donor ability of the ylide substituents, the diylidyltetrylenes **Y_2_E** show high HOMO and particularly high LUMO energies. In contrast, the chloro and perfluorphenyl‐substituted compounds show the lowest HOMO energies as a consequence of their electron withdrawing properties. Also, the HMDS‐substituted tetrylenes show high HOMO energies as a result of their high steric demand and the resulting larger Y−E−N bond angles (Table 1). Lastly, the Py, Pyr, B^1^ and B^2^ substituted systems show surprisingly high HOMO energies, despite their acute C^Ylide^−E−R‐angles. This is a consequence of Bents rule, i. e. the tendency of less electron withdrawing substituents to concentrate atomic *s*‐character in their bonding orbitals.^[32]^ As a consequence the lone pair at the tetrylene centre has a higher *p*‐character (Table 1) and is therefore destabilized. Thus, the lowest HOMO‐LUMO gaps can be found for the boryl substituted systems YEB^1^ and YEB^2^, thus suggesting that those should be best suited for bond activation reactions, which correlates well with the observations made by Aldridge and coworkers on terphenyl‐substituted germylenes.^[5a]^

Alike the HOMO and LUMO energies the energy of the singlet‐triplet gap E_ST_ was shown to correlate with the propensity of tetrylenes to undergo bond activations.^[10]^ Therefore we also calculated E_ST_ for all compounds, which are given in Table 2 and Table 3 as well as the SI. In general, the tetrylenes with a given substitution pattern can be divided into three categories according to the trends in the singlet‐triplet gaps:

**Table 2:** E_ST_ [in kJ/mol] of the investigated cyclic tetrylenes.

	Si	Ge	Sn
**I**	245.6	213.8	162.7
**II**	220.1	215.5	186.2
**III**	157.0	191.7	217.2
**IV**	107.9	83.1	49.7
**V**	52.0	60.5	56.8

**Table 3:** E_ST_, ΔG^TS^ and ΔG^Pro^ [kJ/mol] for the single‐site H_2_‐activation with the ^Ph^Y‐substituted acyclic ylide‐substituted tetrylenes (Pathway A).

		Si			Ge			Sn		
Y	R	E_ST_	ΔG^TS^	ΔG^Pro^	E_ST_	ΔG^TS^	ΔG^Pro^	E_ST_	ΔG^TS^	ΔG^Pro^
PhY	Y	150.0	126.3	−126.2	161.4	184.0	−44.3	146.5	229.2	−0.8
	Cl	172.4	161.4	−109.2	170.9	217.5	−21.3	146.9	269.1	29.4
	HMDS	158.7	143.4	−122.3	186.3	197.0	−31.5	154.9	249.6	22.9
	C6F5	127.8	125.3	−114.5	191.2	185.4	−29.2	166.4	247.8	26.6
	Py	127.7	105.5	−125.9	138.9	154.7	−42.9	122.3	204.8	6.9
	Pyr	133.4	108.8	−123.3	149.0	162.8	−47.3	129.2	215.5	5.2
	B1	112.6	91.0	−120.8	119.2	132.0	−59.7	113.9	183.1	−12.6
	B2	102.0	77.6	−128.3	113.0	124.3	−52.1	109.0	182.4	1.8

1. The E_ST_ increases from the silylenes over the germylenes to the stannylenes. This is the expected trend according to hybridization defects within the groups of the main group elements. However, it must be noted that the formal excitation of an electron must not necessarily take place at the tetrylene centre (i. e. from the lone pair into the vacant orbital).^[17]^ Instead, the formal excitation may also take place into the backbone of the ligand. In these cases, the E_ST_ should be independent of the central atom. This divergent behaviour is predominant for tetrylenes with a high electron density at the group 14 element and for those stabilized by a tosyl group due to the coordination of the oxygen atoms and the elevation of the LUMO energy.
2. The E_ST_ decreases from the silylene over the germylene to the stannylene. This presumably is the result of a formal excitation of an electron from the lone pair at the ylidic carbon atom either into the vacant orbital at the group 14 element or in the ylide backbone. Since the lone pair at the ylidic carbon atom is less stabilized with decreasing electronegativity of the central element, the E_ST_ decreases when going down the group. This is also in line with the calculated NBO charges (Table 1) and is the predominant effect for the tetrylenes with a high electron density at the ylidic carbon atom.
3. The combination of the behaviours described above results in a maximum of E_ST_ for the germylene.

As was previously described by several groups, electron donating groups stabilize the divalent group 14 compounds, but result in higher singlet‐triplet gaps compared to the other groups.[^4^, ^7^] Therefore, the lowest singlet‐triplet gaps are obtained by the introduction of boryl substituents, in particular **B^2^**. Thus, ylidyltetrylenes follow the same principles as established for aminocarbenes. Consequently, the lowest singlet‐triplet gap is found for ^**Tol**^ **YSiB^2^** with an energy of 95.4 kJ/mol, while the highest value is calculated for ^**Tol**^ **YSnCl** with 220.2 kJ/mol.

### Cyclic ylide tetrylenes (CYT)

**Figure 6:**
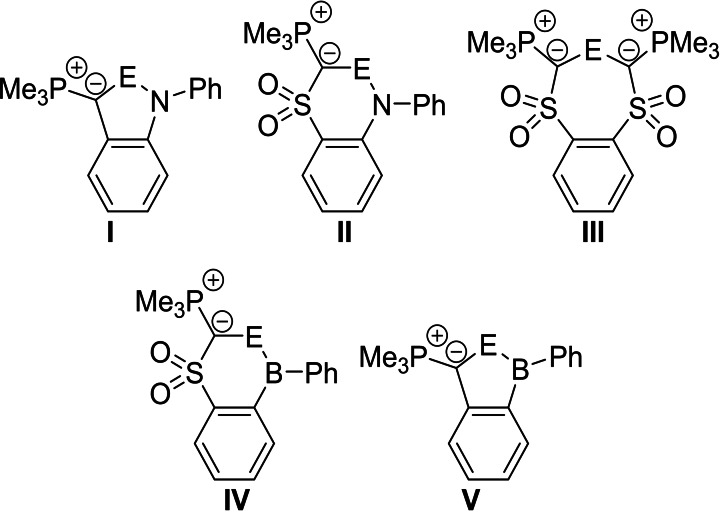
Investigated CYTs **I**–**V**.

Besides the acyclic ylide substituted tetrylenes we also examined the impact of a cyclic geometry by calculating compounds **I**–**V** (Figure 6).^[33]^ To save computational costs, a trimethyl phosphonium instead of a PPh_3_ group was used. Due to increased ring strain in the cyclic structures, sulfonyl‐coordination to the central element was only observed for the tin compounds **II^Sn^** and **III^Sn^**. For **IV**, the lowest energy structure exhibits an interaction between the sulfonyl group and the boron atom. Overall, the cyclic systems showed similar substituent effects on the HOMO and LUMO energies as well as on E_ST_ as found for the acyclic derivatives. As such, the highest HOMO and lowest LUMO energies and the lowest E_ST_ were found for the boryl substituted systems **IV** and **V** (Table 2).

### H−H‐bond activation

**Scheme 1:**
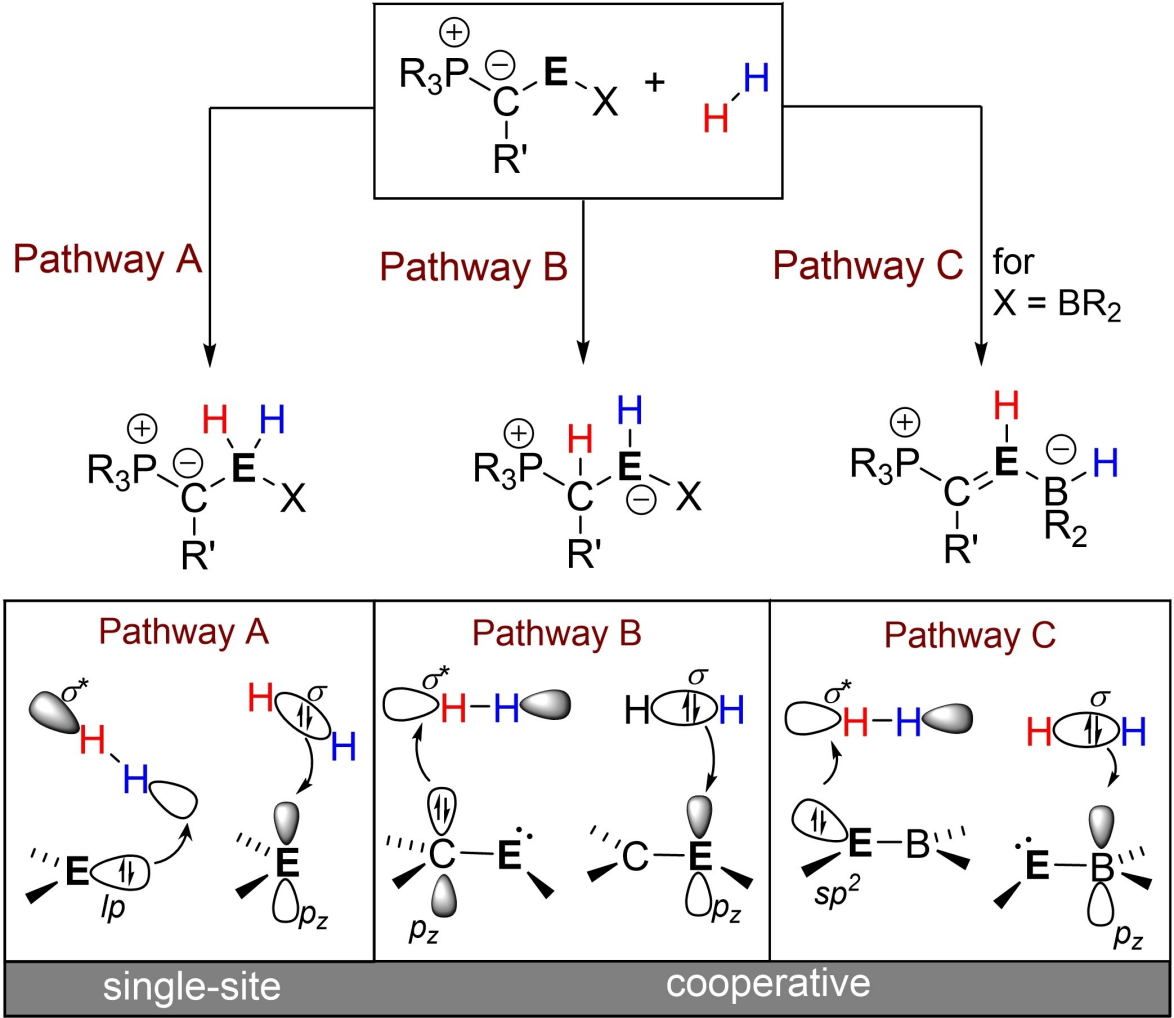
Possible pathways for the activation of dihydrogen by ylide‐substituted tetrylenes and the corresponding orbital interactions.

Next, we addressed the potential of the tetrylenes to activate dihydrogen. While previous reports on the use of ylide‐substituted carbenes and their heavier congeners only considered single site activations at the central element, we considered three possible pathways: A) the “classical” splitting at the tetrylene centre[^2^, ^5^, ^34^] and B) activation via element ligand cooperation by 1,2 addition across the E−C or C) across the E−B linkage (Scheme 1). Pathway A results in the formation of a tetrel dihydride (Scheme 1, Pathway A) and is based on the interaction of the tetrylenes lone pair with the antibonding σ* orbital of the dihydrogen with a concomitant donation of electron density from the bonding *σ*‐orbital of H_2_ into the vacant orbital at the tetrylene centre.^[2]^ In case of pathway B, cooperative H−H activation is achieved through electron‐donation from the lone pair at the ylidic carbon atom, while the group 14 element acts as acceptor site. In pathway C, this element‐ligand cooperativity is reversed with the group 14 element acting as donor and the boron centre as acidic site. The results of the single‐site H_2_ activation reactions with the tetrylenes with the Ph‐substituted ylide ^Ph^Y are given in Table 3, those for all other systems in the Supporting Information (Table S2.2).

In line with the calculated singlet‐triplet and HOMO‐LUMO gaps, the stannylenes showed the highest barriers between 190 (for ^**Ph**^ **YSnB^2^**) and 312 kJ/mol (for ^**Tos**^ **YSnHMDS**) for the single‐site activation (Pathway A), the silylenes the lowest (77.6–174.5 kJ/mol). This corroborates with previous studies on other tetrylenes.^[35]^ In general, the use of an electron‐donating second substituent is detrimental for facile H_2_ activation, whereas the use of electron withdrawing groups, especially boryl substituents, results in lower barriers. As a result, the lowest barrier of only 78 kJ/mol is found for ^**Ph**^ **YSiB^2^**. This barrier is sufficiently low for dihydrogen activation at room temperature. The influence of the nature of the ylide on the activation energies is somewhat smaller than the impact of the R substituent, but still significant. As such, ^Ph^Y in general provides lower reaction barriers than the electron poorer ^F^Y and ^Tos^Y, especially for heavier tetrylenes. Regarding the thermodynamics of the activation processes it is noteworthy that in contrast to the silylenes and germylenes, most of the reaction processes of the stannylenes are thermodynamically uphill due to the high stability of the +2 oxidation state of tin.

In case of the cyclic ylide substituted tetrylenes, H_2_ activation is again thermodynamically most favoured for the boryl substituted systems with a maximum gain of energy of 147.3 kJ/mol for **V^Si^** (Table 4). In contrast, the thermodynamically most unfavoured reactions are observed for the most electron rich tetrylenes **I**, which is also in line with their high HOMO/LUMO separations and their high E_ST_. Unfortunately, we were not able to locate the transition states for the H_2_ activation with most of the borylated species since optimizations always resulted in the cooperative pathways C. Sole exception was **IV^Si^** which showed a low reaction barrier of only 93.8 kJ/mol. This low activation barrier is in line with the acyclic systems and should be easily accessible even at room temperature. It is interesting to note that the cyclic diylidyltetrylenes **III** show higher barriers than the corresponding acyclic systems ^**Tos**^ **Y_2_E**, which reflects the impact of the R−E−R angle on the frontier orbitals.

**Table 4:** Comparison of the activation barriers and reaction energies for the reaction of the cyclic tetrylenes **I**–**V** with dihydrogen via pathway A (single‐site) as well as B and C (cooperative). ΔG^TS^ and ΔG^Pro^ are given in kJ/mol.

		Si		Ge		Sn^[a]^	
Tetrylene/Pathway		ΔG^TS^	ΔG^Pro^	ΔG^TS^	ΔG^Pro^	ΔG^TS^	ΔG^Pro^
**I**	A	224.1	−53.8	274.0	34.1	321.2	88.8
	B	185.4	25.5	188.4	15.2	174.3	−3.8
**II**	A	174.9	−99.9	232.6	−12.7	285.7	56.3
	B	161.8	−0.5	153.0	−8.1	n.o.	−4.7
**III**	A	124.1	−144.4	185.6	−53.6	262.0	18.9
	B	121.6	22.7	122.5	21.5	135.4	29.5
**IV**	A	93.8	−136.7	n.o.	−75.9	no.	−15.6
	B	113.1	3.4	109.3	5.2	114.9	31.7
	C	83.0	−41.0	88.2	−31.3	113.5	−30.7
**V**	A	n.o.^[a]^	−147.3	n.o.^[a]^	−84.3	n.o.^[a]^	−25.0
	B	80.6	−80.6	89.0	−63.5	94.7	−42.1
	C	41.2	−115.5	57.1	−75.9	55.4	−57.8

[a] n.o.=not observed. The corresponding transition state could not be located. Optimizations always gave the corresponding transition states of the cooperative bond activations.

**Figure 7:**
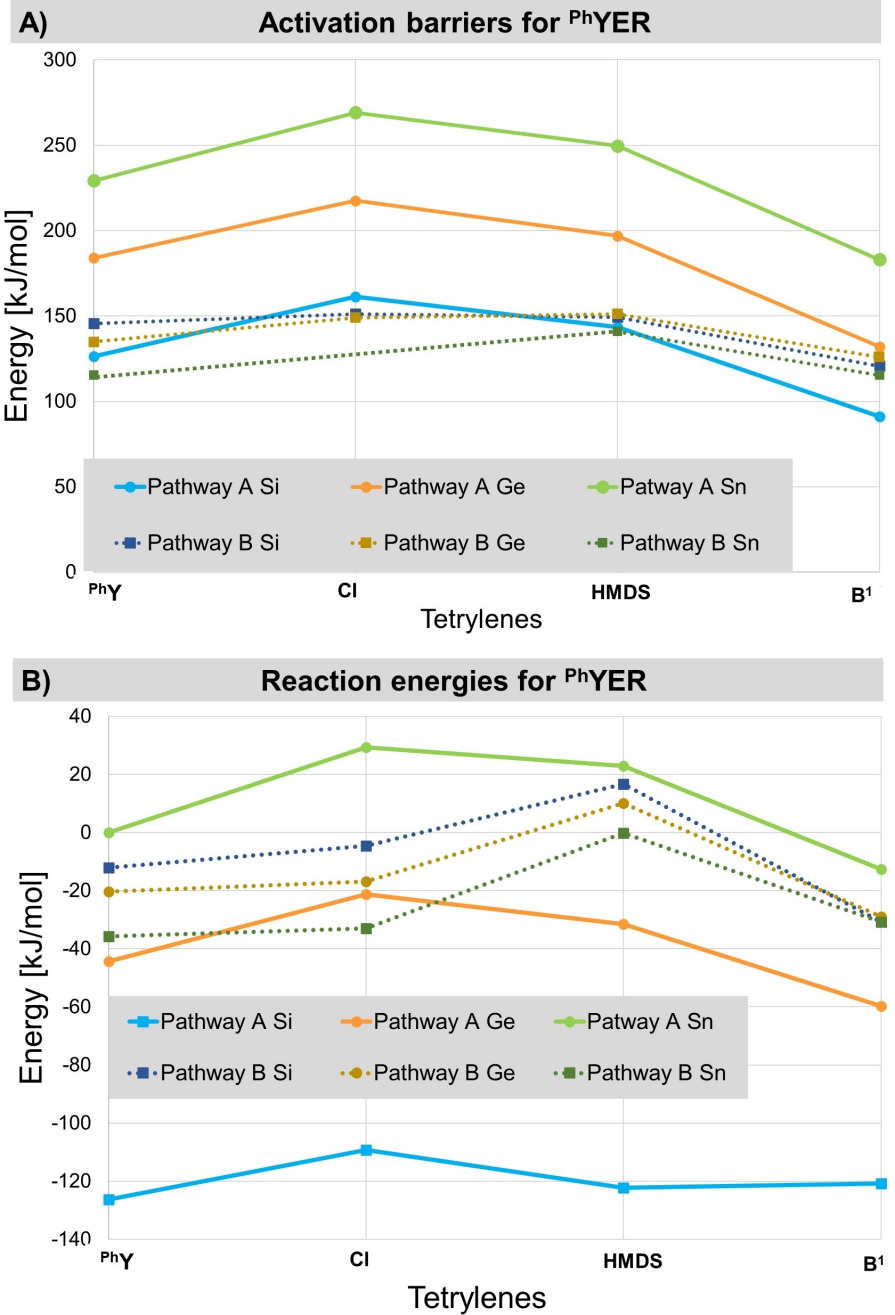
Comparison of the activation barriers and reaction energies for the reaction of the acyclic tetrylenes ^**Ph**^ **YER** (with R=^Ph^Y, Cl, HMDS, B^1^; E=Si, Ge, Sn) with dihydrogen via pathway A (single‐site) and B (cooperative).

Next, we addressed the cooperative dihydrogen activation via pathway B and C for a selection of tetrylenes. Table 5 shows the results for pathway B for the acyclic tetrylenes ^**Tos**^ **YER** and ^**Ph**^ **YER**, Figure 7 and Table 4 a comparison of the activation barriers and reaction energies of pathways A and B for ^**Ph**^ **YER** and pathways A−C for the cyclic tetrylenes (values are given in the SI). The most distinct difference of the cooperative bond activation pathways (pathway B and C) compared to the single‐site bond activations is the independence of their activation barriers and reaction energies of the group 14 element. While for the activation of H_2_ at the element center the reaction barriers clearly decrease from Sn to Si and the energy gain increases in the same direction, only small energetic differences can be observed for the cooperative pathways. Thus, the reaction energies for pathway B and C only vary by 30 kJ/mol with the group 14 element, whereas for the single‐site processes differences of even more than 100 kJ/mol can be observed.

**Table 5:** ΔG^TS^ and ΔG^Pro^ [kJ/mol] of the H_2_‐activation on AYTs along the E−C unit (Pathway B).

		Si		Ge		Sn	
Y	R	ΔG^TS^	ΔG^Pro^	ΔG^TS^	ΔG^Pro^	ΔG^TS^	ΔG^Pro^
^Tos^Y	Y	207.9	134.3^[a]^	206.8	158.5^[a]^	191.5	149.2
	Cl	158.5	41.9	n.d.	25.8^[a]^	136.3	15.3
	HMDS	179.5	39.7	172.5	28.6^[a]^	n.d	50.4
	B1	115.4	18.0	122.8	25.7^[a]^	n.d.	40.5
^Ph^Y	Y	145.6	−12.1	134.8	−20.4	115.5	−35.7
	Cl	151.2	−4.6	149.1	−16.9	n.d.	−33.0
	HMDS	149.4	16.7	151.2	10.1	141.0	−0.1
	B1	120.6	−30.8	126.0	−28.9	115.3	−30.7

[a] In these products, the C(ylide)‐element bond is broken und the ylide is coordinating via the sulfonyl group.

**Table 6:** ΔG values of the coordinated species, the transition states, and the products of the phenol activation on CYTs relative to the starting materials in kJ/mol.

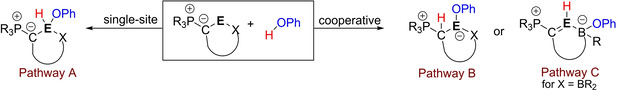										
		Si			Ge			Sn		
	Pathway	ΔG^coord^	ΔG^TS^	ΔG^Pro^	ΔG^coord^	ΔG^TS^	ΔG^Pro^	ΔG^coord^	ΔG^TS^	ΔG^Pro^
**I**	A	−1.1	114.7	−168.9	1.7	163.0	−23.8	n.o.	n.o.	47.8
	B	−3.0	63.3	−64.7	−4.1	55.5	−39.2	−7.7	27.3	−60.1
**II**	A	n.o.	102.9	−197.3	n.o.	148.0	−51.1	n.o.	n.o.	27.6
	B	2.2	43.8	−88.6	4.4	31.7	−65.7	−7.6	31.7	−80.9
**III**	A	−6.2	43.1	−240.6	7.8	108.1	−97.4	6.6	154.3	−7.2
	B	−21.2	45.1	−64.2	−16.0	28.5	−49.0	−36.6	16.4	−44.6
**IV**	A	0.8	69.5	−226.3	−6.2	80.2	−120.5	−5.1	104.9	−60.7
	B	2.7	23.6	−54.2	−3.6	22.7	−32.7	−6.4	7.8	−44.5
	C	51.6	118.5	−89.2	60.5	145.0	−49.3	89.8	204.0	−143.9^[a]^
**V**	A	2.9	62.9	−231.2	3.9	86.9	−110.3	0.0	122.2	−58.5
	B	−5.7	18.0	−140.8	−8.4	14.7	−101.8	−12.6	−8.6	−102.0
	C	2.9	79.4	−158.5	3.9	91.9	−144.8	n.o.	109.3	−149.0^[a]^

[a] Product of the E−C or E−B bond cleavage.

The transition state energies of pathway B and C are in general in the range of those for the single‐site activation for the silylenes. Thus, these pathways are particularly kinetically favored over the single‐site mechanism for the germylenes and stannylenes. In case of the silylenes the barriers of all processes A−C are in the same range. For the acyclic tetrylenes ^**Ph**^ **YER**, the activation energies for pathway B lie between 115 and 150 kJ/mol and are thus slightly too high to be overcome at room temperature but should be accessible at elevated temperatures. This is especially interesting for the thermodynamically more stable germylenes and stannylenes, which exhibit much higher barriers via pathway A.

It is noteworthy that the energies of the dihydrogen activation with the acyclic, phenyl‐substituted system ^**Ph**^ **YER** depend only little on the nature of substituent R, while the differences are greater in the tosyl compound. Similar to the single‐site process the lowest activation barriers are found for the systems with a boryl group. Comparison of the values of the two different acyclic tetrylenes ^**Ph**^ **YER** and ^**Tos**^ **YER** (Table 5) also shows that the activation and reaction energies of the cooperative H_2_ splitting are considerably affected by the substituent in the ylide‐backbone. This is easy to understand, since the ylide‐substituent is directly bound to the anionic carbon center and hence controls the electron density at the active ligand site. Thus, the barriers for the tetrylenes ^**Tos**^ **YER** with the anion‐stabilizing sulfonyl group are in general higher and the energy gain lower than those found for the systems with the ^**Ph**^ **Y** group. This is also a consequence of the additional stabilization of the low‐valent group 14 element through additional coordination of the tosyl group. It is also noteworthy, that in case of the sulfonyl‐substituted systems cooperative bond activation often led to the cleavage of the C−E bond and hence in the elimination of the ylide. This cooperates with the experimental observations and the facile formation of free ylide in reactions with compounds **5** and **6** and thus confirms the necessity for cyclic structures to ensure thermodynamic stability of the molecular framework upon E−H bond activation.^[15]^

The cyclic tetrylenes (Table 4) showed similar trends in the activation and reaction energies for the single‐site and cooperative bond activation processes than their acyclic congeners (Table 4), including the independence of pathway B from the nature of the group 14 element. Unfortunately, we could only in one case (for **IV^Si^**) locate the transition states for all three different pathways A–C. Here, the cooperative bond activation via addition of H_2_ across the Si−B linkage was found to be the most favourable process. In general, pathway C exhibited very low activation barriers of up to only 41 k/mol for **V^Si^**, thus making this process particularly facile and also thermodynamically favourable. In some cases, also the barrier for the reverse reactions (H_2_ elimination) was found to require less than 130 kJ/mol thus suggesting that also reversible activation processes should be possible. It is also noteworthy that the reaction barriers for the sulfonyl substituted derivatives **IV** are significantly higher than those for the Ph‐substituted ylide **V**. This can be explained by the additional O→B‐interaction in **IV** which stabilizes the tetrylene and thus increases ΔG^TS^.

Overall, the comparison of the different pathways A−C with the cyclic and acyclic ylide‐substituted tetrylenes clearly shows that the cooperative bond activation reactions are clearly favoured for the stannylenes and germylenes. In particular, *push*‐*pull* systems such as the boryl substituted compounds exhibit low barriers. These compounds even seem to be attractive target systems for reversible hydrogenation processes and thus for catalytic applications. However, to this end, cyclic tetrylenes need to be constructed to prevent ligand dissociation as was already experimentally observed for acyclic ylide‐substituted tetrylenes.^[15]^

### O−H‐bond activation on CYTs

After having examined the activation of dihydrogen we next turned our attention towards the activation of the polar O−H bond. Surprisingly, even though the high reactivity of tetrylenes towards OH‐groups is well known, its mechanism has little been investigated.[^4^, ^36^] Because of their greater synthetic potential in reversible processes we focussed our studies on the cyclic ylide‐substituted tetrylenes **I**–**V** and investigated the three different mechanisms discussed above for the activation of phenol (Table 6). Due to the Lewis basicity of phenol this process was found to be a two‐step process for most of the tetrylenes proceeding via formation of an intermediate coordination complex. In this complex, phenol coordinates either to the vacant orbital at the group 14 element (pathways A and B) or the vacant orbital at boron (pathway C). In general, these precoordinated species thermodynamically only slightly differ from the starting materials. Sole exceptions are the precoordinated species of **IV** for pathway C because of the intramolecular stabilization of the boron centre through the sulfonyl group. In these cases, the phenol coordination complexes are clearly disfavoured relative to the starting materials.

In almost all cases, the phenol activation products are thermodynamically favoured over the tetrylenes independent of the activation mechanism. The only exceptions were found for the activation reactions with the stannylenes **I^Sn^** and **II^Sn^** via pathway A due to the higher stability of tin(II) compounds (Table 6). Consistently, the thermodynamically most favoured products were found for the single‐site O−H activations by the silylenes due to the instability of silicon(II) compounds and the oxophilicity of silicon and the high Si−O bonding energies, respectively. Thus, pathway A is especially favourable for the silicon compounds. Nonetheless, pathway B is always kinetically favoured over pathway A, even for the silylenes. In general, low activation barriers are observed, which should easily be overcome at room temperature. Similar to the dihydrogen activation, the barriers for pathway B – in contrast to pathway A – decrease when going down the group. Also thermodynamically, the stability of the 1,2‐addition relative to the single site activation products increases from Si to Sn. Thus, the cooperative activation processes are particularly favoured for the heavier carbenes.

As for the dihydrogen activation, the lowest barriers are found for the boryl substituted systems **IV** and **V**. It is interesting to note that pathway C, gives thermodynamically more stable products than pathway B, probably due to the oxophilicity of boron. However, they required higher activation energies suggest that they are only accessible when reactions are performed under thermodynamic control.

## Conclusion

In summary, the present computational studies demonstrate that ylide‐substituted tetrylenes possess remarkably high donor properties that combined with π‐accepting boryl substituents lead to systems with ideal properties for bond activation reactions (low singlet‐triplet and HOMO‐LUMO gaps). These compounds are not only capable of activating H−H and O−H bond via single site processes at the divalent group 14 element centre, but also via cooperative mechanisms with the ylidic carbon atom acting as basic site. Accordingly, the reactivity of these species is also influenced by the substituent in the ylide backbone, which thus offers a further handle for reactivity control. Overall, the cooperative activation processes are particularly beneficial for germylenes and stannylenes which usually are more stable than their silicon counterparts and hence feature higher barriers in the “classical” single‐site activation processes. Since the bifunctional bond activation via protonation of the ylide would result in the cleavage of the element‐carbon bond, these processes are especially interesting for cyclic tetrylenes to prevent destructive elimination of the ylide substituent. For the boryl‐functionalised compounds also a further cooperative activation pathway was found to be a viable alternative in which the tetrylene centre acts as base and boron as acid.

Overall, the presented calculations suggest that ylide‐substituted heavier carbenes are promising main group systems for reversible bond activations via element ligand cooperation. Experimental endeavours should particularly focus on the construction of cyclic compounds due to their higher stability towards elimination of the ylide.

## Conflict of interest

The authors declare no conflict of interest.

## Supporting information

As a service to our authors and readers, this journal provides supporting information supplied by the authors. Such materials are peer reviewed and may be re‐organized for online delivery, but are not copy‐edited or typeset. Technical support issues arising from supporting information (other than missing files) should be addressed to the authors.

Supporting Information
