# Supplementary material for: Single‐Site and Cooperative Bond Activation Reactions with Ylide‐Functionalized Tetrylenes: A Computational Study
Source: Eur J Inorg Chem. 2021 Oct 26;2021(47):5004–13. doi: 10.1002/ejic.202100816 (PMC9298247; doi:10.1002/ejic.202100816)
Supplement: Supplementary file 1 — Supporting Information [file EJIC-2021-5004-s001.pdf]

European Journal of Inorganic Chemistry

Supporting Information

Single-Site and Cooperative Bond Activation Reactions with Ylide-Functionalized Tetrylenes: A Computational Study

Henning Steinert, Julian Löffler, and Viktoria H. Gessner*

Index

1 General remarks ... 3
2 Overview Over Important Computational Results ... 4
2.1 HOMO and LUMO Energies ... 4
2.2 Results from the NBO analysis ... 8
2.3 Singlet-triplet gaps and energies for the single-site activation of H_2 ... 9
2.4 Energies for the H_2 activation reactions with the cyclic tetrylenes (pathway A-C) ... 10
3 Energies and coordinates of the small molecules ... 12
3.1 Energies ... 12
3.2 Coordinates of the structures ... 12
4 Results of the Calculations of all Silylenes ... 13
4.1 Energies ... 13
4.1.1 Energies of the Silylenes ... 13
4.1.2 Energies of the H_2 activation ... 15
4.1.3 Energies of the phenol activation ... 18
4.2 Coordinates of the Structures ... 20
4.2.1 Singlet state structures ... 20
4.2.2 Triplet state structures ... 52
4.2.3 H_2 activation transition states ... 84
4.2.4 H_2 activated products ... 128
4.2.5 Phenol coordinated species ... 176
4.2.6 Phenol activation transition states ... 186
4.2.7 Phenol activated products ... 198
5 Results of the Calculations of all Germylenes ... 210
5.1 Energies ... 210
5.1.1 Energies of the Germylenes ... 210
5.1.2 Energies of the H_2 activation ... 212
5.1.3 Energies of the phenol activation ... 215
5.2 Coordinates of the Structures ... 217
5.2.1 Singlet state structures ... 217
5.2.2 Triplet state structures ... 253
5.2.3 H_2 activation transition states ... 284
5.2.4 H_2 activated products ... 328
5.2.5 Phenol coordinated species ... 376
5.2.6 Phenol activation transition states ... 386
5.2.7 Phenol activated products ... 398
6 Results of the Calculations of all Stannylenes ... 410
6.1 Energies ... 410
6.1.1 Energies of the Stannylenes ... 410
6.1.2 Energies of the H_2 activation ... 412
6.1.3 Energies of the phenol activation ... 415
6.2 Coordinates of the Structures ... 417
6.2.1 Singlet state structures ... 417
6.2.2 Triplet state structures ... 449
6.2.3 H_2 activation transition states ... 481
6.2.4 H_2 activated products ... 523
6.2.5 Phenol coordinated species ... 572
6.2.6 Phenol activation transition states ... 581
6.2.7 Phenol activated products ... 590
7 Comparision of Structural Parameters ... 602
8 References ... 603

1 General remarks

All computational studies were carried out without symmetry restrictions. If it was not possible to obtain starting coordinates from crystal structures GaussView 6.0[1] were used. Calculations were performed with the Gaussian16 Revision B.01[2] or the Gaussian16 Revision C.01[3] program packages using Density-Functional Theory (DFT).[4] Energy optimizations were carried out with the PW6B95D3 functional[5] and def2svp basis set[6] as well as the MWB46 ECP[7] as implemented in Gaussian for Sn together with GRIMMES D3 dispersion correction with Becke-Johnson damping.[8] To determine the nature of the structure harmonic vibrational frequency analyses were performed on the same level of theory.[9] No imaginary frequencies were observed for the ground states; for transition states, one imaginary frequency corresponding to the translational motion was observed. Single point energies were calculated on PW6B95D3[5]/def2tzvp[6] level of theory with the MWB46 ECP[7] as implemented in Gaussian for Sn. The energies are corrected by 7.9259 kJ/mol to a 1 M standard solution.

Chemcraft 3D[10] and Gimp[11] were used for graphical representation.

2 Overview Over Important Computational Results

2.1 HOMO and LUMO Energies

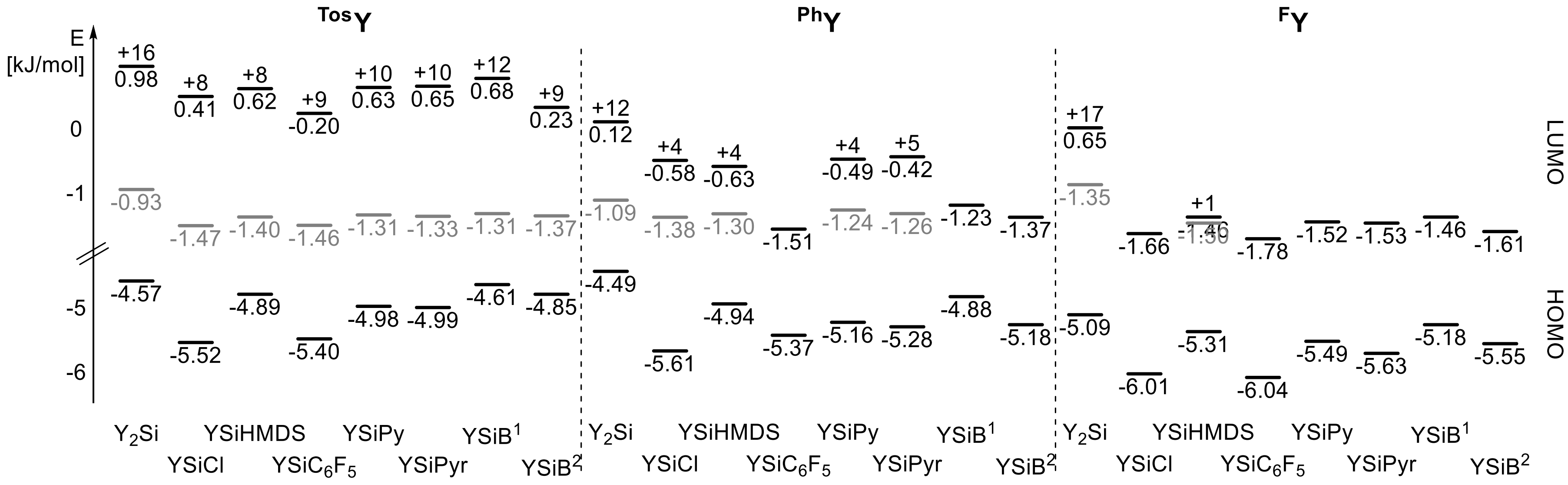

Figure S2.1. HOMO and LUMO energies of the acyclic silylenes. Energies of the orbitals cantered at the silicon center are given in black. In case that these orbitals are not the HOMO or LUMO, the HOMO and LUMO energies are given in grey.

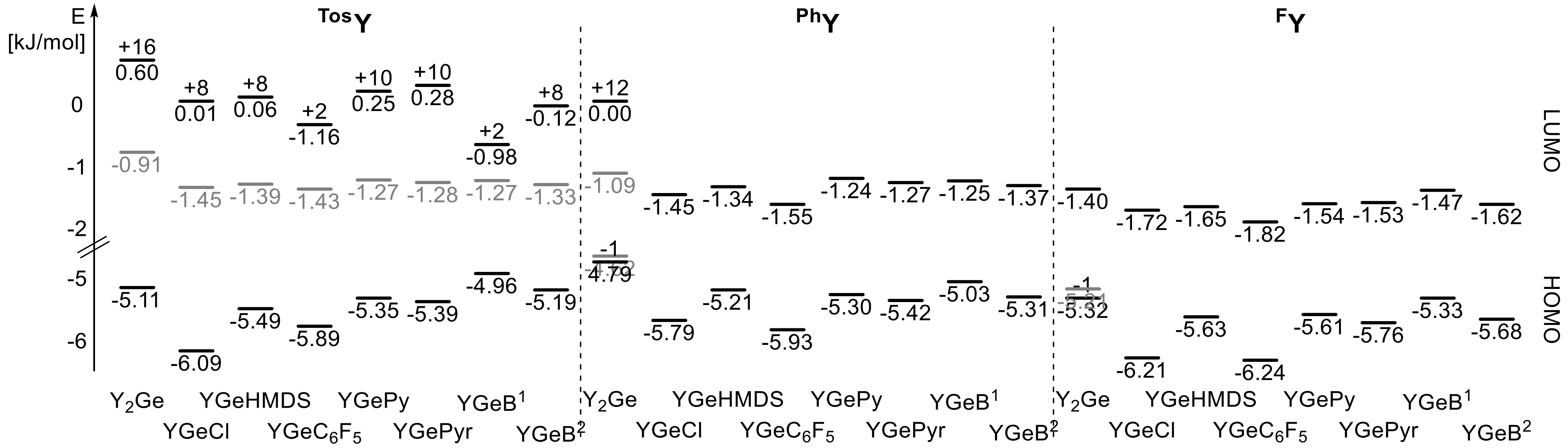

Figure S2.2. HOMO- and LUMO-energies ot the acyclic germylenes. Energies of the orbitals cantered at the germanium center are given in black. In case that these orbitals are not the HOMO or LUMO, the HOMO and LUMO energies are given in grey.

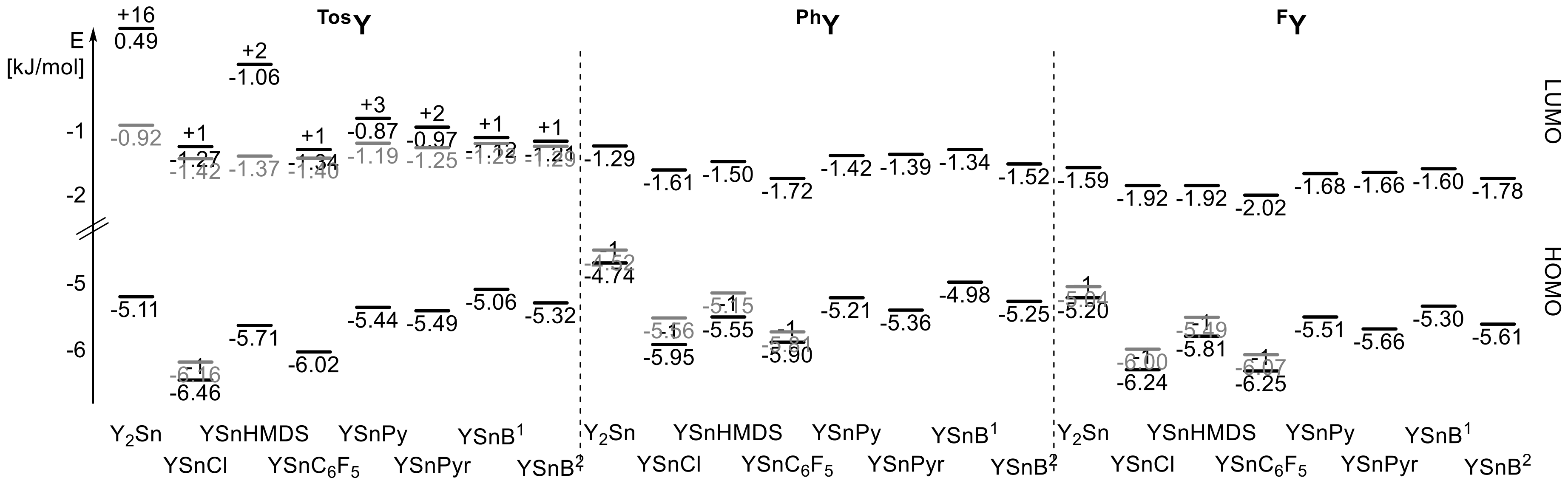

Figure S2.3. HOMO- and LUMO-energies oft the acyclic stannylenes. Energies of the orbitals centered at the tin center are given in black. In case that these orbitals are not the HOMO or LUMO, the HOMO and LUMO energies are given in grey.

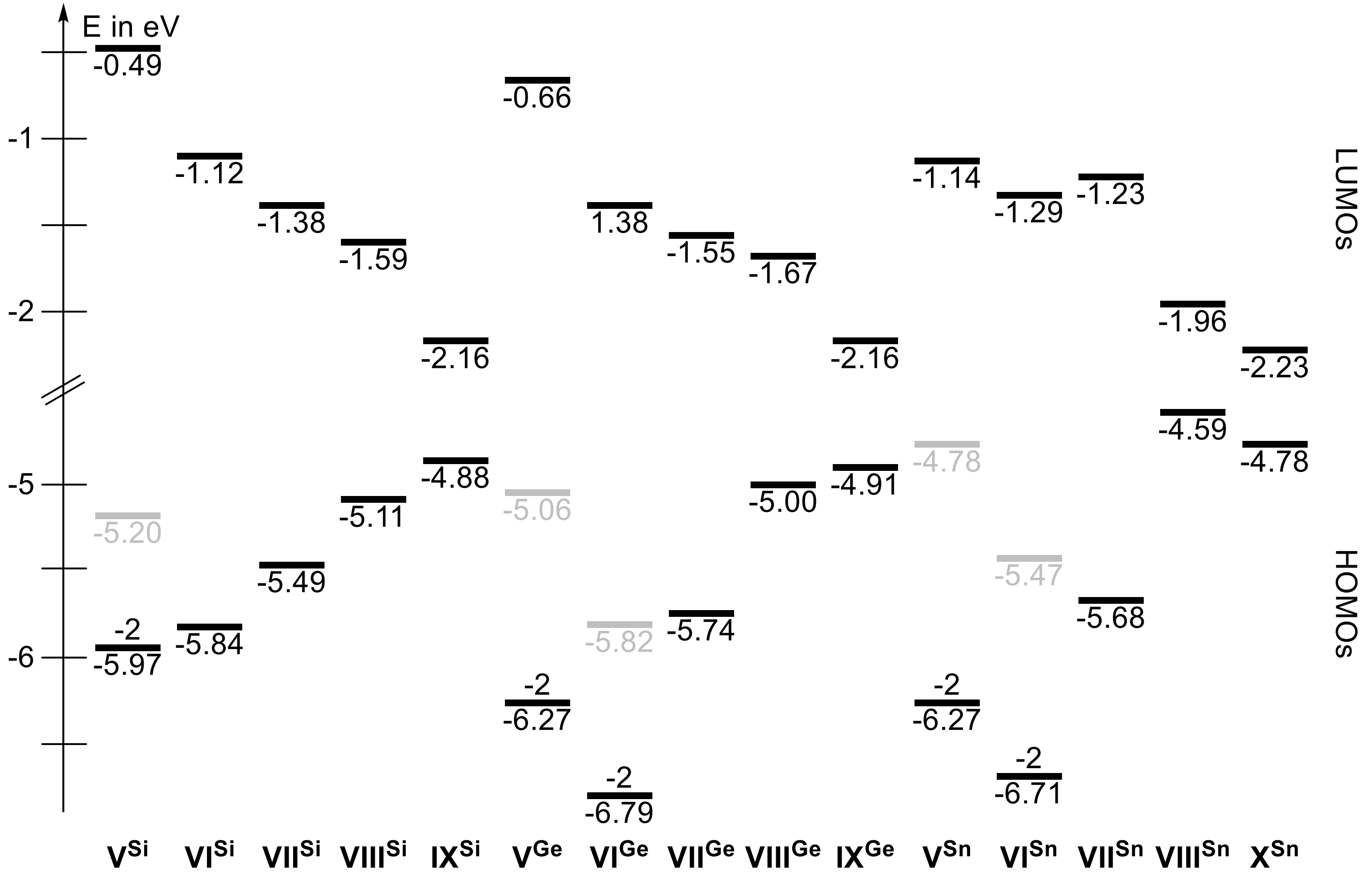

Figure S2.4. HOMO- and LUMO-energies of the CYTs. Energies of the orbitals centered at the group 14 element center are given in black. In case that these orbitals are not the HOMO or LUMO, the HOMO and LUMO energies are given in grey.

2.2 Results from the NBO analysis

Table S2.1 C–E–R Angle [°] and NBO charges at the tetrylene and the ylidic carbon centre as well as the *s*- and *p*-character [%] of the lone pair at the group 14 element for selected examples of the investigated acyclic tetrylenes.

		Si					Ge					Sn				
Y	R	C–Si–R-angle	NBO-Analysis				C–Ge–R-angle	NBO-Analysis				C–Sn–R-angle	NBO-Analysis			
			q(Si)	q(C)	*s*	*p*		q(Ge)	q(C)	*s*	*p*		q(Sn)	q(C)	*s*	*p*
TosY	Y	103.8	0.97	-1.49[a]	72	28	102.7	0.99	-1.49[a]	81	19	100.4	1.22	-1.54[a]	84	16
	Cl	100.6	0.91	-1.49	78	22	99.5	0.96	-1.50	86	14	97.5	1.16	-1.55	89	11
	HMDS	104.3	1.10	-1.46	73	27	103.5	1.12	-1.47	83	17	99.9	1.31	-1.53	86	14
	C_6F_5	96.5	1.00	-1.47	72	28	141.7	1.01	-1.47	81	19	90.9	1.21	-1.53	86	14
	Py	94.7	0.99	-1.46	72	28	93.1	1.00	-1.46	80	20	89.2	1.17	-1.52	85	15
	Pyr	93.0	0.98	-1.45	71	29	91.0	0.98	-1.46	81	19	88.4	1.18	-1.52	85	15
	B^1	92.0	0.64	-1.43	67	33	90.3	0.66	-1.43	76	24	88.1	0.92	-1.48	81	19
	B^2	89.2	0.64	-1.44	69	31	87.6	0.66	-1.44	78	22	86.6	0.92	-1.49	81	19
PhY	Y	107.4	0.73	-1.14[a]	68	32	105.1	0.72	-1.12[a]	76	24	101.7	0.95	-1.17[a]	84	16
	Cl	98.9	0.77	-1.21	78	22	96.8	0.79	-1.19	84	16	93.5	0.98	-1.23	88	12
	HMDS	103.6	0.97	-1.17	74	26	101.0	0.96	-1.14	81	19	97.8	1.16	-1.20	86	14
	C_6F_5	93.3	0.84	-1.17	74	26	91.3	0.84	-1.15	80	20	87.2	1.04	-1.21	85	15
	Py	95.4	0.84	-1.18	73	27	93.5	0.83	-1.16	79	21	89.9	1.00	-1.20	84	16
	Pyr	94.6	0.84	-1.18	73	27	92.2	0.82	-1.16	79	21	88.4	1.01	-1.21	84	16
	B^1	92.2	0.48	-1.15	67	33	90.8	0.48	-1.13	73	27	87.3	0.75	-1.18	80	20
	B^2	89.4	0.48	-1.15	70	30	87.3	0.48	-1.28	76	24	84.4	0.73	-1.18	81	19
FY	Y	107.7	0.81	-1.17[a]	68	32	105.5	0.80	-1.15[a]	76	24	101.6	1.07	-1.21[a]	84	16
	Cl	98.9	0.81	-1.23	79	21	96.7	0.84	-1.21	85	15	92.8	1.03	-1.26	88	12

**HMDS**	102.8	1.02	-1.20	74	26	100.0	1.02	-1.17	82	18	96.7	1.23	-1.24	87	13
**C_6F_5**	94.2	0.88	-1.20	74	26	92.1	0.88	-1.18	80	20	82.8	1.09	-1.24	86	14
**Py**	95.2	0.87	-1.20	73	27	93.1	0.86	-1.18	79	21	89.6	1.05	-1.23	84	16
**Pyr**	93.9	0.88	-1.21	73	27	92.2	0.87	-1.19	79	21	88.6	1.06	-1.24	85	15
**B^1**	93.4	0.52	-1.18	74	26	91.7	0.53	-1.16	74	26	89.2	0.81	-1.21	80	20
**B^2**	87.6	0.53	-1.18	77	23	85.7	0.54	-1.16	77	23	82.8	0.79	-1.21	82	18

2.3 Singlet-triplet gaps and energies for the single-site activation of H_2

Table S2.2. E_{ST}, ΔG^{TS} and ΔG^{Pro} [kJ/mol] for the single-site H_2-activation with the acyclic ylide-substituted tetrylenes (Pathway A).

		**Si**			**Ge**			**Sn**		
**Y**	**R**	**E_{ST}**	**ΔG^{TS}**	**ΔG^{Pro}**	**E_{ST}**	**ΔG^{TS}**	**ΔG^{Pro}**	**E_{ST}**	**ΔG^{TS}**	**ΔG^{Pro}**
**^{Tos}Y**	**Y**	143.7	n.o.	-122.2	179.1	204.8	-36.0	211.8	289.7	50.5
	**Cl**	192.0	157.8	-100.9	214.3	212.3	-18.6	220.2	283.2	56.5
	**HMDS**	152.0	n.o.	-138.2	180.0	220.5	-45.8	208.0	304.7	37.6
	**C_6F_5**	156.9	146.9	-120.5	179.6	205.4	-46.5	193.4	263.0	27.9
	**Py**	138.8	106.7	-132.4	153.6	163.7	-55.5	173.2	232.5	20.4
	**Pyr**	126.4	96.9	-132.4	141.3	145.9	-58.4	161.5	231.0	17.1
	**B^1**	119.4	85.6	-125.9	132.1	150.9	-60.5	148.3	209.5	8.3
	**B^2**	95.4	n.o.	-151.2	111.8	n.o.	-44.3	130.6	n.o.	-10.5
**^{Ph}Y**	**Y**	150.0	126.3	-126.2	161.4	184.0	-44.3	146.5	229.2	-0.8
	**Cl**	172.4	161.4	-109.2	170.9	217.5	-21.3	146.9	269.1	29.4
	**HMDS**	158.7	143.4	-122.3	186.3	197.0	-31.5	154.9	249.6	22.9
	**C_6F_5**	127.8	125.3	-114.5	191.2	185.4	-29.2	166.4	247.8	26.6

	**Py**	127.7	105.5	-125.9	138.9	154.7	-42.9	122.3	204.8	6.9
	**Pyr**	133.4	108.8	-123.3	149.0	162.8	-47.3	129.2	215.5	5.2
	**B[1]**	112.6	91.0	-120.8	119.2	132.0	-59.7	113.9	183.1	-12.6
	**B[2]**	102.0	77.6	-128.3	113.0	124.3	-52.1	109.0	182.4	1.8
**^{F}Y**	**Y**	157.4	174.5	-114.3	176.8	232.6	-43.7	171.3	269.3	10.9
	**Cl**	185.2	168.6	-100.5	191.7	228.4	-52.1	169.2	276.5	45.1
	**HMDS**	155.3	159.7	-129.5	181.6	209.5	-59.7	170.4	265.4	17.9
	**C_6F_5**	148.6	155.9	-106.5	167.0	212.9	-43.7	153.9	279.8	39.4
	**Py**	132.8	139.2	-125.4	154.3	190.6	-27.8	180.8	256.7	6.7
	**Pyr**	139.9	143.1	-120.8	162.0	200.5	-35.9	152.7	262.7	20.1
	**B[1]**	116.6	86.3	-125.9	137.6	133.1	-56.4	141.7	197.7	-7.5
	**B[2]**	109.3	93.2	-114.9	112.4	134.9	-56.6	112.5	192.8	-2.4

2.4 Energies for the H_2 activation reactions with the cyclic tetrylenes (pathway A-C)

Table S2.3. ΔG^{TS} and ΔG^{Pro} [kJ/mol] of the geminal H_2-activation on CYTs (Pathway A).

	**Si**		**Ge**		**Sn[a]**	
	**ΔG^{TS}**	**ΔG^{Pro}**	**ΔG^{TS}**	**ΔG^{Pro}**	**ΔG^{TS}**	**ΔG^{Pro}**
**I**	224.1	-53.8	274.0	34.1	321.2	88.8
**II**	174.9	-99.9	232.6	-12.7	285.7	56.3
**III**	124.1	-144.4	185.6	-53.6	262.0	18.9

**IV**	93.8	-136.7	- [a]	-75.9	- [a]	-15.6
**V**	- [a]	-147.3	- [a]	-84.3	- [a]	-25.0

[a] The transition states could not be located. Optimizations always gave the corresponding transition states of the cooperative bond activations (see below).

Table S2.4. ΔG^{TS} and ΔG^{Pro} [kJ/mol] of the H_2-activation on CYTs along the E–C unit (Pathway B).

	**Si**		**Ge**		**Sn[a]**	
	**ΔG^{TS}**	**ΔG^{Pro}**	**ΔG^{TS}**	**ΔG^{Pro}**	**ΔG^{TS}**	**ΔG^{Pro}**
**I**	185.4	25.5	188.4	15.2	174.3	-3.8
**II**	161.8	-0.5	153.0	-8.1	n.o.	-4.7
**III**	121.6	22.7	122.5	21.5	135.4	29.5
**IV**	113.1	3.4	109.3	5.2	114.9	31.7
**V**	80.6	-80.6	89.0	-63.5	94.7	-42.1

Table S2.5. ΔG^{TS} and ΔG^{Pro} of geminal H_2-activation on CYTs along the E–B-unit (Pathway C).

	**Si**		**Ge**		**Sn[a]**	
	**ΔG^{TS}**	**ΔG^{Pro}**	**ΔG^{TS}**	**ΔG^{Pro}**	**ΔG^{TS}**	**ΔG^{Pro}**
**IV**	83.0	-41.0	88.2	-31.3	113.5	-30.7
**V**	41.2	-115.5	57.1	-75.9	55.4	-57.8

3 Energies and coordinates of the small molecules

3.1 Energies

Silylene	E(SCF)	Corr(H)	Corr(G)	ΔG [hartree]	ΔG [kJ/mol]	1M ΔG [kJ/mol]
**H_2**	-1.17460969	0.01336	-0.001467	–	–	–
**Phenol**	-307.981709	0.112406	0.077115	–	–	–

3.2 Coordinates of the structures

H_2

E = -1.17460968871

H 0.000000 0.000000 0.378370

H 0.000000 0.000000 -0.378370

Phenol

E = -307.981709139

C -0.217884 -1.220414 -0.000109

C -0.943371 -0.028277 -0.000083

C -0.266887 1.191644 0.000026

C 1.123412 1.215967 -0.000123

C 1.850346 0.031438 0.000088

C 1.169006 -1.183541 0.000019

H -0.762765 -2.161400 -0.000064

H -0.834318 2.122927 0.000128

H 1.639089 2.174594 -0.000011

H 2.937644 0.053554 0.000225

H 1.726288 -2.118685 0.000096

O -2.291156 -0.110815 0.000103

H -2.664420 0.774632 -0.000104

4 Results of the Calculations of all Silylenes

4.1 Energies

4.1.1 Energies of the Silylenes

Table S4.1. Energies of the Silylenes

Silylene	E(SCF)	Corr(H)	Corr(G)	ΔG [hartree]	ΔG [kJ/mol]
**$^{Tos}Y_2Si$**	-4082.96063973	0.886537	0.734124	–	–
**^{Tos}YSiCl**	-2646.96958766	0.446979	0.353325	–	–
**^{Tos}YSiHMDS**	-3060.76630909	0.691976	0.570221	–	–
**$^{Tos}YSiC_6F_5$**	-2915.29857635	0.504315	0.392825	–	–
**^{Tos}YSiPy**	-2434.44288792	0.528460	0.426992	–	–
**^{Tos}YSiPyr**	-2450.49912647	0.516491	0.415638	–	–
**^{Tos}YSiB2**	-2593.44571423	0.546337	0.442165	–	–
**^{Tos}YSiB1**	-2862.63192165	0.693337	0.574820	–	–
**$^{Ph}Y_2Si$**	-2905.76188502	0.799644	0.668877	–	–
**^{Ph}YSiCl**	-2058.37585009	0.404100	0.320216	–	–
**^{Ph}YSiHMDS**	-2472.17006049	0.648191	0.533817	–	–
**$^{Ph}YSiC_6F_5$**	-2326.70822592	0.461736	0.361813	–	–
**^{Ph}YSiPy**	-1845.84660072	0.485408	0.393326	–	–
**^{Ph}YSiPyr**	-1861.90830830	0.473476	0.381348	–	–
**^{Ph}YSiB2**	-2004.85620145	0.503131	0.407145	–	–
**^{Ph}YSiB1**	-2274.04404077	0.650821	0.540947	–	–
**$^{F}Y_2Si$**	-3899.58524078	0.727462	0.580705	–	–
**^{F}YSiCl**	-2555.28314520	0.367892	0.274602	–	–
**^{F}YSiHMDS**	-2969.07661631	0.612123	0.491482	–	–
**$^{F}YSiC_6F_5$**	-2823.61374882	0.425442	0.315421	–	–
**^{F}YSiPy**	-2342.75509713	0.449370	0.349815	–	–
**^{F}YSiPyr**	-2358.81572896	0.437384	0.337152	–	–
**^{F}YSiB2**	-2501.76361265	0.467031	0.362673	–	–
**^{F}YSiB1**	-2770.95300182	0.615011	0.499730	–	–
**I**	-1307.89084248	0.320586	0.254411	–	–
**II**	-1857.08091178	0.334564	0.261082	–	–
**III**	-2619.14154696	0.374215	0.293142	–	–
**IV**	-1827.11222845	0.332335	0.257908	–	–
**V**	-1277.88807407	0.318802	0.250747	–	–

Table S4.2. Energies of the silylenes in triplet state, energy of the singlet-triplet gap.

Silylene	E(SCF)	Corr(H)	Corr(G)	ΔG [hartree]	ΔG [kJ/mol]
**$^{Tos}Y_2Si$**	-4082.90541156	0.886824	0.733616	0.054720	143.667806
**^{Tos}YSiCl**	-2646.89377311	0.446893	0.350646	0.073136	192.017387
**^{Tos}YSiHMDS**	-3060.70706357	0.691624	0.568852	0.057877	151.954803
**$^{Tos}YSiC_6F_5$**	-2915.23607959	0.504296	0.390088	0.059760	156.899250
**^{Tos}YSiPy**	-2434.38897524	0.528269	0.425945	0.052866	138.798843
**^{Tos}YSiPyr**	-2450.44899349	0.516212	0.413639	0.048134	126.375764
**^{Tos}YSiB2**	-2593.40539726	0.546533	0.438202	0.036354	95.447348
**^{Tos}YSiB1**	-2862.58972114	0.694374	0.578104	0.045485	119.419581
**$^{Ph}Y_2Si$**	-2905.70713866	0.799180	0.671261	0.057130	149.995760
**^{Ph}YSiCl**	-2058.30841460	0.403431	0.318431	0.065650	172.365361
**^{Ph}YSiHMDS**	-2472.11061784	0.647579	0.534816	0.060442	158.689552
**$^{Ph}YSiC_6F_5$**	-2326.65839556	0.460921	0.360648	0.048665	127.770903
**^{Ph}YSiPy**	-1845.79625794	0.485086	0.391634	0.048651	127.732623
**^{Ph}YSiPyr**	-1861.85620551	0.473056	0.380073	0.050828	133.448363
**^{Ph}YSiB2**	-2004.81483694	0.503001	0.404643	0.038863	102.033520
**^{Ph}YSiB1**	-2274.00252995	0.650259	0.542314	0.042878	112.575716
**$^{F}Y_2Si$**	-3899.52249077	0.726016	0.577895	0.059940	157.372496
**^{F}YSiCl**	-2555.21180512	0.367510	0.273801	0.070539	185.200355
**^{F}YSiHMDS**	-2969.01618544	0.611819	0.490210	0.059159	155.321613
**$^{F}YSiC_6F_5$**	-2823.55664926	0.424819	0.314919	0.056598	148.596894
**^{F}YSiPy**	-2342.70134890	0.449165	0.346664	0.050597	132.843027
**^{F}YSiPyr**	-2358.76111520	0.436901	0.335832	0.053294	139.922767
**^{F}YSiB2**	-2501.72102022	0.467272	0.361705	0.041624	109.284941
**^{F}YSiB1**	-2770.90102294	0.613949	0.492151	0.044400	116.571885
**I**	-1307.79348567	0.319136	0.250584	0.093530	245.562516
**II**	-1856.99498746	0.333681	0.258977	0.083819	220.067625
**III**	-2619.08235191	0.374283	0.293764	0.059817	157.049665
**IV**	-1827.06921715	0.332030	0.255979	0.041082	107.861579
**V**	-1277.86649226	0.318281	0.248967	0.019802	51.989652

4.1.2 Energies of the H_2 activation

Pathway A

Table S4.3. Energies of the transition states of the H_2 activation following pathway A

Silylene	E(SCF)	Corr(H)	Corr(G)	ΔG [hartree]	ΔG [kJ/mol]	1M ΔG [kJ/mol]
$^{Tos}Y_2Si$						
$^{Tos}YSiCl$	-2648.09474676	0.460703	0.365522	0.063115	165.707297	157.781
$^{Tos}YSiHMDS$						
$^{Tos}YSiC_6F_5$	-2916.43414688	0.519119	0.411274	0.058955	154.786714	146.861
$^{Tos}YSiPy$	-2435.58864556	0.542606	0.440330	0.043657	114.621526	106.696
$^{Tos}YSiPyr$	-2451.64781124	0.530847	0.428157	0.039911	104.786062	96.860
$^{Tos}YSiB2$	n.o.	n.o.	n.o.	–	–	–
$^{Tos}YSiB1$	-2863.78984158	0.708304	0.592292	0.035629	93.543251	85.617
$^{Ph}Y_2Si$	-2906.90295520	0.813708	0.684995	0.051124	134.227342	126.301
$^{Ph}YSiCl$	-2059.50135336	0.417842	0.334144	0.064501	169.348419	161.423
$^{Ph}YSiHMDS$	-2473.30450431	0.661988	0.549815	0.057630	151.309790	143.384
$^{Ph}YSiC_6F_5$	-2327.84652376	0.475707	0.374760	0.050726	133.180660	125.255
$^{Ph}YSiPy$	-1846.99403111	0.499349	0.407866	0.043186	113.385572	105.460
$^{Ph}YSiPyr$	-1863.05446373	0.487207	0.395952	0.044525	116.901011	108.975
$^{Ph}YSiB2$	-2006.01208498	0.516718	0.419523	0.032571	85.515522	77.590
$^{Ph}YSiB1$	-2275.19632499	0.664403	0.554836	0.037681	98.932641	91.007
$^{F}Y_2Si$	-3900.70679059	0.741602	0.595646	0.069468	182.387860	174.462
$^{F}YSiCl$	-2556.40629877	0.381510	0.288899	0.067220	176.486366	168.560
$^{F}YSiHMDS$	-2970.20311110	0.626554	0.505759	0.063859	167.661483	159.736
$^{F}YSiC_6F_5$	-2824.73943180	0.439171	0.327440	0.062413	163.864511	155.939
$^{F}YSiPy$	-2343.88774585	0.463075	0.362416	0.056029	147.104002	139.178
$^{F}YSiPyr$	-2359.94742654	0.451125	0.350302	0.057529	151.042620	143.117
$^{F}YSiB2$	-2502.91460494	0.481004	0.376110	0.038521	101.137877	93.212
$^{F}YSiB1$	-2772.10305387	0.628397	0.509595	0.035890	94.228191	86.302
I	-1308.98949021	0.333195	0.265370	0.088184	232.062586	224.137
II	-1858.19991151	0.348197	0.273636	0.069470	182.816082	174.890
III	-2620.28203911	0.388190	0.307853	0.050179	132.050937	124.125
IV	-1828.26230535	0.345777	0.270661	0.038663	101.745447	93.820
V	n.o.	n.o.	n.o.	–	–	–

Table S4.4. Energies of the H_2 activated products following pathway **A**

Silylene	E(SCF)	Corr(H)	Corr(G)	ΔG [hartree]	ΔG [kJ/mol]	1M ΔG [kJ/mol]
**$^{Tos}Y_2Si$**	-4084.20221490	0.905735	0.756086	-0.043537	-114.305087	-122.231
**^{Tos}YSiCl**	-2648.19894660	0.465524	0.371191	-0.035416	-92.985423	-100.911
**^{Tos}YSiHMDS**	-3062.01021364	0.710441	0.588421	-0.049628	-130.298005	-138.224
**$^{Tos}YSiC_6F_5$**	-2916.53728533	0.523588	0.412583	-0.042874	-112.566507	-120.492
**^{Tos}YSiPy**	-2435.68222444	0.546615	0.442860	-0.047392	-124.427308	-132.353
**^{Tos}YSiPyr**	-2451.73835013	0.534822	0.431387	-0.047398	-124.443429	-132.369
**^{Tos}YSiB2**	-2594.69234496	0.564347	0.458131	-0.054588	-143.320958	-151.247
**^{Tos}YSiB1**	-2863.86877333	0.711715	0.590654	-0.044941	-117.992628	-125.919
**$^{Ph}Y_2Si$**	-2907.00165557	0.817788	0.687517	-0.045054	-118.288968	-126.215
**^{Ph}YSiCl**	-2059.61012190	0.422292	0.339833	-0.038578	-101.286913	-109.213
**^{Ph}YSiHMDS**	-2473.40958015	0.666935	0.553715	-0.043545	-114.327377	-122.253
**$^{Ph}YSiC_6F_5$**	-2327.93992590	0.479251	0.376858	-0.040578	-106.538359	-114.464
**^{Ph}YSiPy**	-1847.08712655	0.503605	0.412827	-0.044948	-118.011400	-125.937
**^{Ph}YSiPyr**	-1863.14632866	0.491315	0.399348	-0.043944	-115.374164	-123.300
**^{Ph}YSiB2**	-2006.09230180	0.520629	0.421314	-0.045855	-120.391469	-128.317
**^{Ph}YSiB1**	-2275.28042292	0.668346	0.558259	-0.042993	-112.879388	-120.805
**$^{F}Y_2Si$**	-3900.82263892	0.746355	0.601497	-0.040529	-106.410130	-114.336
**^{F}YSiCl**	-2556.51391356	0.385772	0.294033	-0.035261	-92.576948	-100.503
**^{F}YSiHMDS**	-2970.31815159	0.631347	0.510647	-0.046294	-121.543879	-129.470
**$^{F}YSiC_6F_5$**	-2824.84605413	0.443557	0.334097	-0.037553	-98.594463	-106.520
**^{F}YSiPy**	-2343.99344619	0.467164	0.367363	-0.044724	-117.423892	-125.350
**^{F}YSiPyr**	-2360.05064089	0.454835	0.352989	-0.042998	-112.891938	-120.818
**^{F}YSiB2**	-2502.99787334	0.484614	0.380128	-0.040729	-106.934048	-114.860
**^{F}YSiB1**	-2772.18989022	0.632859	0.515605	-0.044937	-117.981391	-125.907
**I**	-1309.09965466	0.337770	0.269667	-0.017439	-45.892404	-53.818
**II**	-1858.30978327	0.352567	0.278854	-0.034942	-91.952365	-99.878
**III**	-2620.39041897	0.393005	0.313967	-0.051850	-136.448079	-144.374
**IV**	-1828.35408176	0.349865	0.274635	-0.048936	-128.779781	-136.706
**V**	-1279.13297082	0.335689	0.266501	-0.052943	-139.324944	-147.251

Pathway B

Table S4.5. Energies of the transition states of the H_2 activation following pathway **B**

Silylene	E(SCF)	Corr(H)	Corr(G)	ΔG [hartree]	ΔG [kJ/mol]	1M ΔG [kJ/mol]
$^{Tos}Y_2Si$	-4084.06900568	0.899216	0.748627	0.082214	215.852116	207.926
^{Tos}YSiCl	-2648.09725670	0.461091	0.368316	0.063399	166.453097	158.527
^{Tos}YSiHMDS	-3061.88581469	0.705785	0.585043	0.071393	187.442499	179.517
^{Tos}YSiB1	-2863.77567360	0.707814	0.589464	0.046969	123.316368	115.390
$^{Ph}Y_2Si$	-2906.89499356	0.813815	0.684376	0.058467	153.505444	145.580
^{Ph}YSiCl	-2059.50608727	0.417814	0.334989	0.060612	159.138086	151.212
^{Ph}YSiHMDS	-2473.30116145	0.662058	0.548776	0.059935	157.358575	149.433
^{Ph}YSiB1	-2275.18420059	0.663695	0.553983	0.048953	128.525701	120.600
I	-1309.00443033	0.333177	0.265557	0.073465	193.328269	185.402
II	-1858.20497995	0.347771	0.273719	0.064496	169.726809	161.801
III	-2620.28368690	0.388604	0.308551	0.049232	129.557263	121.631
IV	-1828.25649934	0.345942	0.272193	0.045984	121.011392	113.085
V	-1279.04316670	0.332233	0.263485	0.033644	88.537265	80.611

Table S4.6. Energies of the H_2 activated products following pathway **B**

Silylene	E(SCF)	Corr(H)	Corr(G)	ΔG [hartree]	ΔG [kJ/mol]	1M ΔG [kJ/mol]
$^{Tos}Y_2Si$	-4084.10313370	0.905776	0.754707	0.054166	142.212039	134.286
^{Tos}YSiCl	-2648.14565424	0.466285	0.372292	0.018977	49.824344	41.898
^{Tos}YSiHMDS	-3061.94179912	0.710952	0.587763	0.018129	47.596738	39.671
^{Tos}YSiB1	-2863.81681644	0.713541	0.593515	0.009877	25.931742	18.006
$^{Ph}Y_2Si$	-2906.96124534	0.819298	0.690563	-0.001598	-4.194636	-12.121
^{Ph}YSiCl	-2059.57109201	0.423933	0.340646	0.001265	3.320595	-4.605
^{Ph}YSiHMDS	-2473.35955050	0.668840	0.556610	0.009380	24.626291	16.700
^{Ph}YSiB1	-2275.25060654	0.670602	0.562711	-0.008725	-22.907756	-30.834
I	-1309.07213660	0.339175	0.272356	0.012698	33.416232	25.490
II	-1858.27449606	0.353707	0.281431	0.002835	7.460119	-0.466
III	-2620.32742581	0.393862	0.314601	0.011630	30.605030	22.679
IV	-1828.30293237	0.350921	0.276831	0.004286	11.278541	3.353
V	-1279.10934965	0.337705	0.268261	-0.027621	-72.686682	-80.613

Pathway C

Table S4.7. Energies of the transition states of the H_2 activation following pathway **C**

Silylene	E(SCF)	Corr(H)	Corr(G)	ΔG [hartree]	ΔG [kJ/mol]	1M ΔG [kJ/mol]
**IV**	-1828.25649934	0.345942	0.272193	0.034538	90.888085	82.962
**V**	-1279.05823590	0.332770	0.263562	0.018687	49.175244	41.249

Table S4.8. Energies of the H_2 activated products following pathway **C**

Silylene	E(SCF)	Corr(H)	Corr(G)	ΔG [hartree]	ΔG [kJ/mol]	1M ΔG [kJ/mol]
**IV**	-1828.30293237	0.350921	0.276831	-0.01256	-33.053814	-40.980
**V**	-1279.12258772	0.336082	0.268223	-0.04087	-107.54300	-115.469

4.1.3 Energies of the phenol activation

Pathway A

Table S4.9. Energies of the coordination of phenol following pathway **A**

Silylene	E(SCF)	Corr(H)	Corr(G)	ΔG [hartree]	ΔG [kJ/mol]	1M ΔG [kJ/mol]
**I**	-1615.89086215	0.435071	0.352456	0.002612	6.874557	-1.051
**II**	n.o.	n.o.	n.o.	–	–	–
**III**	-2927.14122292	0.488028	0.388889	0.000663	1.743568	-6.182
**IV**	-2135.11326429	0.446833	0.357688	0.00333	8.761845	0.836
**V**	-1585.88590786	0.433235	0.348114	0.004117	10.833496	2.908

Table S4.10. Energies of the transition states of the phenol activation following pathway **A**

Silylene	E(SCF)	Corr(H)	Corr(G)	ΔG [hartree]	ΔG [kJ/mol]	1M ΔG [kJ/mol]
**I**	-1615.84108205	0.428558	0.346757	0.046592	122.609485	114.684
**II**	-2165.03765807	0.442926	0.355438	0.042105	110.803346	102.877
**III**	-2927.12149679	0.482668	0.387923	0.019379	50.998290	43.072
**IV**	-2135.08344927	0.440775	0.354033	0.029429	77.444977	69.519
**V**	-1585.85963685	0.426798	0.344675	0.026896	70.778938	62.853

Table 4.11. Energies of the phenol activated products following pathway **A**

Silylene	E(SCF)	Corr(H)	Corr(G)	ΔG [hartree]	ΔG [kJ/mol]	1M ΔG [kJ/mol]
I	-1615.95541074	0.432376	0.353092	-0.061153	-160.927948	-168.854
II	-2165.15463655	0.447029	0.358073	-0.071974	-189.405460	-197.331
III	-2927.23442788	0.486974	0.392826	-0.088399	-232.629461	-240.555
IV	-2135.20117430	0.444622	0.359088	-0.082980	-218.370186	-226.296
V	-1585.97718445	0.430637	0.350234	-0.084834	-223.247131	-231.173

Pathway B

Table S4.12. Energies of the coordination of phenol following pathway **B**

Silylene	E(SCF)	Corr(H)	Corr(G)	ΔG [hartree]	ΔG [kJ/mol]	1M ΔG [kJ/mol]
I	-1615.89171800	0.435087	0.352579	0.001881	4.950459	-2.975
II	-2165.07934981	0.447891	0.358799	0.003863	10.165989	2.240
III	-2927.12121346	0.483198	0.388404	-0.005031	-13.240239	-21.166
IV	-2135.11433287	0.446627	0.359477	0.004048	10.653308	2.727
V	-1585.88951218	0.433138	0.348437	0.000843	2.218390	-5.708

Table S4.13. Energies of the transition states of the phenol activation following pathway **B**

Silylene	E(SCF)	Corr(H)	Corr(G)	ΔG [hartree]	ΔG [kJ/mol]	1M ΔG [kJ/mol]
I	-1615.86250014	0.429071	0.348587	0.027049	71.180954	63.255
II	-2165.06210801	0.443048	0.357384	0.019653	51.719252	43.793
III	-2927.12121346	0.483198	0.388404	0.020142	53.005038	45.079
IV	-2135.09967690	0.440763	0.352772	0.011981	31.528579	23.603
V	-1585.87884112	0.427817	0.346803	0.009859	25.945191	18.019

Table S 4.14. Energies of the phenol activated products following pathway **B**

Silylene	E(SCF)	Corr(H)	Corr(G)	ΔG [hartree]	ΔG [kJ/mol]	1M ΔG [kJ/mol]
I	-1615.91712301	0.433832	0.354461	-0.021587	-56.809204	-64.735
II	-2165.11660870	0.447510	0.361462	-0.030653	-80.665521	-88.591
III	-2927.16806522	0.488048	0.393623	-0.021395	-56.301773	-64.228
IV	-2135.13462731	0.444999	0.358099	-0.017574	-46.247684	-54.174
V	-1585.94278554	0.432488	0.350243	-0.050506	-132.909164	-140.835

Pathway C

Table S4.15. Energies of the coordination of phenol following pathway **C**

Silylene	E(SCF)	Corr(H)	Corr(G)	ΔG [hartree]	ΔG [kJ/mol]	1M ΔG [kJ/mol]
IV	-2135.09391264	0.447376	0.357676	0.022678	59.540955	51.615
V	-1585.88590781	0.433235	0.348118	0.004121	10.844129	2.918

Table S4.16. Energies of the transition states of the phenol activation following pathway **C**

Silylene	E(SCF)	Corr(H)	Corr(G)	ΔG [hartree]	ΔG [kJ/mol]	1M ΔG [kJ/mol]
IV	-2135.06325820	0.440853	0.352500	0.048156	126.434599	118.509
V	-1585.85623114	0.427223	0.347568	0.033180	87.316201	79.390

Table S4.17. Energies of the phenol activated products following pathway **C**

Silylene	E(SCF)	Corr(H)	Corr(G)	ΔG [hartree]	ΔG [kJ/mol]	1M ΔG [kJ/mol]
IV	-2135.14675609	0.444228	0.356903	-0.030939	-81.229034	-89.155
V	-1585.95026060	0.430044	0.350986	-0.057222	-150.584187	-158.510

4.2 Coordinates of the Structures

4.2.1 Singlet state structures

$^{Tos}Y_2Si$
E = -4082.96063973
C -4.425165 -2.557985 -0.380552
H -4.395470 -2.848330 0.666705
C -3.331163 -1.913870 -0.932643
C 3.223866 1.929205 -0.890726
O -0.710318 -2.007647 -0.667189
C 3.142770 1.625396 -2.247317
H 2.239652 1.154278 -2.640155
C -2.700327 2.944338 2.861427
H -3.690690 3.234250 2.511102
C -5.538874 -2.798881 -1.180004
H -6.407106 -3.297862 -0.751507
C 1.989447 -5.074160 -1.364776
H 1.852903 -6.110086 -1.061009
C 2.141560 -4.087932 -0.397627
H 2.112651 -4.351279 0.657035
C -2.344844 2.454986 -2.067598
H -2.456582 1.411878 -2.357352
C -2.295711 2.793845 -0.712804
C -5.093393 1.186168 -0.364373
H -4.791033 1.719292 -1.263216
C -6.387076 0.692691 -0.250498
H -7.094319 0.841372 -1.064044

C 1.995416 -4.737621 -2.714388
H 1.867165 -5.512372 -3.468100
C 1.482677 -0.137344 -0.121882
Si -0.058568 -0.193544 -1.280732
O 2.336417 1.755843 1.544963
S 1.861980 1.470515 0.177717
P 2.465423 -1.405791 0.415581
C 4.216105 1.920344 -3.071490
H 4.159465 1.680988 -4.133087
C 4.676688 -0.306530 1.689877
H 3.996814 -0.142108 2.522592
C 0.748300 -1.851099 2.503280
H 0.081799 -1.225278 1.917226
C -5.867019 -0.186274 1.932735
H -6.166025 -0.727675 2.828059
C -4.570244 0.302106 1.825225
H -3.851849 0.129253 2.624433
C 2.301912 -2.419669 -2.139625
H 2.402022 -1.375583 -2.433040
C -1.909550 2.075242 2.103024
C -3.322312 -1.518431 -2.267782
H -2.439151 -1.023268 -2.678511
C -2.089872 4.118264 -0.330625
H -2.003502 4.377038 0.721715
C -4.182816 0.999931 0.677763
C 2.494247 -3.429520 3.989482
H 3.177621 -4.039709 4.577202
C 2.036255 -2.095819 2.036804
C 2.915575 -2.886759 2.782059
H 3.927060 -3.070493 2.421540
C 2.308382 -2.758444 -0.782581
C 2.150692 -3.410828 -3.101053
H 2.139947 -3.141793 -4.155190
C -6.780035 -2.631943 -3.367428
H -6.516921 -3.126987 -4.310239
H -7.263130 -1.680876 -3.629152
H -7.520991 -3.252104 -2.852739
C 1.197892 -3.195716 4.446021
H 0.871230 -3.630054 5.389346
C 5.375490 2.521665 -2.562861
C -6.775958 0.011304 0.898674
H -7.790434 -0.373378 0.985433
C 6.389177 -0.658121 -0.479871
H 7.054754 -0.791292 -1.330376
C -1.552210 0.124084 -0.080043
P -2.433237 1.452218 0.490277
O -2.227050 -2.047896 1.411063
S -1.941986 -1.470195 0.093397
O 0.676717 2.212844 -0.329334
C -5.568323 -2.401570 -2.515524
C -4.438266 -1.765327 -3.048453
H -4.439159 -1.460262 -4.094305
C -2.220914 3.446117 -3.032451
H -2.252657 3.179138 -4.086692
C -2.033529 4.770138 -2.650081
H -1.925395 5.544483 -3.407338
C -1.964523 5.103880 -1.301622
H -1.796107 6.136310 -1.002395
C 5.080466 -1.118070 -0.551479
H 4.728325 -1.610793 -1.454817

C 0.324016 -2.410957 3.702297
H -0.694681 -2.232629 4.039137
C 4.221120 -0.951897 0.536959
C 6.845142 -0.029361 0.674532
H 7.871174 0.330035 0.728780
C 5.418829 2.832748 -1.205079
H 6.311238 3.302813 -0.793090
C 5.988425 0.147552 1.755607
H 6.339255 0.649536 2.655128
C -0.948403 3.056661 4.513819
H -0.571054 3.448729 5.456740
C -2.217307 3.428106 4.070317
H -2.829421 4.100404 4.668564
C 4.349329 2.539368 -0.363513
H 4.379112 2.757217 0.701355
C 6.542587 2.802556 -3.461513
H 7.296371 3.418021 -2.959470
H 6.229439 3.324676 -4.373971
H 7.030531 1.870259 -3.777376
C -0.166718 2.189643 3.759614
H 0.831917 1.904904 4.082191
C -0.653802 1.687154 2.559355
H -0.061715 0.996103 1.967751

^{Tos}YSiCl
E = -2646.96958766
C -3.203311 0.720140 -1.296143
H -2.835752 0.852505 -2.310816
C -2.415484 1.127445 -0.233191
O -0.787209 3.127641 0.246068
C 2.767483 -1.625334 -1.802360
H 2.273881 -2.573977 -1.592477
C -4.440905 0.140814 -1.031787
H -5.065274 -0.187908 -1.861314
C 0.972031 -0.863245 2.778034
H 0.171193 -0.126233 2.769808
C 1.602935 -1.209260 1.579237
C -1.191531 -2.171828 0.189103
H -1.039054 -2.144782 1.266729
C -2.280205 -2.849443 -0.342533
H -2.977847 -3.355960 0.321198
Si 0.508276 2.616108 1.593509
C -1.595636 -2.216123 -2.565963
H -1.758069 -2.224597 -3.641807
C -0.506623 -1.529243 -2.040950
H 0.166123 -0.985405 -2.700700
C 2.346364 -0.454016 -1.162565
C -2.841907 0.979558 1.083670
H -2.201835 1.313936 1.901415
C 2.657712 -2.124347 1.587404
H 3.172627 -2.377265 0.663912
C -0.293243 -1.521210 -0.660373
C -6.219397 -0.668087 0.560947
H -6.866245 0.007692 1.134517
H -6.099872 -1.580603 1.159347
H -6.744043 -0.935551 -0.361716
C -2.477716 -2.879073 -1.720371
H -3.330849 -3.412695 -2.134912
C 0.349333 1.061087 0.421004
P 0.998449 -0.459195 0.045899

O -0.632501 1.847648 -1.990301
S -0.792190 1.791178 -0.540358
C -4.892514 -0.032617 0.275202
C -4.074691 0.401213 1.328469
H -4.416667 0.282495 2.355805
C 1.388834 -1.440730 3.971678
H 0.901485 -1.161152 4.903261
C 2.431935 -2.360191 3.975110
H 2.759617 -2.807611 4.911497
C 3.066732 -2.698995 2.784079
H 3.891861 -3.408126 2.787037
C 4.412310 -0.353572 -3.016168
H 5.220895 -0.313515 -3.743704
C 3.802395 -1.572307 -2.726006
H 4.129549 -2.481026 -3.227368
C 3.983447 0.810856 -2.390032
H 4.451694 1.764384 -2.624585
C 2.945733 0.768476 -1.464304
H 2.594956 1.679249 -0.979096
Cl 2.222193 3.608094 0.712871

^{Tos}YSiHMDS
E = -3060.76630909
S 0.600033 -1.597672 0.514769
P 0.624320 1.314130 0.158564
Si -3.848885 -0.010115 -1.184397
Si -3.330896 -2.412094 0.618466
O -0.295001 -2.571787 -0.294350
O 0.586283 -1.761831 1.969902
N -2.760287 -1.255275 -0.586120
C 0.047948 -0.228543 -0.220436
C 2.246985 -2.015995 -0.018597
C 2.531411 -1.982813 -1.381093
H 1.735499 -1.757059 -2.093795
C 3.823371 -2.243922 -1.802434
H 4.054890 -2.223709 -2.866671
C 4.839484 -2.536944 -0.881448
C 4.517733 -2.574924 0.474217
H 5.294744 -2.805406 1.201721
C 3.223384 -2.315828 0.915659
H 2.961999 -2.331968 1.970944
C 6.237506 -2.796418 -1.355505
H 6.261171 -3.602043 -2.099830
H 6.896380 -3.079547 -0.528514
H 6.662894 -1.905092 -1.835402
C 2.326810 1.260919 0.783557
C 3.406069 1.318012 -0.101074
H 3.234510 1.513206 -1.158067
C 4.697736 1.123901 0.371119
H 5.537429 1.170018 -0.319323
C 4.915790 0.869726 1.722066
H 5.929205 0.719277 2.089269
C 3.840575 0.795937 2.601190
H 4.008878 0.583945 3.655053
C 2.544591 0.985103 2.136355
H 1.700889 0.902491 2.818870
C -0.333776 2.161408 1.443481
C 0.133324 3.300799 2.106610
H 1.105129 3.723768 1.853144
C -0.633830 3.875832 3.111283

H -0.271567 4.760374 3.631413
C -1.858415 3.310971 3.461856
H -2.452994 3.758437 4.256053
C -2.314961 2.170127 2.812045
H -3.262598 1.718402 3.098573
C -1.553941 1.590464 1.802436
H -1.882945 0.682606 1.295623
C 0.600894 2.324145 -1.344633
C 0.798483 1.671750 -2.565101
H 0.928117 0.591112 -2.580228
C 0.786032 2.400877 -3.748091
H 0.928988 1.888563 -4.697136
C 0.573225 3.774933 -3.718745
H 0.555457 4.342583 -4.647150
C 0.369551 4.423942 -2.504717
H 0.190830 5.496949 -2.482051
C 0.380935 3.702290 -1.317635
H 0.197364 4.211157 -0.374306
C -4.877319 0.775556 0.187336
H -5.583063 1.486466 -0.264018
H -4.233235 1.344243 0.868191
H -5.458034 0.066857 0.786073
C -2.841308 1.413682 -1.896787
H -3.521469 2.192665 -2.267520
H -2.189400 1.113410 -2.726953
H -2.213328 1.869903 -1.120768
C -4.979921 -0.663487 -2.533161
H -5.636885 -1.458812 -2.162763
H -4.375042 -1.081640 -3.348164
H -5.610037 0.134114 -2.948739
C -3.075446 -1.702419 2.339884
H -3.608989 -0.750787 2.463238
H -2.004197 -1.524159 2.503558
H -3.426754 -2.392901 3.118006
C -2.520989 -4.104752 0.540034
H -3.203381 -4.834863 0.996408
H -1.565963 -4.133183 1.071122
H -2.332502 -4.415126 -0.495054
C -5.160948 -2.788881 0.336551
H -5.279089 -3.344897 -0.603116
H -5.827693 -1.921902 0.296553
H -5.512706 -3.439720 1.148629
Si -1.166789 -1.273058 -1.430966

$^{Tos}YSiC_6F_5$
E = -2915.29857635
C 3.342128 -2.075227 -1.252367
H 2.898207 -2.068113 -2.244826
C 2.524853 -1.932399 -0.144145
O 0.121398 -2.693450 0.589264
C -0.590431 3.127643 -1.832081
H 0.188264 3.814247 -1.501244
C 4.713524 -2.215444 -1.061176
H 5.365526 -2.323261 -1.926723
C 0.938059 1.845512 2.626774
H 1.445966 0.882583 2.626761
C 0.338748 2.310280 1.450777
C 3.259182 1.544112 0.029979
H 3.157848 1.664832 1.106391
C 4.521823 1.499858 -0.544261

H 5.404530 1.588149 0.085723
C 3.528527 1.215775 -2.721771
H 3.631989 1.079255 -3.796272
C 2.259981 1.253709 -2.152737
H 1.377780 1.128734 -2.777267
C -0.672201 1.839592 -1.293188
C 3.045310 -1.935254 1.147685
H 2.376377 -1.824037 2.002447
C -0.383565 3.503759 1.466515
H -0.902088 3.846139 0.575689
C 2.122171 1.433058 -0.774250
C 6.745923 -2.353761 0.422871
H 6.977469 -3.225109 1.048282
H 7.158515 -1.474418 0.934310
H 7.274806 -2.470432 -0.528294
C 4.657320 1.342725 -1.921085
H 5.648664 1.309304 -2.368869
C 0.259568 -0.336343 0.503774
P 0.478901 1.260779 -0.017573
O 0.513285 -1.764740 -1.788989
S 0.781847 -1.653416 -0.355760
C 5.268052 -2.214011 0.217457
C 4.410956 -2.076970 1.318819
H 4.827205 -2.083837 2.325329
C 0.848043 2.592715 3.794323
H 1.311930 2.223721 4.706500
C 0.147819 3.794253 3.800225
H 0.070382 4.374331 4.717723
C -0.472949 4.243279 2.639699
H -1.043304 5.169682 2.648447
C -2.482477 2.643789 -3.242861
H -3.198887 2.963506 -3.997067
C -1.499462 3.527637 -2.802478
H -1.441511 4.531396 -3.218619
C -2.539284 1.351303 -2.735366
H -3.293281 0.653074 -3.092783
C -1.625990 0.942703 -1.769564
H -1.629162 -0.077606 -1.393519
Si -0.617656 -1.438598 1.861514
C -2.401934 -1.411632 1.049255
C -4.334066 -2.477580 0.022577
C -4.255704 -0.077426 0.190250
C -4.913495 -1.235142 -0.208253
C -3.032185 -0.198044 0.831189
C -3.096906 -2.545248 0.655228
F -2.578039 -3.752361 0.858460
F -2.413568 0.937570 1.191961
F -4.970083 -3.575506 -0.358550
F -6.086406 -1.153560 -0.817112
F -4.787307 1.110902 -0.064825

^{Tos}YSiPy
E = -2434.44288792
C -3.244091 -1.579776 1.074514
H -2.812831 -1.767016 2.054936
C -2.418361 -1.569107 -0.036472
O -0.309152 -2.899109 -0.853411
C 1.823974 2.240582 2.105955
H 1.173553 3.097201 1.930539
C -4.603977 -1.337673 0.902927

H -5.260973 -1.337429 1.771555
C 0.278102 1.832844 -2.558274
H -0.355296 0.958959 -2.699165
C 0.843470 2.078494 -1.302758
C -2.175853 1.966225 0.058579
H -2.027246 2.132891 -1.006990
C -3.414000 2.213813 0.634711
H -4.232606 2.580932 0.019025
C -2.566126 1.502640 2.776069
H -2.720222 1.310468 3.835965
C -1.324367 1.246056 2.204207
H -0.519180 0.834696 2.809160
C 1.674905 1.075288 1.345010
C -2.921945 -1.332041 -1.312993
H -2.245280 -1.330428 -2.168717
C 1.704176 3.163709 -1.130323
H 2.180189 3.342381 -0.170185
C -1.121793 1.494596 0.844982
C -6.600396 -0.810360 -0.542354
H -7.054614 -1.523817 -1.241381
H -6.763034 0.192490 -0.958510
H -7.143328 -0.873761 0.406035
C -3.607488 1.989830 1.995435
H -4.579325 2.184879 2.444861
C 0.175386 -0.583413 -0.632941
P 0.415780 0.946237 0.044153
O -0.426111 -2.047647 1.574447
S -0.661442 -1.778648 0.154952
C -5.138795 -1.087311 -0.359407
C -4.276504 -1.094423 -1.465223
H -4.680023 -0.909350 -2.459949
C 0.556435 2.682188 -3.622132
H 0.121239 2.480537 -4.598565
C 1.401577 3.771726 -3.442883
H 1.623370 4.432017 -4.279114
C 1.977871 4.008211 -2.199214
H 2.653823 4.849502 -2.060950
C 3.591928 1.187316 3.356188
H 4.344505 1.232418 4.141270
C 2.785397 2.296060 3.105403
H 2.902026 3.202174 3.696687
C 3.425057 0.021058 2.619660
H 4.039371 -0.853686 2.822395
C 2.462353 -0.041481 1.617217
H 2.299822 -0.961025 1.061017
Si 0.794622 -1.798271 -2.056291
C 2.450523 -2.236467 -1.179101
N 3.391385 -1.325908 -1.482224
C 3.942844 -3.344898 0.347878
C 4.574455 -1.400244 -0.886340
C 4.904628 -2.388195 0.037792
H 4.160230 -4.135129 1.066138
H 5.305931 -0.632849 -1.152343
H 5.891236 -2.405228 0.496692
C 2.699956 -3.270070 -0.268644
H 1.906696 -3.976872 -0.031398

^{Tos}YSiPyr
E = -2450.49912647
C -3.395518 -1.328597 1.114875

H -2.992534 -1.415620 2.120855
C -2.552510 -1.505545 0.030952
O -0.455028 -2.969467 -0.595299
C 1.916606 2.138277 2.073010
H 1.398428 3.053907 1.789199
C -4.735175 -1.030666 0.883020
H -5.404648 -0.884107 1.729472
C 0.544333 1.589360 -2.622205
H -0.173911 0.780481 -2.743025
C 1.069009 1.861840 -1.354937
C -2.047809 2.046601 -0.170917
H -1.841665 2.090996 -1.239064
C -3.292516 2.423075 0.312572
H -4.057889 2.771174 -0.378014
C -2.588729 1.887822 2.554740
H -2.802593 1.813393 3.619078
C -1.340307 1.502214 2.075929
H -0.592211 1.106983 2.759504
C 1.655054 0.942154 1.394784
C -3.019424 -1.397510 -1.276670
H -2.330512 -1.537839 -2.110784
C 2.035966 2.856806 -1.205054
H 2.481621 3.051585 -0.233787
C -1.060969 1.598540 0.711698
C -6.673240 -0.576414 -0.663395
H -7.166112 -1.367457 -1.242818
H -6.770932 0.351357 -1.241848
H -7.225680 -0.450671 0.273242
C -3.561013 2.351780 1.677523
H -4.538275 2.646959 2.054756
C 0.085960 -0.665042 -0.573654
P 0.458781 0.867689 0.029777
O -0.630556 -1.874835 1.743527
S -0.811690 -1.755302 0.295901
C -5.233164 -0.907844 -0.412680
C -4.354102 -1.100447 -1.488214
H -4.728775 -1.014705 -2.507464
C 0.967917 2.325442 -3.721506
H 0.561976 2.102471 -4.705880
C 1.919390 3.327515 -3.566272
H 2.254616 3.898958 -4.429712
C 2.455395 3.588218 -2.309662
H 3.212910 4.359746 -2.187774
C 3.448983 0.976163 3.521575
H 4.153484 0.989793 4.351156
C 2.817313 2.154560 3.129180
H 3.020926 3.085588 3.654478
C 3.165618 -0.217913 2.869907
H 3.639841 -1.144166 3.187387
C 2.263703 -0.240532 1.811142
H 2.008031 -1.178070 1.323206
Si 0.659845 -2.010251 -1.878938
C 2.390778 -2.225107 -1.047314
N 2.796244 -3.259554 -0.304168
N 3.147201 -1.143337 -1.316057
C 4.015177 -3.188567 0.221442
C 4.360102 -1.087028 -0.792366
C 4.859068 -2.104621 0.015909
H 4.335374 -4.034921 0.835333
H 4.958532 -0.198961 -1.012952

H 5.852684 -2.056401 0.454804

^{Tos}YSiB2
E = -2593.44571423
C -3.349642 -2.087111 0.896981
H -2.821079 -2.380777 1.800549
C -2.624377 -1.777209 -0.240834
O -0.441869 -2.621745 -1.422304
C 1.350537 2.205368 2.256819
H 0.634103 3.026350 2.260695
C -4.738330 -2.005030 0.851379
H -5.317639 -2.241957 1.742674
C -0.687830 2.345891 -2.215417
H -1.260031 1.445354 -2.432411
C 0.008206 2.445071 -1.005218
C -2.843936 1.680100 0.520214
H -2.820125 2.041228 -0.506250
C -4.042662 1.654167 1.218498
H -4.955167 2.001383 0.738246
Si 0.346444 -1.178739 -2.496594
C -2.913778 0.721272 3.133451
H -2.941684 0.336396 4.150829
C -1.708883 0.740588 2.438318
H -0.803547 0.354891 2.902389
C 1.196907 1.136919 1.365517
C -3.255134 -1.395502 -1.422824
H -2.656093 -1.160293 -2.304086
C 0.797096 3.568013 -0.750328
H 1.376366 3.638105 0.165839
C -1.669454 1.236769 1.133623
C -6.895421 -1.519480 -0.358808
H -7.311805 -2.184303 -1.126217
H -7.217180 -0.500500 -0.610178
H -7.346183 -1.788931 0.601626
C -4.076810 1.181588 2.528218
H -5.018376 1.162055 3.073683
C -0.227985 -0.348448 -0.780722
P -0.150924 1.060153 0.152053
O -0.469488 -2.248168 1.138684
S -0.845754 -1.773254 -0.193288
C -5.400579 -1.617872 -0.312204
C -4.636520 -1.319092 -1.449492
H -5.139243 -1.023573 -2.369560
C -0.614801 3.376035 -3.144617
H -1.152461 3.289357 -4.086364
C 0.158233 4.501267 -2.879338
H 0.220286 5.304015 -3.611573
C 0.867183 4.592908 -1.686227
H 1.488292 5.463268 -1.485040
C 3.314290 1.145427 3.168213
H 4.150566 1.156639 3.864671
C 2.410869 2.207273 3.153048
H 2.533634 3.038560 3.844636
C 3.141054 0.066560 2.310606
H 3.835295 -0.771422 2.323973
C 2.072844 0.055680 1.419485
H 1.891407 -0.805405 0.781537
C 4.200750 -0.804391 -0.940209
C 3.928644 -2.094817 -0.490651
C 5.352725 -0.134038 -0.577720

C 4.798595 -2.776795 0.338524
C 6.238429 -0.813787 0.262425
H 5.547108 0.876122 -0.928006
C 5.968679 -2.107481 0.709496
H 4.571324 -3.782209 0.682739
H 7.158574 -0.323111 0.573082
H 6.683117 -2.607214 1.360519
B 2.220726 -1.414747 -1.720121
O 3.165538 -0.379890 -1.709261
O 2.720720 -2.484852 -0.975181

TosYSiB1

E = -2862.63192165
C -4.031388 -0.153484 0.579168
H -3.823801 -0.606409 1.545580
C -3.068520 -0.208259 -0.413805
O -1.316891 -1.950387 -1.259697
C 1.759870 1.866074 2.767456
H 1.501708 2.916218 2.644221
C -5.235228 0.491696 0.311133
H -5.997655 0.545765 1.087006
C 0.675885 2.578687 -1.989599
H -0.022724 1.827049 -2.353891
C 1.081718 2.547475 -0.650300
C -1.959866 3.016210 0.392085
H -1.628176 3.297749 -0.605843
C -3.151565 3.517236 0.896957
H -3.750254 4.197096 0.294144
Si 0.251259 -1.110092 -2.101224
C -2.823870 2.263661 2.929839
H -3.165617 1.960665 3.917493
C -1.629218 1.756084 2.429676
H -1.048937 1.046967 3.016126
C 1.300378 0.908941 1.854831
C -3.284298 0.356451 -1.668477
H -2.505337 0.290762 -2.429574
C 2.014414 3.477338 -0.190136
H 2.377372 3.437870 0.831796
C -1.187129 2.144356 1.163567
C -6.768414 1.788155 -1.212326
H -7.264239 1.374255 -2.099220
H -6.595265 2.854099 -1.411092
H -7.462720 1.711922 -0.369460
C -3.580851 3.146319 2.168818
H -4.516123 3.540017 2.562314
C -0.233655 0.038788 -0.541789
P 0.264371 1.335933 0.421944
O -1.555074 -1.500630 1.266423
S -1.486505 -0.954423 -0.091525
C -5.479553 1.076994 -0.929897
C -4.486997 0.993714 -1.917139
H -4.666879 1.437414 -2.895608
C 1.184428 3.547252 -2.846198
H 0.870211 3.559822 -3.887841
C 2.092387 4.490050 -2.375812
H 2.485338 5.251224 -3.047323
C 2.509389 4.450062 -1.050440
H 3.236519 5.171615 -0.683594
C 2.793159 0.124156 4.070995
H 3.383880 -0.181343 4.932727

C 2.515546 1.474164 3.863427
H 2.877648 2.221459 4.566836
C 2.293744 -0.831357 3.195040
H 2.485106 -1.889204 3.359157
C 1.542980 -0.443511 2.089178
H 1.129570 -1.190646 1.415198
N 2.917606 -1.678195 -0.549172
C 2.561124 -3.557363 0.605645
C 3.430784 -2.543569 0.411738
H 2.674267 -4.437130 1.229111
H 4.410988 -2.403391 0.851955
B 1.619980 -2.169319 -0.987083
C 0.380338 -4.292950 -0.232614
C -0.200327 -4.671223 -1.442801
C -0.120219 -4.814285 0.960127
C -1.273127 -5.550047 -1.457302
H 0.194705 -4.256745 -2.368646
C -1.184771 -5.706698 0.938560
H 0.307818 -4.489261 1.905971
C -1.766878 -6.078177 -0.268150
H -1.724078 -5.829001 -2.407717
H -1.572629 -6.100713 1.876229
H -2.605468 -6.771452 -0.282107
C 3.523290 -0.459127 -0.877748
C 3.374974 0.070038 -2.163865
C 4.292695 0.244558 0.057035
C 3.983443 1.270681 -2.505122
H 2.798866 -0.482284 -2.904425
C 4.914940 1.433472 -0.299993
H 4.386747 -0.135416 1.071643
C 4.767800 1.953239 -1.581765
H 3.851504 1.667414 -3.509961
H 5.513131 1.963532 0.439428
H 5.250984 2.888540 -1.855914
N 1.457504 -3.386870 -0.225218

$^{Ph}Y_2Si$
E = -2905.76188502
P 2.954755 0.325352 -0.009650
P -2.934264 0.459325 -0.055550
C 1.487190 -0.465577 -0.423282
C 1.551602 -1.919561 -0.644785
C 2.449795 -2.774469 0.019350
H 3.129582 -2.368268 0.765728
C 2.478726 -4.140739 -0.233878
H 3.188128 -4.768149 0.304236
C 1.603312 -4.708018 -1.151751
H 1.616685 -5.779402 -1.341877
C 0.703496 -3.881360 -1.820565
H 0.001130 -4.306411 -2.535895
C 0.686600 -2.517036 -1.579410
H -0.023823 -1.879896 -2.103735
C 3.526514 -0.006153 1.704858
C 4.809744 -0.430935 2.050236
H 5.571445 -0.557044 1.284975
C 5.115198 -0.721206 3.376794
H 6.116236 -1.060502 3.636353
C 4.147231 -0.585790 4.365074
H 4.389571 -0.815992 5.400858
C 2.862533 -0.170440 4.024676

H 2.096493 -0.076263 4.791980
C 2.550426 0.105005 2.700198
H 1.537160 0.397813 2.418365
C 2.815655 2.135283 -0.118082
C 2.986948 2.982094 0.973874
H 3.185269 2.571755 1.960857
C 2.887023 4.360593 0.804195
H 3.009511 5.018395 1.662541
C 2.631367 4.893194 -0.452288
H 2.551088 5.971020 -0.581029
C 2.485145 4.047593 -1.550415
H 2.288823 4.462056 -2.537117
C 2.578993 2.675476 -1.386263
H 2.448297 2.007473 -2.236101
C 4.377854 -0.027612 -1.095089
C 4.266451 -0.936847 -2.147553
H 3.340889 -1.486899 -2.292095
C 5.338606 -1.130597 -3.012820
H 5.246601 -1.845695 -3.827805
C 6.517342 -0.413828 -2.842318
H 7.352788 -0.568208 -3.522780
C 6.623988 0.513527 -1.809570
H 7.537142 1.092355 -1.685036
C 5.556932 0.710181 -0.942596
H 5.631597 1.454097 -0.149675
C -1.449220 -0.389033 0.136096
C -1.547224 -1.835327 0.403346
C -0.736667 -2.421323 1.389200
H -0.053518 -1.780218 1.944827
C -0.765681 -3.785706 1.633133
H -0.107640 -4.208943 2.390455
C -1.624878 -4.615026 0.915633
H -1.647378 -5.685633 1.109891
C -2.442512 -4.057610 -0.059700
H -3.110998 -4.691783 -0.640753
C -2.394670 -2.692341 -0.316562
H -2.999111 -2.280872 -1.124825
C -4.262166 -0.205643 1.010499
C -4.324597 0.185952 2.349679
H -3.644654 0.945498 2.727032
C -5.257203 -0.383820 3.207256
H -5.297835 -0.065337 4.246985
C -6.133824 -1.356735 2.738937
H -6.862038 -1.805114 3.412146
C -6.076046 -1.755311 1.408861
H -6.754383 -2.520196 1.036275
C -5.146137 -1.183892 0.548232
H -5.111520 -1.504223 -0.489748
C -2.770942 2.226242 0.370860
C -3.367396 3.232245 -0.386764
H -3.913062 2.986388 -1.293575
C -3.245243 4.564532 -0.001195
H -3.706124 5.342901 -0.606160
C -2.531783 4.899095 1.141907
H -2.434269 5.941821 1.438582
C -1.932499 3.898183 1.902496
H -1.357724 4.154791 2.790088
C -2.045148 2.571256 1.516787
H -1.545632 1.787019 2.081799
C -3.676098 0.443969 -1.732574

C -5.013307 0.777216 -1.971789
H -5.665804 1.038185 -1.139398
C -5.511844 0.770414 -3.268452
H -6.553290 1.029224 -3.450280
C -4.680607 0.429668 -4.333057
H -5.074475 0.420888 -5.347766
C -3.351297 0.096492 -4.099782
H -2.701052 -0.173285 -4.929559
C -2.850058 0.102580 -2.801960
H -1.811771 -0.161474 -2.598269
Si 0.030041 0.667488 -0.217898

^{Ph}YSiCl
E = -2058.37585009
P 0.535194 -0.035511 -0.022218
C -0.727790 1.128310 -0.165787
C -2.093722 0.547478 -0.121032
C -2.709052 0.219785 1.094066
H -2.185316 0.432766 2.024045
C -3.967498 -0.370316 1.124510
H -4.424334 -0.617457 2.081511
C -4.644777 -0.636914 -0.061192
H -5.630782 -1.097377 -0.038411
C -4.056152 -0.297446 -1.275439
H -4.582042 -0.491335 -2.208897
C -2.796322 0.287273 -1.304012
H -2.331172 0.544083 -2.253808
C 0.760286 -0.680100 1.669795
C 1.161157 -1.993524 1.923328
H 1.309529 -2.688155 1.098918
C 1.367068 -2.414371 3.232692
H 1.673415 -3.440012 3.428681
C 1.179754 -1.528015 4.288652
H 1.338671 -1.861679 5.312358
C 0.785518 -0.216969 4.038082
H 0.637360 0.477194 4.862782
C 0.572907 0.206071 2.732036
H 0.252489 1.226692 2.521435
C 2.136568 0.660141 -0.504501
C 3.275662 0.490218 0.282666
H 3.211063 -0.041433 1.229245
C 4.494713 1.010120 -0.139481
H 5.378778 0.882144 0.481605
C 4.582134 1.693016 -1.346994
H 5.537279 2.099972 -1.673378
C 3.448922 1.860404 -2.137699
H 3.513552 2.399289 -3.080502
C 2.228217 1.349163 -1.719654
H 1.336653 1.486730 -2.328115
C 0.265495 -1.521033 -1.038419
C -0.835939 -2.341324 -0.761738
H -1.493315 -2.113889 0.075860
C -1.093772 -3.448401 -1.559318
H -1.955491 -4.075987 -1.341874
C -0.258889 -3.750203 -2.631370
H -0.465578 -4.618340 -3.254368
C 0.840460 -2.944501 -2.902889
H 1.500397 -3.180961 -3.735099
C 1.103789 -1.832483 -2.110297
H 1.968960 -1.210955 -2.328078

Si -0.298153 2.890100 -0.145792
Cl -2.268018 3.711864 -0.125623

[Ph]YSiHMDS
E = -2472.17006049
P 1.667797 0.159234 0.021429
C 0.000005 -0.137615 -0.306101
C -0.387880 -1.488114 -0.750696
C 0.206460 -2.663268 -0.257815
H 1.004167 -2.596378 0.479019
C -0.228455 -3.919645 -0.659656
H 0.250696 -4.807471 -0.249881
C -1.277695 -4.048319 -1.564263
H -1.625810 -5.032623 -1.871006
C -1.864882 -2.897053 -2.082274
H -2.674052 -2.976435 -2.807475
C -1.419372 -1.641637 -1.691806
H -1.862285 -0.748465 -2.120264
C 2.293130 -0.633820 1.547443
C 3.429171 -1.441584 1.601268
H 4.023683 -1.614786 0.707661
C 3.793522 -2.048050 2.799797
H 4.675805 -2.684148 2.834398
C 3.032502 -1.848312 3.945625
H 3.319548 -2.326547 4.880121
C 1.895510 -1.045813 3.894436
H 1.290935 -0.894340 4.786353
C 1.520580 -0.449745 2.698148
H 0.616760 0.160345 2.643271
C 1.990862 1.934409 0.227996
C 2.587878 2.466812 1.367978
H 2.840669 1.823657 2.207283
C 2.850587 3.832490 1.438373
H 3.307261 4.246757 2.334891
C 2.528383 4.661446 0.372170
H 2.732657 5.728776 0.431703
C 1.946369 4.127314 -0.775879
H 1.694723 4.774513 -1.613462
C 1.679172 2.769620 -0.850728
H 1.214171 2.348025 -1.740515
C 2.813650 -0.316243 -1.310125
C 2.371972 -1.016359 -2.433109
H 1.332505 -1.322312 -2.509784
C 3.266242 -1.316630 -3.456112
H 2.917053 -1.864662 -4.328761
C 4.594502 -0.917445 -3.367544
H 5.289332 -1.154679 -4.170992
C 5.034497 -0.202486 -2.256572
H 6.069686 0.126558 -2.191609
C 4.146723 0.102512 -1.233976
H 4.485149 0.681731 -0.375031
Si -1.107207 1.184479 0.331862
N -2.760108 0.495562 0.113402
Si -3.753644 1.288309 -1.093021
Si -3.431023 -0.434669 1.442889
C -2.609145 2.224535 -2.263612
H -2.072220 3.031027 -1.746717
H -1.857318 1.561559 -2.713635
H -3.191284 2.674900 -3.078871
C -4.758021 0.083667 -2.135249

H -5.656348 -0.267153 -1.616154
H -5.084015 0.588592 -3.054939
H -4.171966 -0.797469 -2.422257
C -4.932842 2.521297 -0.302274
H -5.532795 3.051597 -1.053608
H -5.626170 2.021690 0.386991
H -4.369223 3.265143 0.275706
C -5.131122 -1.116192 1.026219
H -5.508720 -1.672804 1.894383
H -5.862218 -0.332312 0.793208
H -5.083778 -1.808415 0.176862
C -3.587128 0.684120 2.950247
H -3.988495 0.149414 3.821291
H -2.607638 1.097938 3.226460
H -4.248185 1.533652 2.734006
C -2.337136 -1.888006 1.896107
H -1.297356 -1.586049 2.078046
H -2.716753 -2.363741 2.810664
H -2.326376 -2.637511 1.095070

$^{Ph}YSiC_6F_5$
E = -2326.70822592
P -1.787069 -0.072308 -0.078053
C -0.105242 -0.113258 -0.474474
C 0.717579 0.926254 0.199115
C 1.326196 0.687742 1.439016
H 1.164365 -0.271303 1.928841
C 2.163286 1.633610 2.019914
H 2.639756 1.417843 2.974789
C 2.402366 2.844867 1.378333
H 3.064352 3.582269 1.828179
C 1.794805 3.102266 0.153115
H 1.980613 4.044160 -0.360100
C 0.960458 2.154950 -0.428139
H 0.501582 2.346663 -1.396159
C -2.129046 -0.747159 1.579996
C -3.224876 -0.313303 2.331220
H -3.870616 0.475278 1.949145
C -3.487883 -0.886483 3.569479
H -4.339989 -0.544790 4.153668
C -2.660654 -1.891656 4.061785
H -2.865555 -2.335838 5.034028
C -1.569549 -2.325398 3.315852
H -0.919142 -3.108062 3.700741
C -1.300984 -1.755218 2.076628
H -0.441533 -2.082989 1.491476
C -2.777332 -1.035117 -1.245419
C -3.749030 -1.935231 -0.807696
H -3.905438 -2.095828 0.256520
C -4.510686 -2.637710 -1.735234
H -5.261807 -3.345112 -1.390218
C -4.308388 -2.441476 -3.096313
H -4.903901 -2.995310 -3.819524
C -3.340763 -1.542656 -3.536327
H -3.175119 -1.394352 -4.601216
C -2.573074 -0.842084 -2.616425
H -1.802418 -0.151859 -2.954086
C -2.473479 1.609783 -0.026001
C -2.021252 2.480416 0.972850
H -1.313462 2.130559 1.722811

C -2.471865 3.792864 1.005566
H -2.111843 4.465805 1.780877
C -3.375816 4.245378 0.048488
H -3.727176 5.275036 0.076300
C -3.832384 3.381848 -0.940412
H -4.544272 3.730889 -1.685601
C -3.383582 2.065811 -0.980575
H -3.748768 1.394033 -1.754068
C 2.389482 -1.106222 -0.997386
C 4.279367 -1.368076 0.513390
C 4.364074 0.294399 -1.228128
C 4.958653 -0.353932 -0.151365
Si 0.532824 -1.521500 -1.416513
C 3.096298 -0.100079 -1.632661
C 3.009344 -1.718214 0.077052
F 2.336710 -2.636845 0.787381
F 2.527686 0.567393 -2.640660
F 5.009250 1.274736 -1.842558
F 6.169719 0.001560 0.247005
F 4.837827 -1.961617 1.559289

^{Ph}*YSiPy*
E = -1845.84660072
P -1.085754 -0.116716 -0.005344
C 0.596140 -0.451050 0.178718
C 1.491246 0.725072 0.021934
C 1.643993 1.680976 1.036274
C 2.542109 2.734577 0.901996
C 3.313438 2.855775 -0.249145
C 3.169387 1.918706 -1.268443
C 2.263907 0.874092 -1.138162
C -1.839173 0.731852 1.425258
C -2.879199 1.653220 1.287729
H -3.238559 1.926326 0.297449
C -3.454127 2.223435 2.418448
H -4.261119 2.945262 2.308862
C -2.998470 1.873001 3.685400
H -3.449408 2.322384 4.568198
C -1.964715 0.951427 3.825191
H -1.607170 0.676944 4.815616
C -1.383760 0.383693 2.698054
H -0.565940 -0.331686 2.789130
C -2.059032 -1.624814 -0.241897
C -3.221992 -1.860020 0.491229
H -3.547133 -1.146207 1.244281
C -3.963623 -3.015121 0.266957
H -4.865463 -3.197678 0.847551
C -3.551507 -3.933107 -0.691482
H -4.132031 -4.837512 -0.863618
C -2.394128 -3.699897 -1.428885
H -2.065052 -4.420355 -2.174622
C -1.645171 -2.552761 -1.204834
H -0.731546 -2.373710 -1.767807
C -1.442318 0.981719 -1.412920
C -0.942738 2.289897 -1.385312
H -0.395316 2.648052 -0.515820
C -1.144320 3.134792 -2.468390
H -0.747248 4.147370 -2.440012
C -1.846989 2.688113 -3.583532
H -2.002982 3.352599 -4.431270

C -2.352438 1.393680 -3.611679
H -2.908650 1.042156 -4.478379
C -2.151940 0.540745 -2.531297
H -2.554972 -0.468781 -2.560954
C 3.039830 -1.698525 0.571324
C 5.124816 -0.669798 1.191303
C 4.833934 -1.850973 -0.861680
C 5.654271 -1.111531 -0.017355
Si 1.161945 -2.122995 0.622986
N 3.569024 -2.144075 -0.578179
H 5.733333 -0.088240 1.882909
H 6.682457 -0.893838 -0.299026
H 5.220116 -2.223602 -1.813748
H 1.060203 1.579452 1.949966
H 2.648489 3.458384 1.708795
H 4.024921 3.673306 -0.350021
H 3.770711 2.000451 -2.172464
H 2.163765 0.130139 -1.925800
C 3.804720 -0.971191 1.490406
H 3.351616 -0.615934 2.415139

^{Ph}YSiPyr
E = -1861.90830830
P -1.071905 -0.123572 -0.047297
C 0.588782 -0.451548 -0.379565
C 1.479949 0.730439 -0.237140
C 2.031285 1.089972 1.000653
C 2.865413 2.196248 1.120554
C 3.168697 2.969893 0.004620
C 2.636795 2.621117 -1.233188
C 1.803072 1.516164 -1.351330
C -1.432097 0.032553 1.735937
C -2.457073 0.851381 2.216975
H -3.044492 1.453208 1.525919
C -2.723642 0.900782 3.580395
H -3.519756 1.542496 3.952859
C -1.971879 0.134465 4.466183
H -2.179973 0.178326 5.533648
C -0.952237 -0.683121 3.989431
H -0.361373 -1.280920 4.680413
C -0.679843 -0.733796 2.627365
H 0.124258 -1.362214 2.243418
C -2.165044 -1.423153 -0.672430
C -3.202707 -1.939916 0.103349
H -3.349362 -1.590025 1.122604
C -4.044072 -2.913942 -0.423637
H -4.847027 -3.320403 0.187793
C -3.855594 -3.370132 -1.722970
H -4.513520 -4.134970 -2.131276
C -2.822580 -2.854734 -2.500721
H -2.667918 -3.216579 -3.514967
C -1.975818 -1.886477 -1.979120
H -1.154823 -1.492239 -2.574435
C -1.644406 1.454409 -0.749654
C -1.107700 2.643913 -0.242055
H -0.404250 2.615447 0.588729
C -1.467055 3.862723 -0.800989
H -1.040083 4.781837 -0.405242
C -2.364047 3.906950 -1.864353
H -2.644036 4.863864 -2.300810

C -2.904316 2.728898 -2.366945
H -3.610762 2.760095 -3.194028
C -2.546421 1.503933 -1.813402
H -2.975705 0.586431 -2.209612
C 3.003812 -1.766487 -0.425695
C 4.773472 -1.564374 0.998372
C 4.974696 -1.022404 -1.287344
C 5.573881 -1.094183 -0.035256
Si 1.126644 -2.171836 -0.585903
N 3.707742 -1.358308 -1.489041
N 3.504784 -1.902025 0.811206
H 5.171291 -1.668700 2.011327
H 6.610638 -0.807766 0.123812
H 5.539998 -0.678610 -2.157604
H 1.813038 0.473736 1.870708
H 3.285530 2.451557 2.092244
H 3.822657 3.835151 0.097621
H 2.874647 3.213715 -2.115204
H 1.388945 1.240741 -2.319202

^{Ph}YSiB2
E = -2004.85620145
P -1.628311 -0.113231 -0.043984
C 0.012776 -0.560385 -0.387767
C 0.988751 0.545799 -0.173925
C 1.617118 0.742295 1.063867
C 2.574117 1.738052 1.234452
C 2.917456 2.568656 0.173843
C 2.291876 2.397349 -1.058080
C 1.338238 1.402596 -1.227417
C -2.001328 -0.082989 1.742583
C -2.958133 0.781231 2.281727
H -3.476622 1.490610 1.639450
C -3.244912 0.737855 3.641086
H -3.987506 1.414886 4.058727
C -2.581800 -0.166341 4.465343
H -2.805266 -0.195372 5.530161
C -1.631316 -1.030022 3.930476
H -1.110846 -1.736969 4.573324
C -1.339273 -0.988510 2.572194
H -0.592710 -1.655650 2.141457
C -2.828982 -1.238778 -0.794348
C -3.930914 -1.709109 -0.080570
H -4.063340 -1.435758 0.963680
C -4.857013 -2.540107 -0.702373
H -5.710764 -2.911579 -0.139463
C -4.688825 -2.898332 -2.034579
H -5.413532 -3.550981 -2.517683
C -3.591072 -2.428711 -2.750440
H -3.452870 -2.715078 -3.790848
C -2.659525 -1.605021 -2.134033
H -1.788656 -1.250284 -2.681230
C -2.038112 1.568473 -0.604140
C -1.418405 2.651131 0.031657
H -0.749542 2.479739 0.873694
C -1.649484 3.944940 -0.414535
H -1.158690 4.779948 0.080944
C -2.499451 4.170535 -1.493475
H -2.678747 5.185888 -1.841962
C -3.121344 3.099143 -2.123993

H -3.791444 3.272383 -2.963634
C -2.892749 1.799722 -1.682836
H -3.386279 0.966574 -2.178121
H 1.368445 0.079788 1.891029
H 3.063289 1.855046 2.199899
H 3.675619 3.338507 0.303171
H 2.555997 3.039108 -1.897058
H 0.858149 1.261558 -2.194015
Si 0.400383 -2.301393 -0.742074
C 4.321736 -0.863343 -0.943409
C 4.300603 -1.326514 0.369657
C 5.379780 -0.128687 -1.440389
C 5.336227 -1.076224 1.247721
C 6.430239 0.135887 -0.558235
H 5.378693 0.232610 -2.465218
C 6.409526 -0.328231 0.756596
H 5.304779 -1.439882 2.271432
H 7.281609 0.718122 -0.904883
H 7.245356 -0.101948 1.415538
B 2.389493 -1.865805 -0.592791
O 3.130165 -1.984478 0.586110
O 3.168821 -1.234074 -1.560558

^{Ph}YSiB1
E = -2274.04404077
P 1.702728 -0.481255 -0.072151
C -0.000383 -0.260501 -0.282675
C -0.769481 -0.344807 0.989011
C -0.986082 0.784920 1.790981
H -0.575987 1.741202 1.472901
C -1.724805 0.702650 2.965013
H -1.886071 1.597903 3.564177
C -2.259068 -0.516588 3.372338
H -2.840556 -0.582467 4.290251
C -2.045221 -1.648967 2.593258
H -2.463736 -2.607751 2.895451
C -1.306829 -1.564822 1.418571
H -1.154378 -2.449831 0.802225
C 2.527626 0.992336 0.622312
C 3.692442 0.884902 1.387766
H 4.108918 -0.095692 1.611464
C 4.316074 2.029633 1.869136
H 5.220962 1.941866 2.467383
C 3.780145 3.283751 1.590690
H 4.266531 4.178933 1.974053
C 2.622229 3.394163 0.828172
H 2.194362 4.369832 0.610198
C 1.994668 2.251987 0.343326
H 1.083058 2.333371 -0.249116
C 2.549666 -0.867901 -1.622963
C 3.743340 -0.241766 -1.978768
H 4.166375 0.530304 -1.340185
C 4.387871 -0.596706 -3.159501
H 5.315000 -0.099852 -3.437773
C 3.847045 -1.578105 -3.981254
H 4.352597 -1.852916 -4.905130
C 2.655594 -2.206103 -3.627701
H 2.226015 -2.967965 -4.274810
C 2.002985 -1.850525 -2.455806
H 1.060355 -2.320647 -2.182308

C 2.116803 -1.803240 1.109832
C 1.796935 -1.613714 2.459726
H 1.365687 -0.670098 2.790904
C 2.021454 -2.630505 3.377637
H 1.763025 -2.477000 4.423288
C 2.568048 -3.840678 2.960524
H 2.742691 -4.636665 3.681991
C 2.893314 -4.031019 1.622562
H 3.327068 -4.973261 1.293621
C 2.668563 -3.016823 0.697346
H 2.929466 -3.171919 -0.347028
Si -0.611005 0.297851 -1.900074
N -3.552558 -0.026536 -0.724922
C -3.984110 2.009470 0.101121
C -4.439090 0.742483 0.024623
H -4.460830 2.870261 0.555823
H -5.375041 0.346256 0.399397
B -2.437668 0.806695 -1.124599
N -2.787536 2.112216 -0.604091
C -1.964536 3.243300 -0.575082
C -1.885384 4.053198 0.562771
C -1.178444 3.563366 -1.687846
C -1.035162 5.152175 0.584933
H -2.474455 3.796917 1.440857
C -0.319884 4.654011 -1.652390
H -1.258104 2.954518 -2.586192
C -0.245260 5.458761 -0.518763
H -0.983815 5.768992 1.480504
H 0.284637 4.884175 -2.527725
H 0.417631 6.321582 -0.498526
C -3.657732 -1.414594 -0.867249
C -3.027967 -2.053594 -1.940505
C -4.348941 -2.194187 0.065202
C -3.062311 -3.436110 -2.057664
H -2.521843 -1.454509 -2.695839
C -4.393929 -3.575968 -0.068832
H -4.809090 -1.719537 0.927834
C -3.745373 -4.209161 -1.123651
H -2.560928 -3.911291 -2.899087
H -4.929855 -4.165023 0.673601
H -3.776384 -5.292342 -1.220013

$^{F}Y_2Si$
E = -3899.58524078
P -2.956785 -0.891816 -0.132779
P 2.837423 -1.241497 -0.021376
C -1.520108 -0.092021 -0.649391
C -1.454189 1.359938 -0.729690
C -2.186622 2.253645 0.063965
C -2.054149 3.632428 -0.002097
C -1.125871 4.189283 -0.870711
C -0.369491 3.345178 -1.673954
C -0.558690 1.974301 -1.620363
C -3.117354 -1.131721 1.676716
C -3.989768 -0.407327 2.488228
H -4.682370 0.304806 2.047654
C -3.957464 -0.573072 3.869440
H -4.637221 0.002211 4.494763
C -3.056470 -1.457902 4.448763
H -3.032307 -1.582063 5.529714

C -2.178965 -2.178781 3.643156
H -1.469607 -2.871434 4.092125
C -2.203952 -2.012514 2.265922
H -1.506467 -2.567145 1.633285
C -3.043330 -2.575041 -0.824597
C -3.676034 -3.607067 -0.132199
H -4.024573 -3.451721 0.886447
C -3.854860 -4.846099 -0.740207
H -4.340101 -5.650419 -0.190852
C -3.414493 -5.054411 -2.040924
H -3.552509 -6.024809 -2.514104
C -2.798926 -4.018899 -2.740554
H -2.454259 -4.177846 -3.760305
C -2.616199 -2.783217 -2.138726
H -2.126185 -1.970764 -2.671854
C -4.503977 -0.145605 -0.729055
C -4.465105 0.760921 -1.788978
H -3.508855 1.055697 -2.213531
C -5.648716 1.272908 -2.309322
H -5.611460 1.985906 -3.130383
C -6.873107 0.872005 -1.787522
H -7.797788 1.274990 -2.196096
C -6.916622 -0.055725 -0.750985
H -7.873740 -0.386390 -0.352688
C -5.737294 -0.567410 -0.225747
H -5.776611 -1.305854 0.573459
C 1.391004 -0.302148 0.014366
C 1.521270 1.115450 0.342534
C 0.709986 1.703819 1.320503
C 0.726009 3.066289 1.572972
C 1.608118 3.893495 0.887898
C 2.454876 3.345077 -0.065805
C 2.381150 1.986361 -0.334067
C 4.131877 -0.461628 0.998953
C 4.013968 -0.510998 2.390485
H 3.208687 -1.078613 2.850385
C 4.923382 0.162939 3.194598
H 4.823354 0.116868 4.277045
C 5.955381 0.896040 2.617252
H 6.665807 1.426945 3.247960
C 6.073955 0.953664 1.233678
H 6.873650 1.532904 0.776704
C 5.167348 0.278566 0.424039
H 5.252832 0.347904 -0.656883
C 2.515064 -2.889454 0.697415
C 3.067097 -4.062349 0.185792
H 3.680016 -4.036222 -0.710641
C 2.808153 -5.283517 0.801546
H 3.233081 -6.194918 0.385817
C 2.003936 -5.340993 1.932167
H 1.802224 -6.297546 2.410424
C 1.445786 -4.172632 2.444657
H 0.805034 -4.213778 3.323398
C 1.690308 -2.954805 1.827405
H 1.215231 -2.045061 2.192978
C 3.613986 -1.523857 -1.644240
C 4.883817 -2.098472 -1.763768
H 5.446286 -2.371837 -0.871404
C 5.436722 -2.307021 -3.020629
H 6.424919 -2.753738 -3.111230

C 4.728295 -1.937184 -4.161264
H 5.163907 -2.097992 -5.145697
C 3.473853 -1.349748 -4.043695
H 2.928234 -1.042824 -4.933544
C 2.916056 -1.138564 -2.787335
H 1.950776 -0.645198 -2.678647
Si -0.084774 -1.274551 -0.503863
F 3.154036 1.505453 -1.310352
F 3.288020 4.122489 -0.739461
F 1.612192 5.196170 1.120624
F -0.122838 3.593737 2.443945
F -0.155176 0.959550 2.001802
F 0.204204 1.224671 -2.414874
F 0.560038 3.854858 -2.469693
F -0.932320 5.498277 -0.907391
F -2.777890 4.412434 0.784829
F -3.054770 1.768871 0.955277

^{F}YSiCl
E = -2555.28314520
P -1.194303 0.048498 0.079074
C 0.036260 -0.662930 -0.902035
C 1.423857 -0.348313 -0.526071
C 2.233777 -1.265116 0.147349
C 3.552104 -0.983822 0.477212
C 4.101125 0.242533 0.122950
C 3.327159 1.176321 -0.554536
C 2.013822 0.867797 -0.878391
C -1.593244 -0.887091 1.588442
C -2.229432 -0.268373 2.670093
H -2.461113 0.795173 2.631708
C -2.557935 -1.011302 3.796131
H -3.052233 -0.530015 4.637645
C -2.247900 -2.368414 3.849756
H -2.500252 -2.947159 4.736267
C -1.609225 -2.982222 2.778558
H -1.357654 -4.039672 2.824136
C -1.280484 -2.244121 1.646133
H -0.763132 -2.709375 0.809372
C -2.728612 0.221244 -0.863421
C -3.965530 -0.163951 -0.346685
H -4.030398 -0.610764 0.642685
C -5.116364 0.008976 -1.108244
H -6.078765 -0.302850 -0.708061
C -5.036343 0.569518 -2.377761
H -5.939047 0.700023 -2.971422
C -3.803187 0.956677 -2.894991
H -3.738276 1.387950 -3.891583
C -2.649062 0.779476 -2.144837
H -1.677323 1.060352 -2.547745
C -0.715248 1.684338 0.703766
C 0.269200 1.740769 1.696152
H 0.660811 0.820505 2.128241
C 0.750962 2.969158 2.127849
H 1.522349 3.008064 2.894073
C 0.247238 4.145458 1.580420
H 0.626330 5.107743 1.918626
C -0.740996 4.092156 0.604025
H -1.139527 5.011172 0.179491
C -1.220823 2.864947 0.160964

H -1.988843 2.829733 -0.608148
Si -0.472040 -1.843082 -2.187969
Cl 1.458847 -2.486136 -2.826401
F 1.303129 1.778530 -1.533273
F 3.849100 2.346497 -0.889429
F 5.355470 0.522336 0.434798
F 4.290949 -1.872621 1.122843
F 1.742804 -2.446204 0.498617

^{F}YSiHMDS
E = -2969.07661631
P -1.869213 -0.305989 -0.137902
C -0.192410 -0.075901 -0.464557
C 0.455203 1.221912 -0.323319
C 0.088468 2.218367 0.586494
C 0.755827 3.426862 0.709661
C 1.840909 3.698013 -0.113463
C 2.223249 2.751767 -1.056732
C 1.525333 1.559435 -1.165909
C -2.245571 -0.714057 1.603756
C -2.828188 0.175151 2.505573
H -3.174830 1.148906 2.169307
C -2.933578 -0.169181 3.849979
H -3.381004 0.533382 4.550072
C -2.457853 -1.394600 4.299891
H -2.536480 -1.655898 5.353376
C -1.869187 -2.283466 3.403365
H -1.485575 -3.240692 3.750608
C -1.758472 -1.942015 2.063546
H -1.273455 -2.626474 1.363228
C -2.507022 -1.715185 -1.096611
C -3.517868 -2.535011 -0.593896
H -3.868439 -2.411081 0.428439
C -4.074837 -3.524117 -1.398219
H -4.855652 -4.167424 -0.997736
C -3.633055 -3.691192 -2.704944
H -4.067066 -4.468538 -3.330878
C -2.636494 -2.862786 -3.214001
H -2.288950 -2.990135 -4.237042
C -2.076754 -1.875388 -2.416482
H -1.291650 -1.229602 -2.804437
C -2.945814 1.066807 -0.637239
C -2.465420 2.032321 -1.522678
H -1.435471 1.987135 -1.867334
C -3.309532 3.041046 -1.973954
H -2.928340 3.795427 -2.659009
C -4.634809 3.082109 -1.556664
H -5.292726 3.873383 -1.910597
C -5.124711 2.103588 -0.696408
H -6.166359 2.122957 -0.382596
C -4.285745 1.095272 -0.241051
H -4.676299 0.321998 0.418808
Si 0.747739 -1.668529 -0.601980
N 2.381560 -1.216259 -0.028702
Si 3.718134 -1.661555 -1.098373
Si 2.643380 -1.078585 1.712080
C 3.039832 -2.161431 -2.780657
H 2.421894 -3.065903 -2.719775
H 2.440773 -1.367537 -3.242337
H 3.884168 -2.374921 -3.450898

C 4.902479 -0.226183 -1.340537
H 5.469105 0.010065 -0.433753
H 5.626535 -0.479167 -2.127120
H 4.367291 0.676636 -1.655222
C 4.662261 -3.153767 -0.443597
H 5.477616 -3.420370 -1.129238
H 5.103419 -2.986058 0.545968
H 3.990530 -4.018909 -0.368094
C 4.377516 -0.468189 2.102038
H 4.492502 -0.426593 3.193449
H 5.176895 -1.105459 1.708580
H 4.527175 0.546594 1.712522
C 2.375539 -2.769252 2.498268
H 2.560265 -2.745417 3.580383
H 1.337287 -3.094762 2.342301
H 3.028127 -3.530892 2.054640
C 1.484819 0.109855 2.587534
H 0.427763 -0.033911 2.340422
H 1.598057 -0.054106 3.668127
H 1.751769 1.152799 2.383661
F 1.884065 0.731997 -2.143417
F -0.934489 1.992375 1.415207
F 0.370139 4.316923 1.611619
F 2.496373 4.842548 -0.006452
F 3.236596 3.007809 -1.872256

$^{F}YSiC_6F_5$
E = -2823.61374882
P -2.104130 -0.194936 -0.034501
C -0.426104 -0.451107 -0.386008
C 0.506672 0.557616 0.146591
C 1.288695 0.334535 1.280909
C 2.252231 1.239666 1.704724
C 2.436412 2.422925 0.998375
C 1.661670 2.687219 -0.124970
C 0.712625 1.760913 -0.531533
C -2.668458 -1.041841 1.471778
C -3.858689 -0.660663 2.101702
H -4.442400 0.170818 1.708104
C -4.288299 -1.337924 3.234952
H -5.213930 -1.042058 3.724565
C -3.530436 -2.388986 3.746926
H -3.866784 -2.914882 4.638425
C -2.341795 -2.759229 3.129257
H -1.742843 -3.571192 3.535879
C -1.906469 -2.086848 1.992059
H -0.965132 -2.356763 1.516935
C -3.138934 -0.752204 -1.407901
C -4.293927 -1.509117 -1.214233
H -4.587239 -1.815717 -0.212935
C -5.060931 -1.889446 -2.310216
H -5.955471 -2.489959 -2.159435
C -4.680891 -1.512499 -3.593170
H -5.282126 -1.815229 -4.448276
C -3.527740 -0.757081 -3.788213
H -3.224593 -0.469541 -4.792649
C -2.751461 -0.380779 -2.701019
H -1.835701 0.191426 -2.843634
C -2.451350 1.557243 0.279219
C -2.007062 2.102476 1.488724

H -1.551660 1.458867 2.240548
C -2.143347 3.462881 1.728493
H -1.791623 3.884171 2.667855
C -2.726968 4.283223 0.767053
H -2.831827 5.349913 0.954903
C -3.176730 3.741956 -0.431874
H -3.635376 4.382282 -1.182274
C -3.037392 2.381118 -0.680796
H -3.383215 1.963958 -1.623707
C 1.950373 -1.669126 -1.056508
C 4.001625 -2.137421 0.164212
C 3.984595 -0.418767 -1.527858
C 4.663582 -1.151378 -0.558754
Si 0.039801 -1.950778 -1.288755
C 2.644500 -0.696671 -1.758124
C 2.659936 -2.379336 -0.103270
F 2.032374 -3.302346 0.629804
F 1.997937 0.044025 -2.664000
F 4.619340 0.526594 -2.203511
F 5.941639 -0.908421 -0.325111
F 4.652058 -2.821661 1.091847
F -0.002178 2.030876 -1.616659
F 1.835844 3.815606 -0.792780
F 3.342383 3.297568 1.398334
F 2.984545 0.992641 2.778733
F 1.133731 -0.785909 1.978078

^{F}YSiPy
E = -2342.75509713
P -1.610152 -0.095516 0.018299
C -0.027461 -0.740261 -0.228301
C 1.112733 0.152576 0.036360
C 1.955641 -0.035609 1.132374
C 3.146208 0.661365 1.279480
C 3.500051 1.615976 0.334340
C 2.671442 1.849115 -0.756417
C 1.500246 1.119757 -0.893211
C -2.291213 -0.386405 1.681558
C -3.382184 0.358476 2.142089
H -3.816519 1.134609 1.512818
C -3.903403 0.114054 3.405261
H -4.752505 0.692954 3.763126
C -3.333137 -0.865568 4.215328
H -3.739171 -1.051734 5.207845
C -2.240947 -1.597040 3.763592
H -1.788067 -2.353341 4.401307
C -1.716681 -1.359361 2.496724
H -0.848851 -1.909778 2.137283
C -2.781037 -0.780442 -1.177485
C -4.033906 -1.269153 -0.810558
H -4.334045 -1.278829 0.234613
C -4.893780 -1.764936 -1.785230
H -5.866370 -2.157769 -1.496298
C -4.508368 -1.766992 -3.120592
H -5.182986 -2.158403 -3.879642
C -3.257354 -1.278429 -3.488742
H -2.951100 -1.288256 -4.532594
C -2.389429 -0.792052 -2.521489
H -1.399353 -0.428412 -2.793719
C -1.641970 1.714229 -0.131467

C -1.013863 2.458307 0.872797
H -0.590780 1.952929 1.740169
C -0.928314 3.839601 0.764190
H -0.433323 4.413544 1.544761
C -1.474651 4.485587 -0.341111
H -1.406890 5.568381 -0.425833
C -2.108132 3.749122 -1.335470
H -2.538896 4.253214 -2.198042
C -2.190084 2.364651 -1.236039
H -2.680385 1.793179 -2.020619
C 2.085369 -2.457620 -0.735367
C 4.229171 -2.938553 0.249234
C 3.981112 -1.564395 -1.685392
C 4.811790 -2.139183 -0.726961
F 0.752270 1.332906 -1.969474
F 3.016423 2.753548 -1.661352
F 4.625043 2.299947 0.471076
F 3.936747 0.437281 2.318636
F 1.647127 -0.942861 2.054999
Si 0.155268 -2.459498 -0.793828
N 2.662320 -1.710927 -1.691586
H 4.841854 -3.414681 1.013667
H 5.885486 -1.966207 -0.752671
H 4.406576 -0.946424 -2.480169
C 2.851402 -3.114782 0.232690
H 2.363614 -3.732376 0.986132

^{F}YSiPyr
E = -2358.81572896
P -1.601648 -0.069733 0.009954
C -0.048862 -0.695501 -0.412465
C 1.114487 0.132413 -0.049968
C 1.937520 -0.160432 1.038378
C 3.124658 0.521718 1.269576
C 3.495647 1.557953 0.421590
C 2.683294 1.896518 -0.654191
C 1.511150 1.189091 -0.871326
C -2.259820 -0.693776 1.586796
C -3.331034 -0.050891 2.216992
H -3.767160 0.843907 1.773693
C -3.829971 -0.549145 3.412658
H -4.664120 -0.050488 3.902269
C -3.256385 -1.681581 3.987327
H -3.645869 -2.067973 4.927342
C -2.182000 -2.310846 3.369936
H -1.725417 -3.187004 3.825438
C -1.679241 -1.818675 2.169310
H -0.823938 -2.289505 1.687174
C -2.822512 -0.427449 -1.276789
C -4.100512 -0.907038 -0.992999
H -4.393495 -1.114423 0.033329
C -4.996204 -1.141842 -2.030888
H -5.988899 -1.527767 -1.808760
C -4.621407 -0.894466 -3.346356
H -5.324309 -1.082435 -4.155710
C -3.344971 -0.417271 -3.631879
H -3.046797 -0.233286 -4.661847
C -2.441646 -0.190164 -2.602913
H -1.431963 0.159834 -2.814565
C -1.531270 1.727793 0.254365

C -0.914752 2.198184 1.418892
H -0.582198 1.492840 2.179717
C -0.724344 3.560703 1.602898
H -0.239582 3.921581 2.507599
C -1.151736 4.459881 0.629974
H -1.001528 5.528023 0.774020
C -1.770522 3.995229 -0.524988
H -2.106638 4.697548 -1.284847
C -1.957832 2.630890 -0.718095
H -2.434020 2.272066 -1.627550
C 1.987404 -2.371211 -1.155950
C 3.771154 -3.140702 0.036082
C 4.050099 -1.586068 -1.717400
C 4.631084 -2.332790 -0.697486
F 0.763284 1.522458 -1.917664
F 3.039553 2.885511 -1.460199
F 4.620004 2.221964 0.638656
F 3.897034 0.203742 2.298108
F 1.603031 -1.134140 1.876657
Si 0.062348 -2.298856 -1.262977
N 2.744217 -1.597833 -1.947245
N 2.464786 -3.173787 -0.196648
H 4.149202 -3.781158 0.836666
H 5.699052 -2.298426 -0.497020
H 4.661507 -0.950991 -2.363827

^{F}YSiB2
E = -2501.76361265
P 2.012065 -0.023599 0.096022
C 0.473836 0.044399 0.902559
C -0.686975 0.040914 -0.015404
C -1.358671 -1.126860 -0.376027
C -2.508145 -1.109168 -1.155823
C -2.989867 0.099834 -1.636677
C -2.317617 1.279529 -1.337196
C -1.177164 1.234969 -0.551262
C 2.733768 -1.686330 -0.039594
C 3.760308 -1.941577 -0.955820
H 4.124654 -1.144209 -1.602887
C 4.303745 -3.215598 -1.048494
H 5.101548 -3.414334 -1.761316
C 3.820673 -4.238489 -0.235092
H 4.244109 -5.238081 -0.313054
C 2.792732 -3.988291 0.666206
H 2.407158 -4.789906 1.292545
C 2.244898 -2.713319 0.764977
H 1.424998 -2.509146 1.451153
C 3.229045 1.046373 0.900634
C 4.549620 0.654410 1.116933
H 4.880449 -0.341388 0.832015
C 5.441225 1.534484 1.720943
H 6.468025 1.222380 1.899849
C 5.019971 2.802769 2.103576
H 5.720243 3.487077 2.578729
C 3.701308 3.195049 1.890653
H 3.367335 4.183310 2.199488
C 2.802554 2.318842 1.298476
H 1.761934 2.604293 1.150177
C 1.807237 0.514986 -1.625291
C 1.248968 -0.393326 -2.531861

H 1.082491 -1.427202 -2.231408
C 0.893097 0.026682 -3.806021
H 0.451542 -0.680898 -4.504630
C 1.096203 1.351212 -4.183681
H 0.813533 1.680993 -5.181433
C 1.659222 2.253102 -3.287986
H 1.820142 3.287760 -3.583099
C 2.010990 1.840090 -2.007586
H 2.436177 2.553613 -1.305755
Si 0.435023 0.094556 2.722989
C -3.568233 0.661969 1.810847
C -3.553711 -0.725160 1.928042
C -4.620013 1.334252 1.219423
C -4.587628 -1.509231 1.455720
C -5.666451 0.550129 0.728150
H -4.608086 2.415952 1.115028
C -5.649315 -0.840356 0.842090
H -4.550604 -2.592553 1.529939
H -6.506806 1.034131 0.235462
H -6.477237 -1.417990 0.436986
B -1.601001 0.049875 2.594960
O -2.364595 -1.110094 2.468030
O -2.389830 1.156771 2.279487
F -0.555767 2.377723 -0.278986
F -0.916646 -2.301157 0.058590
F -3.153582 -2.233072 -1.428211
F -2.787716 2.433973 -1.785716
F -4.100307 0.135405 -2.353163

^{F}YSiB1
E = -2770.95300182
P 2.035793 0.396953 0.046825
C 0.391122 0.122205 -0.405357
C -0.498131 -0.431862 0.627664
C -1.413257 0.364311 1.318981
C -2.378375 -0.170354 2.158069
C -2.397528 -1.540850 2.388853
C -1.458470 -2.356243 1.770767
C -0.522468 -1.797718 0.912699
C 2.411592 2.132768 0.444840
C 3.607713 2.472168 1.086925
H 4.324840 1.695342 1.351317
C 3.873058 3.798065 1.401537
H 4.804189 4.061475 1.899382
C 2.942262 4.786885 1.089322
H 3.149509 5.824905 1.342921
C 1.745366 4.448543 0.468972
H 1.006748 5.213984 0.239326
C 1.475691 3.122208 0.146691
H 0.528714 2.848122 -0.315872
C 3.169372 -0.161511 -1.249316
C 4.294090 0.557300 -1.649829
H 4.514136 1.525437 -1.206327
C 5.122981 0.044473 -2.642603
H 5.993260 0.612764 -2.964354
C 4.835205 -1.182771 -3.228621
H 5.485368 -1.578598 -4.006521
C 3.710782 -1.900831 -2.829649
H 3.479462 -2.857671 -3.293667
C 2.871638 -1.391214 -1.848995

H 1.972011 -1.928879 -1.550246
C 2.434004 -0.501262 1.572750
C 1.919659 0.000749 2.773203
H 1.392734 0.954528 2.780229
C 2.074515 -0.718707 3.950068
H 1.668610 -0.327260 4.880554
C 2.744970 -1.938760 3.936009
H 2.865347 -2.502952 4.858780
C 3.260134 -2.437480 2.745053
H 3.784566 -3.390632 2.733558
C 3.102671 -1.724352 1.561475
H 3.497067 -2.123789 0.629994
Si -0.086325 0.616039 -2.090225
N -2.685631 -1.102889 -1.674087
C -4.237850 0.373083 -1.026658
C -3.984167 -0.941370 -1.198387
H -5.167590 0.844646 -0.730694
H -4.666239 -1.774910 -1.079774
B -2.065081 0.201454 -1.782190
N -3.113689 1.113127 -1.384982
C -3.002749 2.498682 -1.188863
C -3.670604 3.134267 -0.139081
C -2.179957 3.255585 -2.027508
C -3.509304 4.497709 0.067905
H -4.269618 2.544102 0.550720
C -2.007784 4.615439 -1.800985
H -1.685595 2.773130 -2.868707
C -2.673257 5.246496 -0.754589
H -4.028641 4.975524 0.896667
H -1.360038 5.187475 -2.463094
H -2.545965 6.313550 -0.584145
C -2.044277 -2.342169 -1.800570
C -0.950466 -2.472449 -2.662666
C -2.433557 -3.448171 -1.038891
C -0.236639 -3.661253 -2.721399
H -0.674325 -1.639767 -3.308712
C -1.716287 -4.635483 -1.106136
H -3.268133 -3.362192 -0.347353
C -0.608152 -4.748461 -1.937974
H 0.612697 -3.737532 -3.398599
H -2.018293 -5.472972 -0.480612
H -0.044108 -5.677603 -1.979905
F -1.380696 1.682608 1.161474
F -3.267515 0.614716 2.751723
F -3.316272 -2.069924 3.182513
F -1.495964 -3.666725 1.967709
F 0.333813 -2.612228 0.305259

l
E = -1307.89084248
C -1.631093 2.185146 0.105973
C 0.493361 1.018306 0.103009
C 1.158918 2.239807 0.233555
C 0.430365 3.421237 0.285614
C -0.962027 3.397023 0.214501
H -2.720649 2.187350 0.066654
H 2.243336 2.263135 0.303823
H 0.954369 4.369709 0.387260
H -1.527484 4.326220 0.252194
N 1.076829 -0.241792 0.060797

Si -0.073639 -1.621699 -0.029334
C 2.476466 -0.415537 0.008615
C 3.249039 0.203913 -0.979761
C 3.105121 -1.262929 0.923559
C 4.618847 -0.013468 -1.039471
H 2.758771 0.848756 -1.706178
C 4.474577 -1.490028 0.849595
H 2.502120 -1.738778 1.694180
C 5.239183 -0.862621 -0.126931
H 5.205880 0.475462 -1.815131
H 4.947372 -2.156317 1.568988
H 6.312369 -1.034534 -0.179543
C -0.926758 0.973185 0.055542
C -1.421022 -0.385420 -0.019640
P -3.076553 -0.797234 -0.082923
C -4.045878 -0.269126 1.362358
H -3.947694 0.813732 1.489781
H -5.104372 -0.532075 1.254881
H -3.628292 -0.756194 2.249748
C -3.970989 -0.129434 -1.518322
H -5.031920 -0.403035 -1.492958
H -3.873891 0.960863 -1.533223
H -3.504557 -0.526193 -2.426091
C -3.285917 -2.589994 -0.175453
H -4.351412 -2.841100 -0.204803
H -2.788777 -2.967512 -1.074846
H -2.816879 -3.055017 0.697751

II
E = -1857.08091178
C 0.300288 3.093763 -0.240201
C 0.109878 1.736831 -0.005270
C -1.085220 1.077133 -0.332088
C -2.108112 1.863767 -0.888643
C -1.927237 3.219612 -1.108632
C -0.721663 3.846231 -0.796056
H 1.257970 3.531368 0.033709
H -3.048180 1.390500 -1.159395
H -2.740836 3.795991 -1.545527
H -0.586243 4.909914 -0.977262
N -1.234272 -0.306415 -0.184838
S 1.359599 0.851185 0.862051
O 2.625917 1.614048 0.764257
O 0.855598 0.571601 2.205828
C 1.493507 -0.603860 -0.052532
Si 0.003629 -1.595020 -0.370804
C -2.570715 -0.775801 -0.013056
C -3.276558 -0.412489 1.136385
C -3.174052 -1.605901 -0.956429
C -4.571712 -0.870322 1.332246
H -2.785939 0.224430 1.869930
C -4.467720 -2.074458 -0.747471
H -2.620764 -1.878957 -1.852649
C -5.171400 -1.705881 0.392661
H -5.113492 -0.583169 2.231539
H -4.929198 -2.724841 -1.488210
H -6.184741 -2.069125 0.551454
P 3.115184 -1.129596 -0.291066
C 4.046366 -0.086141 -1.436108
H 5.089847 -0.411383 -1.513179

H 3.564745 -0.125513 -2.418553
H 3.988117 0.934275 -1.043482
C 3.110219 -2.799493 -0.979451
H 2.567061 -2.804535 -1.930193
H 4.140978 -3.132222 -1.141921
H 2.604471 -3.480182 -0.286711
C 4.068564 -1.186952 1.244241
H 4.059643 -0.175684 1.665032
H 3.567452 -1.866349 1.941327
H 5.098137 -1.515759 1.064112

III
E = -2619.14154696
C -0.435060 1.646577 -0.544084
C 0.435033 1.646570 0.544309
C 0.865249 2.856458 1.085850
C 0.434439 4.059716 0.543305
C -0.433929 4.059749 -0.543255
C -0.865003 2.856539 -1.085693
H 1.546809 2.826123 1.932725
H 0.775956 4.999030 0.973823
H -0.775246 4.999092 -0.973875
H -1.546576 2.826365 -1.932568
S -0.996757 0.163857 -1.419169
S 0.996193 0.163689 1.419077
O -2.263314 0.602243 -2.043798
O 0.119382 -0.196082 -2.309179
O -0.120155 -0.196468 2.308702
O 2.262725 0.601437 2.044191
C -1.336032 -1.092412 -0.332423
C 1.335589 -1.092147 0.331740
Si 0.000304 -2.342628 -0.001447
P 2.881851 -1.019158 -0.416437
P -2.881640 -1.019373 0.416859
C 2.865964 -2.176439 -1.798980
H 3.839989 -2.194021 -2.299634
H 2.618972 -3.179234 -1.432145
H 2.082316 -1.842158 -2.489651
C 3.205369 0.615477 -1.114990
H 3.255613 1.336191 -0.291881
H 4.143584 0.622444 -1.680866
H 2.356373 0.853938 -1.766774
C 4.285408 -1.424152 0.650552
H 4.177095 -2.456633 0.999163
H 5.241030 -1.301481 0.128204
H 4.223362 -0.750089 1.511328
C -2.864670 -2.177470 1.798730
H -3.838309 -2.195907 2.300114
H -2.617373 -3.179869 1.431013
H -2.080806 -1.843217 2.489150
C -3.205080 0.614961 1.116156
H -3.255346 1.335817 0.293135
H -4.143416 0.621741 1.681801
H -2.356140 0.853453 1.767995
C -4.285929 -1.423567 -0.649503
H -5.241258 -1.300124 -0.126812
H -4.223651 -0.749525 -1.510284
H -4.178571 -2.456122 -0.998188

IV
E = -1827.11222845
C 0.880574 2.966537 0.296341
C 0.292593 1.715634 0.320202
C -0.872961 1.341589 -0.354410
C -1.469263 2.333490 -1.136655
C -0.914262 3.611152 -1.186072
C 0.243051 3.935819 -0.474125
H 1.785201 3.181788 0.862353
H -2.381101 2.112703 -1.690633
H -1.398537 4.381097 -1.784897
H 0.644228 4.946174 -0.518859
B -1.210469 -0.204426 -0.078125
S 0.902463 0.236492 1.058350
O 1.818496 0.426913 2.186424
O -0.479970 -0.389863 1.345218
C 1.487639 -0.741937 -0.228237
Si 0.138110 -1.286461 -1.341904
C -2.725303 -0.666445 0.008717
C -3.692544 0.189655 0.554704
C -3.162981 -1.920456 -0.433184
C -5.027377 -0.185619 0.657992
H -3.387215 1.174683 0.908566
C -4.495577 -2.308634 -0.335931
H -2.441665 -2.611984 -0.871431
C -5.433392 -1.439495 0.211126
H -5.755385 0.500984 1.088182
H -4.804259 -3.291230 -0.690101
H -6.478083 -1.737350 0.287524
P 3.183721 -1.030789 -0.249361
C 4.169305 0.487594 -0.184045
H 5.242476 0.268527 -0.208812
H 3.896695 1.126251 -1.030946
H 3.920222 1.002349 0.750889
C 3.608495 -1.901397 -1.766838
H 3.347301 -1.273404 -2.624508
H 4.677842 -2.138704 -1.778515
H 3.013062 -2.818551 -1.824018
C 3.748075 -2.036094 1.144332
H 3.436558 -1.517650 2.058070
H 3.246238 -3.008317 1.100277
H 4.835369 -2.171584 1.134316

V
E = -1277.88807407
C -1.780886 2.097975 0.011807
C 0.433554 1.138249 0.250994
C 0.951483 2.425968 0.385548
C 0.129073 3.552762 0.336217
C -1.232404 3.376278 0.136065
H -2.857098 2.014457 -0.132361
H 2.021356 2.552864 0.549233
H 0.545551 4.551352 0.450894
H -1.890069 4.243392 0.084115
B 1.205114 -0.207696 0.263928
Si -0.189364 -1.763245 0.359134
C 2.728728 -0.383366 0.047806
C 3.512780 0.519421 -0.696022
C 3.380255 -1.507670 0.588347
C 4.874559 0.317727 -0.876532

H 3.035432 1.381002 -1.160065
C 4.748429 -1.696347 0.442793
H 2.789087 -2.242513 1.134575
C 5.497518 -0.784116 -0.294917
H 5.457315 1.021787 -1.468399
H 5.231217 -2.565344 0.886709
H 6.567352 -0.937922 -0.427761
C -0.971249 0.959806 0.088535
C -1.415681 -0.448295 0.053138
P -3.078190 -0.856753 -0.154965
C -4.198385 -0.247950 1.148441
H -4.113685 0.838629 1.242528
H -5.239389 -0.517708 0.936874
H -3.885213 -0.698500 2.096345
C -3.807855 -0.274001 -1.718291
H -4.868113 -0.542476 -1.787206
H -3.698977 0.811511 -1.802282
H -3.254196 -0.737753 -2.541565
C -3.335127 -2.643834 -0.167239
H -4.403689 -2.854122 -0.290438
H -2.761128 -3.089280 -0.985211
H -2.966131 -3.069812 0.770828

4.2.2 Triplet state structures

$^{Tos}Y_2Si$
E = -4082.90541156
C 4.859768 2.174408 0.130888
H 4.835303 2.208169 1.217443
C 3.700360 1.851802 -0.556780
C -3.667937 -1.867943 -0.539401
O 1.240080 2.551265 -0.000834
C -3.652014 -1.824717 -1.929902
H -2.728349 -1.573317 -2.449757
C 2.528648 -3.152101 2.485982
H 3.479370 -3.499821 2.083963
C 6.026333 2.419122 -0.584591
H 6.941719 2.663649 -0.046786
C -1.346920 4.876984 -1.784432
H -0.968806 5.868600 -1.545341
C -1.598893 3.973453 -0.757312
H -1.397696 4.252492 0.273418
C 2.220309 -2.333760 -2.366884
H 2.543751 -1.322384 -2.610381
C 2.032401 -2.693937 -1.026992
C 5.021757 -1.440098 -0.664320
H 4.622829 -1.736473 -1.630823
C 6.386731 -1.223974 -0.524289
H 7.042050 -1.346199 -1.384422
C -1.557192 4.510213 -3.107470
H -1.355885 5.220304 -3.907449
C -1.591656 0.013989 -0.251737
Si 0.002961 0.011868 -1.153480
O -2.595525 -1.319331 1.773867
S -2.176909 -1.478840 0.365544
P -2.378641 1.466946 0.235562
C -4.819622 -2.089282 -2.628891
H -4.814108 -2.050709 -3.717717
C -4.704211 0.898651 1.660794
H -4.037911 0.756683 2.507744
C -0.600838 1.779382 2.314778

H -0.034161 1.012922 1.800179
C 6.067816 -0.695661 1.803486
H 6.472972 -0.398764 2.768843
C 4.700352 -0.901151 1.668139
H 4.036715 -0.742600 2.514235
C -2.246499 2.317079 -2.394631
H -2.557501 1.298983 -2.626096
C 1.810332 -2.146919 1.829178
C 3.687087 1.792129 -1.946670
H 2.761204 1.547630 -2.465813
C 1.552919 -3.962541 -0.709879
H 1.347700 -4.226025 0.324069
C 4.171238 -1.287334 0.433145
C -2.057422 3.758627 3.613876
H -2.628485 4.530643 4.126154
C -1.826362 2.187920 1.801227
C -2.560136 3.184887 2.453480
H -3.522088 3.506853 2.056792
C -2.062856 2.695290 -1.059025
C -2.006280 3.227633 -3.413155
H -2.152573 2.930742 -4.449734
C 7.326475 2.542750 -2.737748
H 7.142118 2.974031 -3.727986
H 7.844894 1.585516 -2.891448
H 8.015439 3.202212 -2.198819
C -0.822622 3.351342 4.117512
H -0.430066 3.812497 5.022159
C -6.007226 -2.407003 -1.959254
C 6.912192 -0.857818 0.710750
H 7.982615 -0.692986 0.819636
C -6.396294 1.176310 -0.533536
H -7.053785 1.281050 -1.394295
C 1.597249 0.003530 -0.251856
P 2.365909 -1.454278 0.251884
O 2.612626 1.348420 1.760268
S 2.201171 1.496200 0.348432
O -1.199134 -2.524220 0.032341
C 6.050474 2.339785 -1.977232
C 4.859945 2.031464 -2.645968
H 4.856395 1.979778 -3.734254
C 1.968826 -3.253227 -3.374640
H 2.118443 -2.970440 -4.414683
C 1.504546 -4.526573 -3.053547
H 1.294711 -5.243786 -3.844958
C 1.290142 -4.875048 -1.726158
H 0.900094 -5.859121 -1.475345
C -5.034379 1.408798 -0.676974
H -4.640064 1.700133 -1.646899
C -0.091952 2.365024 3.466942
H 0.885636 2.052121 3.825415
C -4.181159 1.278859 0.421369
C -6.915968 0.816292 0.705718
H -7.984048 0.638566 0.817263
C -5.985418 -2.469238 -0.565707
H -6.898421 -2.720886 -0.027133
C -6.068771 0.676752 1.799415
H -6.469311 0.384573 2.768126
C 0.804842 -3.261106 4.168694
H 0.411235 -3.703090 5.082409
C 2.025089 -3.701552 3.657686

H 2.584006 -4.480350 4.173077
C -4.824328 -2.198871 0.149440
H -4.802013 -2.219568 1.236352
C -7.277964 -2.638663 -2.720409
H -7.959805 -3.298819 -2.173352
H -7.084625 -3.082864 -3.703206
H -7.808578 -1.691009 -2.890752
C 0.089643 -2.266487 3.513753
H -0.877464 -1.928993 3.878135
C 0.599432 -1.705589 2.349771
H 0.043569 -0.934264 1.830507

^{Tos}YSiCl
E = -2646.89377311
C -3.156385 0.945617 -1.141665
H -2.791842 1.015883 -2.163701
C -2.338495 1.367313 -0.103917
O -0.712387 3.428191 -0.083565
C 2.449683 -1.595644 -1.978548
H 1.957181 -2.535326 -1.730318
C -4.409150 0.422202 -0.846707
H -5.051733 0.084787 -1.659147
C 0.791395 -1.237016 2.635311
H -0.066304 -0.570608 2.711153
C 1.472610 -1.348549 1.416414
C -1.439350 -2.028333 0.172562
H -1.265752 -2.026360 1.245726
C -2.546662 -2.690872 -0.339869
H -3.232120 -3.197262 0.336562
Si 1.327595 2.059541 1.795983
C -1.902027 -2.048083 -2.570513
H -2.085676 -2.046941 -3.642995
C -0.797697 -1.373700 -2.064020
H -0.139272 -0.821718 -2.729399
C 2.098585 -0.418362 -1.308611
C -2.765024 1.292310 1.217332
H -2.113425 1.642481 2.016316
C 2.594409 -2.171268 1.327311
H 3.150917 -2.243004 0.396792
C -0.553445 -1.375366 -0.688368
C -6.181021 -0.314806 0.783033
H -6.587986 0.056007 1.729947
H -6.088848 -1.406640 0.872141
H -6.913223 -0.115410 -0.007134
C -2.774589 -2.708131 -1.712465
H -3.640458 -3.231741 -2.113356
C 0.398148 1.160170 0.540494
P 0.839503 -0.422014 -0.009171
O -0.444251 1.643375 -1.874667
S -0.718139 2.022116 -0.475905
C -4.852877 0.306820 0.471636
C -4.013776 0.757400 1.496675
H -4.350044 0.690538 2.530677
C 1.218375 -1.958508 3.740611
H 0.684363 -1.866634 4.684010
C 2.336839 -2.782832 3.645653
H 2.677257 -3.340325 4.516019
C 3.024433 -2.883622 2.443071
H 3.907554 -3.514723 2.369167
C 4.021379 -0.353385 -3.313241

H 4.774755 -0.326971 -4.098300
C 3.414203 -1.561018 -2.976231
H 3.687753 -2.475864 -3.497992
C 3.658738 0.818416 -2.659714
H 4.121815 1.764107 -2.932419
C 2.693519 0.792150 -1.659100
H 2.389539 1.707656 -1.157323
Cl 3.052299 3.132407 1.260876

^{Tos}*YSiHMDS*
E = -3060.70706357
S 0.882594 -1.637772 0.793889
P 0.600688 1.148725 0.073240
Si -4.086256 0.218016 -0.750271
Si -3.376036 -2.651229 0.209417
O 0.153088 -2.906170 0.701512
O 1.079796 -1.029593 2.124380
N -2.937333 -1.116162 -0.587545
C 0.136095 -0.481267 -0.252170
C 2.521566 -1.945518 0.153171
C 2.689827 -2.230950 -1.198467
H 1.821533 -2.261323 -1.855006
C 3.966062 -2.455210 -1.688840
H 4.103338 -2.672737 -2.747580
C 5.083452 -2.410482 -0.845539
C 4.880931 -2.143582 0.508029
H 5.737999 -2.108049 1.179469
C 3.606906 -1.907873 1.013895
H 3.441645 -1.675813 2.063334
C 6.461042 -2.627421 -1.395952
H 6.503504 -3.523738 -2.026507
H 7.199663 -2.739293 -0.595538
H 6.773480 -1.780009 -2.021417
C 2.376788 1.350588 0.413801
C 3.271710 1.350862 -0.659486
H 2.900900 1.299931 -1.680904
C 4.638442 1.416349 -0.426874
H 5.329694 1.413824 -1.267056
C 5.119332 1.485965 0.877306
H 6.190973 1.541304 1.059421
C 4.231932 1.472037 1.947683
H 4.606335 1.509551 2.968720
C 2.862583 1.393643 1.722272
H 2.170194 1.336981 2.557965
C -0.259063 1.879909 1.494304
C 0.119443 3.109998 2.041656
H 0.952706 3.666642 1.614221
C -0.549584 3.610416 3.151447
H -0.253685 4.567503 3.576289
C -1.586027 2.879595 3.726276
H -2.103112 3.268211 4.601527
C -1.950680 1.648314 3.193433
H -2.747021 1.066177 3.653067
C -1.290224 1.144887 2.078742
H -1.559874 0.175069 1.663376
C 0.261279 2.154586 -1.399299
C 0.434152 1.570962 -2.659122
H 0.752923 0.532784 -2.728426
C 0.187991 2.310985 -3.808675
H 0.320274 1.849482 -4.784955

C -0.240314 3.631727 -3.709922
H -0.444781 4.206294 -4.611299
C -0.410926 4.214900 -2.460101
H -0.752971 5.244398 -2.378439
C -0.159196 3.480293 -1.305985
H -0.325785 3.937653 -0.334494
C -4.948697 0.533912 0.891908
H -5.685316 1.339881 0.773801
H -4.217058 0.859714 1.640887
H -5.472640 -0.340401 1.292128
C -3.196528 1.805519 -1.195589
H -3.933574 2.616430 -1.281121
H -2.645746 1.743257 -2.142884
H -2.485776 2.086437 -0.409453
C -5.348939 -0.140211 -2.091889
H -5.979625 -1.003366 -1.853172
H -4.838462 -0.347977 -3.041167
H -6.005715 0.726712 -2.243704
C -2.950915 -2.476026 2.025823
H -3.462789 -1.606535 2.459854
H -1.867753 -2.339416 2.131549
H -3.237513 -3.365016 2.602294
C -2.497356 -4.092759 -0.591268
H -2.856696 -5.028635 -0.141094
H -1.414078 -4.034729 -0.448721
H -2.719959 -4.126849 -1.665765
C -5.216194 -3.002865 0.002814
H -5.450577 -3.209580 -1.049162
H -5.886640 -2.210222 0.351033
H -5.454947 -3.909532 0.575338
Si -1.316707 -0.968216 -1.249992

$^{Tos}YSiC_6F_5$
E = -2915.23607959
C 3.969844 -1.244850 -1.503473
H 3.980249 -0.477796 -2.273848
C 2.752076 -1.710037 -1.032832
O 0.557996 -2.127119 -2.417763
C 0.475739 3.708479 -0.327328
H 1.058037 3.877616 0.578195
C 5.145913 -1.755470 -0.964563
H 6.104266 -1.386718 -1.327983
C 0.025491 0.073028 2.824260
H 0.629448 -0.792001 2.558346
C -0.139986 1.120418 1.908477
C 3.138134 0.652104 1.578712
H 2.591016 0.100307 2.337741
C 4.517794 0.762378 1.683081
H 5.033375 0.293559 2.518600
C 4.570878 2.054425 -0.347766
H 5.129138 2.595294 -1.109191
C 3.190574 1.939160 -0.463856
H 2.678687 2.358409 -1.325881
C 0.141930 2.407021 -0.716101
C 2.696739 -2.690231 -0.046727
H 1.732882 -3.053240 0.307916
C -0.971020 2.192268 2.233654
H -1.152489 2.983369 1.511607
C 2.463635 1.250743 0.511070
C 6.388668 -3.239296 0.650478

H 6.374053 -4.332371 0.735268
H 6.529392 -2.840132 1.664403
H 7.264237 -2.954614 0.058037
C 5.235534 1.470642 0.724040
H 6.317002 1.558548 0.808717
C 0.272642 -0.551082 -0.368496
P 0.672615 1.000701 0.292575
O 1.653602 0.166557 -2.460341
S 1.241898 -1.040693 -1.720674
C 5.121030 -2.720427 0.041424
C 3.876544 -3.183425 0.486597
H 3.835537 -3.946726 1.262800
C -0.596693 0.125652 4.064630
H -0.460137 -0.690526 4.770694
C -1.407968 1.207670 4.390935
H -1.903093 1.243407 5.359370
C -1.600561 2.233369 3.473206
H -2.252762 3.069530 3.715915
C -0.649176 4.565440 -2.274293
H -0.960916 5.410272 -2.885551
C 0.073528 4.786318 -1.104449
H 0.329485 5.799344 -0.800960
C -0.964564 3.270211 -2.668705
H -1.517319 3.098257 -3.589572
C -0.569545 2.187242 -1.892520
H -0.792394 1.168464 -2.198058
Si -1.012517 -1.690065 0.205461
C -2.824835 -1.337636 -0.056281
C -5.113486 -2.179659 -0.191512
C -4.706698 0.186529 -0.364659
C -5.594830 -0.884272 -0.355667
C -3.351052 -0.052864 -0.206994
C -3.751113 -2.384013 -0.040717
F -3.322531 -3.632495 0.117282
F -2.542139 1.007648 -0.180238
F -5.958317 -3.197666 -0.184024
F -6.891346 -0.671248 -0.497766
F -5.165203 1.420951 -0.505009

^{Tos}YSiPy
E = -2434.44288792
C -3.244091 -1.579776 1.074514
H -2.812831 -1.767016 2.054936
C -2.418361 -1.569107 -0.036472
O -0.309152 -2.899109 -0.853411
C 1.823974 2.240582 2.105955
H 1.173553 3.097201 1.930539
C -4.603977 -1.337673 0.902927
H -5.260973 -1.337429 1.771555
C 0.278102 1.832844 -2.558274
H -0.355296 0.958959 -2.699165
C 0.843470 2.078494 -1.302758
C -2.175853 1.966225 0.058579
H -2.027246 2.132891 -1.006990
C -3.414000 2.213813 0.634711
H -4.232606 2.580932 0.019025
C -2.566126 1.502640 2.776069
H -2.720222 1.310468 3.835965
C -1.324367 1.246056 2.204207
H -0.519180 0.834696 2.809160

C 1.674905 1.075288 1.345010
C -2.921945 -1.332041 -1.312993
H -2.245280 -1.330428 -2.168717
C 1.704176 3.163709 -1.130323
H 2.180189 3.342381 -0.170185
C -1.121793 1.494596 0.844982
C -6.600396 -0.810360 -0.542354
H -7.054614 -1.523817 -1.241381
H -6.763034 0.192490 -0.958510
H -7.143328 -0.873761 0.406035
C -3.607488 1.989830 1.995435
H -4.579325 2.184879 2.444861
C 0.175386 -0.583413 -0.632941
P 0.415780 0.946237 0.044153
O -0.426111 -2.047647 1.574447
S -0.661442 -1.778648 0.154952
C -5.138795 -1.087311 -0.359407
C -4.276504 -1.094423 -1.465223
H -4.680023 -0.909350 -2.459949
C 0.556435 2.682188 -3.622132
H 0.121239 2.480537 -4.598565
C 1.401577 3.771726 -3.442883
H 1.623370 4.432017 -4.279114
C 1.977871 4.008211 -2.199214
H 2.653823 4.849502 -2.060950
C 3.591928 1.187316 3.356188
H 4.344505 1.232418 4.141270
C 2.785397 2.296060 3.105403
H 2.902026 3.202174 3.696687
C 3.425057 0.021058 2.619660
H 4.039371 -0.853686 2.822395
C 2.462353 -0.041481 1.617217
H 2.299822 -0.961025 1.061017
Si 0.794622 -1.798271 -2.056291
C 2.450523 -2.236467 -1.179101
N 3.391385 -1.325908 -1.482224
C 3.942844 -3.344898 0.347878
C 4.574455 -1.400244 -0.886340
C 4.904628 -2.388195 0.037792
H 4.160230 -4.135129 1.066138
H 5.305931 -0.632849 -1.152343
H 5.891236 -2.405228 0.496692
C 2.699956 -3.270070 -0.268644
H 1.906696 -3.976872 -0.031398

^{Tos}YSiPyr
E = -2450.44899349
C 3.741865 0.114333 -1.334767
H 3.582819 1.069220 -1.830094
C 2.700314 -0.797341 -1.282694
O 0.935310 -1.342394 -3.145081
C -1.372769 3.076864 0.615603
H -0.934517 3.072023 1.613368
C 4.953686 -0.204841 -0.730278
H 5.772160 0.513287 -0.761357
C -0.804507 -1.544282 2.130261
H 0.011620 -2.045852 1.613306
C -1.205852 -0.268590 1.719337
C 1.981143 0.435462 1.922444
H 1.582093 -0.488104 2.334062

C 3.223039 0.890711 2.342696
H 3.785739 0.319361 3.078018
C 3.025769 2.790935 0.876196
H 3.436193 3.707481 0.457239
C 1.784586 2.336644 0.445276
H 1.240997 2.879004 -0.323598
C -1.228403 1.967138 -0.221341
C 2.859320 -2.027139 -0.650668
H 2.030126 -2.732783 -0.621946
C -2.279223 0.355485 2.353963
H -2.626689 1.326629 2.011993
C 1.248424 1.163000 0.980773
C 6.429489 -1.748424 0.609688
H 6.794437 -2.740963 0.318819
H 6.307761 -1.758746 1.701397
H 7.206916 -1.016238 0.368610
C 3.743677 2.073412 1.825453
H 4.716774 2.430368 2.157501
C -0.071326 -0.705910 -0.866707
P -0.321834 0.502095 0.337516
O 1.199603 1.054011 -2.331886
S 1.136125 -0.390293 -2.049692
C 5.135462 -1.420383 -0.072536
C 4.069453 -2.328750 -0.047815
H 4.195052 -3.288082 0.453085
C -1.455167 -2.172533 3.186211
H -1.138786 -3.165002 3.499674
C -2.516864 -1.541005 3.823616
H -3.031735 -2.037391 4.643917
C -2.929797 -0.280356 3.404805
H -3.770808 0.208958 3.891621
C -2.592904 4.212833 -1.120006
H -3.128130 5.092084 -1.473993
C -2.063046 4.196356 0.167215
H -2.179330 5.059586 0.819390
C -2.427703 3.115963 -1.959830
H -2.827352 3.137056 -2.971418
C -1.745513 1.990620 -1.514224
H -1.597186 1.128899 -2.159882
Si -1.161296 -2.132486 -1.153332
C -2.975585 -1.810457 -0.958167
N -3.866348 -2.809124 -1.108632
N -3.289766 -0.547383 -0.617322
C -5.135584 -2.506776 -0.909861
C -4.566430 -0.264525 -0.421661
C -5.563886 -1.224707 -0.556647
H -5.858528 -3.317304 -1.034421
H -4.804941 0.767265 -0.148565
H -6.613538 -0.992495 -0.398328

^{Tos}YSiB2
E = -2593.40539726
C 2.928538 -2.358075 -1.287294
H 2.628996 -2.100066 -2.300323
C 2.002430 -2.244789 -0.262590
O -0.562660 -2.789926 -0.330007
C 0.222921 3.242622 -1.729188
H 1.179615 3.642377 -1.393938
C 4.221787 -2.774307 -0.989359
H 4.954514 -2.858188 -1.791112

C 1.128230 1.522039 2.764466
H 1.406491 0.469388 2.752850
C 0.692426 2.134513 1.582596
C 3.442568 0.919931 0.312051
H 3.277708 0.935363 1.386482
C 4.731978 0.769587 -0.180746
H 5.564658 0.665342 0.511705
Si -1.252219 -0.711544 1.757411
C 3.886535 0.874357 -2.434310
H 4.056205 0.847056 -3.508743
C 2.592521 1.017758 -1.948192
H 1.751789 1.073565 -2.634487
C -0.264426 2.043389 -1.197852
C 2.345935 -2.559335 1.048980
H 1.600805 -2.472487 1.838741
C 0.293317 3.470854 1.607233
H -0.078569 3.949612 0.705487
C 2.367790 1.055333 -0.570141
C 6.009458 -3.473723 0.643961
H 6.034699 -4.308858 1.353757
H 6.558429 -2.641650 1.106568
H 6.559412 -3.772136 -0.254616
C 4.955635 0.753208 -1.554129
H 5.967237 0.637372 -1.938666
C 0.013999 -0.400990 0.478753
P 0.664959 1.137670 0.065101
O 0.406882 -1.130693 -1.996437
S 0.345471 -1.682451 -0.630821
C 4.602219 -3.071747 0.318806
C 3.640781 -2.963433 1.331571
H 3.915652 -3.204595 2.357896
C 1.185572 2.247835 3.946204
H 1.523858 1.764088 4.860030
C 0.795017 3.583526 3.963254
H 0.831726 4.149599 4.891941
C 0.345154 4.190140 2.796676
H 0.023440 5.229409 2.809877
C -1.715399 3.386087 -3.148952
H -2.285150 3.911833 -3.912971
C -0.506466 3.914076 -2.700560
H -0.129025 4.846711 -3.115083
C -2.187897 2.184664 -2.633913
H -3.124433 1.763527 -2.993624
C -1.462806 1.504447 -1.660737
H -1.814710 0.551021 -1.273822
C -5.248546 -1.346196 0.969977
C -4.782522 -1.049911 -0.309232
C -6.574291 -1.650148 1.209624
C -5.617706 -1.045526 -1.408749
C -7.428274 -1.646558 0.104145
H -6.924807 -1.880945 2.211923
C -6.960741 -1.351277 -1.176212
H -5.237932 -0.820944 -2.402022
H -8.480876 -1.881919 0.246317
H -7.655469 -1.362262 -2.013352
B -3.094471 -0.938968 1.095035
O -4.211585 -1.277030 1.848032
O -3.449789 -0.788815 -0.244083

^{Tos}YSiB1
E = -2862.58972114
C 4.149488 0.909441 0.529567
H 3.884320 1.357635 1.484152
C 3.207209 0.891117 -0.487279
O 1.459767 2.734056 -1.120493
C -1.217997 -1.922399 2.486224
H -0.781078 -2.903718 2.308487
C 5.392957 0.329311 0.311707
H 6.130974 0.330685 1.113195
C 0.521108 -2.533305 -1.992442
H 1.298522 -1.809884 -2.234137
C -0.215844 -2.376158 -0.809989
C 2.741880 -2.377952 0.514452
H 2.548974 -2.775457 -0.478266
C 3.922605 -2.709742 1.165977
H 4.644092 -3.359179 0.673900
Si -0.894043 0.747382 -1.876782
C 3.259536 -1.375501 3.058084
H 3.462215 -0.976375 4.049863
C 2.077812 -1.036140 2.411019
H 1.371305 -0.359031 2.884362
C -0.902144 -0.847077 1.648299
C 3.503834 0.330671 -1.724692
H 2.755444 0.349587 -2.515567
C -1.257327 -3.256758 -0.526417
H -1.888349 -3.109193 0.345265
C 1.808712 -1.546018 1.137785
C 7.023967 -0.960493 -1.106069
H 7.296166 -1.014207 -2.165664
H 6.986687 -1.991278 -0.724850
H 7.830120 -0.447499 -0.569771
C 4.180403 -2.213901 2.439924
H 5.105185 -2.477703 2.949802
C 0.461327 0.385619 -0.694839
P 0.284719 -1.018556 0.284426
O 1.502540 1.820328 1.248604
S 1.586995 1.597713 -0.209148
C 5.706116 -0.274187 -0.907321
C 4.746109 -0.253468 -1.924846
H 4.980676 -0.701780 -2.889717
C 0.242942 -3.583092 -2.853642
H 0.819217 -3.697140 -3.769520
C -0.780060 -4.479662 -2.551570
H -0.999948 -5.303310 -3.228125
C -1.531059 -4.307687 -1.396997
H -2.354306 -4.982051 -1.172689
C -2.586172 -0.464790 3.829691
H -3.247295 -0.316006 4.681600
C -2.070192 -1.732168 3.565077
H -2.320945 -2.570749 4.211724
C -2.240848 0.611806 3.021217
H -2.624163 1.606975 3.234083
C -1.397090 0.424435 1.930237
H -1.083942 1.263474 1.313484
N -3.658725 0.796501 -0.432696
C -3.653453 2.819680 0.525534
C -4.306548 1.635832 0.465116
H -3.910457 3.711913 1.085631
H -5.223011 1.339611 0.962048

B -2.505642 1.495357 -0.953069
C -1.595784 3.817814 -0.389870
C -1.288957 4.414522 -1.610381
C -0.918690 4.203676 0.764721
C -0.307717 5.393987 -1.671535
H -1.823080 4.096719 -2.502838
C 0.062354 5.183831 0.695760
H -1.142254 3.710232 1.708772
C 0.367841 5.782465 -0.520176
H -0.063622 5.851467 -2.628142
H 0.605369 5.462829 1.596226
H 1.145689 6.541297 -0.574192
C -4.009119 -0.554903 -0.584263
C -3.972089 -1.154469 -1.845646
C -4.412206 -1.308806 0.520929
C -4.361271 -2.477993 -1.998152
H -3.660095 -0.563252 -2.703097
C -4.809619 -2.631021 0.357765
H -4.393791 -0.855578 1.509836
C -4.794781 -3.219209 -0.902428
H -4.333751 -2.931317 -2.987138
H -5.128222 -3.204435 1.226567
H -5.115553 -4.251344 -1.030631
N -2.565681 2.788346 -0.331942

$^{Ph}Y_2Si$
E = -2905.70713866
P 2.606626 0.456204 -0.103103
P -2.656625 0.321122 -0.154571
C 1.795072 -0.552514 -1.222852
C 2.113545 -1.974842 -1.385480
C 3.058181 -2.661300 -0.597059
H 3.637517 -2.123264 0.147743
C 3.257718 -4.031661 -0.718050
H 3.992061 -4.517438 -0.076377
C 2.523419 -4.781193 -1.627853
H 2.669928 -5.856124 -1.711206
C 1.590595 -4.123392 -2.427815
H 0.998595 -4.685086 -3.149155
C 1.398305 -2.757723 -2.315202
H 0.652252 -2.263004 -2.938587
C 2.128212 0.177544 1.634159
C 2.824431 -0.739729 2.428530
H 3.791716 -1.118858 2.105105
C 2.285002 -1.178563 3.632363
H 2.833742 -1.898148 4.236864
C 1.048699 -0.704998 4.058943
H 0.621859 -1.057538 4.995807
C 0.353988 0.218209 3.283136
H -0.615480 0.587825 3.606696
C 0.886612 0.652673 2.077330
H 0.316839 1.341732 1.459882
C 2.307248 2.210770 -0.457098
C 2.347314 3.164798 0.563406
H 2.419693 2.850332 1.602581
C 2.306630 4.522777 0.250414
H 2.334652 5.259663 1.050926
C 2.243138 4.933131 -1.074624
H 2.221223 5.994172 -1.316632
C 2.207686 3.982317 -2.096206

H 2.160244 4.298774 -3.136038
C 2.243258 2.629940 -1.791869
H 2.233250 1.881552 -2.582065
C 4.432529 0.367546 -0.132452
C 5.051884 -0.151461 -1.269944
H 4.437814 -0.568271 -2.066728
C 6.438430 -0.143165 -1.368448
H 6.919268 -0.557375 -2.252349
C 7.208234 0.389245 -0.339240
H 8.293810 0.394211 -0.418447
C 6.592465 0.917532 0.791717
H 7.193711 1.339433 1.594818
C 5.206726 0.908709 0.895793
H 4.723993 1.323938 1.779876
C -1.262327 -0.546922 -0.575624
C -1.185116 -1.994693 -0.230759
C -0.239761 -2.455946 0.698565
H 0.409991 -1.734912 1.183093
C -0.095080 -3.809051 0.972940
H 0.669236 -4.132059 1.677647
C -0.905071 -4.745298 0.339343
H -0.785767 -5.807027 0.547408
C -1.857771 -4.310708 -0.576346
H -2.491904 -5.032480 -1.089208
C -1.990108 -2.956810 -0.861696
H -2.713767 -2.635895 -1.606277
C -2.991009 0.334546 1.638878
C -3.083911 1.526091 2.364766
H -3.026806 2.483197 1.850464
C -3.258900 1.494318 3.744584
H -3.337361 2.427993 4.298568
C -3.338819 0.276426 4.413081
H -3.476475 0.253631 5.492425
C -3.244608 -0.913629 3.694885
H -3.306153 -1.869817 4.210953
C -3.073195 -0.890235 2.316933
H -3.002286 -1.825756 1.764949
C -2.575993 2.055077 -0.674012
C -3.688157 2.683441 -1.237502
H -4.605749 2.124209 -1.401913
C -3.627329 4.027245 -1.594289
H -4.498146 4.506162 -2.037319
C -2.459490 4.751174 -1.385831
H -2.412735 5.802054 -1.665997
C -1.350642 4.134301 -0.815083
H -0.434097 4.691943 -0.643560
C -1.405722 2.791770 -0.459452
H -0.532473 2.316759 -0.021591
C -4.217174 -0.297832 -0.888124
C -5.402465 -0.448181 -0.169971
H -5.428597 -0.216970 0.892922
C -6.552976 -0.896494 -0.813882
H -7.474430 -1.022875 -0.248654
C -6.525068 -1.182237 -2.173836
H -7.425860 -1.532616 -2.674422
C -5.343821 -1.025499 -2.896363
H -5.320564 -1.250398 -3.960928
C -4.192344 -0.591321 -2.254505
H -3.253477 -0.486883 -2.800493
Si 0.104709 0.067872 -1.646102

^{Ph}YSiCl
E = -2058.30841460
P -0.335285 -0.309527 0.006673
C 0.537794 1.078539 -0.554181
C 2.000798 1.118730 -0.529362
C 2.814566 -0.020403 -0.651534
H 2.352239 -0.991041 -0.823049
C 4.199354 0.073278 -0.596693
H 4.804105 -0.826986 -0.693461
C 4.812363 1.311110 -0.437142
H 5.897376 1.385866 -0.398380
C 4.023602 2.456877 -0.338309
H 4.493177 3.431766 -0.218213
C 2.642265 2.363697 -0.384003
H 2.023753 3.256398 -0.298946
C -0.448633 -1.720991 -1.141110
C -0.899418 -2.977816 -0.722315
H -1.173314 -3.139930 0.319549
C -0.989198 -4.022407 -1.633065
H -1.341366 -4.998399 -1.305046
C -0.625458 -3.820549 -2.962321
H -0.693570 -4.641377 -3.673729
C -0.170799 -2.574995 -3.380034
H 0.118869 -2.417330 -4.416774
C -0.081553 -1.525082 -2.471983
H 0.278397 -0.545429 -2.787125
C -2.029911 0.187119 0.420199
C -3.132110 -0.627222 0.152584
H -3.006463 -1.570274 -0.372196
C -4.406525 -0.215318 0.527318
H -5.262362 -0.849081 0.303770
C -4.589153 1.002351 1.171509
H -5.589194 1.323105 1.456686
C -3.491825 1.816125 1.442434
H -3.627691 2.778893 1.930112
C -2.217978 1.412918 1.072028
H -1.365915 2.066149 1.247948
C 0.408346 -1.002137 1.525900
C 1.257902 -2.111176 1.496908
H 1.435611 -2.641247 0.563974
C 1.882380 -2.545882 2.659792
H 2.541948 -3.410591 2.625075
C 1.668998 -1.876812 3.859583
H 2.159537 -2.218242 4.768999
C 0.828807 -0.768827 3.894409
H 0.658432 -0.240084 4.829877
C 0.203247 -0.331342 2.734362
H -0.451134 0.535913 2.772389
Si -0.464045 2.153234 -1.667126
Cl -1.053747 4.007697 -0.795390

^{Ph}YSiHMDS
E = -2472.11061784
P -1.696551 -0.208925 -0.090899
C -0.230296 0.483146 -0.648177
C 0.131330 1.887758 -0.481702
C -0.489085 2.738335 0.454647
H -1.267139 2.341697 1.103865
C -0.110377 4.066753 0.593914

H -0.617756 4.689270 1.329599
C 0.920819 4.597276 -0.174773
H 1.225697 5.634654 -0.054471
C 1.559789 3.770385 -1.096249
H 2.368865 4.162639 -1.711537
C 1.171391 2.448654 -1.249355
H 1.669014 1.819903 -1.986112
C -1.729239 -0.674913 1.665529
C -2.190794 0.213222 2.643903
H -2.758237 1.096511 2.354377
C -1.918826 -0.017793 3.985888
H -2.284824 0.678548 4.737612
C -1.170474 -1.129780 4.367271
H -0.949332 -1.302600 5.418681
C -0.708038 -2.018164 3.402817
H -0.120046 -2.886319 3.694766
C -0.986762 -1.799581 2.058271
H -0.612851 -2.489822 1.303796
C -2.013544 -1.741759 -1.015718
C -2.744247 -2.791093 -0.451653
H -3.082015 -2.727296 0.580922
C -3.033353 -3.921187 -1.206709
H -3.595664 -4.738983 -0.760340
C -2.604431 -4.007887 -2.527745
H -2.828891 -4.896265 -3.115027
C -1.896056 -2.955811 -3.099334
H -1.568624 -3.016038 -4.135265
C -1.606352 -1.820908 -2.351954
H -1.061677 -0.988667 -2.793475
C -3.160795 0.820566 -0.399000
C -3.092464 1.844920 -1.345280
H -2.145805 2.061747 -1.834649
C -4.231056 2.581471 -1.649643
H -4.172464 3.384626 -2.381304
C -5.438937 2.294958 -1.021805
H -6.328227 2.874398 -1.262708
C -5.513153 1.264565 -0.089513
H -6.458985 1.033015 0.396190
C -4.378042 0.525594 0.220294
H -4.437089 -0.284745 0.945827
Si 1.098884 -0.770332 -0.868956
N 2.705045 -0.401101 -0.260326
Si 4.053487 -0.301346 -1.401348
Si 2.919100 -0.435011 1.504411
C 3.431361 -0.429111 -3.168197
H 2.927180 -1.384186 -3.359253
H 2.729695 0.373001 -3.428099
H 4.292842 -0.356302 -3.847502
C 4.959951 1.335023 -1.234894
H 5.592160 1.383513 -0.342491
H 5.605072 1.491230 -2.109789
H 4.241327 2.162972 -1.187389
C 5.224046 -1.743645 -1.113511
H 6.092444 -1.690608 -1.783210
H 5.597677 -1.780388 -0.083256
H 4.702199 -2.689446 -1.309719
C 4.601566 0.244390 2.000949
H 4.710724 0.107372 3.085478
H 5.451130 -0.252724 1.519442
H 4.668388 1.319435 1.796319

C 2.810740 -2.220078 2.078164
H 2.792038 -2.296405 3.173270
H 1.904832 -2.695372 1.683155
H 3.674451 -2.788567 1.707789
C 1.658644 0.655940 2.348761
H 0.627288 0.409144 2.082264
H 1.759465 0.565130 3.438875
H 1.829599 1.703667 2.070457

$^{Ph}YSiC_6F_5$
E = -2326.65839556
P -1.249429 -0.576874 0.148487
C -0.861601 0.563189 -1.104196
C -1.739203 1.700335 -1.367141
C -3.097898 1.739899 -0.996304
H -3.553037 0.876045 -0.513436
C -3.882798 2.859663 -1.236360
H -4.928259 2.854849 -0.932355
C -3.345180 3.974919 -1.869258
H -3.962081 4.850028 -2.063169
C -2.005511 3.954290 -2.255491
H -1.568291 4.820493 -2.749452
C -1.218724 2.842321 -2.007700
H -0.168128 2.839724 -2.295494
C -1.894650 0.306466 1.598712
C -2.923610 -0.191353 2.398162
H -3.392722 -1.143599 2.162702
C -3.365782 0.543670 3.492600
H -4.171178 0.155048 4.112507
C -2.786336 1.772406 3.789548
H -3.137677 2.346021 4.645071
C -1.765339 2.274047 2.987929
H -1.318707 3.240820 3.210160
C -1.320771 1.549253 1.890180
H -0.538116 1.945907 1.244937
C 0.243485 -1.456637 0.681487
C 1.039919 -0.944938 1.706788
H 0.712351 -0.077312 2.274902
C 2.266822 -1.536080 1.991566
H 2.891217 -1.127027 2.783206
C 2.697543 -2.636984 1.258725
H 3.666089 -3.085443 1.471606
C 1.898678 -3.159648 0.246138
H 2.237988 -4.015446 -0.332620
C 0.673858 -2.571970 -0.046409
H 0.053847 -2.971696 -0.846202
C -2.431382 -1.885607 -0.292267
C -3.141967 -1.761003 -1.486149
H -2.968266 -0.893771 -2.122175
C -4.047195 -2.748494 -1.859701
H -4.600185 -2.649951 -2.791400
C -4.235469 -3.862459 -1.049320
H -4.940734 -4.636720 -1.345256
C -3.513156 -3.999026 0.133884
H -3.649604 -4.879551 0.758521
C -2.608094 -3.015799 0.512288
H -2.025277 -3.132155 1.425612
C 2.190449 0.398634 -1.243864
C 3.486684 1.596815 0.444946
C 4.428496 -0.347991 -0.608711

C 4.536289 0.695220 0.303246
Si 0.565180 0.031408 -2.131634
C 3.274585 -0.472007 -1.366348
C 2.347781 1.444164 -0.330692
F 1.378433 2.345458 -0.159234
F 3.194139 -1.512934 -2.192126
F 5.408849 -1.234370 -0.712401
F 5.627320 0.824020 1.041740
F 3.590163 2.593979 1.313152

^{Ph}YSiPy
E = -1845.84660072
P -1.085754 -0.116716 -0.005344
C 0.596140 -0.451050 0.178718
C 1.491246 0.725072 0.021934
C 1.643993 1.680976 1.036274
C 2.542109 2.734577 0.901996
C 3.313438 2.855775 -0.249145
C 3.169387 1.918706 -1.268443
C 2.263907 0.874092 -1.138162
C -1.839173 0.731852 1.425258
C -2.879199 1.653220 1.287729
H -3.238559 1.926326 0.297449
C -3.454127 2.223435 2.418448
H -4.261119 2.945262 2.308862
C -2.998470 1.873001 3.685400
H -3.449408 2.322384 4.568198
C -1.964715 0.951427 3.825191
H -1.607170 0.676944 4.815616
C -1.383760 0.383693 2.698054
H -0.565940 -0.331686 2.789130
C -2.059032 -1.624814 -0.241897
C -3.221992 -1.860020 0.491229
H -3.547133 -1.146207 1.244281
C -3.963623 -3.015121 0.266957
H -4.865463 -3.197678 0.847551
C -3.551507 -3.933107 -0.691482
H -4.132031 -4.837512 -0.863618
C -2.394128 -3.699897 -1.428885
H -2.065052 -4.420355 -2.174622
C -1.645171 -2.552761 -1.204834
H -0.731546 -2.373710 -1.767807
C -1.442318 0.981719 -1.412920
C -0.942738 2.289897 -1.385312
H -0.395316 2.648052 -0.515820
C -1.144320 3.134792 -2.468390
H -0.747248 4.147370 -2.440012
C -1.846989 2.688113 -3.583532
H -2.002982 3.352599 -4.431270
C -2.352438 1.393680 -3.611679
H -2.908650 1.042156 -4.478379
C -2.151940 0.540745 -2.531297
H -2.554972 -0.468781 -2.560954
C 3.039830 -1.698525 0.571324
C 5.124816 -0.669798 1.191303
C 4.833934 -1.850973 -0.861680
C 5.654271 -1.111531 -0.017355
Si 1.161945 -2.122995 0.622986
N 3.569024 -2.144075 -0.578179
H 5.733333 -0.088240 1.882909

H 6.682457 -0.893838 -0.299026
H 5.220116 -2.223602 -1.813748
H 1.060203 1.579452 1.949966
H 2.648489 3.458384 1.708795
H 4.024921 3.673306 -0.350021
H 3.770711 2.000451 -2.172464
H 2.163765 0.130139 -1.925800
C 3.804720 -0.971191 1.490406
H 3.351616 -0.615934 2.415139

^{Ph}YSiPyr
E = -1861.85620551
P -0.808352 -0.439517 -0.026802
C 0.246055 0.592966 -0.898489
C -0.009674 2.053789 -0.897174
C 0.893623 2.923353 -0.265947
C 0.680599 4.296688 -0.261060
C -0.440628 4.836159 -0.884061
C -1.341731 3.989511 -1.523344
C -1.125700 2.617483 -1.534741
C -0.459786 -0.544429 1.761724
C -1.418209 -1.010185 2.668534
H -2.406473 -1.303921 2.316791
C -1.111216 -1.090427 4.020462
H -1.856980 -1.452735 4.725533
C 0.147754 -0.700231 4.472085
H 0.384865 -0.759139 5.532906
C 1.096038 -0.229350 3.571570
H 2.077774 0.082193 3.923205
C 0.797572 -0.147323 2.214354
H 1.540443 0.230075 1.509454
C -0.737101 -2.132874 -0.672210
C -0.651154 -3.242916 0.168068
H -0.587052 -3.109272 1.245153
C -0.627133 -4.523929 -0.373968
H -0.548377 -5.385785 0.285486
C -0.695512 -4.702200 -1.750593
H -0.674059 -5.705888 -2.170888
C -0.783223 -3.597271 -2.593080
H -0.827697 -3.733025 -3.671623
C -0.798589 -2.315998 -2.059254
H -0.845270 -1.445921 -2.711868
C -2.553220 0.080835 -0.057260
C -2.918869 1.197067 0.702388
H -2.180636 1.688427 1.334668
C -4.215984 1.687164 0.640411
H -4.491243 2.560744 1.227678
C -5.158092 1.065106 -0.174001
H -6.175339 1.449244 -0.220628
C -4.800761 -0.048925 -0.925364
H -5.537310 -0.541327 -1.557173
C -3.500639 -0.540872 -0.870832
H -3.227760 -1.414154 -1.459230
C 3.403647 0.011034 -0.800805
C 5.639078 -0.435495 -0.909672
C 4.716230 0.703824 0.938411
C 5.848913 0.184215 0.322944
Si 1.744383 -0.101542 -1.663800
N 3.511148 0.622794 0.395837
N 4.447536 -0.524129 -1.468425

H 6.475235 -0.874792 -1.460619
H 6.835091 0.255950 0.773763
H 4.786010 1.204549 1.907771
H 1.769041 2.492829 0.219303
H 1.395880 4.950809 0.235264
H -0.607850 5.911836 -0.879137
H -2.215182 4.401786 -2.026259
H -1.823715 1.960597 -2.050784

^{Ph}YSiB2
E = -2004.81483694
P 1.270373 -0.493673 0.025065
C 0.415562 0.816715 -0.689930
C 1.025091 2.166817 -0.677297
C 1.475891 2.779418 -1.856462
C 2.021584 4.057626 -1.842409
C 2.142599 4.756807 -0.646110
C 1.699102 4.167053 0.533740
C 1.142453 2.894882 0.517345
C 2.511469 -1.317463 -1.036763
C 3.523039 -2.108029 -0.481786
H 3.589856 -2.228444 0.598756
C 4.448793 -2.732690 -1.307802
H 5.235183 -3.346887 -0.873351
C 4.373657 -2.566547 -2.688428
H 5.102678 -3.052840 -3.333947
C 3.374137 -1.774465 -3.241963
H 3.318179 -1.638338 -4.320041
C 2.444116 -1.147968 -2.418551
H 1.659297 -0.521272 -2.841535
C 0.114138 -1.781064 0.560347
C 0.342198 -3.141687 0.352985
H 1.236656 -3.475003 -0.167915
C -0.592251 -4.072933 0.793604
H -0.418362 -5.133069 0.620885
C -1.749593 -3.651876 1.439483
H -2.481700 -4.384450 1.774032
C -1.977787 -2.294635 1.649837
H -2.888784 -1.959564 2.142128
C -1.049817 -1.359217 1.212226
H -1.236523 -0.295421 1.343188
C 2.250681 0.013988 1.467268
C 3.417342 0.751833 1.242287
H 3.744636 0.953852 0.223358
C 4.149308 1.238398 2.316222
H 5.051692 1.818675 2.135564
C 3.725379 0.989211 3.618593
H 4.300711 1.370808 4.459937
C 2.568722 0.251513 3.845668
H 2.239076 0.051048 4.863099
C 1.828278 -0.234872 2.773196
H 0.923362 -0.810921 2.954353
H 1.391131 2.231423 -2.794139
H 2.360035 4.507821 -2.774334
H 2.572977 5.756414 -0.633940
H 1.775327 4.707289 1.476124
H 0.777037 2.449291 1.440652
Si -0.929884 0.363279 -1.854755
C -4.464100 0.639668 0.271077
C -4.949962 0.202231 -0.959957

C -5.297330 0.847244 1.353183
C -6.293372 -0.045480 -1.165039
C -6.657906 0.597315 1.155412
H -4.904877 1.192585 2.306125
C -7.144536 0.161245 -0.076431
H -6.659607 -0.385601 -2.129938
H -7.351330 0.748721 1.980012
H -8.210501 -0.022022 -0.194018
B -2.763608 0.434993 -1.120854
O -3.913241 0.078266 -1.827111
O -3.117591 0.795722 0.184428

^{Ph}YSiB1
E = -2274.00252995
P -0.609489 1.368202 -0.080558
C 0.720028 0.619063 -0.875276
C 2.096997 1.008116 -0.556751
C 2.479277 1.503117 0.705955
H 1.737842 1.584575 1.499758
C 3.787101 1.887503 0.972699
H 4.038452 2.269766 1.961163
C 4.768703 1.781386 -0.005317
H 5.793518 2.082882 0.202342
C 4.418815 1.268453 -1.252657
H 5.175747 1.161506 -2.028301
C 3.113800 0.888379 -1.523094
H 2.849918 0.494522 -2.504059
C -1.156558 0.524429 1.444846
C -2.389202 0.787910 2.051494
H -3.094720 1.469116 1.577703
C -2.716560 0.177940 3.256032
H -3.676045 0.386584 3.725440
C -1.818186 -0.698819 3.859600
H -2.075149 -1.172715 4.805358
C -0.601460 -0.981319 3.247849
H 0.093781 -1.684116 3.701399
C -0.271059 -0.375974 2.039695
H 0.674830 -0.601446 1.546742
C -2.091767 1.485270 -1.128504
C -3.087544 0.506526 -1.089917
H -3.009069 -0.335904 -0.406503
C -4.191652 0.610101 -1.929261
H -4.964747 -0.152540 -1.885330
C -4.301935 1.673914 -2.816809
H -5.167329 1.748807 -3.472733
C -3.306153 2.644252 -2.864950
H -3.386560 3.479218 -3.558021
C -2.207282 2.554261 -2.020762
H -1.437216 3.321167 -2.055703
C -0.230227 3.088393 0.399747
C -0.708316 3.674949 1.571532
H -1.319268 3.100959 2.263410
C -0.391230 4.996123 1.869403
H -0.767087 5.446343 2.786088
C 0.406944 5.735772 1.004407
H 0.657139 6.767887 1.242551
C 0.895036 5.151666 -0.160541
H 1.533322 5.721187 -0.832887
C 0.583220 3.832681 -0.461643
H 0.985757 3.362630 -1.357402

Si 0.267712 -0.875802 -1.857785
N 1.567183 -2.848512 0.103408
C -0.139526 -3.794050 1.197151
C 1.204015 -3.642678 1.183221
H -0.747598 -4.394704 1.863723
H 1.945993 -4.097018 1.830345
B 0.373015 -2.432842 -0.589720
N -0.690555 -3.102707 0.125150
C -2.072106 -2.974127 -0.071303
C -2.934720 -2.808549 1.014097
C -2.596915 -2.998942 -1.366720
C -4.301249 -2.664382 0.805605
H -2.523456 -2.764503 2.018946
C -3.963480 -2.858962 -1.566313
H -1.921679 -3.139653 -2.207139
C -4.823184 -2.690854 -0.483831
H -4.960844 -2.524569 1.660135
H -4.359559 -2.883785 -2.579783
H -5.894348 -2.584578 -0.644239
C 2.907997 -2.515057 -0.177364
C 3.412794 -2.696043 -1.465435
C 3.739357 -2.016532 0.825322
C 4.740162 -2.393439 -1.739840
H 2.755160 -3.086920 -2.238147
C 5.066307 -1.718287 0.544135
H 3.331603 -1.843462 1.819676
C 5.573433 -1.912017 -0.736040
H 5.126894 -2.543080 -2.746193
H 5.702820 -1.313917 1.328452
H 6.612738 -1.674351 -0.953516

$^{F}Y_2Si$
E = -3899.52249077
P -2.770086 -1.091896 -0.094947
P 2.916165 -1.002642 0.041525
C -1.556993 -0.045501 -0.727702
C -1.637642 1.386351 -0.900332
C -2.582361 2.207539 -0.262267
C -2.569337 3.591394 -0.330762
C -1.582036 4.238901 -1.063121
C -0.634783 3.468909 -1.728022
C -0.693197 2.087751 -1.673204
C -3.013171 -1.091032 1.709854
C -4.132829 -0.512757 2.310439
H -4.931509 -0.104907 1.695651
C -4.218414 -0.435776 3.695715
H -5.092716 0.020244 4.155651
C -3.188139 -0.927882 4.489673
H -3.256878 -0.861293 5.573820
C -2.067005 -1.499808 3.896151
H -1.255325 -1.880378 4.513262
C -1.979277 -1.583281 2.513546
H -1.102670 -2.021252 2.037833
C -2.225665 -2.757108 -0.541897
C -2.409512 -3.869728 0.296395
H -2.834782 -3.739934 1.288915
C -2.043893 -5.134846 -0.134966
H -2.184785 -5.987224 0.527499
C -1.501619 -5.322116 -1.408712
H -1.211486 -6.317625 -1.738266

C -1.355607 -4.232646 -2.261350
H -0.959070 -4.374213 -3.264893
C -1.730440 -2.960751 -1.851835
H -1.660724 -2.118849 -2.536321
C -4.417127 -0.896948 -0.837984
C -4.613415 0.003506 -1.884299
H -3.791861 0.634176 -2.216336
C -5.856883 0.088121 -2.501769
H -6.008314 0.795582 -3.314480
C -6.901512 -0.727530 -2.082395
H -7.873715 -0.658501 -2.566693
C -6.704552 -1.636848 -1.046140
H -7.519244 -2.281305 -0.722054
C -5.465119 -1.724767 -0.426954
H -5.308692 -2.441600 0.378898
C 1.488443 -0.066342 0.156795
C 1.426929 1.346822 0.495745
C 0.439724 1.835603 1.367074
C 0.234410 3.189608 1.576587
C 1.063724 4.122505 0.965694
C 2.080203 3.682822 0.127665
C 2.231894 2.324265 -0.104427
C 4.151332 -0.555801 1.292822
C 4.215223 -1.222849 2.518612
H 3.559777 -2.068045 2.714983
C 5.124762 -0.814154 3.485703
H 5.173589 -1.341857 4.436039
C 5.970808 0.263505 3.239980
H 6.679791 0.583446 4.001133
C 5.913128 0.927006 2.019784
H 6.575488 1.767242 1.821619
C 5.011162 0.518701 1.043682
H 4.977139 1.031757 0.085730
C 2.368393 -2.710699 0.319345
C 2.735825 -3.778469 -0.498817
H 3.411785 -3.616174 -1.335198
C 2.217764 -5.046999 -0.258713
H 2.501249 -5.875612 -0.904023
C 1.333904 -5.254281 0.798536
H 0.922480 -6.246480 0.973980
C 0.959709 -4.196141 1.615905
H 0.256951 -4.355289 2.431755
C 1.461600 -2.920314 1.374731
H 1.176062 -2.077730 2.001650
C 3.821278 -1.017269 -1.536166
C 5.113591 -1.542148 -1.616219
H 5.608328 -1.914636 -0.719692
C 5.768714 -1.581897 -2.841837
H 6.776499 -1.987634 -2.905745
C 5.136688 -1.096110 -3.982810
H 5.652242 -1.124412 -4.941034
C 3.853790 -0.562830 -3.900193
H 3.366741 -0.169787 -4.790132
C 3.193911 -0.519521 -2.677621
H 2.201222 -0.080034 -2.589789
Si 0.030156 -0.955700 -0.529548
F 3.161299 1.952431 -0.987181
F 2.862011 4.559639 -0.481277
F 0.856728 5.415731 1.149060
F -0.759121 3.602604 2.349590

F -0.381231 0.990910 1.980636
F 0.249656 1.409454 -2.333558
F 0.338930 4.062464 -2.404262
F -1.521913 5.561485 -1.103235
F -3.483470 4.297180 0.315841
F -3.531691 1.634908 0.485691

^{F}YSiCl
E = -2555.21180512
P 1.052560 0.303420 0.170029
C 0.121269 -0.491460 -1.045135
C -1.342800 -0.457941 -0.889392
C -2.136291 0.571856 -1.394363
C -3.501429 0.639191 -1.151785
C -4.104779 -0.334684 -0.364949
C -3.343763 -1.370139 0.164568
C -1.983656 -1.420685 -0.105585
C 1.599435 1.999973 -0.190369
C 2.068440 2.821356 0.840621
H 2.110475 2.444808 1.862302
C 2.465163 4.122693 0.563531
H 2.829345 4.761192 1.365889
C 2.387342 4.610628 -0.738836
H 2.693849 5.632549 -0.953842
C 1.907300 3.800041 -1.761186
H 1.832954 4.185879 -2.775681
C 1.509773 2.494984 -1.489517
H 1.108595 1.859473 -2.277210
C 2.508694 -0.681065 0.590282
C 3.760093 -0.117679 0.848536
H 3.905205 0.957264 0.775631
C 4.832517 -0.939686 1.172704
H 5.809709 -0.500265 1.362178
C 4.661413 -2.318531 1.241460
H 5.506448 -2.958449 1.488092
C 3.415549 -2.881768 0.982091
H 3.282684 -3.960770 1.017832
C 2.339064 -2.069212 0.654353
H 1.368652 -2.501677 0.415259
C -0.017081 0.486407 1.626561
C -0.946505 1.532609 1.617301
H -0.890801 2.301283 0.847253
C -1.953913 1.576306 2.571123
H -2.679693 2.386774 2.555617
C -2.036529 0.579444 3.539700
H -2.830007 0.609208 4.283750
C -1.106240 -0.454337 3.558939
H -1.169476 -1.231349 4.317749
C -0.098630 -0.507514 2.601742
H 0.613250 -1.330185 2.604543
Si 1.034313 -1.097107 -2.489729
Cl 1.635606 -3.121650 -2.529672
F -1.275157 -2.396136 0.450104
F -3.919021 -2.287350 0.925281
F -5.402238 -0.275811 -0.120303
F -4.227432 1.634429 -1.637088
F -1.575253 1.555512 -2.088388

^{F}YSiHMDS
E = -2969.01618544

P -1.889361 -0.340779 -0.165830
C -0.298292 0.111727 -0.631877
C 0.323969 1.407810 -0.470894
C -0.081085 2.379181 0.459194
C 0.576039 3.583687 0.648514
C 1.703099 3.885810 -0.105835
C 2.143162 2.957896 -1.042287
C 1.450255 1.774255 -1.231923
C -2.125141 -0.869436 1.558791
C -2.598152 0.006626 2.540339
H -3.001695 0.975322 2.256843
C -2.523644 -0.342722 3.882590
H -2.892705 0.346995 4.638788
C -1.964779 -1.561319 4.259888
H -1.897933 -1.826435 5.313341
C -1.496539 -2.440389 3.290027
H -1.060748 -3.394562 3.579613
C -1.580579 -2.103587 1.943618
H -1.209561 -2.790291 1.184453
C -2.342285 -1.782430 -1.177042
C -3.247029 -2.742804 -0.712847
H -3.653274 -2.668655 0.293864
C -3.621288 -3.799185 -1.533097
H -4.319599 -4.547504 -1.163604
C -3.103765 -3.902356 -2.821552
H -3.394987 -4.734650 -3.459406
C -2.221660 -2.937263 -3.295359
H -1.823954 -3.008525 -4.305641
C -1.845621 -1.873994 -2.483359
H -1.166171 -1.108237 -2.851367
C -3.156751 0.902750 -0.537203
C -2.854845 1.962736 -1.392983
H -1.849563 2.063492 -1.795083
C -3.841982 2.880381 -1.733763
H -3.601228 3.709524 -2.395855
C -5.131418 2.738198 -1.233066
H -5.901840 3.458513 -1.501319
C -5.439379 1.671737 -0.393597
H -6.449895 1.553513 -0.007792
C -4.456421 0.753651 -0.047207
H -4.699688 -0.082382 0.607080
Si 0.796343 -1.379202 -0.721461
N 2.396917 -1.214015 -0.028030
Si 3.814318 -1.485218 -1.065695
Si 2.501524 -1.192354 1.748573
C 3.287574 -1.860399 -2.825219
H 2.674203 -2.768529 -2.879574
H 2.721377 -1.039102 -3.276221
H 4.190418 -2.026569 -3.430163
C 4.934681 0.017487 -1.066910
H 5.370927 0.216453 -0.082552
H 5.761850 -0.139968 -1.772135
H 4.387193 0.911004 -1.388095
C 4.744822 -3.001392 -0.451937
H 5.610191 -3.206491 -1.095951
H 5.113209 -2.901555 0.575332
H 4.086196 -3.879340 -0.485493
C 4.201473 -0.621206 2.314096
H 4.232044 -0.682827 3.410423
H 5.036547 -1.213921 1.926169

H 4.368290 0.427521 2.039300
C 2.185400 -2.929929 2.384134
H 2.143367 -2.960859 3.480646
H 1.233462 -3.309599 1.992821
H 2.980254 -3.609588 2.050413
C 1.300570 0.036287 2.493234
H 0.281531 -0.040204 2.104905
H 1.256443 -0.115672 3.580395
H 1.655588 1.058826 2.311585
F 1.901009 0.953572 -2.180597
F -1.121386 2.116852 1.256659
F 0.141168 4.442768 1.558925
F 2.343878 5.033125 0.063554
F 3.211865 3.223470 -1.781951

$^{F}YSiC_6F_5$
E = -2823.55664926
P -0.491681 1.348149 0.238096
C -0.605488 -0.026782 -0.797199
C -1.688648 -1.004068 -0.700740
C -2.587067 -1.218050 -1.749964
C -3.595930 -2.168886 -1.680137
C -3.739657 -2.935025 -0.529786
C -2.867602 -2.747420 0.535703
C -1.857451 -1.802792 0.434537
C -0.885735 2.928559 -0.568531
C -0.824371 4.117306 0.164878
H -0.506158 4.100520 1.207127
C -1.172682 5.318945 -0.436817
H -1.119828 6.245395 0.131428
C -1.595120 5.335259 -1.764322
H -1.869849 6.278023 -2.233538
C -1.674923 4.150697 -2.487449
H -2.015308 4.162227 -3.520706
C -1.320997 2.943945 -1.892393
H -1.392750 2.007010 -2.443340
C 1.183554 1.474596 0.914543
C 2.062717 2.502716 0.574044
H 1.728780 3.321153 -0.059258
C 3.381429 2.459810 1.015913
H 4.070150 3.252121 0.731362
C 3.822265 1.399984 1.800035
H 4.857751 1.364305 2.132827
C 2.948261 0.367737 2.134579
H 3.298185 -0.474513 2.728455
C 1.636374 0.394218 1.683542
H 0.964504 -0.438377 1.889259
C -1.682944 1.261346 1.604235
C -3.033917 1.183781 1.248790
H -3.322187 1.215913 0.198517
C -4.005886 1.070295 2.232583
H -5.054935 1.005852 1.951583
C -3.636516 1.040662 3.574582
H -4.398735 0.949549 4.345820
C -2.295962 1.131954 3.930400
H -2.007111 1.115850 4.979219
C -1.316053 1.239883 2.949125
H -0.269383 1.309151 3.234877
C 2.294539 -0.830770 -1.297706
C 4.655053 -0.725325 -0.711628

C 3.301532 -2.530974 0.127776
C 4.532401 -1.890331 0.035276
Si 0.742773 -0.035279 -2.026299
C 2.208916 -1.998146 -0.539859
C 3.543464 -0.220246 -1.370836
F 3.691118 0.918390 -2.040951
F 1.045692 -2.631135 -0.417102
F 3.188568 -3.632296 0.854642
F 5.579427 -2.374612 0.681978
F 5.815765 -0.088004 -0.748610
F -1.032217 -1.665694 1.469362
F -2.998070 -3.479979 1.629877
F -4.702745 -3.837589 -0.449244
F -4.432966 -2.337396 -2.691520
F -2.497690 -0.480549 -2.852425

^{F}YSiPy
E = -2342.75509713
P -1.610152 -0.095516 0.018299
C -0.027461 -0.740261 -0.228301
C 1.112733 0.152576 0.036360
C 1.955641 -0.035609 1.132374
C 3.146208 0.661365 1.279480
C 3.500051 1.615976 0.334340
C 2.671442 1.849115 -0.756417
C 1.500246 1.119757 -0.893211
C -2.291213 -0.386405 1.681558
C -3.382184 0.358476 2.142089
H -3.816519 1.134609 1.512818
C -3.903403 0.114054 3.405261
H -4.752505 0.692954 3.763126
C -3.333137 -0.865568 4.215328
H -3.739171 -1.051734 5.207845
C -2.240947 -1.597040 3.763592
H -1.788067 -2.353341 4.401307
C -1.716681 -1.359361 2.496724
H -0.848851 -1.909778 2.137283
C -2.781037 -0.780442 -1.177485
C -4.033906 -1.269153 -0.810558
H -4.334045 -1.278829 0.234613
C -4.893780 -1.764936 -1.785230
H -5.866370 -2.157769 -1.496298
C -4.508368 -1.766992 -3.120592
H -5.182986 -2.158403 -3.879642
C -3.257354 -1.278429 -3.488742
H -2.951100 -1.288256 -4.532594
C -2.389429 -0.792052 -2.521489
H -1.399353 -0.428412 -2.793719
C -1.641970 1.714229 -0.131467
C -1.013863 2.458307 0.872797
H -0.590780 1.952929 1.740169
C -0.928314 3.839601 0.764190
H -0.433323 4.413544 1.544761
C -1.474651 4.485587 -0.341111
H -1.406890 5.568381 -0.425833
C -2.108132 3.749122 -1.335470
H -2.538896 4.253214 -2.198042
C -2.190084 2.364651 -1.236039
H -2.680385 1.793179 -2.020619
C 2.085369 -2.457620 -0.735367

C 4.229171 -2.938553 0.249234
C 3.981112 -1.564395 -1.685392
C 4.811790 -2.139183 -0.726961
F 0.752270 1.332906 -1.969474
F 3.016423 2.753548 -1.661352
F 4.625043 2.299947 0.471076
F 3.936747 0.437281 2.318636
F 1.647127 -0.942861 2.054999
Si 0.155268 -2.459498 -0.793828
N 2.662320 -1.710927 -1.691586
H 4.841854 -3.414681 1.013667
H 5.885486 -1.966207 -0.752671
H 4.406576 -0.946424 -2.480169
C 2.851402 -3.114782 0.232690
H 2.363614 -3.732376 0.986132

^{F}YSiPyr
E = -2358.76111520
P -0.278570 1.188221 0.170947
C -0.238047 -0.312341 -0.665849
C 1.031134 -1.057301 -0.570022
C 1.268692 -1.985659 0.445238
C 2.497284 -2.616062 0.595553
C 3.533493 -2.317094 -0.279789
C 3.335716 -1.393806 -1.300099
C 2.098137 -0.780702 -1.429136
C -1.104391 1.161625 1.788564
C -0.935954 2.210867 2.699716
H -0.307469 3.063402 2.442538
C -1.559008 2.156061 3.938797
H -1.427921 2.969834 4.649384
C -2.343765 1.053638 4.272689
H -2.828526 1.010103 5.246378
C -2.497364 0.006776 3.371736
H -3.099854 -0.859516 3.637843
C -1.875449 0.050517 2.126740
H -1.976275 -0.783522 1.429736
C -1.066926 2.480790 -0.827598
C -1.971551 3.400254 -0.296459
H -2.255524 3.347769 0.751613
C -2.530945 4.374005 -1.117004
H -3.244601 5.082795 -0.702063
C -2.188031 4.435449 -2.462836
H -2.630534 5.197165 -3.101944
C -1.288835 3.516738 -2.996961
H -1.028097 3.555615 -4.052488
C -0.733995 2.535321 -2.186763
H -0.048486 1.794829 -2.597802
C 1.429380 1.677353 0.545902
C 2.060296 1.056794 1.629680
H 1.478981 0.440019 2.314312
C 3.428558 1.204713 1.815336
H 3.917280 0.711174 2.652867
C 4.171142 1.976065 0.925724
H 5.244441 2.086403 1.067264
C 3.543375 2.607818 -0.142541
H 4.122856 3.214963 -0.834741
C 2.175070 2.457180 -0.337726
H 1.692088 2.936363 -1.186223
C -2.899119 -2.035266 -1.156532

C -4.713336 -3.304596 -1.695683
C -3.812592 -3.328043 0.485218
C -4.772563 -3.809482 -0.397719
F 1.953112 0.131093 -2.385206
F 4.329623 -1.100187 -2.124124
F 4.707601 -2.909723 -0.142789
F 2.697575 -3.481112 1.578604
F 0.325846 -2.241999 1.340216
Si -1.588976 -0.834601 -1.759485
N -2.888859 -2.451767 0.121323
N -3.796130 -2.434758 -2.078499
H -5.438197 -3.620650 -2.450515
H -5.526240 -4.530671 -0.092675
H -3.791022 -3.661639 1.525963

^{F}YSiB2
E = -2501.72102022
P -0.845216 1.263072 0.189689
C -0.271333 -0.003960 -0.820460
C -1.018244 -1.272302 -0.750630
C -2.116734 -1.550594 -1.564448
C -2.872364 -2.706139 -1.418508
C -2.541895 -3.617934 -0.423544
C -1.458128 -3.371967 0.410957
C -0.711459 -2.215713 0.233979
C -1.896500 2.521836 -0.599601
C -2.630806 3.424363 0.177177
H -2.574298 3.380618 1.264675
C -3.441964 4.367485 -0.439278
H -4.014131 5.069166 0.164439
C -3.527812 4.408149 -1.829336
H -4.166658 5.145992 -2.311056
C -2.810153 3.501817 -2.601098
H -2.887990 3.525437 -3.686026
C -1.996164 2.553604 -1.989001
H -1.448187 1.821331 -2.580237
C 0.539713 2.128936 0.972112
C 0.632032 3.518328 1.061367
H -0.153186 4.149575 0.652685
C 1.749604 4.097779 1.651890
H 1.826163 5.181512 1.711604
C 2.772662 3.297501 2.149608
H 3.649433 3.757083 2.601935
C 2.684189 1.911340 2.057543
H 3.490751 1.282604 2.429687
C 1.572830 1.324660 1.467234
H 1.508395 0.243413 1.357710
C -1.908622 0.520542 1.457577
C -3.217835 0.189464 1.091069
H -3.615789 0.528413 0.135105
C -3.998357 -0.591754 1.932783
H -5.012300 -0.856080 1.639673
C -3.479319 -1.039777 3.144209
H -4.088768 -1.657169 3.801120
C -2.183644 -0.698400 3.517536
H -1.779566 -1.045332 4.466138
C -1.394078 0.076454 2.675268
H -0.373565 0.321027 2.960236
Si 1.099137 0.316027 -1.982243
C 4.551106 -0.982748 -0.127308

C 5.095627 -0.434154 -1.286813
C 5.340781 -1.549312 0.854288
C 6.457220 -0.428353 -1.519187
C 6.719560 -1.546600 0.628597
H 4.901005 -1.978269 1.750903
C 7.264915 -0.998785 -0.532268
H 6.869492 0.001456 -2.428068
H 7.380687 -1.983918 1.373781
H 8.343406 -1.016335 -0.674201
B 2.908634 -0.195036 -1.366860
O 4.090589 0.050817 -2.061993
O 3.199501 -0.847410 -0.167094
F 0.293969 -1.991815 1.071368
F -2.502029 -0.663356 -2.474970
F -3.918425 -2.934349 -2.198224
F -1.154097 -4.236413 1.366273
F -3.259131 -4.718028 -0.269707

^{F}YSiB1
E = -2770.90102294
P -1.558349 1.435406 0.034983
C -0.304383 0.298951 -0.211624
C -0.515583 -1.023830 -0.791685
C 0.315971 -1.535232 -1.801802
C 0.205824 -2.832031 -2.283169
C -0.780164 -3.675457 -1.789589
C -1.634255 -3.207187 -0.801915
C -1.485246 -1.920268 -0.314074
C -1.410738 2.950401 -0.976532
C -2.443623 3.894071 -0.952636
H -3.313857 3.733176 -0.316373
C -2.368208 5.029120 -1.747513
H -3.173040 5.761264 -1.725792
C -1.267223 5.223063 -2.579157
H -1.209439 6.112239 -3.204197
C -0.251063 4.276347 -2.621812
H 0.602575 4.418286 -3.281370
C -0.322437 3.135683 -1.826285
H 0.459700 2.378305 -1.869180
C -1.635078 1.990944 1.765638
C -1.563142 3.324835 2.164892
H -1.476710 4.116473 1.425246
C -1.575333 3.644351 3.519501
H -1.508118 4.685852 3.827407
C -1.662321 2.639054 4.474786
H -1.669699 2.893391 5.532966
C -1.726838 1.304970 4.078898
H -1.783505 0.515060 4.825563
C -1.703573 0.978944 2.730366
H -1.722437 -0.060179 2.406521
C -3.190763 0.820423 -0.451967
C -3.333867 0.364368 -1.765583
H -2.487925 0.406892 -2.450499
C -4.553665 -0.142596 -2.192305
H -4.661327 -0.504448 -3.212720
C -5.634893 -0.185258 -1.316646
H -6.590674 -0.582263 -1.652962
C -5.495903 0.280363 -0.013925
H -6.341517 0.249128 0.670096
C -4.274172 0.779665 0.423796

H -4.162978 1.128580 1.448243
Si 1.387982 0.854300 0.253681
N 2.632347 -1.850094 1.066884
C 4.810661 -1.513961 0.654490
C 3.886724 -2.443930 0.976799
H 5.883770 -1.636650 0.569012
H 4.037776 -3.490554 1.213823
B 2.774771 -0.447854 0.735051
N 4.198952 -0.275432 0.514149
C 4.878434 0.878148 0.105798
C 5.967678 0.805689 -0.767964
C 4.456589 2.131475 0.560902
C 6.616466 1.963661 -1.175012
H 6.285831 -0.160960 -1.151384
C 5.100983 3.285890 0.134956
H 3.641485 2.187063 1.280144
C 6.185652 3.210533 -0.731586
H 7.459766 1.889981 -1.859360
H 4.758757 4.252686 0.499711
H 6.693929 4.115493 -1.057626
C 1.495264 -2.568949 1.470645
C 0.582387 -1.994784 2.360084
C 1.281752 -3.873633 1.019063
C -0.517139 -2.723096 2.791392
H 0.757550 -0.980088 2.713425
C 0.176741 -4.593966 1.456384
H 1.975467 -4.311912 0.304412
C -0.730284 -4.023135 2.342303
H -1.220207 -2.268168 3.487400
H 0.010849 -5.601467 1.079118
H -1.601945 -4.583392 2.672075
F 1.262380 -0.767136 -2.332801
F 1.026649 -3.263478 -3.230221
F -0.887296 -4.919952 -2.233830
F -2.544031 -4.017585 -0.280024
F -2.288915 -1.560621 0.687993

l
E = -1307.79348567
C 1.679103 2.157942 -0.085798
C -0.498359 1.022336 -0.209483
C -1.109048 2.278548 -0.402812
C -0.349505 3.430780 -0.388525
C 1.046809 3.373396 -0.214831
H 2.764159 2.129993 0.007495
H -2.181119 2.330915 -0.569449
H -0.834188 4.394554 -0.532272
H 1.628748 4.292955 -0.208411
N -1.126706 -0.179328 -0.186852
Si 0.050762 -1.613376 -0.630468
C -2.495107 -0.356545 -0.022333
C -3.251766 0.435157 0.857688
C -3.134027 -1.419116 -0.680259
C -4.600726 0.180176 1.051214
H -2.761996 1.229727 1.415426
C -4.484591 -1.668158 -0.474999
H -2.555598 -2.030407 -1.371138
C -5.230267 -0.869190 0.384455
H -5.164882 0.800005 1.746318
H -4.959563 -2.494437 -1.001294

H -6.289047 -1.064628 0.540762
C 0.944319 0.941599 -0.074564
C 1.422232 -0.378174 0.041247
P 3.067280 -0.813016 0.213301
C 4.193799 -0.332741 -1.138524
H 4.205611 0.757292 -1.240052
H 5.212665 -0.695972 -0.959706
H 3.805207 -0.760561 -2.069153
C 3.828514 -0.159991 1.728281
H 4.883914 -0.448361 1.792920
H 3.745732 0.931388 1.739151
H 3.279961 -0.556232 2.588734
C 3.178372 -2.611116 0.309808
H 4.206206 -2.929001 0.513729
H 2.504147 -2.960594 1.099401
H 2.838618 -3.038248 -0.640848

II
E = -1856.99498746
C 0.860224 2.788917 -0.037937
C 0.392366 1.509123 0.230635
C -0.797876 1.025093 -0.348876
C -1.471131 1.881080 -1.235296
C -1.006499 3.161718 -1.492975
C 0.161961 3.627655 -0.896691
H 1.784490 3.104629 0.441897
H -2.375190 1.520957 -1.720461
H -1.558191 3.797254 -2.183380
H 0.528699 4.630589 -1.102671
N -1.282256 -0.260543 -0.093912
S 1.347355 0.491796 1.358532
O 2.689428 1.110755 1.407611
O 0.574890 0.342550 2.583435
C 1.461658 -0.990324 0.481023
Si -0.195025 -1.666049 0.196929
C -2.662684 -0.513979 -0.066243
C -3.571066 0.399352 0.487100
C -3.142252 -1.749192 -0.516081
C -4.914874 0.074790 0.581706
H -3.206206 1.351909 0.863633
C -4.489059 -2.075188 -0.396871
H -2.447311 -2.441221 -0.995325
C -5.384712 -1.163841 0.145612
H -5.604479 0.792784 1.022107
H -4.838319 -3.043892 -0.749512
H -6.440345 -1.412145 0.230548
P 2.858093 -1.139181 -0.516240
C 3.122173 0.234444 -1.672361
H 4.010030 0.070111 -2.293224
H 2.232383 0.337346 -2.303343
H 3.241825 1.145567 -1.076610
C 2.698927 -2.630709 -1.519509
H 1.787886 -2.557817 -2.123769
H 3.575962 -2.740235 -2.167006
H 2.613136 -3.501018 -0.860861
C 4.383007 -1.300761 0.436339
H 4.452311 -0.417441 1.078725
H 4.309210 -2.195094 1.063433
H 5.255660 -1.370314 -0.222540

III
E = -2619.08235191
C -0.409926 1.526299 -0.568104
C 0.410013 1.526450 0.568026
C 0.793784 2.745029 1.129484
C 0.396921 3.951206 0.569265
C -0.397185 3.951055 -0.569733
C -0.793879 2.744732 -1.129751
H 1.430356 2.720205 2.010410
H 0.712436 4.887827 1.024629
H -0.712834 4.887556 -1.025252
H -1.430460 2.719682 -2.010668
S -1.004479 0.086778 -1.527225
S 1.004689 0.087129 1.527349
O -2.232326 0.607082 -2.161383
O 0.132227 -0.283529 -2.384045
O -0.131948 -0.283042 2.384314
O 2.232615 0.607457 2.161335
C -1.402534 -1.169494 -0.457734
C 1.402578 -1.169309 0.457977
Si 0.000007 -2.247694 0.000401
P 2.861476 -0.958617 -0.436634
P -2.861563 -0.958553 0.436596
C 2.865072 -2.096330 -1.831049
H 3.791011 -1.975283 -2.404314
H 2.777833 -3.126394 -1.471837
H 1.990718 -1.849931 -2.445362
C 3.069800 0.698018 -1.137480
H 3.086021 1.424694 -0.318399
H 4.006746 0.755053 -1.703076
H 2.211143 0.887138 -1.792244
C 4.336717 -1.260752 0.564998
H 4.322953 -2.300188 0.908881
H 5.257825 -1.058707 0.006877
H 4.258467 -0.600345 1.435758
C -2.865435 -2.095900 1.831311
H -3.791553 -1.974817 2.404284
H -2.777980 -3.126042 1.472376
H -1.991278 -1.849261 2.445804
C -3.070024 0.698267 1.136950
H -3.086134 1.424712 0.317662
H -4.007060 0.755416 1.702383
H -2.211490 0.887613 1.791791
C -4.336649 -1.260934 -0.565203
H -5.257855 -1.058655 -0.007324
H -4.258195 -0.600804 -1.436151
H -4.322891 -2.300483 -0.908749

IV
E = -1827.06921715
C 0.819469 3.290608 0.459940
C 0.295292 2.011392 0.458815
C -0.949146 1.639081 -0.072263
C -1.721686 2.678054 -0.614956
C -1.239954 3.979129 -0.602478
C 0.017168 4.294470 -0.070596
H 1.806066 3.497086 0.869224
H -2.706953 2.469361 -1.026423
H -1.856172 4.778895 -1.009819
H 0.364314 5.325312 -0.070556

B -1.201762 0.099817 0.197097
S 1.142444 0.526237 0.856452
O 2.031186 0.528292 2.018682
O -0.212944 -0.300967 1.196390
C 1.693035 -0.104379 -0.573055
Si 0.170254 -0.361822 -1.763871
C -2.530242 -0.699722 0.155002
C -3.505279 -0.468234 -0.827947
C -2.797782 -1.683035 1.118909
C -4.698159 -1.177799 -0.842111
H -3.311292 0.263249 -1.612210
C -3.990432 -2.395885 1.109803
H -2.053201 -1.878885 1.889237
C -4.946430 -2.144108 0.130118
H -5.437736 -0.983351 -1.617289
H -4.178604 -3.151277 1.871413
H -5.881340 -2.701966 0.120621
P 2.899793 -1.311033 -0.369429
C 4.449675 -0.655901 0.292167
H 5.188542 -1.454035 0.424973
H 4.833634 0.100303 -0.400041
H 4.226473 -0.181129 1.253011
C 3.269536 -2.030062 -1.980506
H 3.600475 -1.235422 -2.657006
H 4.051137 -2.791492 -1.883820
H 2.353771 -2.478377 -2.382497
C 2.408012 -2.668479 0.726232
H 2.210965 -2.237941 1.715198
H 1.475693 -3.094206 0.338248
H 3.179094 -3.443371 0.801639

V
E = -1277.86649226
C 1.811710 2.084161 -0.115823
C -0.408332 1.061400 -0.121699
C -0.947210 2.355471 -0.251984
C -0.146729 3.486996 -0.287763
C 1.239187 3.344980 -0.209774
H 2.898802 2.011123 -0.082647
H -2.027135 2.466934 -0.335717
H -0.593712 4.474882 -0.380709
H 1.880808 4.224714 -0.235762
B -1.285468 -0.207943 -0.092137
Si 0.137927 -1.592689 -0.127917
C -2.816790 -0.403194 -0.008769
C -3.634300 0.475903 0.726161
C -3.454004 -1.485137 -0.642904
C -5.009165 0.292912 0.809707
H -3.176485 1.307161 1.260007
C -4.828007 -1.667167 -0.572239
H -2.847903 -2.193639 -1.207678
C -5.613418 -0.776060 0.155023
H -5.613954 0.986701 1.391858
H -5.291564 -2.510555 -1.081876
H -6.691001 -0.918895 0.216636
C 1.022654 0.922882 -0.076752
C 1.536251 -0.440247 -0.019990
P 3.182483 -0.855859 0.111181
C 4.238714 -0.347660 -1.282688
H 4.177729 0.737124 -1.414077

H 5.282924 -0.639036 -1.120866
H 3.855052 -0.823116 -2.191519
C 4.013763 -0.203382 1.591761
H 5.076195 -0.471771 1.606573
H 3.910120 0.885926 1.617404
H 3.511539 -0.614185 2.473790
C 3.365945 -2.651573 0.206601
H 4.424393 -2.919113 0.292855
H 2.817898 -3.024442 1.078105
H 2.943797 -3.106258 -0.695775

4.2.3 H_2 activation transition states

Pathway A

$^{Tos}Y_2Si$
Not observed.

$^{Tos}YSiCl$
E = -2648.09474676
S 0.739044 1.934663 -0.831398
P -0.911119 -0.435098 0.052049
O 0.709102 3.327485 -0.307170
O 0.559972 1.689315 -2.263056
C -0.438153 1.200108 0.165345
C -1.019857 -1.149713 1.718441
C -1.985072 -2.091644 2.075213
H -2.735426 -2.408797 1.355133
C -2.004636 -2.607662 3.366229
H -2.765907 -3.333875 3.643726
C -1.063031 -2.192159 4.301372
H -1.084682 -2.596113 5.311626
C -0.098938 -1.252380 3.947816
H 0.634659 -0.919221 4.679097
C -0.076980 -0.727876 2.662250
H 0.660984 0.021971 2.380571
C 0.330297 -1.405234 -0.848374
C 1.320552 -2.119153 -0.170292
H 1.327356 -2.151096 0.916612
C 2.301093 -2.792679 -0.887715
H 3.072003 -3.346861 -0.356610
C 2.297095 -2.754931 -2.277984
H 3.065291 -3.284975 -2.838050
C 1.315940 -2.037023 -2.954686
H 1.317280 -1.998337 -4.041956
C 0.332915 -1.358863 -2.245481
H -0.419121 -0.776214 -2.772417
C -2.480310 -0.750989 -0.792717
C -2.801803 -2.037652 -1.236951
H -2.100920 -2.858732 -1.089493
C -4.008726 -2.262484 -1.885844
H -4.257911 -3.261920 -2.236691
C -4.891524 -1.205974 -2.095615
H -5.835220 -1.382681 -2.608291
C -4.564934 0.075005 -1.664301
H -5.248072 0.903444 -1.839000
C -3.357730 0.309667 -1.014972
H -3.089513 1.315078 -0.695115
C 2.339356 1.270776 -0.410674
C 2.785688 1.353146 0.906232
H 2.178734 1.870424 1.648955

C 3.997419 0.774705 1.244760
H 4.351025 0.832427 2.273635
C 4.781920 0.122561 0.282965
C 4.318735 0.078518 -1.030887
H 4.917396 -0.419895 -1.792222
C 3.100792 0.649866 -1.386137
H 2.722606 0.610074 -2.404709
C 6.091306 -0.498021 0.667313
H 5.965733 -1.217989 1.485948
H 6.801499 0.262656 1.016714
H 6.550458 -1.021453 -0.177466
Si -1.052729 2.551831 1.229293
H -2.141100 1.341913 1.875343
H -1.521918 2.026464 2.576830
Cl -2.650941 3.645062 0.408783

^{Tos}YSiHMDS
Not observed.

$^{Tos}YSiC_6F_5$
E = -2916.43414688
S 1.599836 -1.782071 -1.023782
P 0.312664 0.417531 0.459876
O 1.372460 -2.561367 -2.242283
O 1.543477 -2.487153 0.272564
C 0.507612 -0.441506 -1.037297
C -0.639912 1.928940 0.154337
C -1.803709 2.232041 0.860926
H -2.189576 1.539822 1.604781
C -2.490254 3.413900 0.597185
H -3.409019 3.632625 1.136549
C -2.008911 4.303482 -0.354754
H -2.545897 5.228411 -0.555048
C -0.851745 4.003342 -1.068021
H -0.482664 4.689861 -1.826933
C -0.178029 2.814702 -0.826963
H 0.711278 2.560575 -1.402326
C 1.891523 0.936179 1.209349
C 2.383421 2.238919 1.097652
H 1.780027 3.022089 0.648528
C 3.652576 2.547679 1.572498
H 4.025800 3.565975 1.483865
C 4.439638 1.561515 2.156064
H 5.438651 1.802372 2.515309
C 3.943154 0.269462 2.290683
H 4.551833 -0.505933 2.750857
C 2.670270 -0.044849 1.833390
H 2.292349 -1.061447 1.918922
C -0.535152 -0.481260 1.785549
C -0.640913 0.085914 3.059872
H -0.163059 1.041459 3.274802
C -1.359266 -0.572029 4.048825
H -1.445873 -0.131297 5.039913
C -1.967166 -1.795295 3.771057
H -2.534281 -2.305908 4.547087
C -1.836780 -2.372005 2.513470
H -2.296120 -3.333755 2.295976
C -1.111996 -1.719948 1.521464
H -0.953708 -2.185817 0.555103
C 3.267665 -1.132935 -1.075941

C 3.552897 0.143154 -1.540674
H 2.747462 0.765520 -1.925339
C 4.853127 0.622575 -1.461655
H 5.071857 1.633558 -1.803312
C 5.882756 -0.163144 -0.938476
C 5.576213 -1.458371 -0.513955
H 6.367403 -2.090738 -0.112999
C 4.277358 -1.943744 -0.569637
H 4.029086 -2.933830 -0.194171
C 7.268063 0.390412 -0.796354
H 7.544353 1.012155 -1.655541
H 8.012717 -0.405914 -0.694587
H 7.338906 1.025340 0.098442
Si -0.700410 -0.531538 -2.410172
H -0.815760 1.124261 -2.597410
H -0.590163 0.527319 -3.500763
C -2.460206 -0.447875 -1.689741
C -2.930708 -1.620546 -1.104984
C -3.278749 0.669198 -1.549945
C -4.080728 -1.677556 -0.331976
C -4.444067 0.650520 -0.795290
C -4.849732 -0.529991 -0.185627
F -2.941796 1.833805 -2.091868
F -2.216567 -2.739081 -1.224694
F -5.161632 1.753907 -0.638916
F -5.953766 -0.557124 0.538653
F -4.446705 -2.802854 0.260673

^{Tos}*YSiPy*
E = -2435.58864556
S 0.341335 -1.875138 0.689703
P -0.143205 0.992855 -0.017519
O -0.249559 -3.113764 0.121206
O 0.252914 -1.642993 2.136720
C -0.405752 -0.671959 -0.247567
C -0.000781 1.790565 -1.644231
C -0.503430 3.065128 -1.911131
H -1.027500 3.621105 -1.137484
C -0.353347 3.617605 -3.177739
H -0.753971 4.608020 -3.383917
C 0.297077 2.905411 -4.180219
H 0.407835 3.340169 -5.171699
C 0.796171 1.633230 -3.918544
H 1.296472 1.069205 -4.702989
C 0.646088 1.072863 -2.656232
H 1.015999 0.070617 -2.445437
C 1.409150 1.304909 0.867182
C 2.595949 1.551926 0.174147
H 2.592627 1.621996 -0.911188
C 3.784997 1.709837 0.874695
H 4.708473 1.900964 0.332290
C 3.793294 1.620842 2.262862
H 4.725985 1.747214 2.809546
C 2.613562 1.363156 2.953486
H 2.620776 1.281112 4.038373
C 1.420397 1.200609 2.260788
H 0.502020 0.970646 2.796045
C -1.413465 1.900245 0.905963
C -1.148831 3.162438 1.445936
H -0.161758 3.609689 1.333390

C -2.142652 3.837334 2.143839
H -1.935719 4.817060 2.569835
C -3.397028 3.254909 2.305482
H -4.172878 3.783959 2.855774
C -3.656877 1.995252 1.775938
H -4.632470 1.532588 1.909723
C -2.666086 1.313949 1.078225
H -2.852993 0.321258 0.675647
C 2.081599 -1.918323 0.302515
C 2.471007 -2.072036 -1.026303
H 1.713305 -2.219952 -1.795647
C 3.818140 -2.034636 -1.343809
H 4.128522 -2.150662 -2.381703
C 4.791114 -1.857514 -0.350020
C 4.371159 -1.730428 0.972600
H 5.113574 -1.595526 1.758225
C 3.020467 -1.759389 1.307465
H 2.680041 -1.644128 2.333584
C 6.245323 -1.815374 -0.712080
H 6.872401 -1.634152 0.166825
H 6.450288 -1.023722 -1.444191
H 6.567981 -2.761363 -1.165829
Si -1.531204 -1.549410 -1.437148
H -2.184378 -0.028847 -1.863038
H -1.796586 -0.646668 -2.662064
C -3.234094 -1.913432 -0.691951
C -5.469650 -2.254134 -1.049553
C -4.625636 -2.527465 1.166349
C -5.705156 -2.547579 0.291852
H -6.294932 -2.269142 -1.765658
H -4.759027 -2.761088 2.221763
H -6.708469 -2.793411 0.633393
N -4.273317 -1.951492 -1.534433
C -3.367908 -2.209147 0.670264
H -2.494466 -2.184071 1.320408

^{Tos}YSiPyr
E = -2451.64781124
S -1.344536 -0.478967 -1.756435
P 0.389182 0.665320 0.256182
O -1.116368 -1.417605 -2.860225
O -1.390667 0.970151 -2.024521
C -0.160405 -0.768512 -0.533254
C 1.404848 0.180267 1.682423
C 2.689918 0.684235 1.883609
H 3.117677 1.384346 1.171547
C 3.441170 0.267610 2.977153
H 4.445417 0.661239 3.119752
C 2.916802 -0.652800 3.877213
H 3.507457 -0.977335 4.731737
C 1.641902 -1.170448 3.675541
H 1.233425 -1.906098 4.365171
C 0.894099 -0.766498 2.577087
H -0.083836 -1.207045 2.394817
C -0.982353 1.692190 0.895248
C -1.702883 1.283618 2.018934
H -1.412110 0.393318 2.567576
C -2.809389 2.006667 2.446724
H -3.363287 1.674202 3.322118
C -3.208702 3.144189 1.753900

H -4.077615 3.709037 2.086444
C -2.497494 3.554484 0.630865
H -2.811898 4.435822 0.075671
C -1.392315 2.832171 0.198000
H -0.871915 3.127047 -0.708207
C 1.372890 1.810085 -0.751719
C 1.794187 3.035910 -0.229737
H 1.522056 3.320092 0.786706
C 2.551475 3.899727 -1.011621
H 2.880614 4.853457 -0.603978
C 2.877499 3.544360 -2.317594
H 3.465915 4.222766 -2.932804
C 2.442717 2.331357 -2.841935
H 2.684869 2.060644 -3.867418
C 1.689813 1.462008 -2.062247
H 1.337992 0.514330 -2.461774
C -2.930668 -0.908097 -1.060123
C -3.317636 -2.245174 -1.039879
H -2.672862 -2.994981 -1.494927
C -4.522436 -2.591027 -0.448550
H -4.829305 -3.636306 -0.430478
C -5.356611 -1.619717 0.118370
C -4.949880 -0.286241 0.067050
H -5.589252 0.485698 0.493882
C -3.744490 0.078589 -0.523041
H -3.434051 1.119397 -0.572833
C -6.662168 -2.010427 0.743824
H -7.363886 -2.388389 -0.011509
H -7.137556 -1.160690 1.244573
H -6.527200 -2.809642 1.483187
Si 0.694423 -2.370355 -0.687604
H 0.814027 -2.723288 0.934628
H 0.301306 -3.467648 0.314536
C 2.572470 -2.242111 -0.533562
C 4.587954 -3.194573 -0.100621
C 4.429568 -0.947232 -0.795952
C 5.241527 -2.002106 -0.407570
H 5.154760 -4.076603 0.208128
H 4.850217 0.024871 -1.066700
H 6.323127 -1.909236 -0.353572
N 3.108497 -1.061249 -0.854007
N 3.273886 -3.323861 -0.169254

^{Tos}YSiB2
Not determined.

^{Tos}YSiB1
E = -2863.78984158
S -1.653428 1.531369 -0.205710
P -0.233959 -0.984682 0.354349
O -1.524396 2.645751 -1.155241
O -1.632750 1.812592 1.242768
C -0.466775 0.384457 -0.640304
C 0.238442 -2.370771 -0.722686
C 1.309721 -3.226868 -0.475670
H 1.957274 -3.073246 0.382232
C 1.589529 -4.261534 -1.363318
H 2.434127 -4.917458 -1.165830
C 0.814436 -4.441579 -2.501293
H 1.039766 -5.251125 -3.192889

C -0.238721 -3.569700 -2.767780
H -0.834661 -3.689914 -3.670285
C -0.523034 -2.535343 -1.888162
H -1.320274 -1.824752 -2.105251
C -1.746150 -1.504724 1.231055
C -2.662719 -2.384560 0.650232
H -2.465754 -2.823055 -0.323723
C -3.834434 -2.711478 1.319894
H -4.542984 -3.397222 0.859740
C -4.099159 -2.164911 2.571149
H -5.017430 -2.423741 3.095142
C -3.192626 -1.283500 3.149436
H -3.399630 -0.846252 4.124059
C -2.019835 -0.949270 2.483963
H -1.325441 -0.238902 2.924939
C 0.979479 -0.809464 1.695275
C 1.321851 -1.878138 2.531414
H 0.902468 -2.867739 2.357842
C 2.175076 -1.670032 3.606062
H 2.444381 -2.502410 4.253321
C 2.668249 -0.392380 3.866775
H 3.331132 -0.230168 4.714819
C 2.297941 0.676473 3.059365
H 2.665329 1.678625 3.267684
C 1.449912 0.471207 1.974551
H 1.118832 1.301684 1.354867
C -3.250750 0.783972 -0.512010
C -3.501232 0.197100 -1.748674
H -2.731580 0.223404 -2.519229
C -4.723840 -0.415715 -1.973789
H -4.923001 -0.880146 -2.939165
C -5.712192 -0.442485 -0.981472
C -5.443202 0.179597 0.237112
H -6.202595 0.175095 1.018311
C -4.218070 0.791655 0.479552
H -3.988181 1.258995 1.434030
C -7.014205 -1.146965 -1.219238
H -7.787807 -0.806327 -0.522924
H -6.904141 -2.232195 -1.082906
H -7.378201 -0.986597 -2.240666
Si 0.732643 1.011256 -1.932531
H 1.487543 -0.485090 -1.943890
H 1.041279 -0.247416 -2.824396
N 3.613688 0.816721 -0.470409
C 3.594171 2.830732 0.507681
C 4.265798 1.660843 0.423537
H 3.844367 3.722589 1.071264
H 5.198424 1.375568 0.896147
N 2.495005 2.786059 -0.334283
C 4.005918 -0.519113 -0.635716
C 4.040665 -1.094434 -1.908621
C 4.386800 -1.284610 0.470170
C 4.462430 -2.407669 -2.070653
H 3.769529 -0.485956 -2.768792
C 4.822545 -2.592920 0.298568
H 4.317826 -0.848445 1.464673
C 4.867036 -3.159405 -0.971531
H 4.490386 -2.841300 -3.068486
H 5.121811 -3.175202 1.168394
H 5.215011 -4.181963 -1.104942

C 1.506007 3.800091 -0.367542
C 1.208360 4.439077 -1.569497
C 0.795592 4.123616 0.786519
C 0.200009 5.391862 -1.613679
H 1.768559 4.171533 -2.462514
C -0.210347 5.079283 0.735447
H 1.013017 3.602045 1.716505
C -0.509400 5.715450 -0.462740
H -0.037528 5.879596 -2.556880
H -0.779212 5.306292 1.634401
H -1.308432 6.452730 -0.504082
B 2.437937 1.497183 -0.975527

$^{Ph}Y_2Si$
E = -2906.90295520
P 2.753197 0.132709 0.215171
P -2.632125 0.377815 -0.066065
C 1.435329 -0.960559 0.191859
C 1.614990 -2.288482 -0.413812
C 2.871263 -2.910402 -0.567089
H 3.769420 -2.417667 -0.199893
C 3.004839 -4.150118 -1.180152
H 3.996648 -4.590249 -1.275617
C 1.891142 -4.831030 -1.658907
H 1.996270 -5.804843 -2.132817
C 0.638909 -4.239105 -1.517010
H -0.250713 -4.749642 -1.883625
C 0.503685 -2.996002 -0.915723
H -0.483436 -2.547732 -0.819635
C 4.137990 -0.395842 1.298069
C 5.480567 -0.075360 1.091481
H 5.781545 0.500088 0.219702
C 6.445968 -0.509796 1.993451
H 7.491990 -0.260777 1.825043
C 6.078927 -1.264688 3.102996
H 6.838191 -1.604895 3.804709
C 4.743320 -1.596830 3.306333
H 4.453828 -2.201272 4.163658
C 3.776775 -1.170308 2.403633
H 2.730480 -1.449124 2.531199
C 2.277175 1.764896 0.856472
C 2.633607 2.192477 2.133581
H 3.233588 1.552967 2.776823
C 2.197588 3.430136 2.595738
H 2.466621 3.755531 3.598670
C 1.419761 4.246083 1.782394
H 1.080428 5.214091 2.147097
C 1.086314 3.830418 0.494916
H 0.483785 4.465790 -0.150888
C 1.513934 2.595268 0.030526
H 1.247056 2.264168 -0.970543
C 3.496754 0.522758 -1.408351
C 3.186519 -0.248259 -2.529936
H 2.527891 -1.106539 -2.429478
C 3.705313 0.091516 -3.774718
H 3.464359 -0.521042 -4.641506
C 4.519425 1.210396 -3.914647
H 4.919408 1.475759 -4.891532
C 4.811432 1.998027 -2.805495
H 5.431515 2.886000 -2.912240

C 4.301968 1.657354 -1.558137
H 4.511602 2.292144 -0.698165
C -1.767135 -0.917361 0.646876
C -2.483904 -2.037757 1.265065
C -1.831283 -3.252959 1.564135
H -0.765357 -3.339337 1.365133
C -2.509150 -4.335541 2.100936
H -1.960725 -5.254983 2.302531
C -3.872882 -4.261531 2.381340
H -4.401682 -5.111954 2.806836
C -4.534298 -3.067132 2.125743
H -5.593473 -2.967379 2.360204
C -3.854406 -1.979128 1.591489
H -4.401833 -1.050012 1.444948
C -3.266815 1.675638 1.054127
C -2.321238 2.522454 1.649066
H -1.278488 2.470233 1.337205
C -2.705215 3.410841 2.644270
H -1.959434 4.063734 3.094533
C -4.031138 3.459460 3.065655
H -4.331278 4.155655 3.846623
C -4.971511 2.610905 2.491064
H -6.007791 2.639562 2.822305
C -4.592813 1.719972 1.492345
H -5.336524 1.056912 1.053088
C -1.567924 1.260061 -1.256943
C -0.714142 0.500711 -2.066122
H -0.609095 -0.567054 -1.884247
C -0.008388 1.109912 -3.096280
H 0.649441 0.513927 -3.723183
C -0.140757 2.476645 -3.325739
H 0.419482 2.949997 -4.129808
C -0.993732 3.233389 -2.528661
H -1.107644 4.300728 -2.709583
C -1.711697 2.627934 -1.502318
H -2.388669 3.223238 -0.893687
C -4.023243 -0.133159 -1.124456
C -4.927610 0.811331 -1.614978
H -4.855484 1.850504 -1.296509
C -5.915091 0.429900 -2.515290
H -6.618536 1.169450 -2.892761
C -5.997758 -0.893670 -2.936141
H -6.772129 -1.192845 -3.640125
C -5.086551 -1.832623 -2.463645
H -5.145559 -2.867020 -2.795936
C -4.096737 -1.456344 -1.563456
H -3.379348 -2.185107 -1.193219
Si -0.056716 -0.313970 1.051525
H 0.097779 -0.405990 2.615264
H -0.054609 -1.374269 2.398153

^{Ph}*YSiCl*
E = -2059.50135336
P -0.448909 0.191008 -0.019870
C 0.894660 -0.751236 -0.535028
C 2.257931 -0.246493 -0.301190
C 2.519431 1.020850 0.261636
H 1.699552 1.666998 0.568963
C 3.812718 1.492019 0.445939
H 3.958637 2.481740 0.876268

C 4.908842 0.716131 0.088888
H 5.922352 1.084209 0.233970
C 4.678701 -0.547894 -0.445085
H 5.518309 -1.186375 -0.715969
C 3.387855 -1.020104 -0.631871
H 3.244972 -2.024824 -1.021818
C -0.612136 0.418257 1.782909
C -1.787642 0.925194 2.347560
H -2.620011 1.215630 1.707531
C -1.911462 1.025932 3.727417
H -2.829541 1.416542 4.161906
C -0.867721 0.612863 4.550913
H -0.966259 0.690315 5.632048
C 0.291039 0.082926 3.994418
H 1.099596 -0.259499 4.636805
C 0.418511 -0.023850 2.613548
H 1.310251 -0.464827 2.173282
C -2.016254 -0.584630 -0.489505
C -2.513708 -1.625730 0.300615
H -1.986839 -1.919710 1.207131
C -3.674193 -2.287076 -0.076788
H -4.054085 -3.103009 0.534261
C -4.344597 -1.909216 -1.236467
H -5.253659 -2.429585 -1.531793
C -3.857666 -0.866257 -2.016533
H -4.384278 -0.568218 -2.920752
C -2.694151 -0.201680 -1.646033
H -2.310532 0.610656 -2.259517
C -0.455124 1.851358 -0.784688
C -0.996234 2.989258 -0.184574
H -1.415807 2.939099 0.816428
C -0.979863 4.204686 -0.859437
H -1.400797 5.089368 -0.385824
C -0.421115 4.292242 -2.130316
H -0.407004 5.246403 -2.653622
C 0.132994 3.163448 -2.725051
H 0.588015 3.231919 -3.711062
C 0.121879 1.947093 -2.053671
H 0.576807 1.060411 -2.493880
Si 0.442552 -2.360638 -1.252683
H 1.649282 -2.244389 -2.447970
H 0.609631 -2.543799 -2.755959
Cl 1.425479 -3.992576 -0.341203

^{Ph}YSiHMDS
E = -2473.30450431
P -1.643857 -0.168171 -0.157357
C -0.063522 0.477140 -0.319054
C 0.207391 1.831310 0.176772
C -0.662132 2.534832 1.038203
H -1.593260 2.077746 1.368646
C -0.375037 3.815904 1.490599
H -1.084295 4.315738 2.148844
C 0.801959 4.455685 1.118440
H 1.031126 5.455618 1.480597
C 1.679484 3.784052 0.272303
H 2.609306 4.259373 -0.038900
C 1.389016 2.508138 -0.187113
H 2.090250 2.007666 -0.847308
C -2.169233 -0.714381 1.509086

C -3.296331 -1.519448 1.705622
H -3.896062 -1.835474 0.852854
C -3.638121 -1.941439 2.984677
H -4.513007 -2.571896 3.131159
C -2.855984 -1.567290 4.074078
H -3.124314 -1.900670 5.074804
C -1.724218 -0.782746 3.882022
H -1.100776 -0.502396 4.728505
C -1.376908 -0.363262 2.602844
H -0.473599 0.221064 2.438378
C -1.871126 -1.641703 -1.190625
C -1.586791 -2.911694 -0.684487
H -1.290832 -3.032376 0.355160
C -1.670221 -4.023340 -1.513458
H -1.438401 -5.009694 -1.116997
C -2.042173 -3.874206 -2.845071
H -2.105515 -4.746292 -3.493099
C -2.338024 -2.611257 -3.348296
H -2.635381 -2.493029 -4.388252
C -2.253660 -1.496141 -2.524899
H -2.480561 -0.508449 -2.920931
C -2.900579 1.037906 -0.713623
C -4.200841 1.089935 -0.210602
H -4.510622 0.419634 0.586605
C -5.105631 2.017798 -0.714318
H -6.117846 2.054134 -0.316390
C -4.717007 2.900913 -1.715938
H -5.426355 3.629080 -2.104749
C -3.417222 2.861853 -2.210290
H -3.103533 3.562941 -2.981036
C -2.509099 1.937592 -1.709475
H -1.481128 1.914624 -2.068943
Si 1.179401 -0.616807 -1.128915
H 1.493755 0.573026 -2.356190
H 1.281923 -0.445056 -2.650547
Si 2.795898 -1.073453 1.293045
Si 4.274261 0.115356 -1.090241
C 1.448809 -2.370139 1.508825
H 0.446325 -1.934700 1.444881
H 1.532531 -3.155107 0.745686
H 1.545310 -2.838659 2.497618
C 4.408623 -1.934898 1.736387
H 5.292931 -1.293837 1.648445
H 4.345379 -2.263675 2.782600
H 4.567768 -2.824209 1.114304
C 2.521576 0.325542 2.513056
H 1.632443 0.910101 2.250131
H 2.400413 -0.062960 3.533550
H 3.370677 1.019885 2.515142
C 4.009635 1.033296 -2.710561
H 3.608059 0.385968 -3.499766
H 3.361125 1.912922 -2.629059
H 4.999451 1.377629 -3.042715
C 5.452006 -1.288158 -1.514766
H 6.331057 -0.898158 -2.045376
H 5.807093 -1.833743 -0.634818
H 4.951874 -2.007796 -2.176070
C 5.105094 1.320953 0.092579
H 5.415858 0.850772 1.032981
H 6.000225 1.756504 -0.370492

H 4.414819 2.137512 0.342254
N 2.787346 -0.495573 -0.374727

$^{Ph}YSiC_6F_5$
E = -2327.84652376
P -1.716075 -0.155098 -0.096324
C -0.299394 0.682031 -0.594049
C 0.026870 1.974210 0.032559
C 1.134918 2.739750 -0.382849
H 1.771651 2.364632 -1.180554
C 1.440995 3.964207 0.193774
H 2.309611 4.513691 -0.166289
C 0.658456 4.489977 1.216514
H 0.900776 5.449834 1.667581
C -0.437734 3.754787 1.651930
H -1.068505 4.134817 2.454229
C -0.742700 2.527884 1.078927
H -1.604027 1.990297 1.470599
C -3.203719 0.893433 -0.244178
C -3.184286 1.849502 -1.263203
H -2.289098 1.948927 -1.875814
C -4.283851 2.673498 -1.467902
H -4.260215 3.419068 -2.260047
C -5.403497 2.555926 -0.651025
H -6.262574 3.205684 -0.806859
C -5.419315 1.617556 0.375736
H -6.288194 1.533636 1.025473
C -4.322730 0.788073 0.582912
H -4.338260 0.074091 1.402022
C -2.010236 -1.617405 -1.123161
C -1.315636 -2.795399 -0.830088
C -1.471232 -3.909631 -1.643056
C -2.319235 -3.854770 -2.745246
C -3.013867 -2.685392 -3.034932
C -2.861754 -1.564891 -2.225908
C -1.690114 -0.837554 1.596199
C -2.649686 -1.763393 2.020073
H -3.449813 -2.067854 1.346240
C -2.564480 -2.324986 3.287426
H -3.310379 -3.048160 3.611261
C -1.515973 -1.974463 4.133670
H -1.446679 -2.418804 5.124776
C -0.547109 -1.073025 3.707161
H 0.283976 -0.813561 4.359517
C -0.627606 -0.509265 2.438437
H 0.140637 0.175917 2.086589
Si 0.806315 -0.271754 -1.683412
H 0.915975 0.123433 -3.161705
H 1.260227 1.017577 -2.659229
C 2.570138 -0.308305 -0.979761
C 3.953208 -1.170992 0.842704
C 4.926217 0.294327 -0.795887
C 5.055091 -0.483874 0.347691
H -0.645847 -2.832269 0.027510
H -0.924181 -4.822610 -1.417879
H -2.440154 -4.729589 -3.381300
H -3.679198 -2.643596 -3.894826
H -3.404375 -0.649582 -2.453011
C 3.696090 0.367243 -1.437107
C 2.745580 -1.076079 0.167497

F 1.707490 -1.755779 0.668144
F 3.623090 1.150926 -2.511760
F 5.972710 0.959315 -1.258506
F 6.220381 -0.567897 0.964985
F 4.069763 -1.912392 1.933268

^{Ph}YSiPy
E = -1846.99403111
P -1.026107 -0.217048 -0.020287
C 0.525649 0.306072 -0.535895
C 0.894511 1.719866 -0.351150
C 2.009660 2.272140 -1.011869
H 2.606763 1.629491 -1.655744
C 2.364183 3.605173 -0.866303
H 3.235368 3.981977 -1.400392
C 1.624571 4.457000 -0.051360
H 1.904643 5.502157 0.062593
C 0.523519 3.938552 0.620141
H -0.072257 4.577800 1.270203
C 0.171615 2.602635 0.479990
H -0.690594 2.244114 1.039667
C -2.330774 0.933264 -0.576114
C -2.104173 1.603087 -1.782001
H -1.160061 1.450300 -2.302940
C -3.064024 2.469916 -2.288769
H -2.878399 2.992499 -3.224909
C -4.250176 2.680822 -1.592927
H -4.999413 3.364332 -1.987877
C -4.472069 2.028515 -0.385018
H -5.392986 2.201485 0.168308
C -3.515154 1.158705 0.125968
H -3.690896 0.671935 1.081406
C -1.453522 -1.838618 -0.710315
C -1.073691 -3.004354 -0.040313
H -0.572897 -2.940261 0.923651
C -1.326220 -4.245618 -0.609621
H -1.020946 -5.150414 -0.088351
C -1.957854 -4.329526 -1.846012
H -2.153049 -5.303036 -2.291677
C -2.341199 -3.170695 -2.513112
H -2.838475 -3.234775 -3.478756
C -2.091014 -1.926074 -1.948366
H -2.390296 -1.020585 -2.472091
C -1.254835 -0.445747 1.780178
C -2.357953 -1.123001 2.310782
H -3.124470 -1.521131 1.646814
C -2.463033 -1.318004 3.682216
H -3.320591 -1.849730 4.089886
C -1.465541 -0.845321 4.530999
H -1.548137 -1.001090 5.605000
C -0.356682 -0.190544 4.006613
H 0.431565 0.164995 4.666973
C -0.246234 0.002253 2.633626
H 0.629738 0.490623 2.208773
Si 1.645881 -1.022024 -1.115046
H 1.763993 -1.267071 -2.635949
H 2.183940 -0.283758 -2.532983
C 3.428682 -0.845698 -0.512875
N 4.366171 -1.512009 -1.200390
C 5.057421 -0.084230 1.085376

C 5.620345 -1.465889 -0.775121
C 6.024650 -0.771568 0.363032
H 5.325483 0.478460 1.978632
H 6.354862 -2.016289 -1.368458
H 7.068816 -0.773141 0.668349
C 3.742066 -0.117623 0.641710
H 2.958031 0.426291 1.166609

^{Ph}YSiPyr
E = -1863.05446373
P -1.051894 -0.187158 -0.030136
C 0.434422 0.289867 -0.739655
C 0.936213 1.652206 -0.488036
C 2.007067 2.179065 -1.234346
H 2.470834 1.553588 -1.994887
C 2.483987 3.464738 -1.029855
H 3.313462 3.825720 -1.636616
C 1.917806 4.290212 -0.062612
H 2.294093 5.298202 0.099742
C 0.867061 3.792519 0.698541
H 0.410310 4.409768 1.470963
C 0.392484 2.503080 0.496074
H -0.417217 2.154379 1.134412
C -2.337574 1.075922 -0.318195
C -2.235083 1.801388 -1.508944
H -1.392046 1.611461 -2.171679
C -3.181424 2.768648 -1.820963
H -3.090746 3.333510 -2.746513
C -4.230193 3.025832 -0.944108
H -4.968412 3.788328 -1.185310
C -4.326673 2.317784 0.248973
H -5.137701 2.526346 0.943931
C -3.382915 1.346662 0.565287
H -3.456354 0.818402 1.511876
C -1.674064 -1.728077 -0.758139
C -1.332501 -2.959857 -0.194965
H -0.739287 -2.995998 0.716430
C -1.741367 -4.139496 -0.804770
H -1.466006 -5.096548 -0.366712
C -2.492274 -4.095531 -1.974260
H -2.811338 -5.020564 -2.450762
C -2.836496 -2.869944 -2.536011
H -3.426182 -2.833592 -3.449730
C -2.428595 -1.687569 -1.931695
H -2.697197 -0.729949 -2.372851
C -0.992507 -0.535255 1.761861
C -2.058587 -1.111740 2.460864
H -2.984380 -1.357490 1.941661
C -1.925791 -1.407499 3.811465
H -2.754761 -1.860341 4.352027
C -0.726911 -1.137592 4.467284
H -0.623652 -1.371989 5.525253
C 0.343009 -0.590443 3.768387
H 1.287549 -0.401087 4.274322
C 0.218827 -0.295175 2.414095
H 1.069383 0.088858 1.848075
Si 1.481533 -1.083060 -1.318492
H 1.609702 -1.371786 -2.828589
H 2.076399 -0.403559 -2.732291
C 3.260876 -0.949895 -0.693518

N 3.407459 -0.402218 0.520208
N 4.263984 -1.446647 -1.430194
C 4.638828 -0.316887 1.007263
C 5.485597 -1.357783 -0.930453
C 5.745330 -0.779864 0.309413
H 4.747322 0.139326 1.994142
H 6.298543 -1.762308 -1.539069
H 6.753209 -0.702295 0.709102

^{Ph}YSiB2
E = -2006.01208498
P -1.643538 -0.220405 -0.009521
C -0.132294 0.246325 -0.670893
C 0.310355 1.641338 -0.503391
C 1.350571 2.176439 -1.287137
H 1.837775 1.536198 -2.019072
C 1.766135 3.493542 -1.159999
H 2.575684 3.856998 -1.791502
C 1.161796 4.347113 -0.242049
H 1.489501 5.379711 -0.141468
C 0.133789 3.846809 0.548218
H -0.354864 4.487642 1.280840
C -0.277242 2.526275 0.425233
H -1.074034 2.179712 1.081126
C -2.942978 1.000138 -0.401481
C -2.811515 1.688664 -1.611078
H -1.936799 1.503195 -2.232719
C -3.772514 2.615638 -1.993873
H -3.659780 3.152569 -2.933509
C -4.864769 2.868269 -1.170239
H -5.614404 3.599013 -1.467881
C -4.990771 2.196761 0.040908
H -5.836715 2.401856 0.693972
C -4.032163 1.266973 0.428308
H -4.130259 0.766979 1.387926
C -2.207238 -1.804927 -0.690736
C -1.855215 -3.004769 -0.067880
C -2.216570 -4.217401 -0.641885
C -2.930277 -4.238264 -1.835076
C -3.284381 -3.044684 -2.456559
C -2.923864 -1.829596 -1.888151
C -1.679690 -0.487994 1.799389
C -2.756926 -1.098799 2.450249
H -3.627143 -1.422516 1.880144
C -2.706123 -1.324478 3.819914
H -3.544086 -1.804091 4.322106
C -1.578221 -0.948909 4.545408
H -1.539193 -1.128021 5.618285
C -0.495831 -0.363061 3.898601
H 0.393263 -0.085750 4.461129
C -0.540158 -0.139591 2.526218
H 0.314364 0.290070 2.003426
Si 0.931724 -1.146270 -1.213918
H 0.931414 -1.404610 -2.756876
H 1.527397 -0.531271 -2.653031
H -1.291462 -2.990444 0.862690
H -1.933565 -5.149223 -0.156822
H -3.212516 -5.189049 -2.283230
H -3.845172 -3.059189 -3.388861
H -3.200138 -0.896989 -2.375822

C 5.039618 -0.912095 -0.736410
C 4.590856 -0.193998 0.369564
C 6.383849 -1.114629 -0.977833
C 5.464866 0.358156 1.284427
C 7.277062 -0.561380 -0.056630
H 6.720296 -1.677143 -1.844579
C 6.827794 0.159246 1.049269
H 5.099781 0.920613 2.139498
H 8.346118 -0.695523 -0.207675
H 7.553068 0.577643 1.743963
B 2.841527 -0.851101 -0.792590
O 3.230845 -0.157315 0.349731
O 3.964786 -1.329145 -1.460717

^{Ph}YSiB1
E = -2275.19632499
P 1.779359 -0.789674 -0.009784
C 0.217407 -0.161806 0.293389
C 0.024720 0.719667 1.453480
C 1.065192 1.495878 2.007651
H 2.062806 1.452672 1.573513
C 0.862008 2.337442 3.092357
H 1.699276 2.918266 3.477108
C -0.394542 2.450305 3.679318
H -0.556915 3.117806 4.523218
C -1.434755 1.678273 3.169020
H -2.425536 1.730218 3.618563
C -1.228857 0.830246 2.089750
H -2.057274 0.226705 1.726094
C 2.995994 0.260695 -0.881042
C 4.175971 -0.265177 -1.419312
H 4.392262 -1.328625 -1.321462
C 5.061590 0.562247 -2.096761
H 5.977008 0.150094 -2.517114
C 4.770888 1.916587 -2.244516
H 5.465170 2.565496 -2.775305
C 3.589512 2.436860 -1.729537
H 3.352370 3.489946 -1.857618
C 2.695221 1.610217 -1.055557
H 1.751799 2.000661 -0.673234
C 1.693607 -2.269326 -1.058637
C 1.694122 -2.131071 -2.448730
H 1.820206 -1.147391 -2.896721
C 1.522147 -3.246186 -3.258639
H 1.515555 -3.131764 -4.340634
C 1.350920 -4.502485 -2.687108
H 1.215709 -5.375619 -3.322677
C 1.354611 -4.644293 -1.303046
H 1.225611 -5.626984 -0.853874
C 1.524498 -3.531271 -0.488296
H 1.524603 -3.643694 0.593993
C 2.578314 -1.294862 1.552685
C 3.951699 -1.211621 1.782922
H 4.612612 -0.799840 1.024919
C 4.481589 -1.636290 2.996690
H 5.553547 -1.565387 3.170412
C 3.645701 -2.140091 3.986889
H 4.063227 -2.466905 4.937421
C 2.273740 -2.212464 3.766766
H 1.613433 -2.588524 4.545562

C 1.739410 -1.787503 2.557475
H 0.664373 -1.810608 2.386325
Si -1.124029 -0.611586 -0.896029
N -4.058077 0.464159 -0.389203
C -3.670816 2.662341 -0.237318
C -4.610434 1.707314 -0.087330
H -3.769625 3.737082 -0.137375
H -5.660355 1.825991 0.151349
B -2.659556 0.652292 -0.717222
N -2.477014 2.078698 -0.651003
C -1.337213 2.863917 -0.917779
C -0.840237 3.735875 0.051689
C -0.739991 2.820455 -2.179005
C 0.209490 4.592251 -0.257577
H -1.273279 3.721210 1.050024
C 0.308013 3.680905 -2.482769
H -1.133176 2.131471 -2.923861
C 0.772826 4.582849 -1.529725
H 0.588972 5.269903 0.504679
H 0.755085 3.654150 -3.474894
H 1.577826 5.273308 -1.775544
C -4.762591 -0.737812 -0.256101
C -4.461635 -1.813231 -1.097039
C -5.758924 -0.893566 0.712354
C -5.134370 -3.020068 -0.962435
H -3.704179 -1.682554 -1.869561
C -6.438131 -2.099509 0.830120
H -5.982602 -0.073680 1.390750
C -6.129763 -3.170425 -0.001978
H -4.885383 -3.846805 -1.625358
H -7.208781 -2.205286 1.591658
H -6.661179 -4.114548 0.096994
H -2.077308 -1.508922 0.164566
H -1.648002 -2.053591 -0.657108

$^{F}Y_2Si$
E = -3900.70679059
P 3.078275 0.624163 0.166503
P -2.575477 1.335420 0.008691
C 1.489921 0.021647 0.058211
C 1.224717 -1.290781 -0.538737
C 1.849578 -2.478204 -0.141216
C 1.463961 -3.727671 -0.600361
C 0.444181 -3.828802 -1.539638
C -0.160221 -2.671997 -2.011509
C 0.259603 -1.435366 -1.542180
C 3.860140 0.531783 1.812200
C 3.260491 -0.269838 2.783763
H 2.362140 -0.830580 2.532490
C 3.814535 -0.341420 4.058348
H 3.343336 -0.964691 4.815540
C 4.962371 0.381095 4.362517
H 5.392569 0.323686 5.360779
C 5.561422 1.185057 3.394578
H 6.456974 1.754640 3.635544
C 5.009253 1.266639 2.123455
H 5.464751 1.909450 1.370749
C 3.222056 2.400842 -0.228463
C 3.661624 2.833364 -1.481231
H 3.951832 2.110108 -2.239487

C 3.756813 4.192788 -1.759397
H 4.116611 4.518786 -2.733447
C 3.407270 5.129779 -0.792597
H 3.489422 6.193436 -1.009682
C 2.958493 4.705044 0.453180
H 2.677115 5.432870 1.211467
C 2.870194 3.348425 0.739316
H 2.534301 3.021649 1.720324
C 4.136927 -0.285059 -0.990600
C 3.660644 -0.472021 -2.293700
H 2.692356 -0.057850 -2.572766
C 4.408587 -1.197980 -3.210524
H 4.033061 -1.342639 -4.221457
C 5.627903 -1.751398 -2.829830
H 6.210425 -2.327685 -3.546064
C 6.096413 -1.579296 -1.532258
H 7.042758 -2.023302 -1.230394
C 5.353458 -0.849374 -0.610388
H 5.713491 -0.732987 0.409165
C -1.516071 0.195076 0.770410
C -1.934526 -1.167229 1.087203
C -1.088661 -2.180280 1.599671
C -1.472289 -3.504545 1.761028
C -2.774809 -3.909986 1.517774
C -3.671425 -2.945486 1.085602
C -3.255529 -1.635177 0.917994
C -3.942986 1.905212 1.077244
C -3.652486 2.016145 2.438658
H -2.661333 1.733904 2.793230
C -4.624288 2.463604 3.325564
H -4.391739 2.548958 4.385248
C -5.894559 2.787278 2.859891
H -6.659009 3.129810 3.554748
C -6.192076 2.657601 1.507181
H -7.190378 2.892806 1.143313
C -5.220794 2.217537 0.614869
H -5.469021 2.099234 -0.436804
C -1.637378 2.840465 -0.394907
C -0.702837 2.771650 -1.437017
H -0.550983 1.834449 -1.966207
C 0.041790 3.891231 -1.773305
H 0.783873 3.828316 -2.566028
C -0.154123 5.089704 -1.091594
H 0.433497 5.965987 -1.357919
C -1.096525 5.166525 -0.074331
H -1.255971 6.104263 0.454362
C -1.841526 4.044606 0.275887
H -2.572401 4.107583 1.078222
C -3.276283 0.904797 -1.619875
C -3.822810 1.906484 -2.428283
H -3.860980 2.934726 -2.070506
C -4.295396 1.598407 -3.697761
H -4.722056 2.381829 -4.321037
C -4.205901 0.294186 -4.175481
H -4.567349 0.055259 -5.173941
C -3.639504 -0.699532 -3.385000
H -3.541698 -1.715421 -3.760471
C -3.178267 -0.395814 -2.110631
H -2.713212 -1.168783 -1.505393
Si 0.176515 0.918454 0.984736

H 0.506755 1.283749 2.442354
H 0.289498 0.235188 2.585632
F 2.808374 -2.430223 0.780099
F 2.055377 -4.823932 -0.152447
F 0.063549 -5.013509 -1.989530
F -0.574902 -4.383636 2.184195
F -1.106786 -2.754153 -2.941792
F 0.174341 -1.938281 1.954310
F -0.278623 -0.355900 -2.108456
F -3.146164 -5.171085 1.681147
F -4.200718 -0.779734 0.500411
F -4.934256 -3.268117 0.847819

^{F}YSiCl
E = -2556.40629877
P 0.933145 0.142769 0.253410
C -0.110732 -1.052188 -0.420891
C -1.548580 -0.764177 -0.492080
C -2.075530 0.454436 -0.935691
C -3.432175 0.741751 -0.937211
C -4.335455 -0.214687 -0.493884
C -3.861224 -1.444152 -0.053113
C -2.496530 -1.692587 -0.037314
C 1.857074 1.195869 -0.908756
C 2.893661 2.010646 -0.441985
H 3.153480 2.009786 0.616294
C 3.598243 2.812697 -1.329454
H 4.403067 3.448196 -0.965469
C 3.279971 2.792895 -2.685406
H 3.837617 3.416250 -3.381923
C 2.259718 1.972233 -3.152264
H 2.019775 1.947906 -4.213100
C 1.546614 1.172886 -2.265851
H 0.753122 0.517679 -2.617580
C 2.257017 -0.563114 1.280119
C 3.375995 -1.112692 0.642443
H 3.455432 -1.085259 -0.443725
C 4.387450 -1.695734 1.395878
H 5.251747 -2.124675 0.893115
C 4.294345 -1.731686 2.783175
H 5.090197 -2.185004 3.371073
C 3.185884 -1.183375 3.419367
H 3.112980 -1.202137 4.504846
C 2.168143 -0.600874 2.672439
H 1.311741 -0.165605 3.180917
C -0.053238 1.233685 1.317984
C -0.009502 2.623386 1.218719
H 0.651209 3.099411 0.498331
C -0.832764 3.400898 2.026141
H -0.799989 4.485120 1.942072
C -1.703028 2.797390 2.926675
H -2.349925 3.410217 3.551381
C -1.758473 1.409442 3.020203
H -2.449145 0.933671 3.713291
C -0.942100 0.627958 2.214285
H -1.000627 -0.459172 2.257942
Si 0.509338 -2.575199 -1.194686
H 0.905164 -3.774736 -0.350597
H 1.583942 -2.954501 0.052009
Cl 2.133648 -2.226351 -2.512579

F -2.090076 -2.853314 0.466647
F -4.713070 -2.354921 0.392075
F -5.634284 0.037860 -0.498729
F -3.869543 1.913833 -1.370800
F -1.248405 1.400611 -1.383831

[F]YSiHMDS
E = -2970.20311110
P -1.889420 -0.451287 -0.185933
C -0.204653 -0.172977 -0.275062
C 0.315760 1.181322 -0.193805
C -0.093630 2.098337 0.784056
C 0.431203 3.373924 0.904237
C 1.436031 3.786065 0.037401
C 1.879048 2.909437 -0.945381
C 1.314196 1.647975 -1.056778
C -2.610826 -0.965288 1.406285
C -3.990236 -1.113816 1.582904
H -4.672281 -0.911378 0.757498
C -4.493600 -1.501755 2.818216
H -5.567379 -1.613565 2.955111
C -3.624797 -1.740251 3.880530
H -4.021611 -2.044341 4.847279
C -2.254393 -1.578647 3.711186
H -1.574203 -1.752551 4.542402
C -1.748871 -1.185131 2.477206
H -0.681960 -1.027836 2.332424
C -2.294554 -1.766704 -1.383508
C -3.171999 -2.821187 -1.121593
H -3.646898 -2.912390 -0.148895
C -3.416678 -3.781798 -2.095444
H -4.093613 -4.605704 -1.878570
C -2.792058 -3.700390 -3.335598
H -2.984912 -4.457191 -4.093484
C -1.908791 -2.658519 -3.599411
H -1.402342 -2.598956 -4.560676
C -1.654641 -1.698977 -2.628274
H -0.922494 -0.912747 -2.809435
C -2.812505 1.042813 -0.672602
C -3.486005 1.821735 0.270734
H -3.502530 1.520717 1.314517
C -4.113484 2.999992 -0.117816
H -4.630715 3.602955 0.625607
C -4.072852 3.409897 -1.445035
H -4.563261 4.333714 -1.745700
C -3.398394 2.640305 -2.387864
H -3.358898 2.958345 -3.427573
C -2.768928 1.463907 -2.004517
H -2.240861 0.872358 -2.747654
Si 0.835850 -1.698828 -0.271596
H 1.108279 -1.856398 -1.996964
H 0.664360 -2.662336 -1.448966
Si 2.756645 -1.308685 1.898642
Si 3.915947 -1.247182 -0.947013
C 1.819995 -2.700966 2.747723
H 0.758515 -2.751336 2.478943
H 2.265801 -3.665857 2.473474
H 1.892599 -2.588877 3.838110
C 4.545519 -1.485909 2.440295
H 5.231846 -0.777375 1.963751

H 4.570351 -1.291489 3.521187
H 4.925669 -2.499895 2.275739
C 2.150329 0.352491 2.530629
H 1.059225 0.440994 2.482382
H 2.454620 0.507661 3.574069
H 2.573239 1.165874 1.924614
C 3.519136 -1.538629 -2.758674
H 3.194162 -2.570739 -2.941098
H 2.773572 -0.857345 -3.175801
H 4.461509 -1.390858 -3.306675
C 5.211476 -2.567397 -0.563456
H 5.804414 -2.753141 -1.469050
H 5.909756 -2.286054 0.230637
H 4.734748 -3.514860 -0.280054
C 4.702441 0.450023 -0.752433
H 4.807385 0.733685 0.302375
H 5.705658 0.460474 -1.199135
H 4.110049 1.225332 -1.249092
N 2.528042 -1.413885 0.142839
F 1.739984 0.865711 -2.045611
F 2.818576 3.296964 -1.797961
F 1.953011 5.001494 0.135753
F 0.010521 4.186591 1.862453
F -0.977024 1.706355 1.710083

$^{F}YSiC_6F_5$
E = -2824.73943180
P 1.436062 -0.568637 0.099839
C -0.117960 -0.709968 -0.603104
C -0.712189 -2.049336 -0.763222
C -1.846440 -2.247069 -1.572474
H -2.284777 -1.393079 -2.084097
C -2.413514 -3.501641 -1.746451
H -3.290998 -3.602646 -2.383348
C -1.873665 -4.620753 -1.121870
H -2.320530 -5.603380 -1.256723
C -0.753406 -4.454473 -0.315872
H -0.310399 -5.311287 0.189301
C -0.190786 -3.198696 -0.134712
H 0.676294 -3.120849 0.518657
C 2.615659 -1.725445 -0.670108
C 2.372383 -2.076805 -2.000571
H 1.515961 -1.646256 -2.515985
C 3.209046 -2.975792 -2.644568
H 3.011879 -3.250454 -3.678577
C 4.290549 -3.531500 -1.966714
H 4.944797 -4.238768 -2.472843
C 4.528016 -3.193165 -0.639832
H 5.364861 -3.635530 -0.103363
C 3.689421 -2.295915 0.013164
H 3.867761 -2.062709 1.058891
C 2.052079 1.140969 -0.095750
C 1.591599 2.134144 0.773324
C 2.007559 3.452045 0.671095
C 2.900181 3.808349 -0.333046
C 3.365986 2.847693 -1.220374
C 2.932525 1.532423 -1.102697
C 1.596404 -0.810317 1.903282
C 2.745293 -0.423946 2.599667
H 3.591681 0.009472 2.066950

C 2.803329 -0.570347 3.979529
H 3.697237 -0.265850 4.520227
C 1.713104 -1.094611 4.668859
H 1.758349 -1.205931 5.750476
C 0.561200 -1.457363 3.980154
H -0.299464 -1.846712 4.519827
C 0.494954 -1.307651 2.599112
H -0.418594 -1.551211 2.060374
Si -1.016502 0.845901 -0.878951
H -0.935780 1.514224 -2.256194
H -1.472222 0.593178 -2.486568
C -2.859450 0.774785 -0.458711
C -4.685876 0.024001 0.973075
C -5.160683 1.470811 -0.885878
C -5.600640 0.737851 0.211057
F 0.703694 1.846172 1.709338
F 1.549200 4.368338 1.500842
F 3.301669 5.055875 -0.443965
F 4.209158 3.187700 -2.176729
F 3.386474 0.670306 -1.994038
C -3.808506 1.481030 -1.192236
C -3.338439 0.060268 0.634168
F -3.417905 2.185406 -2.253825
F -2.503101 -0.625113 1.412852
F -5.099227 -0.671423 2.019870
F -6.884081 0.721240 0.524805
F -6.029477 2.148971 -1.617934

^{F}YSiPy
E = -2343.88774585
P -0.507760 0.700097 0.162818
C 1.004888 0.357663 -0.559181
C 2.025100 1.421952 -0.603582
C 3.170156 1.291693 -1.412556
H 3.298250 0.384127 -1.998533
C 4.135166 2.285590 -1.485456
H 5.002032 2.134653 -2.127067
C 4.006194 3.461684 -0.753510
H 4.763704 4.240421 -0.811572
C 2.889895 3.615453 0.059930
H 2.762398 4.521990 0.649690
C 1.927393 2.617606 0.139610
H 1.080303 2.783549 0.803000
C -1.211743 2.275530 -0.430132
C -0.836798 2.684573 -1.712593
H -0.163097 2.057548 -2.293782
C -1.310817 3.883061 -2.224750
H -1.010756 4.199911 -3.221300
C -2.159475 4.680911 -1.462493
H -2.529308 5.621921 -1.865259
C -2.525442 4.282016 -0.182398
H -3.179299 4.908899 0.420429
C -2.048169 3.084114 0.338861
H -2.317931 2.798357 1.351404
C -1.693459 -0.644671 -0.188156
C -1.629198 -1.824474 0.558370
C -2.487773 -2.887608 0.329478
C -3.434955 -2.791944 -0.683201
C -3.514802 -1.638952 -1.451674
C -2.642136 -0.585450 -1.207136

C -0.581287 0.783385 1.989359
C -1.790674 0.734144 2.687849
H -2.734883 0.672144 2.147067
C -1.793051 0.737101 4.076981
H -2.736460 0.693371 4.617561
C -0.589152 0.782771 4.774253
H -0.592108 0.782203 5.862494
C 0.616378 0.809869 4.081929
H 1.558610 0.826160 4.625655
C 0.622909 0.799328 2.691672
H 1.562559 0.784538 2.141203
Si 1.256254 -1.380412 -1.079096
H 1.015494 -1.730852 -2.563018
H 1.874568 -1.081013 -2.610579
C 2.951504 -2.086752 -0.647347
N 3.355534 -3.158251 -1.341331
C 4.921762 -2.151771 0.726944
C 4.515700 -3.717186 -1.027051
C 5.338877 -3.258811 -0.000856
H 5.534151 -1.754777 1.535354
H 4.813429 -4.585096 -1.620384
H 6.279471 -3.760228 0.216522
F -0.706357 -1.976058 1.492536
F -2.398110 -3.991297 1.045464
F -4.254858 -3.794675 -0.913970
F -4.410985 -1.550824 -2.416741
F -2.744381 0.471077 -1.993128
C 3.712270 -1.553535 0.399345
H 3.355608 -0.673486 0.932993

^{F}*YSiPyr*
E = -2359.94742654
P -0.454878 0.669637 0.184067
C 0.915160 0.277530 -0.758203
C 2.041251 1.231064 -0.797019
C 3.072189 1.093892 -1.743900
H 3.034509 0.258707 -2.440860
C 4.128300 1.990139 -1.814373
H 4.900472 1.841834 -2.568007
C 4.208733 3.067387 -0.936936
H 5.038309 3.769272 -0.991782
C 3.211037 3.219681 0.018145
H 3.252411 4.045680 0.726588
C 2.157333 2.318396 0.091740
H 1.415277 2.470314 0.873034
C -1.020143 2.375804 -0.132794
C -0.762128 2.895576 -1.404353
H -0.271792 2.266117 -2.144436
C -1.118235 4.201021 -1.706913
H -0.909175 4.600987 -2.696813
C -1.731086 4.998600 -0.744517
H -2.007745 6.023910 -0.982745
C -1.978327 4.489358 0.524527
H -2.445196 5.113520 1.283708
C -1.617863 3.182228 0.835290
H -1.786197 2.809048 1.840886
C -1.824149 -0.487194 -0.183072
C -1.896475 -1.727298 0.458257
C -2.909126 -2.636210 0.188429
C -3.873723 -2.323878 -0.761045

C -3.818797 -1.108551 -1.429558
C -2.794169 -0.214419 -1.146970
C -0.283763 0.516610 1.996668
C -1.372280 0.578454 2.870886
H -2.378680 0.743908 2.486425
C -1.178330 0.399053 4.234241
H -2.026941 0.443728 4.914051
C 0.100182 0.149391 4.726480
H 0.250981 0.008095 5.795080
C 1.178374 0.057068 3.853726
H 2.172841 -0.165154 4.235207
C 0.990670 0.227812 2.485976
H 1.820638 0.097836 1.789400
Si 1.060190 -1.480041 -1.215532
H 0.759156 -1.916298 -2.663480
H 1.648143 -1.310767 -2.772177
C 2.751550 -2.206862 -0.793553
N 3.347580 -1.686404 0.285814
N 3.240630 -3.215023 -1.525988
C 4.524413 -2.191551 0.634045
C 4.413632 -3.710885 -1.165957
C 5.125458 -3.223362 -0.073349
H 5.006964 -1.756095 1.512111
H 4.808900 -4.532027 -1.769232
H 6.091781 -3.631044 0.212250
F -0.972821 -2.095545 1.327555
F -2.947698 -3.800120 0.808097
F -4.836837 -3.179842 -1.028162
F -4.729683 -0.813989 -2.338488
F -2.774043 0.908527 -1.841994

^{F}YSiB2
E = -2502.91460494
P -1.564088 -0.821280 0.008780
C -0.190660 0.113700 -0.429640
C -0.178404 1.568544 -0.330385
C 0.980486 2.279381 0.026592
C 1.029605 3.663519 0.098503
C -0.116683 4.415292 -0.129471
C -1.292494 3.757227 -0.461230
C -1.303255 2.374031 -0.555269
C -2.773310 -1.234334 -1.298936
C -2.394500 -2.194889 -2.244732
H -1.452207 -2.729123 -2.126453
C -3.209907 -2.464040 -3.336619
H -2.905705 -3.214228 -4.063726
C -4.407863 -1.775695 -3.499272
H -5.047088 -1.987939 -4.354154
C -4.783755 -0.811485 -2.570488
H -5.715178 -0.263497 -2.697926
C -3.970268 -0.535995 -1.477899
H -4.265189 0.233852 -0.770239
C -0.991212 -2.439407 0.627074
C 0.250288 -2.517887 1.265049
C 0.670254 -3.718398 1.824305
C -0.149759 -4.840497 1.767526
C -1.396292 -4.759802 1.155329
C -1.819162 -3.563569 0.587997
C -2.485309 -0.137024 1.416171
C -3.766267 -0.601601 1.726144

H -4.253501 -1.333222 1.084630
C -4.419612 -0.140678 2.861761
H -5.418394 -0.504199 3.094516
C -3.796426 0.778240 3.700252
H -4.311302 1.142159 4.587233
C -2.509870 1.219142 3.412183
H -2.011518 1.923058 4.075415
C -1.849145 0.757682 2.279363
H -0.832301 1.079876 2.071372
Si 1.025253 -0.884894 -1.398749
H 0.975533 -0.467225 -2.926783
H 1.407838 0.324415 -2.482260
H 0.888188 -1.636668 1.320313
H 1.644900 -3.772339 2.304677
H 0.181740 -5.780628 2.204278
H -2.042919 -5.634016 1.114538
H -2.794231 -3.508675 0.108047
C 5.130268 -0.619205 -0.974939
C 4.737925 -0.860332 0.339174
C 6.458748 -0.501253 -1.330558
C 5.656301 -0.993761 1.361221
C 7.396521 -0.635499 -0.303685
H 6.750117 -0.311888 -2.360094
C 7.004125 -0.876118 1.012448
H 5.336920 -1.178447 2.383371
H 8.455483 -0.548660 -0.537247
H 7.762832 -0.972962 1.786103
B 2.935626 -0.707984 -0.912145
O 3.378783 -0.930594 0.387899
O 4.019970 -0.529514 -1.760174
F 2.096478 1.617264 0.317492
F 2.157296 4.271696 0.433116
F -0.084528 5.736183 -0.039409
F -2.458675 1.795266 -0.897554
F -2.396858 4.450382 -0.695170

^{F}YSiB1
E = -2772.10305387
P 1.652668 1.353247 -0.082609
C 0.259946 0.359453 -0.111039
C 0.356002 -0.997569 -0.637175
C 1.390613 -1.877238 -0.282756
C 1.482876 -3.175816 -0.750726
C 0.507797 -3.669560 -1.605839
C -0.543816 -2.843537 -1.976424
C -0.594469 -1.533458 -1.521206
C 2.545826 1.564258 1.496007
C 3.513952 2.560483 1.656029
H 3.754381 3.225846 0.827217
C 4.159699 2.708516 2.876433
H 4.915563 3.481588 3.000379
C 3.831517 1.874727 3.943506
H 4.333855 1.997307 4.901276
C 2.858899 0.893442 3.789266
H 2.597255 0.247387 4.625404
C 2.216389 0.737725 2.565453
H 1.457337 -0.025512 2.413830
C 1.255344 3.082884 -0.511889
C 0.774335 3.933943 0.488373
H 0.697588 3.577427 1.513745

C 0.385390 5.230368 0.176182
H 0.008630 5.883226 0.960927
C 0.477036 5.690385 -1.133447
H 0.174435 6.707108 -1.376706
C 0.960007 4.850240 -2.130853
H 1.040216 5.207410 -3.155558
C 1.346479 3.550522 -1.823931
H 1.728614 2.904757 -2.610614
C 2.845618 0.749261 -1.309402
C 4.198038 0.574788 -1.017917
H 4.580240 0.816603 -0.029417
C 5.056692 0.066719 -1.986418
H 6.110044 -0.072308 -1.752006
C 4.570796 -0.274624 -3.243368
H 5.245335 -0.677590 -3.996366
C 3.219388 -0.114320 -3.534745
H 2.832285 -0.394765 -4.512126
C 2.356445 0.389591 -2.570812
H 1.292773 0.490534 -2.781655
Si -1.270312 0.997299 0.707358
N -4.149017 -0.198608 0.653924
C -3.833676 -2.351671 1.172656
C -4.754554 -1.442875 0.800213
H -3.976909 -3.393088 1.433966
H -5.823275 -1.574398 0.679918
B -2.735595 -0.341105 0.926782
N -2.587673 -1.733695 1.286872
C -1.449562 -2.448749 1.695601
C -1.241266 -3.760229 1.260963
C -0.520545 -1.865012 2.560636
C -0.118618 -4.466918 1.672536
H -1.947707 -4.211770 0.567571
C 0.606224 -2.574002 2.956787
H -0.705763 -0.858402 2.932349
C 0.817851 -3.875268 2.513193
H 0.041416 -5.478128 1.302758
H 1.326600 -2.107451 3.626373
H 1.706370 -4.422769 2.818307
C -4.843447 0.930226 0.195339
C -4.532574 2.192941 0.704486
C -5.844904 0.815001 -0.772708
C -5.198089 3.319826 0.240283
H -3.771841 2.277506 1.479307
C -6.519001 1.944589 -1.218556
H -6.074657 -0.161689 -1.192454
C -6.197832 3.202839 -0.719839
H -4.940816 4.296970 0.645179
H -7.293926 1.839782 -1.975765
H -6.723767 4.085780 -1.076806
H -2.183226 1.364732 -0.632408
H -1.837976 2.228834 -0.020145
F -1.584404 -0.772319 -1.977419
F 2.326707 -1.469528 0.578546
F 2.456851 -3.972135 -0.332365
F -1.481666 -3.301569 -2.794960
F 0.555980 -4.928388 -2.021487

/
E = -1308.98949021
C 1.683067 2.173236 -0.238080

C -0.469624 1.066156 -0.239903
C -1.095655 2.306796 -0.306575
C -0.338155 3.477793 -0.312957
C 1.047157 3.413196 -0.268534
H 2.772773 2.139990 -0.246438
H -2.179400 2.362189 -0.363599
H -0.842605 4.440591 -0.364832
H 1.641582 4.324815 -0.277407
N -1.122681 -0.176859 -0.224604
C -2.493919 -0.353651 -0.017766
C -3.201628 0.366542 0.954169
C -3.176055 -1.324619 -0.759859
C -4.551878 0.123313 1.162469
H -2.677118 1.103524 1.557323
C -4.524079 -1.573772 -0.533805
H -2.636857 -1.870912 -1.532708
C -5.223202 -0.847307 0.423285
H -5.083547 0.690215 1.925066
H -5.033661 -2.334854 -1.122281
H -6.280900 -1.035934 0.594521
C 0.953642 0.980893 -0.225434
C 1.472190 -0.384639 -0.194145
P 3.047046 -0.820457 0.231511
C 4.355091 -0.393797 -0.967439
H 4.365528 0.689951 -1.124256
H 5.343075 -0.719170 -0.620758
H 4.117506 -0.873441 -1.922838
C 3.626421 -0.083568 1.791193
H 4.688357 -0.286228 1.969396
H 3.461444 0.998454 1.750865
H 3.022851 -0.489219 2.609623
C 3.172774 -2.612771 0.431558
H 4.180926 -2.885653 0.760722
H 2.436247 -2.943899 1.171120
H 2.956749 -3.104098 -0.522864
Si 0.055042 -1.476698 -0.467130
H -0.218725 -2.494730 -1.538920
H -0.068622 -1.495752 -2.251411

II
E = -1858.19991151
C 0.762787 2.889278 -0.236736
C 0.379351 1.601061 0.127478
C -0.853411 1.051583 -0.292205
C -1.661306 1.875517 -1.099919
C -1.281405 3.161887 -1.440961
C -0.061945 3.683852 -1.016025
H 1.728897 3.246329 0.114235
H -2.605252 1.478759 -1.464556
H -1.944453 3.759749 -2.064042
H 0.240030 4.692243 -1.288623
N -1.260245 -0.252605 -0.013719
S 1.505452 0.717958 1.194388
O 2.846189 1.304922 0.965991
O 0.951466 0.748167 2.542636
C 1.509156 -0.874190 0.526512
C -2.641558 -0.564039 -0.008726
C -3.525112 0.164595 0.795141
C -3.132619 -1.632845 -0.761578
C -4.873050 -0.163908 0.828301

H -3.137022 0.989882 1.388724
C -4.481084 -1.967115 -0.711746
H -2.443534 -2.198385 -1.386347
C -5.357791 -1.232955 0.079074
H -5.549572 0.412454 1.456768
H -4.848979 -2.802429 -1.304777
H -6.413797 -1.492510 0.113715
P 2.832344 -1.220401 -0.510470
C 3.081455 -0.053201 -1.875582
H 3.952972 -0.326682 -2.480911
H 2.180262 -0.038671 -2.498081
H 3.225543 0.937393 -1.431918
C 2.565731 -2.839404 -1.266529
H 1.645885 -2.820071 -1.860887
H 3.414697 -3.094532 -1.909935
H 2.455971 -3.592136 -0.479399
C 4.407056 -1.302761 0.370000
H 4.538959 -0.336476 0.867402
H 4.343072 -2.091242 1.126760
H 5.237282 -1.500740 -0.317095
Si -0.146150 -1.574833 0.447086
H -0.383144 -1.385505 2.090534
H -0.741167 -2.411699 1.544077

III
E = -2620.28203911
C 0.359755 1.537118 0.568191
C -0.436759 1.547661 -0.582941
C -0.816596 2.768386 -1.141249
C -0.436782 3.969874 -0.559288
C 0.333197 3.960327 0.596653
C 0.726259 2.750251 1.151819
H -1.435013 2.748360 -2.035008
H -0.746429 4.910562 -1.010364
H 0.632773 4.893674 1.069281
H 1.345316 2.718133 2.044984
S 0.952040 0.080214 1.503002
S -1.017506 0.094184 -1.521678
O 2.163329 0.603771 2.169274
O -0.189448 -0.314594 2.341220
O 0.151402 -0.319784 -2.329273
O -2.214778 0.614146 -2.206705
C 1.411626 -1.146970 0.427096
C -1.399219 -1.151685 -0.459583
P -2.842999 -0.938434 0.444519
P 2.904473 -0.866404 -0.387513
C -2.893596 -2.056429 1.853490
H -3.824608 -1.893745 2.407759
H -2.844512 -3.095863 1.515406
H -2.024709 -1.824990 2.479651
C -3.072154 0.725819 1.121418
H -3.091749 1.440661 0.291928
H -4.014545 0.780595 1.677865
H -2.221210 0.935723 1.778687
C -4.314188 -1.247155 -0.565241
H -4.302952 -2.290986 -0.895868
H -5.239679 -1.032637 -0.018694
H -4.225091 -0.599967 -1.445003
C 3.009705 -1.965344 -1.812513
H 3.962963 -1.805835 -2.328255

H 2.926504 -3.007334 -1.488712
H 2.170121 -1.725617 -2.476926
C 3.094500 0.807692 -1.049890
H 3.059398 1.521088 -0.220151
H 4.048090 0.902085 -1.581236
H 2.254296 0.985805 -1.731286
C 4.350486 -1.155085 0.659608
H 5.283995 -0.913119 0.139110
H 4.223421 -0.521031 1.543605
H 4.353052 -2.203946 0.973781
Si 0.110688 -2.217373 -0.284831
H -0.120207 -3.531496 0.455762
H -0.374978 -2.799751 1.243519

IV
E = -1828.26230535
C -0.631190 3.161817 -0.214273
C -0.154731 1.862229 -0.238801
C 1.038151 1.424225 0.340268
C 1.813318 2.399705 0.971000
C 1.380221 3.722255 0.999537
C 0.170630 4.107748 0.414827
H -1.575179 3.429491 -0.684798
H 2.762205 2.124826 1.430286
H 1.999326 4.478056 1.480464
H -0.138732 5.150503 0.442600
B 1.222382 -0.158478 0.110947
S -0.981195 0.435571 -0.858605
O -1.837211 0.661555 -2.025203
O 0.347044 -0.364927 -1.151967
C -1.678971 -0.409127 0.407568
C 2.665757 -0.790639 -0.024974
C 3.667132 -0.059879 -0.681195
C 3.009857 -2.057885 0.456613
C 4.948648 -0.571398 -0.854116
H 3.433851 0.932072 -1.068590
C 4.289171 -2.578763 0.290929
H 2.253791 -2.646323 0.976480
C 5.263873 -1.835634 -0.366167
H 5.705075 0.018059 -1.370433
H 4.527516 -3.568347 0.678247
H 6.267077 -2.238896 -0.495722
P -3.251103 -1.013463 0.133450
C -4.474430 0.285069 -0.170101
H -5.468533 -0.140013 -0.347963
H -4.500206 0.955631 0.694742
H -4.145932 0.845177 -1.052132
C -3.762998 -1.918279 1.606169
H -3.780187 -1.232339 2.458935
H -4.753558 -2.361365 1.457873
H -3.025718 -2.703073 1.808727
C -3.412170 -2.154474 -1.268090
H -3.071311 -1.616853 -2.159970
H -2.751761 -3.011938 -1.099446
H -4.444328 -2.496707 -1.405106
Si -0.231241 -1.161236 1.406498
H -0.515191 -0.735778 2.864635
H -0.059361 0.205465 2.283609

V
Not observed.

Pathway B

$^{Tos}Y_2Si$
E = -4084.06900568
C 3.778949 2.926393 -0.131907
H 3.371071 3.256561 0.820231
C 3.073672 1.996633 -0.879027
C -4.049463 -1.473805 -1.378428
O 0.470470 1.748864 -1.264278
C -4.198532 -0.855530 -2.615919
H -3.332004 -0.441520 -3.131524
C 2.647359 -3.115070 2.319958
H 3.664381 -2.774264 2.508103
C 5.000893 3.388325 -0.610764
H 5.567456 4.109973 -0.023645
C -0.903935 4.925107 0.594370
H -0.303843 5.554092 1.248562
C -1.177328 3.615474 0.969571
H -0.763256 3.223098 1.892447
C 2.836686 -2.195978 -2.594053
H 2.057602 -1.455826 -2.779507
C 3.294174 -2.400785 -1.293359
C 5.251020 -0.464113 0.310783
H 5.457391 -0.937526 -0.646635
C 6.231323 0.297405 0.937580
H 7.207448 0.409647 0.469798
C -1.373922 5.420508 -0.615489
H -1.150341 6.444345 -0.909736
C -1.779015 0.040044 -0.735879
Si -0.123239 0.117488 -1.956782
O -2.655444 -1.944992 0.780719
S -2.429621 -1.563898 -0.629362
P -2.275099 1.073134 0.581622
C -5.463650 -0.760049 -3.173648
H -5.587175 -0.270219 -4.138843
C -4.515288 0.092933 1.945554
H -3.795941 -0.453242 2.548931
C -0.546897 -0.259540 2.276818
H -0.248608 -0.785105 1.376977
C 4.705502 0.793923 2.737658
H 4.486929 1.294086 3.678863
C 3.720228 0.038255 2.117487
H 2.725092 -0.030261 2.555119
C -2.391616 3.291462 -1.091706
H -2.931632 2.649993 -1.782725
C 1.933441 -2.632660 1.221004
C 3.559387 1.534745 -2.098996
H 2.987961 0.800048 -2.665164
C 4.254297 -3.382576 -1.031595
H 4.592562 -3.555712 -0.009885
C 3.995905 -0.604761 0.905006
C -1.341777 1.134671 4.553698
H -1.653279 1.677388 5.443978
C -1.507875 0.739029 2.183145
C -1.914628 1.434997 3.325374
H -2.685452 2.202014 3.255636
C -1.938316 2.795909 0.135502

C -2.115802 4.601043 -1.459082
H -2.467364 4.974831 -2.418311
C 6.850221 3.411812 -2.324426
H 7.581418 2.592546 -2.341269
H 7.255600 4.208921 -1.692936
H 6.775600 3.794572 -3.349756
C -0.383221 0.127235 4.646360
H 0.051582 -0.117684 5.613941
C -6.585389 -1.282446 -2.518161
C 5.961557 0.921374 2.150675
H 6.730264 1.519214 2.636873
C -6.363282 1.405540 0.331185
H -7.080829 1.916972 -0.307242
C 1.455825 -0.303030 -0.483221
P 2.606029 -1.408806 0.059786
O 1.369354 1.934948 1.079753
S 1.566214 1.299749 -0.231228
O -1.622101 -2.467809 -1.456881
C 5.520272 2.935301 -1.822990
C 4.775316 2.007561 -2.562892
H 5.160840 1.648077 -3.516196
C 3.353571 -2.964821 -3.633062
H 2.987520 -2.813233 -4.646421
C 4.324463 -3.925385 -3.376132
H 4.728311 -4.522337 -4.191872
C 4.774886 -4.136102 -2.074135
H 5.528003 -4.895344 -1.872530
C -5.003551 1.581012 0.111475
H -4.678503 2.233120 -0.693125
C 0.007971 -0.574144 3.511778
H 0.736321 -1.379945 3.582065
C -4.067563 0.934807 0.923456
C -6.803566 0.583801 1.363861
H -7.869686 0.449436 1.537324
C -6.399466 -1.919372 -1.291636
H -7.260127 -2.334581 -0.768643
C -5.877620 -0.071918 2.166467
H -6.214368 -0.726597 2.967945
C 0.730803 -4.428096 2.970920
H 0.255822 -5.116862 3.667289
C 2.043027 -4.017018 3.190296
H 2.595325 -4.389496 4.051099
C -5.137826 -2.016859 -0.714871
H -4.985829 -2.486638 0.253924
C -7.953637 -1.131952 -3.112277
H -7.944215 -1.312064 -4.193566
H -8.337060 -0.113579 -2.958585
H -8.668195 -1.824852 -2.655750
C 0.024690 -3.956958 1.867655
H -1.007890 -4.249056 1.690129
C 0.629740 -3.072739 0.984487
H 0.071751 -2.705517 0.123714
H -1.875092 0.785571 -1.840422
H -1.309969 1.217938 -2.730712

^{Tos}YSiCl
E = -2648.09725670
C -2.381881 -0.325453 -1.300390
C -2.725322 1.019543 -1.372987
H -2.063540 1.722907 -1.876509

C 2.938664 3.099542 1.947271
H 3.886064 3.242337 2.462621
C 2.575767 1.828723 1.519853
H 3.250801 0.992040 1.677187
C 2.107546 4.187281 1.700902
H 2.400145 5.181758 2.031899
C 0.368175 0.204565 -1.415572
Si 1.589766 0.820524 -2.891910
O -0.622981 -2.244285 -1.466854
S -0.835097 -0.888252 -2.002241
P 0.815267 0.019717 0.246337
C -3.908037 1.451323 -0.790054
H -4.176965 2.505869 -0.836731
C -0.758760 -1.874114 1.539150
H -0.100506 -2.598682 1.067736
C 2.535938 -2.014790 -0.500029
H 2.134209 -1.849366 -1.496843
C 0.543634 2.738174 0.585324
H -0.385075 2.596749 0.032049
C 3.468729 -2.485813 2.085707
H 3.826043 -2.676822 3.095538
C 2.086054 -1.231550 0.559970
C 2.536461 -1.482388 1.859630
H 2.147556 -0.902238 2.695895
C 1.366322 1.639044 0.851609
C 0.911718 4.007023 1.014880
H 0.267037 4.857240 0.802656
C 3.934729 -3.253186 1.020769
H 4.662206 -4.042638 1.199821
C -4.763311 0.552567 -0.144066
C -2.497091 -0.022137 2.679448
H -3.176842 0.705234 3.118953
O -0.917446 -0.697617 -3.451962
C -1.438454 0.416339 1.894867
H -1.298625 1.481925 1.740162
C 3.461891 -3.024806 -0.266289
H 3.809971 -3.637539 -1.094808
C -0.559850 -0.507211 1.323342
C -2.684709 -1.382147 2.902625
H -3.512806 -1.723436 3.521053
C -4.410808 -0.797928 -0.121237
H -5.068590 -1.514438 0.369353
C -1.816256 -2.304663 2.330840
H -1.963180 -3.370201 2.494716
C -3.225668 -1.242869 -0.692849
H -2.927315 -2.287771 -0.653099
C -6.008128 1.030052 0.540654
H -6.823204 0.303942 0.445937
H -6.351463 1.987342 0.134438
H -5.827291 1.174916 1.615494
H 0.456392 1.683238 -1.801265
H 0.911835 2.363744 -2.365336
Cl 3.363428 1.083372 -1.689591

^{Tos}YSiHMDS
E = -3061.88581469
C -2.471868 -1.979731 -0.015799
C -2.754848 -2.150387 -1.366663
H -1.948187 -2.098072 -2.096821
C 0.148570 4.544699 -2.066253

H 0.535816 5.544341 -1.880793
C 0.051176 3.641187 -1.014868
H 0.381936 3.938183 -0.023455
C -0.232028 4.169835 -3.349701
H -0.147632 4.877322 -4.172237
C -0.129020 -0.423053 -0.528183
Si 1.374151 -1.427666 -1.549676
O -0.887904 -1.125408 1.876175
S -0.794242 -1.664938 0.502888
P -0.598225 1.130489 0.104668
C -4.066288 -2.370503 -1.757781
H -4.295759 -2.499845 -2.814919
C -2.727970 1.153209 1.943763
H -1.967034 1.030614 2.709197
C 1.451467 1.012481 1.947979
H 1.726168 0.106733 1.409033
C -0.811439 1.979491 -2.533427
H -1.174080 0.969429 -2.718581
C 0.687855 3.316039 3.325677
H 0.385243 4.209208 3.868577
C 0.338387 1.740949 1.531961
C -0.049190 2.890021 2.228602
H -0.937811 3.441811 1.924283
C -0.431908 2.351407 -1.238726
C -0.712257 2.885415 -3.581886
H -1.004684 2.582037 -4.584914
C 1.804541 2.591862 3.734986
H 2.377538 2.921817 4.599468
C -5.101530 -2.433869 -0.816959
C -4.682576 1.344371 -0.031488
H -5.442584 1.408093 -0.807416
O -0.051918 -2.917448 0.314483
C -3.339938 1.320056 -0.384983
H -3.064816 1.367482 -1.435335
C 2.182336 1.442710 3.049620
H 3.047696 0.870826 3.376939
C -2.353759 1.234521 0.600723
C -5.050447 1.287725 1.308900
H -6.102654 1.310405 1.586351
C -4.781220 -2.284188 0.532335
H -5.572947 -2.335667 1.278708
C -4.072551 1.190154 2.292542
H -4.355982 1.129624 3.341424
C -3.472268 -2.054641 0.940727
H -3.216973 -1.910196 1.987736
C -6.520470 -2.633879 -1.257751
H -6.937866 -1.704560 -1.669788
H -7.160311 -2.940887 -0.423913
H -6.593202 -3.394844 -2.043493
H 0.485006 0.052143 -1.718191
H 1.222127 -0.073794 -2.477392
Si 3.951857 0.283041 -0.885082
Si 3.462385 -2.527563 0.241482
C 2.807815 -4.118081 -0.503260
H 1.718212 -4.192456 -0.431422
H 3.100604 -4.210428 -1.557098
H 3.246810 -4.963328 0.045127
C 5.339602 -2.672410 0.176643
H 5.883279 -1.779188 0.501430
H 5.642134 -3.499233 0.833656

H 5.669118 -2.924262 -0.839205
C 2.896192 -2.391371 2.026152
H 3.160544 -3.300849 2.581784
H 3.351851 -1.540229 2.547862
H 1.805752 -2.278995 2.065772
C 2.995734 1.842575 -1.298473
H 3.717937 2.668663 -1.361990
H 2.438164 1.812065 -2.240977
H 2.297854 2.088092 -0.491113
C 5.185689 -0.012924 -2.269852
H 4.659785 -0.231667 -3.208217
H 5.814332 0.873083 -2.431357
H 5.845862 -0.860008 -2.050576
N 2.903649 -1.119279 -0.695076
C 4.877756 0.672488 0.721457
H 5.964963 0.614840 0.584093
H 4.636547 1.688566 1.058500
H 4.610765 -0.013687 1.533093

^{Tos}*YSiB1*
E = -2863.77567360
C -3.424535 0.382263 -1.318754
C -3.403221 1.270682 -2.390707
H -2.628117 1.177909 -3.147259
C 3.476283 0.181943 2.156074
H 4.488234 -0.205779 2.067907
C 2.420586 -0.525698 1.593628
H 2.612214 -1.456160 1.065618
C 3.239499 1.383388 2.811959
H 4.066589 1.935139 3.254569
C -0.765967 -0.131127 -0.468622
Si 0.089543 1.211868 -1.619906
O -2.668602 -1.885848 -0.243646
S -2.167527 -0.871063 -1.192990
P -0.284883 -0.947330 0.986378
C -4.379779 2.251753 -2.468809
H -4.366210 2.952512 -3.302670
C -1.857395 -1.915256 3.141579
H -1.289176 -2.839224 3.084895
C -0.036171 -3.432855 -0.248854
H -0.538978 -2.981482 -1.098874
C 0.887575 1.180456 2.345105
H -0.122048 1.574366 2.432746
C 1.282922 -4.600394 1.916887
H 1.802215 -5.052009 2.759815
C 0.253453 -2.674262 0.882855
C 0.919816 -3.262139 1.966192
H 1.159467 -2.668992 2.847514
C 1.116311 -0.040650 1.704695
C 1.947384 1.892043 2.889036
H 1.762809 2.846521 3.376737
C 0.979601 -5.359590 0.789380
H 1.260252 -6.410565 0.751192
C -5.388118 2.350448 -1.503733
C -3.387973 0.406590 3.257618
H -3.993225 1.310586 3.285678
O -1.866104 -1.283877 -2.569572
C -2.386438 0.280450 2.303201
H -2.236252 1.073553 1.571652
C 0.325584 -4.775055 -0.286935

H 0.098364 -5.360082 -1.174737
C -1.603781 -0.875372 2.248238
C -3.625317 -0.626364 4.157893
H -4.413540 -0.531896 4.902270
C -5.400888 1.424814 -0.458530
H -6.194558 1.470871 0.286243
C -2.863178 -1.787515 4.092399
H -3.058363 -2.607924 4.780108
C -4.427350 0.437130 -0.359847
H -4.441127 -0.302765 0.436634
C -6.432490 3.421424 -1.603469
H -6.946240 3.385575 -2.572261
H -5.983674 4.419508 -1.515997
H -7.186870 3.321934 -0.816333
H -0.624870 1.335689 -0.050631
H -0.302903 2.247023 -0.348278
N 3.012074 2.047842 -0.734094
C 4.214603 0.226584 -1.237710
C 4.287015 1.491374 -0.778121
H 5.028982 -0.454094 -1.452836
H 5.173727 2.063557 -0.534923
C 2.514401 -1.351128 -2.014534
C 1.400135 -1.482101 -2.845434
C 3.268188 -2.493524 -1.718271
C 1.062818 -2.714047 -3.388878
H 0.798888 -0.613833 -3.112531
C 2.939901 -3.718801 -2.283068
H 4.116730 -2.420386 -1.041590
C 1.842738 -3.835550 -3.130223
H 0.176996 -2.780158 -4.016511
H 3.543655 -4.593026 -2.046311
H 1.588612 -4.797092 -3.571855
C 2.793045 3.368816 -0.309051
C 1.742757 4.121619 -0.840038
C 3.630859 3.965248 0.639102
C 1.513493 5.419556 -0.401825
H 1.118671 3.696270 -1.622771
C 3.406155 5.268631 1.061424
H 4.451063 3.393235 1.064573
C 2.339479 6.002272 0.553357
H 0.687202 5.985078 -0.828579
H 4.069114 5.711005 1.803076
H 2.160801 7.021283 0.889725
N 2.889151 -0.093508 -1.512593
B 2.059744 1.051156 -1.186770

$^{Ph}Y_2Si$
E = -2906.89499356
P 2.975321 0.362773 -0.071366
P -3.004461 0.425580 -0.070158
C 1.496034 -0.043392 -0.797270
C 1.228633 -1.407942 -1.249592
C 1.752655 -2.566336 -0.647680
H 2.373782 -2.465616 0.239476
C 1.444773 -3.834046 -1.118855
H 1.862697 -4.704767 -0.615734
C 0.580123 -4.002132 -2.197106
H 0.328669 -4.998318 -2.555572
C 0.046474 -2.872901 -2.810925
H -0.619670 -2.982101 -3.666653

C 0.378180 -1.603539 -2.356320
H 0.006835 -0.722899 -2.883741
C 3.176860 0.037141 1.725899
C 3.718309 -1.166787 2.186986
H 4.167321 -1.865472 1.483133
C 3.696158 -1.479478 3.540756
H 4.127401 -2.418028 3.883769
C 3.119181 -0.600865 4.452003
H 3.097004 -0.848540 5.511525
C 2.569942 0.596129 4.002891
H 2.115379 1.287395 4.709977
C 2.600090 0.915900 2.650923
H 2.164483 1.851981 2.306785
C 3.324688 2.143697 -0.252976
C 4.150840 2.827033 0.642418
H 4.534258 2.322176 1.526621
C 4.488524 4.154920 0.408873
H 5.130417 4.680314 1.113357
C 4.008519 4.808143 -0.721660
H 4.269949 5.849522 -0.900812
C 3.203747 4.125717 -1.627330
H 2.833500 4.628536 -2.518390
C 2.870439 2.795551 -1.401737
H 2.259013 2.248847 -2.115993
C 4.423949 -0.433791 -0.844594
C 4.309218 -0.982338 -2.122607
H 3.338252 -1.005411 -2.610721
C 5.433053 -1.494909 -2.760731
H 5.335043 -1.931865 -3.752461
C 6.675447 -1.449229 -2.137335
H 7.552986 -1.850910 -2.640744
C 6.797949 -0.882271 -0.872494
H 7.770381 -0.832662 -0.386395
C 5.676643 -0.374395 -0.228530
H 5.776440 0.073880 0.759115
C -1.339331 0.066260 0.130041
C -1.033112 -1.225893 0.789160
C -0.135907 -1.274895 1.867969
H 0.313884 -0.351061 2.222770
C 0.186272 -2.471733 2.492726
H 0.882542 -2.464738 3.329572
C -0.380286 -3.667937 2.060884
H -0.126185 -4.607570 2.548200
C -1.268472 -3.644801 0.991668
H -1.705932 -4.571538 0.623190
C -1.581055 -2.445844 0.362578
H -2.237559 -2.459202 -0.505389
C -3.952349 0.084374 1.450106
C -4.157141 1.088150 2.399197
H -3.841874 2.108109 2.190976
C -4.771481 0.792692 3.610080
H -4.933070 1.584062 4.339434
C -5.178444 -0.508140 3.888113
H -5.655174 -0.738920 4.838950
C -4.977813 -1.512308 2.947483
H -5.292546 -2.532153 3.159307
C -4.372191 -1.219707 1.731443
H -4.225611 -2.008425 0.997714
C -3.246090 2.183134 -0.460567
C -4.192451 2.607861 -1.393938

H -4.792767 1.880452 -1.934113
C -4.363155 3.964417 -1.648989
H -5.099932 4.285031 -2.382768
C -3.592578 4.904654 -0.975443
H -3.726086 5.965534 -1.178960
C -2.644270 4.487598 -0.047143
H -2.028266 5.217450 0.473825
C -2.466291 3.134085 0.207205
H -1.713979 2.817200 0.925754
C -3.947337 -0.464980 -1.367338
C -5.334571 -0.625986 -1.314518
H -5.895802 -0.246967 -0.461782
C -5.997900 -1.274774 -2.349891
H -7.077725 -1.403565 -2.304393
C -5.282198 -1.763196 -3.439711
H -5.803989 -2.274896 -4.246358
C -3.901284 -1.604729 -3.494257
H -3.338759 -1.992505 -4.341423
C -3.235466 -0.957967 -2.459406
H -2.152282 -0.852146 -2.468676
Si 0.048837 1.179901 -0.797347
H -0.511304 1.096134 0.953924
H 0.258504 1.728443 0.976649

^{Ph}YSiCl
E = -2059.50608727
P -0.534757 -0.143488 -0.040760
C 0.827092 0.647168 -0.727156
C 2.178051 0.141206 -0.415460
C 3.244646 0.362892 -1.306805
H 3.068656 0.938852 -2.213611
C 4.517992 -0.124085 -1.053979
H 5.314338 0.070061 -1.770948
C 4.784169 -0.853773 0.101569
H 5.783893 -1.235737 0.298012
C 3.752195 -1.071911 1.006311
H 3.937471 -1.627001 1.924737
C 2.479229 -0.573682 0.758210
H 1.701147 -0.743784 1.501734
C -2.037783 0.100241 -1.040674
C -2.353791 -0.800977 -2.059675
H -1.735372 -1.681678 -2.220106
C -3.463303 -0.579295 -2.866343
H -3.704922 -1.287074 -3.656635
C -4.262566 0.540696 -2.661755
H -5.131085 0.712302 -3.294714
C -3.951363 1.441052 -1.648454
H -4.570667 2.321081 -1.488369
C -2.843103 1.224423 -0.839360
H -2.596147 1.938599 -0.056597
C -1.009061 0.468246 1.613556
C -2.245982 0.188477 2.204157
H -2.977629 -0.422438 1.676291
C -2.551988 0.709951 3.454312
H -3.515036 0.492079 3.911918
C -1.632107 1.521326 4.114893
H -1.877160 1.933577 5.091896
C -0.409829 1.817556 3.522831
H 0.302868 2.463800 4.030727
C -0.097335 1.295629 2.271722

H 0.850628 1.537596 1.789513
C -0.287499 -1.940347 0.064950
C 0.473279 -2.549884 -0.937876
H 0.926199 -1.935919 -1.714176
C 0.671190 -3.923959 -0.920631
H 1.270849 -4.391527 -1.698718
C 0.119112 -4.696128 0.097011
H 0.280636 -5.772274 0.112438
C -0.628954 -4.091377 1.100731
H -1.052796 -4.691086 1.903644
C -0.832354 -2.715641 1.088343
H -1.402600 -2.249400 1.887736
Si 0.417067 2.517123 -1.206945
Cl 2.284806 3.425361 -0.763133
H 0.913808 1.931225 -2.821057
H 0.914026 1.153399 -2.234395

^{Ph}YSiHMDS
E = -2473.30116145
P -1.693447 0.228292 -0.107178
C -0.052819 -0.144480 -0.403007
C 0.332303 -1.488935 0.116382
C -0.034342 -2.670256 -0.545959
H -0.591674 -2.600465 -1.478906
C 0.299628 -3.921369 -0.038819
H -0.002648 -4.819980 -0.574300
C 1.027800 -4.024313 1.142360
H 1.296623 -5.001640 1.539065
C 1.409503 -2.864734 1.811304
H 1.976174 -2.931233 2.739098
C 1.059086 -1.618136 1.307000
H 1.332760 -0.714968 1.845643
C -2.889477 -0.679190 -1.158101
C -4.155903 -1.059750 -0.710485
H -4.453925 -0.856189 0.316221
C -5.036531 -1.701141 -1.575631
H -6.019736 -2.003297 -1.219985
C -4.661947 -1.957064 -2.890671
H -5.352207 -2.461232 -3.564430
C -3.403739 -1.571282 -3.344286
H -3.108695 -1.768936 -4.372899
C -2.520402 -0.937583 -2.479415
H -1.528313 -0.642206 -2.820771
C -2.100534 1.974162 -0.375535
C -3.185218 2.367702 -1.159923
H -3.804093 1.621911 -1.652623
C -3.475884 3.719158 -1.317452
H -4.321348 4.019282 -1.933086
C -2.690503 4.680617 -0.692900
H -2.920154 5.736978 -0.819250
C -1.608284 4.292731 0.091668
H -0.986195 5.041334 0.577755
C -1.310743 2.946130 0.245898
H -0.463189 2.638805 0.852292
C -2.207599 -0.195923 1.592924
C -2.273291 -1.547183 1.955201
H -2.116330 -2.321525 1.206534
C -2.534133 -1.906981 3.271070
H -2.574283 -2.960328 3.540946
C -2.738332 -0.927138 4.237157

H -2.942117 -1.210934 5.267908
C -2.686219 0.416014 3.881359
H -2.855482 1.187196 4.630305
C -2.420928 0.782551 2.566370
H -2.388489 1.836304 2.300710
Si 1.246264 1.183503 -1.067024
N 2.804045 0.507545 -0.455528
Si 3.414158 1.305828 0.983579
Si 3.841516 -0.478663 -1.477695
C 1.976209 2.172998 1.847502
H 1.646957 3.046608 1.269785
H 1.111592 1.512331 1.999984
H 2.303356 2.533342 2.831833
C 4.194431 0.114911 2.217826
H 5.269060 -0.007674 2.044112
H 4.066420 0.504900 3.236428
H 3.729670 -0.877029 2.171410
C 4.656380 2.649324 0.547287
H 5.021709 3.167425 1.444179
H 5.527140 2.246903 0.015058
H 4.183641 3.392720 -0.107884
C 5.496509 -0.838846 -0.655012
H 6.139670 -1.327858 -1.399198
H 6.023575 0.055867 -0.303684
H 5.386870 -1.527503 0.190677
C 4.198957 0.447689 -3.075894
H 4.836107 -0.142620 -3.747721
H 3.276151 0.694685 -3.615899
H 4.715643 1.391483 -2.856259
C 3.107466 -2.160560 -1.865385
H 2.164730 -2.110744 -2.420050
H 3.824461 -2.728662 -2.474300
H 2.924211 -2.721287 -0.939955
H 1.223278 0.119881 -2.474805
H 0.566656 -0.232929 -1.848593

^{Ph}YSiB1
E = -2275.18420059
P 1.725272 0.788111 0.026891
C 0.187884 0.033305 0.135415
C 0.006238 -1.092291 1.065898
C -1.167981 -1.208782 1.834870
H -1.951548 -0.460399 1.717086
C -1.346126 -2.244775 2.739724
H -2.270498 -2.297881 3.313414
C -0.348903 -3.200440 2.929497
H -0.485707 -4.008234 3.645943
C 0.820496 -3.105466 2.184221
H 1.609252 -3.846084 2.307669
C 0.986693 -2.081577 1.259972
H 1.903969 -2.044187 0.673024
C 1.551816 2.584106 -0.255824
C 1.506339 3.468844 0.823284
H 1.651722 3.102049 1.836913
C 1.284715 4.823983 0.607166
H 1.254454 5.506787 1.453877
C 1.106459 5.304323 -0.685813
H 0.933080 6.365689 -0.853378
C 1.149777 4.427141 -1.764303
H 1.005866 4.797197 -2.777370

C 1.369718 3.071366 -1.553189
H 1.387449 2.384439 -2.396969
C 2.836309 0.262491 -1.324318
C 3.917046 1.035551 -1.759640
H 4.103827 2.011422 -1.312747
C 4.745808 0.562053 -2.769081
H 5.587218 1.163890 -3.107219
C 4.496066 -0.678637 -3.351229
H 5.146023 -1.046499 -4.143023
C 3.413643 -1.442069 -2.929151
H 3.215418 -2.407796 -3.389789
C 2.580575 -0.970817 -1.920018
H 1.717242 -1.548094 -1.591658
C 2.665633 0.603756 1.572594
C 1.962698 0.668317 2.781182
H 0.881766 0.793185 2.768247
C 2.637495 0.543501 3.987430
H 2.081800 0.586681 4.921812
C 4.015660 0.348046 3.999815
H 4.542462 0.244260 4.946546
C 4.716621 0.276020 2.802111
H 5.792793 0.114919 2.807234
C 4.045684 0.401869 1.589875
H 4.600951 0.329150 0.658118
Si -1.025948 0.527859 -1.348350
N -2.681460 -2.062681 -0.750541
C -4.645074 -1.341819 0.034281
C -3.904075 -2.441825 -0.210644
H -5.668694 -1.281049 0.384730
H -4.185038 -3.482176 -0.098507
B -2.622088 -0.619144 -0.809462
N -3.926788 -0.207470 -0.335943
C -4.432749 1.090790 -0.161775
C -5.211109 1.413253 0.952943
C -4.162561 2.080843 -1.110216
C -5.707363 2.701407 1.112563
H -5.408089 0.653318 1.706023
C -4.648496 3.369312 -0.935956
H -3.575064 1.825108 -1.990245
C -5.426498 3.687289 0.173245
H -6.310224 2.937357 1.987704
H -4.425354 4.128883 -1.682903
H -5.812379 4.696213 0.303080
C -1.650560 -2.973195 -1.014131
C -0.762537 -2.728970 -2.064610
C -1.487296 -4.124540 -0.240688
C 0.272829 -3.616559 -2.325953
H -0.911762 -1.855930 -2.700152
C -0.448040 -5.004869 -0.507617
H -2.149353 -4.301187 0.602857
C 0.441897 -4.756373 -1.546985
H 0.942094 -3.421430 -3.161937
H -0.323327 -5.886423 0.118395
H 1.254495 -5.449470 -1.754169
H -0.936284 1.227014 0.246057
H -1.558566 1.776990 -0.242117

/
E = -1309.00443033
C 1.670449 2.178436 -0.249992

C -0.460117 1.035130 -0.270476
C -1.109181 2.266661 -0.307785
C -0.370667 3.450142 -0.282471
C 1.014708 3.410451 -0.235588
H 2.760415 2.160777 -0.260511
H -2.194428 2.305376 -0.348778
H -0.891859 4.405335 -0.303082
H 1.593053 4.331937 -0.212038
N -1.085155 -0.219321 -0.243922
Si -0.023409 -1.659821 -0.457054
C -2.466383 -0.368827 -0.033954
C -3.129610 0.295960 1.006774
C -3.197655 -1.254231 -0.833072
C -4.483022 0.085510 1.227505
H -2.565941 0.969176 1.648821
C -4.549276 -1.475778 -0.594084
H -2.691464 -1.762518 -1.652182
C -5.202378 -0.802669 0.431180
H -4.979294 0.610291 2.042353
H -5.097207 -2.172225 -1.226471
H -6.262546 -0.968118 0.611523
C 0.957718 0.980057 -0.284402
C 1.484638 -0.390964 -0.358367
P 3.032203 -0.801510 0.224258
C 4.437569 -0.262319 -0.803840
H 4.436196 0.828802 -0.891651
H 5.391637 -0.589184 -0.373793
H 4.318892 -0.687301 -1.806224
C 3.407658 -0.138951 1.876589
H 4.443712 -0.338239 2.172616
H 3.229122 0.941986 1.864385
H 2.718376 -0.591622 2.596988
C 3.203788 -2.597173 0.332227
H 4.202006 -2.862970 0.695987
H 2.438717 -2.992069 1.009025
H 3.044610 -3.038141 -0.657600
H 0.280205 -1.370065 -2.212160
H 1.017134 -0.854429 -1.676327

II
E = -1858.20497995
C 0.119960 3.177921 -0.025447
C -0.014282 1.795259 -0.008039
C -1.161882 1.143666 -0.481625
C -2.208009 1.951122 -0.949852
C -2.087891 3.332290 -0.953997
C -0.923592 3.955538 -0.504823
H 1.041263 3.612883 0.356249
H -3.114106 1.471710 -1.313531
H -2.914088 3.935228 -1.326762
H -0.837114 5.039527 -0.520655
N -1.244219 -0.253469 -0.516190
S 1.224782 0.816132 0.778209
O 2.417010 1.653421 1.033822
O 0.619025 0.183424 1.956510
C 1.593988 -0.359636 -0.433438
C -2.472378 -0.830922 -0.106846
C -2.977673 -0.520084 1.160325
C -3.171313 -1.720934 -0.923727
C -4.171557 -1.079843 1.590519

H -2.398350 0.144540 1.798553
C -4.357336 -2.295066 -0.476355
H -2.780635 -1.954408 -1.912599
C -4.865858 -1.972294 0.776141
H -4.555199 -0.832211 2.578733
H -4.893406 -2.989199 -1.121177
H -5.798262 -2.415794 1.119608
P 3.150835 -1.052572 -0.158116
C 4.504625 0.029263 -0.670047
H 5.479182 -0.397929 -0.408278
H 4.442145 0.189519 -1.751427
H 4.347587 0.982982 -0.154873
C 3.295243 -2.585375 -1.100723
H 3.138022 -2.379065 -2.164879
H 4.289601 -3.021250 -0.955900
H 2.525241 -3.289129 -0.766823
C 3.446617 -1.448873 1.583502
H 3.379460 -0.510772 2.145782
H 2.650024 -2.113613 1.932644
H 4.429373 -1.911496 1.728915
Si 0.029561 -1.353999 -1.158239
H 0.367215 -0.227947 -2.496786
H 1.058435 0.000213 -1.793793

III
E = -2620.28368690
C 0.363720 1.595953 0.533262
C -0.450438 1.589143 -0.601218
C -0.845132 2.799170 -1.170356
C -0.457260 4.008206 -0.609236
C 0.336610 4.015418 0.530928
C 0.744574 2.814482 1.095696
H -1.477207 2.761943 -2.054088
H -0.777723 4.943132 -1.064798
H 0.642998 4.955872 0.984563
H 1.380058 2.795961 1.977431
S 0.955879 0.152080 1.470951
S -1.024129 0.106484 -1.482769
O 2.185594 0.669939 2.107338
O -0.159013 -0.240189 2.347671
O 0.156678 -0.339675 -2.262943
O -2.207778 0.603067 -2.207702
C -1.389182 -1.105401 -0.395737
P -2.848988 -0.967606 0.475016
P 2.896990 -0.906880 -0.367147
C -2.834278 -2.123639 1.855088
H -3.773779 -2.057182 2.413934
H -2.698504 -3.143239 1.479595
H -1.984574 -1.852075 2.490782
C -3.114684 0.680969 1.173399
H -3.139034 1.401326 0.347829
H -4.059919 0.721284 1.726181
H -2.267802 0.899589 1.833614
C -4.317787 -1.303024 -0.532366
H -4.277841 -2.340418 -0.880963
H -5.249075 -1.124788 0.017495
H -4.248918 -0.639965 -1.402559
C 3.011938 -2.012913 -1.783709
H 3.967466 -1.846748 -2.293506
H 2.928770 -3.054967 -1.462012

H 2.172584 -1.777854 -2.449936
C 3.103352 0.759914 -1.042397
H 3.082347 1.481395 -0.219832
H 4.055556 0.833102 -1.579630
H 2.262503 0.938947 -1.723002
C 4.325202 -1.207542 0.700935
H 5.267690 -0.999792 0.181746
H 4.208419 -0.551803 1.570121
H 4.303327 -2.250367 1.034555
H -0.099695 -2.838174 1.196029
C 1.375034 -1.139488 0.433445
H 0.662422 -2.164625 1.231361
Si 0.041289 -2.348780 -0.413227

IV
E = -1828.25649934
C 0.669641 3.177787 0.495705
C 0.190413 1.877994 0.433601
C -0.981498 1.464044 -0.210250
C -1.726355 2.476315 -0.827239
C -1.294600 3.795460 -0.765736
C -0.109874 4.153796 -0.109657
H 1.598180 3.416492 1.010443
H -2.657245 2.229523 -1.336234
H -1.895411 4.575913 -1.230355
H 0.195473 5.197195 -0.069769
B -1.161299 -0.120283 -0.079370
S 0.997193 0.408274 0.930113
O 1.929128 0.483203 2.057296
O -0.296047 -0.419843 1.201993
C -2.604706 -0.745623 0.050257
C -3.066657 -1.309140 1.245331
C -3.487980 -0.730317 -1.038209
C -4.352854 -1.828868 1.351827
H -2.400478 -1.335846 2.106671
C -4.778152 -1.238261 -0.937432
H -3.153020 -0.320119 -1.993088
C -5.216461 -1.792238 0.261803
H -4.685889 -2.262909 2.293934
H -5.442786 -1.210485 -1.800006
H -6.223902 -2.196938 0.344070
C 1.641348 -0.281645 -0.490754
H 1.097385 0.382450 -1.717034
P 3.156681 -1.076414 -0.288152
C 4.487394 0.016259 0.265175
H 4.619796 0.812199 -0.474943
H 4.184558 0.457243 1.220114
H 5.423579 -0.540103 0.385815
C 3.648365 -1.759987 -1.880786
H 4.593766 -2.304627 -1.781797
H 2.853558 -2.427622 -2.231897
H 3.762307 -0.944218 -2.602294
C 3.096449 -2.445460 0.895381
H 4.064584 -2.949712 0.990874
H 2.793835 -2.025343 1.861314
H 2.333820 -3.158194 0.562548
Si 0.091789 -1.072875 -1.554603
H 0.402559 0.353001 -2.445003

V
E = -1279.04316670
C -1.780857 2.109305 0.036896
C 0.433767 1.086488 0.167727
C 0.974161 2.378359 0.326863
C 0.177327 3.506520 0.342745
C -1.208363 3.355860 0.192323
H -2.863201 2.048881 -0.067776
H 2.050286 2.478263 0.465912
H 0.609374 4.495557 0.480169
H -1.849722 4.236836 0.205486
B 1.221964 -0.227199 0.157437
C 2.764388 -0.379145 0.031514
C 3.538185 0.481533 -0.768615
C 3.442728 -1.421220 0.688360
C 4.911579 0.316329 -0.898709
H 3.044627 1.283403 -1.316381
C 4.819208 -1.576979 0.583917
H 2.870535 -2.120043 1.298796
C 5.558537 -0.708583 -0.213920
H 5.483067 0.990715 -1.534830
H 5.318025 -2.385590 1.116057
H 6.635945 -0.834868 -0.308121
C -0.985763 0.935930 0.022543
P -3.144959 -0.795045 -0.120821
C -4.076626 -0.290284 1.363617
H -3.956064 0.786037 1.522776
H -5.142222 -0.531469 1.275693
H -3.648944 -0.813200 2.225807
C -4.097134 -0.115207 -1.518590
H -5.153921 -0.397342 -1.447811
H -4.013634 0.975107 -1.543582
H -3.671481 -0.511391 -2.446613
C -3.385940 -2.581562 -0.238254
H -4.456665 -2.811869 -0.268487
H -2.899896 -2.956173 -1.145146
H -2.914855 -3.066902 0.622449
Si -0.110653 -1.767737 0.327289
H 0.089943 -1.878921 -1.419825
C -1.473606 -0.415937 -0.116205
H -0.587034 -1.265490 -1.284756

Pathway C

IV
E = -1828.25649934
C -0.841344 2.986337 -0.224582
C -0.261507 1.730304 -0.278490
C 0.939797 1.368563 0.336800
C 1.590694 2.367030 1.065312
C 1.046461 3.647004 1.131767
C -0.155497 3.961504 0.492427
H -1.776134 3.202362 -0.738765
H 2.533743 2.146780 1.564732
H 1.571452 4.424609 1.684253
H -0.550981 4.973702 0.547651
B 1.252024 -0.187732 0.052949
S -0.931617 0.258385 -0.984976
O -1.793001 0.504162 -2.143326
O 0.462136 -0.405748 -1.264007

C -1.566438 -0.700240 0.223802
C 2.754451 -0.679083 -0.028101
C 3.716858 0.189559 -0.564491
C 3.193713 -1.941698 0.385426
C 5.050753 -0.182943 -0.686533
H 3.410568 1.180666 -0.899308
C 4.526633 -2.323572 0.270938
H 2.470980 -2.639637 0.807253
C 5.460639 -1.444153 -0.265801
H 5.773845 0.512375 -1.110642
H 4.837955 -3.312749 0.603575
H 6.504931 -1.739711 -0.354938
P -3.252040 -0.955274 0.194416
C -4.224253 0.573882 0.140070
H -5.299926 0.364971 0.137409
H -3.962607 1.194224 1.003655
H -3.951498 1.102912 -0.780252
C -3.716295 -1.848961 1.689683
H -3.464045 -1.239204 2.563122
H -4.787491 -2.076659 1.682659
H -3.137259 -2.777801 1.739166
C -3.836470 -1.927711 -1.218515
H -3.500583 -1.405585 -2.121448
H -3.361392 -2.913907 -1.185686
H -4.927253 -2.034327 -1.223063
Si -0.185596 -1.611746 1.213893
H -0.201030 -0.538730 2.451807
H 0.419164 -0.168481 1.653343

V
E = -1279.05823590
C -1.777187 2.110375 0.060327
C 0.438630 1.099952 0.112324
C 0.979785 2.386869 0.266660
C 0.180499 3.518263 0.312776
C -1.202502 3.364358 0.196146
H -2.862195 2.047521 -0.009025
H 2.058883 2.491801 0.370766
H 0.616383 4.506707 0.440999
H -1.847281 4.242443 0.226980
C 2.773322 -0.377493 -0.017575
C 3.602365 0.524064 -0.707938
C 3.397941 -1.479023 0.593129
C 4.979404 0.343699 -0.772320
H 3.154204 1.368900 -1.228379
C 4.775458 -1.648511 0.561991
H 2.783611 -2.219786 1.105731
C 5.571597 -0.735966 -0.125321
H 5.594995 1.050161 -1.327051
H 5.231240 -2.503275 1.059167
H 6.650919 -0.873563 -0.165917
C -0.985692 0.942238 0.041263
C -1.482216 -0.422377 -0.032958
P -3.148599 -0.794897 -0.094719
C -4.127005 -0.246609 1.344378
H -4.012248 0.832966 1.482485
H -5.189233 -0.488716 1.224480
H -3.731661 -0.747450 2.234496
C -4.054283 -0.138155 -1.535695
H -5.119989 -0.390091 -1.489349

H -3.939061 0.948748 -1.587476
H -3.609612 -0.570811 -2.438206
C -3.421368 -2.580065 -0.171586
H -4.496934 -2.784007 -0.214892
H -2.923043 -2.987461 -1.056785
H -2.983164 -3.055222 0.711484
Si -0.192115 -1.737858 -0.167118
H 0.461380 -1.396792 1.718507
H 0.057003 -2.095458 1.757377
B 1.226382 -0.218574 0.038287

4.2.4 H_2 activated products

Pathway A

$^{Tos}Y_2Si$
E = -4084.20221490
C -4.628741 -2.209900 0.351861
H -4.557965 -2.131465 1.434037
C -3.531806 -1.862194 -0.418824
C 3.531643 1.862444 -0.418385
O -0.999278 -2.311456 0.055316
C 3.578400 1.941199 -1.806934
H 2.699939 1.669850 -2.390739
C -2.515136 3.343945 2.325617
H -3.388029 3.772491 1.833598
C -5.797143 -2.623922 -0.280045
H -6.665844 -2.888924 0.321602
C 1.293097 -5.045984 -1.790770
H 0.869594 -6.013793 -1.530740
C 1.514684 -4.104438 -0.792021
H 1.241190 -4.327338 0.235460
C -2.306485 2.546015 -2.456810
H -2.639774 1.543492 -2.720028
C -2.044965 2.857833 -1.118646
C -4.975884 1.326430 -0.779533
H -4.589076 1.554025 -1.768595
C -6.324859 1.034403 -0.628331
H -6.976107 1.032873 -1.500070
C 1.591494 -4.747142 -3.114623
H 1.414847 -5.487134 -3.893064
C 1.558170 -0.147742 -0.385405
O 2.391327 1.083617 1.789225
S 2.028834 1.302810 0.373737
P 2.328437 -1.586116 0.139988
C 4.750441 2.349474 -2.422181
H 4.794253 2.407443 -3.509365
C 4.645967 -1.025561 1.590574
H 3.984133 -0.985399 2.451318
C 0.730419 -1.721648 2.381936
H 0.202795 -0.902944 1.911257
C -5.998427 0.739949 1.737743
H -6.392099 0.501840 2.723937
C -4.645767 1.024909 1.590894
H -3.983828 0.984529 2.451547
C 2.306126 -2.545432 -2.457274
H 2.639119 -1.542771 -2.720343
C -1.829350 2.274419 1.740498
C -3.578756 -1.940607 -1.807341
H -2.700393 -1.669112 -2.391224

C -1.514336 4.104471 -0.791378
H -1.240645 4.327123 0.236103
C -4.127983 1.334812 0.330661
C 2.082202 -3.853991 3.542066
H 2.613497 -4.686062 4.000208
C 1.829541 -2.274820 1.740165
C 2.515429 -3.344428 2.325019
H 3.388301 -3.772830 1.832837
C 2.044912 -2.857588 -1.119130
C 2.091764 -3.491967 -3.448843
H 2.297562 -3.242897 -4.487906
C -7.156467 -3.091880 -2.349908
H -6.968694 -3.782574 -3.180493
H -7.669919 -2.215737 -2.769910
H -7.848214 -3.576022 -1.652621
C 0.967517 -3.302707 4.173567
H 0.627928 -3.712508 5.123296
C 5.881540 2.691063 -1.669578
C -6.839879 0.747111 0.631968
H -7.897948 0.520098 0.749232
C 6.324824 -1.034548 -0.628828
H 6.975980 -1.032787 -1.500634
C -1.558223 0.147859 -0.385590
P -2.328434 1.586099 0.140225
O -2.391441 -1.083770 1.788847
S -2.028923 -1.302796 0.373334
O 0.999178 2.311490 0.055798
C -5.881847 -2.690619 -1.669883
C -4.750894 -2.348841 -2.422533
H -4.794835 -2.406650 -3.509716
C -2.092036 3.492688 -3.448227
H -2.298085 3.243882 -4.487304
C -1.591357 4.747657 -3.113842
H -1.414639 5.487757 -3.892165
C -1.292654 5.046153 -1.789980
H -0.868846 6.013795 -1.529830
C 4.975798 -1.326344 -0.779989
H 4.588858 -1.553567 -1.769083
C 0.289541 -2.238217 3.593014
H -0.596529 -1.810800 4.056662
C 4.128014 -1.334987 0.330299
C 6.840017 -0.747752 0.631514
H 7.898127 -0.520912 0.748740
C 5.797058 2.623961 -0.279794
H 6.665922 2.888627 0.321758
C 5.998685 -0.740839 1.737379
H 6.392494 -0.503111 2.723610
C -0.966987 3.301800 4.173963
H -0.627240 3.711419 5.123714
C -2.081738 3.853251 3.542713
H -2.612922 4.685275 4.001071
C 4.628684 2.209849 0.352210
H 4.558092 2.131089 1.434372
C 7.155671 3.093305 -2.349942
H 7.852996 3.564927 -1.649669
H 6.968355 3.795464 -3.171035
H 7.662204 2.219659 -2.783230
C -0.289127 2.237386 3.593130
H 0.597010 1.809878 4.056574
C -0.730199 1.721044 2.382031

H -0.202692 0.902401 1.911109
Si 0.000040 0.000142 -1.376763
H 0.127707 1.189255 -2.257411
H -0.127582 -1.188877 -2.257543

^{Tos}YSiCl
E = -2648.19894660
C -3.167105 0.803615 -1.199231
H -2.784415 0.856021 -2.215702
C -2.366366 1.238438 -0.155892
O -0.756599 3.310317 -0.116141
C 2.518158 -1.635093 -1.918191
H 2.071580 -2.584396 -1.623330
C -4.429028 0.290134 -0.916801
H -5.059274 -0.059699 -1.733501
C 0.890987 -1.255441 2.672877
H -0.014275 -0.654365 2.742886
C 1.580226 -1.321495 1.456564
C -1.377876 -2.075865 0.263183
H -1.215651 -2.020456 1.336443
C -2.473053 -2.774173 -0.226591
H -3.159325 -3.255614 0.466844
C -1.818153 -2.225798 -2.479060
H -1.993915 -2.273754 -3.551761
C -0.724647 -1.517143 -1.995120
H -0.066856 -0.988989 -2.680187
C 2.122132 -0.446917 -1.293436
C -2.815963 1.186099 1.159948
H -2.174137 1.543244 1.963728
C 2.774411 -2.038738 1.381817
H 3.339327 -2.069982 0.454177
C -0.492029 -1.453485 -0.619566
C -6.243651 -0.380221 0.700079
H -6.830894 0.287178 1.342596
H -6.147677 -1.335795 1.233181
H -6.818397 -0.564442 -0.213319
C -2.689670 -2.856222 -1.598767
H -3.547379 -3.406018 -1.981691
C 0.350332 1.097649 0.549972
P 0.881294 -0.449277 0.025921
O -0.467699 1.531918 -1.910894
S -0.742395 1.899112 -0.511435
C -4.897019 0.202975 0.393352
C -4.071031 0.664249 1.426706
H -4.424484 0.615407 2.456159
C 1.377192 -1.922360 3.788434
H 0.836489 -1.863630 4.730708
C 2.561905 -2.648210 3.704853
H 2.946878 -3.164536 4.582032
C 3.260818 -2.699429 2.505020
H 4.196945 -3.250044 2.440567
C 4.018192 -0.382306 -3.323298
H 4.761053 -0.356161 -4.118374
C 3.469123 -1.600685 -2.928365
H 3.778038 -2.523767 -3.414751
C 3.611548 0.798715 -2.714464
H 4.029424 1.751575 -3.031666
C 2.659079 0.772434 -1.700935
H 2.322596 1.694875 -1.233017
Cl 2.580271 3.481017 0.873018

Si 1.183450 2.194781 1.745481
H 1.974776 1.369534 2.690583
H 0.228885 3.040731 2.491259

^{Tos}YSiHMDS
E = -3062.01021364
S 0.749044 -1.563345 0.736758
P 0.614615 1.206313 0.035481
Si -4.005609 0.209009 -0.664935
Si -3.354364 -2.652248 0.204700
O -0.063473 -2.780258 0.590579
O 0.941276 -1.005977 2.088847
N -2.868659 -1.138876 -0.594534
C 0.114047 -0.390243 -0.336572
C 2.388729 -1.976351 0.154845
C 2.586087 -2.267408 -1.191376
H 1.741602 -2.240300 -1.878364
C 3.861697 -2.571013 -1.638352
H 4.021966 -2.794137 -2.692653
C 4.949682 -2.598420 -0.756509
C 4.716733 -2.325684 0.590838
H 5.550044 -2.346926 1.292123
C 3.442491 -2.013347 1.053535
H 3.254388 -1.777289 2.098184
C 6.330559 -2.893490 -1.260312
H 6.335497 -3.760127 -1.932068
H 7.023253 -3.096147 -0.436963
H 6.731248 -2.043415 -1.829607
C 2.394484 1.304606 0.419275
C 3.324375 1.263982 -0.622683
H 2.989433 1.235634 -1.656600
C 4.684345 1.259544 -0.344263
H 5.401796 1.226101 -1.161489
C 5.124860 1.298252 0.975165
H 6.191287 1.297829 1.192799
C 4.202487 1.324166 2.014676
H 4.542873 1.336430 3.048128
C 2.839281 1.317938 1.742676
H 2.119302 1.291507 2.555866
C -0.205220 1.950207 1.475394
C 0.211438 3.176527 2.002659
H 1.042380 3.713949 1.546656
C -0.416998 3.698369 3.125550
H -0.093102 4.653375 3.534304
C -1.450462 2.990212 3.733869
H -1.937195 3.395098 4.619136
C -1.851818 1.761408 3.222150
H -2.646447 1.198834 3.708055
C -1.231448 1.236711 2.093798
H -1.528043 0.267413 1.693148
C 0.340981 2.272449 -1.408538
C 0.634589 1.756711 -2.674551
H 0.973762 0.726511 -2.765830
C 0.463559 2.543123 -3.806323
H 0.688359 2.131273 -4.787882
C -0.008533 3.845931 -3.684666
H -0.149909 4.459108 -4.572565
C -0.311632 4.359708 -2.428722
H -0.695897 5.372733 -2.330233
C -0.138548 3.577870 -1.293066

H -0.407401 3.980647 -0.320398
C -4.867679 0.473886 0.987274
H -5.598926 1.286700 0.882588
H -4.138454 0.782846 1.745860
H -5.398207 -0.403630 1.369393
C -3.137102 1.837269 -1.018938
H -3.884422 2.640116 -0.953111
H -2.672906 1.890970 -2.009681
H -2.369050 2.051897 -0.267897
C -5.269103 -0.048394 -2.030066
H -5.900724 -0.926606 -1.859951
H -4.753692 -0.186397 -2.989569
H -5.923772 0.828537 -2.121989
C -2.987826 -2.525519 2.039592
H -3.521166 -1.677110 2.487560
H -1.911723 -2.385578 2.191812
H -3.292039 -3.435321 2.573588
C -2.557414 -4.152724 -0.581082
H -2.991561 -5.052157 -0.123114
H -1.474731 -4.177802 -0.438357
H -2.779697 -4.184746 -1.655692
C -5.202766 -2.952350 -0.041753
H -5.416350 -3.154528 -1.099253
H -5.867879 -2.148556 0.289786
H -5.473323 -3.854696 0.523607
Si -1.309935 -0.976860 -1.374237
H -0.916812 -2.269541 -1.984991
H -1.474037 0.006124 -2.476909

$^{Tos}YSiC_6F_5$
E = -2916.53728533
C 3.301696 -2.034031 -1.316865
H 2.927098 -1.813861 -2.313422
C 2.418206 -2.028290 -0.249876
O -0.096040 -2.822754 -0.168851
C -0.253184 3.166167 -1.736634
H 0.562392 3.776101 -1.348357
C 4.648029 -2.295006 -1.081332
H 5.347654 -2.290422 -1.916197
C 1.123195 1.930885 2.673236
H 1.728664 1.026902 2.697781
C 0.431759 2.271721 1.504883
C 3.408837 1.342243 0.227939
H 3.280403 1.341926 1.306142
C 4.691888 1.342705 -0.301352
H 5.550019 1.342377 0.367383
C 3.776212 1.324905 -2.527918
H 3.915586 1.305843 -3.606845
C 2.488557 1.319699 -2.003582
H 1.629925 1.265958 -2.667382
C -0.451089 1.868548 -1.254677
C 2.857355 -2.296603 1.043404
H 2.147081 -2.292259 1.868580
C -0.434031 3.365394 1.515304
H -1.027140 3.603185 0.636751
C 2.298297 1.343719 -0.620133
C 6.573421 -2.812374 0.462958
H 6.723054 -3.809891 0.895712
H 6.989379 -2.088949 1.176030
H 7.161359 -2.754464 -0.458676

C 4.877260 1.339942 -1.680545
H 5.883979 1.339510 -2.094135
C 0.277925 -0.403362 0.568217
P 0.611477 1.197253 0.053698
O 0.646526 -1.147412 -1.928472
S 0.696795 -1.643867 -0.541544
C 5.120632 -2.546623 0.205445
C 4.201584 -2.547380 1.263196
H 4.550796 -2.750398 2.275071
C 0.992685 2.707383 3.815175
H 1.534453 2.434673 4.718379
C 0.151650 3.816532 3.810582
H 0.043050 4.421198 4.708828
C -0.568797 4.134843 2.666265
H -1.251550 4.981868 2.667647
C -2.133858 2.892581 -3.215352
H -2.798111 3.296210 -3.977111
C -1.096779 3.676346 -2.714361
H -0.946036 4.687391 -3.087294
C -2.314783 1.593477 -2.754868
H -3.115619 0.975747 -3.155869
C -1.466800 1.076018 -1.782747
H -1.570564 0.049723 -1.441092
Si -0.893562 -0.943271 1.878004
H -1.036091 0.122662 2.901117
H -0.472797 -2.198200 2.537378
C -2.616047 -1.176838 1.128656
C -4.369930 -2.505271 0.102253
C -4.422334 -0.103794 -0.107743
C -4.977755 -1.350258 -0.375718
C -3.267872 -0.045832 0.656671
C -3.200003 -2.405598 0.848865
F -2.652196 -3.527640 1.281237
F -2.738090 1.159189 0.893352
F -4.913849 -3.682605 -0.156113
F -6.083447 -1.436356 -1.093345
F -4.986577 0.994775 -0.587432

^{Tos}YSiPy
E = -2435.68222444
C 3.489364 -1.411766 -0.961288
H 3.121110 -1.511410 -1.979411
C 2.604796 -1.578677 0.092417
O 0.629602 -3.315955 0.207610
C -1.725076 1.885086 -2.157045
H -1.202221 2.801326 -1.883303
C 4.816369 -1.094110 -0.690599
H 5.511704 -0.952527 -1.517169
C -0.184937 1.899832 2.396892
H 0.666354 1.241918 2.562638
C -0.890477 1.824073 1.191718
C 2.219476 1.905326 0.039946
H 2.046446 2.001692 1.107636
C 3.425614 2.339017 -0.493239
H 4.181076 2.766984 0.162446
C 2.700524 1.661983 -2.686514
H 2.889237 1.554362 -3.752747
C 1.493442 1.220377 -2.157077
H 0.757932 0.743525 -2.798798
C -1.475723 0.700323 -1.457736

C 3.033528 -1.448948 1.409448
H 2.323286 -1.583029 2.223528
C -2.045395 2.587843 1.025462
H -2.639288 2.491309 0.121384
C 1.240619 1.353374 -0.790092
C 6.689760 -0.554510 0.908037
H 7.128133 -1.197772 1.680589
H 6.752610 0.478475 1.276815
H 7.314557 -0.625144 0.011682
C 3.665627 2.223143 -1.858805
H 4.611560 2.563236 -2.276225
C -0.037117 -0.850502 0.676769
P -0.294822 0.670839 -0.078055
O 0.727467 -1.650624 -1.695515
S 0.888874 -1.952563 -0.259424
C 5.269127 -0.936631 0.618484
C 4.356623 -1.126191 1.664170
H 4.693754 -1.017132 2.694542
C -0.599698 2.767464 3.397896
H -0.044909 2.817823 4.332458
C -1.734118 3.551138 3.213122
H -2.063562 4.226622 4.000303
C -2.460116 3.451327 2.032263
H -3.365123 4.039709 1.895921
C -3.280369 0.714443 -3.573000
H -3.989534 0.719811 -4.398803
C -2.631418 1.892006 -3.209235
H -2.827273 2.815263 -3.750916
C -3.013495 -0.469021 -2.893904
H -3.507289 -1.393215 -3.186367
C -2.107562 -0.480445 -1.839784
H -1.872447 -1.404850 -1.318176
C -2.886822 -1.680777 1.277282
N -3.465752 -0.485809 1.069064
C -4.721047 -2.812978 0.212788
C -4.642776 -0.440141 0.461369
C -5.314592 -1.573394 0.011008
H -5.209175 -3.724112 -0.130320
H -5.075819 0.551571 0.309444
H -6.276226 -1.480198 -0.489182
C -3.490620 -2.868681 0.858215
H -2.988484 -3.820315 1.023049
Si -1.143885 -1.520266 1.992088
H -1.261223 -0.583693 3.140565
H -0.637792 -2.824682 2.476774

^{Tos}YSiPyr
E = -2451.73835013
C -3.504393 -1.563999 0.650182
H -3.139091 -1.916081 1.611894
C -2.624529 -1.496787 -0.418415
O -0.743104 -3.246987 -0.957470
C 1.799154 1.312394 2.454070
H 1.288218 2.272824 2.391712
C -4.821493 -1.153055 0.473050
H -5.512925 -1.196249 1.313771
C 0.124826 2.238010 -1.981870
H -0.692717 1.578371 -2.268598
C 0.844387 1.971053 -0.812833
C -2.188985 1.837317 0.414408

H -2.030186 2.170839 -0.607018
C -3.371807 2.168340 1.061334
H -4.125557 2.753534 0.538704
C -2.628660 0.991593 3.026359
H -2.801750 0.649938 4.044829
C -1.444153 0.653552 2.382695
H -0.708404 0.029781 2.882662
C 1.510993 0.303147 1.530420
C -3.049341 -1.041395 -1.662327
H -2.343446 -0.998045 -2.490059
C 1.950567 2.758121 -0.494307
H 2.554899 2.525493 0.377547
C -1.213911 1.086105 1.074962
C -6.678645 -0.192153 -0.935943
H -7.133071 -0.613909 -1.840569
H -6.712838 0.901112 -1.039436
H -7.306647 -0.463961 -0.081237
C -3.591187 1.749647 2.369918
H -4.519306 2.008929 2.875933
C 0.046975 -0.770001 -0.849779
P 0.299514 0.566118 0.201616
O -0.747795 -2.063947 1.281098
S -0.927050 -2.020706 -0.183952
C -5.269466 -0.671818 -0.756377
C -4.362426 -0.628229 -1.822635
H -4.695877 -0.264441 -2.794091
C 0.480083 3.306018 -2.794592
H -0.084342 3.503777 -3.703416
C 1.566902 4.105196 -2.456473
H 1.850176 4.938761 -3.096283
C 2.304867 3.823941 -1.312288
H 3.172004 4.430454 -1.059042
C 3.354318 -0.153532 3.562905
H 4.078292 -0.331588 4.355994
C 2.725864 1.085402 3.463413
H 2.951894 1.872924 4.179599
C 3.043582 -1.168219 2.664547
H 3.514756 -2.144694 2.755113
C 2.117768 -0.945572 1.651487
H 1.841470 -1.743006 0.965662
Si 1.186849 -1.143864 -2.252923
H 1.230565 -0.031110 -3.240514
H 0.795659 -2.398257 -2.924219
C 2.951708 -1.265477 -1.550762
N 3.499381 -2.459543 -1.302715
N 3.510526 -0.091677 -1.219951
C 4.675289 -2.475238 -0.687194
C 4.684618 -0.118681 -0.605712
C 5.330618 -1.310929 -0.305167
H 5.112853 -3.456226 -0.486500
H 5.124864 0.844249 -0.334653
H 6.291405 -1.332284 0.203300

^{Tos}YSiB2
E = -2594.69234496
C -1.441389 2.986678 -1.290577
H -1.088418 2.668766 -2.268637
C -0.861493 2.441401 -0.157218
O 1.657375 1.869644 0.234787
C -1.170587 -3.247892 -1.897570

H -2.250459 -3.238063 -1.751084
C -2.473635 3.908679 -1.146827
H -2.938173 4.334514 -2.035337
C -1.995341 -1.490197 2.620198
H -1.788963 -0.422645 2.685628
C -1.707685 -2.175193 1.434103
C -3.480826 0.135089 -0.043661
H -3.487904 0.023915 1.038180
C -4.502372 0.836300 -0.668919
H -5.302871 1.269558 -0.072935
C -3.465392 0.443689 -2.808298
H -3.451264 0.573090 -3.888578
C -2.434764 -0.253078 -2.187534
H -1.604921 -0.641267 -2.772302
C -0.354624 -2.371819 -1.173516
C -1.287500 2.812923 1.114787
H -0.816200 2.373604 1.992881
C -1.924626 -3.551212 1.363649
H -1.673892 -4.101274 0.460637
C -2.448748 -0.423254 -0.801866
C -4.065786 5.257919 0.267960
H -3.795308 6.082174 0.939385
H -4.949130 4.769162 0.700568
H -4.359996 5.687699 -0.694994
C -4.499137 0.984635 -2.052947
H -5.301323 1.533816 -2.542336
C 0.054801 -0.079191 0.682311
P -1.045742 -1.216876 0.041359
O 0.438483 0.848798 -1.752821
S 0.447753 1.236847 -0.333190
C -2.933943 4.286980 0.113287
C -2.320168 3.727297 1.241653
H -2.660444 4.017820 2.235056
C -2.513801 -2.173252 3.712027
H -2.730818 -1.634213 4.631808
C -2.739455 -3.543997 3.633237
H -3.139091 -4.080303 4.491693
C -2.440351 -4.230497 2.462111
H -2.600000 -5.305110 2.402684
C 0.773063 -4.106219 -3.029200
H 1.215303 -4.785428 -3.755742
C -0.604495 -4.117279 -2.819929
H -1.238184 -4.799280 -3.383392
C 1.580172 -3.221460 -2.324211
H 2.653885 -3.199753 -2.498692
C 1.020858 -2.347546 -1.397429
H 1.646082 -1.632991 -0.865418
C 4.665438 0.147552 -0.085615
C 4.981677 1.012804 0.959155
C 5.511163 -0.039251 -1.160350
C 6.161814 1.728747 0.979122
C 6.706860 0.683204 -1.151064
H 5.248219 -0.712423 -1.972068
C 7.025293 1.547215 -0.104011
H 6.395493 2.401392 1.799899
H 7.401997 0.570646 -1.980431
H 7.964837 2.095159 -0.131534
B 3.006647 0.155375 1.352405
O 3.458527 -0.427214 0.169135
O 3.975076 0.993798 1.874862

Si 1.216568 -0.328350 2.103793
H 1.131567 -1.756908 2.527076
H 0.851064 0.493093 3.298663

^{Tos}YSiB1
E = -2863.86877333
C 4.057170 1.037451 -1.140930
H 4.020942 1.922350 -0.510093
C 2.868398 0.496804 -1.602584
O 0.711423 1.693054 -2.439777
C 0.206098 -0.365040 3.692599
H 1.171143 -0.843523 3.852831
C 5.258213 0.421524 -1.475825
H 6.194366 0.838251 -1.105686
C 0.618930 -3.133339 -0.397665
H 0.995505 -2.538298 -1.227756
C 0.319224 -2.498355 0.810668
C 3.324025 -1.561424 1.066060
H 2.990785 -2.463227 0.557995
C 4.666064 -1.405627 1.387791
H 5.376519 -2.187568 1.127623
C 4.188479 0.749802 2.355667
H 4.526507 1.657090 2.852073
C 2.844888 0.604376 2.034031
H 2.138022 1.400293 2.254666
C -0.260971 -0.144553 2.392883
C 2.867437 -0.626446 -2.423217
H 1.922139 -1.008127 -2.806983
C -0.196123 -3.239789 1.873042
H -0.452900 -2.751355 2.809861
C 2.408881 -0.558854 1.393968
C 6.578519 -1.422207 -2.573738
H 6.590271 -1.813058 -3.597662
H 6.737088 -2.276603 -1.900448
H 7.433739 -0.748578 -2.454509
C 5.097632 -0.253416 2.036339
H 6.149601 -0.133775 2.289048
C 0.296631 0.038500 -0.559257
P 0.661152 -0.715348 0.931011
O 1.662313 2.280620 -0.152870
S 1.313436 1.272471 -1.163854
C 5.285751 -0.729225 -2.262994
C 4.070770 -1.237071 -2.740263
H 4.074190 -2.120984 -3.377353
C 0.418554 -4.499935 -0.534776
H 0.650685 -4.988088 -1.479059
C -0.092570 -5.238573 0.528542
H -0.258916 -6.308418 0.417876
C -0.401629 -4.607903 1.728176
H -0.812839 -5.181118 2.556587
C -1.790652 0.653394 4.580011
H -2.386307 0.966820 5.435359
C -0.558391 0.036208 4.781216
H -0.189722 -0.130439 5.791473
C -2.255666 0.875566 3.288805
H -3.214223 1.362781 3.119960
C -1.489271 0.482797 2.196877
H -1.839304 0.678207 1.186342
N -4.148191 0.536821 -0.851095
C -4.047233 2.581645 0.035749

C -4.855437 1.517923 -0.168722
H -4.283943 3.541723 0.480238
H -5.908668 1.411646 0.062862
B -2.793770 1.003043 -1.072555
C -1.755689 3.284320 -0.400580
C -1.113716 3.741550 -1.546615
C -1.403379 3.803714 0.846079
C -0.122960 4.709113 -1.446406
H -1.385342 3.321735 -2.510620
C -0.416889 4.775871 0.938327
H -1.902385 3.435156 1.739708
C 0.223972 5.232881 -0.207984
H 0.387256 5.043570 -2.346833
H -0.140936 5.170609 1.914386
H 1.004686 5.987027 -0.132617
C -4.696732 -0.718954 -1.157463
C -4.347542 -1.360968 -2.347803
C -5.586513 -1.346112 -0.281869
C -4.866932 -2.613353 -2.646212
H -3.675793 -0.859899 -3.042546
C -6.115546 -2.591420 -0.595238
H -5.842189 -0.863142 0.658645
C -5.756939 -3.233885 -1.775625
H -4.581818 -3.101640 -3.576132
H -6.805391 -3.069268 0.098093
H -6.169482 -4.211428 -2.016151
N -2.793780 2.325498 -0.502959
Si -1.278310 -0.251077 -1.503605
H -1.782194 -1.556321 -0.967598
H -1.007376 -0.479421 -2.950673

$^{Ph}Y_2Si$
E = -2907.00165557
P 2.973180 0.335191 -0.072433
P -3.021755 0.426766 -0.060505
C 1.473662 -0.045199 -0.773059
C 1.215555 -1.403660 -1.264427
C 1.764299 -2.559576 -0.683928
H 2.394493 -2.460938 0.197570
C 1.467493 -3.827172 -1.165643
H 1.901603 -4.698388 -0.677498
C 0.593444 -3.991331 -2.235344
H 0.347578 -4.985932 -2.602071
C 0.038571 -2.860768 -2.829079
H -0.637404 -2.969123 -3.677216
C 0.352430 -1.592626 -2.359118
H -0.057588 -0.709780 -2.849672
C 3.261529 -0.083864 1.695242
C 3.882613 -1.272039 2.087316
H 4.332838 -1.922803 1.339603
C 3.934648 -1.630692 3.429887
H 4.426294 -2.556840 3.721376
C 3.356817 -0.812943 4.394668
H 3.396058 -1.095214 5.445006
C 2.720505 0.364658 4.011620
H 2.257769 1.004027 4.760911
C 2.671870 0.726607 2.671688
H 2.161517 1.642265 2.373606
C 3.299294 2.127047 -0.158949
C 4.197742 2.748152 0.710949

H 4.659433 2.180535 1.516581
C 4.500622 4.095941 0.556569
H 5.197860 4.574331 1.241679
C 3.912739 4.830062 -0.468161
H 4.147928 5.886372 -0.585267
C 3.028557 4.211023 -1.345787
H 2.569947 4.779694 -2.152283
C 2.728037 2.862561 -1.198836
H 2.045233 2.367559 -1.887070
C 4.387637 -0.396629 -0.961331
C 4.214098 -0.876987 -2.260119
H 3.220340 -0.888383 -2.699646
C 5.309499 -1.337060 -2.981843
H 5.165189 -1.721137 -3.989640
C 6.581968 -1.305940 -2.421750
H 7.437131 -1.666653 -2.990415
C 6.762878 -0.805163 -1.136213
H 7.758484 -0.766115 -0.698550
C 5.670294 -0.349882 -0.408847
H 5.817184 0.047565 0.594423
C -1.400003 -0.002634 0.178517
C -1.087932 -1.268683 0.867773
C -0.156105 -1.280190 1.917924
H 0.267639 -0.333146 2.244526
C 0.219233 -2.458998 2.546900
H 0.948139 -2.425378 3.355152
C -0.344775 -3.671880 2.158603
H -0.055104 -4.598257 2.651488
C -1.282055 -3.683818 1.131235
H -1.721419 -4.625133 0.804125
C -1.637821 -2.503390 0.489171
H -2.324189 -2.540883 -0.356180
C -4.064763 0.034915 1.387823
C -4.245199 0.961818 2.416291
H -3.845796 1.968399 2.319921
C -4.943856 0.609246 3.564257
H -5.086896 1.343403 4.354805
C -5.457689 -0.676276 3.702760
H -5.999626 -0.952834 4.605238
C -5.279394 -1.605536 2.684608
H -5.676488 -2.613438 2.787243
C -4.591865 -1.252195 1.529236
H -4.467364 -1.980983 0.731951
C -3.146730 2.217158 -0.364445
C -3.910195 2.754333 -1.401532
H -4.461891 2.098700 -2.070197
C -3.953235 4.130757 -1.595964
H -4.545501 4.540169 -2.411990
C -3.236652 4.979366 -0.759915
H -3.269913 6.055771 -0.917991
C -2.470271 4.449870 0.273424
H -1.898139 5.107653 0.924567
C -2.422274 3.076112 0.469783
H -1.804453 2.655770 1.261031
C -3.970563 -0.345920 -1.430642
C -5.366920 -0.305464 -1.491497
H -5.932551 0.176258 -0.694463
C -6.031998 -0.886221 -2.564032
H -7.119093 -0.855192 -2.609896
C -5.308473 -1.514426 -3.575850

H -5.832624 -1.973780 -4.412040
C -3.920547 -1.563663 -3.512989
H -3.354874 -2.063762 -4.296945
C -3.252550 -0.980085 -2.440766
H -2.168295 -1.027607 -2.355233
Si 0.008566 1.033988 -0.414848
H 0.483969 2.068984 0.572333
H -0.453716 1.856763 -1.583978

^{Ph}YSiCl
E = -2059.61012190
P -0.476247 0.232372 -0.009140
C 0.674708 -0.485768 -1.052531
C 2.063511 -0.002976 -1.002391
C 2.407898 1.294467 -0.574020
H 1.629089 1.991542 -0.269289
C 3.726881 1.726154 -0.525878
H 3.941693 2.738117 -0.185497
C 4.763559 0.884693 -0.911509
H 5.796673 1.224191 -0.878899
C 4.451639 -0.405669 -1.329475
H 5.245953 -1.091583 -1.620237
C 3.135845 -0.843308 -1.364237
H 2.937963 -1.874889 -1.650511
C 0.244039 0.465509 1.651607
C -0.004656 1.562360 2.476361
H -0.653697 2.368059 2.143972
C 0.602454 1.644155 3.724873
H 0.409026 2.504597 4.362398
C 1.461077 0.637459 4.152560
H 1.938270 0.707970 5.128184
C 1.722384 -0.451146 3.326681
H 2.408173 -1.231849 3.648986
C 1.123912 -0.536105 2.076652
H 1.349116 -1.368884 1.409402
C -1.932640 -0.833057 0.176767
C -1.844588 -1.966565 0.988593
H -0.937943 -2.163415 1.556048
C -2.905983 -2.859600 1.050076
H -2.828362 -3.744561 1.677758
C -4.056959 -2.627778 0.303808
H -4.885864 -3.331445 0.350712
C -4.147202 -1.500926 -0.506269
H -5.043453 -1.322270 -1.096791
C -3.087902 -0.604056 -0.573363
H -3.155524 0.265870 -1.222251
C -1.150187 1.838064 -0.549161
C -0.627043 2.403070 -1.712020
H 0.172283 1.881310 -2.237465
C -1.133931 3.606792 -2.190389
H -0.718968 4.046535 -3.094956
C -2.173494 4.240057 -1.518792
H -2.573140 5.179337 -1.896423
C -2.715205 3.668107 -0.370133
H -3.541139 4.154640 0.145016
C -2.208263 2.468817 0.114041
H -2.652119 2.008870 0.996391
Cl 0.842545 -3.708578 -0.654258
Si 0.270845 -2.058176 -1.854087
H 1.026201 -2.249102 -3.115892

H -1.174723 -2.254823 -2.105059

^{Ph}YSiHMDS
E = -2473.40958015
P -1.715123 0.131013 -0.060902
C -0.068433 -0.248684 0.092298
C 0.327852 -1.589253 0.588084
C -0.224143 -2.789979 0.112797
H -0.983046 -2.757084 -0.666105
C 0.196779 -4.023393 0.598680
H -0.251208 -4.934629 0.205159
C 1.193472 -4.100108 1.565705
H 1.529087 -5.066348 1.937282
C 1.749647 -2.921403 2.057174
H 2.520273 -2.960194 2.826378
C 1.314947 -1.691120 1.583474
H 1.724971 -0.772518 1.992078
C -2.658439 -0.746349 -1.373688
C -3.981920 -1.172159 -1.254359
H -4.532541 -1.001076 -0.332537
C -4.598726 -1.835660 -2.310544
H -5.628898 -2.171494 -2.207822
C -3.902196 -2.075495 -3.490022
H -4.387928 -2.596700 -4.312823
C -2.578899 -1.661028 -3.611682
H -2.025186 -1.858238 -4.527506
C -1.956605 -1.008122 -2.555227
H -0.910135 -0.710702 -2.630516
C -1.896348 1.891071 -0.459408
C -2.324543 2.347357 -1.703699
H -2.619718 1.638786 -2.474334
C -2.363686 3.714758 -1.962884
H -2.690860 4.069412 -2.938129
C -1.981653 4.621908 -0.982411
H -2.009916 5.690009 -1.189713
C -1.564907 4.166850 0.266887
H -1.267253 4.876376 1.036356
C -1.522933 2.805791 0.529754
H -1.183363 2.441159 1.497808
C -2.704877 -0.086485 1.454823
C -2.256795 -0.900303 2.497030
H -1.310955 -1.426729 2.408976
C -3.018151 -1.028667 3.653689
H -2.662378 -1.666048 4.460643
C -4.221176 -0.343582 3.783419
H -4.811184 -0.444499 4.692446
C -4.664806 0.480757 2.753920
H -5.598400 1.030601 2.856104
C -3.909654 0.611147 1.595002
H -4.251233 1.270688 0.797977
N 2.815950 0.457929 -0.283232
Si 3.412068 1.479102 1.018160
Si 3.833404 -0.614895 -1.248000
C 1.954671 2.337084 1.839957
H 1.454078 3.040178 1.163812
H 1.205855 1.606651 2.173982
H 2.306799 2.897753 2.715772
C 4.325859 0.532614 2.368625
H 5.374125 0.346236 2.112710
H 4.313513 1.131619 3.289228

H 3.859614 -0.434195 2.592327
C 4.583748 2.769836 0.317172
H 4.961504 3.444120 1.097011
H 5.450240 2.291792 -0.159387
H 4.077130 3.375379 -0.445205
C 5.408629 -1.061747 -0.328893
H 6.003073 -1.727876 -0.968430
H 6.030729 -0.188500 -0.096373
H 5.195345 -1.594941 0.605095
C 4.326630 0.248242 -2.843383
H 4.976213 -0.386787 -3.460361
H 3.442268 0.508909 -3.437585
H 4.868314 1.177851 -2.624170
C 2.935807 -2.215789 -1.636538
H 1.961454 -2.045569 -2.109683
H 3.541295 -2.820597 -2.325200
H 2.763600 -2.801913 -0.725031
Si 1.181088 0.692971 -0.879839
H 0.844337 2.140714 -0.958994
H 1.248831 0.248677 -2.314105

$^{Ph}YSiC_6F_5$
E = -2327.93992590
P -1.773358 0.000539 -0.057667
C -0.209314 0.184804 -0.671518
C 0.696295 1.201589 -0.064769
C 1.290636 0.993578 1.190145
H 1.030164 0.095808 1.749876
C 2.225042 1.883290 1.706499
H 2.679253 1.688833 2.676925
C 2.592939 3.009777 0.975808
H 3.335790 3.701034 1.369318
C 2.005662 3.241899 -0.263342
H 2.286877 4.119570 -0.842841
C 1.063265 2.353260 -0.771403
H 0.613241 2.529010 -1.746503
C -1.972607 -0.860880 1.548961
C -3.042274 -0.603320 2.410048
H -3.776971 0.158352 2.153740
C -3.165543 -1.315039 3.598010
H -3.996817 -1.108629 4.269542
C -2.224322 -2.285134 3.930324
H -2.320250 -2.837647 4.863204
C -1.157509 -2.543992 3.075361
H -0.416911 -3.297892 3.334671
C -1.029817 -1.832314 1.887751
H -0.188525 -2.018965 1.220894
C -2.771925 -0.953378 -1.233731
C -3.639698 -1.967508 -0.826204
H -3.732161 -2.214676 0.228896
C -4.375183 -2.672957 -1.772080
H -5.044281 -3.468437 -1.450402
C -4.250050 -2.370383 -3.123698
H -4.823844 -2.928612 -3.861024
C -3.387935 -1.358268 -3.534334
H -3.285154 -1.121765 -4.591366
C -2.649788 -0.649934 -2.594486
H -1.965727 0.138102 -2.905901
C -2.597890 1.596551 0.229661
C -2.106399 2.419505 1.249120

H -1.294489 2.073027 1.886376
C -2.651989 3.680595 1.449362
H -2.259873 4.317898 2.239305
C -3.695733 4.125762 0.643795
H -4.124581 5.113075 0.804348
C -4.192576 3.307431 -0.364729
H -5.012776 3.650367 -0.992382
C -3.644379 2.046542 -0.576035
H -4.035702 1.411441 -1.368262
C 2.371217 -1.046780 -1.162436
C 3.964696 -1.501264 0.621738
C 4.412566 0.267345 -0.949126
C 4.784528 -0.477243 0.162790
Si 0.566593 -1.066677 -1.757919
H 0.598731 -0.783665 -3.222368
H 0.009434 -2.438607 -1.603330
C 3.224076 -0.040202 -1.596891
C 2.777282 -1.764240 -0.047126
F 1.982758 -2.706541 0.468490
F 2.882851 0.716867 -2.636257
F 5.185382 1.253790 -1.371157
F 5.914241 -0.206388 0.791559
F 4.316212 -2.198480 1.691628

^{Ph}YSiPy
E = -1847.08712655
P -0.468187 -0.537587 -0.054517
C -0.438522 0.698243 -1.218019
C -1.511316 1.726197 -1.162126
C -1.221623 3.055715 -0.811322
C -2.214690 4.026259 -0.768238
C -3.534706 3.695460 -1.059472
C -3.842833 2.386798 -1.416359
C -2.844421 1.421548 -1.478964
C -0.157791 -0.041575 1.681761
C -0.461179 -0.882577 2.758073
H -0.926544 -1.851154 2.578803
C -0.172937 -0.481537 4.055742
H -0.407582 -1.137869 4.891738
C 0.410393 0.763045 4.286231
H 0.630700 1.077966 5.304735
C 0.704992 1.602920 3.218991
H 1.157019 2.576869 3.396607
C 0.421453 1.203143 1.916044
H 0.658428 1.848406 1.072373
C 0.808235 -1.767728 -0.446734
C 1.747974 -2.192682 0.494180
H 1.724352 -1.795997 1.505974
C 2.730599 -3.106952 0.132201
H 3.464520 -3.427515 0.868823
C 2.781866 -3.602727 -1.166305
H 3.553956 -4.316989 -1.446425
C 1.848928 -3.181434 -2.109133
H 1.889635 -3.560410 -3.128219
C 0.866938 -2.265227 -1.753191
H 0.149219 -1.909689 -2.490385
C -2.061626 -1.418804 0.108864
C -3.087171 -0.806129 0.836142
H -2.902760 0.140612 1.341280
C -4.343541 -1.393565 0.904917

H -5.137136 -0.904892 1.466621
C -4.585589 -2.600841 0.256522
H -5.569534 -3.062513 0.313595
C -3.567311 -3.218734 -0.461209
H -3.749956 -4.166943 -0.963041
C -2.309857 -2.629463 -0.538630
H -1.519380 -3.122572 -1.099559
C 2.650571 1.179788 -1.078108
C 4.755384 0.364161 -0.237520
C 3.732191 2.364107 0.569429
C 4.790984 1.460393 0.613982
N 2.691601 2.231036 -0.241658
H 5.557680 -0.372481 -0.229976
H 5.617203 1.616010 1.304593
H 3.728194 3.234572 1.229922
H -0.189466 3.320654 -0.577854
H -1.955566 5.048673 -0.497107
H -4.314664 4.453634 -1.018434
H -4.867923 2.116317 -1.666195
H -3.088403 0.409325 -1.795124
C 3.673199 0.226759 -1.098464
H 3.601174 -0.624256 -1.775561
Si 1.093535 1.064046 -2.158067
H 0.904943 2.381559 -2.820411
H 1.452445 0.071228 -3.220207

^{Ph}YSiPyr
E = -1863.14632866
P -1.081886 -0.113557 -0.041038
C 0.460936 -0.004014 -0.742748
C 1.238848 1.236678 -0.666043
C 1.073314 2.199684 0.346216
C 1.839771 3.357670 0.384719
C 2.826918 3.587168 -0.567312
C 3.031057 2.634353 -1.562107
C 2.249386 1.489891 -1.615115
C -1.170824 -0.345924 1.771679
C -2.363168 -0.258978 2.499542
H -3.296841 -0.017661 1.991864
C -2.357420 -0.470885 3.871974
H -3.285860 -0.403829 4.436048
C -1.161749 -0.764559 4.524601
H -1.158424 -0.927772 5.600881
C 0.024391 -0.843474 3.804760
H 0.959396 -1.067846 4.314400
C 0.026363 -0.633981 2.428202
H 0.958134 -0.689418 1.859407
C -1.949661 -1.539853 -0.773534
C -2.747402 -2.405743 -0.024839
H -2.858339 -2.261153 1.046168
C -3.384576 -3.477424 -0.640773
H -3.998109 -4.151302 -0.046127
C -3.229454 -3.695792 -2.004834
H -3.725986 -4.538442 -2.482189
C -2.427954 -2.842092 -2.755569
H -2.290477 -3.014801 -3.821010
C -1.789148 -1.770813 -2.144815
H -1.138762 -1.116352 -2.723370
C -2.107160 1.365240 -0.366262
C -2.352763 2.337263 0.604997

H -1.979250 2.205540 1.617810
C -3.067740 3.485773 0.282990
H -3.247526 4.239610 1.046839
C -3.542681 3.673087 -1.009329
H -4.102054 4.572386 -1.259996
C -3.295549 2.711470 -1.984240
H -3.660146 2.855025 -2.999319
C -2.577364 1.566670 -1.666085
H -2.378454 0.824427 -2.435665
C 3.113633 -1.358916 -0.436205
C 4.358170 -0.816336 1.395836
C 5.374199 -1.262453 -0.684205
C 5.526172 -0.904970 0.648378
N 4.183580 -1.488457 -1.226150
N 3.165379 -1.041462 0.864969
H 4.387853 -0.551986 2.455413
H 6.502627 -0.707265 1.083793
H 6.242633 -1.365988 -1.339252
H 0.352987 2.020834 1.142418
H 1.674716 4.079444 1.183753
H 3.433573 4.489768 -0.530654
H 3.802482 2.789012 -2.315332
H 2.415816 0.768657 -2.415961
Si 1.369184 -1.539498 -1.182232
H 1.565803 -1.805594 -2.638300
H 0.729953 -2.751978 -0.595786

^{Ph}YSiB2
E = -2006.09230180
P -1.570822 -0.205367 -0.057180
C -0.252881 0.214214 -1.028422
C 0.379628 1.538643 -0.762395
C 1.445870 1.676933 0.139666
C 2.029153 2.914631 0.389793
C 1.550278 4.052932 -0.250121
C 0.488812 3.939797 -1.142955
C -0.083441 2.699036 -1.397666
C -1.205529 -0.829288 1.632436
C -2.117818 -0.734855 2.687506
H -3.086672 -0.262838 2.528852
C -1.786630 -1.233599 3.941964
H -2.498198 -1.155214 4.761911
C -0.543578 -1.825513 4.150824
H -0.284280 -2.210490 5.135427
C 0.368446 -1.916673 3.104923
H 1.344158 -2.371882 3.264315
C 0.040508 -1.417265 1.849035
H 0.760878 -1.472087 1.033702
C -2.584877 -1.491988 -0.839489
C -3.195793 -2.511090 -0.108374
H -3.046444 -2.576599 0.966819
C -3.984965 -3.454491 -0.757298
H -4.454072 -4.251265 -0.183529
C -4.167081 -3.385690 -2.134195
H -4.782246 -4.128137 -2.639179
C -3.556940 -2.372318 -2.867423
H -3.691159 -2.320538 -3.946015
C -2.767262 -1.427675 -2.224501
H -2.272101 -0.637301 -2.787126
C -2.650417 1.223096 0.269691

C -2.202441 2.216761 1.146011
H -1.257147 2.091673 1.672683
C -2.954890 3.368629 1.335367
H -2.595473 4.142007 2.011110
C -4.160358 3.533433 0.659949
H -4.750131 4.435557 0.811603
C -4.611836 2.545193 -0.208102
H -5.556412 2.669565 -0.734043
C -3.858066 1.393577 -0.407498
H -4.213034 0.624786 -1.090908
H 1.805621 0.793423 0.665920
H 2.857359 2.988656 1.092826
H 2.002298 5.023744 -0.054649
H 0.105594 4.825098 -1.648127
H -0.914306 2.608504 -2.095324
Si 0.682676 -1.046279 -1.982932
H 0.659269 -0.846541 -3.467205
H 0.144279 -2.415269 -1.726630
C 4.662478 -0.331401 -0.988239
C 4.253974 -1.081080 0.110842
C 5.925475 0.222581 -1.064144
C 5.085732 -1.314599 1.188415
C 6.774815 -0.006634 0.020148
H 6.231226 0.807307 -1.927367
C 6.364607 -0.758190 1.121964
H 4.752595 -1.901776 2.040195
H 7.778970 0.411675 0.005061
H 7.055326 -0.913995 1.947889
B 2.582139 -0.966955 -1.324416
O 2.971223 -1.490841 -0.091401
O 3.639829 -0.268847 -1.884056

^{Ph}YSiB1
E = -2275.28042292
P 1.767138 -0.707208 0.035126
C 0.153152 -0.187989 0.207518
C -0.256330 0.507596 1.433607
C 0.617956 1.311999 2.196420
H 1.652191 1.431477 1.883672
C 0.193228 1.986297 3.333844
H 0.908004 2.596414 3.884816
C -1.129430 1.907473 3.759547
H -1.464571 2.449014 4.641748
C -2.007311 1.097400 3.042824
H -3.041242 0.987480 3.367689
C -1.574060 0.396296 1.926296
H -2.267688 -0.277136 1.427974
C 3.042872 0.604898 -0.068355
C 4.372998 0.432769 0.319274
H 4.698543 -0.501039 0.770161
C 5.287434 1.465326 0.144951
H 6.322417 1.327565 0.452130
C 4.879099 2.670876 -0.416341
H 5.596746 3.477726 -0.553574
C 3.550578 2.850614 -0.787556
H 3.222897 3.796508 -1.210379
C 2.629417 1.825536 -0.607013
H 1.577430 1.965593 -0.860933
C 1.954269 -1.645254 -1.506449
C 2.628321 -1.126641 -2.610813

H 3.095430 -0.146135 -2.553704
C 2.693397 -1.861834 -3.789573
H 3.216814 -1.453483 -4.651715
C 2.089452 -3.111622 -3.867103
H 2.140451 -3.683488 -4.791787
C 1.420632 -3.633796 -2.763462
H 0.946769 -4.611412 -2.822557
C 1.352910 -2.904893 -1.584316
H 0.824823 -3.305027 -0.720042
C 2.358138 -1.870336 1.316171
C 1.674171 -1.981754 2.528281
H 0.803877 -1.359361 2.717785
C 2.098304 -2.898277 3.484242
H 1.561333 -2.975827 4.427473
C 3.193266 -3.718202 3.234238
H 3.518195 -4.438022 3.983213
C 3.863396 -3.629213 2.018106
H 4.707788 -4.283238 1.808940
C 3.446541 -2.711755 1.061235
H 3.957610 -2.664432 0.100287
N -3.966542 0.342964 -0.479421
C -3.724332 2.528015 -0.071429
C -4.575983 1.484016 0.030013
H -3.890550 3.569913 0.177396
H -5.604852 1.478379 0.370610
B -2.623588 0.683953 -0.890118
N -2.539323 2.097918 -0.650143
C -1.432850 2.956029 -0.803121
C -0.947046 3.671065 0.292285
C -0.845291 3.123374 -2.057573
C 0.083329 4.586454 0.118736
H -1.373505 3.484179 1.276684
C 0.185445 4.041094 -2.223943
H -1.227979 2.550213 -2.899229
C 0.637350 4.790410 -1.141460
H 0.456456 5.141569 0.977133
H 0.624662 4.183214 -3.209731
H 1.425906 5.528260 -1.277858
C -4.553917 -0.929941 -0.381874
C -4.422920 -1.846417 -1.427060
C -5.267171 -1.296689 0.762424
C -4.985735 -3.110943 -1.321231
H -3.882812 -1.549797 -2.322968
C -5.839868 -2.559054 0.855109
H -5.350376 -0.591854 1.587375
C -5.699922 -3.473373 -0.183264
H -4.874132 -3.816111 -2.142750
H -6.389673 -2.832967 1.753715
H -6.145580 -4.462955 -0.106877
Si -1.040022 -0.511406 -1.184653
H -1.501012 -1.934493 -1.277683
H -0.393067 -0.256178 -2.510327

$^{F}Y_2Si$
E = -3900.82263892
P 3.002688 0.852097 -0.129381
P -2.988565 1.007307 -0.038192
C 1.488788 0.339696 -0.727891
C 1.204431 -1.018491 -1.157918
C 1.635421 -2.188547 -0.520221

C 1.187899 -3.452498 -0.871015
C 0.271422 -3.607606 -1.904080
C -0.152042 -2.479320 -2.594862
C 0.341651 -1.234036 -2.243404
C 3.158558 1.065536 1.676078
C 3.400051 -0.035903 2.503438
H 3.641622 -1.002411 2.071201
C 3.312948 0.094081 3.882793
H 3.495050 -0.772291 4.514505
C 2.971375 1.315866 4.451489
H 2.892930 1.410638 5.532855
C 2.724295 2.413657 3.635544
H 2.451683 3.371536 4.074302
C 2.817259 2.291991 2.254922
H 2.608085 3.152301 1.623700
C 3.437840 2.486068 -0.818489
C 4.509373 3.220507 -0.302519
H 5.052787 2.852440 0.566976
C 4.878098 4.421018 -0.894479
H 5.711663 4.990889 -0.488608
C 4.181844 4.894264 -2.004419
H 4.470219 5.837814 -2.464074
C 3.124785 4.159354 -2.527087
H 2.582768 4.522704 -3.397815
C 2.756660 2.952289 -1.941628
H 1.942683 2.359247 -2.354537
C 4.364628 -0.212402 -0.683931
C 4.275342 -0.792965 -1.952083
H 3.385751 -0.633390 -2.556927
C 5.324840 -1.560823 -2.440178
H 5.245912 -2.016504 -3.425073
C 6.472320 -1.743469 -1.675263
H 7.291935 -2.348170 -2.058579
C 6.574852 -1.145178 -0.424408
H 7.475642 -1.275633 0.171890
C 5.526562 -0.378597 0.071565
H 5.616450 0.089951 1.048973
C -1.378201 0.493867 0.174804
C -1.103176 -0.752650 0.886119
C -0.214625 -0.778891 1.970925
C 0.170731 -1.956970 2.592275
C -0.343921 -3.172517 2.156387
C -1.257235 -3.186496 1.111943
C -1.612829 -1.995482 0.498203
C -4.038486 0.412709 1.327821
C -4.149272 1.139418 2.514682
H -3.683093 2.117797 2.602184
C -4.863493 0.619587 3.586965
H -4.951897 1.196241 4.505516
C -5.462214 -0.632410 3.487170
H -6.017331 -1.039489 4.330129
C -5.350827 -1.362119 2.309159
H -5.814625 -2.342964 2.226348
C -4.645833 -0.842410 1.229449
H -4.563728 -1.414163 0.307970
C -3.020359 2.827259 -0.029643
C -3.778292 3.577004 -0.930493
H -4.376991 3.078659 -1.688603
C -3.753654 4.965820 -0.872601
H -4.340733 5.544439 -1.582977

C -2.975892 5.613509 0.081081
H -2.956907 6.701055 0.120449
C -2.213984 4.871140 0.977521
H -1.594916 5.373423 1.718068
C -2.229890 3.483420 0.921233
H -1.616064 2.893949 1.600619
C -3.921156 0.507774 -1.527095
C -5.311519 0.638818 -1.602471
H -5.869835 1.041591 -0.757740
C -5.982225 0.239577 -2.751640
H -7.064536 0.338909 -2.809508
C -5.271955 -0.300156 -3.822392
H -5.802753 -0.623074 -4.716187
C -3.890913 -0.437915 -3.744963
H -3.328509 -0.876371 -4.566816
C -3.216944 -0.030253 -2.599350
H -2.141583 -0.146392 -2.518839
Si 0.048927 1.484806 -0.464129
H 0.570854 2.559079 0.444381
H -0.428252 2.243492 -1.664701
F 2.447190 -2.099436 0.537391
F 1.569004 -4.508322 -0.166476
F -0.190828 -4.807357 -2.218598
F 0.029224 -4.301753 2.737053
F -1.721970 -4.339481 0.652004
F -1.012993 -2.594167 -3.597844
F -2.430981 -2.071961 -0.555774
F -0.050094 -0.185303 -2.972925
F 0.275256 0.367652 2.436355
F 1.017379 -1.942139 3.613265

^{F}YSiCl
E = -2556.51391356
P -0.995190 0.345950 0.079727
C 0.069705 -0.664576 -0.810303
C 1.509140 -0.444106 -0.724653
C 2.098202 0.827239 -0.711986
C 3.462243 1.035583 -0.578266
C 4.315406 -0.052534 -0.455365
C 3.780443 -1.335256 -0.472992
C 2.409613 -1.511083 -0.581942
C -0.132040 1.012170 1.530202
C -0.217965 2.349214 1.916851
H -0.826157 3.046808 1.346580
C 0.497793 2.797710 3.021098
H 0.431455 3.842530 3.317184
C 1.304838 1.919890 3.736430
H 1.867458 2.277241 4.596685
C 1.405636 0.588569 3.344340
H 2.048693 -0.097312 3.891786
C 0.696260 0.135101 2.240155
H 0.790930 -0.898330 1.908733
C -2.411462 -0.635633 0.654240
C -2.348919 -1.333468 1.861771
H -1.512682 -1.183420 2.539497
C -3.351715 -2.231779 2.197271
H -3.296644 -2.773674 3.138963
C -4.413289 -2.451162 1.325590
H -5.188270 -3.170531 1.582527
C -4.487086 -1.748948 0.128589

H -5.318582 -1.914276 -0.553190
C -3.495525 -0.834830 -0.204890
H -3.556077 -0.288450 -1.143939
C -1.779552 1.732056 -0.801061
C -1.353536 2.054103 -2.087137
H -0.534646 1.487508 -2.527167
C -1.975463 3.083819 -2.785365
H -1.641752 3.334666 -3.790128
C -3.022630 3.787930 -2.201517
H -3.508890 4.592355 -2.750162
C -3.458352 3.460772 -0.919504
H -4.285095 4.005251 -0.467708
C -2.843370 2.430201 -0.221108
H -3.197921 2.157301 0.772648
Cl -1.257777 -3.692235 -0.596747
Si -0.626252 -2.064564 -1.750037
H 0.378226 -2.561585 -2.718225
H -1.855103 -1.666593 -2.485475
F 3.953852 2.265336 -0.578386
F 5.621747 0.127825 -0.335246
F 4.579816 -2.382014 -0.338429
F 1.938234 -2.754544 -0.521612
F 1.321062 1.906425 -0.849882

^{F}YSiHMDS
E = -2970.31815159
P -1.931361 -0.463916 -0.063493
C -0.242309 -0.275091 0.012016
C 0.317491 1.067611 -0.158304
C -0.005869 2.155796 0.655985
C 0.557477 3.413736 0.498953
C 1.499752 3.620577 -0.500239
C 1.842808 2.568827 -1.341728
C 1.235860 1.332847 -1.179779
C -2.894523 -0.453297 1.488769
C -4.286984 -0.324074 1.491231
H -4.825446 -0.207296 0.550982
C -4.983291 -0.329895 2.693119
H -6.066513 -0.224931 2.693314
C -4.293480 -0.462266 3.895915
H -4.840194 -0.463697 4.837046
C -2.908270 -0.581051 3.896807
H -2.367727 -0.672790 4.836567
C -2.207860 -0.571952 2.695348
H -1.121434 -0.641985 2.681644
C -2.323613 -2.053627 -0.863150
C -3.342002 -2.901903 -0.426348
H -3.935793 -2.643395 0.446407
C -3.588416 -4.095194 -1.096827
H -4.378956 -4.755850 -0.746726
C -2.823246 -4.447602 -2.202900
H -3.017389 -5.384025 -2.722536
C -1.799848 -3.610024 -2.636761
H -1.185893 -3.889622 -3.490493
C -1.545613 -2.421474 -1.966522
H -0.723296 -1.773777 -2.268483
C -2.672320 0.872361 -1.051831
C -3.113608 2.041661 -0.426921
H -3.095631 2.118493 0.657662
C -3.562502 3.111955 -1.192421

H -3.898315 4.022191 -0.699840
C -3.579078 3.020269 -2.579576
H -3.932316 3.858865 -3.176529
C -3.142386 1.856366 -3.204623
H -3.155260 1.780563 -4.289947
C -2.685970 0.786465 -2.445216
H -2.342458 -0.119282 -2.939518
N 2.558022 -1.213680 0.445132
Si 3.596366 -1.752742 -0.879339
Si 3.235665 -0.603440 1.960046
C 2.572805 -2.596744 -2.209757
H 2.178102 -3.561647 -1.871415
H 1.730778 -1.973836 -2.532148
H 3.211764 -2.778558 -3.084030
C 4.577924 -0.353585 -1.662928
H 5.434442 -0.055757 -1.049126
H 4.974884 -0.701500 -2.626521
H 3.970437 0.535330 -1.860145
C 4.831020 -3.014931 -0.226660
H 5.453418 -3.406807 -1.042065
H 5.505364 -2.579931 0.522114
H 4.311882 -3.861051 0.241759
C 4.851865 0.302509 1.664962
H 5.192435 0.728566 2.618106
H 5.651508 -0.346762 1.289472
H 4.717338 1.128597 0.955713
C 3.540798 -2.024172 3.149980
H 3.968052 -1.664428 4.095389
H 2.603318 -2.546176 3.377989
H 4.235908 -2.755773 2.719867
C 2.061869 0.632622 2.752494
H 1.004881 0.347337 2.682361
H 2.309119 0.746142 3.816142
H 2.170951 1.615845 2.278605
Si 0.866295 -1.659093 0.535499
H 0.595934 -2.909598 -0.222084
H 0.615558 -2.038520 1.966680
F 1.529788 0.382338 -2.061180
F 2.719498 2.764625 -2.317791
F 2.056243 4.810752 -0.658324
F 0.223466 4.408471 1.308483
F -0.861508 1.983047 1.664906

$^{F}YSiC_6F_5$
E = -2824.84605413
P 2.125337 -0.216359 -0.026978
C 0.466023 -0.512163 -0.273883
C -0.482792 0.582829 -0.480193
C -1.414122 0.541272 -1.526459
C -2.426974 1.477261 -1.676938
C -2.524010 2.532375 -0.777487
C -1.621485 2.615588 0.275398
C -0.644053 1.642412 0.421997
C 3.212564 -0.897389 -1.326389
C 4.549171 -0.495075 -1.420485
H 4.949267 0.233360 -0.715590
C 5.361492 -1.009869 -2.422091
H 6.399817 -0.692244 -2.494585
C 4.843832 -1.922781 -3.338015
H 5.481363 -2.323858 -4.123602

C 3.510915 -2.308359 -3.259686
H 3.099769 -3.007397 -3.984999
C 2.692172 -1.791204 -2.260262
H 1.638877 -2.060661 -2.216822
C 2.744394 -0.894169 1.544991
C 3.955370 -1.575832 1.674712
H 4.585198 -1.750314 0.806321
C 4.351910 -2.055005 2.918807
H 5.293102 -2.592658 3.014105
C 3.545068 -1.856419 4.033431
H 3.857457 -2.234757 5.004968
C 2.331646 -1.186249 3.904929
H 1.691399 -1.041062 4.772584
C 1.925670 -0.711858 2.664735
H 0.968244 -0.208527 2.548708
C 2.507724 1.553406 -0.070862
C 2.204752 2.248572 -1.245181
H 1.772738 1.718094 -2.092886
C 2.451524 3.612294 -1.324116
H 2.209289 4.154079 -2.235995
C 3.007659 4.282558 -0.237894
H 3.200140 5.351785 -0.300906
C 3.316954 3.589226 0.926843
H 3.750320 4.113789 1.775786
C 3.064951 2.224520 1.015658
H 3.290443 1.685322 1.933269
C -2.075788 -1.888609 0.303198
C -4.403371 -1.773480 -0.398174
C -3.703251 -0.609050 1.592548
C -4.701371 -0.964199 0.691152
C -2.416706 -1.082912 1.383346
C -3.099324 -2.223394 -0.575112
F -2.852645 -2.962053 -1.647678
F -1.474932 -0.700109 2.245282
F -3.989138 0.166158 2.624718
F -5.934560 -0.533861 0.873524
F -5.357798 -2.104874 -1.250209
F 0.126448 1.712565 1.505591
F -1.725357 3.600328 1.153883
F -3.468462 3.447265 -0.921453
F -3.288674 1.380644 -2.677174
F -1.368003 -0.469861 -2.392692
Si -0.245652 -2.196457 -0.080353
H 0.398840 -2.935148 1.041705
H -0.212335 -3.105652 -1.257669

^{F}*YSiPy*
E = -2343.99344619
P -1.566115 -0.102628 0.093633
C -0.013655 -0.521291 0.659677
C 1.110999 0.397439 0.501183
C 2.147612 0.412101 1.446388
C 3.324437 1.124846 1.277453
C 3.490950 1.922240 0.152842
C 2.487416 1.950176 -0.806727
C 1.342315 1.188866 -0.632609
C -2.907260 -0.588409 1.235466
C -4.127846 0.094246 1.201693
H -4.265661 0.930962 0.518453
C -5.161042 -0.269912 2.056431

H -6.104241 0.271837 2.024018
C -4.984327 -1.314197 2.957920
H -5.792334 -1.597744 3.629509
C -3.766370 -1.982699 3.011059
H -3.614251 -2.787941 3.726740
C -2.729869 -1.617773 2.159753
H -1.773726 -2.127755 2.241103
C -2.029875 -0.830696 -1.516525
C -3.348585 -0.863490 -1.979453
H -4.157321 -0.464687 -1.371099
C -3.637466 -1.419069 -3.219323
H -4.665335 -1.440976 -3.576122
C -2.614677 -1.955652 -3.996673
H -2.843546 -2.396886 -4.965101
C -1.304797 -1.936861 -3.531708
H -0.504116 -2.365536 -4.131001
C -1.006461 -1.375774 -2.294705
H 0.021503 -1.369797 -1.928259
C -1.756096 1.697214 -0.019930
C -1.408252 2.447315 1.106448
H -1.031655 1.940178 1.993757
C -1.535569 3.829779 1.082143
H -1.259687 4.413709 1.957820
C -2.010639 4.465346 -0.061593
H -2.108475 5.549116 -0.079750
C -2.352749 3.718713 -1.183687
H -2.713977 4.216178 -2.081425
C -2.222223 2.334230 -1.168266
H -2.466038 1.751837 -2.054154
C 2.198943 -2.431418 0.199236
C 4.583117 -2.659850 0.412434
C 3.535755 -1.872413 -1.583934
C 4.700554 -2.183383 -0.885590
F 0.474989 1.159201 -1.646767
F 2.649394 2.671664 -1.907228
F 4.596177 2.635592 -0.008642
F 4.276373 1.073115 2.197965
F 2.042496 -0.354334 2.533606
N 2.322611 -1.987890 -1.062543
H 5.468346 -2.913129 0.993557
H 5.673085 -2.048568 -1.353926
H 3.592700 -1.501915 -2.609636
C 3.312254 -2.789663 0.961347
H 3.179195 -3.132550 1.985699
Si 0.453101 -2.285448 0.916249
H -0.510793 -3.172129 0.198615
H 0.511932 -2.790200 2.322113

^{F}YSiPyr
E = -2360.05064089
P 1.541561 -0.110088 -0.093860
C -0.016694 -0.494520 -0.668540
C -1.127417 0.431730 -0.468302
C -2.151644 0.528482 -1.422818
C -3.282716 1.311962 -1.248485
C -3.426587 2.066813 -0.091296
C -2.443919 1.994997 0.887472
C -1.341776 1.175602 0.700623
C 2.876081 -0.534669 -1.267328
C 4.097714 0.143561 -1.196112

H 4.238110 0.938363 -0.465127
C 5.128329 -0.169205 -2.073580
H 6.072122 0.368834 -2.011023
C 4.947982 -1.156801 -3.036202
H 5.753739 -1.400093 -3.726015
C 3.728968 -1.819272 -3.126637
H 3.573589 -2.579148 -3.889619
C 2.694955 -1.505847 -2.251957
H 1.738355 -2.009230 -2.363230
C 2.015231 -0.917542 1.475087
C 3.335227 -0.968879 1.932474
H 4.141198 -0.537682 1.343043
C 3.629276 -1.584220 3.142548
H 4.658173 -1.620008 3.495197
C 2.610571 -2.162022 3.895268
H 2.843548 -2.649416 4.840279
C 1.299363 -2.124551 3.435252
H 0.502018 -2.584492 4.015557
C 0.996168 -1.503810 2.228312
H -0.031859 -1.479748 1.864202
C 1.732891 1.682116 0.108224
C 1.385745 2.484516 -0.981967
H 1.011515 2.019477 -1.893086
C 1.510453 3.864325 -0.891865
H 1.235043 4.488700 -1.739320
C 1.982389 4.445445 0.281790
H 2.078185 5.527285 0.351694
C 2.324241 3.646845 1.367603
H 2.683339 4.101782 2.288441
C 2.196260 2.264332 1.286216
H 2.439723 1.640797 2.143760
C -2.216282 -2.454265 -0.296825
C -4.434828 -2.901388 -0.523124
C -3.557372 -2.091461 1.512116
C -4.669994 -2.495295 0.783581
F -0.490589 1.060684 1.722485
F -2.588231 2.675505 2.016179
F -4.490938 2.837838 0.080075
F -4.214912 1.362413 -2.188532
F -2.060367 -0.190369 -2.539801
Si -0.484809 -2.232249 -1.054933
H 0.478948 -3.163855 -0.393286
H -0.560682 -2.639539 -2.484200
N -2.343130 -2.067303 0.981155
N -3.221235 -2.884095 -1.062788
H -5.252653 -3.247591 -1.159141
H -5.667891 -2.496781 1.215127
H -3.650420 -1.771569 2.552294

^{F}YSiB2
E = -2502.99787334
P -1.964949 -0.094406 0.083222
C -0.473783 -0.442101 0.829099
C 0.690114 0.411418 0.578092
C 1.613725 0.675433 1.600923
C 2.815227 1.337769 1.388034
C 3.132019 1.803306 0.119957
C 2.241243 1.579567 -0.921622
C 1.063929 0.887837 -0.687421
C -3.397253 -0.209703 1.210815

C -4.554487 0.529569 0.944698
H -4.593443 1.187919 0.078060
C -5.649953 0.449572 1.795656
H -6.543240 1.033181 1.582081
C -5.598503 -0.364187 2.922503
H -6.454989 -0.424472 3.591170
C -4.444164 -1.087620 3.201767
H -4.391261 -1.712522 4.090845
C -3.345063 -1.006651 2.354717
H -2.438280 -1.551220 2.604747
C -2.398474 -1.166089 -1.332783
C -3.703245 -1.279142 -1.820944
H -4.518129 -0.738762 -1.344626
C -3.970411 -2.096456 -2.912130
H -4.988130 -2.180604 -3.288153
C -2.940464 -2.813480 -3.514359
H -3.152851 -3.458465 -4.365018
C -1.643591 -2.713184 -3.023246
H -0.837455 -3.279492 -3.485097
C -1.369778 -1.892393 -1.935256
H -0.356461 -1.812352 -1.545153
C -2.018257 1.626140 -0.485139
C -1.712686 2.616608 0.452413
H -1.453111 2.330548 1.470901
C -1.732599 3.952932 0.077031
H -1.490194 4.723321 0.806128
C -2.057774 4.303803 -1.230516
H -2.071499 5.351741 -1.523908
C -2.358325 3.318008 -2.163874
H -2.603519 3.592053 -3.187895
C -2.335620 1.976957 -1.795733
H -2.549422 1.203674 -2.530466
C 3.333603 -1.671939 -0.592142
C 3.874178 -1.645932 0.689296
C 4.028074 -1.201395 -1.689451
C 5.135929 -1.145286 0.942900
C 5.301493 -0.685255 -1.443744
H 3.583163 -1.199849 -2.680805
C 5.843539 -0.659427 -0.157534
H 5.532463 -1.104622 1.953406
H 5.878881 -0.282082 -2.272608
H 6.834028 -0.236336 -0.006598
B 1.782717 -2.295647 0.836215
O 2.932149 -2.081125 1.575407
O 2.051086 -2.129586 -0.518427
F 0.312977 0.591016 -1.750309
F 1.368778 0.213753 2.824466
F 3.672916 1.504426 2.383816
F 2.563163 1.951406 -2.152695
F 4.282781 2.419100 -0.102567
Si -0.104175 -2.133473 1.503260
H -1.048272 -3.125105 0.904013
H -0.202440 -2.317327 2.981641

^{F}YSiB1
E = -2772.18989022
P -1.944282 0.947861 0.108366
C -0.327915 0.472160 -0.108445
C 0.314562 -0.412976 0.861335
C -0.155841 -1.685910 1.191189

C 0.556642 -2.566986 1.990997
C 1.786602 -2.190694 2.511292
C 2.281822 -0.923687 2.228322
C 1.538704 -0.055523 1.445419
C -3.283651 -0.035312 -0.648188
C -4.620045 0.147157 -0.279210
H -4.878170 0.869982 0.494475
C -5.616740 -0.602337 -0.890639
H -6.656471 -0.463369 -0.600549
C -5.281599 -1.536258 -1.868357
H -6.062799 -2.125909 -2.344718
C -3.951770 -1.726024 -2.226778
H -3.687795 -2.466763 -2.978049
C -2.950159 -0.979532 -1.615045
H -1.900904 -1.141135 -1.858728
C -2.149149 2.629814 -0.556412
C -3.248496 3.023109 -1.321658
H -4.029859 2.308272 -1.566172
C -3.338742 4.329551 -1.788564
H -4.195093 4.627985 -2.389974
C -2.336702 5.248634 -1.496114
H -2.410663 6.269640 -1.865902
C -1.234225 4.859514 -0.742382
H -0.440300 5.570601 -0.523797
C -1.134899 3.553950 -0.279307
H -0.261745 3.228952 0.284521
C -2.384263 0.974997 1.876339
C -2.872929 -0.185250 2.483638
H -3.070722 -1.070614 1.883691
C -3.100145 -0.210447 3.855149
H -3.474328 -1.119561 4.321626
C -2.850859 0.919409 4.625847
H -3.032597 0.898390 5.698686
C -2.369104 2.077589 4.023846
H -2.176288 2.965593 4.622407
C -2.130997 2.105538 2.655490
H -1.752715 3.014163 2.193362
N 3.547862 -0.095907 -1.086522
C 3.245942 -2.310754 -1.001553
C 4.117921 -1.313236 -0.734629
H 3.393824 -3.380797 -0.913124
H 5.135715 -1.382310 -0.368930
B 2.210646 -0.332873 -1.568970
N 2.082939 -1.771618 -1.536442
C 0.992265 -2.558156 -1.943989
C 0.599594 -3.689582 -1.224995
C 0.302432 -2.218515 -3.110632
C -0.477013 -4.452207 -1.658371
H 1.122637 -3.958744 -0.310501
C -0.768688 -2.989635 -3.540977
H 0.631933 -1.356487 -3.685194
C -1.166384 -4.108758 -2.816270
H -0.782350 -5.319436 -1.076494
H -1.290597 -2.715114 -4.456227
H -2.005190 -4.714226 -3.153770
C 4.215654 1.131041 -0.907814
C 4.128245 2.117407 -1.890221
C 4.967727 1.371219 0.242973
C 4.776143 3.333531 -1.716990
H 3.555775 1.914673 -2.792529

C 5.626481 2.583149 0.401629
H 4.993261 0.619101 1.027511
C 5.532793 3.570442 -0.574433
H 4.698619 4.096377 -2.489438
H 6.205052 2.762755 1.305844
H 6.046761 4.520611 -0.444264
Si 0.682242 0.978601 -1.589201
H 1.201832 2.379551 -1.535823
H -0.165144 0.947659 -2.824195
F -1.300719 -2.121078 0.663255
F 0.099445 -3.795892 2.202504
F 2.488785 -3.035736 3.251670
F 3.459429 -0.554086 2.718091
F 2.034506 1.157936 1.216285

I
E = -1279.05823590
C 1.668230 2.181834 -0.003779
C -0.475910 1.082643 -0.192618
C -1.084619 2.318530 -0.374065
C -0.324601 3.489363 -0.346434
C 1.046363 3.421682 -0.150080
H 2.749458 2.152146 0.133066
H -2.156095 2.372741 -0.547826
H -0.815745 4.450612 -0.483312
H 1.643795 4.330972 -0.118786
N -1.128326 -0.161962 -0.210922
C -2.509325 -0.340398 -0.062635
C -3.279927 0.445634 0.805749
C -3.138554 -1.388946 -0.745277
C -4.635857 0.195603 0.963848
H -2.801451 1.241851 1.369856
C -4.492787 -1.642821 -0.568724
H -2.551396 -1.999821 -1.429766
C -5.254142 -0.847484 0.279969
H -5.214683 0.817617 1.644832
H -4.957488 -2.464475 -1.111158
H -6.316900 -1.039514 0.411085
C 0.936775 0.990675 -0.035504
C 1.441287 -0.376715 0.039438
P 3.071735 -0.802919 0.128039
C 4.122467 -0.225106 -1.245904
H 4.031125 0.862642 -1.333115
H 5.174772 -0.492151 -1.095216
H 3.751650 -0.671847 -2.174343
C 3.957243 -0.240556 1.618757
H 5.015686 -0.524511 1.594293
H 3.874910 0.848642 1.696443
H 3.470258 -0.681085 2.494944
C 3.216222 -2.606274 0.126674
H 4.266396 -2.911527 0.177865
H 2.676959 -3.012199 0.989578
H 2.767528 -3.001946 -0.791507
Si 0.027058 -1.502462 -0.151051
H -0.337554 -2.436067 0.959652
H 0.015043 -2.376087 -1.372502

II
E = -1858.30978327
C 0.725090 2.865269 -0.062015

C 0.314075 1.560083 0.175754
C -0.853048 1.031245 -0.404758
C -1.590684 1.881759 -1.239864
C -1.189666 3.190459 -1.465605
C -0.028104 3.692251 -0.884090
H 1.636798 3.211103 0.421154
H -2.492399 1.496481 -1.710373
H -1.788991 3.823526 -2.117551
H 0.284019 4.718205 -1.065569
N -1.259051 -0.292295 -0.186935
S 1.270678 0.535353 1.273954
O 2.578588 1.201485 1.445507
O 0.440523 0.268197 2.444950
C 1.475407 -0.893813 0.347554
C -2.640078 -0.566808 -0.080224
C -3.477373 0.262449 0.676759
C -3.181643 -1.707424 -0.677493
C -4.822359 -0.041673 0.815901
H -3.052245 1.137494 1.163190
C -4.528557 -2.017284 -0.516396
H -2.544098 -2.348106 -1.285147
C -5.357285 -1.184286 0.223438
H -5.458612 0.611992 1.409977
H -4.931333 -2.912425 -0.986834
H -6.412107 -1.422302 0.342623
P 2.983648 -1.052047 -0.439349
C 3.440644 0.342611 -1.504226
H 4.423197 0.187919 -1.964006
H 2.675729 0.463478 -2.278754
H 3.457824 1.238824 -0.875414
C 2.965372 -2.513407 -1.501612
H 2.214363 -2.389524 -2.287924
H 3.953138 -2.648946 -1.954932
H 2.711378 -3.396478 -0.906460
C 4.359893 -1.255300 0.714824
H 4.331631 -0.391372 1.387816
H 4.197364 -2.168335 1.296736
H 5.320285 -1.304334 0.189351
Si -0.131798 -1.662083 0.006469
H -0.745946 -2.517852 1.048899
H -0.030035 -2.493307 -1.233155

III
E = -2620.39041897
C -0.398195 1.556905 -0.575182
C 0.398326 1.556886 0.575169
C 0.773188 2.773027 1.146164
C 0.386037 3.978775 0.577376
C -0.385793 3.978784 -0.577466
C -0.772981 2.773041 -1.146230
H 1.392569 2.746324 2.039222
H 0.691389 4.916080 1.038342
H -0.691094 4.916093 -1.038458
H -1.392339 2.746328 -2.039308
S -0.978930 0.097542 -1.507142
S 0.979019 0.097507 1.507183
O -2.179106 0.614205 -2.192395
O 0.178785 -0.315656 -2.321486
O -0.178626 -0.315435 2.321738
O 2.179373 0.614081 2.192197

C -1.402942 -1.134756 -0.439305
C 1.402826 -1.134917 0.439414
P 2.876356 -0.911205 -0.412667
P -2.876501 -0.911040 0.412697
C 2.954676 -2.034622 -1.818277
H 3.892900 -1.870076 -2.358848
H 2.902283 -3.072406 -1.476141
H 2.095736 -1.815739 -2.463004
C 3.081576 0.751549 -1.096241
H 3.075679 1.472590 -0.271987
H 4.027857 0.823008 -1.643971
H 2.232509 0.938072 -1.763874
C 4.334005 -1.200258 0.620051
H 4.329825 -2.243591 0.952341
H 5.264406 -0.976719 0.085772
H 4.224528 -0.551537 1.496054
C -2.955020 -2.034699 1.818090
H -3.893056 -1.869897 2.358910
H -2.903122 -3.072425 1.475703
H -2.095871 -1.816274 2.462672
C -3.081567 0.751635 1.096509
H -3.075246 1.472805 0.272374
H -4.028020 0.823214 1.643921
H -2.232671 0.937877 1.764456
C -4.334119 -1.199831 -0.620147
H -5.264548 -0.976393 -0.085870
H -4.224579 -0.550915 -1.495997
H -4.329953 -2.243089 -0.952679
Si -0.000114 -2.237453 -0.000035
H -0.428970 -3.131465 1.109294

IV
E = -1828.35408176
C 0.752359 3.036625 0.228358
C 0.223949 1.758168 0.260193
C -0.944900 1.344969 -0.381075
C -1.620665 2.317892 -1.118092
C -1.129558 3.621036 -1.169082
C 0.041941 3.984850 -0.501615
H 1.665694 3.287852 0.764324
H -2.544034 2.061632 -1.636510
H -1.674262 4.377384 -1.732086
H 0.395609 5.013000 -0.545818
B -1.223045 -0.220788 -0.105012
S 0.930774 0.309081 0.983194
O 1.825283 0.561988 2.114144
O -0.430214 -0.382482 1.281518
C 1.593138 -0.624383 -0.248639
C -2.722929 -0.713945 0.034552
C -3.654852 0.081782 0.716392
C -3.184719 -1.922191 -0.499165
C -4.980653 -0.309294 0.862515
H -3.328747 1.031167 1.141839
C -4.510860 -2.322484 -0.364180
H -2.488164 -2.569275 -1.033029
C -5.413633 -1.515478 0.319020
H -5.681444 0.328452 1.399674
H -4.840645 -3.267735 -0.793009
H -6.452315 -1.824174 0.427267
P 3.255194 -0.977597 -0.170395

C 4.317879 0.486288 -0.070255
H 5.376783 0.210638 -0.013254
H 4.138620 1.117615 -0.946628
H 4.031118 1.035010 0.833832
C 3.698810 -1.881283 -1.666380
H 3.481418 -1.255713 -2.538232
H 4.759824 -2.151972 -1.652786
H 3.085765 -2.787339 -1.724014
C 3.735579 -2.001790 1.244844
H 3.411749 -1.467722 2.145287
H 3.197704 -2.953944 1.187873
H 4.816601 -2.179372 1.276593
Si 0.154947 -1.224332 -1.300333
H 0.165496 -2.723334 -1.333597
H 0.343057 -0.799533 -2.722829

V
Not observed.

Pathway B

$^{Tos}Y_2Si$
E = -4084.10313426
C -4.019273 -2.434375 -1.351154
H -3.836010 -3.194624 -0.595859
C -3.073983 -1.436210 -1.537997
C 3.480948 2.221129 -1.008235
O -0.454220 -1.723033 -1.452160
C 3.453095 2.466784 -2.378784
H 2.575496 2.189234 -2.963445
C -2.805044 1.665469 3.476736
H -3.825166 1.294498 3.404745
C -5.183131 -2.414432 -2.110894
H -5.936590 -3.186137 -1.955119
C 2.127647 -4.808391 -2.130670
H 1.323454 -5.335761 -2.639124
C 1.834447 -3.654382 -1.415404
H 0.810654 -3.289788 -1.351968
C -2.027354 2.808438 -1.445765
H -1.307276 2.043029 -1.739931
C -2.753295 2.637748 -0.268968
C -5.252406 0.692046 0.193364
H -5.216931 1.539286 -0.487914
C -6.420530 -0.053886 0.315229
H -7.298289 0.221682 -0.266622
C 3.432930 -5.283131 -2.201366
H 3.654884 -6.185866 -2.767699
C 2.002989 -0.180652 -0.970816
Si 0.460919 -0.417254 -2.312055
O 2.341289 1.376766 1.202696
S 2.052157 1.486560 -0.238053
P 2.457422 -1.486316 0.198867
C 4.551020 3.068141 -2.972176
H 4.543165 3.256444 -4.044887
C 4.086862 -1.192955 2.462189
H 3.242827 -1.575028 3.031676
C 0.570190 -0.948072 2.164984
H 0.741754 0.096633 1.945214
C -5.331683 -1.516846 1.889893
H -5.355630 -2.387953 2.541736

C -4.160109 -0.783117 1.765478
H -3.257835 -1.095096 2.291046
C 4.172872 -3.458716 -0.813108
H 4.976022 -2.955701 -0.282927
C -1.999226 1.734587 2.340553
C -3.268163 -0.437311 -2.486569
H -2.512650 0.335605 -2.615902
C -3.638434 3.628562 0.164651
H -4.184368 3.500047 1.099712
C -4.120593 0.339259 0.928763
C 0.150671 -3.638633 2.753773
H -0.020720 -4.689730 2.974746
C 1.231114 -1.937534 1.432379
C 1.022233 -3.287959 1.733324
H 1.532547 -4.066282 1.172814
C 2.864680 -2.969463 -0.757888
C 4.453674 -4.609811 -1.540279
H 5.474848 -4.983032 -1.580822
C -6.697936 -1.368371 -3.829422
H -7.453812 -0.777139 -3.292759
H -7.115734 -2.370424 -3.977247
H -6.561765 -0.904200 -4.812492
C -0.506304 -2.651897 3.481273
H -1.191367 -2.933401 4.279180
C 5.664604 3.452105 -2.213092
C -6.464425 -1.151104 1.167654
H -7.379592 -1.733411 1.258874
C 6.238026 -0.187018 1.002807
H 7.071777 0.213226 0.429403
C -1.322696 0.138580 0.025565
P -2.490524 1.119947 0.693983
O -1.789170 -2.498380 0.454012
S -1.645252 -1.346616 -0.454845
O 0.918174 2.293153 -0.669907
C -5.412861 -1.414804 -3.058205
C -4.430605 -0.436727 -3.243098
H -4.584180 0.345013 -3.986185
C -2.207025 3.964971 -2.199068
H -1.634592 4.102739 -3.114360
C -3.103722 4.941378 -1.781047
H -3.242251 5.843674 -2.374551
C -3.819135 4.775589 -0.596291
H -4.513016 5.545698 -0.264190
C 5.058883 -0.528230 0.353583
H 4.981142 -0.388885 -0.724164
C -0.292536 -1.310358 3.191587
H -0.797681 -0.529072 3.757412
C 3.980299 -1.036635 1.081024
C 6.342322 -0.342755 2.381512
H 7.263816 -0.069152 2.891780
C 5.647497 3.215295 -0.838106
H 6.501098 3.518221 -0.233159
C 5.267028 -0.843631 3.107817
H 5.344069 -0.960550 4.186777
C -0.989389 2.518628 4.817558
H -0.591498 2.810716 5.787882
C -2.296726 2.056791 4.713214
H -2.925849 1.993453 5.599374
C 4.559523 2.600553 -0.226440
H 4.536900 2.402047 0.842599

C 6.836284 4.115307 -2.871742
H 6.547441 5.080998 -3.306292
H 7.231062 3.501204 -3.690702
H 7.648155 4.296680 -2.160352
C -0.192325 2.613594 3.678402
H 0.831543 2.975930 3.746883
C -0.696855 2.232056 2.443281
H -0.074963 2.295271 1.552480
H 2.833037 -0.194405 -1.692311
H 1.292253 -1.503364 -3.008122

^{Tos}YSiCl
E = -2648.14565424
C 2.404098 -0.634331 1.221779
C 2.835093 0.599767 1.696053
H 2.250924 1.139728 2.439969
C -2.958271 3.510819 -1.099237
H -3.905969 3.756595 -1.573189
C -2.570962 2.181787 -0.995100
H -3.229629 1.395980 -1.353813
C -2.155744 4.519471 -0.577350
H -2.468779 5.558780 -0.653259
C -0.371389 -0.084836 1.487602
Si -1.931839 -0.483932 2.915491
O 0.553475 -2.465466 0.922322
S 0.856736 -1.319622 1.787480
P -0.806540 0.142272 -0.224441
C 4.025672 1.129100 1.217710
H 4.363916 2.099764 1.577463
C 0.805544 -1.313524 -1.961579
H 0.155665 -2.146669 -1.707204
C -2.485712 -2.077984 -0.052779
H -2.143464 -2.190612 0.974641
C -0.568387 2.870330 0.165919
H 0.360458 2.629793 0.681937
C -3.322723 -1.828951 -2.707720
H -3.642761 -1.736690 -3.743366
C -2.019952 -1.029126 -0.843464
C -2.421886 -0.915829 -2.179439
H -2.021334 -0.122322 -2.809333
C -1.363310 1.857214 -0.376951
C -0.963727 4.197995 0.061333
H -0.342207 4.980548 0.490937
C -3.804158 -2.866151 -1.912625
H -4.507953 -3.584745 -2.328343
C 4.806003 0.429864 0.291976
C 2.515203 0.800483 -2.560692
H 3.181932 1.629283 -2.790370
O 0.959896 -1.465313 3.233977
C 1.456858 0.996160 -1.682733
H 1.302022 1.980130 -1.250211
C -3.379376 -2.993647 -0.595559
H -3.743617 -3.810639 0.022904
C 0.590481 -0.058239 -1.383238
C 2.714198 -0.443842 -3.149409
H 3.538577 -0.591954 -3.844430
C 4.370189 -0.829174 -0.127160
H 4.972086 -1.395552 -0.836590
C 1.860773 -1.498470 -2.846093
H 2.016249 -2.476369 -3.297050

C 3.173187 -1.363901 0.327208
H 2.812536 -2.331316 -0.013186
C 6.065606 1.023953 -0.260704
H 6.857229 0.271841 -0.352894
H 6.437635 1.839652 0.367659
H 5.890825 1.433576 -1.265675
H -0.002091 0.869860 1.867436
H -1.466155 0.752665 3.680085
Cl -3.456520 0.598600 1.733474

^{Tos}YSiHMDS
E = -3061.94179912
C -2.382776 -2.219088 0.270588
C -2.089037 -3.383849 -0.430562
H -1.055242 -3.710478 -0.527705
C 0.617312 4.543055 -2.302803
H 1.028718 5.538460 -2.150785
C 0.279759 3.763528 -1.204417
H 0.445498 4.150156 -0.201892
C 0.444437 4.045242 -3.589873
H 0.718188 4.653109 -4.449922
C -0.504745 -0.249730 -0.414593
Si 1.036652 -1.337357 -1.436652
O -1.617378 -0.399444 2.010569
S -1.056759 -1.274368 0.970408
P -0.692124 1.478555 0.047301
C -3.134414 -4.086575 -1.012185
H -2.918952 -4.998586 -1.566904
C -2.917203 2.052151 1.692468
H -2.260888 1.881978 2.540708
C 1.301841 0.949947 1.873472
H 1.466938 0.025211 1.318845
C -0.406308 1.981366 -2.682512
H -0.779450 0.974315 -2.845415
C 0.935874 3.332387 3.288389
H 0.791475 4.258331 3.840994
C 0.332854 1.871573 1.467124
C 0.147529 3.062124 2.177491
H -0.625277 3.767903 1.876308
C -0.230702 2.477928 -1.388895
C -0.071440 2.767994 -3.778123
H -0.200852 2.371119 -4.782525
C 1.896315 2.412595 3.698125
H 2.505752 2.619901 4.575534
C -4.457156 -3.645105 -0.900318
C -4.627250 2.362681 -0.487944
H -5.291151 2.480988 -1.341711
O 0.014162 -2.189511 1.355782
C -3.273320 2.131399 -0.696289
H -2.890085 2.079521 -1.713670
C 2.073925 1.226370 2.994760
H 2.815135 0.499805 3.317919
C -2.410006 1.969804 0.391894
C -5.126607 2.449172 0.807110
H -6.186520 2.633500 0.971349
C -4.713381 -2.468932 -0.190206
H -5.738392 -2.113527 -0.095655
C -4.268360 2.300761 1.893108
H -4.655759 2.363983 2.907876
C -3.683315 -1.747653 0.399372

H -3.876702 -0.832055 0.955897
C -5.574138 -4.433055 -1.515130
H -5.772092 -5.344840 -0.935670
H -5.325091 -4.748710 -2.534994
H -6.503052 -3.854810 -1.550965
H -1.253942 -0.394438 -1.204223
H 1.253873 -0.160982 -2.389951
Si 3.763975 0.039993 -0.873429
Si 3.118235 -2.651334 0.373294
C 2.049389 -4.156706 0.039773
H 1.034213 -4.031878 0.425042
H 1.994711 -4.367880 -1.034977
H 2.510407 -5.020571 0.539621
C 4.832972 -3.191601 -0.214245
H 5.619317 -2.434077 -0.131801
H 5.149362 -4.061742 0.377243
H 4.782720 -3.508924 -1.264225
C 3.191988 -2.320508 2.223051
H 3.504830 -3.221344 2.767628
H 3.905956 -1.522557 2.461027
H 2.197675 -2.033366 2.585292
C 3.042269 1.747772 -1.175748
H 3.886053 2.441746 -1.298380
H 2.428395 1.799426 -2.080648
H 2.450091 2.107392 -0.327767
C 4.708945 -0.412454 -2.435540
H 3.998676 -0.529434 -3.265021
H 5.423591 0.374721 -2.710653
H 5.259849 -1.353607 -2.331165
N 2.575623 -1.204273 -0.492881
C 4.969068 0.311686 0.555478
H 5.723028 1.049080 0.248236
H 4.436236 0.728255 1.419904
H 5.499156 -0.585902 0.888607

^{Tos}YSiB1
E = -2863.81681644
C -3.273487 1.259037 -1.309149
C -3.001493 2.106994 -2.378155
H -2.237551 1.832198 -3.100671
C 3.438841 -0.591081 2.119304
H 4.359785 -1.122457 1.894334
C 2.244900 -1.035112 1.564873
H 2.249152 -1.900180 0.906857
C 3.454992 0.540486 2.925322
H 4.391924 0.892302 3.352236
C -0.880127 0.280616 -0.238553
Si 0.398218 1.480660 -1.234109
O -3.111795 -1.144210 -0.254195
S -2.356103 -0.246231 -1.143775
P -0.498832 -0.895888 1.089856
C -3.715047 3.291904 -2.479142
H -3.510316 3.967608 -3.308178
C -2.375506 -1.913884 2.921691
H -2.030636 -2.901219 2.628635
C -0.818846 -3.207249 -0.481765
H -1.250430 -2.596244 -1.267056
C 1.078445 0.786979 2.641195
H 0.166134 1.336465 2.852494
C 0.321665 -4.833602 1.484015

H 0.769134 -5.463283 2.250099
C -0.313555 -2.641360 0.687493
C 0.260266 -3.461219 1.671287
H 0.653977 -3.023885 2.587004
C 1.053557 -0.360113 1.844967
C 2.278037 1.237579 3.172823
H 2.292519 2.138604 3.781429
C -0.195230 -5.397794 0.320821
H -0.153065 -6.475423 0.174478
C -4.689233 3.635341 -1.535803
C -3.338072 0.596894 3.633103
H -3.715640 1.582245 3.898275
O -2.004859 -0.716776 -2.482506
C -2.310354 0.484848 2.705920
H -1.921910 1.394839 2.251543
C -0.758409 -4.585927 -0.653731
H -1.149799 -5.018508 -1.570550
C -1.808892 -0.772745 2.351806
C -3.886842 -0.543854 4.208580
H -4.696573 -0.456187 4.930079
C -4.943596 2.753617 -0.482087
H -5.710864 3.002214 0.249643
C -3.405666 -1.796391 3.846626
H -3.842048 -2.695041 4.277576
C -4.240316 1.561541 -0.359532
H -4.438521 0.859008 0.447595
C -5.440571 4.926201 -1.660787
H -5.928627 5.006406 -2.639984
H -4.762353 5.784442 -1.569651
H -6.210460 5.021110 -0.888470
H -1.285166 1.098744 0.372132
H 0.526885 2.373082 -0.005845
N 3.453092 1.330532 -0.698709
C 4.103711 -0.721969 -1.306508
C 4.528893 0.459639 -0.818593
H 4.700588 -1.578990 -1.592681
H 5.546427 0.770684 -0.613676
C 2.048730 -1.740412 -2.117589
C 0.934065 -1.510970 -2.926896
C 2.502920 -3.056452 -1.952956
C 0.322008 -2.563779 -3.593963
H 0.559636 -0.497673 -3.057234
C 1.896725 -4.100606 -2.638845
H 3.343674 -3.259657 -1.293093
C 0.811544 -3.860318 -3.476030
H -0.549123 -2.351840 -4.210071
H 2.274387 -5.113548 -2.509057
H 0.342670 -4.679118 -4.018633
C 3.623431 2.638200 -0.221247
C 2.842511 3.682092 -0.723479
C 4.593292 2.931242 0.743902
C 3.003382 4.974957 -0.244433
H 2.120557 3.476647 -1.511604
C 4.758714 4.229246 1.209406
H 5.209389 2.129293 1.142935
C 3.958946 5.259233 0.725704
H 2.380623 5.771367 -0.647926
H 5.517096 4.434278 1.963404
H 4.086080 6.275110 1.094192
N 2.730541 -0.666289 -1.529021

B 2.233862 0.644547 -1.122065

$^{Ph}Y_2Si$
E = -2906.96124534
P 2.645670 -0.415291 -0.066495
P -2.626953 -0.334784 -0.138956
C 1.272176 0.685330 -0.245277
C 1.584589 2.143411 -0.202182
C 2.196274 2.783080 -1.286798
H 2.447120 2.201634 -2.171853
C 2.446841 4.149382 -1.253931
H 2.922454 4.629589 -2.107546
C 2.067288 4.908181 -0.150441
H 2.245014 5.981790 -0.135718
C 1.440559 4.286964 0.924475
H 1.117577 4.872310 1.783084
C 1.212341 2.916932 0.900909
H 0.717542 2.436446 1.743918
C 3.690609 -0.597822 -1.527486
C 5.029627 -0.975440 -1.373217
H 5.452076 -1.099426 -0.378104
C 5.823735 -1.183427 -2.492917
H 6.865346 -1.472783 -2.369651
C 5.289463 -1.014900 -3.767410
H 5.915985 -1.173308 -4.643368
C 3.959518 -0.640044 -3.921626
H 3.538394 -0.502561 -4.915422
C 3.153717 -0.432646 -2.807354
H 2.109925 -0.124472 -2.935977
C 1.981439 -2.041448 0.365920
C 2.127589 -3.141836 -0.477697
H 2.644354 -3.034577 -1.428781
C 1.603068 -4.373645 -0.102158
H 1.716241 -5.229477 -0.764470
C 0.938059 -4.512261 1.112012
H 0.533595 -5.479638 1.403593
C 0.784019 -3.412965 1.952236
H 0.259002 -3.510210 2.899106
C 1.299055 -2.178207 1.579409
H 1.181259 -1.324300 2.243817
C 3.797106 0.043633 1.268663
C 4.348535 1.332545 1.247963
H 4.106771 2.021692 0.442310
C 5.211239 1.736620 2.257790
H 5.627957 2.741317 2.234032
C 5.539836 0.865091 3.291841
H 6.215153 1.186625 4.082376
C 5.009805 -0.419749 3.308240
H 5.273724 -1.110625 4.106552
C 4.143278 -0.832771 2.301703
H 3.746378 -1.844367 2.320017
C -1.620884 0.821219 -0.858728
C -1.848526 2.250216 -0.612134
C -1.351678 3.214011 -1.519190
H -0.820300 2.863102 -2.403306
C -1.500292 4.573316 -1.303868
H -1.087854 5.272964 -2.030377
C -2.164814 5.052784 -0.174864
H -2.276903 6.121805 -0.004398
C -2.678980 4.129786 0.727091

H -3.205496 4.471244 1.618010
C -2.522188 2.764438 0.516987
H -2.928837 2.080117 1.260765
C -4.366946 0.230090 -0.050646
C -4.809405 1.077160 -1.072264
H -4.104140 1.390688 -1.840076
C -6.124119 1.523827 -1.089524
H -6.456797 2.188124 -1.884678
C -7.007485 1.135266 -0.086844
H -8.036774 1.489313 -0.098785
C -6.570243 0.301077 0.935814
H -7.255348 0.000454 1.726423
C -5.253979 -0.149361 0.956782
H -4.920489 -0.789925 1.769455
C -2.716782 -1.913661 -1.051518
C -1.819771 -2.951964 -0.790163
H -1.067038 -2.832377 -0.015737
C -1.876432 -4.128350 -1.526282
H -1.170602 -4.928726 -1.313042
C -2.822592 -4.276327 -2.535835
H -2.864998 -5.198101 -3.113413
C -3.712518 -3.243082 -2.806467
H -4.453434 -3.351241 -3.596142
C -3.661401 -2.066751 -2.066692
H -4.363189 -1.263712 -2.280538
C -2.245185 -0.884214 1.586904
C -2.713745 -2.075975 2.148127
H -3.322563 -2.753626 1.551158
C -2.402133 -2.403396 3.463124
H -2.771384 -3.333533 3.892194
C -1.616533 -1.545523 4.229555
H -1.375030 -1.802370 5.259591
C -1.144011 -0.359113 3.677443
H -0.532578 0.316367 4.273496
C -1.455752 -0.032930 2.360810
H -1.090857 0.891400 1.913646
Si -0.075115 0.302336 -1.845350
H -0.069760 -1.197447 -1.548517
H 0.649154 0.398937 0.611310

^{Ph}YSiCl
E = -2059.57109201
P 0.605318 0.038198 -0.078541
C -0.713712 -0.377260 -1.210437
C -2.041163 0.235486 -0.906308
C -2.833817 0.682158 -1.968565
H -2.456243 0.591739 -2.986411
C -4.093980 1.220279 -1.743496
H -4.692891 1.556693 -2.587954
C -4.589615 1.330145 -0.448695
H -5.576070 1.753691 -0.271153
C -3.812782 0.888037 0.615708
H -4.188481 0.961547 1.634620
C -2.556667 0.341201 0.388315
H -1.972231 -0.008620 1.237273
C 2.157878 -0.044819 -1.006827
C 3.237572 0.763670 -0.634788
H 3.132266 1.475683 0.181895
C 4.440797 0.680410 -1.323598
H 5.274891 1.314257 -1.030018

C 4.573318 -0.203537 -2.389493
H 5.515533 -0.265880 -2.930411
C 3.498555 -0.999059 -2.770250
H 3.592720 -1.684929 -3.609223
C 2.292144 -0.924643 -2.084263
H 1.461708 -1.551940 -2.406075
C 0.778884 -0.947964 1.424890
C 1.892827 -0.711543 2.237735
H 2.640763 0.024889 1.949789
C 2.050802 -1.419555 3.420968
H 2.919460 -1.234275 4.049228
C 1.100965 -2.365973 3.796262
H 1.227335 -2.923336 4.722526
C -0.003657 -2.601826 2.986895
H -0.744934 -3.346464 3.268373
C -0.170284 -1.898690 1.798389
H -1.029926 -2.111166 1.164782
C 0.466962 1.762468 0.473345
C 0.595606 2.770632 -0.488050
H 0.874207 2.516523 -1.510317
C 0.371583 4.094869 -0.140950
H 0.475879 4.875613 -0.891412
C 0.008947 4.421488 1.163396
H -0.170104 5.460332 1.433261
C -0.129246 3.421757 2.118753
H -0.418475 3.674479 3.136639
C 0.096261 2.092612 1.777459
H -0.020008 1.311268 2.525795
Cl -2.504971 -2.957893 -0.488191
Si -0.856563 -2.372280 -1.795877
H 0.272327 -2.796274 -0.839629
H -0.330177 0.067251 -2.140313

^{Ph}YSiHMDS
E = -2473.35955050
P -1.774504 0.280018 -0.001784
C -0.195627 0.101263 -0.820159
C 0.256045 -1.324451 -0.733108
C 0.139017 -2.202145 -1.818922
H -0.268681 -1.837703 -2.760398
C 0.547612 -3.527104 -1.722165
H 0.451167 -4.184421 -2.584538
C 1.071219 -4.012472 -0.528157
H 1.384529 -5.051553 -0.447513
C 1.208622 -3.149653 0.554431
H 1.639226 -3.507392 1.487887
C 0.828349 -1.819098 0.444088
H 0.963551 -1.134313 1.277422
C -3.085379 -0.663108 -0.842035
C -4.207256 -1.117338 -0.141103
H -4.286701 -0.946474 0.930425
C -5.224914 -1.785537 -0.810384
H -6.092513 -2.138893 -0.256918
C -5.135815 -1.999902 -2.182344
H -5.932581 -2.526276 -2.704120
C -4.029602 -1.537625 -2.886550
H -3.958275 -1.696716 -3.960394
C -3.008406 -0.870545 -2.220366
H -2.152763 -0.502221 -2.780355
C -2.384948 1.989456 0.004518

C -3.617759 2.291234 -0.584421
H -4.216590 1.508479 -1.041255
C -4.089114 3.598742 -0.593241
H -5.049414 3.819373 -1.054443
C -3.336379 4.615984 -0.018516
H -3.705480 5.639797 -0.028951
C -2.109077 4.324116 0.565012
H -1.508327 5.115860 1.006888
C -1.628403 3.020631 0.577016
H -0.662085 2.810366 1.025833
C -1.733634 -0.371228 1.685554
C -1.845284 -1.754074 1.867478
H -2.003186 -2.405976 1.010836
C -1.728127 -2.300184 3.138593
H -1.807502 -3.377042 3.270182
C -1.496043 -1.473265 4.232732
H -1.398048 -1.902814 5.227859
C -1.389431 -0.098011 4.056689
H -1.207599 0.551489 4.909912
C -1.507738 0.455179 2.787225
H -1.421356 1.529477 2.660042
N 2.754056 0.629383 -0.211150
Si 3.691734 0.195041 1.205915
Si 3.559908 0.739456 -1.774369
C 2.756193 0.430303 2.824499
H 2.494074 1.482997 2.982444
H 1.830959 -0.151976 2.908036
H 3.426073 0.119999 3.638956
C 4.334545 -1.584732 1.159893
H 5.357537 -1.628107 0.767313
H 4.350909 -2.013683 2.170986
H 3.708426 -2.223249 0.528960
C 5.210281 1.308303 1.349820
H 5.775287 1.076094 2.262873
H 5.893161 1.180253 0.499903
H 4.919323 2.366105 1.387093
C 4.906807 -0.561797 -1.953724
H 5.328187 -0.499901 -2.966121
H 5.731614 -0.422882 -1.244727
H 4.504902 -1.572923 -1.813907
C 4.313747 2.443220 -2.030224
H 4.772308 2.537610 -3.023760
H 3.525015 3.201323 -1.939690
H 5.079647 2.664063 -1.277152
C 2.329713 0.456727 -3.171231
H 1.548520 1.228321 -3.165306
H 2.847724 0.509612 -4.138481
H 1.856176 -0.528911 -3.091063
H -0.393949 0.378330 -1.865066
Si 1.199929 1.583738 -0.197890
H 0.798641 1.375308 1.278530

^{Ph}YSiB1
E = -2275.25060654
P 1.742454 -0.356993 0.116761
C 0.507614 0.785392 0.747893
C 0.088437 1.712636 -0.357700
C -0.722425 1.299896 -1.421745
H -1.147248 0.300545 -1.413659
C -1.007696 2.158101 -2.475984

H -1.638727 1.809791 -3.291691
C -0.502886 3.455695 -2.485803
H -0.731244 4.128059 -3.310713
C 0.276308 3.890421 -1.419596
H 0.658755 4.909171 -1.399091
C 0.566235 3.027982 -0.370336
H 1.180943 3.375367 0.459092
C 1.253040 -1.442585 -1.248813
C 2.229459 -1.799578 -2.187586
H 3.228047 -1.372647 -2.131316
C 1.928775 -2.703646 -3.199425
H 2.695516 -2.974747 -3.922253
C 0.655485 -3.254977 -3.285487
H 0.421916 -3.964157 -4.077486
C -0.318433 -2.901398 -2.357370
H -1.317028 -3.328390 -2.408400
C -0.023982 -2.005187 -1.338668
H -0.780452 -1.746534 -0.602586
C 2.396910 -1.363827 1.461742
C 2.937480 -2.623418 1.196072
H 2.925187 -3.020122 0.183019
C 3.474487 -3.378782 2.230306
H 3.889213 -4.362522 2.021016
C 3.471331 -2.881045 3.530238
H 3.887201 -3.476494 4.340771
C 2.929895 -1.629074 3.797110
H 2.912587 -1.245158 4.814659
C 2.390447 -0.864517 2.768395
H 1.946588 0.102122 2.992042
C 3.140018 0.610862 -0.528177
C 2.973264 1.332374 -1.717551
H 2.049858 1.242003 -2.288522
C 3.987591 2.164941 -2.169947
H 3.850311 2.724707 -3.092642
C 5.169071 2.287285 -1.443868
H 5.961618 2.941848 -1.801518
C 5.337733 1.573147 -0.263167
H 6.261771 1.663771 0.304171
C 4.326350 0.736520 0.197104
H 4.462481 0.177203 1.120454
N -3.124389 1.360118 0.637753
C -4.478160 -0.123171 -0.350648
C -4.282618 1.187483 -0.110687
H -5.323885 -0.597891 -0.833579
H -4.931821 2.017076 -0.363776
B -2.509879 0.056668 0.873678
N -3.447967 -0.859424 0.230915
C -3.356468 -2.247646 0.082231
C -3.825186 -2.880822 -1.075799
C -2.783021 -3.029177 1.091086
C -3.715488 -4.258837 -1.220274
H -4.257912 -2.283482 -1.875049
C -2.654349 -4.401567 0.926779
H -2.457939 -2.551285 2.011717
C -3.118434 -5.027490 -0.226496
H -4.089096 -4.732176 -2.127036
H -2.198603 -4.990126 1.720910
H -3.024670 -6.104968 -0.344621
C -2.641718 2.638148 0.960099
C -1.913140 2.848239 2.135496

C -2.874402 3.723917 0.110407
C -1.410057 4.106920 2.433296
H -1.747553 2.013769 2.815213
C -2.383497 4.984147 0.426007
H -3.411130 3.571046 -0.822462
C -1.639806 5.184887 1.582774
H -0.843928 4.247710 3.352831
H -2.568034 5.813020 -0.255434
H -1.248438 6.171439 1.822952
H 1.110431 1.384864 1.447884
Si -0.804448 -0.284989 1.918037
H -0.372350 -1.639729 1.370648

I
E = -1828.35408176
C -1.924354 2.276573 -0.509017
C 0.171948 1.087122 -0.140298
C 0.675724 2.240297 0.489319
C -0.101024 3.388190 0.575809
C -1.393516 3.432342 0.058661
H -2.945119 2.277166 -0.893736
H 1.682178 2.237199 0.897951
H 0.320235 4.272773 1.051622
H -1.981702 4.345694 0.108101
N 0.826705 -0.106819 -0.317795
C 2.195969 -0.271734 -0.058414
C 3.158923 0.648979 -0.493749
C 2.633860 -1.430160 0.595620
C 4.508781 0.429468 -0.251419
H 2.837451 1.532731 -1.040257
C 3.985642 -1.651396 0.823754
H 1.890329 -2.159077 0.912480
C 4.931979 -0.719234 0.409901
H 5.239551 1.158309 -0.598649
H 4.301780 -2.559316 1.334914
H 5.990755 -0.890108 0.593797
C -1.171068 1.110893 -0.595714
P -2.559352 -1.023699 0.271532
C -1.465042 -1.249923 1.683150
H -1.071330 -0.271770 1.981509
H -2.011582 -1.707350 2.514527
H -0.630474 -1.888406 1.371622
C -3.987632 -0.087248 0.871754
H -4.478017 -0.615976 1.696716
H -3.645189 0.894556 1.215909
H -4.702653 0.054040 0.053764
C -3.155503 -2.641393 -0.252174
H -3.642859 -3.163213 0.578030
H -3.865217 -2.519211 -1.077837
H -2.284251 -3.206673 -0.606300
Si -0.040905 -1.351234 -1.439062
H 0.212285 -0.607652 -2.753724
C -1.679674 -0.225397 -1.059458
H -2.350245 -0.187488 -1.928334

II
E = -1858.27449606
C -0.590950 3.451798 0.179877
C -0.354307 2.100013 -0.035544
C -1.355955 1.206254 -0.446498

C -2.652253 1.722066 -0.573086
C -2.902714 3.066824 -0.342610
C -1.878020 3.943283 0.014945
H 0.235615 4.086201 0.492066
H -3.451218 1.050211 -0.879130
H -3.916360 3.445393 -0.463544
H -2.085565 4.998475 0.175691
N -1.030112 -0.106788 -0.784836
S 1.187219 1.438709 0.502964
O 2.139170 2.532143 0.694978
O 0.890481 0.558172 1.653004
C -1.774678 -1.151127 -0.225548
C -2.240604 -1.061174 1.095951
C -2.020163 -2.333595 -0.937864
C -2.936008 -2.114344 1.673749
H -2.021070 -0.163304 1.670060
C -2.702229 -3.388027 -0.343463
H -1.667221 -2.412011 -1.964160
C -3.170556 -3.288156 0.962830
H -3.285004 -2.020687 2.701062
H -2.882866 -4.295023 -0.918593
H -3.711417 -4.113322 1.421217
C 1.774904 0.331855 -0.771623
H 2.511391 0.874050 -1.377415
P 2.566387 -1.061472 0.027126
C 3.715182 -0.578427 1.332807
H 4.414341 0.164102 0.933478
H 3.136499 -0.122778 2.141425
H 4.266637 -1.452380 1.696934
C 3.522826 -1.890347 -1.256909
H 3.989263 -2.793830 -0.849370
H 2.834023 -2.152910 -2.069048
H 4.298284 -1.217259 -1.637696
C 1.369769 -2.238568 0.665869
H 1.899702 -3.075624 1.133386
H 0.731181 -1.724668 1.390110
H 0.756262 -2.599751 -0.168347
Si 0.315041 -0.372987 -2.033592
H 0.193755 1.004702 -2.659437

III
E = -2620.32742581
C 0.410792 1.576775 0.521724
C -0.396006 1.541189 -0.615971
C -0.767284 2.742905 -1.217523
C -0.375462 3.961766 -0.681766
C 0.412023 3.992660 0.463166
C 0.807360 2.803419 1.058641
H -1.384757 2.685434 -2.110828
H -0.683845 4.888139 -1.162649
H 0.724090 4.940865 0.895704
H 1.438183 2.803654 1.944136
S 0.931198 0.197885 1.573186
S -0.927812 0.026523 -1.486687
O 2.175864 0.678461 2.208213
O -0.191173 -0.138461 2.446935
O 0.324074 -0.377623 -2.172458
O -2.050638 0.523040 -2.306364
C -1.353892 -1.122650 -0.372235
P -2.857595 -0.868974 0.404331

P 2.806367 -0.927554 -0.456660
C -2.986835 -1.877944 1.889363
H -3.944621 -1.677645 2.382353
H -2.911807 -2.937907 1.630236
H -2.144967 -1.610882 2.537576
C -3.144041 0.838272 0.950809
H -3.117891 1.493874 0.073816
H -4.116689 0.926487 1.447896
H -2.337093 1.108982 1.640367
C -4.276495 -1.255391 -0.657013
H -4.239628 -2.318384 -0.917763
H -5.232922 -1.017195 -0.177331
H -4.144459 -0.666582 -1.571863
C 2.829850 -2.070688 -1.840391
H 3.738821 -1.894648 -2.427625
H 2.797043 -3.097762 -1.466105
H 1.925490 -1.883214 -2.430873
C 2.973599 0.749101 -1.093113
H 3.034881 1.446318 -0.252141
H 3.901428 0.785722 -1.675336
H 2.115424 0.972995 -1.732338
C 4.287487 -1.207747 0.541100
H 5.196648 -1.001908 -0.034288
H 4.220746 -0.538683 1.406937
H 4.302025 -2.247138 0.886876
Si -0.048573 -2.514564 -0.074735
H -0.467505 -2.805214 1.367338
C 1.388082 -1.243081 0.611664
H 1.805563 -1.848874 1.431792

IV
E = -1828.30293237
C -0.473809 3.424877 -0.473203
C -0.130506 2.073875 -0.434160
C 0.996011 1.517508 0.202310
C 1.853698 2.453285 0.812106
C 1.566394 3.804037 0.758942
C 0.411091 4.301115 0.123132
H -1.379503 3.764475 -0.971604
H 2.754881 2.104619 1.314080
H 2.255607 4.512219 1.216746
H 0.222380 5.371803 0.097676
B 1.017327 -0.061959 0.122821
S -1.087291 0.732733 -0.908296
O -2.029666 0.851348 -2.019186
O 0.030387 -0.312830 -1.109707
C 2.369818 -0.846191 -0.069459
C 2.716914 -1.458519 -1.279766
C 3.293028 -0.934335 0.981900
C 3.928892 -2.124541 -1.435987
H 2.020932 -1.407519 -2.116538
C 4.511783 -1.585357 0.830113
H 3.043504 -0.494583 1.949254
C 4.835216 -2.186920 -0.382821
H 4.170509 -2.594000 -2.389087
H 5.209230 -1.634656 1.665443
H 5.785316 -2.704811 -0.503876
C -1.808769 0.102349 0.632630
H -2.351298 0.922716 1.116857
P -2.918352 -1.251635 0.283544

C -4.374329 -0.762427 -0.673210
H -4.949335 -0.026041 -0.100727
H -4.037533 -0.303763 -1.607799
H -5.006123 -1.634075 -0.877707
C -3.532864 -1.943129 1.827599
H -4.266083 -2.728476 1.607884
H -2.674795 -2.334939 2.384491
H -4.007659 -1.148799 2.414190
C -2.042327 -2.514629 -0.646212
H -2.666545 -3.410599 -0.727254
H -1.804798 -2.123376 -1.640927
H -1.109069 -2.738164 -0.113654
Si -0.259021 -0.634828 1.765016
H -0.225223 0.622044 2.625456

V
E = -1279.10934965
C -1.810379 2.169775 -0.410354
C 0.313484 1.028014 -0.047919
C 0.838023 2.248141 0.410753
C 0.068336 3.402593 0.455736
C -1.256804 3.368784 0.029175
H -2.835275 2.165089 -0.777899
H 1.875774 2.283215 0.739281
H 0.500728 4.335899 0.813244
H -1.857227 4.277019 0.027776
B 1.128542 -0.304201 -0.231430
C 2.675115 -0.403174 -0.069955
C 3.536288 0.571577 -0.605551
C 3.279448 -1.498886 0.571242
C 4.918588 0.459279 -0.509812
H 3.111309 1.427509 -1.129546
C 4.659274 -1.606823 0.689325
H 2.641504 -2.276161 0.991875
C 5.487109 -0.627701 0.146583
H 5.556934 1.225998 -0.947088
H 5.094576 -2.463596 1.202108
H 6.569199 -0.713520 0.232064
C -1.045655 1.001970 -0.441438
P -3.015840 -0.827546 0.091434
C -2.626622 -0.720757 1.846006
H -2.422276 0.327021 2.093240
H -3.466829 -1.090547 2.442716
H -1.726288 -1.319907 2.026193
C -4.541892 0.139183 -0.133910
H -5.375144 -0.361421 0.372262
H -4.419551 1.140214 0.288705
H -4.771287 0.225675 -1.201742
C -3.497349 -2.517359 -0.319307
H -4.376611 -2.811492 0.264139
H -3.730866 -2.575611 -1.388536
H -2.649664 -3.176484 -0.100300
Si -0.177692 -1.701244 -0.492539
H -0.159942 -2.550571 -1.730596
C -1.596344 -0.338878 -0.876932
H -1.880199 -0.371154 -1.938559

Pathway C

IV
E = -1828.30293237
C 0.478650 3.287544 0.259278
C 0.065237 1.967947 0.251901
C -1.149489 1.503143 -0.244472
C -2.022367 2.460655 -0.755992
C -1.655537 3.804892 -0.749783
C -0.420388 4.220252 -0.248860
H 1.449555 3.579322 0.653537
H -2.988874 2.157565 -1.156311
H -2.344962 4.550798 -1.141868
H -0.161159 5.276894 -0.251545
B -1.218428 -0.093771 -0.092232
S 1.016673 0.573854 0.763572
O 1.782171 0.731266 2.003629
O -0.245383 -0.372298 1.006889
C 1.834322 -0.074720 -0.536385
C -2.582467 -0.882963 0.031442
C -3.652381 -0.331550 0.747884
C -2.767473 -2.145843 -0.540563
C -4.854735 -1.015998 0.894046
H -3.540442 0.652504 1.203626
C -3.965796 -2.837426 -0.400091
H -1.955817 -2.592644 -1.115140
C -5.014024 -2.272773 0.319444
H -5.671335 -0.566919 1.457502
H -4.085212 -3.818679 -0.857007
H -5.954958 -2.809582 0.429254
P 3.127083 -1.104390 -0.028407
C 4.507575 -0.181983 0.686025
H 5.307717 -0.859305 1.004725
H 4.884674 0.518425 -0.066140
H 4.122934 0.380973 1.542026
C 3.772514 -1.992859 -1.456788
H 4.106876 -1.270972 -2.208888
H 4.610350 -2.625399 -1.143439
H 2.974895 -2.600578 -1.896277
C 2.660955 -2.350325 1.202899
H 2.267917 -1.822908 2.079215
H 1.863752 -2.972400 0.781904
H 3.513751 -2.974204 1.493203
Si 0.793199 -0.462650 -2.098610
H 0.359123 0.956515 -2.399248
H -0.646303 -0.648963 -1.202538

V
E = -1279.12258772
C -1.810379 2.169775 -0.410354
C 0.313484 1.028014 -0.047919
C 0.838023 2.248141 0.410753
C 0.068336 3.402593 0.455736
C -1.256804 3.368784 0.029175
H -2.835275 2.165089 -0.777899
H 1.875774 2.283215 0.739281
H 0.500728 4.335899 0.813244
H -1.857227 4.277019 0.027776
B 1.128542 -0.304201 -0.231430
C 2.675115 -0.403174 -0.069955
C 3.536288 0.571577 -0.605551
C 3.279448 -1.498886 0.571242

C 4.918588 0.459279 -0.509812
H 3.111309 1.427509 -1.129546
C 4.659274 -1.606823 0.689325
H 2.641504 -2.276161 0.991875
C 5.487109 -0.627701 0.146583
H 5.556934 1.225998 -0.947088
H 5.094576 -2.463596 1.202108
H 6.569199 -0.713520 0.232064
C -1.045655 1.001970 -0.441438
P -3.015840 -0.827546 0.091434
C -2.626622 -0.720757 1.846006
H -2.422276 0.327021 2.093240
H -3.466829 -1.090547 2.442716
H -1.726288 -1.319907 2.026193
C -4.541892 0.139183 -0.133910
H -5.375144 -0.361421 0.372262
H -4.419551 1.140214 0.288705
H -4.771287 0.225675 -1.201742
C -3.497349 -2.517359 -0.319307
H -4.376611 -2.811492 0.264139
H -3.730866 -2.575611 -1.388536
H -2.649664 -3.176484 -0.100300
Si -0.177692 -1.701244 -0.492539
H -0.159942 -2.550571 -1.730596
C -1.596344 -0.338878 -0.876932
H -1.880199 -0.371154 -1.938559

4.2.5 Phenol coordinated species

Pathway A

I
E = -1615.89086215
C -0.572452 2.281335 1.669635
C 1.318469 1.118796 0.701855
C 2.082198 1.418164 1.831602
C 1.512121 2.135772 2.874103
C 0.186195 2.562937 2.796519
H -1.599189 2.643392 1.621351
H 3.119126 1.097115 1.888309
H 2.109230 2.370419 3.753095
H -0.253447 3.127106 3.616844
N 1.746887 0.425626 -0.426373
C 3.019594 -0.189293 -0.476052
C 3.897149 0.100214 -1.521137
C 3.391341 -1.135558 0.485110
C 5.123900 -0.548780 -1.607473
H 3.603295 0.840060 -2.262837
C 4.623124 -1.770295 0.401123
H 2.696001 -1.374362 1.288937
C 5.494563 -1.481410 -0.645767
H 5.797262 -0.316014 -2.430426
H 4.899187 -2.506631 1.153789
H 6.457325 -1.984141 -0.711609
C -0.028403 1.556945 0.598089
C -0.625916 1.175471 -0.662269
P -2.268155 1.472409 -1.058793
C -3.474610 0.823346 0.127238
H -3.242700 1.193233 1.130268
H -4.489316 1.128245 -0.152570
H -3.405264 -0.268881 0.139305

C -2.669575 3.236886 -1.235640
H -3.730283 3.387412 -1.466587
H -2.420262 3.755575 -0.303882
H -2.049413 3.654097 -2.035823
C -2.680404 0.707245 -2.641586
H -3.707858 0.961176 -2.922762
H -1.985441 1.057048 -3.412097
H -2.582080 -0.378800 -2.541278
Si 0.544661 0.316938 -1.769428
H 0.408806 -1.371599 0.352049
O -0.064750 -1.666983 1.141689
C -1.294959 -2.092450 0.818951
C -1.665170 -2.414174 -0.491520
C -2.230724 -2.231941 1.847410
C -2.955077 -2.857731 -0.762173
H -0.931338 -2.320986 -1.291348
C -3.514632 -2.679502 1.565033
H -1.925480 -1.977000 2.859481
C -3.890982 -2.990270 0.259869
H -3.225558 -3.112505 -1.786080
H -4.232700 -2.783365 2.376802
H -4.897556 -3.341620 0.044540

II
Not observed.

III
E = -2927.14122292
C -2.182997 -1.384367 -0.666676
C -2.933585 -0.684991 0.285325
C -4.269540 -1.011437 0.497026
C -4.869169 -2.030214 -0.233696
C -4.128167 -2.726021 -1.179393
C -2.793738 -2.400681 -1.395193
H -4.825805 -0.446779 1.240829
H -5.914008 -2.278247 -0.058526
H -4.587290 -3.524910 -1.758509
H -2.194916 -2.929457 -2.133068
S -0.461003 -1.038061 -1.146372
S -2.277360 0.644660 1.330578
O -0.033239 -2.260974 -1.868133
O -0.556109 0.174379 -1.975331
O -1.376388 -0.094889 2.337098
O -3.471496 1.311006 1.852929
C 0.476664 -0.876893 0.253149
C -1.095195 1.525809 0.615920
P -1.425522 2.760877 -0.512663
P 1.525282 -2.219103 0.518462
C 0.022672 3.138237 -1.504436
H -0.183189 3.993826 -2.156259
H 0.864763 3.352795 -0.838311
H 0.254261 2.239472 -2.084508
C -2.803809 2.325399 -1.598796
H -3.669141 2.086182 -0.968521
H -3.055673 3.154925 -2.268528
H -2.503383 1.440289 -2.167910
C -1.942754 4.288076 0.318000
H -1.116839 4.648444 0.940324
H -2.242039 5.066078 -0.393851
H -2.782472 4.028790 0.973239

C 2.316584 -2.032739 2.132395
H 2.993769 -2.878293 2.296462
H 2.877723 -1.096159 2.161785
H 1.551012 -2.010384 2.915029
C 0.711515 -3.838557 0.611152
H 0.242443 -4.045490 -0.353603
H 1.441177 -4.619005 0.855742
H -0.057556 -3.792002 1.389828
C 2.858687 -2.375683 -0.691500
H 3.503864 -3.234130 -0.472567
H 2.386965 -2.489870 -1.672305
H 3.444707 -1.449757 -0.674765
Si 0.310620 0.534881 1.483991
H 2.149416 2.011002 1.239689
O 2.575903 2.333534 0.417248
C 3.624646 1.546240 0.109318
C 4.347780 0.861851 1.090175
C 4.014243 1.442875 -1.227665
C 5.450737 0.092651 0.733839
H 4.050253 0.963358 2.132991
C 5.122540 0.680000 -1.570777
H 3.432898 1.967191 -1.982331
C 5.848623 -0.000831 -0.595714
H 6.009786 -0.428960 1.509122
H 5.418440 0.610752 -2.616077
H 6.715772 -0.596958 -0.870762

IV
E = -2135.11326429
C -1.533397 3.514281 0.968681
C -1.366194 2.165638 0.712017
C -2.284319 1.332468 0.061909
C -3.459901 1.948784 -0.374248
C -3.671037 3.304597 -0.132541
C -2.726636 4.086662 0.536981
H -0.775105 4.090504 1.496066
H -4.225703 1.365090 -0.883198
H -4.600966 3.766240 -0.461433
H -2.926239 5.139323 0.726368
B -1.685503 -0.150873 -0.042066
S 0.070804 1.181047 0.986928
O 0.939094 1.636575 2.076089
O -0.702938 -0.148589 1.209193
C 0.816319 0.931511 -0.522137
Si -0.260403 -0.087649 -1.632896
C -2.593989 -1.436857 -0.050471
C -2.468706 -2.443336 0.914946
C -3.543486 -1.635164 -1.063578
C -3.264012 -3.584357 0.882359
H -1.732604 -2.322758 1.708637
C -4.346158 -2.769618 -1.100270
H -3.645400 -0.889647 -1.853953
C -4.210573 -3.752049 -0.123532
H -3.145962 -4.348991 1.649520
H -5.077274 -2.892391 -1.898572
H -4.834948 -4.643719 -0.149547
P 2.332562 1.689195 -0.777829
C 2.286276 3.478378 -0.488139
H 3.256495 3.945694 -0.689766
H 1.512316 3.924925 -1.121247

H 2.020476 3.631695 0.564539
C 2.827720 1.407285 -2.486380
H 2.065692 1.812964 -3.159038
H 3.800818 1.871807 -2.679198
H 2.885181 0.325019 -2.646717
C 3.671752 1.071434 0.264282
H 3.323566 1.111944 1.302031
H 3.867514 0.027457 -0.000521
H 4.582124 1.669280 0.141543
C 4.165368 -2.744091 -0.313687
C 4.326422 -2.470658 1.040445
C 3.222140 -2.080154 1.795453
C 1.964357 -1.975271 1.216905
C 1.810134 -2.278506 -0.134485
C 2.910535 -2.640923 -0.908014
H 5.019246 -3.046379 -0.917208
H 5.306088 -2.559045 1.504654
H 3.338187 -1.855961 2.854136
H 1.091553 -1.673010 1.787514
H 2.778154 -2.861044 -1.967177
O 0.558740 -2.203688 -0.666094
H 0.513781 -2.696943 -1.492460

V
E = -1585.88590786
C 1.357973 1.014363 2.211550
C -0.854327 0.812678 1.238410
C -1.365529 0.556063 2.509432
C -0.537122 0.503831 3.631388
C 0.823543 0.731383 3.468228
H 2.428993 1.196720 2.139353
H -2.436686 0.392617 2.626162
H -0.947433 0.296002 4.617554
H 1.486194 0.699262 4.332570
B -1.635275 0.824965 -0.104568
C -3.120580 0.412049 -0.292569
C -3.694668 -0.683384 0.376726
C -3.940932 1.127053 -1.180991
C -5.023366 -1.036754 0.179138
H -3.071904 -1.281570 1.040423
C -5.281374 0.803059 -1.354065
H -3.510844 1.954889 -1.745034
C -5.824494 -0.284299 -0.676300
H -5.441239 -1.898879 0.696910
H -5.900596 1.385556 -2.034480
H -6.869236 -0.553620 -0.823782
C 0.538505 1.061789 1.075935
C 0.966184 1.323473 -0.308549
P 2.633702 1.504320 -0.706757
C 3.444385 2.959613 0.033134
H 3.347468 2.928763 1.122634
H 4.505313 3.006230 -0.238020
H 2.929328 3.855285 -0.330158
C 3.673678 0.085915 -0.257529
H 4.726554 0.270819 -0.499489
H 3.572055 -0.136961 0.808473
H 3.307263 -0.783366 -0.813611
C 2.876755 1.706440 -2.484498
H 3.944496 1.841681 -2.690997
H 2.496695 0.819468 -3.000390

H 2.305698 2.570938 -2.836627
Si -0.298027 1.350428 -1.618457
H -0.949422 -1.510343 -1.725508
O -0.761151 -1.531319 -0.780631
C 0.428886 -2.128664 -0.542206
C 1.211317 -2.653520 -1.570698
C 0.856132 -2.229280 0.782534
C 2.396422 -3.317467 -1.270123
H 0.877589 -2.556525 -2.603740
C 2.046063 -2.885955 1.066380
H 0.245578 -1.795448 1.571415
C 2.819669 -3.440864 0.049114
H 2.990019 -3.741154 -2.078325
H 2.369186 -2.967387 2.102716
H 3.744649 -3.963170 0.282955

Pathway B

I
E = -1615.89171800
C 1.866734 0.806051 2.077267
C -0.042054 -0.455986 1.271453
C -0.806006 0.025669 2.337066
C -0.239401 0.906612 3.248319
C 1.091916 1.304203 3.116977
H 2.910225 1.112248 2.003142
H -1.842672 -0.282377 2.443159
H -0.840692 1.284049 4.073126
H 1.529201 1.995351 3.834808
N -0.468429 -1.323894 0.276363
C -1.826658 -1.704371 0.156458
C -2.167424 -3.056090 0.095730
C -2.833726 -0.740828 0.040929
C -3.492146 -3.439401 -0.084280
H -1.379980 -3.801069 0.191137
C -4.155461 -1.130224 -0.124731
H -2.569630 0.313482 0.066419
C -4.492047 -2.479749 -0.190203
H -3.742495 -4.497596 -0.134203
H -4.926097 -0.367106 -0.218552
H -5.528919 -2.780821 -0.326255
C 1.324458 -0.084824 1.138310
P 3.539639 -0.395254 -0.540062
C 3.847625 1.353314 -0.928822
H 3.684963 1.962362 -0.033496
H 4.869977 1.506995 -1.292114
H 3.125174 1.666992 -1.691035
C 4.844833 -0.868050 0.632470
H 5.839240 -0.614368 0.247999
H 4.680228 -0.356651 1.586408
H 4.776195 -1.947022 0.806366
C 3.889119 -1.302519 -2.061775
H 4.917247 -1.108777 -2.385091
H 3.748562 -2.374231 -1.886881
H 3.186970 -0.983502 -2.839066
C 1.942743 -0.715384 -0.013519
H 0.896643 0.984544 -0.975792
Si 0.778157 -1.813717 -0.920203
O 0.438640 1.415299 -1.714380

C -0.701348 2.001554 -1.279014
C -1.773043 2.094981 -2.167264
C -0.828732 2.510862 0.014504
C -2.966602 2.673509 -1.755573
H -1.654576 1.686349 -3.167902
C -2.031058 3.078364 0.419051
H 0.006377 2.438412 0.709448
C -3.107536 3.162099 -0.459028
H -3.800452 2.731145 -2.453158
H -2.122185 3.452959 1.437054
H -4.047320 3.605596 -0.137206

//
E = -2165.07934981
C 0.274147 1.245196 2.683320
C 0.278976 0.227444 1.734373
C -0.885483 -0.173976 1.059067
C -2.069091 0.513417 1.379047
C -2.077285 1.519192 2.329675
C -0.908871 1.895150 2.991344
H 1.216480 1.501114 3.163418
H -2.986308 0.252651 0.858926
H -3.012448 2.032607 2.544349
H -0.923660 2.687058 3.736605
N -0.873080 -1.169100 0.074694
S 1.783165 -0.649056 1.460566
O 2.906931 0.195878 1.925634
O 1.655274 -1.975505 2.062808
C -2.123836 -1.815731 -0.170044
C -2.670406 -2.649756 0.806783
C -2.793446 -1.636430 -1.378854
C -3.877240 -3.294145 0.573277
H -2.130703 -2.785421 1.741924
C -3.996874 -2.294660 -1.612761
H -2.362458 -0.972959 -2.125729
C -4.542966 -3.121162 -0.638137
H -4.296840 -3.945102 1.337982
H -4.512389 -2.152179 -2.560609
H -5.486199 -3.632108 -0.820652
C 1.818906 -0.758222 -0.257203
Si 0.413668 -1.625313 -1.082057
O 0.374116 1.272521 -1.841251
C -0.508896 2.208687 -1.415895
C -0.244854 3.020034 -0.313289
C -1.703849 2.358097 -2.118023
C -1.180840 3.963719 0.089286
H 0.688371 2.895646 0.233983
C -2.631635 3.306801 -1.708028
H -1.887429 1.718154 -2.978059
C -2.378869 4.112733 -0.601666
H -0.969856 4.582963 0.959240
H -3.565106 3.413887 -2.257845
H -3.109687 4.852177 -0.281719
H 0.898112 0.925320 -1.095115
P 3.369738 -0.497740 -0.961221
C 3.266135 -0.887804 -2.720409
H 4.228171 -0.690351 -3.204957
H 2.989031 -1.938760 -2.854238
H 2.487621 -0.258801 -3.168416
C 4.675507 -1.501647 -0.218859

H 5.652552 -1.292298 -0.668116
H 4.688940 -1.259827 0.850065
H 4.418062 -2.558893 -0.339735
C 3.904821 1.225376 -0.847294
H 3.188338 1.844089 -1.399587
H 3.885613 1.495735 0.213490
H 4.910427 1.357341 -1.261794

III
E = -2927.12121346
C 2.796486 -0.282000 0.142728
C 2.378161 0.600033 -0.854888
C 3.322577 1.169586 -1.708321
C 4.672815 0.878134 -1.564669
C 5.090275 0.010730 -0.563088
C 4.153663 -0.561814 0.287226
H 2.973083 1.850864 -2.480065
H 5.396049 1.331068 -2.239826
H 6.146202 -0.221999 -0.439920
H 4.448429 -1.248818 1.076642
S 1.708484 -1.050468 1.378193
S 0.665033 1.013780 -1.303077
O 2.512458 -2.187014 1.858821
O 1.407335 0.067016 2.315143
O 0.172362 -0.127119 -2.083264
O 0.809557 2.306773 -2.007791
C 0.254097 -1.482534 0.697568
P -0.064212 2.856074 0.789701
P 0.199218 -2.893394 -0.259451
C -0.762608 2.887240 2.450897
H -0.622127 3.885308 2.881147
H -1.826366 2.632286 2.421186
H -0.241108 2.131706 3.050401
C 1.691807 3.223612 0.997978
H 2.158184 3.305495 0.011520
H 1.819965 4.158373 1.554659
H 2.129768 2.383482 1.551072
C -0.802983 4.216295 -0.141915
H -1.887455 4.065872 -0.179077
H -0.576641 5.183957 0.319828
H -0.397082 4.163853 -1.157011
C -1.304632 -2.946476 -1.244601
H -1.307207 -3.854648 -1.856864
H -2.179492 -2.940170 -0.586340
H -1.325635 -2.044991 -1.864683
C 1.597542 -3.014545 -1.402210
H 2.520556 -3.024950 -0.811351
H 1.532477 -3.929977 -2.000769
H 1.576867 -2.128361 -2.044658
C 0.270913 -4.412531 0.728605
H 0.333652 -5.311816 0.104964
H 1.157452 -4.323125 1.366976
H -0.616175 -4.460770 1.369034
C -0.335281 1.286676 0.078220
H -1.562757 0.762485 -0.457571
Si -0.984612 -0.162574 1.284589
O -2.209210 -0.228641 -0.349695
C -3.558851 -0.191665 -0.311802
C -4.258826 0.626763 -1.200512
C -4.258410 -0.986787 0.597978

C -5.648076 0.640915 -1.177752
H -3.700230 1.231294 -1.912424
C -5.647614 -0.967498 0.606608
H -3.698721 -1.601172 1.301128
C -6.350641 -0.154999 -0.277872
H -6.186942 1.278949 -1.876273
H -6.185757 -1.591700 1.318003
H -7.438292 -0.142563 -0.266376

IV
E = -2135.11433287
C 3.502534 2.362533 0.630421
C 2.278702 1.729767 0.508007
C 1.143707 2.250636 -0.121111
C 1.288341 3.519519 -0.686680
C 2.501519 4.195505 -0.581765
C 3.600338 3.634540 0.074746
H 4.336354 1.891372 1.147144
H 0.444851 3.989719 -1.190867
H 2.595793 5.192173 -1.010368
H 4.530352 4.193352 0.154926
B -0.050522 1.197131 -0.051606
S 1.876780 0.078073 0.962895
O 2.661064 -0.479479 2.069332
O 0.365196 0.386385 1.280860
C 1.780073 -0.852074 -0.446095
Si 0.325373 -0.327533 -1.513447
C -1.568695 1.613594 0.007053
C -2.360898 1.402070 1.141369
C -2.200446 2.141279 -1.128634
C -3.724535 1.678182 1.137650
H -1.900276 0.993123 2.040733
C -3.557165 2.441815 -1.133424
H -1.618883 2.293118 -2.039672
C -4.328952 2.201992 0.000227
H -4.319822 1.481679 2.028436
H -4.020354 2.849618 -2.031135
H -5.396268 2.416702 -0.005835
P 2.671072 -2.314699 -0.466625
C 4.443274 -2.109271 -0.158157
H 4.962929 -3.073803 -0.162226
H 4.863451 -1.453916 -0.928135
H 4.554690 -1.632159 0.821674
C 2.479833 -3.074910 -2.087894
H 2.870019 -2.391963 -2.849215
H 3.014300 -4.030380 -2.122305
H 1.412563 -3.226729 -2.280615
C 2.095359 -3.514897 0.762223
H 2.215605 -3.047264 1.746277
H 1.029255 -3.692735 0.582030
H 2.653396 -4.457070 0.720232
C -3.113700 -1.311624 -1.095769
C -2.374897 -1.642678 0.032626
C -3.005840 -2.034088 1.208514
C -4.394063 -2.072010 1.257716
C -5.146357 -1.734590 0.137916
C -4.500550 -1.359140 -1.035760
H -2.592748 -0.986724 -1.993835
H -2.407232 -2.291735 2.080550
H -4.889282 -2.366744 2.181057

H -6.233007 -1.758800 0.181175
H -5.081004 -1.080413 -1.912946
O -1.001464 -1.584070 -0.026697
H -0.645708 -1.118889 0.755212

V
E = -1585.88951218
C -0.867358 2.975669 -0.796854
C 1.115463 1.655620 -0.321333
C 1.856152 2.526981 -1.122569
C 1.260658 3.608273 -1.771178
C -0.100753 3.822622 -1.596739
H -1.921300 3.212172 -0.660152
H 2.930176 2.370507 -1.220146
H 1.851721 4.283236 -2.386552
H -0.580716 4.673713 -2.079026
B 1.660754 0.491430 0.555434
C 3.082105 -0.117901 0.471196
C 3.787735 -0.232595 -0.740124
C 3.702788 -0.619267 1.628278
C 5.051216 -0.805920 -0.791540
H 3.321352 0.111503 -1.662413
C 4.982166 -1.160004 1.591062
H 3.161681 -0.580038 2.573569
C 5.657369 -1.258009 0.378142
H 5.570555 -0.898830 -1.744064
H 5.447895 -1.525350 2.504905
H 6.652612 -1.698376 0.341496
C -0.286894 1.875576 -0.149336
C -0.957819 0.920034 0.743150
P -2.662514 0.925389 1.008641
C -3.248789 2.286599 2.069060
H -2.964974 3.242005 1.613831
H -4.335119 2.257536 2.212510
H -2.741932 2.204806 3.036387
C -3.732561 1.021793 -0.462686
H -3.494448 1.894020 -1.076148
H -3.577293 0.117410 -1.056591
H -4.781527 1.074670 -0.149936
C -3.188456 -0.604097 1.808966
H -4.268778 -0.575629 1.991105
H -2.935809 -1.438996 1.143783
H -2.640403 -0.736519 2.746869
Si 0.091163 -0.206553 1.739097
O 0.454674 -1.279080 -1.148168
C -0.679191 -2.009736 -1.122134
C -1.795779 -1.683153 -1.889562
C -0.712517 -3.130799 -0.291603
C -2.939799 -2.474053 -1.822452
H -1.759017 -0.810814 -2.541788
C -1.857327 -3.912471 -0.234008
H 0.164477 -3.359110 0.309168
C -2.980376 -3.590088 -0.994574
H -3.804727 -2.214086 -2.431173
H -1.874763 -4.782188 0.420177
H -3.874592 -4.207254 -0.945997
H 0.287001 -0.398431 -1.507593

Pathway C

IV
E = -2135.09391264
C 1.586899 3.185785 0.559032
C 1.105558 1.894736 0.377769
C -0.176848 1.607057 -0.119607
C -0.946568 2.725002 -0.473193
C -0.487840 4.026198 -0.297335
C 0.776238 4.264430 0.229963
H 2.596823 3.321895 0.938645
H -1.931523 2.577522 -0.911748
H -1.125141 4.862517 -0.580487
H 1.136677 5.281642 0.369132
B -0.628584 0.101774 -0.317131
S 2.222985 0.578541 0.827623
O 3.584984 1.166711 0.891433
O 1.738611 -0.058599 2.053203
C 2.197256 -0.545162 -0.511118
Si 0.705164 -1.064028 -1.369447
C -1.247484 -0.729762 0.879767
C -1.260727 -0.254417 2.197680
C -1.823560 -1.988546 0.644050
C -1.830468 -0.993494 3.226511
H -0.800100 0.706725 2.418208
C -2.403952 -2.730844 1.665857
H -1.831734 -2.392044 -0.370992
C -2.409327 -2.232434 2.964147
H -1.824309 -0.600810 4.242128
H -2.850565 -3.700215 1.449744
H -2.860009 -2.808828 3.770524
P 3.783604 -1.187666 -0.812292
C 4.926374 -0.021541 -1.591382
H 5.919356 -0.466755 -1.719781
H 4.516447 0.267004 -2.565014
H 4.975301 0.856978 -0.941369
C 3.658046 -2.597973 -1.931247
H 3.172372 -2.283067 -2.860547
H 4.660993 -2.985929 -2.141507
H 3.040806 -3.375033 -1.469748
C 4.579567 -1.787025 0.697290
H 4.681204 -0.934798 1.377049
H 3.923146 -2.531693 1.159272
H 5.560518 -2.224458 0.480609
C -3.298491 0.045493 -1.290693
C -4.067524 -0.695921 -2.174641
C -5.433873 -0.814115 -1.944426
C -6.012644 -0.203961 -0.838010
C -5.218868 0.527967 0.040062
C -3.853829 0.660755 -0.178142
H -3.602934 -1.179014 -3.033656
H -6.043778 -1.392520 -2.635057
H -7.080268 -0.303083 -0.656264
H -5.662537 0.998393 0.914788
H -3.218676 1.206951 0.513149
O -1.937258 0.184267 -1.515893
H -1.641567 -0.347922 -2.270693

V
E = -1585.88590781
C 1.175384 -1.619934 1.857066
C -0.965249 -0.851722 1.011273

C -1.418373 -0.746383 2.326485
C -0.598662 -1.063267 3.409990
C 0.692266 -1.512685 3.160373
H 2.197751 -1.966709 1.716465
H -2.428158 -0.381258 2.510867
H -0.961337 -0.964073 4.431035
H 1.344027 -1.778927 3.992032
B -1.738223 -0.472671 -0.280723
C -3.252100 -0.148395 -0.373384
C -4.220457 -0.757190 0.445304
C -3.710728 0.771442 -1.332605
C -5.570849 -0.455417 0.318963
H -3.907501 -1.499473 1.178133
C -5.053572 1.108184 -1.438563
H -2.988345 1.227953 -2.010070
C -5.989070 0.489660 -0.613684
H -6.302211 -0.952999 0.954011
H -5.377915 1.837927 -2.178709
H -7.045775 0.735806 -0.705546
C 0.374879 -1.268304 0.761366
C 0.794757 -1.212092 -0.648570
P 2.423692 -1.534109 -1.108452
C 3.668015 -0.476998 -0.312254
H 3.583617 -0.542200 0.776226
H 4.682629 -0.752454 -0.622323
H 3.455206 0.558198 -0.599557
C 2.972132 -3.245263 -0.807855
H 4.018054 -3.386455 -1.103273
H 2.856169 -3.494713 0.251349
H 2.329021 -3.914916 -1.388693
C 2.676831 -1.278317 -2.877947
H 3.716331 -1.513513 -3.132950
H 1.990503 -1.916025 -3.443195
H 2.448935 -0.236677 -3.124010
Si -0.381750 -0.527495 -1.858307
H -0.522515 2.217537 -1.312877
C 1.191510 1.944568 1.337675
C 2.459725 2.267098 1.801449
C 3.378032 2.923036 0.984132
C 3.018264 3.242081 -0.321030
C 1.754131 2.917089 -0.802221
C 0.831482 2.287977 0.033947
H 0.470011 1.432017 1.969719
H 2.730667 2.001445 2.821793
H 4.365424 3.180532 1.360801
H 3.723895 3.750514 -0.975785
H 1.469446 3.168545 -1.823933
O -0.427220 2.007352 -0.376785

4.2.6 Phenol activation transition states

Pathway A

I
E = -1615.84108205
C -0.477035 2.914776 1.014458
C 1.341702 1.459992 0.354426
C 2.211585 2.176910 1.173799
C 1.731274 3.244591 1.923880
C 0.388970 3.608208 1.851240
H -1.522849 3.217853 0.968980

H 3.260667 1.896506 1.225583
H 2.412514 3.795211 2.569369
H 0.014587 4.438890 2.446182
N 1.693112 0.375002 -0.453341
C 2.944326 -0.275475 -0.378170
C 3.685581 -0.488356 -1.540253
C 3.425612 -0.762899 0.839739
C 4.889952 -1.181049 -1.487281
H 3.307321 -0.099767 -2.484092
C 4.636667 -1.438462 0.889203
H 2.831810 -0.617865 1.739390
C 5.373511 -1.652553 -0.272751
H 5.456807 -1.344132 -2.401984
H 5.000594 -1.816802 1.842672
H 6.319107 -2.189087 -0.230231
C -0.026458 1.835770 0.243547
C -0.770025 1.018065 -0.700909
P -2.435226 1.176949 -1.064171
C -3.529226 0.897190 0.345918
H -3.230624 1.550466 1.172331
H -4.574839 1.088999 0.080127
H -3.403972 -0.143734 0.666905
C -2.880125 2.803988 -1.739411
H -3.957644 2.871315 -1.926925
H -2.582766 3.589190 -1.037263
H -2.329041 2.953462 -2.673750
C -2.914357 -0.025271 -2.320863
H -3.964076 0.124193 -2.594436
H -2.279705 0.095926 -3.205086
H -2.783177 -1.032682 -1.910784
Si 0.323718 -0.224459 -1.362232
H 0.051686 -1.754242 -1.599466
O 0.013311 -2.300756 -0.283762
C -1.127185 -2.366113 0.370616
C -2.275424 -2.978177 -0.172960
C -1.240269 -1.801159 1.659688
C -3.458781 -3.055328 0.554511
H -2.199328 -3.428325 -1.162301
C -2.417495 -1.903681 2.384623
H -0.364769 -1.304415 2.074565
C -3.541311 -2.528753 1.840870
H -4.324559 -3.549201 0.114102
H -2.465174 -1.479367 3.387003
H -4.463445 -2.606442 2.412891

II
E = -2165.03765807
C -0.305362 -2.023239 2.540223
C -0.546865 -1.544720 1.259125
C -1.577414 -0.631368 0.983730
C -2.387064 -0.240676 2.058960
C -2.159523 -0.732421 3.336206
C -1.113789 -1.616300 3.591429
H 0.519823 -2.718146 2.680775
H -3.188491 0.471680 1.882773
H -2.802488 -0.402555 4.150168
H -0.935805 -1.988485 4.597547
N -1.740746 -0.070180 -0.292361
S 0.411677 -2.198143 -0.074832
O 1.619535 -2.839920 0.488000

O -0.473710 -3.031171 -0.885443
C 0.894965 -0.781357 -0.931811
C -3.024837 0.452212 -0.623613
C -4.090972 -0.427070 -0.818150
C -3.219423 1.825551 -0.766648
C -5.343238 0.068441 -1.154867
H -3.914950 -1.495467 -0.710156
C -4.473591 2.313257 -1.121128
H -2.382774 2.497541 -0.582811
C -5.537071 1.438886 -1.312631
H -6.171681 -0.620656 -1.308039
H -4.620178 3.385618 -1.235619
H -6.518209 1.823203 -1.584292
P 2.513703 -0.804162 -1.517935
C 3.764568 -0.601261 -0.238333
H 4.770621 -0.747907 -0.647101
H 3.669188 0.403469 0.186844
H 3.550654 -1.347743 0.533044
C 2.736649 0.546059 -2.694572
H 2.593759 1.499649 -2.175586
H 3.751615 0.507261 -3.104386
H 2.003710 0.457359 -3.503060
C 2.887022 -2.343815 -2.388821
H 2.735060 -3.163609 -1.678533
H 2.182501 -2.458421 -3.218851
H 3.918325 -2.348716 -2.758691
Si -0.375074 0.425455 -1.282964
H -0.002919 1.988944 -1.484224
O 0.124858 2.259439 -0.204761
C 1.296276 2.354634 0.439502
C 1.482730 1.636140 1.630412
C 2.341136 3.155094 -0.040117
C 2.669575 1.753507 2.339183
H 0.671327 1.005465 1.988935
C 3.534314 3.250458 0.670056
H 2.188758 3.723455 -0.956532
C 3.705415 2.557796 1.864938
H 2.790763 1.200941 3.269408
H 4.333354 3.884968 0.289503
H 4.635455 2.644136 2.422442

III
E = -2927.12149679
C -2.212332 -1.385316 -0.593920
C -2.925724 -0.637609 0.354523
C -4.260892 -0.927132 0.616943
C -4.902726 -1.957645 -0.059584
C -4.202534 -2.702431 -0.997968
C -2.867994 -2.414117 -1.262723
H -4.783307 -0.326554 1.357062
H -5.947066 -2.175692 0.153125
H -4.693284 -3.511918 -1.534667
H -2.304006 -2.983460 -1.997795
S -0.500343 -1.099268 -1.163262
S -2.216933 0.706480 1.331255
O -0.131359 -2.352439 -1.856678
O -0.607421 0.096225 -2.012138
O -1.238119 -0.032870 2.294821
O -3.358729 1.392424 1.927102
C 0.505872 -0.923086 0.185711

C -1.047955 1.545269 0.544634
P -1.294605 2.790274 -0.603933
P 1.577034 -2.246651 0.471507
C 0.211663 3.046193 -1.535036
H 0.141764 3.961071 -2.132255
H 1.039020 3.095343 -0.810359
H 0.357630 2.163162 -2.165093
C -2.685943 2.411661 -1.691475
H -3.571861 2.240044 -1.067422
H -2.881476 3.237647 -2.383738
H -2.433130 1.496648 -2.236257
C -1.726819 4.336303 0.232302
H -0.877668 4.644795 0.851239
H -1.979373 5.130182 -0.479741
H -2.580083 4.128554 0.888340
C 2.401972 -1.980316 2.053729
H 3.061406 -2.830674 2.259084
H 2.995211 -1.062297 2.001820
H 1.654899 -1.890810 2.849992
C 0.758065 -3.855507 0.635649
H 0.255241 -4.086126 -0.306674
H 1.494500 -4.631481 0.873340
H 0.017456 -3.788128 1.439833
C 2.866788 -2.425707 -0.772243
H 3.509469 -3.287064 -0.558781
H 2.368860 -2.548699 -1.739218
H 3.459081 -1.503174 -0.779268
Si 0.309085 0.516090 1.309546
H 1.637154 1.391708 1.592341
O 2.365156 2.151869 0.686114
C 3.415375 1.498885 0.255146
C 4.336600 0.902204 1.146745
C 3.680836 1.349788 -1.126365
C 5.453416 0.214748 0.683953
H 4.168459 1.033082 2.215475
C 4.807834 0.678993 -1.576497
H 2.980563 1.786158 -1.835652
C 5.705639 0.096924 -0.680012
H 6.146651 -0.221568 1.403217
H 4.985929 0.598790 -2.648642
H 6.587679 -0.428833 -1.039280

IV
E = -2135.08344927
C 0.304493 -0.269213 3.023089
C -0.406830 -0.490015 1.856755
C -1.294529 0.405806 1.256554
C -1.442701 1.640475 1.889048
C -0.750095 1.904590 3.069240
C 0.106270 0.961548 3.643783
H 0.976215 -1.021819 3.432283
H -2.112226 2.389920 1.470193
H -0.882637 2.866131 3.562178
H 0.621398 1.190811 4.574218
B -1.910026 -0.230235 -0.089100
S -0.216468 -1.809690 0.705876
O 0.252023 -3.078507 1.260388
O -1.679768 -1.768394 0.177475
C 0.683662 -1.203626 -0.604426
Si -0.372366 0.022410 -1.492286

C -3.392736 0.098680 -0.506099
C -4.418791 -0.848004 -0.422697
C -3.728621 1.384917 -0.950145
C -5.728424 -0.524684 -0.764565
H -4.181377 -1.855663 -0.083534
C -5.036182 1.717210 -1.285228
H -2.944536 2.138414 -1.047790
C -6.043204 0.760464 -1.193532
H -6.509860 -1.280263 -0.693468
H -5.270833 2.724404 -1.627078
H -7.067703 1.015251 -1.459906
P 2.262100 -1.850752 -0.814953
C 3.226300 -1.894295 0.714901
H 4.227801 -2.297595 0.528575
H 3.300679 -0.872962 1.102229
H 2.699023 -2.528889 1.435005
C 3.174525 -0.853122 -2.000650
H 3.441798 0.102862 -1.537207
H 4.081272 -1.385079 -2.308586
H 2.537589 -0.660428 -2.870942
C 2.230447 -3.556701 -1.417954
H 1.632110 -4.133600 -0.703679
H 1.733990 -3.577187 -2.393800
H 3.237841 -3.980419 -1.498274
C 3.956652 2.849680 -1.311406
C 4.407825 2.436832 -0.059990
C 3.495477 1.902418 0.848043
C 2.156381 1.756096 0.507305
C 1.703816 2.164264 -0.753305
C 2.617368 2.718934 -1.659191
H 4.655491 3.281382 -2.026143
H 5.455737 2.545987 0.210915
H 3.829546 1.597567 1.839412
H 1.434549 1.344729 1.206928
H 2.257146 3.046328 -2.632556
O 0.418153 1.994071 -1.099907
H 0.292326 1.352995 -2.192007

V
E = -1585.85963685
C 0.963045 2.813971 0.812981
C -1.106541 1.663873 0.260137
C -1.793755 2.585848 1.059844
C -1.133704 3.600467 1.742798
C 0.250018 3.693416 1.620521
H 2.042203 2.940359 0.742013
H -2.877599 2.509080 1.136750
H -1.683032 4.306623 2.361413
H 0.788716 4.474008 2.156996
B -1.743386 0.502097 -0.527675
C -3.160302 -0.093536 -0.390004
C -3.885958 -0.060359 0.815334
C -3.762984 -0.732129 -1.488612
C -5.152876 -0.619710 0.912253
H -3.433418 0.388509 1.697752
C -5.041968 -1.265580 -1.407664
H -3.207507 -0.805009 -2.423352
C -5.738486 -1.212118 -0.203657
H -5.688865 -0.595635 1.859524
H -5.491928 -1.741653 -2.277078

H -6.734895 -1.645072 -0.130638
C 0.313197 1.793318 0.099247
C 0.955782 0.850020 -0.816835
P 2.647597 0.805569 -1.092272
C 3.344791 2.326642 -1.816823
H 3.116103 3.186092 -1.179397
H 4.430579 2.245131 -1.941765
H 2.870616 2.488331 -2.790685
C 3.657046 0.459520 0.373911
H 4.725816 0.539110 0.144703
H 3.399663 1.153974 1.179587
H 3.419540 -0.555976 0.709397
C 3.077213 -0.496210 -2.267126
H 4.150708 -0.452485 -2.481179
H 2.825299 -1.466631 -1.827931
H 2.500938 -0.365398 -3.188474
Si -0.215214 -0.252816 -1.649818
H 0.195098 -1.839066 -1.731539
O -0.115536 -2.201972 -0.532078
C 0.849945 -2.301582 0.384799
C 1.943305 -3.161496 0.202859
C 0.775560 -1.549335 1.568383
C 2.904053 -3.300435 1.199615
H 1.999597 -3.746265 -0.714067
C 1.729264 -1.707490 2.563291
H -0.054487 -0.854923 1.687818
C 2.800417 -2.583692 2.388703
H 3.735768 -3.987326 1.049234
H 1.641599 -1.131338 3.483171
H 3.547381 -2.703669 3.170553

Pathway B

I
E = -1615.86250014
C 2.102173 0.788462 -2.151222
C 0.053680 1.112282 -0.906400
C -0.543450 1.655429 -2.043716
C 0.184792 1.765506 -3.225358
C 1.505479 1.338875 -3.284735
H 3.135682 0.447683 -2.212082
H -1.570931 2.006299 -1.999787
H -0.289030 2.197380 -4.104946
H 2.073200 1.428185 -4.208576
N -0.532210 1.010845 0.355131
C -1.899739 1.314049 0.559845
C -2.268642 2.244094 1.533416
C -2.898023 0.655579 -0.164871
C -3.611232 2.504729 1.784974
H -1.488229 2.756790 2.092303
C -4.236052 0.931416 0.079328
H -2.611197 -0.090448 -0.903107
C -4.600383 1.854568 1.056160
H -3.883406 3.228347 2.551398
H -5.000893 0.404317 -0.488381
H -5.650822 2.062828 1.249889
C 1.395536 0.663168 -0.955888
P 3.499290 -0.091204 0.733459
C 4.400833 -1.338573 -0.232672

H 4.402845 -1.070083 -1.293269
H 5.434627 -1.431526 0.119308
H 3.885387 -2.298504 -0.119829
C 4.464302 1.435169 0.574114
H 5.526159 1.270490 0.787428
H 4.346768 1.815106 -0.447037
H 4.054925 2.178694 1.266080
C 3.631151 -0.647158 2.446485
H 4.675389 -0.849665 2.706475
H 3.217403 0.117467 3.112181
H 3.033690 -1.559116 2.561450
C 1.839852 0.073595 0.320612
H 1.193884 -1.141537 0.466586
Si 0.467114 0.336064 1.685459
O 0.288734 -1.749674 1.041552
C -0.766131 -2.162999 0.314573
C -1.956597 -2.513869 0.959778
C -0.704802 -2.223789 -1.082679
C -3.061508 -2.911572 0.219506
H -1.995464 -2.455606 2.045170
C -1.815517 -2.628558 -1.813715
H 0.216148 -1.937385 -1.588052
C -3.001272 -2.972197 -1.170660
H -3.983969 -3.172867 0.735673
H -1.752623 -2.666355 -2.900103
H -3.869581 -3.285093 -1.746672

II
E = -2165.06210801
C 0.124938 1.501972 2.614273
C 0.162001 0.487959 1.663245
C -0.974248 0.082205 0.950050
C -2.184222 0.719723 1.262695
C -2.231570 1.719605 2.218671
C -1.079621 2.127142 2.893615
H 1.049889 1.768969 3.120917
H -3.079789 0.433936 0.716250
H -3.181593 2.208675 2.425600
H -1.126079 2.921328 3.635142
N -0.899002 -0.868313 -0.074967
S 1.665093 -0.393471 1.404095
O 2.766643 0.322900 2.080808
O 1.468515 -1.795846 1.784781
C -1.975445 -1.791891 -0.146933
C -2.295167 -2.551161 0.983461
C -2.701714 -1.980080 -1.322948
C -3.335594 -3.467223 0.938035
H -1.697032 -2.418302 1.882996
C -3.731512 -2.914548 -1.367869
H -2.454409 -1.387572 -2.200959
C -4.058059 -3.655352 -0.238383
H -3.574479 -4.052658 1.824160
H -4.289455 -3.052842 -2.292226
H -4.869527 -4.379436 -0.273410
C 1.851885 -0.250372 -0.316678
Si 0.352985 -0.974339 -1.371101
O 0.380678 1.093820 -1.720822
C -0.623049 1.974513 -1.492756
C -0.416521 3.074595 -0.659458
C -1.876716 1.759842 -2.065159

C -1.466425 3.942521 -0.390666
H 0.562202 3.223102 -0.206964
C -2.920513 2.632643 -1.784509
H -2.018086 0.903092 -2.720272
C -2.724333 3.724980 -0.944778
H -1.300118 4.791395 0.270106
H -3.898876 2.454556 -2.227607
H -3.545715 4.403846 -0.726239
H 1.223385 0.895095 -0.919987
P 3.505282 -0.480861 -0.793307
C 3.558179 -0.748295 -2.576539
H 4.596041 -0.798890 -2.922383
H 3.031791 -1.676753 -2.821913
H 3.040354 0.081945 -3.070812
C 4.267149 -1.895573 0.030722
H 5.302395 -2.042534 -0.296096
H 4.230818 -1.701630 1.108898
H 3.666235 -2.787096 -0.176198
C 4.528965 0.960099 -0.429487
H 4.160643 1.810340 -1.013309
H 4.403748 1.172472 0.637589
H 5.581039 0.769523 -0.668761

III
E = -2927.12121346
C 2.796486 -0.282000 0.142728
C 2.378161 0.600033 -0.854888
C 3.322577 1.169586 -1.708321
C 4.672815 0.878134 -1.564669
C 5.090275 0.010730 -0.563088
C 4.153663 -0.561814 0.287226
H 2.973083 1.850864 -2.480065
H 5.396049 1.331068 -2.239826
H 6.146202 -0.221999 -0.439920
H 4.448429 -1.248818 1.076642
S 1.708484 -1.050468 1.378193
S 0.665033 1.013780 -1.303077
O 2.512458 -2.187014 1.858821
O 1.407335 0.067016 2.315143
O 0.172362 -0.127119 -2.083264
O 0.809557 2.306773 -2.007791
C 0.254097 -1.482534 0.697568
P -0.064212 2.856074 0.789701
P 0.199218 -2.893394 -0.259451
C -0.762608 2.887240 2.450897
H -0.622127 3.885308 2.881147
H -1.826366 2.632286 2.421186
H -0.241108 2.131706 3.050401
C 1.691807 3.223612 0.997978
H 2.158184 3.305495 0.011520
H 1.819965 4.158373 1.554659
H 2.129768 2.383482 1.551072
C -0.802983 4.216295 -0.141915
H -1.887455 4.065872 -0.179077
H -0.576641 5.183957 0.319828
H -0.397082 4.163853 -1.157011
C -1.304632 -2.946476 -1.244601
H -1.307207 -3.854648 -1.856864
H -2.179492 -2.940170 -0.586340
H -1.325635 -2.044991 -1.864683

C 1.597542 -3.014545 -1.402210
H 2.520556 -3.024950 -0.811351
H 1.532477 -3.929977 -2.000769
H 1.576867 -2.128361 -2.044658
C 0.270913 -4.412531 0.728605
H 0.333652 -5.311816 0.104964
H 1.157452 -4.323125 1.366976
H -0.616175 -4.460770 1.369034
C -0.335281 1.286676 0.078220
H -1.562757 0.762485 -0.457571
Si -0.984612 -0.162574 1.284589
O -2.209210 -0.228641 -0.349695
C -3.558851 -0.191665 -0.311802
C -4.258826 0.626763 -1.200512
C -4.258410 -0.986787 0.597978
C -5.648076 0.640915 -1.177752
H -3.700230 1.231294 -1.912424
C -5.647614 -0.967498 0.606608
H -3.698721 -1.601172 1.301128
C -6.350641 -0.154999 -0.277872
H -6.186942 1.278949 -1.876273
H -6.185757 -1.591700 1.318003
H -7.438292 -0.142563 -0.266376

IV
E = -2135.09967690
C -2.991658 2.385509 -0.944619
C -1.816157 1.680410 -0.740786
C -0.717003 2.114022 0.007889
C -0.859546 3.357735 0.632647
C -2.025995 4.096008 0.459248
C -3.084356 3.627995 -0.327471
H -3.788469 1.991065 -1.573334
H -0.044537 3.757294 1.234883
H -2.114976 5.071955 0.934093
H -3.974085 4.239272 -0.462620
B 0.425852 0.997937 0.015447
S -1.438356 0.045536 -1.260373
O -2.134087 -0.396818 -2.463654
O 0.095710 0.219408 -1.340987
C -1.550580 -0.986193 0.110644
Si -0.285974 -0.316013 1.543291
C 1.955458 1.364280 0.090966
C 2.826292 1.139960 -0.981530
C 2.502545 1.896561 1.267382
C 4.182436 1.430555 -0.883931
H 2.432385 0.710876 -1.901613
C 3.856546 2.194221 1.370046
H 1.856091 2.058331 2.132260
C 4.705361 1.958669 0.292211
H 4.838999 1.234465 -1.730709
H 4.254566 2.603276 2.297994
H 5.768203 2.181917 0.370788
P -3.074581 -1.678046 0.545766
C -4.213654 -0.486200 1.287061
H -5.144939 -0.967558 1.605436
H -3.707578 -0.034674 2.148404
H -4.435058 0.305414 0.562863
C -2.749730 -2.943808 1.785186
H -2.272579 -2.459540 2.645158

H -3.680888 -3.435777 2.086252
H -2.049633 -3.675381 1.368168
C -3.935170 -2.454473 -0.840466
H -4.084218 -1.708848 -1.627905
H -3.297846 -3.247557 -1.245242
H -4.895308 -2.872692 -0.519019
C 2.923983 -1.718883 1.117843
C 1.843170 -2.019515 0.289901
C 2.056283 -2.377930 -1.038717
C 3.354260 -2.439037 -1.532876
C 4.437218 -2.139623 -0.714393
C 4.214069 -1.774555 0.610463
H 2.736995 -1.421135 2.147609
H 1.201734 -2.582618 -1.680159
H 3.516327 -2.714306 -2.573647
H 5.450190 -2.178080 -1.109058
H 5.052655 -1.518916 1.255152
O 0.579787 -1.958521 0.793029
H -0.425161 -1.806962 0.123141

V
E = -1585.87884112
C 0.057744 3.336027 -0.668867
C 1.650480 1.573941 -0.150737
C 2.678857 2.414313 -0.601280
C 2.421038 3.693753 -1.072954
C 1.101393 4.142362 -1.104760
H -0.955027 3.732734 -0.720577
H 3.707125 2.056973 -0.550953
H 3.230400 4.341164 -1.403937
H 0.879780 5.143119 -1.474565
B 1.815876 0.158404 0.439529
C 3.064651 -0.747360 0.299247
C 3.884267 -0.725308 -0.844692
C 3.397061 -1.667117 1.310126
C 4.979186 -1.570603 -0.969021
H 3.639169 -0.045446 -1.659627
C 4.509139 -2.492742 1.206246
H 2.765588 -1.724755 2.196675
C 5.301185 -2.448846 0.062448
H 5.588614 -1.544466 -1.871081
H 4.752832 -3.184457 2.011064
H 6.165087 -3.105323 -0.029349
C 0.299927 2.038415 -0.183731
C -0.712913 1.088455 0.303973
P -2.321998 1.610089 0.657056
C -2.434192 3.022490 1.800498
H -1.834106 3.854986 1.419859
H -3.470946 3.348355 1.940467
H -2.013338 2.708201 2.761934
C -3.312664 2.075843 -0.797250
H -2.822283 2.871127 -1.365743
H -3.395167 1.190300 -1.437022
H -4.314521 2.404521 -0.498358
C -3.286521 0.298337 1.439376
H -4.277024 0.687610 1.700760
H -3.393741 -0.547705 0.751943
H -2.760947 -0.048903 2.335132
Si 0.059750 -0.362138 1.363192
O -0.436638 -1.302302 -0.459828

C -1.550881 -2.058318 -0.613080
C -2.419130 -1.787123 -1.673221
C -1.860875 -3.063853 0.302081
C -3.597825 -2.513316 -1.805863
H -2.148521 -1.014911 -2.391397
C -3.036646 -3.788671 0.152737
H -1.178895 -3.247877 1.129206
C -3.914300 -3.514956 -0.893729
H -4.270593 -2.296595 -2.633981
H -3.273880 -4.572882 0.869566
H -4.835273 -4.083859 -1.000538
H -0.691014 -0.211247 -0.524310

Pathway C

IV
E = -2135.06325820
C 1.747035 -1.625770 2.552564
C 1.181991 -1.106413 1.393520
C -0.139779 -0.638741 1.316108
C -0.860060 -0.674153 2.516600
C -0.316872 -1.181647 3.692866
C 0.984138 -1.671378 3.712913
H 2.777182 -1.973370 2.532317
H -1.877736 -0.287427 2.529405
H -0.916360 -1.193976 4.601746
H 1.410011 -2.074954 4.629441
B -0.788630 -0.066817 -0.040697
S 2.249591 -1.067697 -0.047047
O 3.634694 -1.314098 0.421655
O 1.693228 -1.956390 -1.060817
C 2.216434 0.605003 -0.608587
Si 0.561807 1.169971 -0.895231
C -1.574807 -1.061189 -0.994020
C -1.487791 -2.450480 -0.850052
C -2.373081 -0.572426 -2.039671
C -2.167733 -3.312251 -1.704517
H -0.861465 -2.864379 -0.061146
C -3.061821 -1.425506 -2.892880
H -2.466369 0.505660 -2.183677
C -2.962173 -2.803919 -2.726673
H -2.076898 -4.389281 -1.570719
H -3.678443 -1.015029 -3.691407
H -3.498954 -3.477491 -3.392807
P 3.758818 1.388071 -0.669460
C 4.526568 1.604316 0.951237
H 5.501559 2.095441 0.858730
H 3.860264 2.204992 1.578965
H 4.638459 0.607188 1.387835
C 3.517208 3.034975 -1.368198
H 2.802501 3.589029 -0.750239
H 4.471158 3.572024 -1.403123
H 3.108522 2.942953 -2.379807
C 4.952182 0.528028 -1.716208
H 5.069004 -0.480356 -1.306023
H 4.545047 0.459978 -2.730337
H 5.913917 1.052744 -1.729289
C -2.949462 1.476770 0.404260
C -3.436660 2.771033 0.233539
C -4.796023 3.019554 0.373876

C -5.673684 1.985574 0.682799
C -5.176613 0.697298 0.849661
C -3.819745 0.431730 0.710484
H -2.741677 3.573456 -0.006669
H -5.169931 4.032956 0.239660
H -6.738018 2.182133 0.790421
H -5.852886 -0.122108 1.085737
H -3.433491 -0.577424 0.819837
O -1.606485 1.265739 0.283768
H -0.830268 2.109359 -0.412062

V
E = -1585.85623114
C -1.285923 2.324730 1.334845
C 0.706043 1.092425 0.667796
C 1.343642 1.571651 1.812759
C 0.681057 2.387445 2.727899
C -0.639673 2.754136 2.489079
H -2.302611 2.669142 1.144028
H 2.376512 1.277739 2.001602
H 1.190005 2.732009 3.626521
H -1.164506 3.398163 3.193244
B 1.388688 0.157737 -0.421579
C 2.951985 -0.009921 -0.495683
C 3.770334 1.131157 -0.467368
C 3.597535 -1.249662 -0.594409
C 5.154910 1.041372 -0.534296
H 3.304611 2.114542 -0.393018
C 4.983925 -1.348816 -0.663248
H 2.999517 -2.159490 -0.615485
C 5.771237 -0.203376 -0.633248
H 5.758974 1.947623 -0.510403
H 5.453073 -2.329221 -0.738280
H 6.856181 -0.278221 -0.686174
C -0.626545 1.507111 0.405770
C -1.175037 1.021198 -0.869659
P -2.870597 0.791205 -1.070727
C -3.648185 0.016817 0.371551
H -3.505013 0.655150 1.248448
H -4.718503 -0.144258 0.201439
H -3.147290 -0.939069 0.558735
C -3.837905 2.301654 -1.389063
H -4.903224 2.074021 -1.511203
H -3.709230 3.004692 -0.560005
H -3.452555 2.771722 -2.300180
C -3.226879 -0.282703 -2.478286
H -4.308970 -0.345598 -2.637394
H -2.739189 0.116822 -3.373746
H -2.812833 -1.275012 -2.276030
Si 0.072993 0.407310 -2.029038
H 0.207517 -1.319028 -1.659941
C -0.245910 -1.658551 1.655678
C -1.184295 -2.311840 2.448151
C -2.096131 -3.204108 1.893084
C -2.062252 -3.452325 0.523797
C -1.135614 -2.801502 -0.281298
C -0.227247 -1.909064 0.288427
H 0.468941 -0.968274 2.089130
H -1.193970 -2.118640 3.518954
H -2.822662 -3.710406 2.524809

H -2.758177 -4.159380 0.076167
H -1.090275 -2.993196 -1.352277
O 0.711606 -1.335987 -0.533410

4.2.7 Phenol activated products

Pathway A

I
E = -1615.95541074
C 0.958920 -2.802720 0.999070
C -1.015612 -1.596128 0.302535
C -1.782966 -2.352482 1.180000
C -1.179536 -3.325379 1.978086
C 0.188591 -3.541849 1.895454
H 2.028309 -3.003704 0.935429
H -2.855808 -2.187572 1.239750
H -1.789883 -3.911582 2.662014
H 0.664492 -4.294973 2.520697
N -1.500173 -0.589713 -0.551531
C -2.799546 -0.051933 -0.463480
C -3.546767 0.143633 -1.628059
C -3.342491 0.352403 0.761605
C -4.803013 0.735025 -1.570213
H -3.130156 -0.178186 -2.580331
C -4.604803 0.927045 0.815172
H -2.760575 0.216794 1.670794
C -5.341631 1.124402 -0.349209
H -5.368925 0.881960 -2.488434
H -5.011544 1.236212 1.776464
H -6.328588 1.580048 -0.303832
C 0.384735 -1.828436 0.176783
C 1.038568 -0.988358 -0.820595
P 2.717461 -0.785121 -0.958832
C 3.557387 -0.241814 0.559061
H 3.244167 -0.891404 1.383835
H 4.647762 -0.277217 0.454684
H 3.233018 0.780204 0.786869
C 3.636251 -2.262356 -1.496731
H 4.709301 -2.054620 -1.578525
H 3.479589 -3.075933 -0.781390
H 3.240845 -2.579871 -2.467162
C 3.072384 0.475113 -2.204940
H 4.153862 0.594147 -2.326699
H 2.627003 0.174213 -3.159268
H 2.633876 1.426719 -1.886364
Si -0.194155 0.155586 -1.462922
H -0.514119 0.343640 -2.894340
O 0.100986 1.780485 -1.046704
C 0.709277 2.162198 0.097969
C 1.634718 3.207268 0.039015
C 0.465841 1.530416 1.322223
C 2.312560 3.608386 1.184726
H 1.801166 3.697478 -0.918304
C 1.147523 1.940746 2.461342
H -0.250567 0.713802 1.375012
C 2.077533 2.974868 2.401708
H 3.029541 4.425542 1.124482
H 0.950395 1.437076 3.405982
H 2.608874 3.289669 3.297232

II
E = -2165.15463655
C 3.503001 1.263985 -1.299697
C 2.387947 1.001556 -0.515901
C 1.163569 1.655019 -0.725384
C 1.108311 2.607084 -1.749584
C 2.226702 2.883869 -2.523801
C 3.426799 2.210313 -2.313126
H 4.422065 0.722783 -1.084337
H 0.170554 3.126850 -1.931566
H 2.153622 3.629987 -3.312989
H 4.297979 2.427491 -2.926998
N 0.031711 1.355910 0.048814
S 2.534036 -0.111541 0.862516
O 3.774081 -0.891344 0.661988
O 2.401585 0.703822 2.067969
C 1.184745 -1.154114 0.672902
C -0.864604 2.414500 0.347372
C -0.398306 3.553442 1.010653
C -2.217381 2.311469 0.025039
C -1.278552 4.573286 1.339422
H 0.655842 3.609077 1.274909
C -3.099385 3.329506 0.377352
H -2.572525 1.438448 -0.518724
C -2.633448 4.463882 1.029427
H -0.908484 5.455449 1.858686
H -4.154029 3.235418 0.125312
H -3.321480 5.263085 1.297442
P 1.536611 -2.790117 0.315040
C 2.420597 -3.036240 -1.245890
H 2.658899 -4.093266 -1.408746
H 1.798039 -2.658798 -2.063799
H 3.340742 -2.445688 -1.187493
C 0.013269 -3.753738 0.203045
H -0.591466 -3.405332 -0.638315
H 0.270178 -4.810245 0.070445
H -0.566369 -3.633511 1.124299
C 2.548672 -3.585701 1.586106
H 3.462103 -2.990680 1.688851
H 1.998549 -3.567460 2.532647
H 2.790573 -4.618089 1.309227
Si -0.370191 -0.246697 0.652028
H -1.124532 -0.014992 1.900897
O -1.396248 -1.090013 -0.390555
C -2.709024 -1.402106 -0.430590
C -3.144727 -2.191420 -1.497851
C -3.629961 -0.979668 0.531766
C -4.479550 -2.559998 -1.596766
H -2.417494 -2.493607 -2.248828
C -4.964409 -1.354922 0.420381
H -3.302844 -0.356255 1.359615
C -5.398714 -2.145936 -0.637501
H -4.804314 -3.173742 -2.435174
H -5.672642 -1.018664 1.175656
H -6.444669 -2.433567 -0.716391

III
E = -2927.23442788
C -1.903928 -1.145151 -0.961317
C -2.641018 -0.495707 0.035256

C -4.020040 -0.691755 0.098634
C -4.669170 -1.503899 -0.821840
C -3.939020 -2.133037 -1.821764
C -2.564098 -1.951441 -1.887714
H -4.572081 -0.175927 0.880306
H -5.746631 -1.641048 -0.754983
H -4.435575 -2.767905 -2.552864
H -1.967516 -2.444266 -2.651404
S -0.112942 -0.999333 -1.254262
S -1.982067 0.564877 1.367341
O 0.207984 -2.247284 -1.974602
O 0.052980 0.278793 -1.971559
O -1.444813 -0.378507 2.366326
O -3.148162 1.392672 1.724970
C 0.708025 -0.964710 0.217734
C -0.730787 1.528942 0.780082
P -1.192197 2.863771 -0.199940
P 0.926254 -2.482773 0.987349
C 0.201983 3.510906 -1.132126
H -0.144575 4.354470 -1.739029
H 0.995727 3.826186 -0.450353
H 0.585273 2.702308 -1.762688
C -2.455541 2.432022 -1.420653
H -3.347106 2.074965 -0.894686
H -2.709088 3.307578 -2.028557
H -2.045482 1.632863 -2.048456
C -1.891485 4.218127 0.775142
H -1.118505 4.601056 1.449666
H -2.269380 5.025678 0.137867
H -2.700749 3.785711 1.373696
C 1.412408 -2.227975 2.702738
H 1.535348 -3.200209 3.191722
H 2.352579 -1.669830 2.746966
H 0.620569 -1.648980 3.191842
C -0.591428 -3.464154 1.041706
H -0.911320 -3.668834 0.014568
H -0.427923 -4.406372 1.576224
H -1.348513 -2.856004 1.550906
C 2.186084 -3.507828 0.193948
H 2.275935 -4.488917 0.673875
H 1.886530 -3.613713 -0.854200
H 3.142280 -2.974592 0.233539
Si 0.877455 0.689932 0.965933
H 1.369476 0.559199 2.358473
O 2.027354 1.642766 0.170313
C 3.261372 1.174186 -0.149346
C 4.354575 1.503925 0.651147
C 3.439086 0.373536 -1.279686
C 5.621920 1.036363 0.320218
H 4.192723 2.130476 1.525739
C 4.709880 -0.089306 -1.598639
H 2.569301 0.126373 -1.883073
C 5.805640 0.236626 -0.803002
H 6.472085 1.300618 0.947056
H 4.843804 -0.711524 -2.482120
H 6.798240 -0.127614 -1.060201

IV
E = -2135.20117430
C -0.273828 0.485785 3.036678

C 0.420864 0.623128 1.846799
C 1.315276 -0.306819 1.313475
C 1.498718 -1.477628 2.049808
C 0.822601 -1.655958 3.255063
C -0.048709 -0.684808 3.755055
H -0.952205 1.258839 3.393003
H 2.180111 -2.246464 1.688346
H 0.980756 -2.568734 3.826942
H -0.551210 -0.844276 4.706852
B 1.874977 0.205648 -0.114098
S 0.175045 1.839202 0.591797
O -0.294722 3.138820 1.067802
O 1.622666 1.765465 0.024403
C -0.764442 1.135979 -0.619561
Si 0.293819 -0.210849 -1.366609
C 3.361976 -0.121862 -0.525117
C 4.358207 0.859246 -0.556420
C 3.733264 -1.434762 -0.845756
C 5.672035 0.543848 -0.889394
H 4.093446 1.887804 -0.314499
C 5.045313 -1.758647 -1.171755
H 2.974192 -2.219440 -0.851196
C 6.021996 -0.767219 -1.194978
H 6.429291 1.326703 -0.908370
H 5.306943 -2.787262 -1.416451
H 7.049864 -1.015345 -1.454550
P -2.340061 1.763623 -0.841850
C -3.305408 1.866986 0.686180
H -4.305583 2.269271 0.490509
H -3.383692 0.861332 1.112802
H -2.772708 2.526217 1.380079
C -3.258460 0.711105 -1.978734
H -3.516302 -0.229263 -1.479885
H -4.172497 1.225104 -2.295284
H -2.636776 0.494574 -2.854282
C -2.340316 3.440601 -1.524128
H -1.742078 4.059530 -0.846259
H -1.855300 3.421578 -2.505738
H -3.355074 3.845034 -1.612162
C -3.762413 -3.077505 -1.240575
C -4.248995 -2.698863 0.008221
C -3.403373 -2.036912 0.895272
C -2.095444 -1.732303 0.535218
C -1.619563 -2.084461 -0.732234
C -2.457082 -2.775995 -1.612090
H -4.407308 -3.611816 -1.936362
H -5.271349 -2.935718 0.294863
H -3.761109 -1.762546 1.887020
H -1.421153 -1.231452 1.225654
H -2.065469 -3.057396 -2.587065
O -0.374602 -1.761957 -1.114138
H 0.307016 -0.080451 -2.852666

V
E = -1585.97718445
C -0.969725 -2.861668 0.812707
C 1.067997 -1.648371 0.228684
C 1.785286 -2.561480 1.026029
C 1.164976 -3.588274 1.712724
C -0.225591 -3.713649 1.607307

H -2.045797 -3.017499 0.753555
H 2.867577 -2.455953 1.087902
H 1.738072 -4.285700 2.319291
H -0.736250 -4.507865 2.151724
B 1.683040 -0.454176 -0.504837
C 3.131110 0.087692 -0.409618
C 3.880474 0.049054 0.780160
C 3.738577 0.679880 -1.531230
C 5.170879 0.559715 0.841852
H 3.429663 -0.364043 1.680876
C 5.040099 1.161633 -1.485784
H 3.174162 0.755063 -2.460625
C 5.759280 1.105325 -0.295352
H 5.722772 0.531806 1.779968
H 5.491064 1.599029 -2.374926
H 6.773768 1.498053 -0.250778
C -0.360302 -1.818740 0.075327
C -1.059852 -0.916428 -0.806133
P -2.753477 -0.806491 -0.940339
C -3.610808 -2.288891 -1.570305
H -3.434727 -3.142622 -0.909133
H -4.689586 -2.112028 -1.650901
H -3.199304 -2.528714 -2.556393
C -3.627009 -0.382251 0.598329
H -4.714705 -0.427539 0.471102
H -3.320165 -1.077636 1.387075
H -3.324975 0.628641 0.896230
C -3.180812 0.492755 -2.120767
H -4.267693 0.547378 -2.240760
H -2.799862 1.451954 -1.757031
H -2.712342 0.267251 -3.084629
Si 0.105143 0.321377 -1.502366
H 0.123723 0.471508 -2.985147
O -0.394468 1.908727 -1.058197
C -0.943744 2.214021 0.130253
C -1.934629 3.200481 0.171303
C -0.587015 1.559508 1.314946
C -2.562441 3.515401 1.370836
H -2.191835 3.713038 -0.753762
C -1.217774 1.883893 2.509254
H 0.182521 0.788598 1.293298
C -2.213615 2.856132 2.546379
H -3.331658 4.285900 1.385809
H -0.928957 1.362331 3.420077
H -2.706929 3.103767 3.483752

Pathway B

I
E = -1615.91712301
C 2.060288 0.518485 1.687218
C -0.245393 0.079406 1.031285
C -0.664622 0.608302 2.262471
C 0.267967 1.130326 3.151921
C 1.628887 1.125777 2.863916
H 3.123799 0.472322 1.451404
H -1.719366 0.633482 2.516624
H -0.084481 1.561927 4.087675
H 2.344314 1.570736 3.551345

N -1.041725 -0.393128 0.005609
C -2.446351 -0.351585 0.038089
C -3.164004 -1.459009 -0.435217
C -3.174331 0.769795 0.461248
C -4.551567 -1.449703 -0.477668
H -2.607687 -2.326395 -0.784740
C -4.563357 0.769119 0.429341
H -2.644974 1.654324 0.804568
C -5.262884 -0.338257 -0.038100
H -5.081358 -2.323783 -0.852778
H -5.103511 1.653837 0.762603
H -6.350547 -0.332461 -0.064916
C 1.146379 -0.026265 0.793948
P 1.836507 -2.475261 -0.057562
C 3.206061 -2.682470 1.104981
H 2.972157 -2.130623 2.021920
H 3.358749 -3.741952 1.339242
H 4.121499 -2.268918 0.667812
C 0.391328 -3.234606 0.702516
H 0.618565 -4.259206 1.014927
H 0.096742 -2.630666 1.568478
H -0.429032 -3.227325 -0.024025
C 2.242647 -3.396033 -1.552548
H 2.409893 -4.454189 -1.325726
H 1.402039 -3.279264 -2.247653
H 3.143429 -2.970876 -2.008580
C 1.490223 -0.782524 -0.454622
H 2.349006 -0.379642 -1.008600
Si -0.184430 -0.778313 -1.625326
O 0.065856 0.815325 -2.277748
C 0.604909 1.880169 -1.659229
C -0.165005 2.688379 -0.817499
C 1.956952 2.189172 -1.843459
C 0.420340 3.742331 -0.129491
H -1.220859 2.455390 -0.708860
C 2.534771 3.249624 -1.155381
H 2.537879 1.580297 -2.534181
C 1.775193 4.023814 -0.283835
H -0.189038 4.350315 0.537399
H 3.590354 3.474068 -1.303362
H 2.231484 4.847710 0.260828

II
E = -2165.11660870
C 1.430456 -1.781723 2.162213
C 0.577111 -1.036079 1.356899
C 0.882771 0.260565 0.922532
C 2.078116 0.822670 1.387088
C 2.925791 0.098317 2.210463
C 2.620852 -1.210763 2.585959
H 1.133549 -2.786737 2.452941
H 2.344902 1.823163 1.054698
H 3.858746 0.550966 2.540763
H 3.306023 -1.777946 3.211418
N 0.081954 0.887482 -0.035606
S -1.051576 -1.641810 1.087402
O -1.108597 -3.056419 1.452845
O -1.975422 -0.708311 1.767953
C -0.302282 2.218326 0.173893
C -0.679745 2.645197 1.456389

C -0.375933 3.134090 -0.884094
C -1.100814 3.950150 1.670160
H -0.657640 1.925793 2.272901
C -0.817901 4.433129 -0.662229
H -0.080136 2.815833 -1.881710
C -1.177933 4.853995 0.613684
H -1.386999 4.259941 2.674099
H -0.863571 5.129153 -1.498436
H -1.513523 5.874766 0.783972
C -1.425711 -1.448825 -0.646368
Si -0.312284 -0.009404 -1.607284
O 1.035784 -1.115403 -1.590209
C 2.334037 -0.787326 -1.506819
C 3.232151 -1.746275 -1.028702
C 2.810289 0.485790 -1.842882
C 4.571917 -1.425992 -0.862828
H 2.844185 -2.727783 -0.766099
C 4.154161 0.794787 -1.673558
H 2.115367 1.235998 -2.219493
C 5.042518 -0.153005 -1.175989
H 5.256914 -2.178843 -0.475766
H 4.507923 1.792148 -1.930402
H 6.092761 0.095432 -1.038774
H -1.204558 -2.392805 -1.157993
P -3.152933 -1.028319 -0.804314
C -3.572793 -1.302551 -2.536054
H -4.609063 -1.000945 -2.723304
H -2.888965 -0.700149 -3.146446
H -3.447555 -2.361249 -2.786327
C -3.430619 0.709774 -0.439019
H -4.497934 0.939283 -0.528409
H -3.070441 0.906867 0.575047
H -2.855641 1.313146 -1.152603
C -4.236560 -2.059863 0.201461
H -4.015929 -3.112245 -0.006578
H -4.018205 -1.852708 1.253126
H -5.286086 -1.843333 -0.027046

III
E = -2927.16806522
C -2.626717 -0.358176 -0.047630
C -1.898767 -1.040426 -1.023117
C -2.565382 -1.786463 -1.996513
C -3.950911 -1.866589 -1.995342
C -4.678502 -1.205882 -1.013169
C -4.016393 -0.463976 -0.044323
H -1.974211 -2.308355 -2.744982
H -4.457078 -2.448924 -2.762296
H -5.764876 -1.268683 -0.997018
H -4.558268 0.065687 0.735534
S -1.908136 0.591105 1.335292
S -0.118181 -0.971853 -1.333761
O -3.059235 1.390152 1.794422
O -1.399349 -0.489686 2.218252
O 0.153483 0.283255 -2.034600
O 0.173461 -2.230118 -2.049490
C -0.622379 1.490687 0.799272
P 0.626408 -2.558173 1.066784
P -1.011180 2.898611 -0.094485
C 1.041340 -2.336303 2.800277

H 0.989061 -3.308454 3.304112
H 2.044217 -1.907544 2.885139
H 0.321042 -1.626610 3.222457
C -1.004402 -3.312359 0.972581
H -1.237304 -3.537077 -0.072599
H -0.976461 -4.239461 1.556118
H -1.734177 -2.617262 1.398067
C 1.781141 -3.749551 0.353326
H 2.802117 -3.365467 0.457592
H 1.702163 -4.724371 0.846956
H 1.537769 -3.833686 -0.711919
C 0.393030 3.547525 -1.010408
H 0.067511 4.435131 -1.564199
H 1.206751 3.793745 -0.324235
H 0.745303 2.759721 -1.682148
C -2.309176 2.622597 -1.330925
H -3.218385 2.287748 -0.820471
H -2.515305 3.547405 -1.881522
H -1.957809 1.842653 -2.015158
C -1.643267 4.236633 0.953677
H -1.975987 5.100393 0.366510
H -2.475310 3.815025 1.528726
H -0.853198 4.538154 1.649647
C 0.800219 -1.013640 0.185023
H 1.820292 -1.048457 -0.237714
Si 1.051153 0.717433 1.338011
O 2.019116 1.487423 0.067300
C 3.259110 1.052367 -0.198432
C 3.529609 0.476271 -1.447531
C 4.288946 1.139549 0.747874
C 4.807916 0.015879 -1.743698
H 2.713948 0.403263 -2.164752
C 5.561568 0.673785 0.440266
H 4.068831 1.582173 1.717041
C 5.830699 0.110041 -0.804134
H 5.005018 -0.421843 -2.721380
H 6.354729 0.755786 1.182316
H 6.830235 -0.249382 -1.040469

IV
E = -2135.13462731
C 3.141566 1.900066 1.229458
C 1.930888 1.277662 0.943890
C 0.953800 1.728344 0.040771
C 1.299881 2.886435 -0.678245
C 2.505641 3.527726 -0.435439
C 3.421790 3.059690 0.523828
H 3.816253 1.506427 1.988362
H 0.601369 3.297987 -1.405103
H 2.742679 4.438125 -0.984057
H 4.338420 3.612218 0.717005
B -0.315638 0.784293 0.022852
S 1.373522 -0.272545 1.479630
O 1.929762 -0.817207 2.708748
O -0.136091 -0.001531 1.420473
C 1.553871 -1.329459 0.025168
Si 0.088021 -0.835012 -1.344628
C -1.768107 1.375375 -0.107082
C -2.687348 1.333403 0.947613
C -2.193325 1.944978 -1.315551

C -3.976492 1.831270 0.800312
H -2.390959 0.877913 1.891052
C -3.478116 2.454449 -1.465383
H -1.512718 1.958190 -2.168981
C -4.378649 2.396167 -0.406074
H -4.677158 1.768553 1.631715
H -3.783940 2.886241 -2.417710
H -5.390228 2.781572 -0.523052
P 3.149442 -1.391363 -0.725736
C 3.426820 0.013665 -1.812966
H 4.353890 -0.140056 -2.376340
H 2.566813 0.063242 -2.494566
H 3.489156 0.944334 -1.239602
C 3.195597 -2.886350 -1.726084
H 2.376051 -2.819015 -2.451134
H 4.160062 -2.963508 -2.239900
H 3.043733 -3.762119 -1.086569
C 4.498229 -1.472124 0.475283
H 4.487271 -0.563193 1.084992
H 4.337721 -2.331474 1.134927
H 5.466806 -1.560111 -0.029367
C -3.202289 -1.422318 -1.159843
C -2.247716 -1.898984 -0.253864
C -2.651037 -2.244167 1.038769
C -3.981795 -2.111065 1.415187
C -4.927756 -1.629311 0.516008
C -4.526993 -1.283281 -0.771766
H -2.884077 -1.140722 -2.162463
H -1.896891 -2.596586 1.738865
H -4.280628 -2.378930 2.427817
H -5.967040 -1.516330 0.817684
H -5.252744 -0.890599 -1.481964
O -0.957738 -2.038203 -0.597803
H 1.245840 -2.329772 0.355592

V
E = -1585.94278554
C -1.923694 -3.234103 -0.228224
C -2.287465 -0.841997 0.043109
C -3.644600 -1.112396 0.276607
C -4.137241 -2.409646 0.265223
C -3.271629 -3.472130 0.015808
H -1.274251 -4.081786 -0.440616
H -4.315504 -0.283271 0.498323
H -5.191024 -2.601025 0.460343
H -3.649661 -4.493156 0.003115
B -1.592866 0.558617 0.129948
C -2.337196 1.916210 0.041314
C -3.483658 2.086423 -0.756559
C -1.852950 3.053450 0.713095
C -4.111433 3.320357 -0.877208
H -3.876890 1.234675 -1.310899
C -2.489716 4.283056 0.618309
H -0.957928 2.959624 1.327869
C -3.621658 4.421433 -0.181330
H -4.989597 3.424818 -1.512833
H -2.097667 5.142505 1.159904
H -4.117285 5.387241 -0.266058
C -1.422514 -1.930072 -0.233369
C 0.008819 -1.552642 -0.484226

P 1.279595 -2.702421 -0.026026
C 1.006443 -3.285404 1.655091
H 0.084927 -3.874503 1.698596
H 1.855866 -3.886881 1.994323
H 0.894812 -2.391131 2.281943
C 1.477489 -4.156444 -1.099521
H 2.345156 -4.744977 -0.780476
H 0.587137 -4.790739 -1.077778
H 1.637778 -3.812817 -2.127626
C 2.878747 -1.874988 -0.089750
H 3.682245 -2.606082 0.051301
H 2.997081 -1.372363 -1.056215
H 2.918765 -1.111993 0.696376
Si 0.277603 0.149460 0.594772
O 1.386445 0.937480 -0.520634
C 2.601590 1.431182 -0.270989
C 3.475515 1.609141 -1.353069
C 3.048745 1.764851 1.015148
C 4.758047 2.099990 -1.151895
H 3.112519 1.361247 -2.348719
C 4.338117 2.250661 1.204055
H 2.369873 1.635934 1.856663
C 5.203140 2.421500 0.128259
H 5.418765 2.234936 -2.007263
H 4.666256 2.506584 2.210750
H 6.208161 2.808061 0.283125
H 0.205480 -1.224533 -1.514514

Pathway C

IV
E = -2135.14675609
C 1.080658 3.399034 0.631161
C 0.771081 2.070609 0.336727
C -0.520492 1.643745 -0.032172
C -1.479718 2.668298 -0.099789
C -1.199013 3.990502 0.216264
C 0.087677 4.364089 0.585546
H 2.106577 3.658254 0.881179
H -2.484490 2.410831 -0.425781
H -1.990155 4.736166 0.158724
H 0.323008 5.399658 0.822043
B -1.017165 0.186693 -0.525013
S 2.192514 1.007920 0.574305
O 3.414866 1.794987 0.301559
O 2.133902 0.420073 1.918327
C 2.134950 -0.266424 -0.621675
Si 0.584921 -0.716247 -1.343727
C -0.999587 -1.057976 0.527532
C -0.544797 -0.964150 1.852569
C -1.492950 -2.305998 0.096380
C -0.583497 -2.061031 2.702325
H -0.159817 -0.018801 2.223801
C -1.534221 -3.405574 0.946033
H -1.878729 -2.397723 -0.920440
C -1.073869 -3.285452 2.252684
H -0.232989 -1.958368 3.727938
H -1.933702 -4.353710 0.590135
H -1.103600 -4.142318 2.923810

P 3.695790 -1.001195 -0.795833
C 4.815950 -0.019573 -1.816584
H 5.816355 -0.465307 -1.849208
H 4.402290 0.053207 -2.827779
H 4.847568 0.978778 -1.367524
C 3.542174 -2.620710 -1.575238
H 3.113109 -2.516124 -2.576494
H 4.535178 -3.076864 -1.652164
H 2.886602 -3.256099 -0.971151
C 4.509058 -1.243387 0.797706
H 4.653100 -0.258216 1.253722
H 3.846711 -1.825359 1.445963
H 5.470911 -1.750101 0.661465
C -3.443804 0.033966 -0.958585
C -4.411593 -0.306042 -1.910558
C -5.736524 -0.476772 -1.536522
C -6.124651 -0.315973 -0.208588
C -5.165208 0.024308 0.738208
C -3.834760 0.201053 0.375472
H -4.092768 -0.427584 -2.943462
H -6.475396 -0.740458 -2.291861
H -7.163635 -0.452824 0.083378
H -5.451589 0.157087 1.780359
H -3.093703 0.471519 1.123926
O -2.183095 0.202885 -1.386721
H 0.732470 -1.983265 -2.123434

V
E = -1585.95026060
C -1.318817 2.486319 1.064706
C 0.707036 1.276094 0.471111
C 1.362260 1.946305 1.508063
C 0.699511 2.847493 2.334717
C -0.649644 3.106723 2.110886
H -2.361985 2.746339 0.886400
H 2.414889 1.728728 1.688908
H 1.226658 3.338551 3.150506
H -1.183354 3.813176 2.745339
B 1.433198 0.202162 -0.443424
C 2.985668 -0.000686 -0.495635
C 3.851915 1.098128 -0.594371
C 3.564529 -1.274923 -0.443683
C 5.230957 0.933242 -0.639617
H 3.431558 2.102915 -0.640603
C 4.944043 -1.447676 -0.482820
H 2.915753 -2.146840 -0.374222
C 5.783677 -0.343304 -0.581810
H 5.879935 1.804233 -0.720109
H 5.367143 -2.450347 -0.437071
H 6.863935 -0.475554 -0.615070
C -0.666292 1.571575 0.220442
C -1.286624 0.900406 -0.929087
P -2.963897 0.603147 -1.000013
C -3.642778 -0.036559 0.556285
H -3.416015 0.669019 1.361724
H -4.726356 -0.185346 0.489380
H -3.141495 -0.984131 0.782608
C -4.017456 2.030587 -1.423148
H -5.076469 1.748977 -1.453809
H -3.877098 2.827892 -0.686731

H -3.705753 2.411198 -2.401698
C -3.364389 -0.624924 -2.264401
H -4.450339 -0.751412 -2.328606
H -2.977092 -0.294474 -3.233588
H -2.895140 -1.576775 -1.997932
Si 0.014271 0.098172 -1.880219
H -0.437143 -0.824082 -2.963473
C -0.151375 -1.478060 1.721387
C -1.006411 -2.159791 2.582499
C -1.867834 -3.143940 2.108208
C -1.867917 -3.454091 0.750120
C -1.025486 -2.775728 -0.121092
C -0.165932 -1.788859 0.364678
H 0.522199 -0.712067 2.091803
H -0.992203 -1.915120 3.642964
H -2.527940 -3.672315 2.792627
H -2.524821 -4.232798 0.366235
H -0.998103 -3.011929 -1.183650
O 0.646257 -1.175032 -0.540774

5 Results of the Calculations of all Germylenes

5.1 Energies

5.1.1 Energies of the Germylenes

Table S5.1. Energies of the Silylenes

Germylenes	E(SCF)	Corr(H)	Corr(G)	ΔG [hartree]	ΔG [kJ/mol]
**$^{Tos}Y_2Ge$**	-5871.21689973	0.885738	0.731769	–	–
**^{Tos}YGeCl**	-4435.22592543	0.446613	0.351955	–	–
**^{Tos}YGeHMDS**	-4849.01430566	0.690789	0.567106	–	–
**$^{Tos}YGeC_6F_5$**	-4703.55333624	0.503780	0.391609	–	–
**^{Tos}YGePy**	-4222.69706449	0.527891	0.425257	–	–
**^{Tos}YGePyr**	-4238.75291907	0.515735	0.413854	–	–
**^{Tos}YGeB2**	-4381.70430283	0.545767	0.439486	–	–
**^{Tos}YGeB1**	-4650.89119210	0.692932	0.573252	–	–
**$^{Ph}Y_2Ge$**	-4694.01344921	0.799023	0.666141	–	–
**^{Ph}YGeCl**	-3846.63300659	0.403588	0.318293	–	–
**^{Ph}YGeHMDS**	-4260.41863914	0.647373	0.531173	–	–
**$^{Ph}YGeC_6F_5$**	-4114.96476816	0.460908	0.358484	–	–
**^{Ph}YGePy**	-3634.10198425	0.484882	0.391776	–	–
**^{Ph}YGePyr**	-3650.16359034	0.472821	0.379284	–	–
**^{Ph}YGeB2**	-3793.11527300	0.502586	0.404377	–	–
**^{Ph}YGeB1**	-4062.30320027	0.650364	0.539978	–	–
**$^{F}Y_2Ge$**	-5687.83724891	0.727014	0.579869	–	–
**^{F}YGeCl**	-4343.54097114	0.367370	0.272658	–	–
**^{F}YGeHMDS**	-4757.32516396	0.611587	0.490252	–	–
**$^{F}YGeC_6F_5$**	-4611.87076535	0.424784	0.313116	–	–
**^{F}YGePy**	-4131.01086860	0.448649	0.347003	–	–
**^{F}YGePyr**	-4147.07200084	0.436651	0.334702	–	–
**^{F}YGeB2**	-4290.02330518	0.466553	0.361207	–	–
**^{F}YGeB1**	-4559.21212423	0.613947	0.495631	–	–
**I**	-3096.13968216	0.319744	0.251882	–	–
**II**	-3645.32890364	0.333824	0.258871	–	–
**III**	-4407.39598976	0.373682	0.290554	–	–
**IV**	-3615.37576050	0.332043	0.257519	–	–
**V**	-3066.14845830	0.318298	0.248722	–	–

Table S5.2. Energies of the Germyleness in triplet state, energy of the singlet-triplet gap.

Germylenes	E(SCF)	Corr(H)	Corr(G)	ΔG [hartree]	ΔG [kJ/mol]
**$^{Tos}Y_2Ge$**	-5871.14951304	0.886186	0.732579	0.068197	179.050410
**^{Tos}YGeCl**	-4435.14078424	0.446310	0.348420	0.081606	214.257052
**^{Tos}YGeHMDS**	-4848.94431010	0.690641	0.565670	0.068560	180.003125
**$^{Tos}YGeC_6F_5$**	-4703.48190373	0.503699	0.388594	0.068418	179.630173
**^{Tos}YGePy**	-4222.63585067	0.527646	0.422550	0.058507	153.609656
**^{Tos}YGePyr**	-4238.69569306	0.515493	0.410442	0.053814	141.288683
**^{Tos}YGeB2**	-4381.65898163	0.546078	0.436760	0.042595	111.833698
**^{Tos}YGeB1**	-4650.84416090	0.693883	0.576523	0.050302	132.068426
**$^{Ph}Y_2Ge$**	-4693.95413228	0.798364	0.668315	0.061491	161.444437
**^{Ph}YGeCl**	-3846.56536613	0.402636	0.315744	0.065091	170.897628
**^{Ph}YGeHMDS**	-4260.34931483	0.646999	0.532791	0.070942	186.259035
**$^{Ph}YGeC_6F_5$**	-4114.89071003	0.460028	0.357246	0.072820	191.189251
**^{Ph}YGePy**	-3634.04599361	0.484101	0.388693	0.052908	138.909009
**^{Ph}YGePyr**	-3650.10506049	0.472181	0.377522	0.056768	149.043990
**^{Ph}YGeB2**	-3793.06992151	0.502357	0.402065	0.043039	113.000181
**^{Ph}YGeB1**	-4062.25856791	0.649744	0.540757	0.045411	119.227526
**$^{F}Y_2Ge$**	-5687.76630803	0.725550	0.576278	0.067350	176.827110
**^{F}YGeCl**	-4343.46281835	0.366517	0.267529	0.073024	191.723961
**^{F}YGeHMDS**	-4757.25462418	0.611207	0.488870	0.069158	181.573751
**$^{F}YGeC_6F_5$**	-4611.80588829	0.423805	0.311851	0.063612	167.013464
**^{F}YGePy**	-4130.94998112	0.448424	0.344898	0.058782	154.333401
**^{F}YGePyr**	-4147.00963070	0.436381	0.334044	0.061712	162.025224
**^{F}YGeB2**	-4289.97532452	0.466222	0.356028	0.042802	112.375758
**^{F}YGeB1**	-4559.15481770	0.613446	0.490723	0.052399	137.572341
**I**	-3096.05423687	0.318647	0.247868	0.081431	213.797852
**II**	-3645.24313744	0.332258	0.255194	0.082089	215.525195
**III**	-4407.32392771	0.373570	0.291489	0.072997	191.653755
**IV**	-3615.34098822	0.331872	0.254382	0.031635	83.058428
**V**	-3066.12322495	0.317645	0.246516	0.023027	60.458307

5.1.2 Energies of the H_2 activation

Pathway A

Table S5.3. Energies of the transition states of the H_2 activation following pathway **A**

Germylenes	E(SCF)	Corr(H)	Corr(G)	ΔG [hartree]	ΔG [kJ/mol]	1M ΔG [kJ/mol]
**$^{Tos}Y_2Ge$**	-5872.32885309	0.899807	0.748681	0.081035	212.758256	204.832
**$^{Tos}YGeCl$**	-4436.32799327	0.459574	0.361842	0.083896	220.268551	212.343
**$^{Tos}YGeHMDS$**	-4850.11913580	0.704391	0.582850	0.086991	228.393686	220.468
**$^{Tos}YGeC_6F_5$**	-4704.66376435	0.517617	0.407213	0.081253	213.328645	205.403
**$^{Tos}YGePy$**	-4223.82047984	0.541284	0.437968	0.065372	171.635075	163.709
**$^{Tos}YGePyr$**	-4239.88106424	0.529245	0.424494	0.058572	153.779522	145.854
**$^{Tos}YGeB2$**	n.o.	n.o.	n.o.	–	–	–
**$^{Tos}YGeB1$**	-4652.02225209	0.706647	0.588444	0.060209	158.077938	150.152
**$^{Ph}Y_2Ge$**	-4695.13169882	0.812204	0.681408	0.073094	191.908504	183.983
**$^{Ph}YGeCl$**	-3847.73579749	0.416670	0.330852	0.085845	225.385493	217.460
**$^{Ph}YGeHMDS$**	-4261.53209731	0.660592	0.546603	0.078049	204.916386	196.990
**$^{Ph}YGeC_6F_5$**	-4116.08051812	0.474524	0.371787	0.073630	193.314853	185.389
**$^{Ph}YGePy$**	-3635.22926625	0.497940	0.404944	0.061963	162.683039	154.757
**$^{Ph}YGePyr$**	-3651.28903708	0.485994	0.393685	0.065031	170.738756	162.813
**$^{Ph}YGeB2$**	-3794.25246723	0.515549	0.415870	0.050375	132.260767	124.335
**$^{Ph}YGeB1$**	-4063.43862562	0.663288	0.552615	0.053288	139.908533	131.983
**$^{F}Y_2Ge$**	-5688.93541287	0.740459	0.593570	0.091614	240.531845	232.606
**$^{F}YGeCl$**	-4344.63965990	0.380298	0.285274	0.090004	236.305315	228.379
**$^{F}YGeHMDS$**	-4758.43020898	0.624742	0.502040	0.082820	217.443040	209.517
**$^{F}YGeC_6F_5$**	-4612.97387142	0.437717	0.324237	0.084092	220.782545	212.857
**$^{F}YGePy$**	-4132.12258437	0.461413	0.358283	0.075641	198.595232	190.669
**$^{F}YGePyr$**	-4148.18183200	0.449731	0.347838	0.079382	208.416204	200.490
**$^{F}YGeB2$**	-4291.15537747	0.479535	0.371615	0.054412	142.859753	134.934
**$^{F}YGeB1$**	-4560.34530620	0.627147	0.506435	0.053699	140.985986	133.060
**I**	-3097.21826486	0.331392	0.261761	0.10712496	281.907782	273.982
**II**	-3646.42412950	0.346023	0.269640	0.091408	240.547860	232.622
**III**	-4408.51351673	0.386971	0.305697	0.073522	193.480233	185.554
**IV**	n.o.	n.o.	n.o.	–	–	–
**V**	n.o.	n.o.	n.o.	–	–	–

Table S5.4. Energies of the H_2 activated products following pathway **A**

Germylenes	E(SCF)	Corr(H)	Corr(G)	ΔG [hartree]	ΔG [kJ/mol]	1M ΔG [kJ/mol]
**$^{Tos}Y_2Ge$**	-5872.42401869	0.904050	0.752127	-0.010684	-28.051554	-35.977
**$^{Tos}YGeCl$**	-4436.42158259	0.464058	0.367453	-0.004082	-10.718528	-18.644
**$^{Tos}YGeHMDS$**	-4850.22337441	0.708964	0.585662	-0.014436	-37.901879	-45.828
**$^{Tos}YGeC_6F_5$**	-4704.76238422	0.522007	0.409895	-0.014685	-38.556232	-46.482
**$^{Tos}YGePy$**	-4223.90829333	0.545494	0.442275	-0.018134	-47.611214	-55.537
**$^{Tos}YGePyr$**	-4239.96535233	0.533316	0.430997	-0.019214	-50.445231	-58.371
**$^{Tos}YGeB2$**	-4382.92514797	0.562883	0.455363	-0.028891	-75.854505	-83.780
**$^{Tos}YGeB1$**	-4652.10228587	0.710389	0.588252	-0.020017	-52.554847	-60.481
**$^{Ph}Y_2Ge$**	-4695.22357507	0.816368	0.686331	-0.013859	-36.387254	-44.313
**$^{Ph}YGeCl$**	-3847.83356599	0.420931	0.337694	-0.005082	-13.342033	-21.268
**$^{Ph}YGeHMDS$**	-4261.62255210	0.665090	0.550013	-0.008996	-23.619710	-31.546
**$^{Ph}YGeC_6F_5$**	-4116.16452380	0.477810	0.374047	-0.008116	-21.308430	-29.234
**$^{Ph}YGePy$**	-3635.30673197	0.501657	0.407125	-0.013322	-34.976993	-42.903
**$^{Ph}YGePyr$**	-3651.37222768	0.489750	0.396843	-0.015002	-39.386835	-47.313
**$^{Ph}YGeB2$**	-3794.32403634	0.519452	0.420220	-0.016844	-44.223006	-52.149
**$^{Ph}YGeB1$**	-4063.51376319	0.666848	0.554732	-0.019732	-51.806973	-59.733
**$^{F}Y_2Ge$**	-5689.04492829	0.744601	0.597840	-0.013632	-35.790005	-43.716
**$^{F}YGeCl$**	-4344.73667122	0.384499	0.291942	-0.000339	-0.891072	-8.817
**$^{F}YGeHMDS$**	-4758.53129273	0.629484	0.506721	-0.013583	-35.662380	-43.588
**$^{F}YGeC_6F_5$**	-4613.07173097	0.441827	0.330443	-0.007562	-19.853851	-27.780
**$^{F}YGePy$**	-4132.21905465	0.465709	0.364282	-0.014830	-38.937114	-46.863
**$^{F}YGePyr$**	-4148.27595621	0.453489	0.351908	-0.010673	-28.021125	-35.947
**$^{F}YGeB2$**	-4291.23124642	0.482892	0.374544	-0.018528	-48.644086	-56.570
**$^{F}YGeB1$**	-4560.42388592	0.631535	0.512854	-0.018462	-48.471984	-56.398
**I**	-3097.31387278	0.335856	0.266002	0.015969	42.023933	34.098
**II**	-3646.52404562	0.350828	0.276117	-0.001815	-4.776549	-12.702
**III**	-4408.61051045	0.391654	0.311589	-0.017369	-45.707333	-53.633
**IV**	-3616.59318818	0.348696	0.272989	-0.025821	-67.950543	-75.876
**V**	-3067.36886792	0.334492	0.263982	-0.029005	-76.330981	-84.257

Pathway B

Table S5.5. Energies of the transition states of the H_2 activation following pathway **B**

Germylenes	E(SCF)	Corr(H)	Corr(G)	ΔG [hartree]	ΔG [kJ/mol]	1M ΔG [kJ/mol]
**$^{Tos}Y_2Ge$**	-5872.32529315	0.898422	0.745866	0.081780	214.714095	206.788
**^{Tos}YGeCl**	-3646.52430545	0.352619	0.278128	-0.000068	-0.178852	-8.105
**^{Tos}YGeHMDS**	-4850.13544038	0.704122	0.580903	0.068739	180.474162	172.548
**^{Tos}YGeB1**	-4652.03189161	0.707501	0.587652	0.049777	130.689983	122.764
**$^{Ph}Y_2Ge$**	-4695.15027900	0.812840	0.681252	0.054358	142.716663	134.791
**^{Ph}YGeCl**	-3847.76254297	0.417230	0.331555	0.059802	157.010962	149.085
**^{Ph}YGeHMDS**	-4261.54953800	0.661386	0.546593	0.060598	159.099599	151.174
**^{Ph}YGeB1**	-4063.44202020	0.664054	0.553734	0.051013	133.933998	126.008
**I**	-3097.25319055	0.332821	0.264082	0.074596	196.304168	188.378
**II**	-3646.45487748	0.347149	0.270062	0.061152	160.927000	153.001
**III**	-4408.53661898	0.387066	0.304765	0.049544	130.378310	122.452
**IV**	-3616.51971628	0.345820	0.270044	0.044543	117.217833	109.292
**V**	-3067.30074352	0.331958	0.261833	0.036817	96.887432	88.962

Table S5.6. Energies of the H_2 activated products following pathway **B**.

Germylenes	E(SCF)	Corr(H)	Corr(G)	ΔG [hartree]	ΔG [kJ/mol]	1M ΔG [kJ/mol]
**$^{Tos}Y_2Ge$**	-5872.35282600	0.905361	0.755025	0.063406	166.473552	158.548
**^{Tos}YGeCl**	-4436.40241434	0.465070	0.365219	0.012852	33.742345	25.816
**^{Tos}YGeHMDS**	-4850.19364278	0.709616	0.584261	0.013895	36.480190	28.554
**^{Tos}YGeB1**	-4652.07317039	0.712108	0.591958	0.012804	33.617949	25.692
**$^{Ph}Y_2Ge$**	-4695.21551874	0.818578	0.687382	-0.004752	-12.475959	-20.402
**^{Ph}YGeCl**	-3847.83096179	0.422876	0.336770	-0.003402	-8.930668	-16.857
**^{Ph}YGeHMDS**	-4261.61023276	0.667517	0.553575	0.006885	18.076748	10.151
**^{Ph}YGeB1**	-4063.50731655	0.669862	0.560017	-0.008001	-21.005552	-28.931
**I**	-3097.32626955	0.338532	0.271217	0.008804	23.168196	15.242
**II**	-3646.524305	0.352619	0.278128	-0.000068	-0.178852	-8.105
**III**	-4408.58161691	0.392732	0.311320	0.011190	29.446397	21.520
**IV**	-3616.56412120	0.350198	0.274821	0.005006	13.174729	5.249
**V**	-3067.36305632	0.336989	0.266060	-0.021134	-55.616836	-63.543

Pathway C

Table S5.7. Energies of the transition states of the H_2 activation following pathway **C**

Germylenes	E(SCF)	Corr(H)	Corr(G)	ΔG [hartree]	ΔG [kJ/mol]	1M ΔG [kJ/mol]
**IV**	-3616.52695199	0.345047	0.269262	0.036544	96.167336	88.241
**V**	-3067.31179563	0.330850	0.260750	0.024710	65.026700	57.101

Table S5.8. Energies of the H_2 activated products following pathway **C**

Germylenes	E(SCF)	Corr(H)	Corr(G)	ΔG [hartree]	ΔG [kJ/mol]	1M ΔG [kJ/mol]
**IV**	-3616.57722538	0.349240	0.274007	-0.008880	-23.36745	-31.293
**V**	-3067.36728974	0.335161	0.265602	-0.025815	-67.93416	-75.860

5.1.3 Energies of the phenol activation

Pathway A

Table S5.9. Energies of the coordination of phenol following pathway **A**

Germylenes	E(SCF)	Corr(H)	Corr(G)	ΔG [hartree]	ΔG [kJ/mol]	1M ΔG [kJ/mol]
**I**	-3404.13958251	0.434339	0.350844	0.003646	9.595415	1.670
**II**	n.o.	n.o.	n.o.	–	–	–
**III**	-4715.39303935	0.488131	0.388992	0.005968	15.704323	7.778
**IV**	-3923.37778191	0.446523	0.355616	0.000667	1.755514	-6.170
**V**	-1585.88683398	0.433301	0.349495	0.0044744	11.7748161	3.849

Table S5.10. Energies of the transition states of the phenol activation following pathway **A**

Germylenes	E(SCF)	Corr(H)	Corr(G)	ΔG [hartree]	ΔG [kJ/mol]	1M ΔG [kJ/mol]
**I**	-3404.07242708	0.427676	0.345141	0.064957	170.938770	163.013
**II**	-3953.26932526	0.442556	0.354075	0.059238	155.890191	147.964
**III**	-4715.35079920	0.481943	0.384973	0.044101	116.053953	108.128
**IV**	-3923.34222498	0.440278	0.352957	0.033489	88.129030	80.203
**V**	-3374.109963	0.2601	0.341776	0.0360593	94.8929476	86.967

Table S5.11. Energies of the phenol activated products following pathway **A**

Germylenes	E(SCF)	Corr(H)	Corr(G)	ΔG [hartree]	ΔG [kJ/mol]	1M ΔG [kJ/mol]
I	-3404.14795825	0.430545	0.349505	-0.006046	-15.910635	-23.837
II	-3953.34571228	0.445206	0.354642	-0.016407	-43.175271	-51.101
III	-2620.28203911	0.388190	0.307853	-0.033989	-89.445718	-97.372
IV	-3923.42013075	0.442875	0.354414	-0.042783	-112.58721	-120.513
V	-3.374.188.949	0.429071	0.345614	-0.038915	-102.4097	-110.336

Pathway B

Table S5.12. Energies of the coordination of phenol following pathway **B**

Germylenes	E(SCF)	Corr(H)	Corr(G)	ΔG [hartree]	ΔG [kJ/mol]	1M ΔG [kJ/mol]
I	-3404.14113528	0.434142	0.350194	0.001449	3.812042	-4.114
II	-3953.32866297	0.447138	0.358729	0.004681	12.318111	4.392
III	-4715.38070458	0.482124	0.384534	-0.003047	-8.019563	-15.945
IV	-3923.37873296	0.446326	0.357554	0.001652	4.346752	-3.579
V	-3374.15084779	0.432512	0.346331	-0.000187	-0.492124	-8.418

Table S5.13. Energies of the transition states of the phenol activation following pathway **B**

Germylenes	E(SCF)	Corr(H)	Corr(G)	ΔG [hartree]	ΔG [kJ/mol]	1M ΔG [kJ/mol]
I	-3404.11420025	0.428188	0.345980	0.024117	63.466106	55.540
II	-3953.31439801	0.442337	0.354860	0.015053	39.612704	31.687
III	-4715.38070458	0.482124	0.384534	0.013826	36.384783	28.459
IV	-3923.36338742	0.440558	0.352221	0.011641	30.634675	22.709
V	-3374.14018420	0.427114	0.344452	0.008577	22.571817	14.646

Table S5.14. Energies of the phenol activated products following pathway **B**

Germylenes	E(SCF)	Corr(H)	Corr(G)	ΔG [hartree]	ΔG [kJ/mol]	1M ΔG [kJ/mol]
I	-3404.15650094	0.432830	0.352192	-0.011888	-31.284749	-39.211
II	-3953.35564742	0.446696	0.359035	-0.021936	-57.726160	-65.652
III	-4715.41592234	0.487313	0.390243	-0.015614	-41.090467	-49.016
IV	-3923.38751983	0.444387	0.355248	-0.009415	-24.777579	-32.703
V	-3374.18787417	0.431766	0.347785	-0.035677	-93.887407	-101.813

Pathway C

Table S5.15. Energies of the coordination of phenol following pathway **C**

Germylenes	E(SCF)	Corr(H)	Corr(G)	ΔG [hartree]	ΔG [kJ/mol]	1M ΔG [kJ/mol]
**IV**	-3923.35260516	0.446960	0.355842	0.026072	68.453294	60.527
**V**	-3374.14691193	0.432663	0.347076	0.004483	11.797474	3.872

Table S5.16. Energies of the transition states of the phenol activation following pathway **C**

Germylenes	E(SCF)	Corr(H)	Corr(G)	ΔG [hartree]	ΔG [kJ/mol]	1M ΔG [kJ/mol]
**IV**	-3923.31478853	0.439896	0.350195	0.058242	152.914657	144.989
**V**	-3374.11136171	0.426434	0.345044	0.037924	99.799561	91.874

Table S5.17. Energies of the phenol activated products following pathway **C**

Germylenes	E(SCF)	Corr(H)	Corr(G)	ΔG [hartree]	ΔG [kJ/mol]	1M ΔG [kJ/mol]
**IV**	-3923.39251415	0.443198	0.353935	-0.015744	-41.334588	-49.260
**V**	-3374.20212951	0.429269	0.345654	-0.0520257	-136.909743	-144.836

5.2 Coordinates of the Structures

5.2.1 Singlet state structures

$^{Tos}Y_2Ge$

PW6B95
E = -5871.21689973
C 4.486748 2.520199 -0.331160
H 4.449586 2.795599 0.719869
C 3.388183 1.897202 -0.898929
C -3.387952 -1.897284 -0.898964
O 0.790022 2.147137 -0.566728
C -3.391814 -1.524509 -2.240622
H -2.511150 -1.045181 -2.670803
C 2.761725 -2.865790 2.909748
H 3.785502 -3.086298 2.609111
C 5.612853 2.756577 -1.114566
H 6.482941 3.237778 -0.669352
C -2.024450 5.085522 -1.253985
H -1.836898 6.112143 -0.946149
C -2.143064 4.089812 -0.291645
H -2.037350 4.335871 0.762198
C 2.457741 -2.453386 -2.041495
H 2.601942 -1.415808 -2.339223
C 2.371218 -2.772047 -0.683028
C 5.168078 -1.184475 -0.302610
H 4.875130 -1.718762 -1.203667
C 6.469526 -0.718322 -0.165487

H 7.190122 -0.888814 -0.962875
C -2.126449 4.769743 -2.604333
H -2.024302 5.551772 -3.354448
C -1.583192 0.115377 -0.093423
Ge 0.000037 0.000279 -1.362986
O -2.322140 -1.946522 1.464079
S -1.966693 -1.491905 0.110199
P -2.489553 1.409369 0.506113
C -4.519808 -1.764024 -3.007335
H -4.527399 -1.472077 -4.057030
C -4.618216 0.272641 1.868718
H -3.886529 0.076522 2.649598
C -0.647287 1.764317 2.496596
H -0.036922 1.122005 1.868958
C 5.922859 0.187632 1.999781
H 6.214371 0.730045 2.896992
C 4.618270 -0.272952 1.868712
H 3.886684 -0.076814 2.649685
C -2.457856 2.452966 -2.041690
H -2.602008 1.415356 -2.339319
C 1.950590 -2.056889 2.108007
C 3.392263 1.524219 -2.240538
H 2.511732 1.044677 -2.670746
C 2.142759 -4.090002 -0.291273
H 2.036956 -4.335936 0.762586
C 4.239635 -0.971166 0.718499
C -2.255196 3.379130 4.096492
H -2.883686 4.003027 4.729337
C -1.950804 2.057056 2.107816
C -2.762021 2.865929 2.909499
H -3.785896 3.086117 2.608947
C -2.371481 2.771803 -0.683247
C -2.342469 3.453097 -2.998116
H -2.406659 3.200525 -4.054491
C 6.880491 2.595718 -3.287634
H 6.634261 3.065675 -4.247587
H 7.377316 1.642680 -3.515323
H 7.606170 3.233634 -2.772584
C -0.942213 3.099498 4.474264
H -0.548182 3.513509 5.400875
C -5.653117 -2.376107 -2.454788
C 6.848843 -0.036597 0.986560
H 7.869322 0.327096 1.091953
C -6.469721 0.718067 -0.165243
H -7.190421 0.888595 -0.962529
C 1.583368 -0.115453 -0.093599
P 2.489417 -1.409523 0.506211
O 2.322128 1.946596 1.463983
S 1.966867 1.491774 0.110124
O -0.789886 -2.147308 -0.566468
C 5.653457 2.376140 -2.454562
C 4.520309 1.763770 -3.007142
H 4.528077 1.471629 -4.056782
C 2.342399 -3.453637 -2.997802
H 2.406709 -3.201206 -4.054205
C 2.126292 -4.770224 -2.603877
H 2.024187 -5.552339 -3.353908
C 2.024172 -5.085831 -1.253496
H 1.836546 -6.112404 -0.945546
C -5.168294 1.184225 -0.302515

H -4.875481 1.718577 -1.203576
C -0.137275 2.296904 3.674332
H 0.894801 2.085664 3.943812
C -4.239720 0.970871 0.718476
C -6.848887 0.036279 0.986825
H -7.869350 -0.327424 1.092332
C -5.612719 -2.756378 -1.114730
H -6.482922 -3.237415 -0.669559
C -5.922787 -0.187967 1.999926
H -6.214189 -0.730403 2.897157
C 0.942072 -3.098594 4.474795
H 0.548059 -3.512289 5.401554
C 2.254936 -3.378640 4.096904
H 2.883352 -4.002575 4.729785
C -4.486677 -2.520052 -0.331231
H -4.449627 -2.795370 0.719822
C -6.880203 -2.595403 -3.287862
H -7.604821 -3.235481 -2.773995
H -6.633778 -3.062711 -4.249042
H -7.378441 -1.642487 -3.513016
C 0.137224 -2.295974 3.674802
H -0.894747 -2.084430 3.944457
C 0.647189 -1.763758 2.496875
H 0.036876 -1.121436 1.869172

PBE0
E = -5862.53966783
C 4.430750 2.595263 -0.392551
H 4.372202 2.874042 0.660431
C 3.361318 1.926866 -0.973783
C -3.483384 -1.832761 -1.111528
O 0.755410 2.124970 -0.701212
C -3.504124 -1.388232 -2.434302
H -2.628893 -0.883378 -2.854163
C 2.881071 -2.628103 2.981669
H 3.943474 -2.712467 2.739693
C 5.558346 2.876365 -1.162817
H 6.406145 3.391634 -0.703195
C -2.158881 5.238565 -0.600378
H -2.044699 6.218866 -0.132284
C -2.246719 4.099609 0.197000
H -2.190748 4.189080 1.283649
C 2.431920 -2.542663 -2.036782
H 2.343553 -1.514261 -2.393937
C 2.508199 -2.789396 -0.661051
C 5.180553 -1.061030 -0.350173
H 4.893241 -1.627449 -1.238679
C 6.470320 -0.548493 -0.239377
H 7.189345 -0.713949 -1.044816
C -2.200474 5.126408 -1.989489
H -2.123160 6.021262 -2.611657
C -1.624668 0.098688 -0.200682
Ge -0.024638 0.032569 -1.448582
O -2.359966 -2.062307 1.221105
S -2.035846 -1.515317 -0.110923
P -2.499825 1.323301 0.584098
C -4.645015 -1.585060 -3.200273
H -4.663323 -1.238036 -4.237193
C -4.672028 0.088390 1.819955
H -3.957821 -0.200795 2.592862

C -0.637796 1.381108 2.607657
H -0.008538 0.863815 1.882362
C 5.917067 0.390365 1.916348
H 6.201611 0.964168 2.801286
C 4.622805 -0.112248 1.808909
H 3.893989 0.083026 2.597410
C -2.410796 2.729800 -1.794336
H -2.481834 1.740544 -2.252683
C 1.998157 -2.006517 2.088572
C 3.395217 1.559023 -2.319339
H 2.540402 1.041640 -2.765550
C 2.592474 -4.103478 -0.190411
H 2.627148 -4.301378 0.882916
C 4.253402 -0.849751 0.676683
C -2.265873 2.708179 4.448194
H -2.903047 3.222606 5.171458
C -1.943736 1.732026 2.262557
C -2.761668 2.400250 3.184149
H -3.786927 2.670734 2.918337
C -2.380940 2.842698 -0.398838
C -2.324881 3.871785 -2.585732
H -2.341453 3.779428 -3.673967
C 6.859336 2.766975 -3.323400
H 6.606005 3.096953 -4.341893
H 7.464887 1.850305 -3.421680
H 7.494694 3.534600 -2.859895
C -0.957012 2.358687 4.790419
H -0.571839 2.603722 5.783360
C -5.778441 -2.218254 -2.664088
C 6.840794 0.172869 0.895032
H 7.853680 0.573620 0.980380
C -6.483408 0.767663 -0.193565
H -7.188880 1.029668 -0.985271
C 1.569419 -0.124752 -0.178998
P 2.523476 -1.371220 0.470397
O 2.233212 1.985051 1.367018
S 1.932680 1.492445 0.010623
O -0.884686 -2.150937 -0.850875
C 5.627518 2.504670 -2.508305
C 4.521832 1.850700 -3.076143
H 4.550394 1.563834 -4.131025
C 2.450184 -3.606313 -2.935377
H 2.381542 -3.410548 -4.007738
C 2.541001 -4.915871 -2.465031
H 2.547366 -5.750141 -3.170584
C 2.610151 -5.163123 -1.094134
H 2.667984 -6.189174 -0.723978
C -5.168794 1.220687 -0.263596
H -4.850173 1.837258 -1.106693
C -0.143592 1.700565 3.870198
H 0.884480 1.434023 4.122910
C -4.258841 0.886986 0.745940
C -6.896141 -0.017161 0.882733
H -7.928286 -0.371714 0.935728
C -5.723271 -2.666673 -1.341656
H -6.593164 -3.166446 -0.906875
C -5.990355 -0.355577 1.886914
H -6.308198 -0.978196 2.726384
C 1.042331 -3.028793 4.498779
H 0.669816 -3.428363 5.445303

C 2.399990 -3.132222 4.188037
H 3.088327 -3.609618 4.889514
C -4.581514 -2.481868 -0.562904
H -4.533297 -2.821298 0.472626
C -7.022351 -2.378891 -3.487249
H -7.701388 -3.125161 -3.051700
H -6.786920 -2.685950 -4.517251
H -7.573762 -1.425899 -3.552246
C 0.163452 -2.419924 3.604131
H -0.903027 -2.344285 3.825313
C 0.641508 -1.903367 2.402825
H -0.044859 -1.426033 1.704291

BP86
E = -5866.58336376
C 4.047326 2.774796 0.017847
H 3.678623 3.069523 1.010475
C 3.233592 1.984330 -0.801696
C -4.070459 -1.405940 -1.311310
O 0.585495 2.011226 -1.180066
C -4.186778 -0.659104 -2.495975
H -3.291503 -0.196076 -2.940779
C 2.481363 -2.968609 2.505809
H 3.516398 -2.634004 2.674087
C 5.323264 3.141955 -0.438778
H 5.975205 3.748450 0.210523
C -0.871629 4.990729 0.488306
H -0.328191 5.701167 1.130433
C -1.210691 3.723529 0.989226
H -0.909994 3.433088 2.005370
C 2.881528 -2.139878 -2.568817
H 2.343398 -1.205357 -2.790181
C 3.067507 -2.518712 -1.225756
C 5.185223 -0.619626 0.168663
H 5.277619 -1.123978 -0.804747
C 6.277191 0.072506 0.713968
H 7.229659 0.108285 0.163467
C -1.201660 5.342328 -0.831120
H -0.925055 6.334024 -1.222376
C -1.751835 0.007523 -0.484059
Ge -0.212713 -0.066607 -1.832395
O -2.732630 -2.168596 0.831706
S -2.453622 -1.536123 -0.502435
P -2.265863 1.116538 0.723173
C -5.449365 -0.489974 -3.076051
H -5.546071 0.098691 -4.003116
C -4.553338 0.106290 2.009181
H -3.845014 -0.404879 2.675789
C -0.469371 -0.052689 2.465797
H -0.134820 -0.562195 1.552094
C 4.927881 0.694105 2.644887
H 4.822908 1.217322 3.607815
C 3.826498 0.014421 2.103440
H 2.858458 0.028857 2.624659
C -2.197659 3.149729 -1.170411
H -2.655989 2.398659 -1.832074
C 1.774762 -2.551191 1.357695
C 3.661733 1.583298 -2.078252
H 3.001616 0.967856 -2.707629
C 3.728274 -3.725635 -0.912514

H 3.853357 -4.030145 0.138554
C 3.960945 -0.656566 0.867411
C -1.364188 1.308904 4.760291
H -1.714511 1.838504 5.660149
C -1.513873 0.881624 2.370910
C -1.968624 1.564985 3.519031
H -2.801155 2.283106 3.447442
C -1.885468 2.806482 0.162568
C -1.862724 4.418970 -1.661314
H -2.099285 4.682058 -2.704009
C 7.186078 3.083997 -2.168592
H 7.204278 3.349042 -3.245533
H 7.873000 2.218813 -2.037735
H 7.608920 3.931804 -1.594076
C -0.322322 0.365632 4.856065
H 0.139001 0.157911 5.834631
C -6.607028 -1.050166 -2.484346
C 6.152251 0.721739 1.954461
H 7.010791 1.264799 2.379353
C -6.355923 1.343533 0.237167
H -7.057435 1.820215 -0.464509
C 1.420508 -0.222434 -0.499575
P 2.484630 -1.413748 0.109866
O 1.527073 1.946190 1.207391
S 1.624459 1.423862 -0.192102
O -1.555322 -2.321990 -1.456265
C 5.792128 2.736030 -1.704923
C 4.933902 1.961139 -2.521791
H 5.275909 1.644499 -3.520601
C 3.362318 -2.964445 -3.599776
H 3.205560 -2.673467 -4.649946
C 4.034522 -4.159069 -3.289130
H 4.411692 -4.804416 -4.097916
C 4.217038 -4.539251 -1.945782
H 4.735403 -5.480026 -1.703538
C -4.980275 1.586659 0.115275
H -4.612178 2.255232 -0.676232
C 0.121012 -0.317626 3.710742
H 0.921459 -1.070175 3.781179
C -4.073020 0.974267 1.005680
C -6.834799 0.490853 1.247524
H -7.915143 0.298084 1.338802
C -6.454478 -1.807373 -1.305333
H -7.343652 -2.250849 -0.828423
C -5.932830 -0.125096 2.130823
H -6.302407 -0.804378 2.914749
C 0.518245 -4.212268 3.242702
H 0.020509 -4.847902 3.991912
C 1.849458 -3.803760 3.443898
H 2.397616 -4.126189 4.343134
C -5.193077 -1.992671 -0.716384
H -5.066294 -2.560410 0.216277
C -7.972181 -0.802401 -3.080329
H -8.709838 -1.556653 -2.741194
H -7.943822 -0.816967 -4.189081
H -8.359030 0.196543 -2.779773
C -0.178256 -3.804845 2.090731
H -1.227467 -4.090190 1.924147
C 0.451657 -2.987400 1.141657
H -0.103656 -2.678922 0.241488

^{Tos}YGeCl
E = -4435.22592543
C -3.244339 0.178646 -1.455131
H -2.874687 -0.111165 -2.435652
C -2.427007 0.921184 -0.619906
O -0.690527 2.853404 -0.993212
C 2.494016 -2.536720 -1.176097
H 2.027631 -3.300609 -0.554164
C -4.511151 -0.187312 -1.009485
H -5.156595 -0.776830 -1.659116
C 1.011583 -0.129061 2.887741
H 0.175224 0.533836 2.673054
C 1.598421 -0.850775 1.843140
C -1.379042 -2.034179 1.038034
H -1.175946 -1.599545 2.015142
C -2.527873 -2.787528 0.839974
H -3.219963 -2.943948 1.664849
Ge 0.759715 2.852789 0.558060
C -1.915724 -3.123845 -1.466906
H -2.129489 -3.539760 -2.449400
C -0.767156 -2.363177 -1.278253
H -0.100191 -2.164885 -2.114150
C 2.133180 -1.191812 -1.031153
C -2.857115 1.313180 0.644918
H -2.196841 1.896832 1.286029
C 2.693021 -1.677115 2.103281
H 3.176919 -2.218160 1.294303
C -0.486388 -1.831294 -0.017582
C -6.322525 -0.228956 0.743617
H -6.919778 0.645049 1.032226
H -6.246560 -0.871467 1.630659
H -6.876669 -0.778694 -0.023778
C -2.792203 -3.340540 -0.410237
H -3.692720 -3.932434 -0.562520
C 0.331635 0.924750 0.039614
P 0.895347 -0.666856 0.182185
O -0.615818 0.764903 -2.482576
S -0.771995 1.329935 -1.142179
C -4.965425 0.179345 0.255771
C -4.118792 0.940596 1.073787
H -4.460579 1.243961 2.062608
C 1.507724 -0.247261 4.180236
H 1.050530 0.320992 4.987557
C 2.591875 -1.079855 4.436810
H 2.983530 -1.167424 5.448363
C 3.184386 -1.790581 3.398264
H 4.041897 -2.430943 3.594104
C 4.001143 -1.926272 -2.951151
H 4.732567 -2.213698 -3.704289
C 3.430908 -2.900173 -2.133255
H 3.711634 -3.945167 -2.249057
C 3.629933 -0.594088 -2.816613
H 4.066248 0.165479 -3.461666
C 2.690959 -0.220048 -1.860069
H 2.388198 0.820906 -1.755817
Cl 2.579587 3.197248 -0.752053

^{Tos}YGeHMDS
E = -4849.01430566

S -0.636572 -1.450046 -0.644015
P -0.762268 1.379030 -0.081978
Si 3.853576 0.328480 0.748062
Si 3.441339 -2.377907 -0.633878
O 0.313882 -2.509585 -0.073542
O -0.686999 -1.327720 -2.105938
N 2.870914 -1.067561 0.389725
C -0.151548 -0.156713 0.271020
C -2.255706 -2.012281 -0.147119
C -2.527159 -2.166398 1.209585
H -1.742649 -1.977631 1.943268
C -3.797870 -2.548127 1.604024
H -4.016422 -2.671009 2.664122
C -4.809190 -2.777902 0.661002
C -4.502449 -2.628496 -0.690339
H -5.275432 -2.806775 -1.436674
C -3.229591 -2.245985 -1.103178
H -2.982218 -2.113349 -2.153584
C -6.189078 -3.157668 1.106439
H -6.163734 -3.992801 1.816876
H -6.818370 -3.449761 0.259647
H -6.682267 -2.318542 1.615620
C -2.510804 1.311932 -0.579895
C -3.507784 1.229222 0.395149
H -3.249901 1.324003 1.448246
C -4.829087 1.024364 0.021520
H -5.602360 0.961840 0.784484
C -5.162046 0.901079 -1.324414
H -6.198919 0.743606 -1.615283
C -4.169471 0.964112 -2.295659
H -4.424808 0.850329 -3.347278
C -2.842889 1.159891 -1.928611
H -2.062419 1.172492 -2.686209
C 0.061257 2.219118 -1.463183
C -0.438193 3.406008 -2.009957
H -1.325218 3.874845 -1.584686
C 0.177105 3.969096 -3.119098
H -0.209846 4.892642 -3.545088
C 1.279059 3.340102 -3.695324
H 1.754916 3.776751 -4.571378
C 1.761956 2.149519 -3.165268
H 2.611547 1.649224 -3.625918
C 1.155358 1.582719 -2.048185
H 1.512309 0.639997 -1.630372
C -0.642286 2.393849 1.415252
C -0.814575 1.746391 2.642374
H -0.997481 0.673075 2.656756
C -0.720469 2.466393 3.826930
H -0.848791 1.956804 4.779611
C -0.447299 3.829921 3.793600
H -0.364877 4.391556 4.722104
C -0.266342 4.473854 2.573937
H -0.038315 5.537369 2.546439
C -0.362279 3.760223 1.385329
H -0.191382 4.266439 0.438752
C 4.660152 1.080902 -0.779172
H 5.266959 1.947711 -0.483378
H 3.900745 1.434762 -1.485155
H 5.315947 0.385090 -1.313296
C 2.741678 1.670511 1.461888

H 3.344518 2.513981 1.823086
H 2.124967 1.329297 2.305994
H 2.068550 2.054222 0.686297
C 5.192729 -0.045915 2.015079
H 5.913109 -0.781072 1.637791
H 4.745796 -0.456660 2.929828
H 5.747761 0.862476 2.285668
C 2.885247 -2.056127 -2.397288
H 3.319616 -1.111346 -2.754699
H 1.792969 -1.961937 -2.436227
H 3.190147 -2.852298 -3.088965
C 2.875675 -4.063776 -0.025171
H 3.411080 -4.842934 -0.584885
H 1.801608 -4.225470 -0.150316
H 3.128426 -4.190820 1.036400
C 5.326592 -2.504392 -0.593545
H 5.663479 -2.838594 0.396318
H 5.862678 -1.580998 -0.837071
H 5.638876 -3.266848 -1.320372
Ge 1.244192 -1.259799 1.387821

$^{Tos}YGeC_6F_5$
E = -4703.55333624
C 3.362493 -1.934842 -1.486112
H 2.911050 -1.848022 -2.471402
C 2.552119 -1.874234 -0.365112
O 0.138718 -2.711667 0.248884
C -0.489126 3.319976 -1.658603
H 0.285993 3.970814 -1.254856
C 4.734963 -2.094051 -1.317672
H 5.379410 -2.137039 -2.194567
C 1.058128 1.736476 2.667513
H 1.595455 0.792549 2.591147
C 0.418327 2.257211 1.536998
C 3.337158 1.576328 0.051418
H 3.246789 1.622158 1.134246
C 4.595277 1.571865 -0.534508
H 5.482878 1.615965 0.093299
C 3.584623 1.440348 -2.717778
H 3.678833 1.377863 -3.799978
C 2.320860 1.440870 -2.137307
H 1.434052 1.360216 -2.762279
C -0.588165 1.991896 -1.231621
C 3.085618 -1.979814 0.916982
H 2.426805 -1.930193 1.784244
C -0.338381 3.423997 1.648470
H -0.889817 3.805617 0.793986
C 2.192988 1.523732 -0.748515
C 6.781075 -2.337642 0.133773
H 7.020763 -3.204653 0.761554
H 7.206291 -1.455319 0.630470
H 7.293136 -2.461293 -0.825753
C 4.720156 1.509945 -1.919726
H 5.707891 1.506395 -2.376660
C 0.318082 -0.317869 0.424610
P 0.549851 1.303599 0.001417
O 0.547351 -1.513275 -1.984224
S 0.804947 -1.576768 -0.543818
C 5.300641 -2.190697 -0.047790
C 4.452169 -2.137230 1.067057

H 4.875985 -2.221782 2.066933
C 0.976618 2.402593 3.883369
H 1.475750 1.991483 4.758277
C 0.242356 3.579833 3.983951
H 0.171881 4.097559 4.938551
C -0.420398 4.082564 2.869866
H -1.018072 4.987918 2.952309
C -2.351855 2.968074 -3.144411
H -3.049968 3.353776 -3.884809
C -1.375029 3.806233 -2.610179
H -1.303940 4.840987 -2.939300
C -2.426502 1.638497 -2.747177
H -3.177019 0.978191 -3.176872
C -1.537089 1.144328 -1.798862
H -1.553041 0.096633 -1.507339
Ge -0.652136 -1.501682 1.786500
C -2.486761 -1.344533 0.870205
C -4.376897 -2.294256 -0.325256
C -4.244445 0.091096 -0.012275
C -4.906602 -1.022814 -0.515397
C -3.061646 -0.100969 0.686360
C -3.182681 -2.433815 0.374442
F -2.708357 -3.665327 0.542581
F -2.434346 0.993622 1.147330
F -5.020488 -3.348036 -0.804921
F -6.038791 -0.871257 -1.184468
F -4.735425 1.305363 -0.222400

^{Tos}YGePy
E = -4222.69706449
C 3.230097 -1.386089 -1.386150
H 2.786700 -1.387234 -2.378889
C 2.408036 -1.531536 -0.281642
O 0.254607 -2.932165 0.250903
C -1.598260 2.777809 -1.781426
H -0.938296 3.567650 -1.423258
C 4.599584 -1.230541 -1.192506
H 5.251570 -1.109269 -2.056531
C -0.125690 1.560097 2.739077
H 0.517054 0.681604 2.731968
C -0.702592 1.994784 1.540989
C 2.349340 1.963090 0.221478
H 2.194387 1.946868 1.298711
C 3.603602 2.260131 -0.293344
H 4.426497 2.481329 0.383387
C 2.759518 1.969644 -2.531588
H 2.920625 1.960098 -3.607718
C 1.501573 1.665128 -2.022221
H 0.690508 1.397768 -2.695935
C -1.502511 1.485830 -1.251158
C 2.930009 -1.534895 1.009450
H 2.261558 -1.650283 1.862434
C -1.571440 3.086965 1.550152
H -2.058525 3.410565 0.634626
C 1.288474 1.679335 -0.642010
C 6.622018 -1.021722 0.297089
H 7.039504 -1.799444 0.948188
H 6.824927 -0.056501 0.780452
H 7.169203 -1.041865 -0.650852
C 3.807565 2.271867 -1.670688

H 4.791914 2.505447 -2.071960
C -0.140591 -0.550822 0.473411
P -0.288295 1.072613 0.035014
O 0.404606 -1.624889 -1.942973
S 0.642138 -1.652210 -0.495832
C 5.150888 -1.218088 0.087321
C 4.294099 -1.379523 1.184930
H 4.709273 -1.382203 2.192075
C -0.397248 2.228802 3.925929
H 0.050408 1.881226 4.854607
C -1.251121 3.326655 3.927747
H -1.468783 3.845897 4.859103
C -1.840465 3.750296 2.741574
H -2.523903 4.596925 2.742961
C -3.334966 2.028061 -3.271174
H -4.054334 2.241042 -4.059831
C -2.518565 3.047757 -2.784503
H -2.594750 4.052535 -3.195538
C -3.219315 0.739224 -2.765604
H -3.839855 -0.064789 -3.155304
C -2.299621 0.462553 -1.758669
H -2.172962 -0.549655 -1.383372
Ge -0.975940 -1.962225 1.733528
C -2.612370 -2.180876 0.584109
N -3.559862 -1.322724 0.988343
C -3.965148 -2.897631 -1.265843
C -4.684202 -1.230048 0.286468
C -4.938872 -1.995383 -0.847378
H -4.125915 -3.514024 -2.150006
H -5.425605 -0.509982 0.641201
H -5.878240 -1.884732 -1.385581
C -2.783300 -2.992529 -0.541998
H -1.978504 -3.656185 -0.852482

^{Tos}YGePyr
E = -4238.75291907
C 3.506688 -0.704396 -1.522915
H 3.125818 -0.396151 -2.493446
C 2.639200 -1.286344 -0.613778
O 0.539805 -2.878553 -0.749845
C -1.800725 2.890511 -1.197973
H -1.324808 3.617369 -0.540048
C 4.837649 -0.516619 -1.161604
H 5.523922 -0.054532 -1.870058
C -0.472325 0.654776 2.944835
H 0.328392 -0.064584 2.782345
C -1.035905 1.312305 1.847018
C 2.130906 1.938534 0.902294
H 1.909002 1.574092 1.903456
C 3.380058 2.473999 0.623277
H 4.129934 2.533493 1.409532
C 2.720198 2.831860 -1.666149
H 2.952586 3.168764 -2.674413
C 1.467691 2.290308 -1.394483
H 0.736276 2.183657 -2.192154
C -1.524872 1.525897 -1.054842
C 3.079534 -1.691817 0.643591
H 2.377058 -2.144518 1.343320
C -2.106363 2.185409 2.041544
H -2.582980 2.669747 1.193970

C 1.162810 1.857400 -0.102762
C 6.731814 -0.669789 0.494177
H 7.184462 -1.582322 0.900773
H 6.801731 0.098459 1.276187
H 7.338635 -0.337816 -0.354393
C 3.673354 2.928587 -0.660119
H 4.654366 3.346846 -0.877364
C -0.003501 -0.728433 0.206931
P -0.366458 0.919479 0.208351
O 0.764167 -0.900043 -2.369293
S 0.905843 -1.417391 -1.004099
C 5.305745 -0.899801 0.093657
C 4.405697 -1.496495 0.987729
H 4.756812 -1.811647 1.969646
C -0.958345 0.891881 4.224161
H -0.518127 0.372072 5.072530
C -2.015629 1.774803 4.415834
H -2.401354 1.953893 5.417601
C -2.590698 2.415619 3.323973
H -3.429983 3.093060 3.468159
C -3.242899 2.397987 -3.062189
H -3.917620 2.738622 -3.845462
C -2.664397 3.322942 -2.195195
H -2.880330 4.384029 -2.303796
C -2.944821 1.046283 -2.940608
H -3.378029 0.323696 -3.629023
C -2.080905 0.607173 -1.942480
H -1.813444 -0.444171 -1.864670
Ge -0.744752 -2.485200 0.952014
C -2.529245 -2.158594 0.044612
N -3.058111 -2.776449 -1.012806
N -3.127577 -1.160772 0.718188
C -4.243844 -2.345198 -1.433720
C -4.313475 -0.747357 0.300005
C -4.933227 -1.313462 -0.808910
H -4.666358 -2.846757 -2.308503
H -4.782394 0.068319 0.857024
H -5.901105 -0.969631 -1.165407

^{Tos}YGeB2
E = -4381.70430283
C 3.270516 -1.936643 -1.324865
H 2.715659 -2.084713 -2.248036
C 2.574411 -1.768631 -0.139924
O 0.380656 -2.758150 0.898862
C -1.228492 2.667890 -1.998037
H -0.480435 3.450614 -1.874830
C 4.661751 -1.900300 -1.300766
H 5.216221 -2.026151 -2.229774
C 0.871271 2.109193 2.392923
H 1.439013 1.182335 2.457303
C 0.132266 2.381069 1.235678
C 2.933919 1.740982 -0.430118
H 2.939298 1.957871 0.636185
C 4.119450 1.781881 -1.150191
H 5.049422 2.035048 -0.645188
Ge -0.423396 -1.385902 2.321476
C 2.932152 1.155223 -3.151688
H 2.931257 0.915245 -4.213015
C 1.740338 1.110563 -2.435943

H 0.816586 0.820746 -2.931907
C -1.124580 1.472178 -1.278105
C 3.241467 -1.573606 1.067398
H 2.668492 -1.446034 1.986375
C -0.644726 3.538662 1.169689
H -1.256475 3.741792 0.295307
C 1.736320 1.419857 -1.074049
C 6.855774 -1.635786 -0.087985
H 7.272073 -2.357966 0.625330
H 7.205748 -0.642117 0.221951
H 7.281652 -1.849128 -1.073612
C 4.118380 1.495395 -2.512964
H 5.049707 1.527140 -3.075207
C 0.242846 -0.360576 0.635123
P 0.222316 1.158062 -0.101370
O 0.391269 -1.949158 -1.540230
S 0.792231 -1.720657 -0.149474
C 5.358230 -1.696341 -0.111062
C 4.625050 -1.538832 1.073546
H 5.153702 -1.387879 2.014027
C 0.853755 3.001894 3.456816
H 1.427744 2.781546 4.354364
C 0.091303 4.162779 3.379688
H 0.072316 4.858563 4.216301
C -0.660729 4.425745 2.239905
H -1.273504 5.323147 2.184805
C -3.220558 1.834956 -3.068401
H -4.047249 1.982457 -3.760824
C -2.278807 2.847191 -2.887754
H -2.363191 3.777429 -3.446312
C -3.097914 0.633330 -2.382020
H -3.821705 -0.166348 -2.527483
C -2.041437 0.447210 -1.495914
H -1.896523 -0.506109 -0.994094
C -4.277104 -0.813850 0.605325
C -3.936810 -2.006619 -0.028510
C -5.433232 -0.126105 0.292089
C -4.736879 -2.569045 -1.004462
C -6.250451 -0.686431 -0.693174
H -5.682711 0.807854 0.788416
C -5.910674 -1.882133 -1.327062
H -4.454555 -3.498050 -1.492385
H -7.171772 -0.178592 -0.970906
H -6.572547 -2.289415 -2.088603
B -2.325729 -1.495436 1.392297
O -3.297142 -0.494063 1.492195
O -2.743806 -2.439911 0.457190

^{Tos}YGeB1
E = -4650.89119210
C -4.024855 -0.400020 0.679637
H -3.776583 -0.790097 1.663604
C -3.065953 -0.433204 -0.319037
O -1.200506 -2.121592 -1.030228
C 1.497270 2.209860 2.770136
H 1.159318 3.226270 2.576565
C -5.274231 0.142874 0.396635
H -6.031642 0.178448 1.178666
C 0.434009 2.580955 -2.021785
H -0.224698 1.777472 -2.346311

C 0.832964 2.638112 -0.681258
C -2.258021 2.914730 0.296614
H -1.941297 3.157180 -0.716270
C -3.488920 3.353848 0.763753
H -4.132659 3.945907 0.116203
Ge 0.421363 -1.140012 -2.042278
C -3.083917 2.261030 2.873398
H -3.410111 1.995250 3.876928
C -1.850357 1.815010 2.410600
H -1.224278 1.188987 3.042546
C 1.133331 1.164960 1.912606
C -3.336890 0.050507 -1.596111
H -2.564306 0.006751 -2.364024
C 1.703670 3.646129 -0.266494
H 2.062565 3.680305 0.756963
C -1.427083 2.155441 1.124378
C -6.900883 1.280091 -1.154509
H -7.278161 0.988781 -2.141614
H -6.823379 2.376474 -1.149975
H -7.648510 0.999937 -0.405165
C -3.899644 3.033088 2.055134
H -4.865531 3.378143 2.419305
C -0.272962 0.043871 -0.466352
P 0.092004 1.419123 0.441422
O -1.450154 -1.472084 1.434613
S -1.428541 -1.044952 0.030135
C -5.570780 0.651632 -0.866886
C -4.585091 0.587603 -1.861157
H -4.805418 0.967484 -2.858069
C 0.888568 3.532686 -2.925659
H 0.578076 3.475703 -3.966959
C 1.735998 4.550737 -2.500922
H 2.086976 5.299641 -3.208484
C 2.144104 4.602859 -1.173407
H 2.823456 5.384969 -0.840524
C 2.634181 0.639690 4.199245
H 3.227129 0.435575 5.089012
C 2.258420 1.948348 3.900738
H 2.546674 2.764247 4.560755
C 2.228827 -0.404670 3.377954
H 2.496633 -1.431946 3.613902
C 1.475816 -0.146647 2.235846
H 1.134267 -0.963560 1.603645
N 3.073170 -1.402937 -0.267572
C 2.806128 -3.243040 0.971727
C 3.596053 -2.166482 0.772344
H 2.949857 -4.072514 1.655092
H 4.527979 -1.913235 1.263834
B 1.858748 -2.035390 -0.751482
C 0.748283 -4.206875 0.042846
C 0.315711 -4.725629 -1.176690
C 0.169103 -4.659466 1.227192
C -0.693895 -5.676306 -1.210528
H 0.775920 -4.366084 -2.095292
C -0.829509 -5.624249 1.188207
H 0.477338 -4.220595 2.173669
C -1.266814 -6.135490 -0.028651
H -1.032003 -6.066006 -2.168804
H -1.283150 -5.963678 2.117600
H -2.056055 -6.884048 -0.056722

C 3.586914 -0.154535 -0.636380
C 3.459487 0.290236 -1.956794
C 4.242200 0.665648 0.290168
C 3.980513 1.518776 -2.341100
H 2.985699 -0.356577 -2.693677
C 4.778945 1.882761 -0.108145
H 4.311725 0.354416 1.329956
C 4.656236 2.316184 -1.424144
H 3.869394 1.845433 -3.373334
H 5.289814 2.503574 0.626074
H 5.072968 3.273122 -1.730473
N 1.750393 -3.217479 0.065317

$^{Ph}Y_2Ge$
E = -4694.01344921
P -3.018557 -0.290493 -0.000586
P 3.006926 -0.417890 -0.041197
C -1.542092 0.449278 -0.453584
C -1.563161 1.898297 -0.689589
C -2.462765 2.780525 -0.060504
H -3.169292 2.397894 0.673387
C -2.462205 4.142956 -0.332415
H -3.174948 4.788886 0.178748
C -1.553747 4.683354 -1.234146
H -1.543788 5.751954 -1.439340
C -0.649834 3.832016 -1.865849
H 0.079110 4.235240 -2.567111
C -0.662082 2.471211 -1.606509
H 0.051934 1.816128 -2.103292
C -3.565622 0.083053 1.715207
C -4.832112 0.552946 2.064652
H -5.597584 0.686451 1.304430
C -5.115634 0.879090 3.387695
H -6.103679 1.253023 3.649499
C -4.142322 0.735454 4.369509
H -4.367256 0.993891 5.402541
C -2.873760 0.276075 4.025344
H -2.102571 0.175920 4.786701
C -2.583583 -0.035216 2.703776
H -1.581001 -0.360827 2.420072
C -2.922258 -2.105224 -0.076903
C -3.107200 -2.930507 1.029559
H -3.293050 -2.499449 2.010217
C -3.042160 -4.313520 0.881639
H -3.177700 -4.954536 1.750625
C -2.804169 -4.872097 -0.367310
H -2.751620 -5.953530 -0.479076
C -2.639151 -4.048308 -1.478763
H -2.456180 -4.483235 -2.459196
C -2.700773 -2.671300 -1.336358
H -2.561191 -2.020061 -2.197846
C -4.449322 0.069311 -1.075651
C -4.325181 0.944741 -2.154886
H -3.385773 1.463919 -2.323756
C -5.401499 1.143261 -3.013819
H -5.299950 1.832389 -3.849787
C -6.597037 0.463649 -2.810243
H -7.435766 0.621316 -3.485908
C -6.716793 -0.430960 -1.750434
H -7.643743 -0.980979 -1.599843

C -5.645533 -0.631750 -0.889436
H -5.731409 -1.350077 -0.074284
C 1.499744 0.375255 0.162147
C 1.540542 1.819877 0.433717
C 0.683795 2.379849 1.396566
H -0.000621 1.718559 1.926264
C 0.671855 3.742152 1.651758
H -0.020665 4.142257 2.390691
C 1.533089 4.598933 0.969535
H 1.523044 5.667966 1.173114
C 2.394159 4.069571 0.016360
H 3.064866 4.724368 -0.538713
C 2.387918 2.706099 -0.252618
H 3.027245 2.318453 -1.045400
C 4.317631 0.260656 1.039006
C 4.375080 -0.137504 2.376533
H 3.703644 -0.909157 2.744267
C 5.291550 0.441251 3.245333
H 5.328446 0.117148 4.283475
C 6.156621 1.430725 2.790314
H 6.871994 1.886436 3.472312
C 6.103261 1.836662 1.462298
H 6.772247 2.614689 1.100107
C 5.189727 1.255861 0.590423
H 5.158666 1.582349 -0.445806
C 2.882976 -2.196037 0.354387
C 3.490322 -3.179566 -0.424997
H 4.024404 -2.908809 -1.331594
C 3.395435 -4.519541 -0.060963
H 3.865160 -5.279402 -0.682437
C 2.697347 -4.886240 1.082354
H 2.622071 -5.935259 1.362803
C 2.084581 -3.909689 1.862493
H 1.521067 -4.191346 2.749721
C 2.171119 -2.574071 1.498429
H 1.662421 -1.808825 2.081371
C 3.760792 -0.362907 -1.714146
C 5.102272 -0.679948 -1.951644
H 5.751344 -0.954032 -1.120683
C 5.609441 -0.640357 -3.244328
H 6.653970 -0.887470 -3.424878
C 4.782942 -0.281458 -4.306804
H 5.183626 -0.246967 -5.318286
C 3.450015 0.037830 -4.075194
H 2.803779 0.323301 -4.902858
C 2.940365 -0.001821 -2.781121
H 1.899734 0.253456 -2.577769
Ge -0.028500 -0.772743 -0.233458

^{Ph}YGeCl
E = -3846.63300659
P -0.587884 0.232860 -0.036359
C 0.757387 -0.833345 -0.103267
C 2.078833 -0.161947 -0.060770
C 2.659569 0.243475 1.148322
H 2.147365 0.016179 2.081227
C 3.868823 0.928889 1.169759
H 4.297890 1.235630 2.122368
C 4.532883 1.216093 -0.018617
H 5.480080 1.752120 -0.002672

C 3.981237 0.800262 -1.226384
H 4.497330 1.009372 -2.162046
C 2.770395 0.119927 -1.245925
H 2.333402 -0.195938 -2.191292
C -0.914092 0.942470 1.615025
C -1.467854 2.214203 1.780279
H -1.679847 2.834099 0.911274
C -1.744915 2.689470 3.057090
H -2.171891 3.682431 3.183410
C -1.474204 1.899245 4.169973
H -1.688182 2.275782 5.168468
C -0.925973 0.630460 4.008187
H -0.711172 0.012275 4.877492
C -0.644222 0.153372 2.733757
H -0.204150 -0.833919 2.592299
C -2.114151 -0.619285 -0.514142
C -3.269087 -0.546874 0.265926
H -3.266653 0.021736 1.192934
C -4.421649 -1.212808 -0.136153
H -5.316470 -1.160978 0.480548
C -4.428712 -1.946388 -1.316867
H -5.331388 -2.469417 -1.626714
C -3.281303 -2.016508 -2.101529
H -3.282895 -2.593780 -3.023621
C -2.125688 -1.357683 -1.703444
H -1.221851 -1.418200 -2.306903
C -0.403120 1.693660 -1.105033
C 0.622354 2.600748 -0.809588
H 1.253155 2.449349 0.065016
C 0.840706 3.694901 -1.635321
H 1.644352 4.390462 -1.403054
C 0.039279 3.897313 -2.755133
H 0.212925 4.755932 -3.400977
C -0.985364 3.004332 -3.046783
H -1.618693 3.163515 -3.917222
C -1.207753 1.903175 -2.226045
H -2.013830 1.210908 -2.458911
Ge 0.456741 -2.712822 -0.022918
Cl 2.610753 -3.320184 0.084543

^{Ph}YGeHMDS
E = -4260.41863914
P -1.775855 -0.113140 -0.001379
C -0.113399 0.207922 -0.309305
C 0.274916 1.572399 -0.700136
C -0.326861 2.727664 -0.168305
H -1.123285 2.631268 0.566568
C 0.097496 3.999502 -0.530451
H -0.387548 4.869921 -0.091313
C 1.142954 4.164966 -1.433358
H 1.482406 5.161127 -1.710066
C 1.738255 3.035174 -1.988266
H 2.545147 3.144432 -2.711970
C 1.304809 1.764431 -1.636556
H 1.756246 0.889022 -2.091811
C -2.439031 0.645651 1.527795
C -3.626404 1.375744 1.586501
H -4.235363 1.507376 0.695541
C -4.025252 1.957581 2.786151
H -4.948168 2.532935 2.824167

C -3.247329 1.811282 3.928910
H -3.561460 2.270944 4.863995
C -2.059319 1.087221 3.873220
H -1.440838 0.979150 4.761881
C -1.651413 0.516033 2.675526
H -0.707606 -0.028545 2.617725
C -2.055812 -1.898195 0.182882
C -2.565231 -2.473078 1.344888
H -2.800946 -1.852931 2.206277
C -2.763115 -3.849750 1.406654
H -3.152214 -4.296973 2.319031
C -2.461496 -4.648675 0.311217
H -2.614552 -5.724859 0.364579
C -1.966340 -4.073754 -0.857033
H -1.732067 -4.697406 -1.717184
C -1.765310 -2.703614 -0.924013
H -1.371555 -2.248893 -1.831837
C -2.921080 0.354666 -1.337313
C -2.497467 1.137863 -2.411575
H -1.475587 1.504820 -2.447880
C -3.386381 1.441524 -3.438063
H -3.051555 2.053954 -4.272696
C -4.691058 0.963550 -3.401740
H -5.381620 1.203270 -4.208122
C -5.111986 0.167128 -2.340021
H -6.127807 -0.222378 -2.316537
C -4.229365 -0.141140 -1.313644
H -4.552107 -0.782011 -0.493380
Ge 1.058832 -1.200545 0.302058
N 2.784195 -0.368451 0.073604
Si 3.778675 -1.079621 -1.169691
Si 3.415948 0.538685 1.425988
C 2.649753 -2.029546 -2.348668
H 2.166231 -2.882992 -1.854003
H 1.855988 -1.388262 -2.756753
H 3.229582 -2.422330 -3.194576
C 4.712964 0.189178 -2.201488
H 5.564853 0.617343 -1.662286
H 5.103931 -0.295148 -3.106877
H 4.064798 1.016861 -2.513205
C 5.029178 -2.291301 -0.453539
H 5.645212 -2.756850 -1.234325
H 5.705586 -1.789822 0.251298
H 4.512323 -3.088650 0.096376
C 5.089183 1.302934 1.037203
H 5.454694 1.834102 1.926211
H 5.846034 0.557651 0.762576
H 5.008886 2.030031 0.219885
C 3.621740 -0.612414 2.906117
H 4.008885 -0.086697 3.789101
H 2.661284 -1.070095 3.181465
H 4.312813 -1.430648 2.663588
C 2.271141 1.936741 1.932165
H 1.242476 1.594165 2.105443
H 2.634957 2.397410 2.860720
H 2.230637 2.710395 1.155301

$^{Ph}YGeC_6F_5$
E = -4114.96476816
P -1.833246 0.014934 -0.002859

C -0.161237 -0.135795 -0.389356
C 0.670086 1.046183 -0.048668
C 1.283483 1.184272 1.204566
H 1.132616 0.407223 1.952393
C 2.105596 2.270113 1.485062
H 2.583844 2.345703 2.460268
C 2.327715 3.248493 0.520872
H 2.977977 4.093883 0.737231
C 1.717729 3.132216 -0.724325
H 1.889139 3.888827 -1.488029
C 0.897124 2.046295 -1.003701
H 0.431833 1.947514 -1.982559
C -2.175746 -0.191232 1.777482
C -3.281682 0.411652 2.383553
H -3.939205 1.055296 1.801731
C -3.539213 0.192603 3.731408
H -4.399658 0.664742 4.201423
C -2.695673 -0.624733 4.478534
H -2.896607 -0.790529 5.535265
C -1.593560 -1.224329 3.878235
H -0.929693 -1.859126 4.461384
C -1.331380 -1.008628 2.530021
H -0.462723 -1.465503 2.055689
C -2.828522 -1.225351 -0.866671
C -3.797408 -1.978581 -0.203123
H -3.952223 -1.849758 0.865633
C -4.558202 -2.904870 -0.908110
H -5.307042 -3.496293 -0.385505
C -4.358247 -3.078983 -2.272734
H -4.953480 -3.806836 -2.820745
C -3.393762 -2.328091 -2.938423
H -3.230696 -2.468018 -4.004893
C -2.627557 -1.404743 -2.240031
H -1.861113 -0.825111 -2.751131
C -2.521213 1.651069 -0.395981
C -2.059654 2.753958 0.332534
H -1.347557 2.612846 1.144312
C -2.505023 4.030088 0.016833
H -2.137319 4.883322 0.582861
C -3.413348 4.216072 -1.021487
H -3.760794 5.217757 -1.267140
C -3.878741 3.122746 -1.742840
H -4.593669 3.264672 -2.550743
C -3.434586 1.841198 -1.433711
H -3.805940 0.989676 -1.999524
C 2.429346 -1.237414 -0.588091
C 4.254421 -0.975263 0.996181
C 4.380496 0.089982 -1.161833
C 4.941354 -0.187207 0.080267
Ge 0.504646 -1.833875 -0.926544
C 3.138877 -0.448403 -1.473068
C 3.012305 -1.481577 0.639802
F 2.329753 -2.171082 1.569220
F 2.604214 -0.137948 -2.658409
F 5.034721 0.858243 -2.020543
F 6.127132 0.309862 0.395154
F 4.780810 -1.210266 2.190709

^{Ph}YGePy
E = -3634.10198425

P -1.186685 -0.020647 -0.029322
C 0.509679 -0.275283 0.085726
C 1.350633 0.944103 0.005879
C 1.462705 1.839444 1.079827
C 2.301298 2.946828 1.011098
C 3.054485 3.186370 -0.133561
C 2.952624 2.310938 -1.210932
C 2.107368 1.210879 -1.144818
C -1.954930 0.697628 1.466006
C -3.039880 1.574161 1.402905
H -3.438261 1.880106 0.437500
C -3.607523 2.061203 2.575492
H -4.448478 2.749969 2.523282
C -3.099851 1.672299 3.810971
H -3.543549 2.058723 4.726642
C -2.022051 0.793900 3.877106
H -1.622694 0.490310 4.842788
C -1.448714 0.309952 2.707819
H -0.593351 -0.365398 2.741735
C -2.069288 -1.571284 -0.337585
C -3.176270 -1.956000 0.418736
H -3.522586 -1.329571 1.237691
C -3.832380 -3.148713 0.131258
H -4.689999 -3.449169 0.729662
C -3.391566 -3.954378 -0.912108
H -3.905808 -4.888039 -1.132085
C -2.291410 -3.570408 -1.673439
H -1.941019 -4.201751 -2.487108
C -1.628787 -2.385055 -1.387733
H -0.761359 -2.085267 -1.972199
C -1.658285 1.132955 -1.358979
C -1.190811 2.452140 -1.300429
H -0.595761 2.785291 -0.452642
C -1.485635 3.340988 -2.325692
H -1.112339 4.361693 -2.274215
C -2.250973 2.928700 -3.412466
H -2.479854 3.627867 -4.214537
C -2.725897 1.623821 -3.469897
H -3.331333 1.298256 -4.313500
C -2.431537 0.726745 -2.448258
H -2.811561 -0.290681 -2.501105
C 3.125132 -1.405830 0.363606
C 5.114855 -0.243453 1.053482
C 4.919040 -1.312307 -1.071126
C 5.676662 -0.562316 -0.179158
Ge 1.207701 -2.025278 0.417729
N 3.683450 -1.728777 -0.810191
H 5.673874 0.342610 1.782125
H 6.682046 -0.241502 -0.444152
H 5.332098 -1.589219 -2.044319
H 0.893307 1.648616 1.988009
H 2.375132 3.620611 1.863569
H 3.719040 4.046859 -0.184766
H 3.540658 2.483528 -2.110971
H 2.041539 0.516391 -1.979861
C 3.825368 -0.673709 1.328063
H 3.345562 -0.416089 2.271558

^{Ph}YGePyr
E = -3650.16359034

P -1.170117 -0.017954 -0.011662
C 0.497093 -0.305435 -0.310186
C 1.361785 0.899739 -0.246019
C 1.990327 1.295358 0.943477
C 2.829294 2.403996 0.979053
C 3.053725 3.151463 -0.172721
C 2.434567 2.775497 -1.361156
C 1.601495 1.664847 -1.395890
C -1.575584 0.222675 1.754815
C -2.662272 1.000307 2.164476
H -3.275059 1.513376 1.425320
C -2.958026 1.122689 3.516914
H -3.803201 1.731071 3.833053
C -2.172248 0.472367 4.464220
H -2.402932 0.573921 5.523080
C -1.090267 -0.302332 4.059344
H -0.472819 -0.809060 4.798258
C -0.790234 -0.426542 2.707490
H 0.059925 -1.023971 2.377811
C -2.208705 -1.386257 -0.584490
C -3.240791 -1.902425 0.199473
H -3.418982 -1.507095 1.196851
C -4.035648 -2.933161 -0.290327
H -4.833948 -3.338275 0.328093
C -3.806807 -3.447800 -1.561191
H -4.428578 -4.256673 -1.940240
C -2.779562 -2.933830 -2.347215
H -2.593634 -3.340096 -3.339195
C -1.979621 -1.908064 -1.862337
H -1.164271 -1.511684 -2.464152
C -1.789187 1.503021 -0.796641
C -1.305169 2.731036 -0.330254
H -0.618677 2.760797 0.514742
C -1.692584 3.911540 -0.949174
H -1.306431 4.861410 -0.585349
C -2.565720 3.878271 -2.032738
H -2.868226 4.805141 -2.516459
C -3.052974 2.661399 -2.495641
H -3.740342 2.632498 -3.338761
C -2.666155 1.474461 -1.881593
H -3.053573 0.525821 -2.246770
C 3.058647 -1.506488 -0.253229
C 4.808317 -1.151047 1.163296
C 4.917431 -0.505820 -1.101726
C 5.533631 -0.548247 0.143972
Ge 1.138465 -2.099446 -0.416352
N 3.697245 -0.983249 -1.305708
N 3.584854 -1.629512 0.972017
H 5.227660 -1.247160 2.168311
H 6.529276 -0.142930 0.307280
H 5.427919 -0.067088 -1.963010
H 1.834523 0.700234 1.841532
H 3.314037 2.682571 1.913585
H 3.711502 4.018395 -0.145510
H 2.607282 3.348428 -2.270933
H 1.126154 1.363982 -2.327532

^{Ph}YGeB2
E = -3793.11527300
P 1.647415 -0.044963 -0.009421

C 0.022739 0.489261 -0.252381
C -0.984074 -0.606109 -0.206287
C -1.564381 -1.023781 1.000491
C -2.536754 -2.018704 1.026811
C -2.948165 -2.628402 -0.152930
C -2.373011 -2.236795 -1.358787
C -1.402813 -1.243786 -1.383896
C 2.047864 -0.407464 1.736501
C 2.989051 -1.376202 2.094698
H 3.480480 -1.969877 1.326134
C 3.295186 -1.584756 3.434584
H 4.025624 -2.342804 3.710514
C 2.667009 -0.828748 4.419763
H 2.905637 -0.997017 5.468272
C 1.731596 0.138344 4.065842
H 1.237777 0.729304 4.834542
C 1.420506 0.347853 2.727534
H 0.683015 1.096014 2.436790
C 2.866114 1.175432 -0.557934
C 3.973149 1.504029 0.224357
H 4.101281 1.053779 1.205946
C 4.909659 2.418149 -0.246779
H 5.767036 2.677777 0.370703
C 4.747733 3.000653 -1.498130
H 5.481004 3.717148 -1.863484
C 3.645272 2.673278 -2.282707
H 3.512419 3.134070 -3.259259
C 2.703396 1.767226 -1.815267
H 1.830416 1.519225 -2.415542
C 2.020310 -1.605322 -0.870000
C 1.369522 -2.770889 -0.447148
H 0.700652 -2.742083 0.411646
C 1.570709 -3.965164 -1.125483
H 1.055407 -4.864306 -0.794297
C 2.422311 -4.009481 -2.225514
H 2.578225 -4.947032 -2.755832
C 3.075917 -2.856482 -2.644558
H 3.747894 -2.888647 -3.499853
C 2.876590 -1.655772 -1.970908
H 3.394926 -0.758940 -2.302471
H -1.263519 -0.534132 1.924847
H -2.985625 -2.307675 1.975585
H -3.719610 -3.395636 -0.134624
H -2.689888 -2.703507 -2.290098
H -0.962193 -0.930854 -2.328560
Ge -0.347801 2.363350 -0.301422
C -4.286148 0.851167 -0.757610
C -4.269360 1.044739 0.620869
C -5.306425 0.165319 -1.385536
C -5.273487 0.563149 1.437000
C -6.323452 -0.333698 -0.568297
H -5.300241 0.012873 -2.461426
C -6.307871 -0.138346 0.812514
H -5.244597 0.715522 2.512697
H -7.143806 -0.888459 -1.019113
H -7.116686 -0.542890 1.417651
B -2.400671 1.857579 -0.222056
O -3.129165 1.706451 0.958784
O -3.161585 1.396713 -1.293602

^{Ph}YGeB1
E = -4062.30320027
P 1.800368 -0.376400 0.006635
C 0.088923 -0.244272 -0.136585
C -0.637643 -0.215639 1.158732
C -0.829413 0.978116 1.870275
H -0.422793 1.901878 1.463593
C -1.538830 0.999165 3.065066
H -1.681949 1.943373 3.589438
C -2.066640 -0.178032 3.587654
H -2.626556 -0.162925 4.521032
C -1.871338 -1.373883 2.903972
H -2.279464 -2.302829 3.299781
C -1.161622 -1.392993 1.709154
H -1.023571 -2.328489 1.169144
C 2.630774 1.195069 0.432995
C 3.856796 1.217591 1.104097
H 4.328809 0.284569 1.407912
C 4.468808 2.431531 1.392157
H 5.421844 2.446046 1.917519
C 3.859396 3.625600 1.016361
H 4.336922 4.575396 1.250347
C 2.638924 3.606424 0.349689
H 2.152981 4.535671 0.060711
C 2.023779 2.394154 0.057392
H 1.061438 2.372305 -0.454565
C 2.562414 -0.974662 -1.522530
C 3.712740 -0.396472 -2.057292
H 4.161715 0.470086 -1.576820
C 4.278158 -0.921705 -3.215277
H 5.170511 -0.462123 -3.635335
C 3.702752 -2.024554 -3.835249
H 4.147121 -2.431496 -4.741661
C 2.555423 -2.605336 -3.301656
H 2.099066 -3.463845 -3.790032
C 1.981868 -2.081113 -2.152177
H 1.073649 -2.515848 -1.738863
C 2.321476 -1.505822 1.337670
C 2.047808 -1.142084 2.661684
H 1.586942 -0.179745 2.878789
C 2.355146 -2.010424 3.700144
H 2.131316 -1.722634 4.725262
C 2.941327 -3.243600 3.429985
H 3.181649 -3.922781 4.245823
C 3.221699 -3.606036 2.117730
H 3.685898 -4.566517 1.902706
C 2.912044 -2.742055 1.072191
H 3.136573 -3.032390 0.048372
Ge -0.654244 0.127805 -1.855549
N -3.560222 -0.348839 -0.511435
C -4.127103 1.668062 0.278340
C -4.476678 0.366284 0.256476
H -4.657605 2.499066 0.728490
H -5.359123 -0.095899 0.682422
B -2.538428 0.561415 -0.980382
N -2.972324 1.848557 -0.479694
C -2.245290 3.042659 -0.510127
C -2.232571 3.912058 0.585857
C -1.485604 3.370292 -1.638882
C -1.469104 5.072212 0.553579

H -2.795251 3.650385 1.479283
C -0.713280 4.524297 -1.657845
H -1.523123 2.720690 -2.511248
C -0.700609 5.384637 -0.563588
H -1.466302 5.732861 1.418830
H -0.128593 4.758805 -2.545583
H -0.104573 6.294842 -0.584521
C -3.550268 -1.745334 -0.603698
C -2.950112 -2.368084 -1.703207
C -4.099597 -2.545117 0.403204
C -2.874328 -3.752303 -1.775791
H -2.559922 -1.756186 -2.514535
C -4.036036 -3.929915 0.314362
H -4.537107 -2.076991 1.281252
C -3.416728 -4.544648 -0.768645
H -2.399687 -4.214605 -2.639618
H -4.463041 -4.534488 1.112795
H -3.362308 -5.629434 -0.830146

$^{F}Y_2Ge$
E = -5687.83724891
P 3.001263 0.843266 -0.110541
P -2.903494 1.162916 0.012181
C 1.583016 0.062899 -0.681368
C 1.485996 -1.382658 -0.756970
C 2.216450 -2.293596 0.022019
C 2.074225 -3.670250 -0.059637
C 1.133661 -4.211576 -0.924771
C 0.373771 -3.352241 -1.708644
C 0.572159 -1.983878 -1.640349
C 3.120551 1.048643 1.707502
C 3.991237 0.324525 2.520794
H 4.710103 -0.360118 2.078243
C 3.922337 0.453083 3.904760
H 4.601044 -0.122066 4.531367
C 2.985880 1.299522 4.485248
H 2.932504 1.393284 5.568179
C 2.108964 2.019320 3.677762
H 1.369344 2.679929 4.126618
C 2.171851 1.889452 2.298135
H 1.475404 2.445470 1.665729
C 3.088533 2.542574 -0.765126
C 3.652260 3.582927 -0.026858
H 3.954240 3.421843 1.005591
C 3.821535 4.836417 -0.606962
H 4.253243 5.646394 -0.022353
C 3.439164 5.052659 -1.924894
H 3.569327 6.034665 -2.375815
C 2.893009 4.010391 -2.669829
H 2.595282 4.175003 -3.703371
C 2.722200 2.759275 -2.096244
H 2.290502 1.939475 -2.667444
C 4.571037 0.124304 -0.682131
C 4.564118 -0.793100 -1.732930
H 3.619026 -1.110164 -2.166834
C 5.764637 -1.287972 -2.230552
H 5.753228 -2.009546 -3.044876
C 6.973397 -0.859624 -1.694026
H 7.911415 -1.248929 -2.085123
C 6.984465 0.076727 -0.664305

H 7.929370 0.426952 -0.253689
C 5.788023 0.571386 -0.161808
H 5.800500 1.315009 0.633767
C -1.439970 0.261932 0.022688
C -1.522880 -1.157655 0.341133
C -0.681767 -1.733845 1.302093
C -0.670757 -3.095716 1.556714
C -1.550230 -3.939301 0.888231
C -2.422575 -3.406183 -0.050801
C -2.377319 -2.046835 -0.320939
C -4.163188 0.369930 1.064285
C -4.026646 0.440427 2.453165
H -3.230439 1.035465 2.893885
C -4.906271 -0.247026 3.278802
H -4.792494 -0.184130 4.359058
C -5.925961 -1.015273 2.725761
H -6.612485 -1.557143 3.373389
C -6.062985 -1.093665 1.344872
H -6.853388 -1.699803 0.906994
C -5.186891 -0.404590 0.513757
H -5.287313 -0.487585 -0.564977
C -2.585458 2.821742 0.711507
C -3.157211 3.986795 0.202730
H -3.796207 3.947272 -0.674892
C -2.885536 5.215984 0.796108
H -3.326097 6.120846 0.382360
C -2.047418 5.290476 1.901319
H -1.834770 6.253808 2.360877
C -1.470067 4.130938 2.411440
H -0.802686 4.184983 3.269584
C -1.729746 2.904310 1.817144
H -1.243968 1.999729 2.181674
C -3.728149 1.434690 -1.591159
C -5.010551 1.986504 -1.679548
H -5.556656 2.248091 -0.773541
C -5.596120 2.188101 -2.922771
H -6.594116 2.617006 -2.989138
C -4.908013 1.834384 -4.080943
H -5.369461 1.989898 -5.054384
C -3.640806 1.269515 -3.994445
H -3.110536 0.974576 -4.897532
C -3.051111 1.065205 -2.751633
H -2.075902 0.586927 -2.666125
Ge 0.072503 1.321029 -0.571416
F -3.177482 -1.580929 -1.282895
F -3.254258 -4.198292 -0.709338
F -1.530718 -5.241249 1.125263
F 0.201346 -3.607091 2.414500
F 0.180265 -0.974153 1.970474
F -0.195859 -1.219792 -2.417285
F -0.568723 -3.845847 -2.499634
F 0.934214 -5.519462 -0.977956
F 2.801347 -4.463990 0.710597
F 3.099451 -1.827317 0.909999

^{F}YGeCl
E = -4343.54097114
P 1.160230 -0.311473 0.088067
C -0.053593 0.849973 -0.281289
C -1.450208 0.410549 -0.159416

C -2.255714 0.784430 0.919316
C -3.584224 0.397375 1.020914
C -4.151558 -0.379704 0.018659
C -3.384377 -0.763280 -1.073995
C -2.060210 -0.357290 -1.155374
C 1.527462 -0.505552 1.861452
C 2.153295 -1.659790 2.344083
H 2.392441 -2.476101 1.663870
C 2.463423 -1.765437 3.693380
H 2.950517 -2.663163 4.068839
C 2.144639 -0.726243 4.564675
H 2.382888 -0.814193 5.622993
C 1.515074 0.417641 4.087410
H 1.256050 1.224886 4.769249
C 1.205486 0.531013 2.735877
H 0.695622 1.413264 2.353609
C 2.713124 0.152323 -0.718866
C 3.926100 0.187859 -0.030519
H 3.960465 -0.052226 1.029585
C 5.091183 0.546565 -0.699685
H 6.033742 0.583807 -0.157655
C 5.050845 0.863646 -2.052593
H 5.964592 1.146319 -2.571703
C 3.842901 0.826804 -2.743422
H 3.808566 1.080218 -3.800809
C 2.674364 0.476619 -2.080681
H 1.722171 0.459251 -2.608832
C 0.714261 -1.991044 -0.439411
C -0.304652 -2.645985 0.261066
H -0.739011 -2.186283 1.148240
C -0.766427 -3.879932 -0.177059
H -1.565387 -4.381251 0.365208
C -0.207422 -4.471887 -1.305852
H -0.570960 -5.438604 -1.648456
C 0.816816 -3.830961 -1.993326
H 1.259717 -4.296199 -2.871452
C 1.275840 -2.590257 -1.566214
H 2.072897 -2.091737 -2.112748
Ge 0.480288 2.644467 -0.666487
Cl -1.582049 3.505970 -0.808847
F -1.359299 -0.732798 -2.218851
F -3.922327 -1.507470 -2.028742
F -5.415859 -0.757837 0.107689
F -4.315963 0.764373 2.061588
F -1.751076 1.530961 1.893008

^{F}*YGeHMDS*
E = -4757.32516396
P -1.932029 -0.261788 -0.115510
C -0.279189 0.010663 -0.490930
C 0.358229 1.311196 -0.369836
C -0.032050 2.329847 0.506579
C 0.614041 3.552327 0.599202
C 1.701517 3.817568 -0.222258
C 2.108965 2.849084 -1.132294
C 1.435815 1.640680 -1.208264
C -2.261790 -0.642835 1.643368
C -2.854758 0.251275 2.533639
H -3.247122 1.200288 2.177273
C -2.911849 -0.054227 3.890302

H -3.366998 0.653146 4.580504
C -2.378239 -1.246093 4.364739
H -2.418488 -1.476209 5.427575
C -1.780345 -2.140585 3.480101
H -1.350507 -3.070981 3.845575
C -1.717851 -1.836334 2.128448
H -1.226131 -2.527370 1.439516
C -2.525070 -1.723360 -1.026133
C -3.477110 -2.587251 -0.482751
H -3.812908 -2.458265 0.543877
C -3.994045 -3.624721 -1.251447
H -4.729098 -4.300601 -0.819260
C -3.570467 -3.799228 -2.563655
H -3.972937 -4.614756 -3.161420
C -2.633757 -2.929018 -3.113764
H -2.302025 -3.060696 -4.141505
C -2.115986 -1.891145 -2.351942
H -1.382074 -1.207532 -2.774259
C -3.084255 1.048638 -0.616065
C -2.662205 2.039017 -1.503357
H -1.632853 2.049654 -1.853357
C -3.562849 3.001242 -1.946944
H -3.228246 3.776117 -2.633489
C -4.885229 2.970606 -1.519296
H -5.587347 3.725485 -1.867786
C -5.315149 1.968158 -0.654261
H -6.353419 1.933169 -0.330720
C -4.419486 1.006680 -0.206194
H -4.760604 0.216118 0.460904
Ge 0.754584 -1.642074 -0.625197
N 2.436293 -1.039560 0.052984
Si 3.818725 -1.452293 -0.955442
Si 2.614956 -0.850554 1.790747
C 3.222636 -2.037074 -2.644693
H 2.670382 -2.983312 -2.581526
H 2.585309 -1.298780 -3.146236
H 4.097856 -2.207585 -3.286750
C 4.946624 0.025831 -1.211808
H 5.450493 0.330782 -0.288268
H 5.723349 -0.222192 -1.948000
H 4.384432 0.885729 -1.592768
C 4.814694 -2.882906 -0.237094
H 5.659100 -3.132453 -0.893465
H 5.221524 -2.668114 0.758119
H 4.180878 -3.775538 -0.151607
C 4.306715 -0.162088 2.241196
H 4.378540 -0.095037 3.335042
H 5.147229 -0.770590 1.889858
H 4.428246 0.850915 1.837054
C 2.383478 -2.530914 2.616174
H 2.512115 -2.472967 3.705137
H 1.370889 -2.912312 2.420241
H 3.093208 -3.270360 2.224857
C 1.381232 0.314914 2.593705
H 0.340373 0.129667 2.310149
H 1.456684 0.182221 3.681868
H 1.620048 1.361322 2.372665
F 1.825690 0.783972 -2.148668
F -1.059284 2.113359 1.333392
F 0.204719 4.462011 1.470899

F 2.335605 4.976710 -0.146136
F 3.124898 3.099649 -1.946462

$^{F}YGeC_6F_5$
E = -4611.87076535
P -2.135118 -0.034456 0.058734
C -0.475656 -0.388440 -0.259337
C 0.506855 0.630819 0.142373
C 1.322570 0.475914 1.266111
C 2.365065 1.345121 1.553625
C 2.594470 2.436220 0.723923
C 1.788532 2.637414 -0.390597
C 0.761452 1.745014 -0.663138
C -2.692462 -0.505121 1.725599
C -3.852636 0.051330 2.274938
H -4.415191 0.800275 1.718915
C -4.281046 -0.347368 3.533925
H -5.183451 0.084998 3.961482
C -3.551372 -1.293590 4.250383
H -3.885552 -1.599516 5.239950
C -2.393254 -1.839476 3.709338
H -1.816549 -2.569516 4.273242
C -1.960683 -1.447364 2.446576
H -1.043989 -1.852686 2.022335
C -3.195996 -0.893483 -1.126902
C -4.315187 -1.625281 -0.730604
H -4.567630 -1.711791 0.323676
C -5.099008 -2.260840 -1.687678
H -5.964770 -2.841203 -1.375869
C -4.772386 -2.162799 -3.035288
H -5.386619 -2.664485 -3.780500
C -3.656698 -1.431336 -3.433532
H -3.396192 -1.360165 -4.487345
C -2.864188 -0.801276 -2.484057
H -1.978368 -0.242470 -2.782900
C -2.485631 1.743886 -0.030226
C -1.985187 2.555681 0.993404
H -1.480969 2.105965 1.847935
C -2.129963 3.934403 0.920511
H -1.733751 4.562322 1.715730
C -2.780455 4.508883 -0.167547
H -2.892946 5.589709 -0.224426
C -3.288891 3.703328 -1.180244
H -3.802242 4.151103 -2.028524
C -3.139836 2.322531 -1.117139
H -3.534874 1.698385 -1.915258
C 1.980729 -1.717650 -0.809045
C 4.035763 -1.908758 0.473666
C 3.924985 -0.375482 -1.384410
C 4.638264 -0.955311 -0.339607
Ge -0.019708 -2.089978 -0.992463
C 2.616665 -0.781850 -1.605448
C 2.721154 -2.279032 0.214297
F 2.149579 -3.165356 1.034116
F 1.937818 -0.187186 -2.593222
F 4.497399 0.549508 -2.140207
F 5.889566 -0.592974 -0.115982
F 4.718652 -2.447506 1.471550
F 0.029663 1.946656 -1.750332
F 2.006240 3.676646 -1.179619

F 3.574514 3.279970 0.994266
F 3.136107 1.145693 2.610439
F 1.140985 -0.562265 2.076338

^{F}YGePy
E = -4131.01086860
P -1.631522 0.052139 0.057453
C -0.050274 -0.607538 -0.092446
C 1.086559 0.313312 0.041402
C 1.931982 0.293316 1.152522
C 3.113313 1.019182 1.199680
C 3.457755 1.835278 0.129512
C 2.628581 1.899915 -0.983621
C 1.466869 1.144251 -1.015640
C -2.319554 0.000222 1.744936
C -3.427436 0.781984 2.089436
H -3.877538 1.444508 1.350776
C -3.945505 0.719838 3.375921
H -4.808107 1.326975 3.643388
C -3.354697 -0.112375 4.324519
H -3.758309 -0.154882 5.334428
C -2.245142 -0.878538 3.987244
H -1.775759 -1.518193 4.731806
C -1.724887 -0.823728 2.697839
H -0.842527 -1.399922 2.424359
C -2.797628 -0.810094 -1.025342
C -4.045364 -1.254623 -0.590055
H -4.345967 -1.115868 0.445703
C -4.899890 -1.895685 -1.481013
H -5.868215 -2.252776 -1.136614
C -4.514718 -2.087444 -2.802641
H -5.185194 -2.591721 -3.495930
C -3.269135 -1.644010 -3.239575
H -2.963151 -1.800979 -4.271724
C -2.406858 -1.012288 -2.354324
H -1.421593 -0.678931 -2.678223
C -1.684550 1.822052 -0.349613
C -1.068071 2.708230 0.539905
H -0.645336 2.335908 1.472344
C -0.992869 4.060406 0.234613
H -0.506793 4.744682 0.926905
C -1.537908 4.536297 -0.954403
H -1.478338 5.596266 -1.193446
C -2.159371 3.658989 -1.835788
H -2.588925 4.030320 -2.763874
C -2.230655 2.302490 -1.539038
H -2.711512 1.620126 -2.236064
C 2.185157 -2.368462 -0.346501
C 4.331238 -2.591584 0.717926
C 4.051051 -1.546936 -1.408816
C 4.892251 -1.929508 -0.367804
F 0.720657 1.193344 -2.113241
F 2.964382 2.670243 -2.008456
F 4.574933 2.544608 0.168149
F 3.906187 0.950574 2.259070
F 1.635123 -0.479861 2.193982
Ge 0.168929 -2.475662 -0.435262
N 2.739118 -1.753545 -1.401008
H 4.953272 -2.914071 1.551982
H 5.957725 -1.714539 -0.412708

H 4.459388 -1.039250 -2.286220
C 2.962561 -2.828684 0.719854
H 2.491796 -3.339545 1.559088

^{F}YGePyr
E = -4147.07200084
P -1.617050 0.080707 0.064995
C -0.060677 -0.605892 -0.185981
C 1.096487 0.286981 -0.024895
C 1.919950 0.252858 1.103034
C 3.102379 0.976332 1.178521
C 3.472899 1.796166 0.120102
C 2.664189 1.875968 -1.007332
C 1.496128 1.131076 -1.064294
C -2.244618 -0.050295 1.770375
C -3.294931 0.759969 2.214717
H -3.735795 1.497398 1.544705
C -3.767332 0.629520 3.513761
H -4.584413 1.259614 3.859626
C -3.189195 -0.301473 4.374350
H -3.557026 -0.397679 5.394202
C -2.138041 -1.098681 3.937079
H -1.678428 -1.817338 4.612348
C -1.662425 -0.974709 2.635460
H -0.825790 -1.577949 2.287017
C -2.845262 -0.689508 -1.018715
C -4.094534 -1.108435 -0.562450
H -4.357526 -1.003847 0.487551
C -4.998768 -1.680088 -1.451456
H -5.968344 -2.017580 -1.091201
C -4.661599 -1.828425 -2.791836
H -5.370886 -2.279116 -3.483400
C -3.415099 -1.410121 -3.249840
H -3.147010 -1.533175 -4.297027
C -2.503785 -0.847363 -2.367075
H -1.517677 -0.534488 -2.708179
C -1.617422 1.869497 -0.250713
C -0.968962 2.690408 0.678171
H -0.563940 2.260031 1.593396
C -0.839694 4.050818 0.433612
H -0.329234 4.683958 1.156331
C -1.361722 4.600049 -0.733946
H -1.259907 5.666411 -0.925609
C -2.013940 3.787884 -1.654759
H -2.425230 4.216679 -2.566189
C -2.139413 2.423297 -1.418863
H -2.643942 1.791741 -2.146401
C 2.115195 -2.390920 -0.507877
C 3.948963 -2.759032 0.792415
C 4.124967 -1.654187 -1.284924
C 4.756293 -2.107650 -0.131618
F 0.756920 1.210600 -2.164338
F 3.020846 2.656764 -2.016739
F 4.593867 2.497731 0.184500
F 3.874234 0.897757 2.252841
F 1.594726 -0.513991 2.136798
Ge 0.101742 -2.459411 -0.627293
N 2.818691 -1.787509 -1.474360
N 2.643464 -2.913690 0.603697
H 4.369055 -3.168923 1.714075

H 5.822683 -1.972001 0.030929
H 4.692841 -1.158820 -2.076777

^{F}YGeB2
E = -4290.02330518
P -1.990285 0.107439 0.018105
C -0.489174 -0.753228 0.022711
C 0.700570 0.117713 -0.058345
C 1.367664 0.593267 1.070796
C 2.517951 1.366510 0.982760
C 3.008404 1.723713 -0.264911
C 2.344848 1.305493 -1.412825
C 1.206631 0.524064 -1.297163
C -2.722902 0.411425 1.655282
C -3.723034 1.376696 1.816742
H -4.062979 1.958014 0.960059
C -4.271085 1.602191 3.072061
H -5.048535 2.353137 3.197758
C -3.818440 0.872766 4.169465
H -4.245540 1.054284 5.153951
C -2.814960 -0.075994 4.012298
H -2.451804 -0.635455 4.871803
C -2.262333 -0.306633 2.756451
H -1.456922 -1.026988 2.623186
C -3.236515 -0.732399 -0.992594
C -4.568378 -0.861011 -0.598953
H -4.895301 -0.479570 0.365253
C -5.477620 -1.500814 -1.434416
H -6.513355 -1.610252 -1.119662
C -5.063297 -2.007583 -2.660914
H -5.777686 -2.510110 -3.310185
C -3.733959 -1.883490 -3.054611
H -3.405477 -2.288701 -4.009366
C -2.818212 -1.255209 -2.221758
H -1.770174 -1.173773 -2.507058
C -1.700254 1.768545 -0.653829
C -1.106333 2.714475 0.189565
H -0.994580 2.499793 1.251768
C -0.635147 3.911621 -0.331428
H -0.162568 4.638575 0.325788
C -0.761271 4.174451 -1.692814
H -0.386040 5.110194 -2.102364
C -1.366786 3.243838 -2.529785
H -1.469207 3.450846 -3.592876
C -1.831929 2.038664 -2.015201
H -2.283282 1.304028 -2.678050
Ge -0.500754 -2.663118 0.144305
C 3.574463 -1.772304 -0.473563
C 3.544213 -1.749295 0.917802
C 4.630860 -1.245184 -1.190504
C 4.566314 -1.194028 1.662040
C 5.665507 -0.671089 -0.448231
H 4.631309 -1.250591 -2.277278
C 5.632428 -0.644214 0.946463
H 4.517151 -1.159074 2.746843
H 6.508926 -0.224269 -0.969924
H 6.451356 -0.177618 1.489467
B 1.608729 -2.514367 0.191979
O 2.353645 -2.258033 1.341322
O 2.404884 -2.299232 -0.931589

F 0.594421 0.140440 -2.412876
F 0.910048 0.290084 2.280392
F 3.156032 1.750752 2.078227
F 2.823424 1.635024 -2.603514
F 4.119617 2.433473 -0.364644

^{F}YGeB1
E = -4559.21212423
P 2.050357 0.478837 0.148483
C 0.422511 0.142900 -0.292676
C -0.437133 -0.477674 0.722236
C -1.400216 0.254064 1.421836
C -2.340246 -0.347120 2.243891
C -2.286246 -1.720318 2.451410
C -1.298842 -2.471379 1.827316
C -0.390165 -1.848525 0.984263
C 2.366524 2.217067 0.595884
C 3.558486 2.586419 1.228857
H 4.312551 1.832720 1.454762
C 3.773168 3.911485 1.583396
H 4.700873 4.198053 2.074877
C 2.796394 4.869651 1.319389
H 2.964106 5.906665 1.604371
C 1.604428 4.501020 0.707114
H 0.831256 5.242270 0.514906
C 1.385444 3.175228 0.346227
H 0.442266 2.874074 -0.107360
C 3.192852 0.017376 -1.179772
C 4.261134 0.812024 -1.592104
H 4.433990 1.781581 -1.131059
C 5.092404 0.372823 -2.617742
H 5.917315 1.000486 -2.948633
C 4.864286 -0.856950 -3.224461
H 5.516269 -1.195154 -4.027631
C 3.796557 -1.651046 -2.813843
H 3.612024 -2.610334 -3.293589
C 2.954271 -1.214472 -1.801074
H 2.096491 -1.811694 -1.492276
C 2.511958 -0.443767 1.642054
C 1.984193 -0.003105 2.860555
H 1.410238 0.922297 2.899271
C 2.185116 -0.747110 4.014890
H 1.768120 -0.403555 4.959289
C 2.915675 -1.930975 3.960563
H 3.072014 -2.514928 4.865477
C 3.445163 -2.368336 2.751857
H 4.016862 -3.293009 2.708772
C 3.241638 -1.630534 1.590752
H 3.647470 -1.982961 0.645319
Ge -0.144152 0.677989 -2.041246
N -2.641873 -1.279278 -1.541579
C -4.286385 0.052492 -0.812546
C -3.931251 -1.233207 -1.016339
H -5.239381 0.441221 -0.473231
H -4.538236 -2.121434 -0.888658
B -2.138197 0.074109 -1.650429
N -3.241602 0.888702 -1.198700
C -3.243427 2.276169 -0.983942
C -3.910750 2.833549 0.109478
C -2.537935 3.115369 -1.850251

C -3.864947 4.203402 0.331011
H -4.415542 2.181176 0.818398
C -2.480194 4.482583 -1.609818
H -2.049961 2.690281 -2.725216
C -3.145678 5.036156 -0.520680
H -4.381681 4.620937 1.193285
H -1.923744 5.120863 -2.293915
H -3.108062 6.108218 -0.338885
C -1.910707 -2.462540 -1.711335
C -0.828634 -2.494043 -2.597115
C -2.201320 -3.610731 -0.968399
C -0.029608 -3.624849 -2.694602
H -0.631843 -1.633626 -3.235244
C -1.399890 -4.739366 -1.074203
H -3.023741 -3.600538 -0.257541
C -0.302840 -4.752331 -1.928042
H 0.808392 -3.623842 -3.389928
H -1.626452 -5.609205 -0.461112
H 0.328103 -5.635401 -2.000673
F -1.443465 1.574357 1.281947
F -3.279304 0.376780 2.839927
F -3.181118 -2.313518 3.227280
F -1.266243 -3.785584 2.002209
F 0.507727 -2.606287 0.362944

l
E = -3096.13968216
C -1.607267 2.354117 0.098326
C 0.515167 1.178893 0.115539
C 1.175522 2.406808 0.247703
C 0.450499 3.588846 0.289578
C -0.941833 3.566490 0.206854
H -2.696369 2.357326 0.049834
H 2.259187 2.429982 0.328298
H 0.975908 4.536449 0.392824
H -1.508022 4.495494 0.235832
N 1.118386 -0.064682 0.085288
C 2.514753 -0.224868 0.020020
C 3.276900 0.402756 -0.973208
C 3.160060 -1.073222 0.924203
C 4.646755 0.192328 -1.048038
H 2.776603 1.048561 -1.691911
C 4.529730 -1.293040 0.835112
H 2.568831 -1.553808 1.701217
C 5.281674 -0.657763 -0.146217
H 5.222970 0.687833 -1.827741
H 5.013137 -1.959610 1.547271
H 6.355111 -0.823857 -0.210615
C -0.907656 1.136928 0.058359
C -1.436691 -0.206418 -0.015790
P -3.102103 -0.571733 -0.082389
C -4.067801 -0.021561 1.358637
H -3.946351 1.058627 1.487629
H -5.131288 -0.260994 1.245256
H -3.666060 -0.518285 2.247991
C -3.981976 0.106833 -1.523367
H -5.048089 -0.146007 -1.497893
H -3.863702 1.194803 -1.544778
H -3.522310 -0.304246 -2.428223
C -3.360175 -2.359554 -0.170298

H -4.431949 -2.582289 -0.197118
H -2.878783 -2.753642 -1.071158
H -2.905808 -2.835788 0.704653
Ge -0.073629 -1.550850 -0.015532

II
E = -3645.32890364
C 0.340344 3.199252 -0.281145
C 0.115188 1.854711 -0.006467
C -1.091549 1.211218 -0.332496
C -2.086109 2.008338 -0.929407
C -1.869417 3.351082 -1.188640
C -0.653459 3.958648 -0.876746
H 1.302340 3.623355 -0.001391
H -3.033170 1.549174 -1.200534
H -2.662236 3.932617 -1.656102
H -0.489447 5.012841 -1.087408
N -1.282723 -0.155216 -0.143072
S 1.333427 0.972665 0.908955
O 2.596359 1.748136 0.865855
O 0.772727 0.685615 2.228706
C 1.535167 -0.478106 0.004753
C -2.623372 -0.592621 0.029045
C -3.349342 -0.158701 1.142774
C -3.221474 -1.472101 -0.874068
C -4.650766 -0.595793 1.342226
H -2.866711 0.515614 1.847575
C -4.521466 -1.920437 -0.660226
H -2.660124 -1.796074 -1.748307
C -5.241716 -1.482066 0.444049
H -5.205140 -0.252562 2.213870
H -4.975510 -2.608715 -1.370813
H -6.260245 -1.828631 0.606702
P 3.172571 -0.935295 -0.239815
C 4.068900 0.151780 -1.373629
H 5.120148 -0.144242 -1.462540
H 3.581977 0.116982 -2.353599
H 3.986749 1.163430 -0.963181
C 3.221807 -2.594619 -0.955680
H 2.684766 -2.601139 -1.910028
H 4.261622 -2.895956 -1.120869
H 2.738721 -3.303120 -0.274456
C 4.144211 -0.991239 1.285163
H 4.097845 0.009383 1.728095
H 3.678884 -1.706306 1.971328
H 5.184367 -1.273877 1.087795
Ge -0.002611 -1.566424 -0.300757

III
E = -4407.39598976
C -0.416075 1.796437 -0.559635
C 0.415729 1.796533 0.559395
C 0.822205 3.008174 1.116574
C 0.411607 4.211866 0.559942
C -0.412120 4.211770 -0.560472
C -0.822636 3.007982 -1.116957
H 1.473530 2.978276 1.986722
H 0.735418 5.150783 1.004833
H -0.735995 5.150611 -1.005476
H -1.473963 2.977930 -1.987098

S -0.981555 0.316412 -1.443750
S 0.981293 0.316685 1.443748
O -2.214475 0.786358 -2.110986
O 0.152952 -0.080180 -2.297510
O -0.153158 -0.079823 2.297592
O 2.214186 0.786810 2.110926
C -1.376691 -0.917258 -0.362325
C 1.376687 -0.917121 0.362493
P 2.902346 -0.777109 -0.407430
P -2.902175 -0.777418 0.407882
C 2.930450 -1.952905 -1.774856
H 3.900770 -1.933120 -2.282389
H 2.735908 -2.961644 -1.393038
H 2.129447 -1.663285 -2.465706
C 3.159749 0.858747 -1.133337
H 3.172997 1.595194 -0.322460
H 4.103006 0.897092 -1.689656
H 2.307652 1.050988 -1.796738
C 4.333206 -1.103304 0.651812
H 4.278050 -2.136157 1.011549
H 5.278864 -0.938126 0.123086
H 4.242182 -0.426087 1.507745
C -2.929666 -1.952887 1.775591
H -3.899884 -1.933317 2.283329
H -2.734870 -2.961641 1.393941
H -2.128593 -1.662828 2.466176
C -3.159936 0.858597 1.133294
H -3.173341 1.594766 0.322164
H -4.103225 0.896909 1.689559
H -2.307904 1.051275 1.796637
C -4.333115 -1.104222 -0.651076
H -5.278760 -0.939065 -0.122320
H -4.242302 -0.427243 -1.507224
H -4.277798 -2.137166 -1.010524
Ge 0.000251 -2.254861 -0.000397

IV
E = -3615.37576050
C 0.899993 3.090074 0.257686
C 0.285822 1.855281 0.356072
C -0.892376 1.469679 -0.292192
C -1.465338 2.429197 -1.132724
C -0.883421 3.689109 -1.255905
C 0.281504 4.029257 -0.563437
H 1.813061 3.316100 0.805758
H -2.380244 2.197204 -1.676976
H -1.351493 4.432906 -1.899015
H 0.704542 5.026417 -0.665402
B -1.256983 -0.045553 0.068300
S 0.893150 0.403829 1.150487
O 1.791302 0.641101 2.284473
O -0.497066 -0.206955 1.458178
C 1.500864 -0.618158 -0.076309
C -2.765191 -0.516648 0.182469
C -3.787103 0.402227 0.458724
C -3.141779 -1.857543 0.032586
C -5.115866 0.007022 0.574304
H -3.533184 1.453705 0.592529
C -4.464935 -2.267477 0.152332
H -2.376069 -2.603291 -0.188353

C -5.459320 -1.332203 0.421237
H -5.887129 0.745678 0.788764
H -4.723447 -3.318411 0.030407
H -6.498132 -1.646414 0.510378
P 3.191696 -0.909288 -0.048038
C 4.190799 0.602847 -0.014679
H 5.262232 0.373751 -0.011341
H 3.940612 1.214913 -0.887713
H 3.928603 1.150696 0.897471
C 3.652439 -1.832334 -1.524725
H 3.415961 -1.233052 -2.409652
H 4.721252 -2.071291 -1.502268
H 3.058535 -2.751212 -1.566128
C 3.730523 -1.870003 1.388232
H 3.404435 -1.320633 2.278455
H 3.227085 -2.842172 1.368148
H 4.817526 -2.008095 1.402035
Ge 0.088168 -1.245816 -1.238096

V
E = -3066.14845830
C -1.725874 2.303321 0.051779
C 0.470460 1.296584 0.252375
C 1.010075 2.572508 0.423356
C 0.209630 3.714765 0.413119
C -1.155842 3.566480 0.213335
H -2.803903 2.244411 -0.090165
H 2.083134 2.674267 0.584353
H 0.644201 4.701775 0.556598
H -1.799552 4.445344 0.190890
B 1.246611 -0.041750 0.233156
C 2.769726 -0.205158 0.008567
C 3.531756 0.700904 -0.754042
C 3.443578 -1.315573 0.550231
C 4.893249 0.514038 -0.953378
H 3.036747 1.553543 -1.216335
C 4.811764 -1.487724 0.387178
H 2.870992 -2.053009 1.112343
C 5.538695 -0.573946 -0.370801
H 5.458054 1.219980 -1.560231
H 5.311910 -2.345881 0.832970
H 6.608227 -0.715986 -0.518106
C -0.939508 1.143902 0.089725
C -1.428794 -0.241463 0.006831
P -3.095534 -0.594546 -0.222691
C -4.217375 -0.009326 1.092702
H -4.110584 1.070965 1.227894
H -5.261881 -0.247813 0.861360
H -3.924381 -0.500824 2.026598
C -3.807994 0.058869 -1.767787
H -4.874880 -0.179015 -1.847107
H -3.670708 1.143405 -1.815101
H -3.265010 -0.390879 -2.605777
C -3.401861 -2.373067 -0.301129
H -4.474615 -2.549891 -0.437969
H -2.836918 -2.805244 -1.132380
H -3.053902 -2.843664 0.623513
Ge -0.179596 -1.665164 0.291527

5.2.2 Triplet state structures

$^{Tos}Y_2Ge$

E = -5871.14951304
C -4.878176 -2.192536 0.227352
H -4.833392 -2.217114 1.313464
C -3.733728 -1.868321 -0.484408
C 3.733737 1.868297 -0.484495
O -1.259401 -2.544108 0.036943
C 3.747658 1.820174 -1.874887
H 2.834235 1.573542 -2.414615
C -2.545923 3.177081 2.494468
H -3.507283 3.511303 2.106561
C -6.056281 -2.451338 -0.463951
H -6.959598 -2.697453 0.093202
C 1.376662 -4.861355 -1.752361
H 0.974947 -5.844631 -1.517362
C 1.617022 -3.955770 -0.724062
H 1.384121 -4.225138 0.302524
C -2.336793 2.321410 -2.353698
H -2.670750 1.310173 -2.583488
C -2.110348 2.687285 -1.021097
C -5.086670 1.420137 -0.586591
H -4.710091 1.713106 -1.562933
C -6.446623 1.192687 -0.418529
H -7.119520 1.302797 -1.266636
C 1.628979 -4.507165 -3.071328
H 1.437128 -5.218932 -3.872127
C 1.644489 -0.001369 -0.221007
Ge -0.000010 -0.000054 -1.215518
O 2.611939 1.331898 1.807078
S 2.221029 1.491877 0.390313
P 2.412362 -1.458355 0.276307
C 4.931712 2.073579 -2.550050
H 4.948782 2.031276 -3.638617
C 4.714997 -0.901383 1.742740
H 4.033837 -0.755002 2.577029
C 0.604479 -1.748156 2.335713
H 0.050029 -0.977978 1.812689
C -6.077691 0.684501 1.906122
H -6.461180 0.391054 2.881335
C -4.714995 0.901398 1.742724
H -4.033823 0.755040 2.577007
C 2.336849 -2.321635 -2.353582
H 2.670838 -1.310422 -2.583427
C -1.829692 2.172524 1.834177
C -3.747654 -1.820177 -1.874785
H -2.834224 -1.573589 -2.414522
C -1.617032 3.955661 -0.724264
H -1.384167 4.225097 0.302313
C -4.213337 1.283315 0.494965
C 2.026877 -3.742233 3.651750
H 2.583989 -4.520405 4.170043
C 1.829689 -2.172447 1.834283
C 2.545944 -3.176943 2.494641
H 3.507301 -3.511185 2.106744
C 2.110349 -2.687414 -1.020964
C 2.108911 -3.234550 -3.372558
H 2.288549 -2.947177 -4.406570
C -7.395741 -2.603545 -2.591112
H -7.226715 -3.042374 -3.580771

H -7.924171 -1.651602 -2.743546
H -8.069520 -3.263206 -2.033549
C 0.793678 -3.317632 4.144849
H 0.388401 -3.771115 5.047770
C 6.107237 2.384681 -1.856583
C -6.944658 0.830727 0.828972
H -8.011150 0.656721 0.959863
C 6.446593 -1.192749 -0.418530
H 7.119479 -1.302900 -1.266640
C -1.644460 0.001308 -0.220936
P -2.412372 1.458318 0.276256
O -2.611964 -1.331876 1.807169
S -2.221024 -1.491913 0.390418
O 1.259400 2.544051 0.036788
C -6.107273 -2.384521 -1.856487
C -4.931743 -2.073497 -2.549949
H -4.948826 -2.031175 -3.638514
C -2.108823 3.234255 -3.372728
H -2.288415 2.946806 -4.406728
C -1.628911 4.506894 -3.071568
H -1.437035 5.218606 -3.872410
C -1.376643 4.861177 -1.752616
H -0.974940 5.844470 -1.517672
C 5.086637 -1.420202 -0.586564
H 4.710038 -1.713197 -1.562892
C 0.080241 -2.324289 3.486092
H -0.896018 -1.999191 3.836883
C 4.213324 -1.283343 0.495002
C 6.944645 -0.830738 0.828949
H 8.011138 -0.656721 0.959815
C 6.056257 2.451443 -0.464059
H 6.959570 2.697597 0.093082
C 6.077695 -0.684471 1.906107
H 6.461198 -0.390985 2.881303
C -0.793615 3.317892 4.144623
H -0.388314 3.771447 5.047496
C -2.026824 3.742461 3.651519
H -2.583917 4.520680 4.169761
C 4.878162 2.192547 0.227258
H 4.833394 2.217091 1.313372
C 7.395615 2.603905 -2.591307
H 8.070732 3.260835 -2.032159
H 7.226672 3.045999 -3.579538
H 7.922450 1.651611 -2.747012
C -0.080200 2.324493 3.485928
H 0.896067 1.999416 3.836720
C -0.604469 1.748270 2.335610
H -0.050042 0.978043 1.812636

^{Tos}YGeCl
E = -4435.14078424
C -3.203866 0.373978 -1.511249
H -2.888999 -0.095613 -2.440064
C -2.306957 1.182499 -0.827551
O -0.538691 2.837628 -1.839210
C 2.108911 -2.791229 -0.838439
H 1.521326 -3.410329 -0.161216
C -4.467750 0.163125 -0.976772
H -5.171075 -0.477953 -1.507167
C 0.530806 0.043325 2.854697

H -0.252440 0.729800 2.535857
C 1.200038 -0.733372 1.900110
C -1.767715 -1.682034 1.042598
H -1.579073 -1.165981 1.980378
C -2.945476 -2.399446 0.882398
H -3.669343 -2.433871 1.694012
Ge 1.565731 2.421951 0.494959
C -2.271254 -3.014625 -1.347333
H -2.469466 -3.528481 -2.285720
C -1.094789 -2.290395 -1.198246
H -0.390794 -2.211986 -2.022518
C 1.900242 -1.408184 -0.888860
C -2.665670 1.796906 0.366237
H -1.952829 2.439873 0.880517
C 2.228332 -1.584534 2.301373
H 2.775417 -2.169787 1.567192
C -0.831771 -1.632464 0.006273
C -6.198601 0.467392 0.822679
H -6.440338 1.170443 1.626439
H -6.241556 -0.546429 1.244606
H -6.986714 0.531965 0.063306
C -3.194926 -3.072381 -0.309598
H -4.116503 -3.638398 -0.432095
C 0.435800 1.061198 -0.202753
P 0.668402 -0.614588 0.170401
O -0.487740 0.386458 -2.517862
S -0.665944 1.427846 -1.489071
C -4.847594 0.744296 0.235096
C -3.929355 1.568487 0.892852
H -4.214122 2.042172 1.831455
C 0.877071 -0.049739 4.194498
H 0.353452 0.556662 4.930506
C 1.903776 -0.903263 4.591746
H 2.182108 -0.967324 5.641778
C 2.579403 -1.663317 3.645910
H 3.391921 -2.319480 3.950685
C 3.783252 -2.586753 -2.555962
H 4.521989 -3.047691 -3.208955
C 3.054182 -3.376416 -1.669320
H 3.218051 -4.451451 -1.631447
C 3.561738 -1.216192 -2.617445
H 4.121533 -0.600606 -3.317971
C 2.616985 -0.621188 -1.788210
H 2.426573 0.448004 -1.842143
Cl 3.427597 2.821798 -0.637577

^{Tos}*YGeHMDS*

E = -4848.94431010
S -0.966115 -1.619245 -0.823660
P -0.705279 1.180582 -0.101376
Si 4.074698 0.341419 0.636715
Si 3.400152 -2.521843 -0.403623
O -0.229720 -2.881577 -0.715732
O -1.153910 -1.018194 -2.159106
N 2.971926 -1.021005 0.447188
C -0.242878 -0.448423 0.213113
C -2.610675 -1.933071 -0.200036
C -2.791496 -2.222675 1.149161
H -1.929848 -2.256043 1.814129
C -4.071943 -2.448109 1.627647
H -4.218570 -2.669057 2.684414

C -5.181883 -2.400225 0.774692
C -4.967123 -2.129020 -0.576016
H -5.818105 -2.090675 -1.254988
C -3.688515 -1.892301 -1.069934
H -3.514100 -1.657287 -2.117179
C -6.564315 -2.618905 1.312056
H -6.614584 -3.522751 1.931242
H -7.297149 -2.718637 0.504765
H -6.878591 -1.778178 1.945506
C -2.481086 1.377234 -0.447806
C -3.382133 1.372641 0.620266
H -3.017032 1.325608 1.643952
C -4.747809 1.429393 0.379339
H -5.444103 1.424004 1.215337
C -5.221459 1.494262 -0.927808
H -6.292337 1.542836 -1.116201
C -4.327866 1.483862 -1.992965
H -4.696470 1.517102 -3.016256
C -2.959380 1.414477 -1.759227
H -2.261612 1.360684 -2.590738
C 0.150691 1.918935 -1.520801
C -0.231468 3.151868 -2.059368
H -1.061204 3.707070 -1.623241
C 0.428932 3.656370 -3.172312
H 0.130890 4.615769 -3.590373
C 1.458687 2.925996 -3.760090
H 1.967907 3.317409 -4.638705
C 1.826204 1.691930 -3.235933
H 2.616349 1.108508 -3.704672
C 1.176363 1.185613 -2.116201
H 1.448699 0.214416 -1.707143
C -0.365277 2.179939 1.375282
C -0.549999 1.597356 2.633939
H -0.877593 0.561816 2.701262
C -0.305164 2.334597 3.785290
H -0.446755 1.873673 4.760523
C 0.134088 3.652070 3.689789
H 0.337730 4.224504 4.592688
C 0.316790 4.234322 2.441431
H 0.667879 5.260948 2.362254
C 0.065922 3.502490 1.285316
H 0.242158 3.958965 0.315174
C 4.894805 0.726222 -1.013876
H 5.617877 1.543037 -0.886461
H 4.139642 1.059684 -1.735341
H 5.426625 -0.124688 -1.452243
C 3.141936 1.889804 1.132564
H 3.849346 2.725554 1.227812
H 2.600317 1.789363 2.082045
H 2.413643 2.158681 0.357843
C 5.380100 -0.018447 1.936994
H 6.007848 -0.872762 1.659197
H 4.902002 -0.250927 2.897358
H 6.035478 0.849949 2.086020
C 2.881735 -2.301764 -2.192548
H 3.345327 -1.401422 -2.618837
H 1.790675 -2.193945 -2.241867
H 3.165239 -3.160604 -2.814396
C 2.577282 -4.004898 0.380562
H 2.928579 -4.919618 -0.116799

H 1.488253 -3.956102 0.283634
H 2.842977 -4.073944 1.443530
C 5.254221 -2.844289 -0.288407
H 5.534103 -3.078232 0.746824
H 5.891839 -2.023664 -0.633672
H 5.492222 -3.725690 -0.899308
Ge 1.286107 -0.948079 1.261954

$^{Tos}YGeC_6F_5$
E = -4703.48190373
C 3.720587 -1.566008 -1.605013
H 3.486027 -1.105672 -2.561655
C 2.687979 -1.873017 -0.732770
O 0.296750 -2.775417 -1.340523
C 0.041947 3.429856 -1.236989
H 0.828416 3.912241 -0.657201
C 5.031074 -1.834264 -1.225107
H 5.845551 -1.588724 -1.905402
C 0.837048 0.953796 2.885770
H 1.360185 0.014658 2.714539
C 0.374638 1.700725 1.795321
C 3.446220 1.263406 0.737794
H 3.168732 0.993686 1.753202
C 4.785993 1.448091 0.424399
H 5.542218 1.316219 1.195416
C 4.186818 1.957945 -1.851933
H 4.473808 2.221217 -2.867936
C 2.844519 1.766185 -1.547340
H 2.089737 1.848683 -2.324700
C -0.215318 2.065527 -1.075600
C 2.946583 -2.460410 0.502088
H 2.122366 -2.705722 1.170407
C -0.347031 2.872199 2.018924
H -0.757597 3.431468 1.182891
C 2.466519 1.431889 -0.244394
C 6.738858 -2.645332 0.442213
H 6.853270 -3.636537 0.896628
H 7.057458 -1.908818 1.192773
H 7.430286 -2.575607 -0.403797
C 5.157430 1.802095 -0.869203
H 6.208015 1.949462 -1.112302
C 0.312268 -0.602551 0.097014
P 0.718060 1.082908 0.126626
O 1.111283 -0.601432 -2.370823
S 1.005736 -1.505770 -1.209368
C 5.322984 -2.397982 0.016489
C 4.258431 -2.712163 0.870542
H 4.465396 -3.166352 1.838910
C 0.612406 1.399242 4.181128
H 0.976214 0.814451 5.023212
C -0.094384 2.578098 4.398882
H -0.279187 2.921589 5.414825
C -0.579142 3.306901 3.319745
H -1.151726 4.216670 3.486985
C -1.691677 3.546719 -2.903870
H -2.274341 4.127572 -3.616262
C -0.701529 4.169010 -2.148460
H -0.505194 5.232022 -2.272309
C -1.929051 2.183924 -2.760898
H -2.690633 1.694489 -3.364067

C -1.187037 1.439408 -1.852890
H -1.342658 0.367857 -1.748261
Ge -0.954828 -1.484679 1.216319
C -2.807980 -1.218546 0.591089
C -4.932183 -2.051989 -0.236889
C -4.520534 0.322167 -0.191900
C -5.356640 -0.750552 -0.482490
C -3.277785 0.070634 0.369009
C -3.670796 -2.269805 0.304510
F -3.295339 -3.523262 0.513408
F -2.519867 1.121257 0.685135
F -5.733903 -3.065004 -0.521058
F -6.552593 -0.533192 -1.000065
F -4.925637 1.560998 -0.427630

TosYGePy
E = -4222.63585067
C 3.809465 -0.057159 -1.356938
H 3.637176 0.798087 -2.005906
C 2.766569 -0.936262 -1.114634
O 0.965924 -1.769959 -2.826300
C -1.223686 3.256468 0.200912
H -0.780547 3.393937 1.186993
C 5.039507 -0.276047 -0.744672
H 5.858253 0.419326 -0.925656
C -0.691817 -1.013703 2.470830
H 0.126190 -1.605128 2.063659
C -1.088840 0.160283 1.820902
C 2.133198 0.841756 1.851094
H 1.736794 0.010160 2.428303
C 3.390022 1.347312 2.154108
H 3.967407 0.905511 2.963636
C 3.171739 2.962774 0.381548
H 3.579247 3.785580 -0.202419
C 1.916275 2.455651 0.067654
H 1.358462 2.859186 -0.773034
C -1.098047 2.032296 -0.461157
C 2.942158 -2.036556 -0.280630
H 2.111593 -2.717395 -0.099674
C -2.169556 0.889460 2.315159
H -2.517295 1.774481 1.789906
C 1.382832 1.401570 0.813753
C 6.557457 -1.583719 0.786632
H 6.963472 -2.575225 0.549374
H 6.455398 -1.532558 1.878674
H 7.296577 -0.835301 0.483078
C 3.907811 2.413768 1.424476
H 4.892777 2.809622 1.664835
C 0.002878 -0.742859 -0.661116
P -0.205594 0.658372 0.317804
O 1.252053 0.723643 -2.424055
S 1.183900 -0.654424 -1.903514
C 5.239121 -1.359632 0.108726
C 4.170201 -2.238265 0.327787
H 4.307980 -3.094609 0.987230
C -1.350275 -1.427924 3.623577
H -1.035542 -2.340935 4.124496
C -2.418732 -0.689184 4.119054
H -2.939894 -1.020816 5.015123
C -2.830328 0.465277 3.461795

H -3.677927 1.035345 3.836228
C -2.439737 4.137755 -1.678966
H -2.967042 4.961022 -2.157603
C -1.902106 4.306284 -0.406140
H -2.003496 5.258333 0.111004
C -2.291874 2.925932 -2.346862
H -2.697023 2.801499 -3.348801
C -1.619896 1.870750 -1.741786
H -1.482808 0.921325 -2.252762
Ge -1.180027 -2.242223 -0.651393
C -3.058483 -1.712974 -0.581618
N -3.218872 -0.398877 -0.454285
C -5.423830 -2.066030 -0.505895
C -4.441681 0.106847 -0.358990
C -5.584882 -0.683364 -0.378143
H -6.293460 -2.721403 -0.527714
H -4.510079 1.194318 -0.267864
H -6.571081 -0.232333 -0.299218
C -4.150908 -2.596382 -0.609208
H -3.991817 -3.667764 -0.712821

[Tos]YGePyr
E = -4238.69569306
C 3.803092 -0.123450 -1.363362
H 3.653702 0.734162 -2.014876
C 2.737937 -0.975451 -1.120279
O 0.908163 -1.760531 -2.826725
C -1.132285 3.322933 0.188959
H -0.672715 3.459655 1.167630
C 5.026692 -0.373012 -0.750320
H 5.863568 0.299914 -0.933198
C -0.709489 -0.953128 2.476836
H 0.105488 -1.556645 2.081433
C -1.085784 0.222668 1.817028
C 2.159527 0.822234 1.852273
H 1.741042 0.002691 2.430916
C 3.430506 1.291963 2.153365
H 3.995994 0.834670 2.962659
C 3.255682 2.912368 0.380202
H 3.685757 3.722990 -0.204572
C 1.986046 2.440791 0.067684
H 1.438763 2.858979 -0.772862
C -1.045742 2.088933 -0.461927
C 2.884988 -2.079538 -0.285825
H 2.038302 -2.740684 -0.106398
C -2.165060 0.965274 2.294400
H -2.494983 1.853288 1.762541
C 1.424404 1.402567 0.815291
C 6.505844 -1.708018 0.794582
H 6.833733 -2.746897 0.667162
H 6.422934 -1.528133 1.875083
H 7.293438 -1.053268 0.407758
C 3.977257 2.342837 1.422358
H 4.973400 2.710695 1.661029
C -0.017379 -0.706011 -0.659642
P -0.181750 0.700694 0.319799
O 1.263155 0.725281 -2.425196
S 1.162437 -0.651167 -1.906281
C 5.197602 -1.459505 0.105857
C 4.107188 -2.311430 0.323910

H 4.223275 -3.171336 0.982806
C -1.385233 -1.355763 3.622766
H -1.086842 -2.270310 4.130617
C -2.452170 -0.603577 4.102051
H -2.987685 -0.926013 4.992939
C -2.843951 0.551833 3.435077
H -3.689690 1.132367 3.797566
C -2.355071 4.211687 -1.683021
H -2.868317 5.042668 -2.163605
C -1.793825 4.381623 -0.420171
H -1.864104 5.341912 0.086752
C -2.249200 2.989010 -2.338213
H -2.673110 2.863251 -3.332207
C -1.593910 1.924181 -1.730955
H -1.488867 0.965710 -2.232353
Ge -1.217174 -2.191389 -0.628735
C -3.096278 -1.700788 -0.570467
N -4.035401 -2.656529 -0.550725
N -3.327574 -0.384525 -0.502853
C -5.289613 -2.248717 -0.465932
C -4.588731 0.008747 -0.419030
C -5.642048 -0.899548 -0.396856
H -6.058306 -3.025485 -0.451948
H -4.765444 1.086595 -0.370483
H -6.678186 -0.578971 -0.330749

^{Tos}YGeB2
E = -4381.65898163
C 2.992475 -2.312002 -1.428531
H 2.699651 -2.020546 -2.434360
C 2.065350 -2.215529 -0.403372
O -0.505711 -2.723782 -0.501598
C 0.349708 3.338145 -1.717601
H 1.307965 3.722189 -1.368653
C 4.279396 -2.754937 -1.139955
H 5.013469 -2.824174 -1.941839
C 1.232509 1.484660 2.729096
H 1.506417 0.431564 2.690681
C 0.801652 2.129575 1.562820
C 3.545515 0.922191 0.264132
H 3.378982 0.911256 1.338365
C 4.833612 0.766671 -0.230391
H 5.663299 0.631686 0.460328
Ge -1.238471 -0.709605 1.707734
C 3.994119 0.943396 -2.481634
H 4.165569 0.942285 -3.556139
C 2.701158 1.091415 -1.993993
H 1.862358 1.175440 -2.679868
C -0.146700 2.127665 -1.221666
C 2.401983 -2.571819 0.899704
H 1.657319 -2.495375 1.690914
C 0.407343 3.466141 1.622319
H 0.039278 3.970227 0.732951
C 2.474279 1.095905 -0.615753
C 6.047492 -3.544445 0.475255
H 6.048001 -4.503134 1.008597
H 6.558615 -2.818259 1.121544
H 6.646323 -3.662318 -0.433649
C 5.059706 0.783785 -1.603331
H 6.070331 0.663776 -1.989159

C 0.098823 -0.365280 0.381007
P 0.770432 1.176002 0.017659
O 0.493123 -1.024604 -2.106901
S 0.417699 -1.617922 -0.758640
C 4.651917 -3.097026 0.159091
C 3.689816 -3.002510 1.173080
H 3.959050 -3.275318 2.193007
C 1.288947 2.178593 3.930014
H 1.623101 1.669374 4.831466
C 0.902249 3.514440 3.981957
H 0.937784 4.055125 4.925698
C 0.457747 4.153288 2.830727
H 0.138960 5.192772 2.870753
C -1.584487 3.534745 -3.137266
H -2.148936 4.086087 -3.887045
C -0.373163 4.041602 -2.671270
H 0.011366 4.983236 -3.058044
C -2.065758 2.322163 -2.657982
H -3.003966 1.917529 -3.031859
C -1.347055 1.610054 -1.703280
H -1.704021 0.647071 -1.346119
C -5.256819 -1.273930 0.727339
C -4.741575 -0.958700 -0.528056
C -6.593609 -1.563730 0.914956
C -5.537008 -0.920881 -1.655966
C -7.407951 -1.525610 -0.219467
H -6.982076 -1.809539 1.899487
C -6.891489 -1.211726 -1.476319
H -5.119504 -0.683193 -2.630780
H -8.468054 -1.748429 -0.119189
H -7.556259 -1.196084 -2.337352
B -3.107759 -0.899836 0.935627
O -4.250132 -1.237277 1.643901
O -3.406769 -0.717818 -0.409898

^{Tos}*YGeB1*
E = -4650.84416090
C 4.197993 0.917186 0.583422
H 3.932020 1.341490 1.548669
C 3.248932 0.903940 -0.427272
O 1.467815 2.728789 -1.014772
C -1.101337 -2.039670 2.534907
H -0.658860 -3.011851 2.323223
C 5.448698 0.360628 0.346227
H 6.192066 0.358070 1.142772
C 0.614908 -2.565019 -1.949105
H 1.405481 -1.850962 -2.175657
C -0.131219 -2.410906 -0.772171
C 2.849019 -2.389303 0.531315
H 2.657092 -2.777000 -0.465245
C 4.040549 -2.708242 1.169497
H 4.770219 -3.336845 0.662629
Ge -0.917859 0.730637 -1.849062
C 3.367372 -1.413600 3.084933
H 3.569920 -1.024679 4.080787
C 2.174552 -1.087651 2.451400
H 1.459040 -0.431017 2.939791
C -0.810577 -0.940327 1.719322
C 3.545202 0.370267 -1.676628
H 2.790938 0.390256 -2.461876

C -1.192859 -3.275038 -0.512488
H -1.831007 -3.127070 0.354180
C 1.904746 -1.584159 1.172958
C 7.089855 -0.875880 -1.107019
H 7.355075 -0.905196 -2.169348
H 7.072065 -1.914129 -0.745132
H 7.891616 -0.359999 -0.566903
C 4.299068 -2.225713 2.448328
H 5.232583 -2.479150 2.947432
C 0.510233 0.357549 -0.613303
P 0.365351 -1.066337 0.339764
O 1.545779 1.777098 1.338009
S 1.619390 1.578766 -0.124515
C 5.762687 -0.214719 -0.886077
C 4.794889 -0.190631 -1.896162
H 5.029297 -0.617521 -2.870753
C 0.326381 -3.597199 -2.828017
H 0.909648 -3.708831 -3.739767
C -0.716718 -4.478128 -2.549724
H -0.944871 -5.287615 -3.240475
C -1.477108 -4.307956 -1.400923
H -2.315040 -4.969853 -1.194787
C -2.460202 -0.630754 3.938237
H -3.106662 -0.510020 4.805707
C -1.936981 -1.885777 3.632315
H -2.168603 -2.743137 4.261256
C -2.141764 0.468836 3.149877
H -2.532054 1.454013 3.394127
C -1.316814 0.317713 2.039023
H -1.027073 1.175362 1.436454
N -3.666321 0.738962 -0.248341
C -3.627916 2.734083 0.769372
C -4.282014 1.552036 0.697021
H -3.864327 3.608749 1.365021
H -5.179429 1.239336 1.217892
B -2.539231 1.460159 -0.786670
C -1.601535 3.763123 -0.182855
C -1.333597 4.400570 -1.391782
C -0.893630 4.115405 0.963736
C -0.359640 5.387425 -1.448994
H -1.892231 4.109623 -2.278316
C 0.078546 5.104782 0.899039
H -1.084884 3.589006 1.896994
C 0.345379 5.744235 -0.304936
H -0.145538 5.876931 -2.396863
H 0.645502 5.357838 1.792322
H 1.116559 6.509988 -0.356485
C -4.012454 -0.611986 -0.418636
C -4.005232 -1.185710 -1.692289
C -4.374918 -1.392182 0.682096
C -4.379259 -2.511938 -1.859676
H -3.727412 -0.574183 -2.547016
C -4.757380 -2.716978 0.504456
H -4.337138 -0.958224 1.678719
C -4.768744 -3.281047 -0.766755
H -4.374328 -2.945172 -2.857975
H -5.043119 -3.311336 1.370551
H -5.077103 -4.315545 -0.905899
N -2.569924 2.731267 -0.127204

${}^{Ph}Y_2Ge$
E = -4693.95413228
P 2.625459 0.463153 -0.028416
P -2.691899 0.309372 -0.066120
C 1.844742 -0.559260 -1.155134
C 2.167832 -1.975250 -1.307012
C 3.109465 -2.650664 -0.501516
H 3.679544 -2.102035 0.242300
C 3.316479 -4.020767 -0.604568
H 4.044803 -4.495501 0.052094
C 2.600650 -4.783777 -1.518352
H 2.756480 -5.858158 -1.590944
C 1.676939 -4.138389 -2.338950
H 1.101781 -4.709650 -3.066389
C 1.471076 -2.773838 -2.239976
H 0.732784 -2.291580 -2.881049
C 2.115764 0.180480 1.700313
C 2.796685 -0.741050 2.503429
H 3.769384 -1.119785 2.196454
C 2.235462 -1.182849 3.696121
H 2.772360 -1.905171 4.307927
C 0.992370 -0.708692 4.102465
H 0.548432 -1.064382 5.030167
C 0.313715 0.219708 3.319108
H -0.660294 0.590652 3.628115
C 0.868331 0.657109 2.123991
H 0.313897 1.351918 1.500618
C 2.323873 2.217200 -0.385683
C 2.344732 3.167645 0.637914
H 2.405405 2.850427 1.676939
C 2.297788 4.526776 0.329215
H 2.309088 5.261002 1.132500
C 2.248158 4.940519 -0.995085
H 2.220275 6.002131 -1.234092
C 2.236270 3.993255 -2.020200
H 2.202018 4.312849 -3.059548
C 2.276992 2.639763 -1.719494
H 2.286080 1.894938 -2.512592
C 4.453247 0.387273 -0.018530
C 5.098970 -0.125762 -1.143893
H 4.503125 -0.544084 -1.953758
C 6.487158 -0.109621 -1.213878
H 6.988553 -0.519012 -2.088561
C 7.232403 0.424501 -0.167643
H 8.319341 0.435841 -0.224302
C 6.590334 0.946255 0.951718
H 7.172518 1.369356 1.768130
C 5.202757 0.929522 1.027144
H 4.699501 1.339826 1.902036
C -1.260228 -0.530749 -0.414443
C -1.172430 -1.983010 -0.130212
C -0.203375 -2.468695 0.764229
H 0.457531 -1.759612 1.250002
C -0.060332 -3.827005 1.009740
H 0.716279 -4.166879 1.692617
C -0.885364 -4.747641 0.372139
H -0.765241 -5.813783 0.555535
C -1.855087 -4.291438 -0.515738
H -2.500836 -5.000896 -1.031210
C -1.993226 -2.931926 -0.765679

H -2.734915 -2.593430 -1.484526
C -3.055561 0.281101 1.720455
C -3.196401 1.461696 2.455642
H -3.162167 2.425001 1.951040
C -3.382416 1.410515 3.833503
H -3.494514 2.335556 4.396028
C -3.427653 0.184167 4.488950
H -3.572588 0.146010 5.566924
C -3.290591 -0.995050 3.760011
H -3.326346 -1.957826 4.266107
C -3.107268 -0.952507 2.384065
H -2.998782 -1.879634 1.824549
C -2.610445 2.052479 -0.551433
C -3.741104 2.703994 -1.047622
H -4.670132 2.156202 -1.185395
C -3.683609 4.056274 -1.372067
H -4.568785 4.554471 -1.762170
C -2.499355 4.763376 -1.200436
H -2.454456 5.820329 -1.457221
C -1.371323 4.122151 -0.698018
H -0.440980 4.665957 -0.558768
C -1.424059 2.771872 -0.372218
H -0.537658 2.273724 0.008950
C -4.236953 -0.293410 -0.850541
C -5.425446 -0.504476 -0.153253
H -5.470266 -0.314539 0.917264
C -6.555823 -0.959756 -0.827654
H -7.480038 -1.131411 -0.279058
C -6.504293 -1.194534 -2.196369
H -7.389012 -1.550713 -2.720892
C -5.318759 -0.980342 -2.897449
H -5.276265 -1.166900 -3.968795
C -4.187455 -0.538650 -2.226072
H -3.246223 -0.392510 -2.758916
Ge 0.082034 0.079758 -1.700508

^{Ph}YGeCl
E = -3846.56536613
P 0.521185 -0.338704 -0.041654
C -0.970528 0.552946 0.215589
C -2.270839 -0.052224 0.123840
C -2.523388 -1.433483 0.283654
H -1.699444 -2.103925 0.514882
C -3.809648 -1.940754 0.201941
H -3.979686 -3.006558 0.343360
C -4.883759 -1.090090 -0.052186
H -5.893026 -1.491476 -0.121641
C -4.660831 0.279130 -0.207665
H -5.497148 0.947143 -0.404795
C -3.381630 0.795400 -0.109626
H -3.193264 1.860624 -0.234347
C 1.110291 -1.174505 1.457030
C 2.183165 -2.072659 1.409057
H 2.666764 -2.296576 0.459243
C 2.622036 -2.692671 2.571736
H 3.457068 -3.389085 2.530654
C 1.991887 -2.425741 3.784118
H 2.336033 -2.914443 4.693518
C 0.920998 -1.540203 3.833606
H 0.424171 -1.333179 4.778854

C 0.477938 -0.914003 2.674129
H -0.363078 -0.222624 2.713517
C 1.770735 0.833001 -0.616693
C 3.118258 0.714995 -0.267849
H 3.444355 -0.046956 0.434789
C 4.046603 1.608745 -0.789892
H 5.092540 1.524477 -0.501755
C 3.641636 2.611454 -1.663156
H 4.371684 3.312779 -2.062335
C 2.299087 2.723983 -2.018621
H 1.968645 3.518907 -2.683330
C 1.365099 1.838977 -1.503415
H 0.310830 1.962144 -1.743202
C 0.421207 -1.638054 -1.325336
C 0.255817 -2.989332 -1.006928
H 0.251850 -3.311267 0.032492
C 0.096640 -3.932039 -2.014709
H -0.026332 -4.981668 -1.755525
C 0.093060 -3.535801 -3.347768
H -0.032889 -4.275291 -4.136059
C 0.251054 -2.192358 -3.671209
H 0.249365 -1.875971 -4.712146
C 0.415904 -1.246802 -2.666986
H 0.547954 -0.199859 -2.930401
Ge -0.631306 2.276821 1.267744
Cl -1.149244 3.765742 -0.406748

^{Ph}YGeHMDS
E = -4260.34931483
P 1.796607 0.171820 -0.063323
C 0.348219 -0.479295 -0.698533
C -0.050798 -1.876545 -0.614021
C 0.572676 -2.796948 0.253726
H 1.367243 -2.456224 0.913914
C 0.178114 -4.126360 0.311255
H 0.689639 -4.802557 0.994808
C -0.874491 -4.590452 -0.470715
H -1.192925 -5.629130 -0.413856
C -1.518144 -3.694725 -1.322273
H -2.345090 -4.033913 -1.945147
C -1.113688 -2.371219 -1.396425
H -1.619070 -1.688184 -2.077022
C 1.820783 0.483194 1.730506
C 2.277653 -0.486514 2.629576
H 2.842654 -1.343702 2.266456
C 2.005358 -0.369337 3.986751
H 2.366460 -1.129545 4.676396
C 1.264124 0.710424 4.461095
H 1.043428 0.794395 5.523409
C 0.807228 1.680295 3.575639
H 0.223930 2.523831 3.939901
C 1.083744 1.573390 2.217580
H 0.710991 2.325990 1.524785
C 2.116138 1.780464 -0.845786
C 2.850424 2.770519 -0.186904
H 3.187713 2.610121 0.835227
C 3.142979 3.964765 -0.833268
H 3.708388 4.735478 -0.313179
C 2.712420 4.176110 -2.139843
H 2.938656 5.114970 -2.641663

C 2.000834 3.184077 -2.806579
H 1.672958 3.341842 -3.832041
C 1.709757 1.984194 -2.168979
H 1.166463 1.196252 -2.686895
C 3.264482 -0.829843 -0.443398
C 3.198932 -1.783640 -1.460671
H 2.253248 -1.963979 -1.966651
C 4.338558 -2.496553 -1.813649
H 4.282264 -3.245422 -2.600957
C 5.544636 -2.255255 -1.163738
H 6.434871 -2.815614 -1.443191
C 5.615848 -1.294466 -0.159620
H 6.560221 -1.098282 0.344143
C 4.479449 -0.580209 0.199586
H 4.536263 0.174949 0.982567
Ge -1.046094 0.869528 -0.787934
N -2.726982 0.346774 -0.147606
Si -4.084624 0.306638 -1.273587
Si -2.871646 0.242243 1.615118
C -3.496579 0.575864 -3.036617
H -3.026795 1.558346 -3.167783
H -2.773863 -0.182533 -3.361648
H -4.365447 0.522753 -3.707993
C -4.949850 -1.359870 -1.210630
H -5.542390 -1.495029 -0.299892
H -5.628207 -1.458965 -2.068578
H -4.212945 -2.171286 -1.260001
C -5.297543 1.688096 -0.876114
H -6.149406 1.677529 -1.568921
H -5.696126 1.615414 0.142865
H -4.798614 2.661717 -0.968449
C -4.536812 -0.481394 2.111316
H -4.619353 -0.423376 3.205209
H -5.399085 0.045416 1.687152
H -4.605729 -1.539076 1.830948
C -2.737854 1.967953 2.347069
H -2.696754 1.940464 3.444013
H -1.834373 2.470264 1.980533
H -3.602471 2.576417 2.050053
C -1.577831 -0.908487 2.320482
H -0.557398 -0.623320 2.048837
H -1.645202 -0.921096 3.416929
H -1.744166 -1.928263 1.950366

$^{Ph}YGeC_6F_5$
E = -4114.89071003
P -1.563640 -0.018080 0.183063
C -0.348741 0.577337 -0.952867
C 0.391680 1.801468 -0.635203
C 1.181381 1.915224 0.524315
H 1.208255 1.098509 1.241342
C 2.018677 3.008996 0.709272
H 2.654058 3.049343 1.592199
C 2.059853 4.034105 -0.227185
H 2.716899 4.888303 -0.077693
C 1.266983 3.948408 -1.370360
H 1.298525 4.740010 -2.116678
C 0.460957 2.841518 -1.582813
H -0.119781 2.751654 -2.498834
C -0.979232 -0.097253 1.892865

C -1.288994 0.925984 2.793035
H -2.011330 1.693001 2.523129
C -0.657466 0.973590 4.029954
H -0.900874 1.772969 4.726694
C 0.290768 0.014318 4.371221
H 0.794828 0.066113 5.334187
C 0.595245 -1.012188 3.480742
H 1.339431 -1.764931 3.731481
C -0.038372 -1.073056 2.247448
H 0.203678 -1.875758 1.558119
C -2.155134 -1.635575 -0.325343
C -2.331901 -2.688715 0.573262
H -2.100989 -2.557390 1.627258
C -2.809029 -3.912441 0.118715
H -2.939813 -4.733828 0.819657
C -3.117400 -4.083430 -1.228290
H -3.488666 -5.043632 -1.581399
C -2.960900 -3.031113 -2.121707
H -3.209848 -3.161336 -3.172433
C -2.477391 -1.804646 -1.678006
H -2.381381 -0.975345 -2.374051
C -3.014818 1.076289 0.194935
C -2.871849 2.444175 -0.050220
H -1.895508 2.859206 -0.287480
C -3.985370 3.274950 0.006629
H -3.871045 4.339698 -0.185430
C -5.237843 2.747461 0.301697
H -6.107430 3.400598 0.341413
C -5.381984 1.383949 0.540069
H -6.362150 0.968552 0.764947
C -4.274705 0.546770 0.487098
H -4.388617 -0.520906 0.666030
C 1.986855 -0.766967 -1.346183
C 2.823061 -1.881410 0.653217
C 3.999743 0.020026 -0.219220
C 3.877531 -0.980888 0.735670
Ge 0.401441 -0.375265 -2.618433
C 3.058150 0.108575 -1.238802
C 1.916328 -1.765217 -0.390913
F 0.884249 -2.623862 -0.390936
F 3.178052 1.138425 -2.073345
F 4.985136 0.901395 -0.120364
F 4.749933 -1.068567 1.728600
F 2.668603 -2.811456 1.595297

^{Ph}YGePy
E = -3634.04599361
P -0.978723 -0.388302 0.059771
C 0.222944 0.734562 -0.460859
C 0.045777 2.184607 -0.475368
C 1.150786 2.992411 -0.138944
C 1.057556 4.376113 -0.148016
C -0.138724 5.000837 -0.493039
C -1.240356 4.221911 -0.836521
C -1.149249 2.837322 -0.834007
C -0.403697 -1.422394 1.438766
C -1.291323 -2.298497 2.072852
H -2.320177 -2.383187 1.723521
C -0.863192 -3.051308 3.158132
H -1.552586 -3.735193 3.649404

C 0.443940 -2.920433 3.621982
H 0.777335 -3.509373 4.474599
C 1.315486 -2.027976 3.008525
H 2.331650 -1.909306 3.379476
C 0.895887 -1.271464 1.918231
H 1.568585 -0.547427 1.458684
C -1.547156 -1.492117 -1.269132
C -1.563485 -2.883129 -1.171016
H -1.213414 -3.373389 -0.266424
C -2.001129 -3.648527 -2.247193
H -2.000440 -4.733948 -2.170074
C -2.427290 -3.032783 -3.418129
H -2.767040 -3.635808 -4.257932
C -2.404926 -1.644058 -3.522140
H -2.722590 -1.158807 -4.442724
C -1.958283 -0.876100 -2.456688
H -1.902578 0.208523 -2.547910
C -2.470054 0.393602 0.749218
C -2.285582 1.294618 1.803167
H -1.281279 1.495116 2.174530
C -3.378022 1.941887 2.360781
H -3.229701 2.647780 3.175282
C -4.659577 1.692152 1.875166
H -5.515514 2.202083 2.313130
C -4.846222 0.791964 0.833286
H -5.847180 0.592576 0.455743
C -3.753466 0.141499 0.266485
H -3.903439 -0.559811 -0.551529
C 3.381082 -0.118564 -0.492052
C 5.724012 -0.682280 -0.453940
C 4.648924 0.467905 1.337244
C 5.807481 -0.082821 0.803239
Ge 1.656846 -0.111930 -1.469036
N 3.473637 0.452124 0.716373
H 6.604391 -1.129091 -0.914328
H 6.744132 -0.042616 1.354476
H 4.670085 0.947488 2.318837
H 2.073981 2.495001 0.155188
H 1.925922 4.974559 0.123010
H -0.210959 6.086847 -0.499800
H -2.177508 4.697690 -1.121123
H -2.015473 2.247245 -1.127868
C 4.505289 -0.699052 -1.107750
H 4.405521 -1.154832 -2.091856

^{Ph}YGePyr
E = -3650.10506049
P -0.974307 -0.399161 0.056900
C 0.167716 0.645525 -0.689797
C -0.024500 2.103222 -0.726837
C 0.976419 2.940812 -0.201828
C 0.842943 4.322450 -0.231047
C -0.290540 4.908503 -0.787515
C -1.285911 4.096919 -1.324883
C -1.153120 2.715312 -1.300293
C -0.600927 -0.741529 1.808659
C -1.572671 -1.273916 2.663179
H -2.575593 -1.479016 2.290246
C -1.259450 -1.531790 3.991230
H -2.015070 -1.945933 4.656069

C 0.018299 -1.252178 4.471379
H 0.260464 -1.450974 5.513895
C 0.978274 -0.711411 3.624163
H 1.974131 -0.482771 3.998988
C 0.673900 -0.451335 2.290926
H 1.422493 -0.009217 1.632314
C -1.063225 -2.001991 -0.789171
C -1.074286 -3.211239 -0.093980
H -0.980931 -3.220866 0.989074
C -1.180679 -4.410907 -0.790261
H -1.174375 -5.352020 -0.244220
C -1.286080 -4.408595 -2.175930
H -1.366626 -5.349375 -2.717114
C -1.278965 -3.203279 -2.873366
H -1.350603 -3.198331 -3.958957
C -1.161524 -2.003700 -2.185803
H -1.125646 -1.059451 -2.726559
C -2.672560 0.252488 0.139367
C -2.917567 1.322968 1.005864
H -2.119252 1.696607 1.645811
C -4.171251 1.916818 1.040855
H -4.353547 2.753977 1.711454
C -5.189475 1.444967 0.216636
H -6.172467 1.911201 0.245402
C -4.952118 0.376193 -0.640120
H -5.748616 0.000812 -1.279512
C -3.695656 -0.220123 -0.682561
H -3.515900 -1.056872 -1.354051
C 3.379703 -0.073325 -0.490921
C 5.602232 -0.575690 -0.443804
C 4.587568 0.612064 1.322217
C 5.744644 0.054847 0.792557
Ge 1.711724 -0.163773 -1.530246
N 3.419832 0.553045 0.697914
N 4.447581 -0.641953 -1.080025
H 6.461224 -1.042914 -0.932979
H 6.699637 0.107584 1.308475
H 4.602884 1.126111 2.286923
H 1.855887 2.473835 0.239765
H 1.630502 4.947927 0.186378
H -0.394319 5.991844 -0.810871
H -2.168974 4.544450 -1.778341
H -1.924912 2.091397 -1.746514

^{Ph}YGeB2
E = -3793.06992151
P -1.352431 -0.515601 -0.112458
C -0.467189 0.819881 0.522915
C -1.064594 2.169264 0.466214
C -1.494232 2.835587 1.624935
C -2.027191 4.117515 1.563790
C -2.154710 4.769020 0.341520
C -1.731331 4.126428 -0.818023
C -1.189727 2.849437 -0.756639
C -2.600626 -1.256385 1.000303
C -3.637051 -2.045880 0.491163
H -3.718236 -2.213660 -0.581959
C -4.569023 -2.609589 1.353329
H -5.374170 -3.223429 0.954190
C -4.475698 -2.383457 2.724199

H -5.209452 -2.822170 3.397815
C -3.451509 -1.592212 3.231769
H -3.380621 -1.408992 4.301934
C -2.515035 -1.026676 2.372553
H -1.711017 -0.403018 2.762013
C -0.221772 -1.847245 -0.587497
C -0.486446 -3.193056 -0.332726
H -1.391657 -3.484317 0.194826
C 0.425713 -4.163216 -0.734166
H 0.223814 -5.211619 -0.524318
C 1.596439 -3.795478 -1.388534
H 2.310731 -4.558248 -1.692744
C 1.860344 -2.453050 -1.646744
H 2.781418 -2.159568 -2.146843
C 0.955115 -1.478752 -1.248055
H 1.169076 -0.425745 -1.417919
C -2.329164 -0.064722 -1.575611
C -3.482918 0.702806 -1.385968
H -3.804991 0.959889 -0.377846
C -4.208494 1.147959 -2.481982
H -5.101135 1.750901 -2.329335
C -3.790246 0.828856 -3.770902
H -4.360471 1.178356 -4.629485
C -2.645719 0.062746 -3.962539
H -2.320410 -0.191766 -4.969212
C -1.911918 -0.383075 -2.868054
H -1.016354 -0.981561 -3.021062
H -1.403158 2.326764 2.583651
H -2.349677 4.608868 2.480440
H -2.574137 5.772136 0.293378
H -1.811945 4.628658 -1.780834
H -0.839031 2.364079 -1.665355
Ge 0.916289 0.385005 1.806765
C 4.422786 0.555283 -0.497662
C 4.950598 0.196723 0.741347
C 5.220182 0.703692 -1.615750
C 6.301623 -0.028119 0.919287
C 6.588601 0.476616 -1.445678
H 4.795698 0.988212 -2.575037
C 7.117019 0.119231 -0.205817
H 6.700336 -0.306466 1.891063
H 7.254867 0.583642 -2.299111
H 8.187811 -0.048279 -0.110837
B 2.770467 0.425295 0.957375
O 3.942008 0.118467 1.647279
O 3.077070 0.707022 -0.377246

^{Ph}YGeB1
E = -4062.25856791
P 0.624510 -1.417829 -0.012810
C -0.713761 -0.633534 -0.773589
C -2.082528 -1.039635 -0.470878
C -2.459769 -1.584446 0.774878
H -1.714477 -1.699847 1.560555
C -3.767935 -1.968917 1.035550
H -4.016249 -2.384736 2.011074
C -4.753814 -1.824459 0.066323
H -5.778661 -2.129501 0.268432
C -4.408631 -1.269394 -1.164388
H -5.169321 -1.132452 -1.931440

C -3.105265 -0.880641 -1.427188
H -2.845150 -0.447071 -2.392138
C 1.165255 -0.615444 1.533240
C 2.400863 -0.893367 2.127820
H 3.109953 -1.551925 1.628259
C 2.725516 -0.325072 3.353164
H 3.687291 -0.543670 3.813159
C 1.821679 0.522353 3.989554
H 2.076635 0.963018 4.951740
C 0.602407 0.819057 3.389795
H -0.096306 1.501076 3.868765
C 0.274379 0.256801 2.160522
H -0.671667 0.497472 1.675704
C 2.099057 -1.501194 -1.071282
C 3.084722 -0.513256 -1.008943
H 2.999008 0.308979 -0.302576
C 4.187201 -0.583056 -1.853078
H 4.952736 0.185666 -1.790470
C 4.303851 -1.620315 -2.771022
H 5.166781 -1.667560 -3.432720
C 3.318772 -2.599754 -2.841810
H 3.405131 -3.414337 -3.557981
C 2.222597 -2.545960 -1.990408
H 1.462179 -3.321320 -2.042580
C 0.251477 -3.153875 0.417694
C 0.726557 -3.772849 1.574009
H 1.335942 -3.218411 2.283118
C 0.408463 -5.101686 1.834114
H 0.781770 -5.577247 2.738961
C -0.387265 -5.817115 0.946660
H -0.638163 -6.855372 1.155453
C -0.872508 -5.200828 -0.202766
H -1.509184 -5.751216 -0.892331
C -0.560576 -3.873734 -0.465497
H -0.963446 -3.379627 -1.348194
Ge -0.246139 0.906838 -1.873354
N -1.547855 2.765242 0.309500
C 0.160429 3.664619 1.440899
C -1.181788 3.510273 1.424867
H 0.769555 4.235557 2.132055
H -1.922702 3.932322 2.094626
B -0.355591 2.392245 -0.408430
N 0.709950 3.026052 0.333984
C 2.092174 2.917750 0.127690
C 2.958730 2.710468 1.202684
C 2.614479 3.005587 -1.166193
C 4.326079 2.585914 0.985854
H 2.551070 2.618389 2.205746
C 3.981571 2.883918 -1.374020
H 1.937394 3.179684 -1.998455
C 4.845025 2.672984 -0.301938
H 4.988363 2.413533 1.832343
H 4.375211 2.957568 -2.386091
H 5.916492 2.581029 -0.469137
C -2.891314 2.446336 0.024097
C -3.405582 2.679979 -1.251649
C -3.718312 1.915716 1.014039
C -4.737670 2.398016 -1.525616
H -2.751976 3.095848 -2.014525
C -5.050438 1.639686 0.733906

H -3.304113 1.702603 1.997947
C -5.566837 1.886038 -0.533444
H -5.131677 2.589462 -2.522026
H -5.683812 1.212123 1.508462
H -6.610273 1.666519 -0.750390

$^{F}Y_2Ge$
E = -5687.76630803
P -2.793499 -1.083380 -0.053935
P 2.992093 -0.919022 0.078701
C -1.627156 -0.034705 -0.755664
C -1.703123 1.393558 -0.921991
C -2.652169 2.213016 -0.283880
C -2.654492 3.596341 -0.360137
C -1.676270 4.252965 -1.095891
C -0.715567 3.488665 -1.747905
C -0.756403 2.107627 -1.684013
C -2.919869 -1.086385 1.762906
C -3.971575 -0.460419 2.434707
H -4.794244 -0.024055 1.873747
C -3.958043 -0.373387 3.821910
H -4.779802 0.119327 4.337504
C -2.895434 -0.902165 4.546691
H -2.885938 -0.826705 5.632405
C -1.841894 -1.522182 3.881940
H -1.005075 -1.931513 4.444732
C -1.853470 -1.616624 2.497317
H -1.027856 -2.088053 1.965925
C -2.303356 -2.760427 -0.536062
C -2.461064 -3.867940 0.312514
H -2.829204 -3.731401 1.326449
C -2.138925 -5.140415 -0.136446
H -2.253781 -5.989501 0.535262
C -1.673500 -5.337257 -1.436161
H -1.416534 -6.337268 -1.779393
C -1.564479 -4.250823 -2.300375
H -1.230366 -4.399574 -3.325279
C -1.894337 -2.974364 -1.870155
H -1.838093 -2.130993 -2.554609
C -4.485901 -0.894365 -0.693650
C -4.731623 -0.035199 -1.764001
H -3.921467 0.567998 -2.168573
C -6.007859 0.044185 -2.310508
H -6.197710 0.720349 -3.141610
C -7.037031 -0.736698 -1.796568
H -8.035243 -0.671892 -2.225354
C -6.791333 -1.604950 -0.736228
H -7.594217 -2.221876 -0.337578
C -5.518562 -1.687058 -0.187065
H -5.325419 -2.372001 0.638158
C 1.535409 -0.047020 0.226521
C 1.397836 1.365967 0.515877
C 0.368696 1.826888 1.356949
C 0.114683 3.173489 1.558251
C 0.926435 4.131710 0.963346
C 1.975675 3.724511 0.149004
C 2.179871 2.371773 -0.072629
C 4.186480 -0.471091 1.369381
C 4.244678 -1.171598 2.576306
H 3.616639 -2.046151 2.730225

C 5.113039 -0.757634 3.578570
H 5.159192 -1.311500 4.514061
C 5.921088 0.359479 3.386964
H 6.597503 0.683357 4.175606
C 5.866887 1.058114 2.186283
H 6.499018 1.930126 2.031316
C 5.007154 0.644338 1.175080
H 4.975309 1.185052 0.232141
C 2.509877 -2.660951 0.272498
C 2.946291 -3.674793 -0.579068
H 3.646187 -3.450863 -1.380549
C 2.463069 -4.970288 -0.420351
H 2.798690 -5.756212 -1.093559
C 1.546550 -5.257511 0.587324
H 1.162204 -6.269768 0.698470
C 1.108562 -4.252812 1.441915
H 0.381241 -4.473550 2.221021
C 1.576179 -2.952445 1.280669
H 1.236231 -2.147202 1.929211
C 3.941255 -0.850939 -1.476297
C 5.257774 -1.314799 -1.536030
H 5.746690 -1.687837 -0.636544
C 5.944924 -1.294256 -2.744478
H 6.971438 -1.652707 -2.791351
C 5.320531 -0.809126 -3.889961
H 5.860696 -0.790447 -4.834770
C 4.013148 -0.336400 -3.828021
H 3.530836 0.056621 -4.720556
C 3.321813 -0.353467 -2.622236
H 2.307706 0.038145 -2.552206
Ge 0.060715 -0.979332 -0.652863
F 3.151908 2.029641 -0.919744
F 2.742278 4.626259 -0.442442
F 0.675685 5.417353 1.141035
F -0.907332 3.555506 2.309542
F -0.435909 0.959150 1.955741
F 0.210288 1.440952 -2.324021
F 0.259210 4.088065 -2.417813
F -1.640407 5.576147 -1.154830
F -3.577216 4.293485 0.283885
F -3.589977 1.639211 0.478974

^{F}YGeCl
E = -4343.46281835
P 0.970423 0.509261 0.173070
C -0.017041 -0.759326 -0.491087
C -1.474730 -0.648821 -0.392620
C -2.281089 -0.195283 -1.438312
C -3.661128 -0.106415 -1.318641
C -4.270494 -0.456358 -0.119549
C -3.496791 -0.903262 0.945103
C -2.121047 -0.998998 0.796686
C 1.377458 1.880255 -0.948467
C 1.830724 3.093290 -0.418296
H 1.925871 3.216427 0.659862
C 2.147706 4.144755 -1.268347
H 2.501881 5.086924 -0.855040
C 2.004060 3.993756 -2.645036
H 2.247433 4.820588 -3.309491
C 1.540496 2.793206 -3.171379

H 1.416226 2.678118 -4.245937
C 1.226100 1.734500 -2.326374
H 0.848782 0.799017 -2.734523
C 2.496607 -0.199330 0.814964
C 3.730060 0.447066 0.708103
H 3.810196 1.396409 0.183653
C 4.863288 -0.144056 1.252579
H 5.827089 0.352180 1.159638
C 4.769021 -1.371247 1.901772
H 5.661694 -1.834073 2.317997
C 3.538774 -2.013142 2.010990
H 3.464854 -2.980160 2.503200
C 2.400141 -1.431293 1.472445
H 1.438861 -1.939237 1.527519
C 0.074392 1.307191 1.535641
C -0.988528 2.157301 1.210770
H -1.189348 2.403101 0.168338
C -1.786479 2.684696 2.216318
H -2.614092 3.343264 1.961234
C -1.526624 2.368869 3.547533
H -2.154144 2.780994 4.335238
C -0.467990 1.528093 3.872096
H -0.264811 1.282429 4.912231
C 0.331932 0.991485 2.869293
H 1.152299 0.323957 3.123726
Ge 0.913556 -1.913577 -1.765100
Cl 1.493289 -3.815639 -0.742242
F -1.404644 -1.424903 1.829961
F -4.077060 -1.235039 2.086543
F -5.583069 -0.366577 0.006989
F -4.397498 0.329421 -2.327837
F -1.724590 0.180418 -2.584486

^{F}YGeHMDS
E = -4757.25462418
P -1.951735 -0.296532 -0.147018
C -0.388479 0.179429 -0.667237
C 0.248855 1.464416 -0.511283
C -0.163511 2.453707 0.398005
C 0.486914 3.664553 0.566476
C 1.618348 3.954131 -0.185761
C 2.067514 3.008728 -1.100407
C 1.382130 1.817864 -1.269958
C -2.175784 -0.753100 1.603958
C -2.681427 0.153912 2.539480
H -3.106344 1.097839 2.207735
C -2.615614 -0.132678 3.897298
H -3.009473 0.581874 4.617066
C -2.035660 -1.319888 4.335629
H -1.976238 -1.536026 5.400658
C -1.536595 -2.231085 3.411455
H -1.084154 -3.161757 3.748142
C -1.609858 -1.955534 2.050928
H -1.212252 -2.666546 1.328518
C -2.384547 -1.790450 -1.088477
C -3.264427 -2.746904 -0.571679
H -3.659393 -2.637562 0.436188
C -3.629432 -3.843852 -1.341129
H -4.308854 -4.588205 -0.930742
C -3.126259 -3.992776 -2.630811

H -3.409880 -4.856672 -3.228815
C -2.268892 -3.032840 -3.157082
H -1.883236 -3.139661 -4.168906
C -1.903777 -1.927945 -2.396751
H -1.247482 -1.163235 -2.806715
C -3.251570 0.903773 -0.551906
C -2.973976 1.967558 -1.410529
H -1.967355 2.098448 -1.800623
C -3.986940 2.849558 -1.769589
H -3.765705 3.682262 -2.434026
C -5.277235 2.667176 -1.284381
H -6.067898 3.359257 -1.567659
C -5.560062 1.596868 -0.440742
H -6.570787 1.447618 -0.066427
C -4.551144 0.715210 -0.075602
H -4.773501 -0.122946 0.583675
Ge 0.772708 -1.396536 -0.674073
N 2.459631 -1.082872 0.067884
Si 3.889125 -1.346031 -0.946837
Si 2.502670 -1.007605 1.840051
C 3.397236 -1.773381 -2.704916
H 2.825034 -2.708656 -2.749263
H 2.799510 -0.984563 -3.173350
H 4.311121 -1.910347 -3.300231
C 4.956639 0.195283 -0.969750
H 5.362971 0.438561 0.017599
H 5.804093 0.049702 -1.653031
H 4.381577 1.056880 -1.328028
C 4.868289 -2.814237 -0.291884
H 5.759525 -2.989910 -0.908820
H 5.201823 -2.684670 0.744048
H 4.250007 -3.720871 -0.330950
C 4.176744 -0.389469 2.435875
H 4.176055 -0.409625 3.534199
H 5.029287 -0.986290 2.094597
H 4.339155 0.650115 2.126230
C 2.191152 -2.721185 2.543073
H 2.125522 -2.701932 3.638848
H 1.251291 -3.128284 2.149806
H 2.999026 -3.408220 2.259410
C 1.257612 0.226172 2.501966
H 0.251740 0.111240 2.088404
H 1.182635 0.119690 3.592815
H 1.596531 1.247657 2.286531
F 1.847079 0.977313 -2.192278
F -1.209986 2.206062 1.191411
F 0.042141 4.540786 1.455350
F 2.252856 5.107482 -0.038302
F 3.139576 3.265569 -1.838078

$^{F}YGeC_6F_5$
E = -4611.80588829
P -0.475539 -1.350057 -0.353411
C -0.628899 0.019615 0.716534
C -1.715846 0.979660 0.599881
C -2.635990 1.191208 1.631708
C -3.649373 2.134405 1.534403
C -3.767864 2.903299 0.382779
C -2.869631 2.720858 -0.662525
C -1.865004 1.774813 -0.541454

C -0.867724 -2.920647 0.461888
C -0.718913 -4.108986 -0.259734
H -0.324174 -4.086781 -1.275140
C -1.074461 -5.318689 0.321894
H -0.949628 -6.244719 -0.235479
C -1.594732 -5.342909 1.613108
H -1.874851 -6.291638 2.066664
C -1.763779 -4.158093 2.321589
H -2.178357 -4.175746 3.327129
C -1.400102 -2.943569 1.750520
H -1.533382 -2.013038 2.299256
C 1.205341 -1.437367 -1.008662
C 2.109979 -2.418688 -0.602392
H 1.797712 -3.199296 0.087102
C 3.424673 -2.374557 -1.054379
H 4.132899 -3.129813 -0.721822
C 3.837875 -1.355904 -1.905236
H 4.871482 -1.315943 -2.242689
C 2.940862 -0.364229 -2.295438
H 3.271437 0.450252 -2.936917
C 1.629579 -0.394197 -1.842092
H 0.937928 0.402725 -2.108848
C -1.649032 -1.282952 -1.738125
C -3.001903 -1.176319 -1.396596
H -3.299033 -1.164076 -0.348402
C -3.966067 -1.094519 -2.390912
H -5.016205 -1.008316 -2.120143
C -3.587306 -1.124685 -3.730175
H -4.343147 -1.057539 -4.510052
C -2.245547 -1.246694 -4.072082
H -1.949648 -1.279757 -5.118518
C -1.273150 -1.324904 -3.080491
H -0.226359 -1.423438 -3.356445
C 2.216911 0.845206 1.217124
C 4.524838 0.807685 0.446515
C 3.080984 2.611776 -0.218223
C 4.326073 1.993327 -0.247497
Ge 0.701758 -0.063795 2.169085
C 2.052290 2.031273 0.511293
C 3.474180 0.257900 1.168421
F 3.699998 -0.902936 1.782145
F 0.876166 2.658127 0.508974
F 2.892861 3.738586 -0.890464
F 5.310700 2.518495 -0.960254
F 5.692266 0.183745 0.361422
F -1.006351 1.639364 -1.549237
F -2.973748 3.456213 -1.756483
F -4.730916 3.802654 0.281311
F -4.512476 2.294688 2.523688
F -2.573990 0.447811 2.731664

^{F}YGePy
E = -4130.94998112
P 0.039907 -1.310290 0.210482
C 0.155529 0.234378 -0.529132
C -1.018597 1.112169 -0.427569
C -1.210895 1.957646 0.668063
C -2.339282 2.756803 0.795003
C -3.324326 2.713265 -0.183914
C -3.175255 1.872105 -1.280314

C -2.040463 1.080914 -1.380280
C 0.896192 -1.469141 1.803204
C 0.639566 -2.553446 2.650001
H -0.078888 -3.318916 2.357036
C 1.291714 -2.644421 3.871892
H 1.092670 -3.485858 4.532738
C 2.193775 -1.652460 4.252321
H 2.702651 -1.723695 5.211997
C 2.433446 -0.568149 3.416773
H 3.127119 0.213814 3.719651
C 1.782721 -0.464922 2.189996
H 1.948536 0.403809 1.549080
C 0.655719 -2.622581 -0.882257
C 1.453048 -3.674038 -0.429673
H 1.763570 -3.718536 0.611174
C 1.873165 -4.657061 -1.319595
H 2.504200 -5.469557 -0.965202
C 1.496977 -4.597552 -2.656488
H 1.829494 -5.368131 -3.349417
C 0.704857 -3.547109 -3.112122
H 0.418157 -3.491872 -4.160214
C 0.291073 -2.556362 -2.232526
H -0.307400 -1.716540 -2.583192
C -1.702543 -1.643656 0.597166
C -2.247066 -1.031683 1.731074
H -1.596290 -0.497910 2.423208
C -3.616009 -1.086010 1.959865
H -4.036596 -0.601990 2.838913
C -4.445547 -1.751578 1.062032
H -5.518358 -1.790965 1.240246
C -3.905378 -2.366110 -0.062702
H -4.553208 -2.888484 -0.763536
C -2.536733 -2.309784 -0.300777
H -2.120998 -2.780156 -1.188919
C 3.012722 1.845920 -0.850860
C 4.991617 3.192331 -1.068543
C 3.811826 2.955586 0.991601
C 4.872015 3.510205 0.283962
F -1.950828 0.248750 -2.413403
F -4.125294 1.817048 -2.201408
F -4.403950 3.469010 -0.071478
F -2.497399 3.540568 1.851310
F -0.324023 1.968756 1.653935
Ge 1.627661 0.690624 -1.657683
N 2.911918 2.145774 0.444967
H 5.807910 3.599822 -1.663519
H 5.582214 4.168755 0.778562
H 3.683623 3.175276 2.054184
C 4.054045 2.354158 -1.645949
H 4.110390 2.091380 -2.701060

^{F}YGePyr
E = -4147.00963070
P 0.039286 -1.271449 0.238527
C 0.178078 0.266213 -0.519362
C -1.017378 1.120628 -0.460497
C -1.192837 2.070792 0.548884
C -2.361288 2.812458 0.667767
C -3.399345 2.609780 -0.232997
C -3.262603 1.670862 -1.248836

C -2.085192 0.945219 -1.346004
C 0.845110 -1.389571 1.861784
C 0.546181 -2.444054 2.732033
H -0.173876 -3.207887 2.438860
C 1.158125 -2.507312 3.976310
H 0.925641 -3.325560 4.655080
C 2.062433 -1.517181 4.355851
H 2.539372 -1.566170 5.333132
C 2.345287 -0.462880 3.495760
H 3.041540 0.317305 3.797274
C 1.735455 -0.387799 2.246194
H 1.939639 0.455682 1.583852
C 0.693495 -2.607694 -0.799071
C 1.480302 -3.640905 -0.289401
H 1.754771 -3.653745 0.762405
C 1.935389 -4.646536 -1.135566
H 2.557849 -5.444858 -0.736682
C 1.605159 -4.626911 -2.485743
H 1.965608 -5.414434 -3.144791
C 0.823973 -3.594858 -2.998190
H 0.573974 -3.570873 -4.056745
C 0.374414 -2.581932 -2.162166
H -0.216787 -1.757255 -2.557818
C -1.714590 -1.592702 0.582474
C -2.294806 -0.924371 1.666543
H -1.663757 -0.378169 2.367056
C -3.673230 -0.936203 1.833632
H -4.121153 -0.405870 2.671496
C -4.477511 -1.619575 0.925633
H -5.558224 -1.623448 1.052969
C -3.901915 -2.299445 -0.142301
H -4.529962 -2.838111 -0.848674
C -2.522870 -2.284237 -0.319377
H -2.079278 -2.798487 -1.168848
C 3.087941 1.797078 -0.865352
C 5.004811 2.968021 -1.215898
C 3.994736 2.888321 0.914543
C 5.029346 3.367040 0.119128
F -2.000368 0.025004 -2.302029
F -4.257631 1.470074 -2.098791
F -4.515807 3.309852 -0.124980
F -2.504596 3.694151 1.645861
F -0.253040 2.241074 1.466694
Ge 1.659724 0.697828 -1.653455
N 3.035485 2.109454 0.435909
N 4.051365 2.196555 -1.709433
H 5.787384 3.287538 -1.908880
H 5.812441 4.007871 0.515627
H 3.939392 3.140772 1.976559

^{F}YGeB2
E = -4289.97532452
P -0.824888 1.268738 0.231724
C -0.403969 -0.135428 -0.668595
C -1.224216 -1.341583 -0.540738
C -2.082901 -1.784026 -1.551579
C -2.849448 -2.934375 -1.422134
C -2.786922 -3.673693 -0.247254
C -1.948118 -3.263169 0.781776
C -1.179945 -2.119611 0.621589

C -1.753512 2.540627 -0.684261
C -2.320608 3.622667 -0.002482
H -2.199970 3.711579 1.076977
C -3.047173 4.577101 -0.701207
H -3.486906 5.419607 -0.171097
C -3.219424 4.449344 -2.077893
H -3.792414 5.196574 -2.623699
C -2.672891 3.364153 -2.752719
H -2.819699 3.256374 -3.825332
C -1.941071 2.405444 -2.058360
H -1.528987 1.538753 -2.573046
C 0.666124 2.048200 0.899596
C 0.952550 3.408393 0.783034
H 0.258268 4.075805 0.278391
C 2.149800 3.904814 1.287518
H 2.379530 4.963350 1.184739
C 3.057711 3.050522 1.904002
H 3.998193 3.442704 2.286273
C 2.772959 1.692496 2.020787
H 3.489920 1.018452 2.485589
C 1.580788 1.188653 1.519102
H 1.365563 0.122481 1.566864
C -1.935011 0.847407 1.603267
C -3.238035 0.457754 1.275562
H -3.570370 0.492838 0.238401
C -4.104032 0.024658 2.270049
H -5.114721 -0.283738 2.010951
C -3.677317 -0.013353 3.594752
H -4.356290 -0.353505 4.374166
C -2.386069 0.383779 3.923456
H -2.053650 0.356885 4.959037
C -1.510813 0.809844 2.930593
H -0.497400 1.107524 3.189695
Ge 0.984160 0.060588 -1.988722
C 4.339648 -1.222748 0.165916
C 4.970393 -0.674097 -0.948771
C 5.053450 -1.760882 1.218971
C 6.346232 -0.639834 -1.063546
C 6.446350 -1.729035 1.112726
H 4.547422 -2.190506 2.079644
C 7.077870 -1.181222 -0.003552
H 6.825999 -0.209728 -1.938511
H 7.049960 -2.143406 1.917438
H 8.164707 -1.175451 -0.051658
B 2.797050 -0.472051 -1.211671
O 4.024875 -0.215978 -1.812141
O 2.993117 -1.115599 0.009252
F -0.378063 -1.765115 1.621563
F -2.195722 -1.081125 -2.673444
F -3.657242 -3.319212 -2.398228
F -1.882145 -3.970742 1.898836
F -3.520439 -4.766072 -0.110701

^{F}YGeB1
E = -4559.15481770
P 1.668853 1.398962 -0.023348
C 0.411927 0.273170 0.228850
C 0.604208 -1.052514 0.800159
C -0.218823 -1.560381 1.819973
C -0.116928 -2.860238 2.294451

C 0.854764 -3.712036 1.787064
C 1.701507 -3.248143 0.791114
C 1.559245 -1.958636 0.308433
C 1.557327 2.906851 1.004796
C 2.596987 3.842638 0.968602
H 3.450947 3.682952 0.310349
C 2.549317 4.967955 1.779283
H 3.359296 5.694075 1.747878
C 1.469826 5.159858 2.639220
H 1.434243 6.041214 3.276868
C 0.447281 4.220779 2.693661
H -0.389229 4.360783 3.375234
C 0.490848 3.090086 1.882030
H -0.294898 2.337360 1.935030
C 1.726850 1.970274 -1.749998
C 1.663637 3.307816 -2.138171
H 1.596096 4.094686 -1.391461
C 1.659976 3.637561 -3.490478
H 1.599214 4.682016 -3.789621
C 1.722920 2.638786 -4.454380
H 1.718070 2.901161 -5.510622
C 1.779088 1.301058 -4.069444
H 1.817186 0.516408 -4.822830
C 1.771150 0.965161 -2.723211
H 1.783149 -0.076503 -2.407016
C 3.302644 0.761669 0.431901
C 3.460906 0.293137 1.739408
H 2.626790 0.341502 2.438299
C 4.680330 -0.233573 2.142561
H 4.799347 -0.605035 3.158262
C 5.746707 -0.283823 1.249330
H 6.702293 -0.696381 1.566979
C 5.593058 0.194105 -0.047283
H 6.427107 0.157081 -0.745106
C 4.371273 0.713193 -0.461414
H 4.248471 1.071695 -1.481199
Ge -1.369066 0.873338 -0.224189
N -2.602682 -1.890447 -1.054493
C -4.784421 -1.594830 -0.623910
C -3.846455 -2.507035 -0.954649
H -5.854342 -1.736486 -0.528751
H -3.979507 -3.556116 -1.191219
B -2.774039 -0.494605 -0.722731
N -4.194900 -0.343925 -0.488120
C -4.896554 0.794758 -0.071203
C -5.964572 0.694621 0.825327
C -4.520895 2.056614 -0.541536
C -6.639721 1.835025 1.239136
H -6.245365 -0.279013 1.220107
C -5.191529 3.193680 -0.109331
H -3.720353 2.132644 -1.274762
C -6.255811 3.091126 0.779459
H -7.466136 1.740435 1.941235
H -4.885808 4.168096 -0.485902
H -6.784163 3.982566 1.110736
C -1.455869 -2.592794 -1.462860
C -0.555574 -2.008190 -2.357964
C -1.224492 -3.894077 -1.011061
C 0.547804 -2.725459 -2.797926
H -0.743780 -0.995644 -2.710688

C -0.115479 -4.602967 -1.456719
H -1.907733 -4.339350 -0.290718
C 0.777765 -4.023182 -2.350598
H 1.240436 -2.263529 -3.499648
H 0.064865 -5.607865 -1.079224
H 1.652751 -4.574229 -2.686941
F -1.150081 -0.784247 2.367218
F -0.930824 -3.286233 3.250104
F 0.953897 -4.959796 2.224806
F 2.596336 -4.066049 0.254717
F 2.355150 -1.604165 -0.701820

I
E = -3096.05423687
C 1.649788 2.336083 -0.103603
C -0.521167 1.172002 -0.192788
C -1.133065 2.427474 -0.427205
C -0.382738 3.582086 -0.463277
C 1.015242 3.541301 -0.282972
H 2.733575 2.319894 0.003437
H -2.205218 2.468869 -0.595604
H -0.873793 4.535343 -0.649826
H 1.592523 4.463371 -0.308886
N -1.164608 -0.005996 -0.121780
C -2.537936 -0.151737 0.026073
C -3.293756 0.663547 0.885757
C -3.188241 -1.209595 -0.628295
C -4.651894 0.441096 1.055830
H -2.796578 1.454175 1.442742
C -4.548069 -1.424395 -0.450719
H -2.608318 -1.848074 -1.292883
C -5.292029 -0.599056 0.385789
H -5.215417 1.080135 1.733897
H -5.031236 -2.246593 -0.975942
H -6.357598 -0.769715 0.524066
C 0.930052 1.106027 -0.063295
C 1.456187 -0.183693 0.086311
P 3.122866 -0.540964 0.236597
C 4.186949 -0.049065 -1.160389
H 4.145499 1.036273 -1.295645
H 5.225508 -0.359781 -0.997217
H 3.796731 -0.524012 -2.067036
C 3.917824 0.156746 1.717158
H 4.975638 -0.126544 1.764529
H 3.830923 1.247661 1.705684
H 3.391894 -0.222044 2.599577
C 3.310108 -2.331247 0.367788
H 4.358782 -2.607782 0.518251
H 2.699117 -2.688620 1.204426
H 2.932982 -2.793624 -0.552340
Ge 0.037835 -1.660248 -0.311584

II
E = -3645.24313744
C 0.589456 2.948431 0.099962
C 0.150026 1.636913 0.186042
C -0.914343 1.182728 -0.608203
C -1.532988 2.068474 -1.497762
C -1.079408 3.376240 -1.593872
C -0.021650 3.817948 -0.798096

H 1.399880 3.266185 0.753215
H -2.353005 1.709574 -2.116244
H -1.553673 4.059067 -2.295722
H 0.319550 4.848600 -0.871890
N -1.307164 -0.151348 -0.480857
S 0.865559 0.465446 1.334325
O 2.038458 1.160921 1.917530
O -0.230733 0.076446 2.217554
C 1.388962 -0.785369 0.315458
C -2.574305 -0.455568 -0.102500
C -3.396084 0.492869 0.558708
C -3.077841 -1.763649 -0.308954
C -4.656350 0.136022 0.991778
H -2.994862 1.478547 0.775607
C -4.343189 -2.100105 0.130116
H -2.453357 -2.481847 -0.835487
C -5.141865 -1.157002 0.777960
H -5.266144 0.863904 1.522897
H -4.721199 -3.105518 -0.043370
H -6.138999 -1.427791 1.118703
P 3.070634 -0.737881 -0.024616
C 3.711045 0.794473 -0.763703
H 4.777796 0.715386 -1.002354
H 3.136516 1.003403 -1.673236
H 3.545921 1.598037 -0.038973
C 3.449112 -2.044949 -1.215884
H 2.900471 -1.855926 -2.145867
H 4.525322 -2.067115 -1.418407
H 3.131139 -3.009744 -0.806725
C 4.104566 -1.056375 1.424691
H 3.861119 -0.288523 2.165375
H 3.843582 -2.040553 1.826831
H 5.168882 -1.022479 1.164904
Ge 0.116253 -1.562754 -1.010886

III
E = -4407.32392771
C -0.406397 1.698335 -0.570550
C 0.407133 1.698167 0.570661
C 0.785593 2.917238 1.135138
C 0.393030 4.123839 0.572851
C -0.391169 4.124017 -0.572709
C -0.784293 2.917598 -1.135008
H 1.416215 2.892558 2.020182
H 0.705022 5.060088 1.031412
H -0.702721 5.060413 -1.031268
H -1.414926 2.893228 -2.020049
S -1.006697 0.260622 -1.531362
S 1.006801 0.260226 1.531450
O -2.222004 0.795241 -2.178337
O 0.132718 -0.125303 -2.377198
O -0.132810 -0.125331 2.377200
O 2.222267 0.794421 2.178484
C -1.436143 -0.982168 -0.464418
C 1.435787 -0.982576 0.464359
P 2.887315 -0.758006 -0.432141
P -2.887682 -0.757275 0.432018
C 2.902062 -1.891381 -1.830025
H 3.822849 -1.752714 -2.407615
H 2.834963 -2.924358 -1.474919

H 2.020201 -1.657221 -2.438778
C 3.067867 0.902641 -1.131526
H 3.076371 1.628831 -0.311941
H 4.001927 0.973264 -1.700321
H 2.203910 1.079487 -1.782740
C 4.372666 -1.037784 0.562211
H 4.377225 -2.077304 0.906165
H 5.288734 -0.820767 0.001359
H 4.286586 -0.379221 1.433797
C -2.902778 -1.890811 1.829768
H -3.823623 -1.752040 2.407243
H -2.835820 -2.923756 1.474545
H -2.020968 -1.656878 2.438692
C -3.068049 0.903322 1.131566
H -3.076554 1.629577 0.312040
H -4.002077 0.973977 1.700409
H -2.204047 1.080036 1.782728
C -4.373016 -1.036670 -0.562466
H -5.289076 -0.819477 -0.001665
H -4.286744 -0.378116 -1.434034
H -4.377773 -2.076186 -0.906433
Ge -0.000344 -2.161018 0.000042

IV
E = -3615.34098822
C 0.822298 3.358010 0.511379
C 0.286155 2.086062 0.563048
C -0.990949 1.718847 0.111745
C -1.779823 2.757344 -0.407586
C -1.281719 4.052385 -0.452858
C 0.005232 4.362098 0.001407
H 1.831684 3.560156 0.863022
H -2.790131 2.556390 -0.757009
H -1.910065 4.850702 -0.844153
H 0.365449 5.387522 -0.041257
B -1.241534 0.195413 0.428086
S 1.150956 0.613843 0.985711
O 2.009432 0.651001 2.170984
O -0.210121 -0.216923 1.353725
C 1.733830 -0.032320 -0.412269
C -2.531175 -0.657904 0.366855
C -3.556261 -0.412079 -0.561321
C -2.709368 -1.721979 1.265537
C -4.707324 -1.187352 -0.587001
H -3.436434 0.384682 -1.294562
C -3.860056 -2.499027 1.245395
H -1.926018 -1.929215 1.993150
C -4.864826 -2.233684 0.319113
H -5.485758 -0.980484 -1.319683
H -3.977771 -3.315973 1.955830
H -5.767402 -2.842179 0.300105
P 2.845331 -1.315521 -0.138883
C 4.402062 -0.761219 0.597298
H 5.079267 -1.605861 0.766578
H 4.868282 -0.035504 -0.076894
H 4.164620 -0.266292 1.544317
C 3.258554 -2.078179 -1.719414
H 3.664714 -1.311641 -2.387364
H 3.995086 -2.875602 -1.571640
H 2.343948 -2.487404 -2.163198

C 2.207452 -2.624201 0.941196
H 1.984537 -2.167771 1.913067
H 1.270724 -2.992631 0.507517
H 2.920799 -3.446408 1.066460
Ge 0.153685 -0.305676 -1.724239

V
E = -3066.12322495
C 1.794585 2.265960 -0.145396
C -0.420822 1.236773 -0.145869
C -0.963162 2.528305 -0.306190
C -0.166913 3.660504 -0.349712
C 1.220322 3.522252 -0.258586
H 2.881709 2.196135 -0.110814
H -2.043145 2.634614 -0.397829
H -0.615863 4.646039 -0.456369
H 1.859814 4.403278 -0.292100
B -1.317464 -0.006738 -0.104221
C -2.839751 -0.216282 0.016799
C -3.632245 0.659808 0.783788
C -3.497534 -1.292076 -0.607584
C -5.004119 0.479026 0.906439
H -3.155690 1.487699 1.306467
C -4.868463 -1.472027 -0.494739
H -2.910764 -1.996490 -1.196941
C -5.629518 -0.584921 0.263247
H -5.589897 1.170400 1.510395
H -5.349327 -2.310346 -0.996455
H -6.704906 -0.726595 0.356723
C 1.011307 1.097592 -0.092894
C 1.535490 -0.252008 -0.019779
P 3.179387 -0.644617 0.186538
C 4.303716 -0.161192 -1.165297
H 4.255790 0.921610 -1.316904
H 5.337798 -0.454819 -0.950372
H 3.961107 -0.649626 -2.083675
C 3.935856 0.048131 1.689593
H 5.000853 -0.201807 1.755029
H 3.813445 1.135828 1.690219
H 3.402758 -0.355223 2.556768
C 3.382919 -2.435668 0.328493
H 4.439372 -2.685310 0.473109
H 2.797231 -2.799041 1.179237
H 3.014550 -2.915979 -0.584100
Ge 0.110365 -1.509452 -0.222914

5.2.3 H_2 activation transition states
Pathway A

$^{Tos}Y_2Ge$
E = -5872.32885309
S -2.239495 1.328848 0.436903
S 2.005727 -1.292497 0.276699
P -2.265541 -1.566437 0.108656
P 2.427508 1.580251 0.207837
O -1.316687 2.442412 0.131808
O -2.522764 1.024203 1.854784
O 0.989827 -2.312171 -0.019345
O 2.370255 -1.097410 1.700748
C -1.643484 -0.042789 -0.385049

C -4.075289 -1.459689 0.352993
C -4.586194 -1.259167 1.637935
H -3.907813 -1.202550 2.484723
C -5.954452 -1.102887 1.828129
H -6.341725 -0.950384 2.833636
C -6.820467 -1.128982 0.741746
H -7.890994 -1.003084 0.893215
C -6.314564 -1.305204 -0.542354
H -6.985227 -1.316959 -1.399183
C -4.949646 -1.469953 -0.736534
H -4.570970 -1.613552 -1.744217
C -1.955463 -2.767175 -1.211690
C -2.264411 -2.407244 -2.528344
H -2.648819 -1.409364 -2.736083
C -2.034818 -3.298261 -3.566974
H -2.278696 -3.013386 -4.588559
C -1.471775 -4.543849 -3.301779
H -1.282056 -5.239571 -4.117088
C -1.129246 -4.887863 -2.000075
H -0.657264 -5.846321 -1.794498
C -1.366626 -4.002861 -0.953844
H -1.052090 -4.255297 0.054689
C -1.684798 -2.272900 1.672460
C -2.264042 -3.423547 2.216931
H -3.092085 -3.916289 1.707811
C -1.783394 -3.931236 3.416660
H -2.230720 -4.827062 3.843252
C -0.729116 -3.295190 4.071458
H -0.351630 -3.702431 5.007936
C -0.159149 -2.149008 3.531701
H 0.679204 -1.652417 4.014748
C -0.646773 -1.635874 2.337279
H -0.202048 -0.751500 1.898575
C -3.812960 1.784414 -0.277398
C -3.925277 1.902451 -1.659567
H -3.054865 1.718937 -2.288196
C -5.150630 2.237353 -2.212270
H -5.245155 2.326345 -3.294104
C -6.271634 2.464710 -1.403448
C -6.123026 2.358822 -0.021460
H -6.983209 2.534569 0.623519
C -4.900310 2.018255 0.548231
H -4.778084 1.911504 1.623279
C -7.600710 2.788165 -2.017408
H -8.301498 3.180962 -1.273325
H -8.056922 1.892160 -2.460814
H -7.503005 3.529092 -2.819768
C 1.597181 0.209110 -0.438990
C 1.941048 3.023164 -0.769130
C 1.446990 4.171431 -0.153254
H 1.344031 4.209353 0.927917
C 1.053640 5.257144 -0.927586
H 0.654821 6.145376 -0.442648
C 1.153706 5.201886 -2.311914
H 0.841275 6.053030 -2.914093
C 1.637914 4.053206 -2.931715
H 1.701285 4.000799 -4.016584
C 2.020817 2.962146 -2.165262
H 2.368504 2.049173 -2.645283
C 4.240583 1.417998 0.107665

C 4.966359 1.989945 -0.939617
H 4.479224 2.658354 -1.644484
C 6.320619 1.712213 -1.084761
H 6.877569 2.164416 -1.903089
C 6.959289 0.861601 -0.189174
H 8.017325 0.635841 -0.310981
C 6.244872 0.305291 0.866330
H 6.738654 -0.360810 1.570880
C 4.893411 0.585143 1.023769
H 4.327453 0.128547 1.832669
C 2.074374 1.984495 1.938853
C 2.985015 2.648297 2.762834
H 3.977301 2.902547 2.394775
C 2.618007 2.978014 4.063324
H 3.328870 3.487681 4.710997
C 1.347580 2.658715 4.536435
H 1.068018 2.922553 5.554988
C 0.436582 2.008684 3.709757
H -0.568092 1.763835 4.048414
C 0.805860 1.669007 2.416409
H 0.096841 1.172281 1.765923
C 3.535595 -1.862272 -0.466240
C 3.995800 -1.377557 -1.682248
H 3.397679 -0.647933 -2.224765
C 5.231061 -1.795272 -2.158598
H 5.602091 -1.397948 -3.102747
C 6.014512 -2.701180 -1.439590
C 5.514598 -3.196302 -0.232333
H 6.108120 -3.909226 0.339089
C 4.286773 -2.778361 0.261069
H 3.914963 -3.129389 1.221158
C 7.373813 -3.094331 -1.934346
H 7.731579 -4.008090 -1.448247
H 8.105879 -2.300975 -1.726590
H 7.376431 -3.258410 -3.018192
Ge -0.067547 0.440258 -1.390527
H -0.014554 -0.226894 -2.809041
H 0.101186 -1.157127 -2.035812

^{Tos}YGeCl
E = -4436.32799327
S 0.684486 1.643520 -1.159647
P -0.739595 -0.661573 0.096995
O 0.559760 3.106874 -1.016885
O 0.529765 1.021973 -2.479027
C -0.419442 1.016842 -0.006083
C -1.017061 -1.100871 1.838041
C -1.973072 -2.034591 2.236399
H -2.610465 -2.516745 1.499760
C -2.134409 -2.328770 3.586194
H -2.889893 -3.049334 3.892584
C -1.343713 -1.698889 4.540196
H -1.476281 -1.929242 5.595462
C -0.389172 -0.765155 4.145779
H 0.226001 -0.263859 4.890267
C -0.227889 -0.461688 2.800843
H 0.499712 0.283977 2.482671
C 0.693318 -1.629348 -0.460845
C 1.701662 -1.994316 0.434591
H 1.613515 -1.752439 1.490721

C 2.822360 -2.674358 -0.023182
H 3.605779 -2.954533 0.677764
C 2.940490 -2.994531 -1.371796
H 3.818291 -3.530465 -1.728079
C 1.942213 -2.624433 -2.266367
H 2.037739 -2.863739 -3.323404
C 0.820517 -1.938188 -1.817710
H 0.054388 -1.621969 -2.521163
C -2.134855 -1.284665 -0.873721
C -2.319292 -2.658810 -1.064644
H -1.616472 -3.371396 -0.633898
C -3.389331 -3.112382 -1.823742
H -3.532034 -4.180365 -1.975934
C -4.270841 -2.198771 -2.397771
H -5.107141 -2.556240 -2.995669
C -4.077058 -0.834115 -2.220307
H -4.756423 -0.118162 -2.677767
C -3.006418 -0.370923 -1.462267
H -2.843281 0.697573 -1.337822
C 2.330292 1.212625 -0.612009
C 2.727410 1.519476 0.686583
H 2.040710 2.041289 1.351501
C 3.992855 1.150910 1.112067
H 4.306756 1.384298 2.128942
C 4.880642 0.489319 0.252692
C 4.463370 0.218841 -1.049182
H 5.141456 -0.290879 -1.732461
C 3.191850 0.574665 -1.488166
H 2.847657 0.350658 -2.494925
C 6.241196 0.083170 0.733723
H 6.795423 0.942272 1.131894
H 6.836100 -0.358506 -0.072147
H 6.170787 -0.655151 1.543549
Ge -1.402084 2.466077 0.745158
H -2.383905 1.217515 1.552119
H -1.925092 2.260304 2.195371
Cl -3.107907 3.126523 -0.487858

^{Tos}YGeHMDS
E = -4850.11913580
S 1.160392 -1.967524 0.032740
P 0.630558 0.897505 0.311690
O 0.707714 -3.172273 -0.667037
O 1.193280 -1.986462 1.512408
C 0.310622 -0.572784 -0.532887
C -0.033159 2.274438 -0.665993
C -0.709194 3.339845 -0.072390
H -0.916208 3.330049 0.994774
C -1.127592 4.416726 -0.848986
H -1.663381 5.240145 -0.381811
C -0.866654 4.436880 -2.213480
H -1.196461 5.279354 -2.818116
C -0.194988 3.372810 -2.811141
H -0.001977 3.379255 -3.881640
C 0.216945 2.292306 -2.043940
H 0.726218 1.447672 -2.504646
C 2.394328 1.283966 0.551414
C 3.062777 2.192481 -0.271402
H 2.521771 2.747418 -1.033313
C 4.429390 2.394053 -0.118273

H 4.943792 3.106097 -0.760477
C 5.134831 1.685141 0.847576
H 6.207519 1.834515 0.958112
C 4.468077 0.789235 1.676999
H 5.017000 0.230393 2.431929
C 3.099943 0.594340 1.542592
H 2.579607 -0.123729 2.174428
C -0.061964 1.038115 1.984399
C 0.387914 2.024419 2.868169
H 1.179610 2.708986 2.566134
C -0.168423 2.121950 4.136642
H 0.180406 2.889713 4.824362
C -1.161507 1.227920 4.529870
H -1.589497 1.298331 5.528097
C -1.598382 0.239629 3.655811
H -2.362022 -0.468071 3.970596
C -1.051909 0.142509 2.381140
H -1.380660 -0.627178 1.688340
C 2.879449 -1.782113 -0.439055
C 3.277976 -1.030637 -1.534506
H 2.528302 -0.509414 -2.126723
C 4.630406 -0.920687 -1.827717
H 4.946279 -0.309823 -2.672649
C 5.594287 -1.568538 -1.051329
C 5.162272 -2.352356 0.021322
H 5.897575 -2.875788 0.631361
C 3.815084 -2.456718 0.336720
H 3.477851 -3.032907 1.195318
C 7.053834 -1.386539 -1.338200
H 7.654942 -2.189859 -0.899372
H 7.413652 -0.436981 -0.916284
H 7.253257 -1.356807 -2.415460
Ge -1.340371 -0.640138 -1.549317
H -1.266411 -1.627801 -2.757882
H -1.094271 -2.338131 -1.772912
N -2.923176 -0.812752 -0.563637
Si -3.402958 -2.314719 0.235898
Si -4.064430 0.521606 -0.715193
C -4.129414 1.524946 0.873270
H -3.143612 1.958841 1.082984
H -4.850841 2.348460 0.786219
H -4.410884 0.916966 1.740422
C -5.788829 -0.089094 -1.162134
H -6.219538 -0.791607 -0.440814
H -6.471498 0.767676 -1.239970
H -5.765329 -0.587617 -2.139905
C -3.565526 1.683775 -2.101199
H -2.594409 2.159094 -1.930102
H -3.517420 1.163892 -3.065992
H -4.317818 2.481169 -2.178312
C -4.335193 -3.395455 -0.986027
H -4.627549 -4.348968 -0.526722
H -5.239431 -2.905255 -1.364751
H -3.689516 -3.624826 -1.844294
C -1.984728 -3.321108 0.913702
H -1.421955 -3.833026 0.127554
H -1.250081 -2.756859 1.499288
H -2.419547 -4.084804 1.574574
C -4.514745 -1.920352 1.709686
H -5.407882 -1.334116 1.469474

H -4.848817 -2.863723 2.161618
H -3.951398 -1.370415 2.474221

$^{Tos}YGeC_6F_5$
E = -4704.66376435
S 1.721747 -1.673776 -1.156908
P 0.380932 0.321261 0.594419
O 1.560053 -2.179255 -2.524443
O 1.670743 -2.640135 -0.042257
C 0.602297 -0.397137 -0.944962
C -0.551823 1.855400 0.330304
C -1.678012 2.200618 1.079157
H -2.067193 1.517111 1.829522
C -2.323024 3.409918 0.843691
H -3.214111 3.663617 1.413454
C -1.837464 4.285555 -0.121120
H -2.343771 5.232192 -0.298559
C -0.720923 3.942372 -0.876308
H -0.351768 4.615978 -1.646521
C -0.086068 2.726185 -0.662572
H 0.774365 2.438502 -1.265381
C 1.938114 0.775778 1.412730
C 2.396850 2.093294 1.459362
H 1.782352 2.905156 1.079065
C 3.647791 2.372795 1.997206
H 3.999542 3.401791 2.035657
C 4.446267 1.341577 2.478880
H 5.431167 1.560962 2.887468
C 3.984597 0.029671 2.444231
H 4.608552 -0.779961 2.816495
C 2.728804 -0.257586 1.926204
H 2.377806 -1.287668 1.874954
C -0.498098 -0.660079 1.840791
C -0.680299 -0.178144 3.141644
H -0.254201 0.781092 3.435345
C -1.405001 -0.928255 4.057296
H -1.554537 -0.554487 5.068282
C -1.938278 -2.160333 3.681045
H -2.511929 -2.742779 4.399571
C -1.724254 -2.655246 2.400178
H -2.124359 -3.623688 2.108366
C -0.992919 -1.910101 1.480317
H -0.774154 -2.303699 0.492348
C 3.370032 -0.979098 -1.046211
C 3.633160 0.343871 -1.372943
H 2.819561 0.982357 -1.711608
C 4.918871 0.841764 -1.216537
H 5.119942 1.886871 -1.449030
C 5.957490 0.028885 -0.754347
C 5.674296 -1.308922 -0.470762
H 6.472884 -1.961455 -0.119757
C 4.388505 -1.815194 -0.604848
H 4.155378 -2.844474 -0.342352
C 7.326419 0.595487 -0.529117
H 7.609703 1.294313 -1.324535
H 8.085673 -0.191792 -0.476924
H 7.362046 1.152625 0.418066
Ge -0.752314 -0.191367 -2.333036
H -0.626644 -1.050479 -3.614852
H -0.879210 -1.869280 -2.686562

C -2.542867 -0.362308 -1.490472
C -3.041747 -1.515524 -0.891763
C -3.288114 0.797326 -1.316037
C -4.171233 -1.499946 -0.082948
C -4.424359 0.849224 -0.520426
C -4.866708 -0.311496 0.100381
F -2.889843 1.933923 -1.875072
F -2.439605 -2.688330 -1.034286
F -5.073952 1.989366 -0.336570
F -5.940729 -0.284072 0.867015
F -4.587552 -2.604722 0.515605

^{Tos}*YGePy*
E = -4223.82047984
S 0.343639 -1.847777 0.101220
P 0.071375 1.102514 0.164299
O -0.323071 -2.929685 -0.653084
O 0.228999 -1.880234 1.574362
C -0.256098 -0.406585 -0.568326
C -0.351513 2.400275 -1.028125
C -0.972490 3.588836 -0.643986
H -1.243754 3.753326 0.396036
C -1.267784 4.555618 -1.598684
H -1.760880 5.477189 -1.296431
C -0.944676 4.342450 -2.934236
H -1.182664 5.099361 -3.679028
C -0.325592 3.157040 -3.320464
H -0.080379 2.983192 -4.366126
C -0.029750 2.185762 -2.372973
H 0.432882 1.245136 -2.668399
C 1.834817 1.306705 0.548951
C 2.679321 2.059579 -0.268955
H 2.272614 2.627736 -1.102199
C 4.046828 2.080658 -0.021039
H 4.701195 2.671607 -0.658748
C 4.575649 1.343977 1.033141
H 5.648568 1.350537 1.217649
C 3.734093 0.599891 1.854016
H 4.145957 0.017743 2.675581
C 2.364448 0.587045 1.624971
H 1.707085 -0.015530 2.250320
C -0.778489 1.470508 1.723555
C -0.345162 2.511303 2.550169
H 0.520420 3.109757 2.267462
C -1.011197 2.768401 3.741381
H -0.674089 3.575623 4.388635
C -2.102200 1.984831 4.110209
H -2.619924 2.184705 5.046569
C -2.522747 0.940520 3.293826
H -3.365899 0.318457 3.586776
C -1.859311 0.677565 2.101103
H -2.159697 -0.152625 1.467679
C 2.101635 -1.974653 -0.198910
C 2.699884 -1.344511 -1.281729
H 2.084293 -0.774378 -1.974686
C 4.077082 -1.413834 -1.432292
H 4.550308 -0.900090 -2.268207
C 4.869502 -2.117567 -0.520810
C 4.238079 -2.771361 0.539435
H 4.838181 -3.332959 1.254403

C 2.862387 -2.698052 0.711262
H 2.370683 -3.167762 1.559739
C 6.361610 -2.128969 -0.659574
H 6.788420 -1.177929 -0.310345
H 6.668435 -2.254995 -1.704297
H 6.815851 -2.931292 -0.068962
Ge -1.805012 -0.861509 -1.672643
H -2.492857 0.715041 -1.348092
H -2.303275 0.376648 -2.510648
C -3.375259 -1.527511 -0.677599
C -5.647816 -1.768831 -0.705028
C -4.425311 -2.737864 1.099982
C -5.646871 -2.489063 0.486074
H -6.587357 -1.562388 -1.222651
H -4.373594 -3.308255 2.026361
H -6.580498 -2.850940 0.911434
N -4.543716 -1.302965 -1.275170
C -3.263335 -2.255462 0.509951
H -2.288336 -2.442009 0.954932

^{Tos}YGePyr
E = -4239.88106424
S 0.416757 -1.615469 0.908552
P 0.017041 1.112479 -0.092049
O -0.238684 -2.915670 0.667312
O 0.418275 -1.030831 2.258575
C -0.259099 -0.561319 -0.245698
C -0.204329 1.852676 -1.736577
C -0.888869 3.049283 -1.948847
H -1.330511 3.585166 -1.113207
C -1.037458 3.545698 -3.239631
H -1.580920 4.474879 -3.398380
C -0.506480 2.854419 -4.322322
H -0.628706 3.244746 -5.330786
C 0.168769 1.654761 -4.116613
H 0.572618 1.101883 -4.962269
C 0.314937 1.150837 -2.831310
H 0.809904 0.194670 -2.664484
C 1.720414 1.461783 0.446156
C 2.771202 1.463859 -0.474385
H 2.572813 1.328711 -1.534609
C 4.077045 1.644677 -0.038935
H 4.890651 1.646823 -0.761307
C 4.341663 1.820836 1.315878
H 5.365870 1.963341 1.655539
C 3.299820 1.803934 2.235923
H 3.504951 1.926421 3.297460
C 1.990469 1.619677 1.807579
H 1.179649 1.573747 2.530082
C -1.013063 2.011968 1.098279
C -0.797293 3.367432 1.371985
H -0.009507 3.913410 0.853334
C -1.572968 4.010841 2.325726
H -1.407823 5.064928 2.540001
C -2.553432 3.300309 3.016457
H -3.158441 3.804952 3.767644
C -2.747016 1.948182 2.761560
H -3.498527 1.389718 3.316208
C -1.974112 1.294035 1.806209
H -2.097576 0.226270 1.623270

C 2.143104 -1.841161 0.492612
C 2.494145 -2.177985 -0.811910
H 1.715641 -2.313514 -1.561528
C 3.832297 -2.322233 -1.138735
H 4.110896 -2.580391 -2.160004
C 4.834997 -2.145624 -0.176062
C 4.452667 -1.830387 1.126601
H 5.218307 -1.692188 1.889152
C 3.112287 -1.674872 1.467759
H 2.803742 -1.406895 2.475390
C 6.280472 -2.279993 -0.550994
H 6.928002 -2.250220 0.331358
H 6.591778 -1.467320 -1.221333
H 6.470419 -3.221776 -1.080363
Ge -1.594493 -1.522286 -1.254782
H -2.290702 -0.033995 -1.853741
H -1.900165 -0.858922 -2.655459
C -3.335399 -1.706504 -0.346574
C -5.527837 -2.266620 -0.499101
C -4.512412 -1.852022 1.588311
C -5.658149 -2.195518 0.884176
H -6.381234 -2.524989 -1.130583
H -4.521607 -1.772448 2.677847
H -6.601404 -2.400022 1.384222
N -3.358136 -1.603848 0.980266
N -4.376582 -2.035769 -1.112941

^{Tos}YGeB2
Not determined.

^{Tos}YGeB1
E = -4652.02225209
S -1.630963 1.386269 -0.200397
P -0.275309 -1.193388 0.211468
O -1.586056 2.566142 -1.070285
O -1.521993 1.606139 1.261602
C -0.513088 0.201749 -0.758041
C 0.644311 -2.395386 -0.792727
C 1.652288 -3.206510 -0.276500
H 1.997774 -3.076493 0.744703
C 2.242814 -4.175422 -1.082341
H 3.035339 -4.797749 -0.673532
C 1.839739 -4.332392 -2.401558
H 2.307000 -5.090194 -3.027709
C 0.845526 -3.510636 -2.926897
H 0.536768 -3.617974 -3.964619
C 0.250158 -2.545632 -2.128610
H -0.518357 -1.887863 -2.530823
C -1.806434 -2.042221 0.710384
C -2.266441 -3.173134 0.033595
H -1.669051 -3.621923 -0.755983
C -3.495268 -3.730203 0.367152
H -3.847213 -4.614373 -0.160681
C -4.272559 -3.156051 1.366704
H -5.240620 -3.585727 1.618767
C -3.811836 -2.033492 2.047653
H -4.420699 -1.577942 2.825735
C -2.576903 -1.481199 1.733208
H -2.222868 -0.587999 2.246339
C 0.603486 -0.970519 1.791596

C 0.755903 -2.039938 2.681627
H 0.335694 -3.015480 2.438503
C 1.427884 -1.855503 3.881866
H 1.552339 -2.690696 4.568397
C 1.922239 -0.595317 4.213993
H 2.437676 -0.448149 5.161503
C 1.738606 0.476204 3.348480
H 2.104618 1.466404 3.609593
C 1.083087 0.291894 2.134495
H 0.907766 1.131966 1.467700
C -3.277102 0.703077 -0.398999
C -3.578637 -0.243607 -1.367535
H -2.790480 -0.600945 -2.027284
C -4.868665 -0.750004 -1.445558
H -5.099715 -1.512162 -2.189011
C -5.873086 -0.309484 -0.579774
C -5.552059 0.672424 0.360070
H -6.323903 1.036864 1.037195
C -4.262000 1.174258 0.460985
H -3.996421 1.908137 1.218295
C -7.244636 -0.911276 -0.633547
H -7.985809 -0.271121 -0.143464
H -7.259499 -1.884809 -0.122678
H -7.569373 -1.084623 -1.666001
Ge 1.045835 0.711222 -1.852915
H 0.495838 1.512637 -3.094195
H 0.364529 2.236224 -2.109372
N 3.461267 1.110433 0.026332
C 3.135940 3.058847 1.074454
C 3.890530 1.937741 1.056695
H 3.228621 3.933198 1.708570
H 4.745878 1.683478 1.671318
N 2.193215 2.997397 0.059507
C 4.004260 -0.176966 -0.149423
C 4.272424 -0.666759 -1.430165
C 4.316909 -0.964608 0.962459
C 4.867521 -1.910749 -1.589722
H 4.033181 -0.051389 -2.293298
C 4.928403 -2.201341 0.793865
H 4.070176 -0.603673 1.958626
C 5.214008 -2.677059 -0.480936
H 5.077051 -2.276319 -2.593109
H 5.177410 -2.797471 1.670117
H 5.698708 -3.642700 -0.611455
C 1.174283 3.969830 -0.070803
C 0.988862 4.627188 -1.284573
C 0.332311 4.239704 1.004838
C -0.059434 5.525611 -1.429583
H 1.669964 4.420760 -2.107252
C -0.706563 5.148176 0.856219
H 0.461146 3.693876 1.936859
C -0.909666 5.786644 -0.361082
H -0.212595 6.025285 -2.384112
H -1.379235 5.333301 1.691039
H -1.736753 6.483462 -0.480596
B 2.346754 1.750578 -0.638729

$^{Ph}Y_2Ge$
E = -4695.13169882
P 3.016675 0.229248 0.090020

P -2.937554 0.418236 -0.018796
C 1.452754 -0.329334 -0.254399
C 1.242587 -1.663878 -0.834594
C 2.060289 -2.772031 -0.553417
H 2.884765 -2.658081 0.147340
C 1.815317 -4.019694 -1.113675
H 2.469192 -4.854992 -0.866902
C 0.730566 -4.211554 -1.961685
H 0.527301 -5.192180 -2.387102
C -0.089316 -3.126642 -2.260609
H -0.935984 -3.251121 -2.934942
C 0.169934 -1.875975 -1.719187
H -0.466190 -1.030332 -1.972758
C 3.799131 -0.344169 1.651204
C 4.663335 -1.441734 1.701484
H 5.025527 -1.892090 0.778622
C 5.064207 -1.966565 2.924718
H 5.741238 -2.818384 2.949721
C 4.602272 -1.407277 4.111628
H 4.915265 -1.821200 5.068188
C 3.733942 -0.320906 4.071925
H 3.364840 0.118164 4.996689
C 3.330631 0.203951 2.850781
H 2.654962 1.058773 2.823321
C 3.066448 2.046818 0.222731
C 4.078950 2.687192 0.939188
H 4.791799 2.102558 1.517825
C 4.175817 4.074295 0.923513
H 4.965070 4.567076 1.487883
C 3.266172 4.826628 0.189121
H 3.340736 5.912535 0.178760
C 2.263559 4.189709 -0.536088
H 1.550870 4.775000 -1.112594
C 2.165312 2.804770 -0.526319
H 1.390411 2.293884 -1.094405
C 4.217091 -0.160684 -1.222453
C 3.763158 -0.548563 -2.483851
H 2.698394 -0.688955 -2.651276
C 4.673012 -0.753988 -3.514476
H 4.314494 -1.067904 -4.492721
C 6.033135 -0.558961 -3.298774
H 6.742136 -0.720111 -4.108791
C 6.487241 -0.150478 -2.048558
H 7.549353 0.014837 -1.878681
C 5.582412 0.050220 -1.013491
H 5.939518 0.374127 -0.036747
C -1.665484 -0.487037 0.668272
C -1.908344 -1.873682 1.080234
C -0.857767 -2.727110 1.472398
H 0.153743 -2.333011 1.497645
C -1.075425 -4.051478 1.816774
H -0.221854 -4.669338 2.092539
C -2.358158 -4.593549 1.811309
H -2.527164 -5.633148 2.084532
C -3.418138 -3.770536 1.451247
H -4.436015 -4.158589 1.444875
C -3.200165 -2.445638 1.095082
H -4.067103 -1.849542 0.814035
C -4.357818 0.686021 1.111942
C -4.049678 0.691902 2.474427

H -3.021167 0.497680 2.778835
C -5.046858 0.916375 3.416006
H -4.800103 0.915197 4.475878
C -6.358759 1.124706 3.003581
H -7.141697 1.294208 3.740478
C -6.673513 1.100256 1.648378
H -7.701937 1.248083 1.324283
C -5.678359 0.879144 0.702768
H -5.940318 0.841655 -0.351380
C -2.374663 2.075606 -0.497360
C -1.616604 2.205577 -1.666110
H -1.397125 1.325340 -2.269558
C -1.149240 3.453507 -2.054553
H -0.558707 3.550822 -2.963649
C -1.437995 4.575606 -1.281623
H -1.076473 5.555311 -1.589298
C -2.192173 4.448472 -0.121122
H -2.416805 5.324999 0.483053
C -2.665011 3.200559 0.271476
H -3.255051 3.100610 1.179880
C -3.642496 -0.220638 -1.583628
C -4.414684 0.602767 -2.410265
H -4.631140 1.627226 -2.109254
C -4.879324 0.132086 -3.632549
H -5.475117 0.781100 -4.271547
C -4.569457 -1.161111 -4.042772
H -4.931101 -1.529050 -5.001173
C -3.783970 -1.975829 -3.234572
H -3.526746 -2.983057 -3.557377
C -3.314358 -1.508358 -2.011748
H -2.677645 -2.136030 -1.393703
Ge 0.009377 0.459562 0.784008
H 0.369799 0.865036 2.264834
H 0.372048 -0.327968 2.364173

^{Ph}YGeCl
E = -3847.73579749
P -0.581232 0.279148 0.009854
C 0.949225 -0.393483 -0.372970
C 2.171474 0.396789 -0.187983
C 2.145157 1.720224 0.304792
H 1.203093 2.180351 0.596545
C 3.299728 2.478807 0.439689
H 3.221756 3.497666 0.816314
C 4.540479 1.948880 0.105966
H 5.446627 2.540892 0.215935
C 4.596700 0.637586 -0.355016
H 5.556751 0.188593 -0.605275
C 3.444585 -0.122360 -0.494752
H 3.528248 -1.152826 -0.829977
C -0.896846 0.612969 1.776684
C -2.175916 0.915849 2.255982
H -3.017425 0.980569 1.567057
C -2.384181 1.099222 3.617128
H -3.381624 1.329337 3.986482
C -1.320329 0.973819 4.506610
H -1.485400 1.115960 5.572958
C -0.053072 0.647809 4.036621
H 0.775221 0.529832 4.732052
C 0.159754 0.458083 2.675287

H 1.141803 0.173049 2.302257
C -1.922740 -0.842013 -0.463350
C -2.313817 -1.849863 0.422672
H -1.858555 -1.914234 1.409406
C -3.277881 -2.773064 0.039352
H -3.574145 -3.561808 0.727492
C -3.857094 -2.690988 -1.222945
H -4.611872 -3.415698 -1.522085
C -3.475928 -1.683165 -2.102531
H -3.932061 -1.616382 -3.088035
C -2.509309 -0.757806 -1.726355
H -2.208259 0.028860 -2.415239
C -0.863325 1.837430 -0.901685
C -1.665149 2.879404 -0.433678
H -2.138359 2.816867 0.542347
C -1.843911 4.019820 -1.208519
H -2.468293 4.830269 -0.837791
C -1.220530 4.129088 -2.447174
H -1.358897 5.025715 -3.048331
C -0.408420 3.098851 -2.909743
H 0.096576 3.188800 -3.869318
C -0.225315 1.957419 -2.139212
H 0.430426 1.154925 -2.474749
Ge 0.907920 -2.243572 -0.809268
H 2.095434 -2.065922 -2.106486
H 1.039047 -2.750616 -2.274034
Cl 2.341465 -3.393703 0.415412

^{Ph}YGeHMDS
E = -4261.53209731
P -1.752798 -0.149086 -0.104955
C -0.184920 0.499875 -0.304144
C 0.106966 1.868718 0.118941
C -0.753749 2.614825 0.954504
H -1.680976 2.173828 1.316995
C -0.460404 3.915897 1.340188
H -1.162224 4.448342 1.980594
C 0.714424 4.534098 0.926511
H 0.949053 5.549704 1.237872
C 1.583054 3.819468 0.106914
H 2.511258 4.276564 -0.234839
C 1.287438 2.523395 -0.287782
H 1.981755 1.990183 -0.929469
C -2.267549 -0.639804 1.581810
C -3.382002 -1.453746 1.811256
H -3.978602 -1.811102 0.972602
C -3.715253 -1.831219 3.106308
H -4.579957 -2.469017 3.279158
C -2.937164 -1.403042 4.178608
H -3.198546 -1.701754 5.192036
C -1.818085 -0.608832 3.954157
H -1.197868 -0.286442 4.787951
C -1.479022 -0.233675 2.658999
H -0.584725 0.357368 2.470218
C -1.950681 -1.663625 -1.082895
C -1.637941 -2.907995 -0.531359
H -1.348883 -2.985139 0.514607
C -1.683416 -4.048714 -1.323710
H -1.429844 -5.014847 -0.892412
C -2.044773 -3.953493 -2.663060

H -2.078312 -4.848029 -3.282042
C -2.366476 -2.716037 -3.212113
H -2.654498 -2.640510 -4.258624
C -2.319894 -1.572035 -2.425799
H -2.566629 -0.603780 -2.856777
C -3.024755 1.021127 -0.700140
C -4.322091 1.080486 -0.190126
H -4.619679 0.439819 0.635609
C -5.239391 1.978279 -0.724812
H -6.249438 2.020569 -0.322011
C -4.865836 2.824336 -1.763500
H -5.585049 3.529212 -2.176368
C -3.568753 2.778844 -2.264412
H -3.266998 3.452000 -3.064244
C -2.648131 1.884231 -1.733507
H -1.622116 1.856777 -2.098241
Ge 1.128550 -0.672558 -1.081168
H 1.455752 0.478551 -2.413443
H 1.248984 -0.705726 -2.638920
Si 2.743753 -0.912895 1.465695
Si 4.276400 0.148983 -0.954868
C 1.408418 -2.216476 1.722510
H 0.402826 -1.814728 1.557918
H 1.554927 -3.067755 1.044570
H 1.448802 -2.589528 2.754656
C 4.352419 -1.706864 2.034074
H 5.225453 -1.052046 1.933303
H 4.256494 -1.962791 3.098011
H 4.554755 -2.633252 1.482607
C 2.399005 0.558690 2.580035
H 1.508120 1.107488 2.253971
H 2.254533 0.236652 3.620550
H 3.234448 1.269384 2.560600
C 4.035332 0.945219 -2.640808
H 3.667606 0.236546 -3.392771
H 3.361726 1.809565 -2.633561
H 5.024299 1.292542 -2.972360
C 5.517764 -1.236353 -1.242668
H 6.404265 -0.850942 -1.764326
H 5.855594 -1.710394 -0.315382
H 5.070003 -2.016022 -1.872849
C 5.031897 1.460160 0.165807
H 5.332388 1.062929 1.142634
H 5.923596 1.898927 -0.300848
H 4.305001 2.263583 0.344030
N 2.795768 -0.461179 -0.234228

$^{Ph}YGeC_6F_5$
E = -4116.08051812
P -1.775542 -0.124040 0.002878
C -0.400925 0.699349 -0.606457
C -0.016257 2.000541 -0.046769
C 1.093427 2.726657 -0.526029
H 1.696555 2.306858 -1.327065
C 1.440151 3.966806 -0.008846
H 2.307084 4.483381 -0.418450
C 0.701990 4.549387 1.015849
H 0.976824 5.521426 1.419622
C -0.390811 3.851943 1.517674
H -0.985836 4.274088 2.326262

C -0.736523 2.609186 1.006617
H -1.589942 2.102152 1.452423
C -3.275607 0.914139 -0.087221
C -3.316305 1.862596 -1.112735
H -2.452042 1.968188 -1.767394
C -4.435550 2.670513 -1.270573
H -4.458636 3.411339 -2.067200
C -5.515136 2.542719 -0.402724
H -6.389584 3.179345 -0.523363
C -5.470908 1.611016 0.629257
H -6.308211 1.518822 1.318145
C -4.353807 0.799417 0.791334
H -4.321509 0.091528 1.615046
C -2.124467 -1.613931 -0.966181
C -1.459893 -2.803204 -0.652207
C -1.656649 -3.932612 -1.435912
C -2.515620 -3.880879 -2.529308
C -3.180394 -2.699301 -2.840456
C -2.986947 -1.564590 -2.061232
C -1.644450 -0.747392 1.715466
C -2.559731 -1.664877 2.242624
H -3.398454 -2.009374 1.638646
C -2.381882 -2.168136 3.525199
H -3.093352 -2.885764 3.929044
C -1.286150 -1.766237 4.284563
H -1.145767 -2.164772 5.287555
C -0.361454 -0.873092 3.755654
H 0.507347 -0.574830 4.338569
C -0.534069 -0.369543 2.471332
H 0.201324 0.305393 2.038359
Ge 0.738202 -0.371983 -1.698777
H 0.841060 -0.139119 -3.241792
H 1.221978 0.913632 -2.769132
C 2.544538 -0.375094 -0.895797
C 3.833340 -1.133728 1.031735
C 4.863249 0.298084 -0.604304
C 4.943509 -0.434190 0.573705
H -0.781216 -2.837998 0.198107
H -1.132866 -4.854979 -1.194228
H -2.668636 -4.767227 -3.142058
H -3.854174 -2.659553 -3.693833
H -3.506083 -0.639743 -2.304487
C 3.672015 0.313601 -1.320554
C 2.665470 -1.097392 0.284049
F 1.615669 -1.784506 0.748789
F 3.644745 1.047014 -2.429764
F 5.919965 0.970863 -1.030388
F 6.071964 -0.462342 1.260115
F 3.905424 -1.829836 2.155518

^{Ph}YGePy
E = -3635.22926625
P -1.162732 -0.184726 0.039746
C 0.369847 0.361769 -0.484869
C 0.710664 1.784694 -0.372973
C 1.806133 2.334064 -1.069654
H 2.414263 1.675597 -1.686503
C 2.123348 3.682021 -0.993417
H 2.979754 4.055610 -1.553187
C 1.365502 4.553571 -0.216628

H 1.617027 5.610441 -0.157417
C 0.284172 4.038919 0.489462
H -0.324359 4.693172 1.112301
C -0.030125 2.688376 0.421151
H -0.875747 2.332443 1.007504
C -2.493226 0.911590 -0.564034
C -2.275207 1.544678 -1.791389
H -1.323725 1.398019 -2.300470
C -3.252936 2.368415 -2.334253
H -3.073632 2.862676 -3.286869
C -4.449154 2.572939 -1.653957
H -5.212788 3.222593 -2.077477
C -4.663028 1.957788 -0.425301
H -5.592068 2.126205 0.115744
C -3.687854 1.131389 0.122237
H -3.857985 0.674305 1.093215
C -1.531822 -1.839877 -0.605270
C -1.161434 -2.977784 0.115319
H -0.708463 -2.876617 1.099502
C -1.361294 -4.240229 -0.429486
H -1.063044 -5.123252 0.131717
C -1.931535 -4.372601 -1.690859
H -2.085682 -5.362280 -2.116548
C -2.305450 -3.241182 -2.409084
H -2.754408 -3.342975 -3.394937
C -2.106461 -1.976583 -1.869770
H -2.396903 -1.092453 -2.433495
C -1.409433 -0.360146 1.845851
C -2.498752 -1.045506 2.394063
H -3.249838 -1.488107 1.740546
C -2.610297 -1.189782 3.771360
H -3.456670 -1.728398 4.193213
C -1.633880 -0.656374 4.608823
H -1.722093 -0.772032 5.687444
C -0.539176 0.008627 4.067963
H 0.232350 0.412663 4.720165
C -0.421885 0.149604 2.689237
H 0.443745 0.645286 2.251011
Ge 1.615511 -1.015141 -0.969785
H 1.738554 -1.438908 -2.483027
H 2.147646 -0.310536 -2.479960
C 3.429398 -0.675493 -0.294880
N 4.423254 -1.352664 -0.872144
C 4.932596 0.336470 1.278343
C 5.654607 -1.192116 -0.404963
C 5.967272 -0.361898 0.668175
H 5.129032 1.000419 2.119024
H 6.442532 -1.758010 -0.907607
H 6.995153 -0.269929 1.012443
C 3.641788 0.180512 0.790200
H 2.804937 0.722990 1.227235

^{Ph}YGePyr
E = -3651.28903708
P -1.167546 -0.145901 0.031816
C 0.302344 0.332265 -0.696343
C 0.777200 1.712381 -0.534089
C 1.850726 2.212113 -1.296764
H 2.343238 1.545075 -2.001518
C 2.289924 3.521662 -1.177653

H 3.122426 3.859273 -1.793802
C 1.682949 4.401210 -0.285366
H 2.030416 5.428000 -0.190848
C 0.629545 3.932318 0.490508
H 0.141532 4.591788 1.206968
C 0.193398 2.619115 0.376425
H -0.617848 2.295042 1.025826
C -2.493167 1.051015 -0.349460
C -2.404260 1.710760 -1.578934
H -1.546504 1.515745 -2.221035
C -3.383466 2.621222 -1.954903
H -3.303121 3.135646 -2.910362
C -4.452406 2.886412 -1.105217
H -5.216630 3.604071 -1.397636
C -4.536016 2.244266 0.125411
H -5.363016 2.459781 0.799115
C -3.558801 1.331260 0.506313
H -3.623246 0.856344 1.481224
C -1.723438 -1.752434 -0.604182
C -1.395404 -2.934507 0.063191
H -0.851609 -2.895366 1.004749
C -1.754521 -4.162059 -0.480893
H -1.489938 -5.080427 0.039062
C -2.442252 -4.215087 -1.688023
H -2.722802 -5.177299 -2.112258
C -2.771925 -3.038800 -2.354698
H -3.311468 -3.078192 -3.298754
C -2.412976 -1.809747 -1.816643
H -2.668944 -0.890429 -2.339459
C -1.136796 -0.372619 1.846513
C -2.191589 -0.946134 2.564854
H -3.094178 -1.269325 2.047124
C -2.076979 -1.138077 3.935768
H -2.897222 -1.588740 4.491316
C -0.907288 -0.765809 4.594271
H -0.818477 -0.918075 5.668437
C 0.152439 -0.220026 3.878999
H 1.074783 0.050758 4.388911
C 0.046111 -0.029225 2.504555
H 0.889446 0.356715 1.929164
Ge 1.470157 -1.109777 -1.148110
H 1.620762 -1.585208 -2.642619
H 2.078411 -0.473441 -2.650952
C 3.272508 -0.801610 -0.426315
N 3.314515 -0.119287 0.721163
N 4.333714 -1.317196 -1.051563
C 4.509551 0.095204 1.257776
C 5.520581 -1.100441 -0.504549
C 5.676917 -0.373620 0.671618
H 4.535890 0.662211 2.191088
H 6.386273 -1.522022 -1.021347
H 6.654931 -0.191194 1.109673

^{Ph}YGeB2
E = -3794.25246723
P -1.760397 -0.157697 0.060610
C -0.189662 0.264071 -0.460595
C 0.230027 1.669848 -0.417910
C 1.330405 2.134800 -1.164982
H 1.890705 1.424516 -1.767791

C 1.710245 3.468484 -1.163424
H 2.568917 3.774684 -1.759518
C 1.009212 4.411004 -0.416987
H 1.309365 5.456793 -0.415759
C -0.080096 3.982379 0.332697
H -0.646160 4.694043 0.932193
C -0.454900 2.645823 0.339104
H -1.303765 2.358212 0.957477
C -3.007414 0.993030 -0.615654
C -2.728685 1.552867 -1.866216
H -1.781943 1.318759 -2.350701
C -3.637945 2.416981 -2.462282
H -3.410329 2.853924 -3.432432
C -4.826146 2.735427 -1.812683
H -5.536013 3.417022 -2.277533
C -5.099892 2.193307 -0.561749
H -6.022043 2.450880 -0.044515
C -4.193041 1.326981 0.039194
H -4.408369 0.929354 1.027184
C -2.213660 -1.815785 -0.523396
C -1.964605 -2.941247 0.265027
C -2.218221 -4.212008 -0.238171
C -2.722512 -4.364268 -1.524750
C -2.975059 -3.244398 -2.311504
C -2.720214 -1.972856 -1.814743
C -2.045976 -0.241270 1.867651
C -3.191443 -0.816370 2.428254
H -3.968697 -1.225365 1.783387
C -3.327218 -0.895531 3.808590
H -4.217471 -1.348318 4.240941
C -2.319520 -0.406674 4.636408
H -2.427074 -0.470966 5.717593
C -1.170529 0.147745 4.083699
H -0.375781 0.516261 4.729057
C -1.028437 0.223264 2.701894
H -0.121727 0.630907 2.255064
Ge 0.986686 -1.222762 -0.788254
H 0.975193 -1.724544 -2.304395
H 1.649587 -0.721229 -2.311993
H -1.564380 -2.825079 1.270145
H -2.014779 -5.086118 0.376889
H -2.919895 -5.360054 -1.917081
H -3.371942 -3.361505 -3.317801
H -2.913648 -1.097978 -2.432147
C 5.143620 -0.887525 -0.425230
C 4.727395 0.072916 0.492967
C 6.479801 -1.156349 -0.644760
C 5.626148 0.813785 1.233659
C 7.398428 -0.413336 0.101120
H 6.791217 -1.908645 -1.364366
C 6.981294 0.550168 1.018849
H 5.285232 1.563694 1.942222
H 8.462606 -0.590511 -0.039486
H 7.726406 1.110741 1.579361
B 2.950017 -0.811365 -0.443503
O 3.365567 0.119322 0.496290
O 4.046721 -1.449369 -1.007284

^{Ph}YGeB1
E = -4063.43862562

P 1.872253 -0.774398 0.036213
C 0.322732 -0.139207 0.343612
C 0.119905 0.778319 1.466057
C 1.151917 1.591406 1.984494
H 2.142332 1.558424 1.532583
C 0.946184 2.453854 3.051918
H 1.776621 3.060097 3.411642
C -0.305661 2.558690 3.651239
H -0.470138 3.243520 4.480720
C -1.338864 1.757943 3.170597
H -2.325508 1.805204 3.629800
C -1.130289 0.885438 2.111741
H -1.949478 0.254744 1.773758
C 3.065305 0.224979 -0.926849
C 4.234867 -0.319353 -1.469144
H 4.463639 -1.374591 -1.322308
C 5.094899 0.478418 -2.212337
H 6.001898 0.051575 -2.636364
C 4.789930 1.821724 -2.420336
H 5.464307 2.446970 -3.002701
C 3.620076 2.360529 -1.898054
H 3.372438 3.404874 -2.070857
C 2.751373 1.562947 -1.158774
H 1.816180 1.965925 -0.768413
C 1.749745 -2.316540 -0.917475
C 1.792713 -2.292544 -2.312906
H 1.985815 -1.358027 -2.835545
C 1.574920 -3.459370 -3.035973
H 1.602003 -3.433109 -4.123419
C 1.314461 -4.652732 -2.371965
H 1.142998 -5.565360 -2.939689
C 1.273084 -4.680713 -0.981038
H 1.072031 -5.613845 -0.458660
C 1.488168 -3.516697 -0.254502
H 1.449937 -3.539283 0.832663
C 2.719177 -1.182589 1.602733
C 4.098346 -1.078282 1.785165
H 4.732948 -0.710374 0.983470
C 4.668611 -1.425016 3.005397
H 5.745006 -1.338582 3.140475
C 3.867309 -1.870496 4.050498
H 4.316171 -2.136059 5.005841
C 2.489752 -1.962940 3.877958
H 1.856203 -2.293213 4.698625
C 1.915363 -1.616689 2.662049
H 0.835766 -1.656409 2.526514
Ge -1.089617 -0.599193 -0.897242
N -4.028432 0.529129 -0.276957
C -3.614088 2.724476 -0.139843
C -4.555754 1.777489 0.048209
H -3.698134 3.799814 -0.033736
H -5.593707 1.905053 0.330589
B -2.648566 0.710366 -0.661647
N -2.444184 2.130283 -0.603712
C -1.300748 2.898434 -0.905782
C -0.763021 3.763514 0.047473
C -0.738354 2.840056 -2.182143
C 0.297783 4.594794 -0.290282
H -1.170870 3.761379 1.056359
C 0.321131 3.675779 -2.514251

H -1.165633 2.159709 -2.915947
C 0.830723 4.567907 -1.575146
H 0.711199 5.265022 0.460740
H 0.742363 3.636487 -3.517188
H 1.646772 5.237338 -1.841779
C -4.740899 -0.667373 -0.124869
C -4.491224 -1.735871 -0.990421
C -5.694264 -0.820952 0.885284
C -5.174637 -2.934721 -0.840976
H -3.766919 -1.606766 -1.793682
C -6.384473 -2.019138 1.019052
H -5.875553 -0.006408 1.582580
C -6.128545 -3.083142 0.160843
H -4.966733 -3.756618 -1.523670
H -7.121742 -2.124200 1.812980
H -6.668531 -4.021035 0.271934
H -2.035148 -1.549689 0.220035
H -1.559396 -2.104110 -0.733386

$^{F}Y_2Ge$
E = -5688.93541287
P -3.179496 0.612147 -0.118706
P 2.798555 1.177844 -0.012364
C -1.593151 0.160368 0.286165
C -1.234276 -1.133029 0.849361
C -1.805909 -2.354461 0.469818
C -1.374595 -3.581004 0.948394
C -0.330737 -3.630228 1.863119
C 0.239514 -2.442467 2.301881
C -0.221116 -1.227636 1.816044
C -3.709979 0.384738 -1.855813
C -4.308067 -0.793944 -2.313483
H -4.590804 -1.571934 -1.610226
C -4.539131 -0.980403 -3.670131
H -5.011853 -1.898883 -4.012206
C -4.163849 -0.004457 -4.588191
H -4.343654 -0.156905 -5.650655
C -3.550829 1.161129 -4.144726
H -3.244722 1.924798 -4.856888
C -3.321962 1.353720 -2.787635
H -2.845395 2.272414 -2.447028
C -3.421069 2.400913 0.165168
C -4.535434 3.053072 -0.367903
H -5.213266 2.519166 -1.032473
C -4.778211 4.385392 -0.058262
H -5.647276 4.888895 -0.477014
C -3.910207 5.072066 0.785441
H -4.098521 6.116967 1.025295
C -2.806442 4.421315 1.326020
H -2.127776 4.955660 1.987494
C -2.563479 3.086146 1.024566
H -1.710865 2.559760 1.449243
C -4.413179 -0.179514 0.951788
C -4.033636 -0.540908 2.247000
H -3.007420 -0.382739 2.569273
C -4.968570 -1.083064 3.119971
H -4.664248 -1.370245 4.124388
C -6.288089 -1.251929 2.714381
H -7.018589 -1.678058 3.399329
C -6.676370 -0.864420 1.436234

H -7.711180 -0.980475 1.120445
C -5.744638 -0.325759 0.556764
H -6.058021 -0.016655 -0.438156
C 1.519929 0.186479 -0.617188
C 1.722948 -1.205639 -0.997590
C 0.717200 -2.052220 -1.520430
C 0.881797 -3.416139 -1.719794
C 2.109383 -4.028463 -1.523978
C 3.158221 -3.230434 -1.095559
C 2.958661 -1.876900 -0.879505
C 4.114996 1.468768 -1.244427
C 3.713132 1.511663 -2.581415
H 2.664450 1.342172 -2.825169
C 4.647963 1.744147 -3.583801
H 4.328710 1.775767 -4.623660
C 5.988793 1.919007 -3.258087
H 6.722316 2.092559 -4.043155
C 6.394410 1.853930 -1.928974
H 7.445700 1.970438 -1.673088
C 5.462962 1.627888 -0.922606
H 5.794878 1.553134 0.109404
C 2.139948 2.822203 0.398039
C 1.445672 2.967455 1.605352
H 1.337279 2.115529 2.274220
C 0.879756 4.190102 1.934295
H 0.334369 4.297086 2.870378
C 1.004733 5.273136 1.067694
H 0.555310 6.230757 1.324301
C 1.712917 5.135581 -0.120038
H 1.820769 5.983926 -0.792752
C 2.286910 3.913352 -0.454911
H 2.835778 3.806724 -1.387814
C 3.598024 0.729505 1.567225
C 4.387076 1.662968 2.247006
H 4.554233 2.650756 1.819136
C 4.938519 1.342318 3.480786
H 5.554893 2.070695 4.004093
C 4.688409 0.097394 4.051369
H 5.114694 -0.150943 5.021537
C 3.880887 -0.822010 3.391704
H 3.661187 -1.786771 3.841831
C 3.336757 -0.506535 2.152798
H 2.688590 -1.214925 1.644074
Ge -0.172868 1.135458 -0.612655
H -0.521458 1.868001 -1.947820
H -0.532322 0.726866 -2.351429
F -2.778401 -2.357691 -0.437559
F -1.933943 -4.703170 0.526074
F 0.099131 -4.790891 2.332331
F -0.154891 -4.131916 -2.131899
F 1.199726 -2.482927 3.220789
F -0.493108 -1.595379 -1.827308
F 0.316866 -0.115528 2.320591
F 2.272720 -5.326772 -1.730838
F 4.039746 -1.192752 -0.474355
F 4.361104 -3.753775 -0.907550

^{F}*YGeCl*
E = -4344.63965990
P -0.869599 0.503710 -0.243240

C 0.152635 -0.840479 0.034835
C 1.581210 -0.666137 0.231544
C 2.156481 0.422228 0.900674
C 3.526515 0.602035 1.019531
C 4.395590 -0.327460 0.464618
C 3.871658 -1.425585 -0.208292
C 2.499168 -1.567155 -0.330134
C -1.701953 1.249297 1.193840
C -2.722245 2.190193 1.020269
H -3.013672 2.502627 0.017448
C -3.375402 2.715081 2.127111
H -4.167477 3.449122 1.993094
C -3.023550 2.290227 3.406503
H -3.541384 2.696745 4.273249
C -2.024246 1.339494 3.578156
H -1.762346 0.995232 4.576332
C -1.362898 0.815610 2.472802
H -0.594816 0.053786 2.588217
C -2.264066 0.002487 -1.295902
C -3.373679 -0.606723 -0.700102
H -3.425972 -0.713400 0.382247
C -4.406369 -1.093046 -1.494435
H -5.260259 -1.577991 -1.026312
C -4.345938 -0.959515 -2.876449
H -5.158257 -1.335279 -3.495558
C -3.247425 -0.344216 -3.470115
H -3.201274 -0.233591 -4.551541
C -2.204598 0.130437 -2.685172
H -1.347729 0.607893 -3.154781
C 0.072736 1.802151 -1.091175
C 0.000026 3.147888 -0.732146
H -0.643403 3.462667 0.085410
C 0.774568 4.085937 -1.404670
H 0.718644 5.134253 -1.118996
C 1.627450 3.686278 -2.427900
H 2.236254 4.423895 -2.947130
C 1.715280 2.342359 -2.777438
H 2.394406 2.024312 -3.565718
C 0.946194 1.399726 -2.107553
H 1.027835 0.341540 -2.352706
Ge -0.747461 -2.535054 0.105977
H 0.633018 -3.252841 0.917089
H 0.026941 -3.875698 -0.007721
Cl -1.989496 -2.623174 1.958315
F 2.041153 -2.592312 -1.051342
F 4.688242 -2.308166 -0.762749
F 5.705602 -0.175370 0.580827
F 4.007898 1.645896 1.676530
F 1.363863 1.331072 1.474842

^{F}YGeHMDS
E = -4758.43020898
P -1.963740 -0.369089 -0.194487
C -0.285446 -0.069481 -0.267145
C 0.231354 1.279685 -0.136969
C -0.279420 2.207571 0.784082
C 0.222425 3.486964 0.948817
C 1.296523 3.908974 0.176124
C 1.826548 3.034653 -0.764097
C 1.285651 1.767550 -0.923653

C -2.744481 -0.809090 1.394571
C -4.097395 -1.152862 1.488590
H -4.727327 -1.144508 0.599390
C -4.637636 -1.519713 2.714627
H -5.690755 -1.784608 2.785533
C -3.829947 -1.554657 3.849095
H -4.254469 -1.846379 4.807828
C -2.483610 -1.221581 3.757187
H -1.849658 -1.251553 4.640890
C -1.942319 -0.847761 2.532195
H -0.892321 -0.576991 2.442759
C -2.312808 -1.792651 -1.282814
C -2.829042 -3.003661 -0.817078
H -3.088456 -3.124022 0.230781
C -2.981747 -4.077609 -1.686862
H -3.375145 -5.020187 -1.311805
C -2.619652 -3.954354 -3.022921
H -2.736628 -4.798362 -3.699897
C -2.095959 -2.752801 -3.491258
H -1.798896 -2.653435 -4.533156
C -1.937872 -1.678997 -2.626345
H -1.492659 -0.755905 -2.989147
C -2.830044 1.099632 -0.836010
C -3.943185 1.672167 -0.219776
H -4.348551 1.243272 0.692196
C -4.522033 2.818542 -0.753948
H -5.387292 3.260567 -0.264232
C -3.991744 3.405378 -1.896956
H -4.446021 4.304654 -2.308329
C -2.866431 2.853751 -2.500766
H -2.430201 3.322875 -3.380142
C -2.282399 1.712792 -1.968991
H -1.372668 1.310846 -2.410368
Ge 0.825564 -1.645143 -0.380776
H 1.112283 -1.602446 -2.162078
H 0.689684 -2.596213 -1.606501
Si 2.680381 -1.153659 1.927480
Si 3.996970 -1.081564 -0.856575
C 1.607491 -2.475606 2.730395
H 0.552881 -2.429778 2.432845
H 1.976692 -3.474537 2.464700
H 1.650490 -2.375388 3.823512
C 4.418993 -1.427423 2.583243
H 5.166716 -0.747307 2.160176
H 4.392344 -1.255258 3.667712
H 4.756749 -2.456368 2.415881
C 2.110722 0.535604 2.528777
H 1.020409 0.637170 2.478389
H 2.414325 0.699065 3.571168
H 2.544242 1.336850 1.914705
C 3.672093 -1.290589 -2.692930
H 3.328362 -2.304735 -2.932225
H 2.955472 -0.575393 -3.104388
H 4.636466 -1.142666 -3.200473
C 5.285657 -2.408006 -0.479274
H 6.011845 -2.443821 -1.302701
H 5.846386 -2.235055 0.444009
H 4.812897 -3.396597 -0.410508
C 4.762242 0.610179 -0.555237
H 4.892293 0.815891 0.514716

H 5.751801 0.677036 -1.026681
H 4.139388 1.407151 -0.975081
N 2.574062 -1.285586 0.170913
F 1.785270 1.011426 -1.896933
F 2.822248 3.433738 -1.544586
F 1.791426 5.129237 0.316636
F -0.297253 4.297989 1.858232
F -1.264267 1.828682 1.606158

$^{F}YGeC_6F_5$
E = -4612.97387142
P 1.504443 -0.631991 0.126816
C -0.050676 -0.680165 -0.569221
C -0.722978 -1.969533 -0.779239
C -1.840559 -2.080431 -1.628113
H -2.208606 -1.189524 -2.131862
C -2.472361 -3.295520 -1.851832
H -3.333108 -3.330083 -2.518049
C -2.017367 -4.459346 -1.241285
H -2.515943 -5.410532 -1.414761
C -0.915211 -4.378680 -0.397637
H -0.539028 -5.272056 0.098344
C -0.289136 -3.162501 -0.163887
H 0.558116 -3.148407 0.519532
C 2.603703 -1.850653 -0.666215
C 2.338057 -2.160627 -2.002895
H 1.518376 -1.657516 -2.512036
C 3.104748 -3.110415 -2.660306
H 2.889308 -3.352601 -3.698766
C 4.138918 -3.758331 -1.990260
H 4.737976 -4.505844 -2.506806
C 4.399199 -3.459696 -0.658229
H 5.198824 -3.972981 -0.128177
C 3.629924 -2.511818 0.008653
H 3.823204 -2.309834 1.058258
C 2.207896 1.046800 -0.059663
C 1.828408 2.053929 0.831969
C 2.308587 3.350189 0.726234
C 3.184880 3.670182 -0.303749
C 3.571280 2.695303 -1.213906
C 3.072949 1.403577 -1.092901
C 1.683088 -0.900887 1.926835
C 2.858974 -0.582753 2.612352
H 3.719782 -0.186653 2.073418
C 2.927319 -0.751452 3.989147
H 3.842168 -0.499792 4.522015
C 1.821288 -1.231101 4.686163
H 1.875008 -1.359665 5.765483
C 0.644417 -1.528118 4.008356
H -0.226854 -1.883697 4.554336
C 0.568286 -1.355198 2.630349
H -0.362273 -1.547174 2.099180
Ge -0.965595 0.982375 -0.728199
H -0.833209 1.839337 -2.029733
H -1.417242 0.835926 -2.415833
C -2.880275 0.837299 -0.307048
C -4.682992 -0.088295 1.039278
C -5.193968 1.471317 -0.719357
C -5.615355 0.642776 0.314907
F 0.959460 1.805486 1.797190

F 1.925422 4.280336 1.578379
F 3.645864 4.896605 -0.418290
F 4.398819 3.000862 -2.195054
F 3.451789 0.527326 -2.005045
C -3.839094 1.559355 -1.005519
C -3.334284 0.027322 0.724517
F -3.464793 2.356319 -2.003772
F -2.475778 -0.675776 1.460700
F -5.082325 -0.872222 2.026959
F -6.900405 0.552737 0.607501
F -6.082509 2.166082 -1.410725

^{F}YGePy
E = -4132.12258437
P -0.680978 0.732334 0.196324
C 0.864053 0.519395 -0.492026
C 1.780799 1.666266 -0.566294
C 2.936507 1.631077 -1.371657
H 3.153144 0.727108 -1.936561
C 3.798080 2.714102 -1.466809
H 4.677687 2.634188 -2.103965
C 3.551123 3.889634 -0.764653
H 4.228519 4.737518 -0.840750
C 2.421488 3.951767 0.043082
H 2.202554 4.855226 0.610335
C 1.563671 2.865577 0.147463
H 0.702964 2.962861 0.806819
C -1.513838 2.223668 -0.447985
C -1.159974 2.628363 -1.737800
H -0.430078 2.044549 -2.295528
C -1.725852 3.769045 -2.287021
H -1.441550 4.083133 -3.289058
C -2.646466 4.513532 -1.554804
H -3.088325 5.409613 -1.986466
C -2.992950 4.119391 -0.267933
H -3.703511 4.705368 0.311449
C -2.423609 2.979706 0.290678
H -2.679820 2.699463 1.308338
C -1.728724 -0.727279 -0.140925
C -1.569612 -1.878157 0.635353
C -2.316979 -3.024350 0.415006
C -3.245558 -3.042918 -0.619061
C -3.417562 -1.920329 -1.417115
C -2.654453 -0.783003 -1.181000
C -0.811064 0.853599 2.020334
C -2.025485 0.705129 2.695589
H -2.946878 0.538170 2.137739
C -2.059703 0.745644 4.083869
H -3.006424 0.624576 4.606649
C -0.882339 0.928703 4.803678
H -0.910187 0.957686 5.891191
C 0.330129 1.056241 4.135110
H 1.253536 1.181130 4.696811
C 0.368796 1.008326 2.746182
H 1.317225 1.072000 2.214806
Ge 1.340455 -1.282649 -0.952033
H 1.081225 -1.767571 -2.429536
H 1.943691 -0.940125 -2.549053
C 3.173593 -1.762891 -0.446254
N 3.698205 -2.837748 -1.034637

C 5.124296 -1.454138 0.912461
C 4.916643 -3.222709 -0.676458
C 5.673918 -2.569263 0.292243
H 5.681147 -0.909659 1.673637
H 5.316500 -4.103778 -1.183456
H 6.667173 -2.930207 0.550203
F -0.660571 -1.918425 1.594892
F -2.139058 -4.097373 1.160449
F -3.960322 -4.124685 -0.842174
F -4.295820 -1.940478 -2.402138
F -2.840358 0.241032 -1.993822
C 3.852655 -1.040051 0.538113
H 3.389751 -0.162355 0.986010

^{F}YGePyr
E = -4148.18183200
P -0.596093 0.741993 0.218296
C 0.808181 0.413917 -0.686543
C 1.875992 1.424378 -0.753871
C 2.924758 1.312618 -1.685583
H 2.944173 0.449775 -2.348055
C 3.924467 2.268340 -1.784270
H 4.712620 2.136460 -2.524379
C 3.929518 3.384540 -0.952903
H 4.715787 4.132764 -1.029016
C 2.912548 3.515277 -0.014750
H 2.895047 4.371225 0.658374
C 1.915684 2.554380 0.089298
H 1.156987 2.691113 0.857635
C -1.269183 2.391860 -0.175689
C -1.021111 2.877842 -1.462358
H -0.469338 2.256258 -2.164895
C -1.463442 4.140909 -1.825128
H -1.261212 4.515923 -2.826161
C -2.154555 4.928710 -0.908743
H -2.499655 5.920630 -1.194378
C -2.393077 4.453489 0.375077
H -2.921484 5.071218 1.098367
C -1.945590 3.189713 0.746728
H -2.109486 2.844396 1.763088
C -1.872622 -0.524714 -0.126779
C -1.855324 -1.745970 0.552522
C -2.785572 -2.742973 0.298851
C -3.758046 -2.539009 -0.671800
C -3.792676 -1.343254 -1.376409
C -2.847622 -0.360350 -1.110257
C -0.468054 0.671301 2.042193
C -1.580546 0.677471 2.887871
H -2.586274 0.752819 2.474265
C -1.409170 0.559112 4.261070
H -2.276373 0.559502 4.918592
C -0.128658 0.427068 4.792237
H 0.004030 0.332681 5.868384
C 0.976390 0.392695 3.949336
H 1.974779 0.263575 4.362192
C 0.811249 0.502502 2.572542
H 1.666558 0.418770 1.900163
Ge 1.118873 -1.430575 -1.075836
H 0.804293 -1.997628 -2.511437
H 1.719462 -1.238569 -2.693376

C 2.934928 -1.967767 -0.561993
N 3.448375 -1.308036 0.477874
N 3.531973 -2.968377 -1.211866
C 4.667109 -1.655995 0.873563
C 4.746630 -3.309306 -0.806369
C 5.384833 -2.667165 0.249841
H 5.085479 -1.107401 1.720280
H 5.234096 -4.127462 -1.341886
H 6.384547 -2.946312 0.572772
F -0.919630 -2.011517 1.447228
F -2.738084 -3.887046 0.953721
F -4.643879 -3.478496 -0.925305
F -4.711584 -1.151422 -2.304495
F -2.909182 0.740253 -1.837413

[F]YGeB2
E = -4291.15537747
P -1.594318 -0.810446 0.096916
C -0.287170 0.186517 -0.373203
C -0.325077 1.637756 -0.305602
C 0.826033 2.394295 -0.018717
C 0.832931 3.779538 0.028187
C -0.349780 4.489170 -0.143955
C -1.518237 3.786648 -0.401911
C -1.488564 2.402396 -0.475343
C -2.832107 -1.281193 -1.167358
C -2.446222 -2.217973 -2.133676
H -1.479035 -2.713207 -2.053508
C -3.287384 -2.518082 -3.197435
H -2.976564 -3.250136 -3.940064
C -4.520100 -1.883496 -3.312088
H -5.179947 -2.119700 -4.144703
C -4.904316 -0.941977 -2.363578
H -5.863350 -0.435678 -2.453400
C -4.064834 -0.636307 -1.299079
H -4.367443 0.116068 -0.575822
C -0.916624 -2.402359 0.679243
C 0.348569 -2.412355 1.273516
C 0.860621 -3.591202 1.801752
C 0.107743 -4.759719 1.760920
C -1.163308 -4.747720 1.194833
C -1.676870 -3.574024 0.656066
C -2.505330 -0.182865 1.536980
C -3.736970 -0.738169 1.893347
H -4.191809 -1.506068 1.270397
C -4.382426 -0.320034 3.049744
H -5.342518 -0.755100 3.319639
C -3.800245 0.648700 3.861265
H -4.308888 0.979313 4.764754
C -2.562045 1.183654 3.524468
H -2.095751 1.928967 4.165458
C -1.908321 0.764311 2.371186
H -0.927125 1.160682 2.123612
Ge 1.000409 -0.786992 -1.453427
H 0.898073 -0.343911 -2.991937
H 1.388383 0.551262 -2.445413
H 0.932201 -1.494214 1.319394
H 1.853686 -3.590431 2.246302
H 0.510015 -5.682652 2.174343
H -1.757858 -5.658686 1.167626

H -2.669817 -3.573476 0.210799
C 5.124659 -0.474382 -0.788833
C 4.650564 -0.682045 0.503216
C 6.471785 -0.345426 -1.060261
C 5.499423 -0.770033 1.587857
C 7.340400 -0.433521 0.030321
H 6.828240 -0.182124 -2.073607
C 6.865291 -0.640857 1.324867
H 5.115196 -0.928258 2.591924
H 8.411119 -0.336254 -0.135541
H 7.572603 -0.701526 2.149149
B 2.937243 -0.594620 -0.865070
O 3.290526 -0.772652 0.464615
O 4.064951 -0.427389 -1.647858
F 1.978859 1.773181 0.218562
F 1.956110 4.430317 0.291641
F -0.358089 5.812054 -0.073549
F -2.641033 1.782291 -0.748910
F -2.656992 4.439213 -0.583584

^{F}*YGeB1*
E = -4560.34530620
P -1.740065 1.323854 0.128783
C -0.355543 0.332150 0.163397
C -0.442281 -1.033196 0.657382
C -1.481071 -1.908199 0.299903
C -1.573018 -3.211927 0.753411
C -0.594330 -3.718501 1.596459
C 0.462193 -2.899278 1.968614
C 0.513737 -1.584527 1.528294
C -2.642195 1.540026 -1.447288
C -3.613990 2.533244 -1.603819
H -3.858330 3.193530 -0.771989
C -4.258855 2.685799 -2.824240
H -5.017333 3.456873 -2.944915
C -3.926835 1.858787 -3.895409
H -4.428693 1.984547 -4.853027
C -2.951123 0.880023 -3.744967
H -2.686876 0.238738 -4.584006
C -2.309235 0.720717 -2.521236
H -1.547657 -0.041173 -2.373025
C -1.324408 3.050925 0.552696
C -0.905823 3.924037 -0.455736
H -0.896542 3.592951 -1.492271
C -0.492664 5.212533 -0.139042
H -0.163740 5.882431 -0.930928
C -0.499422 5.642217 1.183461
H -0.178897 6.652555 1.430337
C -0.918779 4.778954 2.190538
H -0.930478 5.111772 3.226438
C -1.326079 3.487468 1.878854
H -1.653604 2.821571 2.673404
C -2.936336 0.722838 1.355793
C -4.292646 0.568461 1.069964
H -4.677355 0.824690 0.086223
C -5.153282 0.061837 2.037385
H -6.209542 -0.060571 1.806683
C -4.666028 -0.299245 3.288297
H -5.342230 -0.700797 4.040576
C -3.311052 -0.160914 3.573628

H -2.922460 -0.457695 4.545601
C -2.446239 0.341719 2.610596
H -1.379982 0.424088 2.815948
Ge 1.242486 0.987586 -0.712516
N 4.125034 -0.306978 -0.580092
C 3.769645 -2.450176 -1.115919
C 4.703841 -1.565095 -0.719307
H 3.893647 -3.493913 -1.377459
H 5.766098 -1.722347 -0.575420
B 2.720963 -0.419481 -0.886108
N 2.540854 -1.802694 -1.254477
C 1.391383 -2.492731 -1.679495
C 1.154216 -3.800875 -1.250312
C 0.481149 -1.889126 -2.550902
C 0.022937 -4.485995 -1.674300
H 1.843804 -4.266948 -0.549721
C -0.653888 -2.577350 -2.960642
H 0.685056 -0.885479 -2.920003
C -0.893933 -3.875464 -2.522744
H -0.159947 -5.494102 -1.306748
H -1.358049 -2.096063 -3.636925
H -1.789513 -4.405590 -2.837677
C 4.837953 0.805820 -0.107959
C 4.575500 2.073620 -0.630338
C 5.809677 0.666182 0.885995
C 5.261607 3.183245 -0.154863
H 3.839177 2.176376 -1.425956
C 6.504120 1.778285 1.344486
H 5.999569 -0.314766 1.315690
C 6.232311 3.042290 0.831377
H 5.043316 4.165284 -0.570495
H 7.255588 1.655456 2.122287
H 6.774154 3.911666 1.197710
H 2.153644 1.423067 0.691843
H 1.772485 2.315892 -0.048995
F 1.510568 -0.833054 1.984761
F -2.421742 -1.490987 -0.551986
F -2.549450 -4.002124 0.328316
F 1.406358 -3.370317 2.772817
F -0.640979 -4.983402 1.994540

I
E = -1585.95026060
C 1.651354 2.362887 0.117609
C -0.481928 1.226109 -0.021896
C -1.089202 2.443316 -0.323409
C -0.337777 3.614781 -0.389338
C 1.030777 3.577633 -0.160291
H 2.726421 2.348804 0.297140
H -2.156032 2.475232 -0.526531
H -0.832585 4.555224 -0.623309
H 1.622291 4.490293 -0.198967
N -1.145540 -0.001852 0.052808
C -2.531186 -0.182229 0.012433
C -3.413284 0.615266 0.753934
C -3.053899 -1.236725 -0.745191
C -4.778115 0.365499 0.720720
H -3.016070 1.418097 1.370175
C -4.419782 -1.489742 -0.760224
H -2.370206 -1.841999 -1.340692

C -5.291485 -0.686750 -0.033350
H -5.448800 0.993183 1.305263
H -4.805348 -2.315054 -1.356383
H -6.362078 -0.879564 -0.050504
C 0.929837 1.164579 0.177659
C 1.491206 -0.160791 0.400669
P 3.082336 -0.600113 0.017434
C 3.615104 -0.068375 -1.638094
H 3.488131 1.017363 -1.712485
H 4.657328 -0.335275 -1.845400
H 2.949027 -0.542213 -2.368196
C 4.377068 0.004686 1.143223
H 5.366197 -0.352313 0.833657
H 4.376627 1.099431 1.152107
H 4.149985 -0.347986 2.154536
C 3.208122 -2.401712 0.028341
H 4.231127 -2.723689 -0.191256
H 2.906275 -2.781962 1.010002
H 2.530064 -2.792606 -0.742547
H -0.137719 -2.736415 -0.447981
H 0.274006 -2.058221 -1.575736
Ge 0.068570 -1.392696 0.266537

//
E = -3646.42412950
C 0.903606 2.925174 -0.050865
C 0.431266 1.649515 0.236616
C -0.788955 1.174535 -0.290820
C -1.489422 2.048774 -1.140650
C -1.018313 3.322266 -1.417444
C 0.182434 3.774751 -0.876533
H 1.846026 3.229624 0.400127
H -2.421719 1.707181 -1.583054
H -1.596684 3.967297 -2.076753
H 0.548605 4.775768 -1.092030
N -1.285282 -0.097396 -0.023227
S 1.398823 0.617379 1.321094
O 2.702006 1.286189 1.474959
O 0.559814 0.341377 2.490894
C 1.583217 -0.843023 0.445858
C -2.674822 -0.327917 -0.065957
C -3.557167 0.488671 0.653002
C -3.187138 -1.408029 -0.787531
C -4.918082 0.226814 0.640131
H -3.154414 1.321625 1.225532
C -4.552873 -1.676572 -0.780684
H -2.508415 -2.020712 -1.379010
C -5.424026 -0.860163 -0.071847
H -5.591903 0.867681 1.206022
H -4.935726 -2.524128 -1.346324
H -6.492189 -1.066763 -0.070541
P 2.917452 -0.918644 -0.626157
C 3.028592 0.446245 -1.817113
H 3.893194 0.333006 -2.480696
H 2.102822 0.485255 -2.401656
H 3.119719 1.375441 -1.243664
C 2.875514 -2.442085 -1.589601
H 1.970561 -2.455729 -2.206268
H 3.768399 -2.497994 -2.222140
H 2.853166 -3.298981 -0.908377

C 4.488632 -0.917362 0.266248
H 4.498696 -0.027074 0.902867
H 4.527868 -1.808843 0.900375
H 5.334665 -0.908876 -0.430255
Ge -0.178573 -1.569986 0.407457
H -0.569040 -2.870514 -0.329853
H -0.014611 -2.270461 -1.329150

III
E = -4408.51351673
C 0.348272 1.728415 0.535327
C -0.456235 1.700868 -0.609685
C -0.844941 2.902976 -1.202197
C -0.467203 4.123325 -0.659998
C 0.309389 4.152166 0.491085
C 0.711107 2.961150 1.079786
H -1.468552 2.852970 -2.091057
H -0.783837 5.048314 -1.137898
H 0.607205 5.100496 0.934112
H 1.334372 2.959335 1.970348
S 0.954103 0.304667 1.514449
S -1.035310 0.220092 -1.506658
O 2.156862 0.866662 2.165486
O -0.187162 -0.074322 2.358881
O 0.133910 -0.212481 -2.302375
O -2.232902 0.721633 -2.205866
C 1.447433 -0.949420 0.491975
C -1.438494 -0.998533 -0.422162
P -2.875280 -0.755114 0.483585
P 2.924257 -0.664656 -0.342763
C -2.926546 -1.836485 1.920049
H -3.855586 -1.655990 2.472130
H -2.879748 -2.884245 1.608820
H -2.053693 -1.594095 2.536448
C -3.080582 0.926587 1.123363
H -3.101807 1.622904 0.278371
H -4.016638 1.002879 1.687879
H -2.220832 1.142628 1.767387
C -4.357446 -1.070560 -0.508493
H -4.360995 -2.121877 -0.814699
H -5.276659 -0.832555 0.038946
H -4.266504 -0.445099 -1.403680
C 3.051057 -1.822391 -1.719198
H 4.003142 -1.672198 -2.239531
H 2.985990 -2.851055 -1.351042
H 2.212234 -1.624688 -2.397422
C 3.070967 0.982270 -1.080205
H 3.030178 1.730141 -0.281565
H 4.016436 1.073451 -1.626506
H 2.218780 1.113164 -1.757590
C 4.385435 -0.872648 0.703260
H 5.308449 -0.625879 0.166476
H 4.246141 -0.208137 1.562705
H 4.418917 -1.907621 1.058985
H -0.077739 -3.499377 0.522293
H -0.375053 -2.692675 1.414723
Ge 0.107547 -2.128394 -0.191939

IV
Not observed.

V
Not observed.

Pathway B

$^{Tos}Y_2Ge$
E = -5872.32529315
C 3.638979 2.965356 0.020368
H 3.115636 3.252837 0.928618
C 3.064652 2.016038 -0.811416
C -4.218166 -1.446085 -1.212975
O 0.505760 1.683915 -1.399294
C -4.443509 -0.807950 -2.429479
H -3.611609 -0.384294 -2.992220
C 2.623859 -3.189106 2.290214
H 3.635200 -2.853837 2.516762
C 4.875622 3.502924 -0.317393
H 5.334608 4.242280 0.337910
C -0.838145 4.920328 0.540632
H -0.245595 5.567776 1.183753
C -1.111862 3.620154 0.946535
H -0.700276 3.250731 1.879788
C 3.175483 -2.193842 -2.542828
H 2.414534 -1.456089 -2.793537
C 3.501869 -2.401931 -1.204593
C 5.247671 -0.381643 0.499090
H 5.525448 -0.836214 -0.449348
C 6.159939 0.414358 1.182295
H 7.153978 0.571902 0.768107
C -1.296754 5.383430 -0.686560
H -1.071090 6.399555 -1.005038
C -1.858128 0.001676 -0.697561
Ge -0.147183 -0.137970 -2.076994
O -2.680640 -2.018253 0.820869
S -2.552059 -1.572391 -0.580513
P -2.235688 1.076468 0.615853
C -5.741157 -0.701600 -2.903618
H -5.923843 -0.196998 -3.851704
C -4.459097 0.187135 2.068450
H -3.738004 -0.367022 2.662868
C -0.537256 -0.325591 2.280209
H -0.284134 -0.864870 1.374807
C 4.520333 0.828818 2.900695
H 4.230122 1.310665 3.832084
C 3.601690 0.039106 2.222646
H 2.588635 -0.073488 2.605044
C -2.307773 3.241412 -1.116803
H -2.836482 2.579224 -1.797142
C 1.940937 -2.670019 1.187759
C 3.702421 1.609193 -1.978859
H 3.238756 0.856088 -2.613361
C 4.450924 -3.368316 -0.859645
H 4.695988 -3.543625 0.187612
C 3.969228 -0.581311 1.023556
C -1.215663 1.110039 4.568439
H -1.481228 1.669956 5.462976
C -1.442556 0.724348 2.201531
C -1.791351 1.441534 3.348970
H -2.522977 2.246990 3.292242

C -1.863712 2.778757 0.126159
C -2.028233 4.540788 -1.516092
H -2.368709 4.887581 -2.489350
C 6.903522 3.645116 -1.806043
H 7.690723 2.945040 -1.492252
H 7.091366 4.597491 -1.299157
H 7.021845 3.800349 -2.884461
C -0.317126 0.048118 4.647173
H 0.116504 -0.223215 5.608225
C -6.821092 -1.230564 -2.185832
C 5.799531 1.014516 2.383805
H 6.514823 1.639619 2.915161
C -6.315615 1.521638 0.481788
H -7.037451 2.039732 -0.146341
C 1.534323 -0.367419 -0.567136
P 2.667035 -1.423926 0.081702
O 1.229294 1.814480 1.003646
S 1.528812 1.245777 -0.321103
O -1.812812 -2.444878 -1.509404
C 5.546163 3.102841 -1.473386
C 4.934631 2.154641 -2.302461
H 5.437929 1.836623 -3.214739
C 3.802740 -2.943073 -3.533897
H 3.538204 -2.785816 -4.577560
C 4.758270 -3.891917 -3.190174
H 5.249601 -4.475449 -3.966572
C 5.082669 -4.105034 -1.851821
H 5.825937 -4.852871 -1.582438
C -4.958404 1.657548 0.222354
H -4.634950 2.284907 -0.603247
C 0.014751 -0.674818 3.506627
H 0.695719 -1.521631 3.566106
C -4.018832 1.001201 1.021418
C -6.748210 0.731046 1.541965
H -7.812338 0.628838 1.746763
C -6.560383 -1.880625 -0.980316
H -7.387879 -2.299026 -0.408626
C -5.818788 0.063430 2.330244
H -6.150795 -0.568670 3.151562
C 0.688879 -4.515256 2.850528
H 0.194171 -5.224157 3.512021
C 1.995948 -4.115373 3.116661
H 2.524434 -4.514804 3.980310
C -5.264693 -1.990445 -0.486782
H -5.052417 -2.474778 0.463320
C -8.222445 -1.075006 -2.695334
H -8.568880 -0.038669 -2.580426
H -8.921114 -1.719643 -2.152000
H -8.290476 -1.319373 -3.762111
C 0.010987 -4.004567 1.747268
H -1.018683 -4.284929 1.538174
C 0.640231 -3.093914 0.908425
H 0.097698 -2.688757 0.054394
H -1.979028 0.646684 -1.852163
H -1.538929 1.023475 -2.843831

^{Tos}YGeCl

^{Tos}YGeHMDS

^{Tos}YGeB1
E = -4652.03189161
C -3.434275 0.506901 -1.235849
C -3.391746 1.417279 -2.287837
H -2.637208 1.304997 -3.062384
C 3.438509 -0.133235 2.281533
H 4.428315 -0.575818 2.199804
C 2.355889 -0.760662 1.676738
H 2.505488 -1.683738 1.122955
C 3.257326 1.058758 2.971207
H 4.105012 1.547670 3.447581
C -0.814909 -0.138488 -0.389460
Ge 0.153636 1.254313 -1.613895
O -2.783542 -1.834457 -0.255455
S -2.227147 -0.799759 -1.150853
P -0.358771 -1.023988 1.023785
C -4.324417 2.442846 -2.328658
H -4.293541 3.159780 -3.148194
C -2.009603 -2.029499 3.106634
H -1.501106 -2.982420 2.992635
C -0.200036 -3.470395 -0.301344
H -0.664993 -2.963155 -1.141682
C 0.908029 1.007494 2.450597
H -0.077298 1.459349 2.528041
C 1.022624 -4.780303 1.839519
H 1.504425 -5.287656 2.672980
C 0.106612 -2.769035 0.862059
C 0.724449 -3.428479 1.932873
H 0.975582 -2.880589 2.840035
C 1.078619 -0.206347 1.779157
C 1.995177 1.639228 3.037447
H 1.855142 2.588410 3.549718
C 0.700509 -5.482342 0.680489
H 0.928360 -6.544260 0.608116
C -5.310730 2.565501 -1.344359
C -3.392433 0.371882 3.363956
H -3.939742 1.308828 3.447178
O -1.925523 -1.166101 -2.542085
C -2.390046 0.244861 2.410485
H -2.183855 1.072853 1.733557
C 0.094521 -4.827226 -0.383182
H -0.147674 -5.368355 -1.294611
C -1.679390 -0.951615 2.286165
C -3.705069 -0.700390 4.192323
H -4.494586 -0.604939 4.935231
C -5.348105 1.617359 -0.319832
H -6.127177 1.681366 0.438940
C -3.017104 -1.900920 4.055548
H -3.272538 -2.751115 4.684887
C -4.418616 0.585649 -0.259145
H -4.455329 -0.170794 0.520776
C -6.303292 3.687857 -1.397862
H -6.785209 3.746760 -2.381425
H -5.813421 4.654923 -1.222562
H -7.086451 3.568188 -0.642198
H -0.670899 1.307428 0.052404
H -0.294057 2.227150 -0.131941
N 3.149361 1.874588 -0.552061
C 4.265619 0.007572 -1.086991
C 4.392853 1.249358 -0.578742

H 5.047295 -0.710830 -1.300923
H 5.301235 1.761084 -0.285692
C 2.501549 -1.446310 -1.962308
C 1.403689 -1.491316 -2.824019
C 3.179746 -2.638457 -1.680746
C 1.004535 -2.687090 -3.404666
H 0.869049 -0.582213 -3.093900
C 2.794183 -3.827233 -2.285956
H 4.014097 -2.632725 -0.982900
C 1.710904 -3.858615 -3.158109
H 0.130580 -2.684437 -4.052084
H 3.340470 -4.741079 -2.059708
H 1.410456 -4.792502 -3.628874
C 2.986199 3.189823 -0.083026
C 1.992051 4.014927 -0.614415
C 3.822383 3.706809 0.911475
C 1.814784 5.306045 -0.135088
H 1.369183 3.653233 -1.428503
C 3.650548 5.004153 1.376065
H 4.598134 3.077786 1.339860
C 2.639110 5.810217 0.864821
H 1.030871 5.927763 -0.563401
H 4.311225 5.384074 2.153476
H 2.501905 6.824137 1.234363
N 2.933503 -0.226893 -1.413242
B 2.161284 0.950003 -1.069520

$^{Ph}Y_2Ge$
E = -4695.15027900
P 3.009226 0.296843 -0.026345
P -3.056937 0.369290 0.002886
C 1.531530 -0.048197 -0.778879
C 1.228480 -1.383643 -1.281555
C 1.746260 -2.575796 -0.741392
H 2.382656 -2.524297 0.139456
C 1.416535 -3.816383 -1.266564
H 1.831358 -4.714053 -0.810420
C 0.533729 -3.925206 -2.337794
H 0.265246 -4.900823 -2.737920
C 0.004286 -2.762747 -2.890055
H -0.677586 -2.823604 -3.738386
C 0.359513 -1.519839 -2.383434
H -0.008031 -0.613195 -2.867888
C 3.241340 -0.177713 1.734883
C 3.854553 -1.382168 2.090926
H 4.320754 -2.000835 1.326079
C 3.880297 -1.796826 3.417568
H 4.366494 -2.734666 3.679348
C 3.283357 -1.019695 4.404366
H 3.301615 -1.345567 5.442605
C 2.657068 0.174159 4.058123
H 2.181051 0.783327 4.824090
C 2.635653 0.593288 2.733829
H 2.134383 1.522432 2.466842
C 3.345314 2.089045 -0.068134
C 4.120726 2.724951 0.904067
H 4.478642 2.169631 1.768131
C 4.441678 4.071229 0.772836
H 5.043049 4.558742 1.537710
C 3.997069 4.791035 -0.330934

H 4.245611 5.846175 -0.429485
C 3.245034 4.156983 -1.314041
H 2.904892 4.711328 -2.186359
C 2.929016 2.809682 -1.190451
H 2.364864 2.299541 -1.968158
C 4.461288 -0.422199 -0.871330
C 4.329978 -0.934280 -2.162287
H 3.347298 -0.974683 -2.625261
C 5.453041 -1.387802 -2.845845
H 5.342967 -1.797260 -3.848023
C 6.709693 -1.317455 -2.254540
H 7.586358 -1.671836 -2.793741
C 6.847679 -0.786198 -0.975488
H 7.831027 -0.717279 -0.514305
C 5.727677 -0.339101 -0.286410
H 5.837916 0.080683 0.712891
C -1.398715 0.024292 0.217391
C -1.050729 -1.292238 0.790306
C -0.124614 -1.374209 1.843175
H 0.309951 -0.457423 2.230380
C 0.241040 -2.589775 2.402468
H 0.960848 -2.604687 3.219383
C -0.313836 -3.777016 1.931199
H -0.028384 -4.732644 2.367385
C -1.232300 -3.722861 0.889074
H -1.662721 -4.640840 0.491346
C -1.585192 -2.503733 0.322585
H -2.265281 -2.492664 -0.527312
C -4.041121 -0.073748 1.474254
C -4.265808 0.857338 2.490480
H -3.946062 1.889245 2.364620
C -4.905744 0.474483 3.662920
H -5.083377 1.210247 4.444898
C -5.317435 -0.843105 3.836151
H -5.813788 -1.142662 4.757312
C -5.095940 -1.775644 2.828993
H -5.413864 -2.808147 2.959073
C -4.465568 -1.394584 1.650525
H -4.303724 -2.127211 0.863665
C -3.297519 2.147725 -0.289317
C -4.187625 2.630200 -1.249938
H -4.753649 1.937853 -1.867627
C -4.344936 4.000064 -1.432483
H -5.037870 4.365450 -2.187800
C -3.616869 4.897384 -0.660226
H -3.740015 5.968725 -0.807644
C -2.724189 4.423342 0.295364
H -2.141344 5.119391 0.894934
C -2.560861 3.056579 0.478760
H -1.850118 2.694839 1.218180
C -3.975797 -0.445616 -1.362751
C -5.367918 -0.571493 -1.364887
H -5.949888 -0.203174 -0.521080
C -6.008882 -1.173027 -2.441725
H -7.092701 -1.273523 -2.439715
C -5.265860 -1.652724 -3.517671
H -5.770690 -2.129001 -4.356200
C -3.880344 -1.532137 -3.516295
H -3.295227 -1.916034 -4.350034
C -3.237717 -0.929837 -2.440395

H -2.152578 -0.855393 -2.404525
Ge 0.041927 1.258942 -0.734230
H -0.580885 1.044851 1.080673
H 0.166908 1.672196 1.187579

^{Ph}YGeCl
E = -3847.76254297
P 0.613081 0.283900 -0.001168
C -0.864474 -0.398508 -0.518744
C -2.126225 0.318579 -0.276169
C -3.248901 0.075597 -1.091224
H -3.184918 -0.681694 -1.869893
C -4.435609 0.771465 -0.921362
H -5.279200 0.552137 -1.574209
C -4.556497 1.742380 0.069632
H -5.488698 2.288217 0.200280
C -3.467740 1.992496 0.896006
H -3.539677 2.739285 1.685448
C -2.281911 1.286624 0.733852
H -1.455158 1.497576 1.411749
C 1.999405 -0.401505 -0.965293
C 2.310403 0.165486 -2.203592
H 1.773096 1.047093 -2.547472
C 3.307776 -0.389240 -2.994614
H 3.546914 0.060673 -3.955965
C 3.998191 -1.516003 -2.558456
H 4.778514 -1.950827 -3.180027
C 3.689400 -2.087601 -1.329284
H 4.222325 -2.972447 -0.987549
C 2.694223 -1.533390 -0.532121
H 2.452547 -1.989476 0.425932
C 1.071195 -0.055918 1.736119
C 2.359745 0.140168 2.244756
H 3.150952 0.516934 1.597169
C 2.638486 -0.168729 3.569796
H 3.641842 -0.016400 3.962827
C 1.637403 -0.683374 4.390665
H 1.859601 -0.928186 5.427598
C 0.360675 -0.898153 3.884479
H -0.418245 -1.313372 4.520481
C 0.076559 -0.590002 2.557829
H -0.917078 -0.771199 2.145849
C 0.659580 2.085350 -0.249726
C -0.066376 2.605695 -1.326026
H -0.663612 1.934951 -1.941067
C -0.044597 3.969775 -1.584199
H -0.617649 4.369207 -2.418316
C 0.693858 4.822782 -0.769654
H 0.705972 5.892338 -0.970529
C 1.406522 4.310092 0.308399
H 1.975583 4.975933 0.954036
C 1.389207 2.944725 0.571888
H 1.933806 2.557076 1.428826
Ge -0.717815 -2.484664 -0.648874
Cl -2.808729 -2.971558 -0.060920
H -1.140123 -1.955165 -2.426147
H -1.016330 -1.138156 -1.929623

^{Ph}YGeHMDS
E = -4261.54953800

P -1.796582 0.184209 -0.054964
C -0.172164 -0.191168 -0.400991
C 0.214009 -1.573432 -0.009144
C -0.146191 -2.681890 -0.792094
H -0.684550 -2.515158 -1.723696
C 0.165060 -3.979989 -0.403179
H -0.133714 -4.818325 -1.030488
C 0.864890 -4.206628 0.777909
H 1.114517 -5.221007 1.083652
C 1.244860 -3.121626 1.562884
H 1.791312 -3.284404 2.490979
C 0.918938 -1.827638 1.175096
H 1.192725 -0.986275 1.805107
C -3.028167 -0.603746 -1.160951
C -4.303493 -0.971452 -0.727967
H -4.585694 -0.830114 0.313589
C -5.211878 -1.522238 -1.625853
H -6.202212 -1.814826 -1.282160
C -4.855276 -1.701465 -2.958579
H -5.567009 -2.135710 -3.658226
C -3.587530 -1.329601 -3.396744
H -3.306105 -1.469199 -4.438626
C -2.676525 -0.785473 -2.499709
H -1.676661 -0.503149 -2.829013
C -2.137820 1.960518 -0.190631
C -3.146090 2.462681 -1.013777
H -3.759767 1.784371 -1.601651
C -3.365085 3.834682 -1.088207
H -4.149880 4.219737 -1.736093
C -2.585383 4.709286 -0.340792
H -2.758965 5.781911 -0.402678
C -1.580675 4.213322 0.484981
H -0.964740 4.893648 1.069387
C -1.354683 2.845908 0.556965
H -0.567888 2.455848 1.198884
C -2.335310 -0.346719 1.608279
C -2.351628 -1.719474 1.889300
H -2.123404 -2.441236 1.106828
C -2.656890 -2.167492 3.167665
H -2.658976 -3.235718 3.374722
C -2.954998 -1.257025 4.176846
H -3.193694 -1.610729 5.178065
C -2.952975 0.105503 3.901155
H -3.196697 0.822231 4.683005
C -2.644538 0.561146 2.623642
H -2.656334 1.628938 2.419900
Ge 1.237034 1.281012 -0.920869
N 2.835697 0.412963 -0.304453
Si 3.390638 1.002253 1.245530
Si 3.850103 -0.501956 -1.405659
C 1.923956 1.815163 2.122245
H 1.643648 2.755109 1.626137
H 1.037499 1.167363 2.161524
H 2.201842 2.067816 3.153873
C 4.076024 -0.347111 2.366611
H 3.949356 -0.056512 3.418174
H 3.562703 -1.303034 2.210444
H 5.146552 -0.506665 2.195460
C 4.690556 2.353271 1.070083
H 4.994532 2.743699 2.050880

H 5.592853 1.993446 0.560739
H 4.289982 3.188773 0.481107
C 5.497608 -0.951841 -0.609682
H 6.150864 -1.369580 -1.387620
H 6.021472 -0.098181 -0.163607
H 5.372269 -1.719643 0.162794
C 4.213857 0.539224 -2.931232
H 4.837001 -0.008108 -3.650884
H 3.287826 0.830477 -3.443921
H 4.743078 1.459493 -2.650917
C 3.087122 -2.131274 -1.934879
H 2.161522 -2.004360 -2.505871
H 3.801815 -2.675407 -2.568000
H 2.859546 -2.754180 -1.060478
H 1.080083 0.163599 -2.486705
H 0.399199 -0.145848 -1.854863

^{Ph}YGeB1
E = -4063.44202020
P 1.768085 0.766502 0.074123
C 0.180905 0.166017 0.280394
C -0.105157 -0.725043 1.410820
C -1.368195 -0.716161 2.036664
H -2.131616 -0.034017 1.668299
C -1.658510 -1.538560 3.115380
H -2.652997 -1.501279 3.557845
C -0.688705 -2.388830 3.644786
H -0.914001 -3.025808 4.497869
C 0.574866 -2.399319 3.063697
H 1.353145 -3.051152 3.458507
C 0.853950 -1.598090 1.963955
H 1.845532 -1.655697 1.516650
C 1.745943 2.396585 -0.745858
C 1.593782 3.553469 0.020956
H 1.561779 3.487219 1.106353
C 1.488794 4.793505 -0.597312
H 1.373095 5.689969 0.008453
C 1.534066 4.887087 -1.984399
H 1.451986 5.858926 -2.467571
C 1.681966 3.737793 -2.753241
H 1.709550 3.805045 -3.838892
C 1.784895 2.495668 -2.138814
H 1.882702 1.597231 -2.744150
C 2.907647 -0.222154 -0.956569
C 4.087460 0.302024 -1.494511
H 4.347183 1.346000 -1.321593
C 4.920135 -0.502773 -2.261152
H 5.837468 -0.092858 -2.679547
C 4.575155 -1.831190 -2.499134
H 5.227395 -2.461015 -3.101427
C 3.395211 -2.350395 -1.979241
H 3.117482 -3.383555 -2.172159
C 2.556293 -1.547551 -1.212345
H 1.618600 -1.940405 -0.815772
C 2.603814 1.022651 1.673239
C 1.818261 1.429506 2.757312
H 0.744078 1.545189 2.625232
C 2.402160 1.654346 3.996391
H 1.782461 1.963295 4.835648
C 3.770970 1.469220 4.167143

H 4.226761 1.640222 5.140604
C 4.553635 1.055357 3.095689
H 5.622658 0.900066 3.227330
C 3.973823 0.830028 1.851410
H 4.592293 0.489556 1.024864
Ge -0.922097 0.326041 -1.516064
N -2.506331 -2.177817 -0.545406
C -4.447413 -1.511806 0.329876
C -3.639367 -2.578636 0.149299
H -5.438861 -1.481759 0.766192
H -3.809907 -3.614969 0.417941
B -2.568728 -0.758846 -0.769784
N -3.858965 -0.383338 -0.238079
C -4.417441 0.899617 -0.130755
C -5.114664 1.286412 1.016545
C -4.271234 1.814126 -1.177444
C -5.655952 2.563126 1.110097
H -5.211654 0.588432 1.845331
C -4.802522 3.091540 -1.071036
H -3.743232 1.505389 -2.077469
C -5.501164 3.473304 0.070672
H -6.193935 2.850595 2.011705
H -4.677623 3.792103 -1.894582
H -5.922534 4.473319 0.148490
C -1.424779 -3.030420 -0.820803
C -0.872169 -3.059206 -2.103843
C -0.896184 -3.851375 0.176020
C 0.184285 -3.916161 -2.388864
H -1.302755 -2.428944 -2.879994
C 0.158872 -4.705580 -0.116397
H -1.296664 -3.781944 1.185621
C 0.697331 -4.749212 -1.399465
H 0.600100 -3.938040 -3.394583
H 0.570514 -5.334373 0.670755
H 1.516279 -5.429860 -1.625381
H -0.891905 1.382161 0.013343
H -1.491426 1.838910 -0.573337

I
E = -3097.25319055
C 1.658587 2.343910 -0.243367
C -0.470552 1.198805 -0.260738
C -1.111452 2.433032 -0.364686
C -0.374990 3.616803 -0.372889
C 1.008691 3.577185 -0.292823
H 2.748434 2.326928 -0.229299
H -2.195431 2.470714 -0.432308
H -0.895313 4.569981 -0.445105
H 1.590308 4.496916 -0.293037
N -1.121344 -0.035282 -0.192554
C -2.501625 -0.164278 0.007106
C -3.179962 0.556164 1.002061
C -3.226883 -1.091774 -0.751201
C -4.535652 0.356629 1.217343
H -2.624774 1.264206 1.613296
C -4.581147 -1.300892 -0.516874
H -2.712655 -1.640144 -1.539155
C -5.246674 -0.574050 0.462624
H -5.041307 0.925021 1.996488
H -5.121647 -2.029918 -1.118294

H -6.308929 -0.729617 0.639175
C 0.949468 1.140861 -0.248287
P 3.082847 -0.571875 0.262170
C 4.443434 -0.016644 -0.819466
H 4.416387 1.072525 -0.923425
H 5.417949 -0.317297 -0.417056
H 4.300444 -0.458688 -1.811278
C 3.512455 0.105527 1.896886
H 4.562577 -0.074232 2.153755
H 3.315294 1.183263 1.886848
H 2.859749 -0.353994 2.646445
C 3.302478 -2.363770 0.375198
H 4.329092 -2.606367 0.669215
H 2.600821 -2.771327 1.110519
H 3.087063 -2.816810 -0.598802
C 1.509970 -0.215408 -0.274386
H 1.071562 -0.796650 -1.573150
Ge -0.035283 -1.598053 -0.271718
H 0.399256 -1.364305 -2.123550

II
E = -3646.45487748
C 0.119960 3.177921 -0.025447
C -0.014282 1.795259 -0.008039
C -1.161882 1.143666 -0.481625
C -2.208009 1.951122 -0.949852
C -2.087891 3.332290 -0.953997
C -0.923592 3.955538 -0.504823
H 1.041263 3.612883 0.356249
H -3.114106 1.471710 -1.313531
H -2.914088 3.935228 -1.326762
H -0.837114 5.039527 -0.520655
N -1.244219 -0.253469 -0.516190
S 1.224782 0.816132 0.778209
O 2.417010 1.653421 1.033822
O 0.619025 0.183424 1.956510
C 1.593988 -0.359636 -0.433438
C -2.472378 -0.830922 -0.106846
C -2.977673 -0.520084 1.160325
C -3.171313 -1.720934 -0.923727
C -4.171557 -1.079843 1.590519
H -2.398350 0.144540 1.798553
C -4.357336 -2.295066 -0.476355
H -2.780635 -1.954408 -1.912599
C -4.865858 -1.972294 0.776141
H -4.555199 -0.832211 2.578733
H -4.893406 -2.989199 -1.121177
H -5.798262 -2.415794 1.119608
P 3.150835 -1.052572 -0.158116
C 4.504625 0.029263 -0.670047
H 5.479182 -0.397929 -0.408278
H 4.442145 0.189519 -1.751427
H 4.347587 0.982982 -0.154873
C 3.295243 -2.585375 -1.100723
H 3.138022 -2.379065 -2.164879
H 4.289601 -3.021250 -0.955900
H 2.525241 -3.289129 -0.766823
C 3.446617 -1.448873 1.583502
H 3.379460 -0.510772 2.145782
H 2.650024 -2.113613 1.932644

H 4.429373 -1.911496 1.728915
Si 0.029561 -1.353999 -1.158239
H 0.367215 -0.227947 -2.496786
H 1.058435 0.000213 -1.793793

III
E = -4408.53661898
C 0.345561 1.793365 0.464735
C -0.467572 1.731202 -0.669409
C -0.856620 2.912107 -1.300875
C -0.466790 4.147728 -0.802913
C 0.322517 4.211292 0.338395
C 0.725261 3.039699 0.964659
H -1.488011 2.830843 -2.182037
H -0.783471 5.058587 -1.307254
H 0.629223 5.172651 0.745734
H 1.356369 3.065204 1.849108
S 0.940240 0.401860 1.482534
S -1.056431 0.210301 -1.472318
O 2.145095 0.976130 2.120320
O -0.185651 0.037656 2.356314
O 0.121536 -0.291444 -2.228138
O -2.229563 0.681950 -2.230157
C -1.433705 -0.940491 -0.329980
P -2.859977 -0.745994 0.580458
P 2.935602 -0.690606 -0.261105
C -2.801210 -1.820946 2.023817
H -3.717357 -1.713988 2.614428
H -2.688685 -2.861447 1.701634
H -1.922015 -1.521577 2.606373
C -3.106277 0.942185 1.185094
H -3.147768 1.612628 0.319026
H -4.039372 1.016899 1.754573
H -2.245939 1.196893 1.813325
C -4.361998 -1.130688 -0.359931
H -4.338097 -2.185847 -0.652004
H -5.276157 -0.916429 0.205638
H -4.317320 -0.517844 -1.267844
C 3.104698 -1.885760 -1.599593
H 4.067164 -1.739563 -2.101860
H 3.036173 -2.906504 -1.211557
H 2.278905 -1.711646 -2.299859
C 3.096780 0.929949 -1.051044
H 3.057172 1.705128 -0.279448
H 4.045303 0.992919 -1.596062
H 2.249276 1.041364 -1.738125
C 4.365576 -0.874142 0.832049
H 5.304837 -0.666624 0.306891
H 4.217904 -0.170639 1.658162
H 4.376703 -1.894212 1.230447
Ge 0.036943 -2.266879 -0.356038
H 0.015669 -2.641162 1.422608
C 1.415088 -0.926049 0.528959
H 0.772788 -1.962128 1.357093

IV
E = -3616.51971628
C 0.698969 3.305714 0.386395
C 0.198830 2.012982 0.420048
C -0.987850 1.575399 -0.179365

C -1.724089 2.556558 -0.855413
C -1.269680 3.868694 -0.891974
C -0.070772 4.251274 -0.276904
H 1.637503 3.563241 0.873211
H -2.666454 2.294038 -1.334177
H -1.863810 4.624801 -1.403101
H 0.252986 5.289182 -0.314085
B -1.195731 0.014866 0.073634
S 0.997312 0.576128 1.019000
O 1.904340 0.728984 2.159491
O -0.307355 -0.224934 1.339233
C -2.634881 -0.603189 0.252383
C -3.062104 -1.152619 1.467384
C -3.546802 -0.611679 -0.813041
C -4.341454 -1.679409 1.614924
H -2.374009 -1.160855 2.311746
C -4.829598 -1.127774 -0.671285
H -3.240806 -0.213966 -1.782661
C -5.232986 -1.666417 0.547220
H -4.646832 -2.100590 2.572112
H -5.516341 -1.118012 -1.516730
H -6.234776 -2.077180 0.661620
C 1.671026 -0.194415 -0.333581
H 1.160152 0.359926 -1.624707
P 3.172177 -0.982579 -0.054873
C 4.504647 0.135256 0.444889
H 4.651680 0.881146 -0.343074
H 4.190013 0.639023 1.364367
H 5.436090 -0.416066 0.615297
C 3.686931 -1.769392 -1.592561
H 4.637654 -2.294342 -1.448798
H 2.905660 -2.470411 -1.906172
H 3.798844 -1.002877 -2.366507
C 3.097052 -2.271379 1.215544
H 4.065997 -2.762774 1.358455
H 2.779361 -1.788896 2.146911
H 2.342925 -3.009556 0.921551
H 0.491951 0.331431 -2.372762
Ge 0.028497 -1.122843 -1.395605

V
E = -3067.30074352
C -1.739911 2.317663 0.084434
C 0.455527 1.250372 0.162958
C 1.019580 2.530100 0.348848
C 0.245194 3.671277 0.406803
C -1.144990 3.548677 0.268502
H -2.824303 2.278351 -0.008639
H 2.099065 2.606799 0.475712
H 0.696933 4.647943 0.567122
H -1.771227 4.439378 0.315305
B 1.248088 -0.055575 0.120404
C 2.791077 -0.193215 -0.011053
C 3.548096 0.668256 -0.826202
C 3.487861 -1.217012 0.654630
C 4.922903 0.520425 -0.962604
H 3.040015 1.457595 -1.378903
C 4.866005 -1.353461 0.545902
H 2.930748 -1.917992 1.276570
C 5.588398 -0.485568 -0.267844

H 5.480923 1.194538 -1.610825
H 5.379201 -2.147510 1.086163
H 6.666892 -0.597929 -0.366707
C -0.969298 1.126918 0.031839
P -3.171972 -0.543489 -0.173029
C -4.107945 -0.082193 1.323793
H -3.978681 0.986917 1.520763
H -5.175252 -0.309715 1.220800
H -3.691723 -0.638389 2.170575
C -4.109554 0.205158 -1.547281
H -5.171379 -0.060950 -1.490536
H -4.006942 1.294055 -1.530445
H -3.687617 -0.162871 -2.488539
C -3.446598 -2.319344 -0.363617
H -4.520502 -2.526379 -0.427597
H -2.946034 -2.668652 -1.272770
H -3.007807 -2.848522 0.488016
Ge -0.093145 -1.687170 0.256326
H 0.023158 -1.550868 -1.604873
C -1.497534 -0.199667 -0.146380
H -0.668575 -0.986445 -1.366584

Pathway C

IV
E = -3616.52695199
C 1.028057 3.068613 0.270755
C 0.354721 1.862604 0.366496
C -0.888279 1.586269 -0.209805
C -1.479927 2.619443 -0.940227
C -0.836040 3.848580 -1.053133
C 0.405014 4.079251 -0.454367
H 1.987009 3.223076 0.762259
H -2.455312 2.472688 -1.401948
H -1.314316 4.654076 -1.607964
H 0.878417 5.054847 -0.544082
B -1.309747 0.081226 0.146608
S 0.935418 0.366913 1.107925
O 1.798626 0.611240 2.265495
O -0.505802 -0.193313 1.411223
C 1.516796 -0.669760 -0.050780
C -2.804715 -0.385424 0.218202
C -3.346978 -0.910558 1.396887
C -3.653237 -0.270633 -0.891550
C -4.679923 -1.301232 1.466849
H -2.706605 -1.011122 2.272141
C -4.986640 -0.657110 -0.828268
H -3.258398 0.114892 -1.832930
C -5.506220 -1.174461 0.354916
H -5.078095 -1.706567 2.396130
H -5.623574 -0.559516 -1.706317
H -6.549770 -1.480254 0.408039
P 3.207666 -0.863658 -0.115001
C 4.133773 0.696121 -0.172617
H 5.212283 0.521480 -0.256739
H 3.779924 1.290598 -1.021716
H 3.923195 1.237182 0.757196
C 3.624469 -1.793286 -1.602816
H 3.276201 -1.235983 -2.478443
H 4.705721 -1.957367 -1.661553

H 3.104122 -2.756835 -1.577490
C 3.905696 -1.759915 1.296656
H 3.595588 -1.216896 2.196563
H 3.469710 -2.764025 1.325837
H 4.998642 -1.824211 1.246224
Ge 0.028615 -1.603702 -1.081475
H 0.077973 -0.391454 -2.329860
H -0.533346 -0.042466 -1.489919

V
E = -3067.31179563
C 1.720620 2.316421 -0.026872
C -0.468500 1.235143 0.077970
C -1.042627 2.517969 0.206329
C -0.277933 3.665921 0.220737
C 1.114952 3.548844 0.094279
H 2.804498 2.280264 -0.127988
H -2.123972 2.592546 0.314900
H -0.738939 4.645109 0.330974
H 1.732706 4.446681 0.095979
B -1.264456 -0.067186 0.056267
C -2.815266 -0.221540 0.070029
C -3.456809 -1.202756 0.843976
C -3.632953 0.589866 -0.735614
C -4.837788 -1.349813 0.839612
H -2.857926 -1.863737 1.470818
C -5.014198 0.433762 -0.762386
H -3.171869 1.346670 -1.369066
C -5.623107 -0.531651 0.032534
H -5.305119 -2.111870 1.461389
H -5.619730 1.071181 -1.405024
H -6.705245 -0.650344 0.019566
C 0.963053 1.114513 -0.016802
C 1.515529 -0.208952 -0.077700
P 3.181004 -0.542187 0.053071
C 4.259716 0.050751 -1.297137
H 4.184101 1.138582 -1.389298
H 5.306136 -0.226431 -1.123582
H 3.908775 -0.395897 -2.233568
C 3.968584 0.096674 1.568022
H 5.045510 -0.106267 1.579333
H 3.798296 1.175532 1.640896
H 3.490480 -0.382755 2.428619
C 3.475815 -2.326174 0.092752
H 4.547587 -2.523840 0.201391
H 2.926348 -2.764610 0.931770
H 3.108483 -2.779919 -0.833420
Ge 0.181869 -1.611565 -0.388721
H -0.581942 -1.222045 1.225791
H -0.055285 -2.003906 1.363797

5.2.4 H_2 activated products
Pathway A

$^{Tos}Y_2Ge$
E = -5872.42401869
C 4.647581 2.195040 0.442141
H 4.554098 2.112197 1.522219
C 3.565070 1.854431 -0.351951
C -3.593929 -1.846227 -0.360378

O 1.023532 2.301553 0.075003
C -3.673369 -1.919974 -1.747746
H -2.805715 -1.658315 -2.351619
C 2.490497 -3.363800 2.369697
H 3.376084 -3.788539 1.897434
C 5.830240 2.607944 -0.163462
H 6.687014 2.867393 0.457428
C -1.188119 5.002996 -1.757141
H -0.702780 5.943540 -1.505319
C -1.432941 4.069225 -0.756421
H -1.113133 4.266774 0.262888
C 2.361575 -2.558361 -2.412720
H 2.720926 -1.563069 -2.669209
C 2.070232 -2.865416 -1.079359
C 5.011406 -1.347346 -0.689388
H 4.640288 -1.573481 -1.684754
C 6.357825 -1.055645 -0.516715
H 7.022777 -1.053123 -1.378051
C -1.541227 4.729649 -3.072973
H -1.346601 5.463692 -3.852771
C -1.603829 0.143968 -0.356086
O -2.404590 -1.086792 1.825290
S -2.068102 -1.304966 0.402588
P -2.340110 1.589880 0.187858
C -4.863910 -2.310419 -2.338535
H -4.932117 -2.364310 -3.424688
C -4.637147 1.056755 1.677809
H -3.958674 1.003463 2.524908
C -0.707730 1.711370 2.406786
H -0.198807 0.882993 1.932382
C 5.994069 -0.763706 1.844213
H 6.372104 -0.526293 2.836680
C 4.643662 -1.048085 1.675722
H 3.967606 -1.007321 2.525441
C -2.356150 2.566334 -2.405477
H -2.748648 1.584524 -2.664917
C 1.817490 -2.292243 1.773056
C 3.642236 1.938267 -1.738814
H 2.776710 1.671710 -2.343613
C 1.510412 -4.102162 -0.762960
H 1.215586 -4.320768 0.259533
C 4.146031 -1.356979 0.407178
C -2.007184 3.873296 3.571728
H -2.517368 4.717669 4.031404
C -1.807886 2.277033 1.778482
C -2.467113 3.362310 2.365278
H -3.339246 3.802463 1.882204
C -2.041536 2.855935 -1.073552
C -2.118900 3.505696 -3.398595
H -2.367592 3.274788 -4.432479
C 7.234075 3.080368 -2.202471
H 7.065752 3.773332 -3.035330
H 7.754678 2.204059 -2.613252
H 7.911686 3.561459 -1.489359
C -0.892303 3.307974 4.190590
H -0.531764 3.718898 5.132092
C -5.983031 -2.638719 -1.562493
C 6.852896 -0.769830 0.751902
H 7.908937 -0.542775 0.886113
C -6.358834 1.095127 -0.507923

H -7.026756 1.104881 -1.366910
C 1.597557 -0.159162 -0.356576
P 2.348844 -1.597946 0.185533
O 2.386778 1.069828 1.830307
S 2.046489 1.290899 0.409100
O -1.057009 -2.325157 0.063613
C 5.944301 2.680420 -1.550831
C 4.828047 2.345339 -2.328023
H 4.894632 2.407286 -3.413838
C 2.145699 -3.499692 -3.408914
H 2.375592 -3.254613 -4.443881
C 1.613917 -4.744582 -3.084748
H 1.435817 -5.480299 -3.866890
C 1.286800 -5.038175 -1.766587
H 0.839369 -5.997348 -1.514832
C -5.009945 1.373319 -0.683920
H -4.641123 1.601629 -1.679440
C -0.240214 2.228904 3.607637
H 0.646142 1.791167 4.060777
C -4.140227 1.367345 0.409224
C -6.852581 0.807417 0.760698
H -7.910529 0.590979 0.897414
C -5.867003 -2.576425 -0.174858
H -6.726067 -2.830489 0.445088
C -5.989895 0.786017 1.849729
H -6.366754 0.547171 2.842299
C 0.895622 -3.334222 4.178284
H 0.532986 -3.749480 5.117072
C 2.027366 -3.880139 3.572679
H 2.548471 -4.713497 4.040144
C -4.679347 -2.180367 0.432380
H -4.584141 -2.105774 1.512899
C -7.277091 -3.021601 -2.215888
H -7.965589 -3.484989 -1.501520
H -7.117494 -3.724598 -3.042125
H -7.780701 -2.140089 -2.636477
C 0.230012 -2.268601 3.585974
H -0.668740 -1.845452 4.028505
C 0.700800 -1.745339 2.388857
H 0.183288 -0.925772 1.908613
Ge -0.004107 -0.011131 -1.427649
H -0.149163 -1.255807 -2.314942
H 0.141602 1.229348 -2.321392

^{Tos}YGeCl
E = -4436.42158259
C -3.271691 0.314575 -1.434159
H -2.934033 -0.090930 -2.384851
C -2.394460 1.076570 -0.679059
O -0.652343 2.829808 -1.559528
C 2.178345 -2.681515 -1.097330
H 1.683468 -3.365205 -0.407874
C -4.551565 0.071285 -0.947252
H -5.241430 -0.532644 -1.535517
C 0.825623 -0.233541 2.895124
H -0.044999 0.396275 2.719155
C 1.451046 -0.860532 1.811119
C -1.621131 -1.799403 1.116452
H -1.399990 -1.299926 2.055899
C -2.787693 -2.542215 0.996998

H -3.468970 -2.615391 1.842080
C -2.215102 -3.078908 -1.280338
H -2.450261 -3.569089 -2.222833
C -1.049541 -2.329385 -1.170326
H -0.393033 -2.208118 -2.027705
C 1.897930 -1.310964 -1.052126
C -2.786778 1.613765 0.542476
H -2.086406 2.218607 1.115916
C 2.604357 -1.615246 2.027261
H 3.121755 -2.078544 1.191626
C -0.740335 -1.699172 0.037052
C -6.327213 0.269665 0.829780
H -6.769038 1.143756 1.321889
H -6.279278 -0.532672 1.579386
H -7.009911 -0.059885 0.039607
C -3.081826 -3.189076 -0.199468
H -3.995710 -3.772961 -0.291470
C 0.332367 1.024181 -0.015264
P 0.732003 -0.636118 0.158295
O -0.564243 0.436024 -2.410868
S -0.746260 1.390453 -1.303520
C -4.963980 0.575283 0.285887
C -4.062323 1.356525 1.019199
H -4.371097 1.771268 1.978176
C 1.331480 -0.385920 4.178359
H 0.839602 0.106250 5.014751
C 2.474252 -1.151690 4.390091
H 2.875409 -1.264617 5.395348
C 3.111548 -1.758331 3.314585
H 4.015978 -2.341835 3.473253
C 3.688284 -2.298861 -2.932263
H 4.390589 -2.684936 -3.668754
C 3.076582 -3.171929 -2.034466
H 3.295910 -4.237123 -2.071006
C 3.395649 -0.940962 -2.897880
H 3.862711 -0.260542 -3.606447
C 2.495730 -0.441551 -1.961956
H 2.249221 0.617571 -1.941363
Cl 2.958359 3.017824 -0.813763
Ge 1.368349 2.493344 0.605829
H 2.135053 2.108020 1.877256
H 0.530681 3.754655 0.790993

^{Tos}YGeHMDS
E = -4850.22337441
S -0.839816 -1.537662 -0.787306
P -0.714099 1.232818 -0.065870
Si 3.999319 0.322069 0.544701
Si 3.367663 -2.513503 -0.427688
O -0.031864 -2.757441 -0.643909
O -1.028617 -0.974772 -2.137855
N 2.904124 -1.049952 0.456049
C -0.215138 -0.365585 0.290068
C -2.483153 -1.952235 -0.213809
C -2.688399 -2.248539 1.130049
H -1.848359 -2.223391 1.822438
C -3.965936 -2.554864 1.569620
H -4.131406 -2.782177 2.622232
C -5.049405 -2.579149 0.682227
C -4.809327 -2.300588 -0.662637

H -5.638944 -2.319054 -1.368376
C -3.532786 -1.985980 -1.117478
H -3.339429 -1.745974 -2.160246
C -6.432884 -2.876272 1.177586
H -6.440898 -3.743051 1.849115
H -7.120243 -3.079660 0.349952
H -6.838284 -2.026778 1.744446
C -2.494374 1.331861 -0.447229
C -3.424682 1.283286 0.593923
H -3.090137 1.251148 1.627919
C -4.784448 1.276307 0.314464
H -5.502463 1.237151 1.130952
C -5.224196 1.320172 -1.005093
H -6.290489 1.317787 -1.223363
C -4.301276 1.353481 -2.043885
H -4.641009 1.369475 -3.077498
C -2.938280 1.349813 -1.770947
H -2.217554 1.329193 -2.583666
C 0.097261 2.002211 -1.497070
C -0.332709 3.230684 -2.008876
H -1.162377 3.758773 -1.539780
C 0.279732 3.765557 -3.134232
H -0.054363 4.721993 -3.531285
C 1.309165 3.067737 -3.761612
H 1.781558 3.481884 -4.650354
C 1.724464 1.837882 -3.264175
H 2.515348 1.282028 -3.764042
C 1.122078 1.301623 -2.131362
H 1.430968 0.332662 -1.740582
C -0.435696 2.278642 1.392292
C -0.726012 1.746754 2.652423
H -1.072116 0.717554 2.730145
C -0.545792 2.516072 3.794584
H -0.768380 2.091795 4.771363
C -0.068460 3.818373 3.688965
H 0.080086 4.418258 4.584725
C 0.230223 4.348700 2.438863
H 0.618220 5.361423 2.352817
C 0.048315 3.583784 1.293121
H 0.313758 3.999059 0.324750
C 4.803843 0.681034 -1.119699
H 5.527816 1.498456 -1.000909
H 4.045493 1.014275 -1.837903
H 5.332254 -0.169797 -1.560869
C 3.089878 1.907342 0.986210
H 3.804203 2.740124 0.929086
H 2.650764 1.912852 1.990042
H 2.294079 2.116576 0.262485
C 5.323183 0.048201 1.849794
H 5.960028 -0.812069 1.615588
H 4.854668 -0.138284 2.825043
H 5.967927 0.931779 1.947977
C 2.894128 -2.322086 -2.233593
H 3.358147 -1.425026 -2.665113
H 1.805373 -2.227543 -2.320692
H 3.209329 -3.187458 -2.831141
C 2.627182 -4.061700 0.321986
H 3.059913 -4.932455 -0.189510
H 1.540769 -4.103125 0.213129
H 2.886825 -4.137872 1.386095

C 5.230803 -2.793260 -0.288269
H 5.497637 -3.034731 0.748889
H 5.863590 -1.961072 -0.613115
H 5.492608 -3.664324 -0.904395
Ge 1.282657 -0.965705 1.357718
H 0.943753 -2.348646 1.920273
H 1.478281 0.022992 2.517710

$^{Tos}YGeC_6F_5$
E = -4704.76238422
C 3.381257 -1.953868 -1.450065
H 3.001751 -1.711843 -2.439676
C 2.501892 -1.975338 -0.379818
O -0.006441 -2.786111 -0.321893
C -0.196586 3.248594 -1.730127
H 0.614374 3.852950 -1.323802
C 4.729399 -2.216124 -1.226301
H 5.425410 -2.190207 -2.063822
C 1.173029 1.900677 2.649372
H 1.791614 1.005303 2.650347
C 0.481905 2.265196 1.487941
C 3.472254 1.378308 0.191027
H 3.341463 1.339422 1.268275
C 4.756508 1.398192 -0.334831
H 5.613143 1.375816 0.335454
C 3.845822 1.453513 -2.562780
H 3.987636 1.470019 -3.641432
C 2.556963 1.429509 -2.041819
H 1.700172 1.397023 -2.709315
C -0.387674 1.937484 -1.282980
C 2.948164 -2.271019 0.904943
H 2.241675 -2.285914 1.733259
C -0.398175 3.346961 1.526146
H -0.991804 3.601026 0.652468
C 2.363482 1.407645 -0.658893
C 6.663109 -2.763409 0.297279
H 6.817511 -3.770485 0.705576
H 7.080326 -2.055876 1.025421
H 7.246839 -2.682031 -0.625271
C 4.944980 1.441935 -1.712953
H 5.952615 1.456198 -2.124060
C 0.358407 -0.380886 0.482967
P 0.676515 1.232058 0.007829
O 0.725985 -1.053594 -2.028951
S 0.777267 -1.590173 -0.656326
C 5.208458 -2.495841 0.052224
C 4.293997 -2.522968 1.113578
H 4.648259 -2.747415 2.119185
C 1.027026 2.642189 3.812772
H 1.569170 2.351332 4.710108
C 0.170896 3.739423 3.836353
H 0.050073 4.316288 4.751177
C -0.548596 4.080954 2.698110
H -1.243016 4.918142 2.720618
C -2.071135 3.002564 -3.221624
H -2.735133 3.421760 -3.975145
C -1.040687 3.779279 -2.696545
H -0.895177 4.800758 -3.042187
C -2.245740 1.691129 -2.795366
H -3.040963 1.079243 -3.216083

C -1.397850 1.153754 -1.833703
H -1.498200 0.119125 -1.516828
Ge -0.883096 -0.968870 1.822174
H -1.043819 0.098069 2.912796
H -0.473783 -2.309054 2.427667
C -2.641760 -1.142913 0.960005
C -4.359297 -2.404340 -0.197818
C -4.401434 0.005211 -0.268771
C -4.949389 -1.221298 -0.627353
C -3.270037 0.016521 0.532483
C -3.215562 -2.351646 0.593073
F -2.684564 -3.498272 0.981953
F -2.745491 1.204882 0.851043
F -4.896175 -3.562920 -0.541483
F -6.030854 -1.261614 -1.385194
F -4.949933 1.130969 -0.701089

^{Tos}YGePy
E = -4223.90829333
C 3.584596 -0.795591 -1.518052
H 3.233104 -0.459307 -2.490406
C 2.682700 -1.383166 -0.645495
O 0.725986 -3.010935 -1.309488
C -1.614095 2.776917 -1.137090
H -1.096436 3.462816 -0.466893
C 4.905536 -0.623055 -1.116858
H 5.613603 -0.150528 -1.796653
C -0.054568 0.666177 2.901345
H 0.765774 -0.028004 2.725622
C -0.754424 1.191878 1.810485
C 2.328955 1.776119 0.798372
H 2.148823 1.410944 1.805239
C 3.537948 2.392852 0.506037
H 4.289646 2.500058 1.285312
C 2.825103 2.715427 -1.770696
H 3.019505 3.072208 -2.780127
C 1.614850 2.094276 -1.485191
H 0.880601 1.941011 -2.271159
C -1.363769 1.403304 -1.065397
C 3.089319 -1.813426 0.613790
H 2.365567 -2.269963 1.286717
C -1.870088 1.997251 2.036104
H -2.462236 2.357491 1.200096
C 1.355556 1.631876 -0.193111
C 6.752487 -0.814608 0.589620
H 7.210649 -1.753268 0.925593
H 6.801765 -0.114085 1.433971
H 7.369022 -0.407903 -0.218564
C 3.785718 2.867964 -0.777942
H 4.734080 3.350919 -1.006145
C 0.026356 -0.974504 0.137715
P -0.191403 0.726353 0.147510
O 0.839646 -0.701590 -2.337507
S 0.975082 -1.576015 -1.155031
C 5.336228 -1.027357 0.145678
C 4.406652 -1.630960 1.002301
H 4.725373 -1.960219 1.990913
C -0.435285 0.985643 4.197585
H 0.114533 0.570669 5.039764
C -1.529958 1.815719 4.415826

H -1.833511 2.061229 5.431734
C -2.250629 2.310889 3.335301
H -3.125700 2.935459 3.502722
C -3.153325 2.395261 -2.948773
H -3.857926 2.782570 -3.682542
C -2.514152 3.269710 -2.072981
H -2.712248 4.338505 -2.124967
C -2.879714 1.032994 -2.899241
H -3.363037 0.350022 -3.594648
C -1.980843 0.535322 -1.963150
H -1.735454 -0.523057 -1.933538
C -2.986934 -1.757567 0.285476
N -3.470164 -0.580826 0.705397
C -4.833963 -2.041624 -1.223554
C -4.607592 -0.133390 0.190363
C -5.330066 -0.822626 -0.778645
H -5.364714 -2.612455 -1.984252
H -4.963044 0.832304 0.558045
H -6.254735 -0.408304 -1.174592
C -3.645818 -2.519062 -0.682025
H -3.221777 -3.465130 -1.013749
Ge -1.209637 -2.167596 1.014026
H -1.283376 -1.878210 2.522127
H -0.814328 -3.621653 0.760786

^{Tos}YGePyr
E = -4239.96535233
C 3.600325 -0.676343 -1.562231
H 3.257313 -0.297511 -2.521975
C 2.693171 -1.311253 -0.728893
O 0.796158 -2.944702 -1.517201
C -1.660681 2.808177 -0.929196
H -1.153709 3.449172 -0.208534
C 4.915904 -0.516261 -1.139511
H 5.628550 -0.009377 -1.789137
C -0.013467 0.376146 2.910765
H 0.789024 -0.313743 2.653111
C -0.723442 1.012966 1.887556
C 2.306987 1.734487 0.892719
H 2.131862 1.312929 1.878465
C 3.502840 2.391471 0.635918
H 4.251209 2.473501 1.421428
C 2.785020 2.820492 -1.622245
H 2.972803 3.235671 -2.610339
C 1.588372 2.159214 -1.373395
H 0.858068 2.032352 -2.167832
C -1.387345 1.437457 -0.963794
C 3.089769 -1.800339 0.511725
H 2.362856 -2.297526 1.151793
C -1.808041 1.827514 2.210251
H -2.406951 2.278171 1.424437
C 1.339327 1.622069 -0.108368
C 6.745093 -0.773467 0.576875
H 7.172329 -1.699976 0.979208
H 6.787457 -0.025176 1.380212
H 7.391915 -0.425195 -0.234998
C 3.741220 2.939668 -0.620377
H 4.679023 3.454723 -0.820220
C 0.016024 -1.014366 0.034140
P -0.194509 0.680509 0.181844

O 0.856644 -0.575486 -2.407403
S 0.998529 -1.516055 -1.277507
C 5.336350 -0.979237 0.106564
C 4.402054 -1.629117 0.922857
H 4.713399 -2.006711 1.896404
C -0.356944 0.589120 4.239005
H 0.199986 0.087801 5.027820
C -1.422700 1.424802 4.555741
H -1.697293 1.586795 5.596327
C -2.150924 2.034871 3.540751
H -3.002191 2.667340 3.784129
C -3.186972 2.545243 -2.773029
H -3.893977 2.977842 -3.478619
C -2.566989 3.357819 -1.826860
H -2.782742 4.424017 -1.795540
C -2.888283 1.188573 -2.831080
H -3.351908 0.556520 -3.585629
C -1.985359 0.632642 -1.931594
H -1.719870 -0.420406 -1.988638
Ge -1.244411 -2.257879 0.798607
H -1.263825 -2.146106 2.334086
H -0.938936 -3.679736 0.347260
C -3.027462 -1.683251 0.178576
N -3.607114 -2.330071 -0.835215
N -3.514192 -0.576859 0.753311
C -4.747222 -1.834279 -1.301473
C -4.653008 -0.089160 0.280119
C -5.331001 -0.690336 -0.771984
H -5.212681 -2.368639 -2.133105
H -5.036466 0.818970 0.751924
H -6.261896 -0.286260 -1.162050

^{Tos}YGeB2
E = -4382.92514797
C 1.399389 -3.083104 -1.266606
H 1.052868 -2.782471 -2.252349
C 0.846118 -2.481937 -0.148184
O -1.648006 -1.796696 0.228640
C 1.343902 3.128408 -2.113908
H 2.422678 3.096871 -1.962338
C 2.398742 -4.036815 -1.099362
H 2.842408 -4.506225 -1.976524
C 2.115617 1.551035 2.472723
H 1.881548 0.493489 2.586521
C 1.847087 2.185929 1.254805
C 3.542027 -0.260433 -0.118977
H 3.548517 -0.110300 0.958273
C 4.537969 -1.022870 -0.713020
H 5.319373 -1.464041 -0.097785
C 3.522388 -0.672697 -2.869955
H 3.505713 -0.843251 -3.944483
C 2.517579 0.086033 -2.280199
H 1.703737 0.481320 -2.882507
C 0.503301 2.304079 -1.358032
C 1.267431 -2.828263 1.132320
H 0.820903 -2.342281 1.998408
C 2.099536 3.551387 1.120613
H 1.863929 4.064968 0.192552
C 2.534786 0.308774 -0.902116
C 3.950303 -5.397900 0.348682

H 3.655100 -6.190718 1.047025
H 4.853239 -4.927750 0.761001
H 4.222913 -5.867657 -0.601893
C 4.532946 -1.223150 -2.090542
H 5.314789 -1.820800 -2.555459
C 0.038270 0.101129 0.605579
P 1.158486 1.180411 -0.092205
O -0.395344 -0.896947 -1.793659
S -0.412871 -1.229520 -0.359168
C 2.852769 -4.392381 0.169513
C 2.266453 -3.775909 1.282767
H 2.602676 -4.046830 2.283076
C 2.649754 2.271670 3.532577
H 2.851913 1.771325 4.477311
C 2.910750 3.630992 3.389918
H 3.322837 4.196802 4.223202
C 2.631078 4.268581 2.186850
H 2.818337 5.334743 2.076963
C -0.572627 3.990000 -3.288716
H -0.994516 4.650456 -4.044087
C 0.803970 3.973646 -3.073789
H 1.457280 4.615277 -3.661768
C -1.404794 3.155265 -2.552477
H -2.477987 3.153534 -2.731416
C -0.871090 2.305552 -1.588888
H -1.515414 1.625982 -1.034601
C -4.570114 -0.126226 -0.304534
C -4.926220 -0.962887 0.750662
C -5.362016 0.012984 -1.425953
C -6.095348 -1.696496 0.734191
C -6.546086 -0.728066 -1.453846
H -5.067201 0.663709 -2.244905
C -6.904896 -1.563289 -0.396767
H -6.360812 -2.346844 1.563207
H -7.199221 -0.653428 -2.320757
H -7.833516 -2.127309 -0.453974
B -2.984469 -0.072220 1.205847
O -3.385705 0.477322 -0.008596
O -3.967047 -0.898688 1.715921
Ge -1.204305 0.498395 2.039744
H -1.090213 2.004211 2.348343
H -0.818129 -0.262356 3.327247

^{Tos}YGeB1
E = -4652.10228587
C -4.161775 -1.042388 -1.042300
H -4.122630 -1.914020 -0.393363
C -2.974930 -0.514691 -1.523256
O -0.849877 -1.752209 -2.363566
C -0.303610 0.488461 3.730759
H -1.226420 1.051929 3.862779
C -5.363958 -0.430268 -1.380555
H -6.298243 -0.836832 -0.994681
C -0.684572 3.131343 -0.394107
H -1.133978 2.542676 -1.192189
C -0.347013 2.499676 0.806673
C -3.396580 1.632663 1.071449
H -3.050976 2.515227 0.538946
C -4.739558 1.509374 1.403582
H -5.437283 2.296736 1.125808

C -4.295184 -0.626292 2.428447
H -4.646332 -1.513573 2.951253
C -2.950990 -0.513035 2.096827
H -2.257054 -1.315099 2.335657
C 0.140661 0.162631 2.445041
C -2.978118 0.592024 -2.366059
H -2.034885 0.964977 -2.763041
C 0.266556 3.231001 1.822737
H 0.556138 2.745187 2.751049
C -2.497330 0.624061 1.422709
C -6.688276 1.394038 -2.506230
H -6.707182 1.762670 -3.538292
H -6.839479 2.263339 -1.850507
H -7.544370 0.725604 -2.365783
C -5.188323 0.383376 2.085739
H -6.240985 0.289565 2.346350
C -0.391124 -0.049350 -0.518289
P -0.744725 0.729853 0.957777
O -1.757432 -2.270668 -0.044891
S -1.417317 -1.289887 -1.087042
C -5.394916 0.704169 -2.190864
C -4.182253 1.199163 -2.686923
H -4.188146 2.070192 -3.341576
C -0.427655 4.484091 -0.567030
H -0.691207 4.968919 -1.504777
C 0.181411 5.212404 0.451069
H 0.393326 6.270759 0.311538
C 0.530354 4.584880 1.641063
H 1.018979 5.148720 2.433075
C 1.604008 -0.644662 4.672764
H 2.174829 -0.960060 5.544191
C 0.427897 0.083413 4.839892
H 0.077925 0.333133 5.839654
C 2.042839 -0.975593 3.396141
H 2.956757 -1.550074 3.255784
C 1.307865 -0.578618 2.283624
H 1.630959 -0.856664 1.284032
N 4.130955 -0.593319 -0.651889
C 3.972273 -2.630786 0.246164
C 4.796223 -1.573688 0.074134
H 4.180749 -3.588752 0.708802
H 5.836891 -1.469975 0.357901
B 2.790618 -1.055260 -0.933979
C 1.693963 -3.316537 -0.298945
C 1.058937 -3.724365 -1.466855
C 1.313801 -3.867827 0.926159
C 0.047850 -4.674406 -1.410427
H 1.349949 -3.280270 -2.414457
C 0.308937 -4.823724 0.974002
H 1.803005 -3.535312 1.839328
C -0.325008 -5.231424 -0.194694
H -0.458842 -4.965682 -2.327468
H 0.011229 -5.242510 1.933421
H -1.121774 -5.971177 -0.153639
C 4.707902 0.652276 -0.952112
C 4.452393 1.259605 -2.183300
C 5.533720 1.300257 -0.030793
C 4.998843 2.501367 -2.477995
H 3.834032 0.739196 -2.912354
C 6.090925 2.534674 -0.339253

H 5.716593 0.841764 0.938607
C 5.823747 3.144044 -1.560717
H 4.787247 2.963366 -3.440361
H 6.730122 3.030096 0.389335
H 6.257673 4.113263 -1.797218
N 2.747838 -2.371415 -0.356465
Ge 1.266102 0.212439 -1.501246
H 1.749948 1.615361 -1.060337
H 0.960337 0.304117 -3.005161

$^{Ph}Y_2Ge$
E = -4695.22357507
P -3.018558 -0.282921 -0.057149
P 3.079889 -0.373285 -0.022236
C -1.516421 0.044425 -0.773437
C -1.215335 1.384737 -1.279794
C -1.771290 2.562257 -0.749795
H -2.432492 2.495462 0.111470
C -1.446971 3.813526 -1.255832
H -1.889493 4.701027 -0.806064
C -0.536379 3.941792 -2.299375
H -0.268876 4.923801 -2.684277
C 0.027356 2.790168 -2.842465
H 0.733600 2.868616 -3.669110
C -0.313295 1.538333 -2.349508
H 0.107965 0.640436 -2.801760
C -3.329199 0.220517 1.687342
C -3.965064 1.420098 2.016152
H -4.406982 2.033528 1.232777
C -4.044673 1.837142 3.340584
H -4.548543 2.771154 3.581716
C -3.479688 1.067188 4.350943
H -3.541181 1.394392 5.387060
C -2.825481 -0.119725 4.031228
H -2.370919 -0.721439 4.815848
C -2.749252 -0.539007 2.709500
H -2.228215 -1.464083 2.462237
C -3.357185 -2.074479 -0.064448
C -4.244367 -2.657330 0.842860
H -4.691075 -2.057276 1.633064
C -4.556626 -4.008379 0.745141
H -5.244851 -4.455862 1.459607
C -3.990206 -4.784790 -0.260126
H -4.232666 -5.843439 -0.332620
C -3.119597 -4.204219 -1.176705
H -2.679874 -4.805163 -1.970213
C -2.810513 -2.852741 -1.086769
H -2.143496 -2.386915 -1.809712
C -4.418122 0.421123 -0.992984
C -4.220884 0.862101 -2.302175
H -3.219084 0.858548 -2.723302
C -5.302683 1.300790 -3.056920
H -5.140061 1.654694 -4.072959
C -6.585092 1.286126 -2.519255
H -7.429780 1.629318 -3.113840
C -6.789502 0.823742 -1.222806
H -7.792990 0.797252 -0.802509
C -5.710375 0.390631 -0.462421
H -5.874992 0.023270 0.549635
C 1.455480 0.023848 0.226270

C 1.092269 1.287910 0.882453
C 0.113612 1.287426 1.890174
H -0.302944 0.332595 2.202684
C -0.316847 2.459328 2.493819
H -1.087638 2.411515 3.261747
C 0.242623 3.681688 2.127693
H -0.088295 4.604520 2.600762
C 1.227391 3.707106 1.145858
H 1.663658 4.655661 0.835853
C 1.634147 2.532504 0.523082
H 2.353496 2.582580 -0.293692
C 4.127304 0.062500 1.411553
C 4.315640 -0.836584 2.463326
H 3.921586 -1.847504 2.394224
C 5.015596 -0.451165 3.599670
H 5.165405 -1.164241 4.408063
C 5.521756 0.840716 3.704292
H 6.064167 1.143400 4.598053
C 5.335083 1.742653 2.663416
H 5.726147 2.755271 2.739482
C 4.647376 1.355586 1.518895
H 4.518413 2.062774 0.703123
C 3.229602 -2.165922 -0.303788
C 3.981049 -2.705867 -1.348286
H 4.511188 -2.051250 -2.035050
C 4.039131 -4.083933 -1.527230
H 4.621518 -4.495109 -2.349452
C 3.349876 -4.932010 -0.668078
H 3.395024 -6.009703 -0.813881
C 2.595332 -4.400303 0.372815
H 2.044687 -5.057796 1.042517
C 2.532314 -3.024956 0.553431
H 1.923310 -2.603015 1.350735
C 4.014049 0.394119 -1.407919
C 5.409567 0.351590 -1.487050
H 5.984688 -0.134568 -0.699380
C 6.061794 0.936213 -2.565264
H 7.148115 0.902665 -2.625893
C 5.326622 1.572530 -3.563891
H 5.841071 2.035468 -4.404123
C 3.940026 1.625575 -3.481841
H 3.364298 2.133223 -4.253665
C 3.285103 1.036511 -2.404389
H 2.202467 1.086765 -2.304305
Ge -0.000500 -1.094981 -0.369578
H -0.467794 -2.126227 0.701232
H 0.457189 -1.958384 -1.576480

^{Ph}*YGeCl*
E = -3847.83356599
P -0.574342 0.325892 0.058623
C 0.707415 -0.301704 -0.883140
C 1.986918 0.418149 -0.901391
C 2.072279 1.810948 -0.702375
H 1.165618 2.393414 -0.545641
C 3.288925 2.479696 -0.699471
H 3.302389 3.556663 -0.537549
C 4.477556 1.789676 -0.906662
H 5.431526 2.312894 -0.909590
C 4.421674 0.412366 -1.098323

H 5.339859 -0.155041 -1.243720
C 3.208258 -0.260170 -1.085696
H 3.213348 -1.344453 -1.183084
C 0.067167 0.944223 1.651647
C -0.384306 2.093890 2.299483
H -1.161403 2.710084 1.854753
C 0.179610 2.473192 3.513070
H -0.173105 3.373559 4.012443
C 1.195456 1.712302 4.080981
H 1.636352 2.015039 5.028982
C 1.659283 0.574017 3.429122
H 2.467451 -0.013761 3.859477
C 1.105722 0.193468 2.214307
H 1.487878 -0.678380 1.681725
C -1.791564 -0.968095 0.424820
C -1.479911 -1.927400 1.391055
H -0.559062 -1.849047 1.963504
C -2.330433 -3.004368 1.599857
H -2.075848 -3.753464 2.346326
C -3.494048 -3.129668 0.847746
H -4.157325 -3.977347 1.009443
C -3.808174 -2.176113 -0.114655
H -4.714419 -2.276401 -0.708641
C -2.959665 -1.096065 -0.329871
H -3.199512 -0.361693 -1.095600
C -1.545463 1.669149 -0.701896
C -1.130815 2.147977 -1.944391
H -0.240342 1.718098 -2.402866
C -1.855119 3.149505 -2.582121
H -1.524600 3.524210 -3.548780
C -3.001786 3.664115 -1.988017
H -3.570536 4.445307 -2.489052
C -3.432989 3.173510 -0.757582
H -4.340312 3.564949 -0.301481
C -2.709847 2.176408 -0.115292
H -3.061989 1.775830 0.834938
Cl 1.522515 -3.392921 0.130343
Ge 0.626943 -2.101818 -1.445741
H 1.508767 -2.374772 -2.670107
H -0.781390 -2.670288 -1.634178

^{Ph}YGeHMDS
E = -4261.62255210
P -1.825677 -0.104535 0.010642
C -0.187649 0.295592 -0.152013
C 0.215126 1.659764 -0.556024
C -0.349987 2.827687 -0.016475
H -1.118978 2.743732 0.748938
C 0.068780 4.090174 -0.422425
H -0.390118 4.973795 0.018638
C 1.077994 4.229704 -1.369492
H 1.412684 5.218167 -1.678092
C 1.647818 3.085349 -1.922832
H 2.428391 3.174074 -2.677751
C 1.214518 1.826464 -1.530546
H 1.636058 0.936860 -1.988108
C -2.766009 0.659826 1.396725
C -4.111801 1.025108 1.339860
H -4.682199 0.877225 0.425996
C -4.726523 1.597477 2.449010

H -5.774597 1.886032 2.395327
C -4.005529 1.806767 3.619852
H -4.489968 2.256647 4.484438
C -2.660632 1.453624 3.679761
H -2.088449 1.627967 4.588835
C -2.041416 0.891396 2.570429
H -0.980242 0.642050 2.599110
C -1.958381 -1.891009 0.293468
C -2.334754 -2.438148 1.517939
H -2.633211 -1.788896 2.338171
C -2.316388 -3.819399 1.693176
H -2.601747 -4.245137 2.652962
C -1.930131 -4.649719 0.648411
H -1.913164 -5.728735 0.789953
C -1.565960 -4.104130 -0.581058
H -1.264307 -4.753894 -1.400119
C -1.579339 -2.728872 -0.759983
H -1.275803 -2.292920 -1.710590
C -2.845273 0.201539 -1.469042
C -2.445533 1.118285 -2.443652
H -1.518670 1.671908 -2.323532
C -3.230677 1.314468 -3.574545
H -2.913098 2.031800 -4.328592
C -4.408648 0.595648 -3.746739
H -5.016822 0.750222 -4.636025
C -4.803304 -0.329990 -2.786035
H -5.716753 -0.905617 -2.922488
C -4.024744 -0.528115 -1.652242
H -4.328690 -1.265348 -0.910038
N 2.829225 -0.389190 0.171830
Si 3.435029 -1.293914 -1.201526
Si 3.796128 0.650445 1.208860
C 1.990989 -2.131979 -2.071797
H 1.554234 -2.938115 -1.470079
H 1.191556 -1.411562 -2.292638
H 2.334392 -2.565997 -3.019792
C 4.305018 -0.220110 -2.485048
H 5.346798 -0.013619 -2.217699
H 4.311509 -0.749252 -3.447559
H 3.801752 0.742592 -2.634602
C 4.657453 -2.599407 -0.621327
H 5.058510 -3.188384 -1.456721
H 5.505946 -2.129598 -0.105189
H 4.179556 -3.288593 0.086750
C 5.352124 1.225610 0.328687
H 5.923889 1.866490 1.013074
H 6.004195 0.393200 0.035494
H 5.116651 1.813906 -0.566148
C 4.326441 -0.298235 2.744782
H 4.947207 0.318542 3.408205
H 3.454894 -0.638984 3.317603
H 4.907721 -1.186091 2.462228
C 2.827581 2.176499 1.715823
H 1.858596 1.926885 2.165289
H 3.399247 2.752431 2.456064
H 2.632861 2.823873 0.851602
Ge 1.114574 -0.762460 0.794871
H 0.850844 -2.276074 0.717421
H 1.180359 -0.413155 2.308793

$^{Ph}YGeC_6F_5$
E = -4116.16452380
P -1.815536 0.073315 0.024730
C -0.281876 0.188355 -0.670014
C 0.637363 1.293848 -0.293769
C 1.192608 1.391350 0.993221
H 0.887037 0.672276 1.752502
C 2.145674 2.354631 1.301629
H 2.566065 2.397190 2.305315
C 2.579038 3.246760 0.324507
H 3.339196 3.990011 0.557177
C 2.033292 3.176485 -0.952716
H 2.363896 3.869184 -1.724756
C 1.067947 2.221030 -1.252157
H 0.648555 2.162270 -2.254704
C -1.971669 -0.453590 1.777806
C -3.037480 -0.058289 2.590450
H -3.801839 0.611279 2.199160
C -3.118812 -0.513858 3.901652
H -3.947311 -0.199769 4.533783
C -2.139361 -1.364262 4.406816
H -2.202338 -1.714591 5.435431
C -1.076667 -1.760342 3.600711
H -0.305830 -2.420614 3.993126
C -0.991883 -1.304735 2.289936
H -0.153052 -1.598557 1.660350
C -2.799601 -1.129029 -0.912507
C -3.604400 -2.083337 -0.289172
H -3.657824 -2.126298 0.796212
C -4.326688 -2.990739 -1.056631
H -4.945793 -3.738695 -0.565455
C -4.252049 -2.949011 -2.444495
H -4.815495 -3.663945 -3.041070
C -3.454059 -1.996175 -3.070788
H -3.392182 -1.962210 -4.156573
C -2.729288 -1.087795 -2.309779
H -2.095681 -0.342473 -2.788481
C -2.692032 1.668561 0.007765
C -2.218612 2.695673 0.831588
H -1.388452 2.510764 1.511177
C -2.805369 3.953503 0.783038
H -2.426780 4.749402 1.421176
C -3.873170 4.193653 -0.076450
H -4.334137 5.178962 -0.110289
C -4.352731 3.172841 -0.889714
H -5.191813 3.355283 -1.558291
C -3.763101 1.913666 -0.852470
H -4.141977 1.120030 -1.493194
C 2.392128 -1.145875 -0.850916
C 3.866018 -1.244296 1.081804
C 4.418089 0.199423 -0.763442
C 4.716600 -0.318309 0.490478
Ge 0.548219 -1.302998 -1.540674
H 0.629209 -1.231078 -3.078447
H -0.031440 -2.671796 -1.149735
C 3.272323 -0.234938 -1.417150
C 2.724996 -1.641514 0.398905
F 1.902890 -2.489956 1.025085
F 3.006711 0.299941 -2.606088
F 5.219475 1.095375 -1.314492

F 5.804940 0.077208 1.126617
F 4.148724 -1.728403 2.281924

^{Ph}YGePy
E = -3635.30673197
P -1.104380 -0.194568 0.022904
C 0.289519 0.214218 -0.834371
C 0.757269 1.623028 -0.716733
C 1.653849 2.026981 0.283545
C 2.044475 3.354870 0.412733
C 1.544131 4.318580 -0.456976
C 0.656865 3.939529 -1.459284
C 0.274316 2.609664 -1.587369
C -0.950999 -0.555928 1.823135
C -2.029173 -0.459046 2.707620
H -3.007320 -0.150287 2.340137
C -1.853390 -0.745969 4.056059
H -2.695081 -0.667397 4.741783
C -0.600184 -1.125260 4.530852
H -0.463572 -1.343794 5.588393
C 0.477710 -1.213507 3.656775
H 1.460894 -1.498530 4.026592
C 0.302360 -0.925763 2.307181
H 1.144105 -0.970671 1.616245
C -1.912776 -1.660351 -0.685488
C -2.499063 -2.655378 0.097213
H -2.463485 -2.589269 1.181933
C -3.116565 -3.744006 -0.509454
H -3.565669 -4.520753 0.106329
C -3.153103 -3.844736 -1.895789
H -3.634481 -4.699832 -2.366718
C -2.567648 -2.855677 -2.680667
H -2.587990 -2.934922 -3.765771
C -1.947722 -1.767803 -2.079652
H -1.470741 -0.994102 -2.679899
C -2.310003 1.169055 0.016494
C -2.044132 2.292779 0.805236
H -1.170533 2.306030 1.455641
C -2.882317 3.398067 0.747763
H -2.662192 4.273703 1.354941
C -3.995606 3.386052 -0.087199
H -4.653813 4.251892 -0.129276
C -4.267402 2.267790 -0.867961
H -5.139852 2.254374 -1.518360
C -3.424761 1.162087 -0.821850
H -3.637939 0.291295 -1.438735
C 3.239586 -0.946203 -0.597121
C 4.855412 -1.451707 1.113996
C 5.158607 0.305556 -0.471733
C 5.632146 -0.409295 0.625094
N 4.002029 0.048284 -1.068693
H 5.191413 -2.044375 1.963928
H 6.588359 -0.154594 1.077415
H 5.746186 1.128171 -0.885707
H 2.042045 1.278246 0.973021
H 2.741427 3.638753 1.199880
H 1.846203 5.359582 -0.355450
H 0.259438 4.684973 -2.146426
H -0.426226 2.311420 -2.365550
C 3.641706 -1.723795 0.493983

H 3.007275 -2.535820 0.847495
Ge 1.486931 -1.137877 -1.472535
H 1.743653 -1.044417 -2.990566
H 0.938949 -2.540083 -1.129031

^{Ph}*YGePyr*
E = -3651.37222768
P -1.198476 -0.031692 0.002341
C 0.344247 -0.012681 -0.701871
C 1.124738 1.221914 -0.789611
C 0.913824 2.336956 0.043570
C 1.691908 3.483251 -0.056096
C 2.736711 3.554976 -0.970632
C 2.980932 2.456918 -1.792133
C 2.186828 1.322957 -1.711655
C -1.310928 0.066841 1.828509
C -2.514449 0.246724 2.519800
H -3.448517 0.359464 1.969666
C -2.519873 0.291060 3.907904
H -3.457206 0.429521 4.443600
C -1.324137 0.162806 4.611873
H -1.329683 0.200605 5.699800
C -0.126606 -0.006366 3.927162
H 0.808647 -0.100122 4.475651
C -0.114045 -0.053549 2.535741
H 0.826906 -0.176093 1.993298
C -2.020612 -1.590824 -0.462793
C -2.798212 -2.330610 0.428798
H -2.921196 -1.998063 1.455628
C -3.399653 -3.514662 0.016351
H -3.997745 -4.087476 0.722275
C -3.228733 -3.971750 -1.285244
H -3.697628 -4.901067 -1.603029
C -2.446881 -3.244495 -2.176791
H -2.297642 -3.602907 -3.193269
C -1.843886 -2.061586 -1.769286
H -1.209239 -1.501919 -2.454821
C -2.254334 1.338865 -0.591898
C -2.540254 2.461868 0.186436
H -2.180656 2.523227 1.211045
C -3.277327 3.514588 -0.345166
H -3.487805 4.388141 0.268601
C -3.735438 3.454441 -1.655610
H -4.312380 4.278902 -2.070214
C -3.448713 2.340722 -2.439199
H -3.799999 2.290299 -3.467798
C -2.707413 1.291848 -1.912346
H -2.475831 0.430159 -2.534078
C 3.102557 -1.204854 -0.066414
C 4.247380 -0.138056 1.586671
C 5.349375 -0.979182 -0.320208
C 5.435951 -0.295478 0.884612
N 4.195208 -1.431251 -0.796876
N 3.091598 -0.590078 1.121360
H 4.227440 0.372391 2.552112
H 6.380122 0.090473 1.260841
H 6.239770 -1.164189 -0.925740
H 0.148337 2.292052 0.815703
H 1.490406 4.322556 0.608501
H 3.354466 4.448101 -1.039251

H 3.794601 2.487541 -2.515684
H 2.387523 0.485270 -2.379962
Ge 1.332904 -1.659900 -0.811702
H 1.563969 -2.201737 -2.238276
H 0.692169 -2.779494 0.030525

^{Ph}YGeB2
E = -3794.32403634
P 1.634594 0.057013 0.062425
C 0.301248 -0.077861 -0.959377
C -0.500528 -1.330773 -0.870194
C -1.492081 -1.506441 0.108666
C -2.244699 -2.673585 0.181772
C -2.013602 -3.707692 -0.719447
C -1.027121 -3.560896 -1.689824
C -0.284669 -2.387622 -1.764519
C 1.341762 0.447415 1.838555
C 2.229258 0.077207 2.852918
H 3.134232 -0.477400 2.607798
C 1.955108 0.406906 4.175475
H 2.647296 0.112729 4.962371
C 0.793648 1.105822 4.493268
H 0.578446 1.358797 5.529881
C -0.094774 1.472515 3.487942
H -1.007002 2.013248 3.732534
C 0.175905 1.141004 2.164864
H -0.530457 1.409840 1.379539
C 2.769481 1.348508 -0.521593
C 3.477155 2.176412 0.350709
H 3.337930 2.082904 1.425237
C 4.351071 3.132787 -0.154529
H 4.896204 3.779937 0.529729
C 4.521596 3.268111 -1.528080
H 5.203389 4.020685 -1.919872
C 3.815926 2.445900 -2.401494
H 3.942578 2.553812 -3.476877
C 2.942380 1.488171 -1.902306
H 2.375211 0.843378 -2.572160
C 2.561586 -1.506968 0.147962
C 1.995333 -2.584938 0.836061
H 1.055080 -2.455967 1.370394
C 2.625453 -3.822702 0.830536
H 2.173915 -4.659438 1.359677
C 3.826929 -3.991228 0.149063
H 4.321136 -4.960986 0.148574
C 4.396529 -2.920005 -0.530777
H 5.338346 -3.047756 -1.060926
C 3.764659 -1.681116 -0.536779
H 4.210786 -0.847156 -1.075076
H -1.662001 -0.708741 0.830777
H -3.011728 -2.774516 0.948156
H -2.599111 -4.623855 -0.665980
H -0.836276 -4.366372 -2.397441
H 0.486820 -2.270560 -2.523437
Ge -0.565242 1.491087 -1.668922
H -0.494249 1.578137 -3.212253
H 0.077811 2.776204 -1.109098
C -4.514157 0.445024 -0.746943
C -4.136708 1.052085 0.447052
C -5.725569 -0.203356 -0.885319

C -4.951313 1.043554 1.562145
C -6.556610 -0.219805 0.236420
H -6.004610 -0.677737 -1.822056
C -6.178506 0.389935 1.433285
H -4.643637 1.520508 2.488847
H -7.519958 -0.721594 0.175728
H -6.853225 0.354432 2.285932
B -2.501681 1.284476 -1.007678
O -2.895714 1.594493 0.290127
O -3.515264 0.605118 -1.658319

^{Ph}YGeB1
E = -4063.51376319
P 1.855259 -0.686721 0.089294
C 0.245090 -0.178895 0.287845
C -0.159026 0.539373 1.496902
C 0.722348 1.349596 2.247908
H 1.757842 1.454581 1.933725
C 0.304101 2.044772 3.374726
H 1.024755 2.657540 3.915111
C -1.018483 1.983034 3.804365
H -1.347925 2.540465 4.678728
C -1.902995 1.168024 3.101315
H -2.937113 1.070122 3.429587
C -1.477597 0.447731 1.994303
H -2.178066 -0.228109 1.508963
C 3.125556 0.630538 -0.045361
C 4.464559 0.464766 0.313529
H 4.803669 -0.467025 0.758560
C 5.371103 1.500401 0.117510
H 6.413206 1.366925 0.401730
C 4.945871 2.703635 -0.436139
H 5.657125 3.513121 -0.590176
C 3.608819 2.877542 -0.778172
H 3.268322 3.821681 -1.194662
C 2.695976 1.848863 -0.576400
H 1.638030 1.983945 -0.807814
C 2.024388 -1.633833 -1.448524
C 2.655368 -1.105862 -2.573683
H 3.104420 -0.116145 -2.532862
C 2.699635 -1.842843 -3.752299
H 3.188869 -1.426694 -4.630645
C 2.118372 -3.104455 -3.808991
H 2.152784 -3.677828 -4.733511
C 1.493918 -3.636665 -2.684351
H 1.038558 -4.623915 -2.726820
C 1.446917 -2.905599 -1.505374
H 0.953868 -3.314159 -0.624503
C 2.479073 -1.833398 1.371254
C 1.818230 -1.930010 2.597409
H 0.949518 -1.307232 2.793591
C 2.262571 -2.831846 3.557994
H 1.744073 -2.897229 4.512441
C 3.354200 -3.653422 3.298275
H 3.694775 -4.362390 4.050656
C 4.000704 -3.580035 2.068455
H 4.842126 -4.235618 1.852175
C 3.564326 -2.675901 1.107337
H 4.057706 -2.640130 0.136679
N -3.958990 0.388843 -0.346478

C -3.674162 2.573090 0.041156
C -4.537121 1.542353 0.173455
H -3.817850 3.618552 0.288736
H -5.555779 1.553371 0.543130
B -2.628032 0.712225 -0.798957
N -2.511457 2.123243 -0.569084
C -1.398267 2.965836 -0.759000
C -0.879999 3.690627 0.314890
C -0.838057 3.109002 -2.028743
C 0.152852 4.595286 0.103628
H -1.283679 3.521115 1.311922
C 0.196667 4.014486 -2.232322
H -1.245400 2.528122 -2.853387
C 0.678525 4.776701 -1.172067
H 0.550554 5.159241 0.945028
H 0.614506 4.137892 -3.229791
H 1.468533 5.506844 -1.338036
C -4.561650 -0.875356 -0.227824
C -4.465018 -1.800260 -1.269131
C -5.255821 -1.224396 0.933500
C -5.043452 -3.055963 -1.143372
H -3.939765 -1.517540 -2.178364
C -5.844736 -2.477623 1.046058
H -5.311352 -0.513711 1.755665
C -5.739500 -3.400477 0.011190
H -4.958542 -3.767956 -1.962151
H -6.379652 -2.737769 1.957614
H -6.197695 -4.382940 0.103300
Ge -1.034554 -0.537690 -1.133321
H -1.492456 -2.018037 -1.161407
H -0.367564 -0.310805 -2.510129

$^{F}Y_2Ge$
E = -5689.04492829
P 3.047828 0.773479 -0.120703
P -3.037414 0.948875 0.001415
C 1.535408 0.307570 -0.749702
C 1.203731 -1.039673 -1.169534
C 1.629219 -2.221456 -0.548022
C 1.165445 -3.477816 -0.903890
C 0.233033 -3.615394 -1.924866
C -0.192510 -2.475817 -2.595756
C 0.315774 -1.237919 -2.239406
C 3.204712 0.949720 1.691588
C 3.451133 -0.163780 2.500644
H 3.694298 -1.122512 2.052496
C 3.371055 -0.055784 3.882572
H 3.557712 -0.932043 4.499211
C 3.030081 1.155673 4.472737
H 2.957105 1.233101 5.555847
C 2.777624 2.265946 3.675384
H 2.506486 3.216638 4.130399
C 2.865276 2.166452 2.292863
H 2.656600 3.038498 1.677812
C 3.520466 2.418068 -0.759716
C 4.599081 3.110166 -0.201596
H 5.117656 2.702157 0.665023
C 5.007449 4.319763 -0.746234
H 5.846448 4.855676 -0.306770
C 4.343343 4.845517 -1.852000

H 4.661612 5.796429 -2.275293
C 3.280087 4.152628 -2.418279
H 2.764354 4.556531 -3.287149
C 2.872818 2.935687 -1.880191
H 2.056387 2.373084 -2.329559
C 4.396719 -0.300726 -0.689780
C 4.294524 -0.880803 -1.956877
H 3.400864 -0.716525 -2.554281
C 5.336657 -1.653897 -2.452883
H 5.247689 -2.109634 -3.436912
C 6.489599 -1.841377 -1.697444
H 7.303435 -2.449622 -2.087414
C 6.604933 -1.243315 -0.447555
H 7.510033 -1.377385 0.141377
C 5.563957 -0.472296 0.056495
H 5.663616 -0.004033 1.033158
C -1.422960 0.462825 0.211851
C -1.115995 -0.791301 0.889297
C -0.207294 -0.831403 1.958270
C 0.183278 -2.015742 2.563761
C -0.340439 -3.226704 2.125674
C -1.268917 -3.228907 1.095010
C -1.629961 -2.031885 0.496537
C -4.070761 0.331017 1.370503
C -4.157657 1.038775 2.570949
H -3.687928 2.014868 2.664844
C -4.851126 0.503009 3.648674
H -4.921112 1.065229 4.577712
C -5.451875 -0.747559 3.541237
H -5.990010 -1.167931 4.388682
C -5.363443 -1.458964 2.350289
H -5.828080 -2.438927 2.261846
C -4.679877 -0.922362 1.264767
H -4.615382 -1.479788 0.333242
C -3.096342 2.769069 0.011804
C -3.869168 3.506826 -0.886465
H -4.463402 2.998901 -1.641621
C -3.866008 4.895985 -0.829913
H -4.465126 5.464536 -1.538365
C -3.094415 5.556695 0.119667
H -3.092094 6.644435 0.158000
C -2.317641 4.826893 1.013748
H -1.703781 5.339377 1.751640
C -2.313122 3.438977 0.959060
H -1.689279 2.859361 1.637794
C -3.976023 0.447965 -1.485615
C -5.368882 0.556311 -1.548826
H -5.927019 0.939976 -0.695111
C -6.042344 0.159316 -2.697228
H -7.126691 0.240359 -2.744983
C -5.331744 -0.354476 -3.780344
H -5.864154 -0.675558 -4.673840
C -3.947868 -0.468886 -3.715848
H -3.385584 -0.886417 -4.548515
C -3.271679 -0.064537 -2.570296
H -2.193642 -0.164332 -2.499287
Ge 0.049846 1.524668 -0.455522
H 0.569595 2.611014 0.520811
H -0.428775 2.300107 -1.706482
F 2.457946 -2.149775 0.498160

F 1.548609 -4.543509 -0.215509
F -0.242309 -4.808222 -2.246905
F 0.037087 -4.361335 2.693145
F -1.744746 -4.376300 0.632582
F -1.068781 -2.574727 -3.587198
F -2.463628 -2.098914 -0.546016
F -0.083496 -0.177825 -2.948997
F 0.291918 0.309852 2.427877
F 1.039931 -2.012435 3.576305

^{F}YGeCl
E = -4344.73667122
P -0.925900 0.551607 0.151079
C 0.082252 -0.595167 -0.626817
C 1.529318 -0.438792 -0.587165
C 2.173047 0.799593 -0.718899
C 3.546713 0.961062 -0.629054
C 4.355843 -0.144079 -0.402546
C 3.766610 -1.396362 -0.274244
C 2.387538 -1.522708 -0.341107
C -0.012125 1.329062 1.512663
C -0.024895 2.703317 1.749073
H -0.604971 3.364205 1.109937
C 0.728255 3.233422 2.790464
H 0.719347 4.306627 2.969010
C 1.499842 2.399309 3.592115
H 2.092305 2.819965 4.402257
C 1.527168 1.029355 3.349862
H 2.142265 0.375734 3.964764
C 0.780123 0.493932 2.308977
H 0.817202 -0.573292 2.093181
C -2.383443 -0.287048 0.836235
C -2.346076 -0.852195 2.112158
H -1.494746 -0.677192 2.764734
C -3.394100 -1.650015 2.548875
H -3.358456 -2.088797 3.543659
C -4.474883 -1.903770 1.711194
H -5.285392 -2.546501 2.048867
C -4.522257 -1.334127 0.444528
H -5.368617 -1.526561 -0.211354
C -3.485752 -0.517993 0.009092
H -3.527215 -0.073173 -0.983185
C -1.650212 1.875349 -0.870098
C -1.215021 2.038863 -2.182818
H -0.425353 1.391228 -2.559718
C -1.786691 3.019099 -2.986750
H -1.444576 3.145642 -4.011839
C -2.794106 3.833307 -2.481513
H -3.241494 4.599292 -3.112117
C -3.239543 3.665638 -1.172371
H -4.035186 4.296410 -0.780954
C -2.674517 2.684562 -0.368280
H -3.037450 2.537624 0.648783
Cl -1.418022 -3.604972 0.023271
Ge -0.712293 -2.124028 -1.433577
H 0.292649 -2.834746 -2.343818
H -1.992832 -1.772363 -2.206846
F 4.089284 2.161024 -0.769097
F 5.670853 -0.009924 -0.322892
F 4.523373 -2.456966 -0.039358

F 1.866070 -2.730722 -0.137534
F 1.439557 1.891427 -0.960466

[F]YGeHMDS
E = -4758.53129273
P -1.999770 -0.445748 -0.079495
C -0.327936 -0.161899 0.002522
C 0.183340 1.196272 -0.173733
C -0.215719 2.286306 0.604923
C 0.306413 3.561657 0.449763
C 1.291904 3.784329 -0.503282
C 1.716174 2.730291 -1.304520
C 1.140761 1.477314 -1.155890
C -2.989007 -0.442939 1.458413
C -4.386786 -0.407082 1.439105
H -4.918125 -0.360665 0.488696
C -5.098653 -0.416155 2.631984
H -6.186352 -0.383773 2.615094
C -4.419568 -0.458577 3.847426
H -4.978984 -0.462563 4.781046
C -3.029713 -0.483106 3.869880
H -2.498269 -0.502549 4.819071
C -2.314376 -0.470028 2.677438
H -1.225506 -0.459676 2.680821
C -2.281468 -2.084710 -0.826251
C -3.245801 -2.986846 -0.375301
H -3.876162 -2.738248 0.474583
C -3.388452 -4.221077 -1.001162
H -4.136709 -4.923746 -0.640047
C -2.572661 -4.561466 -2.074584
H -2.685059 -5.530773 -2.556665
C -1.603829 -3.668511 -2.522943
H -0.949872 -3.935492 -3.350708
C -1.455582 -2.437938 -1.898802
H -0.677796 -1.742611 -2.210958
C -2.802105 0.811920 -1.121257
C -3.303607 1.981284 -0.543450
H -3.289057 2.104583 0.536970
C -3.807286 2.994687 -1.351097
H -4.189489 3.905828 -0.895381
C -3.819628 2.845637 -2.733331
H -4.216317 3.639576 -3.363160
C -3.323035 1.681527 -3.311462
H -3.332508 1.560650 -4.392736
C -2.811168 0.668808 -2.510094
H -2.421767 -0.237542 -2.967904
N 2.642931 -1.016808 0.409388
Si 3.584085 -1.650952 -0.938013
Si 3.389766 -0.273085 1.819589
C 2.455992 -2.507849 -2.174504
H 2.016442 -3.429703 -1.776380
H 1.643984 -1.844071 -2.493292
H 3.042955 -2.771108 -3.064250
C 4.551572 -0.323344 -1.854866
H 5.474056 -0.045091 -1.334594
H 4.838750 -0.713486 -2.840906
H 3.959881 0.583388 -2.017192
C 4.817962 -2.920210 -0.295726
H 5.404581 -3.355790 -1.115476
H 5.525115 -2.468204 0.412296

H 4.301700 -3.736939 0.225381
C 5.007084 0.564732 1.368225
H 5.403598 1.064960 2.261836
H 5.771831 -0.138727 1.017624
H 4.858372 1.325964 0.592641
C 3.738413 -1.562725 3.142231
H 4.219356 -1.112701 4.020857
H 2.812727 -2.047980 3.475339
H 4.404113 -2.343890 2.753470
C 2.265246 1.059214 2.520680
H 1.219272 0.741924 2.624112
H 2.620600 1.368404 3.512323
H 2.283788 1.940975 1.867782
Ge 0.873595 -1.525781 0.652712
H 0.600042 -2.895834 0.012370
H 0.725305 -1.725596 2.185299
F 1.502718 0.528686 -2.012682
F 2.631270 2.943132 -2.240700
F 1.812125 4.991154 -0.658122
F -0.102756 4.557396 1.222508
F -1.099835 2.097274 1.586542

$^{F}YGeC_6F_5$
E = -4613.07173097
P -2.176520 -0.062939 0.030931
C -0.519496 -0.378857 0.187973
C 0.470229 0.654558 0.472281
C 1.408289 0.477885 1.498449
C 2.495775 1.316957 1.686115
C 2.646961 2.432022 0.870730
C 1.731668 2.658817 -0.150608
C 0.681797 1.772727 -0.347027
C -3.207588 -0.707677 1.395868
C -4.558585 -0.362909 1.508183
H -5.017931 0.290896 0.766918
C -5.310234 -0.838645 2.574010
H -6.360527 -0.567415 2.661483
C -4.716264 -1.652702 3.536444
H -5.306357 -2.022005 4.373077
C -3.368948 -1.979262 3.438833
H -2.899780 -2.599661 4.199707
C -2.611295 -1.502684 2.372933
H -1.546147 -1.719421 2.304393
C -2.849303 -0.773655 -1.501535
C -4.019037 -1.530353 -1.569338
H -4.595941 -1.733592 -0.670764
C -4.439089 -2.048545 -2.790397
H -5.346735 -2.646761 -2.838413
C -3.697250 -1.811704 -3.941799
H -4.027801 -2.221018 -4.894576
C -2.523745 -1.064626 -3.875291
H -1.933668 -0.890939 -4.772738
C -2.093053 -0.553477 -2.658784
H -1.158261 0.001538 -2.585207
C -2.548987 1.709390 0.051992
C -2.219202 2.424544 1.206831
H -1.785323 1.906280 2.061145
C -2.443085 3.793642 1.259945
H -2.179042 4.350653 2.156481
C -3.005981 4.449607 0.168719

H -3.181819 5.522656 0.212059
C -3.345034 3.736245 -0.975422
H -3.786158 4.248685 -1.827819
C -3.114023 2.366693 -1.039327
H -3.364847 1.812893 -1.941380
C 2.071573 -1.773984 -0.482950
C 4.361651 -1.614982 0.314883
C 3.657031 -0.272427 -1.558959
C 4.646245 -0.673817 -0.667022
C 2.396397 -0.842709 -1.459965
C 3.081209 -2.152560 0.390189
F 2.837058 -3.013696 1.369322
F 1.459927 -0.418762 -2.309164
F 3.925662 0.638114 -2.479444
F 5.856501 -0.156384 -0.752203
F 5.306850 -1.988095 1.159828
F -0.098875 1.973627 -1.404912
F 1.888698 3.700536 -0.952284
F 3.659682 3.262576 1.055715
F 3.375922 1.073781 2.645055
F 1.306775 -0.591244 2.289694
Ge 0.171768 -2.147321 -0.138018
H -0.476711 -2.797912 -1.371219
H 0.102709 -3.145692 1.029295

^{F}YGePy
E = -4132.21905465
P -1.606689 0.010658 0.049155
C -0.050867 -0.424858 0.578806
C 1.067041 0.506667 0.506301
C 2.111147 0.426774 1.441434
C 3.278729 1.167434 1.349926
C 3.430408 2.089945 0.323151
C 2.421624 2.213293 -0.623219
C 1.286294 1.422996 -0.533225
C -2.939994 -0.603267 1.139408
C -4.168936 0.063942 1.176094
H -4.320745 0.959585 0.575517
C -5.192998 -0.391919 1.997612
H -6.142391 0.139397 2.021120
C -4.999517 -1.514678 2.795123
H -5.800170 -1.870000 3.440889
C -3.773906 -2.170973 2.777912
H -3.608451 -3.038983 3.412741
C -2.746815 -1.714112 1.960474
H -1.784841 -2.217816 1.988828
C -2.062781 -0.577879 -1.620878
C -3.384003 -0.625781 -2.075204
H -4.201935 -0.321504 -1.425874
C -3.662890 -1.077309 -3.358932
H -4.692951 -1.111928 -3.708393
C -2.627108 -1.494931 -4.190288
H -2.848066 -1.855507 -5.193323
C -1.313879 -1.461731 -3.735446
H -0.502468 -1.798740 -4.377363
C -1.026414 -1.004542 -2.454009
H 0.003982 -0.990071 -2.095393
C -1.834050 1.809157 0.097383
C -1.480700 2.466792 1.278453
H -1.077714 1.892921 2.111767

C -1.635968 3.843309 1.375474
H -1.355601 4.355686 2.293511
C -2.144966 4.564544 0.299234
H -2.264880 5.643494 0.376402
C -2.493342 3.909893 -0.877140
H -2.881643 4.474753 -1.722179
C -2.334463 2.532480 -0.983563
H -2.583762 2.023245 -1.912032
C 2.279004 -2.254460 -0.122121
C 4.669660 -2.375690 0.056241
C 3.561180 -1.434927 -1.838143
C 4.747650 -1.757543 -1.184496
F 0.417616 1.495129 -1.544294
F 2.570392 3.055354 -1.636759
F 4.526519 2.830744 0.239451
F 4.236693 1.020962 2.254120
F 2.022334 -0.459564 2.435713
N 2.360699 -1.669935 -1.323893
H 5.573400 -2.641472 0.602231
H 5.706613 -1.522483 -1.640937
H 3.585918 -0.952241 -2.817239
C 3.414175 -2.632624 0.594890
H 3.310540 -3.091197 1.576383
Ge 0.471667 -2.280511 0.642203
H -0.495306 -3.126245 -0.212159
H 0.540670 -2.928505 2.043332

^{F}YGePyr
E = -4148.27595621
P -1.611623 0.014119 0.054128
C -0.056732 -0.453009 0.559524
C 1.066148 0.473858 0.518791
C 2.125320 0.330175 1.431239
C 3.316715 1.032242 1.334132
C 3.470887 1.996148 0.345797
C 2.442491 2.192017 -0.566425
C 1.284992 1.434041 -0.480107
C -2.948256 -0.665999 1.098644
C -4.174493 0.003267 1.172225
H -4.318923 0.936814 0.630511
C -5.205475 -0.500468 1.955995
H -6.152683 0.032620 2.008752
C -5.021525 -1.673874 2.679227
H -5.827394 -2.067130 3.295839
C -3.798735 -2.333666 2.625742
H -3.640840 -3.242274 3.203026
C -2.764762 -1.829626 1.845446
H -1.805966 -2.340115 1.845709
C -2.066462 -0.467363 -1.651026
C -3.386396 -0.482923 -2.111081
H -4.205183 -0.216183 -1.446515
C -3.663071 -0.853599 -3.420971
H -4.692199 -0.862669 -3.774712
C -2.626526 -1.222935 -4.273778
H -2.845820 -1.519938 -5.297768
C -1.314313 -1.222153 -3.814473
H -0.502560 -1.520864 -4.474644
C -1.029602 -0.845520 -2.506712
H -0.000818 -0.851932 -2.144351
C -1.835660 1.806640 0.215227

C -1.482283 2.386770 1.436253
H -1.079997 1.760338 2.231152
C -1.638139 3.754076 1.621943
H -1.358438 4.205976 2.571415
C -2.147260 4.543171 0.594468
H -2.267842 5.614782 0.741361
C -2.494920 3.965790 -0.621945
H -2.883242 4.584034 -1.428734
C -2.335994 2.598128 -0.816928
H -2.585631 2.149715 -1.776191
C 2.285983 -2.217362 -0.217454
C 4.531406 -2.360457 0.091143
C 3.624490 -1.234748 -1.774143
C 4.753761 -1.611140 -1.056537
F 0.398973 1.573579 -1.469176
F 2.592081 3.072792 -1.546625
F 4.589553 2.702744 0.262394
F 4.295025 0.813039 2.200562
F 2.027568 -0.587198 2.391986
Ge 0.466270 -2.313805 0.531950
H -0.482488 -3.101350 -0.396877
H 0.522105 -3.037388 1.887770
N 2.399609 -1.534718 -1.362835
N 3.307990 -2.663824 0.511268
H 5.364941 -2.720964 0.697698
H 5.755177 -1.339719 -1.380858
H 3.710008 -0.667469 -2.703499

^{F}YGeB2
E = -4291.23124642
P -2.030188 0.044640 -0.017831
C -0.473488 -0.530411 0.307982
C 0.686370 0.357647 0.381962
C 1.531194 0.348110 1.500564
C 2.728679 1.049581 1.553487
C 3.115626 1.827425 0.471754
C 2.303197 1.877324 -0.654457
C 1.125284 1.147538 -0.690936
C -3.199620 -0.045162 1.385436
C -4.448760 0.580804 1.328278
H -4.747439 1.119312 0.428930
C -5.301336 0.532465 2.423039
H -6.271780 1.023052 2.377743
C -4.908486 -0.134143 3.581773
H -5.576831 -0.169016 4.440118
C -3.659287 -0.739709 3.648435
H -3.344797 -1.245725 4.559000
C -2.800147 -0.690854 2.553977
H -1.804114 -1.128943 2.603853
C -2.827726 -0.843918 -1.392804
C -4.126355 -1.350981 -1.354138
H -4.736942 -1.231954 -0.462858
C -4.637555 -2.034699 -2.452911
H -5.647683 -2.437587 -2.414649
C -3.858574 -2.211077 -3.590304
H -4.261451 -2.748068 -4.447045
C -2.557837 -1.714789 -3.628537
H -1.941105 -1.864368 -4.512434
C -2.038149 -1.042158 -2.531101
H -1.011645 -0.677121 -2.532085

C -2.025725 1.814436 -0.413718
C -1.594141 2.691630 0.585806
H -1.315698 2.301856 1.564267
C -1.517991 4.053516 0.328013
H -1.175094 4.733120 1.105448
C -1.881013 4.545785 -0.922572
H -1.822215 5.613689 -1.123808
C -2.319499 3.675465 -1.914081
H -2.604127 4.060026 -2.891313
C -2.389086 2.309078 -1.664850
H -2.718507 1.628535 -2.446949
C 3.563775 -1.338813 -1.011595
C 3.954555 -1.665183 0.282695
C 4.362937 -0.588142 -1.851384
C 5.163554 -1.254830 0.808611
C 5.584927 -0.162445 -1.328476
H 4.033213 -0.317741 -2.850746
C 5.975666 -0.487984 -0.028345
H 5.442565 -1.491628 1.831363
H 6.240685 0.449815 -1.943321
H 6.929661 -0.124521 0.346731
B 1.888567 -2.338366 -0.009321
O 2.931932 -2.329080 0.897619
O 2.297522 -1.800773 -1.219118
F 0.440841 1.152226 -1.833555
F 1.209752 -0.411250 2.545102
F 3.511887 0.964215 2.618831
F 2.699263 2.564685 -1.716877
F 4.263382 2.486376 0.495945
Ge -0.107812 -2.432281 0.458977
H -0.978559 -3.216966 -0.541388
H -0.375873 -3.041010 1.853305

^{F}YGeB1
E = -4560.42388592
P -2.001336 0.909469 0.198215
C -0.382591 0.451920 0.003114
C 0.240810 -0.504217 0.909413
C -0.249383 -1.789801 1.154406
C 0.441654 -2.728808 1.905376
C 1.675696 -2.407627 2.451732
C 2.193450 -1.133754 2.249959
C 1.468919 -0.206036 1.520698
C -3.326491 -0.013177 -0.659367
C -4.673626 0.151921 -0.322713
H -4.955473 0.828850 0.483561
C -5.652044 -0.556713 -1.008058
H -6.700166 -0.430885 -0.743155
C -5.288498 -1.433792 -2.027446
H -6.055588 -1.991919 -2.561259
C -3.948384 -1.607637 -2.354106
H -3.662430 -2.305833 -3.137612
C -2.965809 -0.900849 -1.668805
H -1.909479 -1.051840 -1.886478
C -2.191099 2.633696 -0.357445
C -3.275813 3.080393 -1.114353
H -4.053874 2.385965 -1.420031
C -3.355740 4.414893 -1.496134
H -4.200875 4.754435 -2.091619
C -2.357513 5.309772 -1.126779

H -2.422859 6.353118 -1.429852
C -1.269001 4.868219 -0.381333
H -0.477552 5.561064 -0.103103
C -1.180360 3.534716 -0.003242
H -0.317606 3.168821 0.551769
C -2.488576 0.813391 1.951837
C -3.026077 -0.372666 2.459100
H -3.234586 -1.204319 1.790263
C -3.285967 -0.493735 3.819731
H -3.697873 -1.423061 4.208075
C -3.020516 0.566318 4.678842
H -3.227665 0.470663 5.742999
C -2.488898 1.750332 4.176722
H -2.282287 2.583752 4.845131
C -2.217978 1.873113 2.819900
H -1.799182 2.800098 2.435367
N 3.583248 -0.094697 -0.953341
C 3.287025 -2.311785 -0.982250
C 4.150163 -1.327154 -0.650090
H 3.436178 -3.384351 -0.941729
H 5.160166 -1.410912 -0.266473
B 2.261769 -0.314163 -1.478127
N 2.131055 -1.750674 -1.512439
C 1.041229 -2.524276 -1.948998
C 0.661079 -3.685700 -1.272172
C 0.337319 -2.141257 -3.092838
C -0.422767 -4.430427 -1.717113
H 1.200724 -3.993867 -0.380257
C -0.738919 -2.897436 -3.537938
H 0.657522 -1.257682 -3.639131
C -1.129766 -4.041418 -2.849746
H -0.719695 -5.318836 -1.163637
H -1.272341 -2.588610 -4.435488
H -1.976084 -4.630782 -3.196670
C 4.238614 1.123271 -0.684824
C 4.187160 2.160761 -1.615438
C 4.943401 1.301312 0.506555
C 4.824333 3.366388 -1.351075
H 3.651855 2.006938 -2.549759
C 5.591220 2.503471 0.756685
H 4.941191 0.507721 1.249549
C 5.533612 3.541840 -0.167821
H 4.775666 4.169769 -2.083718
H 6.132571 2.634601 1.691732
H 6.038827 4.484153 0.033909
Ge 0.707114 1.040134 -1.500341
H 1.210136 2.492769 -1.344825
H -0.159028 1.050289 -2.783653
F -1.387886 -2.180484 0.579744
F -0.032888 -3.963066 2.030750
F 2.361221 -3.310048 3.138828
F 3.375855 -0.816155 2.764630
F 1.986367 1.011663 1.371711

I
E = -3067.31179563
C 1.651092 2.336652 0.010224
C -0.489004 1.231229 -0.205966
C -1.087048 2.471354 -0.412545
C -0.329981 3.642290 -0.382882

C 1.037007 3.576144 -0.158839
H 2.729138 2.307413 0.169611
H -2.154743 2.524279 -0.608728
H -0.819157 4.601516 -0.539749
H 1.636055 4.484179 -0.123865
N -1.165883 0.005310 -0.228551
C -2.540395 -0.162106 -0.054448
C -3.304085 0.655658 0.792861
C -3.182022 -1.234870 -0.689734
C -4.658308 0.412375 0.974487
H -2.821491 1.471144 1.324614
C -4.534135 -1.479842 -0.490554
H -2.604167 -1.872232 -1.358232
C -5.286716 -0.653327 0.336321
H -5.228109 1.059648 1.639518
H -5.004790 -2.320261 -0.998154
H -6.348190 -0.838648 0.485711
C 0.923202 1.141631 -0.024780
C 1.461101 -0.209984 0.071856
P 3.097713 -0.604027 0.158827
C 4.139983 -0.013851 -1.218374
H 4.019293 1.069876 -1.318687
H 5.198701 -0.250060 -1.061064
H 3.785139 -0.480824 -2.143127
C 3.980453 -0.021401 1.645384
H 5.043193 -0.288792 1.617573
H 3.882148 1.066502 1.721094
H 3.503693 -0.466956 2.524663
C 3.286257 -2.403794 0.167468
H 4.344473 -2.679133 0.223472
H 2.758220 -2.820099 1.032250
H 2.854022 -2.818060 -0.750294
Ge 0.027773 -1.430700 -0.137473
H -0.388313 -2.355791 1.029535
H 0.017238 -2.317001 -1.412427

II
E = -3646.52404562
C 0.818318 2.954728 -0.015441
C 0.357523 1.662965 0.212416
C -0.821888 1.179792 -0.388580
C -1.507640 2.064000 -1.237309
C -1.054951 3.356370 -1.452041
C 0.113521 3.813164 -0.846584
H 1.732206 3.265381 0.487221
H -2.414004 1.714065 -1.726286
H -1.618070 4.012352 -2.113676
H 0.467137 4.827521 -1.016661
N -1.288926 -0.120012 -0.189764
S 1.282161 0.614335 1.318327
O 2.582558 1.281552 1.535011
O 0.417999 0.321171 2.458439
C 1.530452 -0.802801 0.388985
C -2.670654 -0.356717 -0.098265
C -3.509215 0.524568 0.599763
C -3.230234 -1.514592 -0.648447
C -4.862402 0.254219 0.726980
H -3.078601 1.414562 1.052805
C -4.585842 -1.788516 -0.499650
H -2.599446 -2.198365 -1.214930

C -5.412093 -0.904749 0.181920
H -5.494555 0.949747 1.276410
H -4.997501 -2.697503 -0.934751
H -6.473560 -1.115515 0.292281
P 3.007517 -0.873978 -0.466186
C 3.365138 0.562404 -1.514957
H 4.336376 0.462913 -2.012517
H 2.567969 0.669691 -2.258478
H 3.365476 1.445032 -0.866515
C 3.005246 -2.311594 -1.561239
H 2.216159 -2.205594 -2.312376
H 3.976131 -2.393704 -2.061318
H 2.819344 -3.217698 -0.975459
C 4.437474 -1.044315 0.626048
H 4.396479 -0.200888 1.323919
H 4.339323 -1.978604 1.188204
H 5.376746 -1.035360 0.061603
Ge -0.140318 -1.607436 0.013485
H -0.794987 -2.446708 1.112686
H -0.078299 -2.428327 -1.294980

III
E = -4408.61051045
C -0.396148 1.723834 -0.576701
C 0.396309 1.723787 0.576708
C 0.767132 2.940779 1.149437
C 0.382727 4.146750 0.579616
C -0.382293 4.146793 -0.579614
C -0.766839 2.940866 -1.149429
H 1.382191 2.914209 2.045326
H 0.685275 5.083814 1.042963
H -0.684740 5.083891 -1.042959
H -1.381902 2.914361 -2.045318
S -0.983913 0.265959 -1.510049
S 0.983900 0.265843 1.510065
O -2.172133 0.797858 -2.205508
O 0.177353 -0.159674 -2.313853
O -0.177457 -0.159694 2.313785
O 2.172120 0.797642 2.205604
C -1.438783 -0.960171 -0.453938
C 1.438714 -0.960292 0.453931
P 2.898295 -0.709293 -0.409451
P -2.898342 -0.709143 0.409479
C 2.979472 -1.834486 -1.813955
H 3.912502 -1.664536 -2.361656
H 2.939008 -2.872373 -1.469911
H 2.114987 -1.622968 -2.454185
C 3.076054 0.954627 -1.098320
H 3.063696 1.676948 -0.275183
H 4.018452 1.038862 -1.650931
H 2.220704 1.128062 -1.761160
C 4.370261 -0.971946 0.610956
H 4.387980 -2.014802 0.944262
H 5.293012 -0.730749 0.071077
H 4.253582 -0.325304 1.487758
C -2.979667 -1.834484 1.813854
H -3.912670 -1.664473 2.361586
H -2.939351 -2.872337 1.469691
H -2.115153 -1.623158 2.454102
C -3.075925 0.954716 1.098545

H -3.063448 1.677142 0.275499
H -4.018335 1.039002 1.651124
H -2.220574 1.127966 1.761439
C -4.370333 -0.971532 -0.610959
H -5.293058 -0.730303 -0.071052
H -4.253589 -0.324799 -1.487687
H -4.388159 -2.014347 -0.944388
Ge -0.000076 -2.142814 -0.000060
H -0.419462 -3.045455 1.171697
H 0.419244 -3.045421 -1.171869

IV
E = -3616.59318818
C 0.721460 3.208286 0.373034
C 0.200866 1.926085 0.392499
C -1.000236 1.528245 -0.196348
C -1.721700 2.521212 -0.859074
C -1.239573 3.827694 -0.893398
C -0.032747 4.175883 -0.282608
H 1.663453 3.446636 0.863005
H -2.674340 2.280432 -1.329267
H -1.819845 4.599407 -1.396736
H 0.314188 5.206754 -0.312893
B -1.245668 -0.042977 0.063338
S 0.967719 0.469013 1.035257
O 1.851679 0.686162 2.181856
O -0.385369 -0.263999 1.345980
C 1.634434 -0.388847 -0.233588
C -2.707862 -0.612196 0.219914
C -3.172392 -1.115642 1.439764
C -3.602156 -0.605846 -0.859258
C -4.473338 -1.589730 1.578688
H -2.496560 -1.132521 2.293709
C -4.905169 -1.071419 -0.727103
H -3.267296 -0.238065 -1.830870
C -5.346514 -1.567218 0.496384
H -4.809101 -1.977914 2.539522
H -5.578655 -1.054148 -1.582985
H -6.364919 -1.937115 0.603508
P 3.237400 -0.928373 -0.064668
C 4.439049 0.405796 0.173169
H 5.455433 0.013288 0.289219
H 4.391634 1.082317 -0.686211
H 4.146951 0.950899 1.077336
C 3.686204 -1.807356 -1.573899
H 3.592366 -1.124448 -2.424360
H 4.711044 -2.187632 -1.507740
H 2.988588 -2.640148 -1.714897
C 3.519821 -2.065957 1.319136
H 3.210185 -1.541121 2.229972
H 2.882231 -2.946361 1.185674
H 4.570113 -2.369514 1.396348
Ge 0.094822 -1.020756 -1.309716
H 0.193316 -2.569104 -1.348363
H 0.309367 -0.552444 -2.770406

V
E = -3067.36886792
C 1.726471 2.305789 -0.087935
C -0.470509 1.231675 -0.118675

C -1.037187 2.515830 -0.268517
C -0.272127 3.662961 -0.315425
C 1.121479 3.538385 -0.211429
H 2.813416 2.269252 -0.026721
H -2.119098 2.592953 -0.367785
H -0.731585 4.641244 -0.436555
H 1.744682 4.432184 -0.240489
B -1.290728 -0.051121 -0.062185
C -2.833143 -0.208587 0.011062
C -3.643031 0.668711 0.753906
C -3.476829 -1.268097 -0.651476
C -5.021303 0.503507 0.821377
H -3.177564 1.481176 1.309599
C -4.856409 -1.421611 -0.613206
H -2.876171 -1.981870 -1.215422
C -5.633622 -0.535786 0.127864
H -5.622363 1.189480 1.416207
H -5.328434 -2.242736 -1.150274
H -6.714157 -0.661759 0.172856
C 0.970478 1.102882 -0.052995
C 1.541131 -0.207906 0.031319
P 3.200777 -0.544861 0.097788
C 4.202756 0.002681 -1.328377
H 4.090371 1.082919 -1.465066
H 5.263558 -0.238477 -1.193171
H 3.819510 -0.495138 -2.225373
C 4.097794 0.120171 1.541981
H 5.164647 -0.128726 1.503149
H 3.980066 1.207789 1.580629
H 3.648878 -0.303075 2.446650
C 3.454822 -2.332983 0.176840
H 4.523890 -2.566413 0.216707
H 2.955063 -2.727681 1.067525
H 3.007173 -2.797459 -0.708196
Ge 0.134426 -1.544163 -0.021967
H 0.185976 -2.486684 1.205683
H 0.287882 -2.490988 -1.240773

Pathway B

$^{Tos}Y_2Ge$
E = -5872.35282600
C 4.028632 2.657623 -0.430204
H 3.762236 3.151203 0.501172
C 3.145680 1.734618 -0.970732
C -3.928717 -1.827803 -1.155951
O 0.511229 1.889332 -0.958331
C -4.036123 -2.108779 -2.514860
H -3.138428 -2.145465 -3.129195
C 2.756923 -2.727672 2.908326
H 3.785450 -2.382218 2.993140
C 5.237017 2.897826 -1.073178
H 5.941902 3.607228 -0.640199
C -1.106131 5.120555 -0.521652
H -0.259921 5.762902 -0.288469
C -1.203190 3.879698 0.096556
H -0.409541 3.545403 0.759550
C 2.157604 -2.525389 -2.097804
H 1.406456 -1.738602 -2.187292
C 2.915816 -2.603872 -0.931072

C 5.342116 -0.781082 0.138576
H 5.352184 -1.387891 -0.764094
C 6.481067 -0.075469 0.512709
H 7.380160 -0.141969 -0.097588
C -2.058679 5.524480 -1.447839
H -1.973365 6.496281 -1.930410
C -2.080461 0.262891 -0.838483
Ge -0.388273 0.661388 -2.189857
O -2.426576 -1.649447 0.985743
S -2.321719 -1.472629 -0.471508
P -2.361982 1.384077 0.530479
C -5.290576 -2.351596 -3.052247
H -5.383987 -2.570326 -4.115208
C -4.056930 0.376260 2.487136
H -3.135557 0.062619 2.969390
C -0.480012 0.198213 2.242842
H -0.457391 -0.645523 1.571963
C 5.305875 0.828617 2.411480
H 5.284035 1.469856 3.290465
C 4.162062 0.137388 2.036491
H 3.235514 0.266237 2.595008
C -3.204991 3.428772 -1.175839
H -3.986712 2.752076 -1.509072
C 2.010939 -2.441664 1.765282
C 3.448390 1.066237 -2.152893
H 2.749147 0.330224 -2.549944
C 3.851990 -3.628024 -0.766836
H 4.423555 -3.700877 0.158765
C 4.181496 -0.691958 0.908182
C -0.527251 2.377192 3.981554
H -0.547635 3.228286 4.658813
C -1.246371 1.318823 1.937066
C -1.291890 2.400876 2.827289
H -1.921986 3.263270 2.617783
C -2.278457 3.038824 -0.202936
C -3.103774 4.670549 -1.784144
H -3.830070 4.960896 -2.540074
C 6.908433 2.446653 -2.900987
H 7.659098 1.763027 -2.478383
H 7.274965 3.467042 -2.742361
H 6.869166 2.259160 -3.979676
C 0.250654 1.259701 4.277784
H 0.842836 1.239309 5.191002
C -6.438419 -2.330930 -2.250182
C 6.468342 0.719384 1.653051
H 7.361203 1.270488 1.942848
C -6.425058 1.110457 1.229076
H -7.346962 1.401921 0.730227
C 1.425321 -0.292630 -0.063911
P 2.584974 -1.394242 0.385310
O 1.717153 2.110036 1.167340
S 1.660326 1.289875 -0.064474
O -1.360555 -2.273063 -1.224932
C 5.573401 2.232325 -2.253056
C 4.653536 1.325004 -2.789253
H 4.893863 0.801519 -3.714039
C 2.354393 -3.462162 -3.108321
H 1.757444 -3.405854 -4.016551
C 3.301738 -4.467989 -2.955391
H 3.454419 -5.197687 -3.748889

C 4.050726 -4.552905 -1.783041
H 4.785210 -5.346835 -1.660349
C -5.203444 1.449733 0.660606
H -5.205440 1.998254 -0.274016
C 0.259793 0.169823 3.420884
H 0.843544 -0.717122 3.658466
C -4.005723 1.090494 1.284689
C -6.467623 0.399461 2.422962
H -7.424936 0.131195 2.865788
C -6.293004 -2.066086 -0.887667
H -7.173188 -2.054250 -0.246243
C -5.280536 0.034140 3.048825
H -5.302083 -0.526254 3.981076
C 0.853448 -3.865598 3.861465
H 0.397880 -4.406857 4.688901
C 2.174622 -3.440198 3.954171
H 2.755267 -3.655239 4.849586
C -5.043747 -1.817413 -0.331040
H -4.928256 -1.620012 0.732035
C -7.785047 -2.608555 -2.846944
H -7.967905 -1.979169 -3.726410
H -8.588930 -2.429782 -2.125811
H -7.858957 -3.652478 -3.178944
C 0.115584 -3.602042 2.709115
H -0.920840 -3.922645 2.624235
C 0.695434 -2.903087 1.660240
H 0.120418 -2.685653 0.761068
H -2.856603 0.530740 -1.568521
H -1.234403 1.948526 -2.673205

^{Tos}YGeCl
E = -4436.40241434
C -3.166967 -0.001145 -0.963728
H -2.596379 -0.391009 -1.803444
C -2.569740 0.889363 -0.083077
O -0.822287 2.744411 0.078214
C 2.887757 -1.924337 -1.507138
H 2.589326 -2.926924 -1.199614
C -4.488496 -0.376112 -0.751356
H -4.959200 -1.077795 -1.438722
C 2.221218 -1.093815 2.538365
H 2.258282 -0.009843 2.441489
C 1.562206 -1.863544 1.574739
C -1.350076 -2.703075 0.168198
H -1.333071 -2.547317 1.246415
C -2.363642 -3.452747 -0.411412
H -3.133123 -3.899049 0.214900
C -1.431132 -3.026446 -2.595535
H -1.472257 -3.138901 -3.676880
C -0.405030 -2.284365 -2.019547
H 0.341794 -1.800577 -2.644668
C 2.198825 -0.803752 -1.031044
C -3.278147 1.423408 0.989510
H -2.800496 2.142399 1.651201
C 1.545827 -3.254855 1.685838
H 1.041462 -3.859310 0.934300
C -0.357897 -2.130768 -0.634015
C -6.652777 -0.257805 0.537566
H -7.324482 0.584491 0.324932
H -6.832289 -0.555999 1.577591

H -6.945766 -1.088754 -0.112158
C -2.404597 -3.613457 -1.794418
H -3.205129 -4.193561 -2.249663
C 0.118126 0.412476 0.733140
P 0.843873 -1.019319 0.144574
O -0.517863 0.906992 -1.689724
S -0.850715 1.289324 -0.314218
C -5.221815 0.131843 0.320915
C -4.594637 1.038515 1.184685
H -5.154351 1.455929 2.020718
C 2.839852 -1.716664 3.614250
H 3.348969 -1.114674 4.363911
C 2.813613 -3.103427 3.729775
H 3.300264 -3.587828 4.573989
C 2.170332 -3.870497 2.765206
H 2.152090 -4.954964 2.851279
C 4.344326 -0.469088 -2.755998
H 5.186789 -0.338489 -3.432834
C 3.958332 -1.753309 -2.372638
H 4.497600 -2.621322 -2.746601
C 3.660826 0.640918 -2.275350
H 3.953978 1.648451 -2.562698
C 2.583035 0.478036 -1.411248
H 2.051815 1.352609 -1.044885
Cl 2.024036 3.922765 -0.853115
Ge 0.765575 3.750752 1.032625
H -0.072242 5.070077 0.728458
H 0.158049 0.698562 1.778795

^{Tos}YGeHMDS
Not observed.

^{Tos}YGeB1
E = -4652.07317039
C 3.793080 1.213892 -0.025110
H 3.480015 1.905839 0.753313
C 2.831716 0.552417 -0.777854
O 0.705603 1.909239 -1.366818
C -0.145451 -2.512165 2.991586
H 0.445637 -3.401029 2.773287
C 5.135759 0.956806 -0.271859
H 5.895394 1.469345 0.316914
C -1.901628 -2.726367 -1.311347
H -2.045066 -1.679524 -1.578307
C -0.891920 -3.111585 -0.426214
C 2.359149 -3.521497 -0.401390
H 1.698254 -3.992593 -1.126713
C 3.708002 -3.853513 -0.376554
H 4.092583 -4.600254 -1.068422
C 4.074901 -2.261902 1.396043
H 4.747201 -1.752164 2.082860
C 2.723315 -1.939932 1.392330
H 2.341400 -1.167088 2.058923
C -0.380982 -1.560742 1.995172
C 3.193984 -0.344664 -1.774402
H 2.424277 -0.863696 -2.342748
C -0.764178 -4.451471 -0.046569
H 0.014524 -4.749403 0.653979
C 1.857858 -2.573849 0.494519
C 6.976414 -0.276035 -1.477805

H 7.224798 -0.279790 -2.545648
H 7.217698 -1.274723 -1.087642
H 7.631589 0.443562 -0.976433
C 4.566296 -3.223615 0.519787
H 5.625003 -3.476657 0.528123
C 0.181976 -0.510893 -0.718273
P 0.193489 -1.870903 0.309243
O 1.114716 1.157841 1.041222
S 1.113260 0.841868 -0.392002
C 5.529805 0.044393 -1.252578
C 4.538221 -0.592769 -2.005016
H 4.825363 -1.307217 -2.775416
C -2.749166 -3.693443 -1.841448
H -3.528566 -3.390125 -2.535569
C -2.610161 -5.027669 -1.478173
H -3.278596 -5.777416 -1.897138
C -1.622625 -5.406496 -0.573091
H -1.517770 -6.448893 -0.279588
C -1.391991 -1.168261 4.549688
H -1.785481 -1.010560 5.552251
C -0.654363 -2.315491 4.267894
H -0.470876 -3.054602 5.045010
C -1.619820 -0.220847 3.558087
H -2.183301 0.683511 3.773375
C -1.119232 -0.412842 2.274818
H -1.278956 0.332188 1.499180
N -3.201569 1.679693 0.200168
C -2.356045 2.989330 1.796296
C -3.333972 2.100305 1.519312
H -2.195255 3.555406 2.707094
H -4.154242 1.774576 2.148718
B -2.068089 2.359082 -0.412147
C -0.448701 4.043372 0.709063
C -0.198801 4.918759 -0.346110
C 0.410732 4.032168 1.808429
C 0.905106 5.759446 -0.306966
H -0.872554 4.921716 -1.197550
C 1.504746 4.887211 1.849130
H 0.239374 3.315055 2.607363
C 1.760834 5.752043 0.790156
H 1.092662 6.433209 -1.141074
H 2.170522 4.864709 2.710452
H 2.622406 6.416436 0.819585
C -3.956812 0.626164 -0.327842
C -4.328532 0.627805 -1.675984
C -4.350513 -0.447836 0.479655
C -5.068699 -0.423297 -2.202013
H -4.059215 1.477680 -2.298073
C -5.108309 -1.484719 -0.048551
H -4.043252 -0.473185 1.522619
C -5.472386 -1.479919 -1.391300
H -5.351588 -0.400634 -3.252842
H -5.404402 -2.311184 0.595014
H -6.068759 -2.292239 -1.802326
N -1.566029 3.182104 0.671844
H -0.212387 -0.577042 -1.730871
Ge -1.231114 1.868492 -2.297755
H -0.971859 3.350338 -2.801838

$^{Ph}Y_2Ge$
E = -4695.21551874
P 2.683237 0.425874 -0.023739
P -2.658418 0.344377 0.058047
C 1.322031 -0.670552 0.166870
C 1.605954 -2.129443 0.121789
C 2.245296 -2.779561 1.184991
H 2.540824 -2.203296 2.059571
C 2.471438 -4.149982 1.147707
H 2.968376 -4.635398 1.986142
C 2.041098 -4.905453 0.060985
H 2.199483 -5.981992 0.043430
C 1.387693 -4.275108 -0.992728
H 1.023032 -4.856364 -1.837299
C 1.184752 -2.901700 -0.966305
H 0.668196 -2.416611 -1.793111
C 3.760030 0.585382 1.419273
C 5.099551 0.951969 1.247681
H 5.506886 1.081997 0.247151
C 5.913877 1.141166 2.356387
H 6.956064 1.421854 2.218780
C 5.399399 0.965178 3.637764
H 6.041458 1.109112 4.504857
C 4.068564 0.601038 3.810130
H 3.662553 0.457626 4.809291
C 3.244295 0.412416 2.706384
H 2.202106 0.115417 2.848431
C 2.013411 2.060435 -0.414473
C 2.156804 3.139062 0.456861
H 2.684984 3.012284 1.399212
C 1.613124 4.374161 0.121565
H 1.723522 5.212966 0.805734
C 0.932030 4.537172 -1.080403
H 0.511883 5.506918 -1.340504
C 0.781073 3.459418 -1.948699
H 0.242951 3.575824 -2.886096
C 1.315133 2.221328 -1.615378
H 1.198545 1.383564 -2.300148
C 3.815115 -0.003148 -1.387567
C 4.340356 -1.302394 -1.417927
H 4.089077 -2.015750 -0.636579
C 5.188647 -1.686316 -2.447772
H 5.585416 -2.699226 -2.464017
C 5.527906 -0.784027 -3.451492
H 6.191930 -1.089429 -4.257851
C 5.022127 0.510343 -3.418335
H 5.293440 1.223958 -4.193876
C 4.170016 0.902995 -2.391491
H 3.789926 1.921233 -2.371115
C -1.661918 -0.812493 0.782068
C -1.854380 -2.237954 0.506934
C -1.348378 -3.211638 1.399540
H -0.830875 -2.873600 2.296163
C -1.469778 -4.568851 1.157067
H -1.049315 -5.274125 1.873333
C -2.115391 -5.039235 0.013505
H -2.206540 -6.106662 -0.177884
C -2.638974 -4.108289 -0.874953
H -3.152074 -4.441925 -1.776634
C -2.509811 -2.744661 -0.637526

H -2.924236 -2.054464 -1.371487
C -4.396593 -0.227281 -0.065056
C -4.846439 -1.094046 0.936389
H -4.147844 -1.416496 1.706679
C -6.158680 -1.548591 0.930645
H -6.496869 -2.228172 1.710438
C -7.032132 -1.148625 -0.076034
H -8.059369 -1.508748 -0.082545
C -6.587275 -0.295570 -1.079773
H -7.264391 0.013632 -1.873966
C -5.273520 0.162636 -1.077319
H -4.934092 0.817460 -1.875980
C -2.770658 1.914367 0.981600
C -1.884230 2.964525 0.732285
H -1.127117 2.859691 -0.039736
C -1.955526 4.133667 1.478526
H -1.256200 4.942676 1.275814
C -2.906516 4.262281 2.486090
H -2.960555 5.178100 3.072130
C -3.786856 3.217452 2.744201
H -4.531760 3.311080 3.531982
C -3.720703 2.048204 1.994302
H -4.414410 1.235397 2.198049
C -2.256828 0.909825 -1.658270
C -2.729340 2.100612 -2.218417
H -3.356328 2.766571 -1.627126
C -2.398150 2.442634 -3.524924
H -2.770413 3.372132 -3.952760
C -1.589755 1.599967 -4.284439
H -1.333307 1.867962 -5.307992
C -1.113073 0.414770 -3.733352
H -0.483096 -0.248705 -4.323552
C -1.443002 0.075077 -2.424607
H -1.073549 -0.847092 -1.977334
Ge -0.083759 -0.276716 1.878709
H -0.080785 1.278512 1.523975
H 0.649586 -0.365727 -0.642684

^{Ph}YGeCl
E = -3847.83096179
P -0.698781 0.206719 0.037597
C 0.742554 -0.232848 0.981099
C 1.988029 0.533463 0.710800
C 2.874457 0.773672 1.767959
H 2.625682 0.404480 2.762456
C 4.066318 1.455042 1.562264
H 4.739503 1.623711 2.401014
C 4.401930 1.918041 0.294140
H 5.335752 2.452455 0.132169
C 3.532069 1.685009 -0.764637
H 3.781031 2.036369 -1.764247
C 2.342924 0.998272 -0.559501
H 1.680186 0.830776 -1.407163
C -2.150414 -0.170325 1.053555
C -2.871117 0.836230 1.698596
H -2.594297 1.879954 1.568645
C -3.954088 0.501851 2.504264
H -4.515947 1.288267 3.003863
C -4.318733 -0.829970 2.667218
H -5.166805 -1.087582 3.298702

C -3.603740 -1.833201 2.019810
H -3.888713 -2.875869 2.143017
C -2.522975 -1.509237 1.210257
H -1.959826 -2.289674 0.699538
C -0.961699 -0.637866 -1.532594
C -2.250214 -0.643017 -2.079185
H -3.077437 -0.183929 -1.539262
C -2.476110 -1.247615 -3.308761
H -3.478081 -1.252997 -3.732822
C -1.423064 -1.854698 -3.987693
H -1.602988 -2.334551 -4.947923
C -0.147905 -1.864080 -3.433921
H 0.672867 -2.360933 -3.946370
C 0.090407 -1.259540 -2.204931
H 1.082762 -1.320090 -1.758903
C -0.740608 1.993455 -0.302144
C -0.244683 2.853612 0.683807
H 0.166083 2.446293 1.606002
C -0.248010 4.225843 0.476190
H 0.143705 4.888333 1.244990
C -0.732156 4.748315 -0.719645
H -0.725168 5.823849 -0.885202
C -1.211099 3.896139 -1.707803
H -1.578610 4.301409 -2.648265
C -1.215821 2.520405 -1.503583
H -1.578138 1.859581 -2.287141
Cl 2.794498 -2.423318 -0.470239
Ge 1.209112 -2.384232 1.191155
H 0.012103 -2.811083 0.201765
H 0.412467 -0.069412 2.014893

^{Ph}YGeHMDS
E = -4261.61023276
P 1.882968 0.219535 -0.013880
C 0.304698 0.064523 0.787260
C -0.153161 -1.358140 0.776502
C -0.047372 -2.174204 1.910464
H 0.347695 -1.757173 2.835461
C -0.449932 -3.504527 1.882146
H -0.363228 -4.112373 2.781080
C -0.953283 -4.058114 0.709563
H -1.260794 -5.101686 0.682060
C -1.077748 -3.257423 -0.421371
H -1.492795 -3.668806 -1.339628
C -0.705585 -1.920742 -0.380088
H -0.832592 -1.284597 -1.252761
C 3.219169 -0.611544 0.906849
C 4.380885 -1.039107 0.255793
H 4.477726 -0.917896 -0.821307
C 5.413208 -1.619805 0.981113
H 6.312579 -1.953054 0.467278
C 5.297376 -1.774499 2.359551
H 6.105746 -2.233309 2.925442
C 4.149452 -1.341260 3.013041
H 4.056246 -1.456508 4.090810
C 3.113518 -0.760498 2.290103
H 2.222525 -0.415570 2.808504
C 2.437318 1.942984 -0.144278
C 3.607263 2.353321 0.503594
H 4.199345 1.642309 1.072985

C 4.022324 3.677491 0.427048
H 4.933497 3.984058 0.936355
C 3.276916 4.603025 -0.293674
H 3.602774 5.639838 -0.350516
C 2.111328 4.202998 -0.937004
H 1.516140 4.923133 -1.493954
C 1.686874 2.882615 -0.862267
H 0.769050 2.584761 -1.361889
C 1.897357 -0.575912 -1.639389
C 2.040925 -1.967422 -1.682865
H 2.185463 -2.530309 -0.762947
C 1.975027 -2.636879 -2.896883
H 2.078492 -3.719445 -2.919845
C 1.763592 -1.925375 -4.073333
H 1.706279 -2.451183 -5.024442
C 1.626069 -0.542468 -4.035331
H 1.461503 0.017487 -4.953123
C 1.692322 0.134212 -2.822669
H 1.584077 1.213644 -2.806541
N -2.782817 0.480255 0.150706
Si -3.720016 -0.076629 -1.214392
Si -3.545023 0.659310 1.718465
C -2.824059 0.079136 -2.864692
H -2.579814 1.122479 -3.095711
H -1.892987 -0.496464 -2.927472
H -3.503783 -0.292686 -3.644489
C -4.287357 -1.875120 -1.043674
H -5.290065 -1.938005 -0.604360
H -4.329015 -2.360304 -2.028378
H -3.608074 -2.452017 -0.408144
C -5.291478 0.956445 -1.402904
H -5.865008 0.639923 -2.284808
H -5.948692 0.853417 -0.529290
H -5.052057 2.022042 -1.514635
C -4.828421 -0.680903 2.033612
H -5.220033 -0.575039 3.054249
H -5.679562 -0.616689 1.344749
H -4.390657 -1.682268 1.937450
C -4.378575 2.339804 1.890596
H -4.826567 2.470659 2.884975
H -3.635411 3.134501 1.743160
H -5.166630 2.476882 1.140121
C -2.267089 0.541932 3.098382
H -1.527923 1.350547 3.024196
H -2.763908 0.632591 4.073809
H -1.741429 -0.419676 3.071214
H 0.463115 0.427041 1.811664
Ge -1.182238 1.573946 0.033384
H -0.783221 1.193882 -1.484549

^{Ph}YGeB1
E = -4063.50731655
P 1.801835 0.361827 -0.045811
C 0.587322 -0.800337 -0.653896
C 0.187616 -1.738861 0.441914
C -0.608403 -1.340076 1.523358
H -1.040612 -0.343926 1.529971
C -0.867755 -2.206457 2.577538
H -1.487150 -1.867809 3.406181
C -0.351319 -3.499567 2.571777

H -0.557589 -4.177841 3.397658
C 0.411421 -3.921653 1.488686
H 0.803323 -4.936550 1.456319
C 0.675032 -3.051533 0.438788
H 1.281117 -3.388442 -0.401199
C 1.310714 1.445861 1.320756
C 2.291753 1.826131 2.245229
H 3.296692 1.415766 2.179584
C 1.987634 2.732090 3.254308
H 2.757805 3.021158 3.966459
C 0.706268 3.262615 3.350735
H 0.469663 3.973677 4.140164
C -0.272302 2.885664 2.436878
H -1.276908 3.296783 2.496655
C 0.024991 1.985784 1.422011
H -0.735466 1.709244 0.696153
C 2.427871 1.378513 -1.398612
C 2.933135 2.654402 -1.141538
H 2.907331 3.058538 -0.131800
C 3.451695 3.416559 -2.180310
H 3.837958 4.413123 -1.977173
C 3.466582 2.909853 -3.476278
H 3.867850 3.510624 -4.290203
C 2.961279 1.640820 -3.734613
H 2.958467 1.248189 -4.748993
C 2.440592 0.870385 -2.701243
H 2.026954 -0.110864 -2.918827
C 3.226641 -0.572497 0.592293
C 3.076226 -1.302490 1.778992
H 2.151373 -1.235439 2.350789
C 4.108782 -2.113716 2.228865
H 3.983454 -2.679950 3.149337
C 5.293263 -2.206351 1.503509
H 6.100425 -2.843953 1.859101
C 5.446107 -1.483734 0.325721
H 6.372462 -1.550717 -0.241185
C 4.416117 -0.668670 -0.132299
H 4.540458 -0.103225 -1.053597
N -3.088533 -1.422938 -0.456477
C -4.413699 0.023444 0.620683
C -4.205714 -1.280831 0.358110
H -5.240176 0.476701 1.154702
H -4.822370 -2.125600 0.640489
B -2.517241 -0.106345 -0.715277
N -3.432967 0.784876 -0.012260
C -3.370753 2.175758 0.136953
C -3.798395 2.791909 1.319512
C -2.874288 2.976798 -0.896747
C -3.725158 4.172531 1.462914
H -4.170197 2.180272 2.138312
C -2.780239 4.352281 -0.734739
H -2.582655 2.512234 -1.834573
C -3.204434 4.961319 0.442543
H -4.066586 4.632028 2.389234
H -2.384241 4.956087 -1.549134
H -3.139058 6.041051 0.559079
C -2.597683 -2.690693 -0.810279
C -1.932543 -2.884018 -2.025184
C -2.762443 -3.783093 0.046240
C -1.422290 -4.131797 -2.355194

H -1.824093 -2.048546 -2.713108
C -2.267296 -5.033195 -0.301434
H -3.247091 -3.643077 1.009128
C -1.584904 -5.216423 -1.498167
H -0.905541 -4.258636 -3.305235
H -2.398659 -5.867278 0.385895
H -1.189575 -6.194807 -1.763865
H 1.176373 -1.378033 -1.380442
Ge -0.819441 0.280464 -1.901335
H -0.380975 1.684785 -1.295128

I
E = -3097.32626955
C 1.651092 2.336652 0.010224
C -0.489004 1.231229 -0.205966
C -1.087048 2.471354 -0.412545
C -0.329981 3.642290 -0.382882
C 1.037007 3.576144 -0.158839
H 2.729138 2.307413 0.169611
H -2.154743 2.524279 -0.608728
H -0.819157 4.601516 -0.539749
H 1.636055 4.484179 -0.123865
N -1.165883 0.005310 -0.228551
C -2.540395 -0.162106 -0.054448
C -3.304085 0.655658 0.792861
C -3.182022 -1.234870 -0.689734
C -4.658308 0.412375 0.974487
H -2.821491 1.471144 1.324614
C -4.534135 -1.479842 -0.490554
H -2.604167 -1.872232 -1.358232
C -5.286716 -0.653327 0.336321
H -5.228109 1.059648 1.639518
H -5.004790 -2.320261 -0.998154
H -6.348190 -0.838648 0.485711
C 0.923202 1.141631 -0.024780
C 1.461101 -0.209984 0.071856
P 3.097713 -0.604027 0.158827
C 4.139983 -0.013851 -1.218374
H 4.019293 1.069876 -1.318687
H 5.198701 -0.250060 -1.061064
H 3.785139 -0.480824 -2.143127
C 3.980453 -0.021401 1.645384
H 5.043193 -0.288792 1.617573
H 3.882148 1.066502 1.721094
H 3.503693 -0.466956 2.524663
C 3.286257 -2.403794 0.167468
H 4.344473 -2.679133 0.223472
H 2.758220 -2.820099 1.032250
H 2.854022 -2.818060 -0.750294
Ge 0.027773 -1.430700 -0.137473
H -0.388313 -2.355791 1.029535
H 0.017238 -2.317001 -1.412427

II
E = -3646.45487748
C -0.757405 3.469557 0.261015
C -0.474801 2.119998 0.087234
C -1.443612 1.181964 -0.306668
C -2.756055 1.652787 -0.452453
C -3.051505 2.994593 -0.264083

C -2.057612 3.914589 0.071533
H 0.044922 4.138360 0.564241
H -3.530972 0.946576 -0.742826
H -4.076195 3.336260 -0.401358
H -2.299801 4.966955 0.198985
N -1.074178 -0.122675 -0.606814
S 1.075141 1.526584 0.680974
O 1.966311 2.665939 0.898482
O 0.774378 0.644436 1.829438
C -1.772422 -1.183944 -0.038111
C -2.270041 -1.089035 1.273548
C -1.940058 -2.398731 -0.721093
C -2.918758 -2.163669 1.864840
H -2.107109 -0.167547 1.828994
C -2.575697 -3.473585 -0.112216
H -1.569519 -2.486402 -1.740580
C -3.075908 -3.366975 1.181518
H -3.292257 -2.063214 2.882969
H -2.694994 -4.403265 -0.666917
H -3.580757 -4.208355 1.651225
C 1.788253 0.442827 -0.539582
H 2.524504 1.011962 -1.118244
P 2.579487 -0.909707 0.308424
C 3.648034 -0.388734 1.667413
H 4.342363 0.374247 1.299471
H 3.014463 0.051930 2.442580
H 4.207163 -1.243540 2.064032
C 3.629142 -1.715471 -0.918317
H 4.111298 -2.594219 -0.476547
H 2.998180 -2.020513 -1.761390
H 4.395540 -1.016960 -1.270507
C 1.401535 -2.130229 0.900555
H 1.942696 -2.933957 1.411907
H 0.703491 -1.634529 1.581408
H 0.847288 -2.536904 0.046798
Ge 0.353537 -0.381104 -1.940487
H 0.167160 1.078762 -2.527082

III
E = -4408.58161691
C 0.437567 1.767939 0.503143
C -0.370958 1.723489 -0.633474
C -0.727769 2.920057 -1.254022
C -0.323719 4.143391 -0.738009
C 0.461196 4.183924 0.407982
C 0.843482 2.999768 1.021653
H -1.345887 2.854480 -2.146259
H -0.621684 5.064973 -1.234437
H 0.782208 5.135186 0.827092
H 1.472920 3.007463 1.907885
S 0.951203 0.402869 1.578114
S -0.930714 0.204728 -1.480979
O 2.186617 0.900075 2.218884
O -0.176539 0.077882 2.448966
O 0.315245 -0.238148 -2.154830
O -2.033882 0.713598 -2.320645
C -1.397823 -0.912075 -0.353645
P -2.886118 -0.608925 0.425593
P 2.831881 -0.739255 -0.427100
C -3.035519 -1.597547 1.922709

H -3.980682 -1.360811 2.423854
H -2.998624 -2.662144 1.674380
H -2.177679 -1.353638 2.559237
C -3.123350 1.111222 0.955138
H -3.084960 1.755848 0.070444
H -4.090140 1.231193 1.456844
H -2.305328 1.368337 1.636555
C -4.324949 -0.963653 -0.621540
H -4.323597 -2.029992 -0.871040
H -5.270819 -0.690437 -0.139519
H -4.180000 -0.389659 -1.543962
C 2.848304 -1.908593 -1.790367
H 3.751622 -1.744610 -2.389146
H 2.824232 -2.930313 -1.400418
H 1.940295 -1.730570 -2.378985
C 2.980614 0.919782 -1.110448
H 3.044782 1.641315 -0.290538
H 3.901690 0.946486 -1.703763
H 2.113629 1.118681 -1.746385
C 4.327386 -0.987929 0.558372
H 5.229791 -0.790071 -0.030384
H 4.264010 -0.299742 1.409461
H 4.352505 -2.018401 0.929392
Ge -0.080985 -2.410786 -0.065487
H -0.502996 -2.656890 1.459283
C 1.426393 -1.034328 0.644661
H 1.825300 -1.640285 1.472485

IV
E = -3616.56412120
C 0.460452 3.491562 0.465307
C 0.104197 2.144795 0.471244
C -1.032747 1.577199 -0.134073
C -1.883412 2.500832 -0.771257
C -1.579111 3.849627 -0.771550
C -0.416206 4.356931 -0.160472
H 1.370702 3.838486 0.950267
H -2.793962 2.147941 -1.252463
H -2.262974 4.547576 -1.252521
H -0.214569 5.425410 -0.175525
B -1.078963 0.006548 0.021363
S 1.046356 0.816305 1.018535
O 1.955300 0.980711 2.152111
O -0.082416 -0.210586 1.250313
C -2.433663 -0.759815 0.252821
C -2.748187 -1.369336 1.473962
C -3.389263 -0.846351 -0.770449
C -3.958189 -2.028368 1.667743
H -2.028011 -1.320635 2.289952
C -4.605181 -1.492475 -0.581154
H -3.168754 -0.409585 -1.746030
C -4.895845 -2.089603 0.642195
H -4.173578 -2.493922 2.629008
H -5.326934 -1.540484 -1.395658
H -5.844249 -2.602831 0.792490
C 1.823666 0.153591 -0.448002
H 2.306715 0.968511 -0.996080
P 2.968154 -1.134624 -0.027189
C 4.419560 -0.551626 0.882763
H 4.976342 0.154545 0.256792

H 4.073107 -0.035861 1.783712
H 5.070868 -1.392281 1.147307
C 3.581207 -1.916269 -1.527802
H 4.331710 -2.671592 -1.266381
H 2.727357 -2.361956 -2.049983
H 4.034371 -1.153903 -2.171064
C 2.128324 -2.348739 0.997162
H 2.780616 -3.213370 1.157438
H 1.872227 -1.882491 1.954670
H 1.205708 -2.647080 0.484106
H 0.203316 0.510799 -2.586051
Ge 0.204817 -0.754195 -1.621861

V
E = -3067.36305632
C -1.715411 2.374961 -0.186940
C 0.427134 1.237698 -0.014369
C 1.001840 2.465489 0.351243
C 0.243582 3.622717 0.458743
C -1.121036 3.575132 0.187318
H -2.775862 2.371750 -0.429535
H 2.066295 2.498397 0.579610
H 0.708233 4.560760 0.757862
H -1.725469 4.478181 0.257878
B 1.194272 -0.125233 -0.080276
C 2.745177 -0.236782 -0.048420
C 3.562866 0.649802 -0.772867
C 3.392826 -1.262600 0.662749
C 4.946431 0.517548 -0.791426
H 3.099503 1.449590 -1.350530
C 4.776138 -1.383829 0.671085
H 2.788739 -1.973435 1.226488
C 5.559749 -0.495353 -0.060714
H 5.551094 1.211530 -1.373759
H 5.248270 -2.181576 1.242737
H 6.644043 -0.594684 -0.064224
C -0.960525 1.202946 -0.293756
P -3.120044 -0.558947 -0.057894
C -3.122926 -0.384724 1.734426
H -2.937727 0.665806 1.985028
H -4.082327 -0.706142 2.152235
H -2.303505 -0.999531 2.125996
C -4.576353 0.360446 -0.659763
H -5.493781 -0.146009 -0.338502
H -4.584341 1.381912 -0.269851
H -4.553218 0.398097 -1.754504
C -3.496037 -2.270068 -0.488876
H -4.492295 -2.539879 -0.122349
H -3.463330 -2.380253 -1.578655
H -2.729477 -2.914126 -0.043891
Ge -0.166721 -1.622016 0.032276
H -0.066838 -2.443345 -1.306328
C -1.531871 -0.127185 -0.695067
H -1.523128 -0.299364 -1.778941

Pathway C

IV
E = -3616.57722538
C 0.385038 3.274442 0.670443

C -0.023618 1.957738 0.557313
C -1.237127 1.529616 0.028048
C -2.114950 2.522298 -0.400138
C -1.751078 3.862797 -0.291686
C -0.515285 4.241643 0.235961
H 1.354095 3.536782 1.089249
H -3.086592 2.251587 -0.810784
H -2.445080 4.634806 -0.620046
H -0.258638 5.295963 0.314955
B -1.300631 -0.077850 0.066042
S 0.934066 0.539709 0.985024
O 1.650540 0.612244 2.262816
O -0.323987 -0.435575 1.130641
C 1.812536 0.012309 -0.319795
C -2.663232 -0.866287 0.162230
C -2.972065 -1.637547 1.287623
C -3.616635 -0.794503 -0.860917
C -4.187637 -2.306419 1.392247
H -2.242710 -1.707092 2.093580
C -4.834376 -1.457595 -0.762869
H -3.396562 -0.217416 -1.760188
C -5.123643 -2.216940 0.367459
H -4.406942 -2.900125 2.278646
H -5.559640 -1.387616 -1.572245
H -6.075676 -2.739249 0.446937
P 3.073422 -1.064628 0.156942
C 4.430326 -0.215950 0.997982
H 5.213382 -0.922783 1.294433
H 4.841359 0.539855 0.321012
H 4.011457 0.280240 1.878930
C 3.774373 -1.841123 -1.311359
H 4.139406 -1.064080 -1.990823
H 4.600096 -2.498658 -1.018597
H 2.997306 -2.415006 -1.826672
C 2.557384 -2.404944 1.263839
H 2.131689 -1.946537 2.163447
H 1.776095 -2.988319 0.764861
H 3.397607 -3.053488 1.536276
Ge 0.773175 -0.282974 -2.031422
H 0.337593 1.230172 -2.176250
H -0.745590 -0.521097 -1.091864

V
E = -3067.36728974
C -1.703673 2.296111 0.068870
C 0.444651 1.161358 -0.200908
C 1.045166 2.428457 -0.268544
C 0.305652 3.598238 -0.198838
C -1.079031 3.526253 -0.034030
H -2.781368 2.272779 0.229634
H 2.123941 2.485783 -0.409719
H 0.797697 4.565926 -0.278419
H -1.671105 4.438368 0.026586
B 1.274190 -0.159655 -0.379300
C 2.829846 -0.280719 -0.196417
C 3.611907 -1.079337 -1.042943
C 3.499883 0.406323 0.827902
C 4.988390 -1.189898 -0.880783
H 3.126210 -1.624856 -1.852253
C 4.875288 0.301872 0.999502

H 2.924190 1.031641 1.510413
C 5.626762 -0.497778 0.143350
H 5.567465 -1.816893 -1.557523
H 5.365095 0.845084 1.806636
H 6.704581 -0.580873 0.273995
C -0.971063 1.092682 -0.004396
C -1.542904 -0.238735 0.121768
P -3.214887 -0.564927 -0.043129
C -4.276877 0.030903 1.312435
H -4.177006 1.115053 1.419666
H -5.327851 -0.222936 1.131943
H -3.936714 -0.437115 2.242361
C -3.941467 0.124959 -1.558942
H -5.022529 -0.048446 -1.599153
H -3.736923 1.199464 -1.600154
H -3.454578 -0.348895 -2.417642
C -3.546159 -2.340027 -0.112079
H -4.625700 -2.508261 -0.189273
H -3.037585 -2.773265 -0.978243
H -3.165361 -2.821692 0.794186
Ge -0.155722 -1.493801 0.361157
H 0.814455 -0.830341 -1.346157
H -0.649671 -2.910684 -0.076174

5.2.5 Phenol coordinated species

Pathway A

I
E = -3404.13958251
C -0.663679 2.089351 1.967787
C 1.258242 1.038246 0.929816
C 1.995052 1.253264 2.099786
C 1.400481 1.869541 3.190185
C 0.068994 2.283471 3.128203
H -1.694558 2.440802 1.933094
H 3.035810 0.943426 2.146672
H 1.981698 2.035958 4.095095
H -0.393246 2.768711 3.985687
N 1.734342 0.446622 -0.228924
C 3.003173 -0.167175 -0.283625
C 3.346502 -1.180937 0.619420
C 3.912739 0.185824 -1.282225
C 4.576525 -1.816816 0.524915
H 2.628718 -1.471055 1.385585
C 5.137921 -0.464497 -1.380216
H 3.645212 0.978713 -1.977909
C 5.478253 -1.463645 -0.475713
H 4.827672 -2.605137 1.232408
H 5.834666 -0.179746 -2.166647
H 6.439895 -1.967269 -0.550227
C -0.095375 1.468722 0.842610
C -0.697381 1.203734 -0.442741
P -2.348288 1.509272 -0.792105
C -3.536260 0.728012 0.333098
H -3.293914 0.989426 1.367238
H -4.556489 1.052941 0.099542
H -3.459709 -0.358786 0.226957
C -2.789590 3.274191 -0.809383
H -3.854588 3.419935 -1.023221

H -2.547810 3.715318 0.163207
H -2.183182 3.774373 -1.571664
C -2.766584 0.885295 -2.435431
H -3.801598 1.144542 -2.681638
H -2.090705 1.319220 -3.179772
H -2.650531 -0.203740 -2.436788
Ge 0.513213 0.434197 -1.707125
H 0.374672 -1.367393 0.388502
O -0.089916 -1.758373 1.141820
C -1.308882 -2.183515 0.780496
C -2.243027 -2.425580 1.791343
C -1.670146 -2.410471 -0.552231
C -3.516013 -2.878318 1.470724
H -1.944787 -2.244291 2.821208
C -2.949497 -2.859727 -0.861464
H -0.936317 -2.244413 -1.339887
C -3.883744 -3.092879 0.143880
H -4.232577 -3.062272 2.269508
H -3.212251 -3.040166 -1.903029
H -4.881870 -3.448371 -0.101813

II
Not observed.

III
E = -4715.39303935
C 1.447790 -1.574912 0.996895
C 2.461309 -1.232680 0.097194
C 3.672494 -1.921865 0.132955
C 3.884191 -2.936627 1.057004
C 2.881660 -3.267520 1.960534
C 1.670991 -2.588041 1.928066
H 4.446802 -1.629141 -0.571600
H 4.835132 -3.465694 1.069979
H 3.038568 -4.056069 2.694020
H 0.864863 -2.836845 2.614008
S -0.191346 -0.790706 1.115908
S 2.356713 0.076865 -1.162931
O -1.010859 -1.841064 1.764843
O 0.018767 0.445381 1.892793
O 1.567985 -0.506351 -2.287984
O 3.772903 0.404056 -1.403580
C -0.795188 -0.498242 -0.427689
C 1.458908 1.358377 -0.610636
P 2.149181 2.452790 0.493069
P -1.533903 -1.856411 -1.169952
C 0.849117 3.513143 1.144030
H 1.257261 4.218620 1.875316
H 0.394121 4.067214 0.315601
H 0.093071 2.864983 1.599453
C 2.967501 1.649412 1.892995
H 3.771100 1.017346 1.496924
H 3.388886 2.392142 2.579699
H 2.219965 1.028105 2.397410
C 3.421773 3.505384 -0.254969
H 2.959077 4.124887 -1.030356
H 3.925514 4.138727 0.484327
H 4.137169 2.827283 -0.734625
C -1.813134 -1.461989 -2.910233
H -2.297881 -2.307757 -3.409514

H -2.450133 -0.574334 -2.986709
H -0.843933 -1.251965 -3.377653
C -0.464709 -3.318060 -1.168453
H -0.313997 -3.633383 -0.130572
H -0.904529 -4.136831 -1.748806
H 0.496314 -3.009749 -1.598333
C -3.126089 -2.354937 -0.480050
H -3.517755 -3.250468 -0.976146
H -2.968142 -2.534804 0.587908
H -3.824293 -1.518457 -0.593151
H -2.263811 2.873174 -0.499520
O -2.121578 2.381676 0.316801
C -3.210004 1.603200 0.556797
C -3.215796 0.809422 1.702476
C -4.297969 1.587463 -0.313462
C -4.332091 0.035945 1.985611
H -2.336582 0.796911 2.339482
C -5.414070 0.810927 -0.013627
H -4.276975 2.199226 -1.215855
C -5.440862 0.037345 1.140750
H -4.328367 -0.584388 2.879808
H -6.267016 0.818529 -0.690058
H -6.313790 -0.567249 1.376849
Ge -0.320261 1.089429 -1.456324

IV
E = -3923.37778191
C 0.443274 1.327380 2.786654
C -0.249190 0.588234 1.844683
C -1.221690 1.076634 0.966413
C -1.456472 2.453223 1.025102
C -0.783102 3.237044 1.959943
C 0.149914 2.687314 2.845497
H 1.162903 0.860341 3.456308
H -2.198934 2.908511 0.371526
H -1.000358 4.302568 2.017155
H 0.642329 3.320644 3.580486
B -1.879971 -0.139026 0.156386
S -0.087947 -1.128129 1.486745
O 0.455645 -1.927018 2.588806
O -1.591843 -1.339239 1.139193
C 0.678381 -1.319194 -0.017325
C -3.392575 -0.081870 -0.275757
C -4.349197 -0.973041 0.224581
C -3.832263 0.889471 -1.187134
C -5.683921 -0.894960 -0.161005
H -4.034966 -1.738842 0.932763
C -5.164875 0.978866 -1.571243
H -3.108312 1.585780 -1.614924
C -6.099447 0.083562 -1.058377
H -6.407097 -1.601710 0.244562
H -5.476940 1.745785 -2.279241
H -7.143821 0.146433 -1.359712
P 2.183693 -2.142666 -0.006181
C 3.430801 -1.349932 1.035847
H 4.378056 -1.900341 1.019967
H 3.581465 -0.325804 0.676718
H 3.037609 -1.323568 2.058540
C 2.833498 -2.199737 -1.684171
H 3.038507 -1.178170 -2.017373

H 3.752189 -2.795941 -1.707221
H 2.076276 -2.640116 -2.341059
C 2.073336 -3.856452 0.570790
H 1.634370 -3.835520 1.573766
H 1.403140 -4.406972 -0.097828
H 3.058099 -4.336822 0.594176
C 4.255045 1.562493 -1.829712
C 4.543335 2.018089 -0.548086
C 3.495655 2.380889 0.297485
C 2.177125 2.287613 -0.124451
C 1.898415 1.837287 -1.412082
C 2.936113 1.468443 -2.266263
H 5.061410 1.282183 -2.505171
H 5.575050 2.097820 -0.213058
H 3.704294 2.742852 1.302731
H 1.349406 2.570735 0.517542
H 2.709475 1.119115 -3.273643
O 0.595710 1.770017 -1.782181
H 0.526910 1.480669 -2.699878
Ge -0.469953 -0.701020 -1.462786

V
E = -1585.88683398
C 0.946310 2.885137 0.861177
C -1.108832 1.692716 0.346729
C -1.804272 2.629105 1.125740
C -1.156830 3.669035 1.778779
C 0.226260 3.775257 1.647601
H 2.022897 3.025616 0.780423
H -2.886641 2.539749 1.209056
H -1.712883 4.384104 2.380687
H 0.757657 4.576533 2.160348
B -1.763379 0.516330 -0.389667
C -3.169949 -0.087953 -0.216369
C -3.870004 -0.035439 1.004036
C -3.792669 -0.748088 -1.290817
C -5.132103 -0.597640 1.136996
H -3.400783 0.431292 1.868281
C -5.067401 -1.283974 -1.173399
H -3.257220 -0.835816 -2.235687
C -5.738416 -1.211795 0.044135
H -5.647953 -0.558950 2.094830
H -5.533732 -1.776824 -2.024503
H -6.731101 -1.647339 0.145939
C 0.311014 1.833999 0.174534
C 0.977972 0.886397 -0.707843
P 2.672771 0.831786 -0.947773
C 3.394236 2.323972 -1.710884
H 3.169693 3.204869 -1.101786
H 4.480361 2.226597 -1.821363
H 2.932820 2.462195 -2.694454
C 3.652748 0.533178 0.547965
H 4.725464 0.610064 0.337100
H 3.377311 1.250690 1.326946
H 3.411715 -0.472754 0.908580
C 3.122097 -0.509134 -2.070050
H 4.201071 -0.479971 -2.256536
H 2.853743 -1.463343 -1.605412
H 2.574882 -0.402772 -3.011991
H 0.321535 -1.833102 -1.711223

O 0.048545 -2.314575 -0.452769
C 0.959506 -2.321488 0.511572
C 0.785254 -1.529523 1.662300
C 2.108410 -3.129287 0.432686
C 1.682525 -1.602861 2.717049
H -0.082444 -0.872988 1.708799
C 3.011114 -3.184067 1.489179
H 2.248106 -3.743625 -0.455711
C 2.802976 -2.430423 2.641841
H 1.512006 -0.997094 3.605909
H 3.882381 -3.833655 1.414803
H 3.506687 -2.483733 3.469843
Ge -0.225884 -0.287323 -1.575735

Pathway B

I
E = -3404.14113528
C 1.740409 1.197867 2.066555
C -0.123581 -0.193450 1.370326
C -0.907165 0.375879 2.381028
C -0.377357 1.358802 3.203511
C 0.944421 1.778631 3.044066
H 2.775973 1.524171 1.971016
H -1.935833 0.050862 2.511041
H -0.998619 1.798995 3.981149
H 1.356661 2.550306 3.691119
N -0.539110 -1.162558 0.478217
C -1.889483 -1.563170 0.384362
C -2.220344 -2.918127 0.453774
C -2.906207 -0.626992 0.161830
C -3.538991 -3.330933 0.296417
H -1.428680 -3.642474 0.635744
C -4.221679 -1.044447 0.019080
H -2.653141 0.427620 0.085744
C -4.546276 -2.397181 0.083331
H -3.779125 -4.391398 0.349777
H -4.997606 -0.302000 -0.159433
H -5.578577 -2.720658 -0.034753
C 1.236922 0.201264 1.213591
P 3.508651 -0.157384 -0.370135
C 3.758101 1.558043 -0.921019
H 3.545702 2.243651 -0.094170
H 4.782993 1.719699 -1.273677
H 3.044984 1.764519 -1.727363
C 4.807740 -0.461410 0.865565
H 5.798841 -0.203860 0.475047
H 4.604091 0.129877 1.763990
H 4.779011 -1.521118 1.140064
C 3.943629 -1.185431 -1.791204
H 4.971457 -0.975737 -2.105074
H 3.849073 -2.241907 -1.519322
H 3.254929 -0.972304 -2.615337
C 1.913568 -0.501855 0.141451
H 0.813009 1.025638 -1.023538
Ge 0.782334 -1.789816 -0.740524
O 0.338109 1.354340 -1.803235
C -0.805432 1.973925 -1.427277
C -1.877808 1.972070 -2.319404
C -0.935688 2.607580 -0.190263

C -3.074849 2.580957 -1.965639
H -1.757364 1.467826 -3.275226
C -2.141226 3.205313 0.157045
H -0.100924 2.607901 0.508860
C -3.218391 3.195154 -0.723875
H -3.909271 2.563877 -2.664728
H -2.234137 3.677180 1.133559
H -4.160902 3.662437 -0.446860

II
E = -3953.32866297
C 0.181404 1.797302 2.564939
C 0.160575 0.685881 1.728212
C -1.001902 0.266463 1.060986
C -2.169638 1.010291 1.301057
C -2.157439 2.106700 2.144161
C -0.983123 2.516201 2.777422
H 1.122883 2.065813 3.038964
H -3.082359 0.723837 0.784585
H -3.077264 2.669748 2.291163
H -0.980373 3.385102 3.431334
N -1.002722 -0.795852 0.159726
S 1.637075 -0.258747 1.551967
O 2.751953 0.476401 2.186879
O 1.406271 -1.627555 2.025018
C -2.164594 -1.601161 0.118925
C -2.652177 -2.170463 1.302042
C -2.821938 -1.876174 -1.082326
C -3.774890 -2.983462 1.279123
H -2.123036 -1.971511 2.231730
C -3.935180 -2.710811 -1.102114
H -2.460621 -1.422268 -2.002492
C -4.421268 -3.262547 0.076356
H -4.140677 -3.418152 2.207755
H -4.433395 -2.915481 -2.048057
H -5.297943 -3.906751 0.060714
C 1.852379 -0.255454 -0.168612
Ge 0.315812 -1.200736 -1.167933
O 0.450168 0.936438 -1.809746
C -0.514519 1.861864 -1.633675
C -1.784121 1.665453 -2.181485
C -0.262693 3.009880 -0.876571
C -2.789159 2.597859 -1.957819
H -1.966847 0.774707 -2.778668
C -1.273056 3.937943 -0.664602
H 0.725127 3.151002 -0.441372
C -2.542860 3.737581 -1.197835
H -3.777069 2.430061 -2.383751
H -1.066416 4.822164 -0.064097
H -3.333577 4.464227 -1.024393
H 1.249859 0.761089 -0.928413
P 3.508686 -0.507444 -0.610164
C 3.562719 -0.891642 -2.373079
H 4.596730 -0.904806 -2.733145
H 3.094810 -1.865253 -2.553631
H 2.990296 -0.124350 -2.908361
C 4.278392 -1.853339 0.315294
H 3.693539 -2.766221 0.162943
H 5.320448 -2.008178 0.014955
H 4.222447 -1.584433 1.376616

C 4.519442 0.964657 -0.348749
H 5.573495 0.766817 -0.572954
H 4.143682 1.767392 -0.992049
H 4.390574 1.251149 0.700283

III
E = -4715.38070458
C -2.810672 0.734030 -0.375220
C -2.114640 1.506145 0.558950
C -2.745852 2.607437 1.135624
C -4.046272 2.947428 0.784067
C -4.734437 2.180486 -0.145719
C -4.113261 1.081155 -0.724429
H -2.192706 3.188494 1.868686
H -4.519385 3.811297 1.246566
H -5.754397 2.435386 -0.426470
H -4.624377 0.461683 -1.457015
S -2.106799 -0.649060 -1.310048
S -0.489787 1.145784 1.298200
O -3.291781 -1.356659 -1.815744
O -1.210680 0.050109 -2.296079
O -0.713177 0.003937 2.192545
O -0.125407 2.431655 1.931421
C -1.050598 -1.553038 -0.416784
P 1.491973 2.202235 -0.525793
P -1.623573 -2.700019 0.705419
C 2.655081 1.707099 -1.815654
H 3.168550 2.601318 -2.186323
H 3.389884 1.007971 -1.396813
H 2.123788 1.215911 -2.636654
C 0.253471 3.267232 -1.301333
H -0.372066 3.693626 -0.509525
H 0.720083 4.072307 -1.879119
H -0.368132 2.634313 -1.947369
C 2.452861 3.226515 0.612141
H 3.307600 2.640886 0.964930
H 2.810505 4.121368 0.089786
H 1.811027 3.493759 1.455571
C -0.330233 -3.142572 1.870851
H -0.697520 -3.903560 2.567656
H 0.538839 -3.515327 1.320165
H -0.031870 -2.229122 2.394629
C -3.056285 -2.090976 1.626063
H -3.833538 -1.824585 0.899831
H -3.437703 -2.857251 2.309778
H -2.740596 -1.199670 2.176664
C -2.199914 -4.220031 -0.099982
H -2.652935 -4.921808 0.609868
H -2.932507 -3.913796 -0.855928
H -1.354221 -4.695668 -0.607845
C 0.726725 0.777986 0.133485
H 1.442392 -0.188963 0.765059
O 1.974017 -1.313125 0.818440
C 3.267522 -1.077917 0.594540
C 3.938107 -0.082310 1.323783
C 3.969700 -1.750040 -0.417697
C 5.259252 0.240753 1.033325
H 3.399180 0.418195 2.126926
C 5.290447 -1.424275 -0.695521
H 3.450938 -2.517600 -0.988336

C 5.943163 -0.420223 0.017148
H 5.761501 1.014784 1.612795
H 5.815830 -1.956241 -1.487417
H 6.976331 -0.165818 -0.209206
Ge 0.685192 -0.906855 -1.161132

IV
E = -3923.37873296
C 3.496023 2.391291 0.720878
C 2.267340 1.768031 0.597648
C 1.138592 2.298077 -0.035818
C 1.295746 3.565683 -0.602739
C 2.513986 4.231275 -0.496113
C 3.606573 3.661611 0.163959
H 4.325336 1.913381 1.238621
H 0.456906 4.042401 -1.108761
H 2.617756 5.226524 -0.925852
H 4.541509 4.211959 0.244976
B -0.065392 1.261030 0.033555
S 1.860812 0.116184 1.051065
O 2.602319 -0.424421 2.195234
O 0.336985 0.426980 1.332676
C 1.825967 -0.829690 -0.341262
C -1.580360 1.685580 0.086343
C -2.355956 1.527813 1.241243
C -2.222947 2.190591 -1.053783
C -3.712036 1.838451 1.255105
H -1.886507 1.138936 2.144839
C -3.572721 2.520136 -1.043710
H -1.655152 2.305718 -1.978852
C -4.327237 2.336735 0.112029
H -4.293555 1.687197 2.163611
H -4.044318 2.907701 -1.945909
H -5.389136 2.576599 0.119037
P 2.621364 -2.341863 -0.255085
C 4.386329 -2.234025 0.136823
H 4.842317 -3.228336 0.199858
H 4.885388 -1.641259 -0.636753
H 4.478424 -1.720699 1.100096
C 2.471153 -3.158945 -1.853811
H 2.926386 -2.525667 -2.621888
H 2.965238 -4.136146 -1.825588
H 1.408686 -3.277616 -2.090668
C 1.912548 -3.460963 0.981443
H 2.023623 -2.973455 1.956819
H 0.845152 -3.570040 0.758793
H 2.406065 -4.439369 0.990207
C -3.145114 -1.487281 -1.004019
C -2.412596 -1.660486 0.164233
C -3.056102 -1.898682 1.374873
C -4.444138 -1.945525 1.414266
C -5.187951 -1.767618 0.252638
C -4.532391 -1.542425 -0.953530
H -2.618319 -1.284241 -1.934087
H -2.466145 -2.032506 2.280050
H -4.946661 -2.120972 2.363679
H -6.274646 -1.799668 0.287977
H -5.105557 -1.391501 -1.866184
O -1.043950 -1.606227 0.099403
H -0.683315 -1.086798 0.843119

Ge 0.328842 -0.286461 -1.519824

V
E = -3374.15084779
C -1.960638 1.716557 1.586501
C -0.031757 0.245037 1.501870
C 0.624837 1.101330 2.388341
C 0.019982 2.258920 2.872484
C -1.271555 2.559730 2.456810
H -2.974037 1.993548 1.299963
H 1.635190 0.851930 2.709901
H 0.545084 2.915461 3.562903
H -1.761592 3.462901 2.819072
B 0.563576 -1.018942 0.837685
C 2.066378 -1.365004 0.734685
C 3.074252 -0.381136 0.705541
C 2.472693 -2.707803 0.622297
C 4.414264 -0.723865 0.582571
H 2.798603 0.671705 0.738165
C 3.813231 -3.058249 0.537620
H 1.710966 -3.487007 0.604334
C 4.787315 -2.063340 0.513017
H 5.171039 0.057581 0.538597
H 4.101908 -4.105797 0.469830
H 5.838914 -2.332720 0.425766
C -1.369079 0.541108 1.097780
C -1.984393 -0.418429 0.167360
P -3.559925 -0.176861 -0.476194
C -3.747474 1.344355 -1.463319
H -3.046348 1.289790 -2.303737
H -4.769837 1.450292 -1.843632
H -3.491889 2.220701 -0.859682
C -4.021897 -1.506435 -1.607245
H -5.026463 -1.316178 -2.001334
H -3.296948 -1.556168 -2.425444
H -3.997054 -2.462806 -1.075957
C -4.918545 -0.120570 0.739002
H -5.883400 0.059609 0.251381
H -4.942694 -1.082848 1.261548
H -4.731240 0.664219 1.478025
O 0.176835 0.274805 -1.837306
C 1.153771 1.191779 -1.663426
C 2.363859 0.981093 -2.324696
C 0.996442 2.311826 -0.845137
C 3.414798 1.869059 -2.143645
H 2.465927 0.098799 -2.951733
C 2.058332 3.191490 -0.668754
H 0.054286 2.477489 -0.324492
C 3.274107 2.975444 -1.309476
H 4.359536 1.687466 -2.653193
H 1.928300 4.052312 -0.015211
H 4.102830 3.665088 -1.164718
H -0.569476 0.407196 -1.227177
Ge -1.013401 -2.055961 -0.204417

Pathway C

IV
E = -3923.35260516
C -1.532472 -3.292681 0.266630

C -1.048454 -1.990512 0.251929
C 0.225923 -1.642272 -0.230107
C 0.980359 -2.705036 -0.748893
C 0.516564 -4.016478 -0.743594
C -0.737249 -4.319687 -0.226030
H -2.532902 -3.475562 0.651578
H 1.957932 -2.503536 -1.182226
H 1.142751 -4.808331 -1.151810
H -1.101327 -5.345083 -0.218379
B 0.694367 -0.129825 -0.226061
S -2.145164 -0.740593 0.902421
O -3.495896 -1.352385 0.993857
O -1.578159 -0.232662 2.152557
C -2.215187 0.509200 -0.307944
C 1.356653 0.523386 1.051741
C 1.403309 -0.135471 2.287521
C 1.950430 1.793471 0.978085
C 2.022511 0.438829 3.390222
H 0.927232 -1.109356 2.384999
C 2.580780 2.371877 2.073439
H 1.934618 2.338973 0.031836
C 2.619532 1.692304 3.286356
H 2.040823 -0.094867 4.339189
H 3.040029 3.354986 1.981888
H 3.109273 2.139851 4.149739
P -3.828382 1.104872 -0.519332
C -4.949517 -0.042432 -1.358261
H -5.960545 0.374090 -1.429974
H -4.555154 -0.243307 -2.359899
H -4.951822 -0.967668 -0.774841
C -3.790001 2.597617 -1.534350
H -3.325463 2.371780 -2.499509
H -4.812563 2.960899 -1.685324
H -3.188207 3.363326 -1.035093
C -4.622880 1.565819 1.039961
H -4.670278 0.666597 1.662016
H -3.995336 2.310981 1.539701
H -5.628204 1.967173 0.869604
C 3.351605 0.045599 -1.246959
C 4.096052 0.910847 -2.034118
C 5.466224 1.009325 -1.818080
C 6.072607 0.257172 -0.819154
C 5.303168 -0.598288 -0.036090
C 3.934902 -0.713996 -0.243462
H 3.609396 1.505567 -2.806675
H 6.056955 1.684494 -2.433459
H 7.143052 0.341110 -0.646623
H 5.769063 -1.181518 0.755127
H 3.318717 -1.358696 0.376525
O 1.986054 -0.070743 -1.459208
H 1.681832 0.534750 -2.150606
Ge -0.685579 1.212302 -1.158029

V
E = -3374.14691193
C -1.382079 -0.187492 2.505199
C 0.830298 -0.300584 1.519568
C 1.342025 0.315557 2.661940
C 0.516989 0.701121 3.718086
C -0.846010 0.446263 3.624301

H -2.454449 -0.374735 2.488905
H 2.414564 0.498279 2.726445
H 0.929756 1.185171 4.600817
H -1.509156 0.735006 4.439310
B 1.637316 -0.702524 0.259524
C 3.114147 -0.321357 -0.029049
C 3.635539 0.947773 0.280644
C 3.978616 -1.238510 -0.650003
C 4.954322 1.278403 -0.004174
H 2.979495 1.692660 0.728657
C 5.309447 -0.927722 -0.902500
H 3.591691 -2.216190 -0.937508
C 5.799207 0.335498 -0.584052
H 5.329944 2.273093 0.231344
H 5.963053 -1.664035 -1.367614
H 6.836212 0.589435 -0.798228
C -0.566462 -0.576788 1.431047
C -1.016251 -1.230629 0.197067
P -2.685698 -1.484603 -0.124484
C -3.531373 -2.642065 1.004663
H -3.430995 -2.297660 2.038424
H -4.594071 -2.739797 0.754448
H -3.039916 -3.617226 0.920798
C -3.700654 0.022362 -0.113566
H -4.756214 -0.206034 -0.300766
H -3.598698 0.540950 0.844141
H -3.315529 0.688310 -0.892999
C -2.949209 -2.194771 -1.764631
H -4.020252 -2.369066 -1.916852
H -2.566102 -1.503265 -2.521086
H -2.394558 -3.133992 -1.851966
H 0.933946 1.106174 -2.020072
C -1.230428 2.238579 -2.201067
C -2.405916 2.977073 -2.107005
C -2.827995 3.475806 -0.879161
C -2.062909 3.222419 0.257382
C -0.883009 2.495102 0.177331
C -0.457618 2.015015 -1.061972
H -0.897697 1.848334 -3.162902
H -2.992647 3.162300 -3.005022
H -3.745422 4.055671 -0.807623
H -2.384588 3.600398 1.226235
H -0.277392 2.297009 1.058767
O 0.721538 1.353479 -1.113883
Ge 0.300048 -1.696977 -1.108601

5.2.6 Phenol activation transition states

Pathway A

I
E = -3923.37045268
C -0.618646 2.899490 1.087704
C 1.251573 1.495710 0.450868
C 2.080385 2.237802 1.294681
C 1.556962 3.282626 2.045540
C 0.206439 3.609731 1.949206
H -1.669860 3.178030 1.016347
H 3.136552 1.989896 1.363304
H 2.210708 3.845816 2.708424

H -0.204222 4.424876 2.541567
N 1.674017 0.436505 -0.347056
C 2.936354 -0.177029 -0.233850
C 3.398532 -0.645970 0.999925
C 3.717703 -0.377009 -1.372783
C 4.625195 -1.288747 1.086810
H 2.777143 -0.512134 1.882715
C 4.937693 -1.037301 -1.282359
H 3.357810 -0.002122 -2.329480
C 5.400287 -1.489619 -0.052345
H 4.971544 -1.652229 2.052586
H 5.534016 -1.189156 -2.180154
H 6.358268 -2.000311 0.019736
C -0.126521 1.836154 0.317788
C -0.855471 1.025348 -0.637361
P -2.530666 1.108282 -0.971578
C -3.588721 0.797943 0.459555
H -3.293123 1.457678 1.281719
H -4.643359 0.962769 0.211808
H -3.430681 -0.239300 0.777525
C -3.071901 2.700094 -1.663314
H -4.153673 2.708332 -1.838277
H -2.809163 3.508208 -0.973128
H -2.540005 2.868241 -2.605571
C -2.972876 -0.139275 -2.198366
H -4.034555 -0.051078 -2.451125
H -2.367883 -0.001029 -3.100818
H -2.781783 -1.130876 -1.772332
Ge 0.294352 -0.227130 -1.379027
H 0.029682 -1.749083 -1.735398
O -0.022285 -2.305879 -0.326032
C -1.085379 -2.351444 0.445273
C -1.087735 -1.686381 1.692441
C -2.259951 -3.041318 0.077431
C -2.175191 -1.771831 2.547805
H -0.196795 -1.123742 1.968971
C -3.353227 -3.102997 0.935359
H -2.271395 -3.560948 -0.880186
C -3.320827 -2.479955 2.180604
H -2.134751 -1.268128 3.513076
H -4.239177 -3.659626 0.630761
H -4.172494 -2.544792 2.854427

II
E = -3953.26932526
C -1.173093 2.235282 2.366952
C -0.847974 1.732418 1.111604
C 0.480027 1.451420 0.746719
C 1.475865 1.734379 1.693425
C 1.151544 2.250593 2.938467
C -0.173535 2.493542 3.292190
H -2.221152 2.430547 2.583250
H 2.511703 1.518938 1.446697
H 1.950503 2.452696 3.649624
H -0.422368 2.892855 4.272565
N 0.806188 0.852841 -0.475807
S -2.144981 1.595736 -0.089237
O -3.426017 1.651181 0.645193
O -1.870676 2.598451 -1.114374
C -1.972127 0.013663 -0.736862

C 2.126091 1.059486 -0.982837
C 2.400443 2.246042 -1.664067
C 3.128585 0.105460 -0.811616
C 3.671009 2.474847 -2.175619
H 1.600378 2.973959 -1.784533
C 4.397263 0.339143 -1.333911
H 2.919265 -0.806552 -0.256748
C 4.671596 1.519979 -2.014214
H 3.879425 3.400486 -2.708774
H 5.175560 -0.409251 -1.198108
H 5.665919 1.698650 -2.418868
P -3.232690 -1.122615 -0.450312
C -3.361376 -1.573368 1.294009
H -4.201510 -2.251778 1.479213
H -2.417574 -2.049066 1.583748
H -3.494837 -0.642114 1.855113
C -2.900406 -2.631854 -1.377429
H -1.931518 -3.036843 -1.060765
H -3.697271 -3.357587 -1.182334
H -2.874674 -2.399010 -2.447493
C -4.856617 -0.529055 -0.971872
H -5.058068 0.404359 -0.437766
H -4.825436 -0.328791 -2.047944
H -5.626181 -1.277430 -0.751562
Ge -0.259705 -0.374233 -1.356429
O 0.057958 -2.515400 -0.445484
H 0.363601 -1.808935 -1.703204
C 1.068205 -2.577147 0.421566
C 2.193181 -3.379262 0.171648
C 1.056945 -1.786274 1.582841
C 3.275191 -3.366278 1.042639
H 2.201356 -3.993362 -0.726794
C 2.144268 -1.776721 2.445352
H 0.189187 -1.155805 1.775814
C 3.264771 -2.560556 2.179392
H 4.143307 -3.987476 0.826376
H 2.118643 -1.137550 3.326619
H 4.119756 -2.546199 2.851769

III
E = -4715.35079920
C -1.945667 -1.540760 -0.751048
C -2.804657 -0.872714 0.133009
C -4.130529 -1.280453 0.250044
C -4.616780 -2.340638 -0.505083
C -3.770229 -3.002519 -1.382737
C -2.445408 -2.600005 -1.504754
H -4.771555 -0.740863 0.942533
H -5.656019 -2.645808 -0.401815
H -4.136852 -3.833519 -1.982031
H -1.767619 -3.101784 -2.190755
S -0.219694 -1.107894 -1.156170
S -2.323724 0.480932 1.239334
O 0.304832 -2.325605 -1.813184
O -0.335732 0.096128 -1.993123
O -1.493819 -0.191394 2.307725
O -3.595653 1.106418 1.615098
C 0.671194 -0.873986 0.261653
C -1.193450 1.476707 0.548205
P -1.606082 2.661656 -0.613850

P 1.715859 -2.165403 0.711214
C -0.156697 3.189504 -1.528721
H -0.369542 4.119705 -2.065499
H 0.676550 3.313849 -0.825371
H 0.099533 2.383734 -2.222625
C -2.855067 2.040262 -1.763081
H -3.728341 1.724484 -1.179607
H -3.150557 2.816102 -2.477778
H -2.420689 1.177928 -2.280334
C -2.352032 4.110083 0.176727
H -1.604604 4.583590 0.822023
H -2.727130 4.833229 -0.556450
H -3.171657 3.740203 0.803950
C 2.453026 -1.751173 2.307309
H 3.098806 -2.574156 2.631788
H 3.052015 -0.840874 2.194687
H 1.667380 -1.580607 3.051113
C 0.855879 -3.742523 0.940923
H 0.407890 -4.019357 -0.018142
H 1.549611 -4.523807 1.270900
H 0.064541 -3.600668 1.684787
C 3.096235 -2.468080 -0.405664
H 3.680057 -3.334692 -0.074876
H 2.682708 -2.639457 -1.403504
H 3.724652 -1.570637 -0.423090
H 1.656755 1.572927 1.750893
O 2.104176 2.143079 0.508961
C 3.145979 1.539171 -0.040624
C 3.114026 1.121916 -1.386947
C 4.320044 1.287272 0.694469
C 4.231417 0.556556 -1.981716
H 2.188114 1.241826 -1.943965
C 5.428284 0.698293 0.092782
H 4.354865 1.604887 1.736079
C 5.401589 0.341278 -1.251930
H 4.183390 0.261028 -3.029113
H 6.329660 0.531513 0.681747
H 6.274641 -0.104369 -1.723919
Ge 0.384627 0.682345 1.313018

IV
E = -3923.34222498
C -0.357842 0.211277 3.102034
C 0.377206 0.444507 1.953312
C 1.276583 -0.447444 1.365794
C 1.409553 -1.689378 1.986815
C 0.689366 -1.966432 3.147270
C -0.176688 -1.027412 3.712460
H -1.035291 0.960292 3.508008
H 2.088640 -2.434847 1.576773
H 0.808217 -2.934125 3.631289
H -0.713832 -1.265691 4.628030
B 1.941422 0.211029 0.059765
S 0.206530 1.785890 0.819075
O -0.225346 3.052343 1.406694
O 1.681086 1.725041 0.300145
C -0.697591 1.236939 -0.497210
C 3.426848 -0.107667 -0.345997
C 4.435717 0.858076 -0.266420
C 3.787732 -1.392516 -0.774340

C 5.752933 0.554011 -0.596328
H 4.178785 1.864711 0.061188
C 5.102519 -1.704814 -1.098848
H 3.019063 -2.161686 -0.868967
C 6.092088 -0.729623 -1.010695
H 6.520676 1.323593 -0.527690
H 5.356737 -2.710956 -1.429222
H 7.122423 -0.969210 -1.268321
P -2.274029 1.892168 -0.679502
C -3.230066 1.916875 0.856054
H -4.229600 2.329576 0.679874
H -3.310535 0.889331 1.225126
H -2.696446 2.536975 1.584008
C -3.208093 0.927716 -1.874984
H -3.468411 -0.039953 -1.432234
H -4.119753 1.469218 -2.150012
H -2.591156 0.758618 -2.763959
C -2.235971 3.610051 -1.249537
H -1.628226 4.168921 -0.528859
H -1.747624 3.647841 -2.228911
H -3.241335 4.041510 -1.312897
C -4.157451 -2.720778 -1.195877
C -4.537101 -2.298976 0.077023
C -3.559077 -1.832139 0.953463
C -2.228254 -1.758712 0.561504
C -1.841031 -2.172204 -0.723220
C -2.828221 -2.662000 -1.595035
H -4.907461 -3.102216 -1.887366
H -5.578317 -2.351219 0.387853
H -3.835170 -1.523953 1.961895
H -1.458055 -1.403291 1.240236
H -2.525447 -2.995084 -2.586012
O -0.573118 -2.078227 -1.116982
H -0.407886 -1.275790 -2.215565
Ge 0.396216 -0.060121 -1.441813

V
E = -1585.86013565
C -1.382848 -0.185828 2.504408
C 0.829910 -0.300291 1.519749
C 1.341360 0.316145 2.662099
C 0.516062 0.702465 3.717731
C -0.847038 0.448258 3.623429
H -2.455332 -0.372278 2.487554
H 2.413940 0.498535 2.726954
H 0.928634 1.186719 4.600442
H -1.510389 0.737827 4.437977
B 1.637148 -0.702503 0.259927
C 3.114132 -0.321739 -0.028398
C 3.635861 0.947239 0.281317
C 3.978347 -1.239108 -0.649406
C 4.954734 1.277526 -0.003513
H 2.980038 1.692320 0.729331
C 5.309260 -0.928676 -0.901923
H 3.591132 -2.216672 -0.936908
C 5.799344 0.334409 -0.583443
H 5.330653 2.272097 0.232023
H 5.962662 -1.665136 -1.367087
H 6.836411 0.588086 -0.797634
C -0.566882 -0.576228 1.430917

C -1.016468 -1.230911 0.197273
P -2.685755 -1.485450 -0.124264
C -3.532356 -2.640223 1.006975
H -3.432898 -2.293501 2.040029
H -4.594848 -2.738296 0.756000
H -3.041074 -3.615695 0.925790
C -3.700328 0.021852 -0.117037
H -4.756161 -0.206485 -0.302800
H -3.597274 0.543031 0.839157
H -3.315378 0.685377 -0.898628
C -2.948756 -2.199401 -1.762837
H -4.019822 -2.373726 -1.914876
H -2.565219 -1.509919 -2.520903
H -2.394425 -3.139040 -1.847676
H 0.935182 1.107916 -2.020743
O 0.721523 1.352666 -1.114168
C -0.457105 2.015190 -1.062377
C -0.883004 2.494539 0.177024
C -1.229039 2.240197 -2.201780
C -2.062799 3.222029 0.256977
H -0.277893 2.295789 1.058647
C -2.404365 2.978943 -2.107834
H -0.895825 1.850691 -3.163750
C -2.827194 3.476506 -0.879783
H -2.384926 3.599290 1.225964
H -2.990481 3.165091 -3.006061
H -3.744570 4.056455 -0.808269
Ge 0.300040 -1.696653 -1.108571

Pathway B

I
E = -3404.11420025
C 2.012563 0.717706 -2.347620
C -0.033283 1.072748 -1.105091
C -0.649179 1.520385 -2.276243
C 0.064885 1.568862 -3.469008
C 1.396903 1.175618 -3.510604
H 3.054662 0.401817 -2.394725
H -1.684942 1.848017 -2.246047
H -0.427147 1.928154 -4.370989
H 1.957608 1.219067 -4.441956
N -0.624381 1.062477 0.152470
C -2.003440 1.306730 0.329404
C -2.427482 2.296677 1.218802
C -2.963197 0.531348 -0.330433
C -3.783014 2.500410 1.453748
H -1.679062 2.905567 1.722847
C -4.314521 0.749580 -0.103939
H -2.633393 -0.258175 -1.002232
C -4.732584 1.732168 0.790392
H -4.096834 3.272536 2.154117
H -5.047080 0.130464 -0.618976
H -5.793378 1.895070 0.970537
C 1.319295 0.648499 -1.138689
P 3.468123 -0.085881 0.516692
C 4.290101 -1.397790 -0.436700
H 4.279118 -1.158893 -1.504164
H 5.326380 -1.529554 -0.105034
H 3.733042 -2.328765 -0.284759

C 4.501526 1.388854 0.303151
H 5.558105 1.178387 0.502328
H 4.384797 1.749083 -0.725189
H 4.141844 2.168756 0.982495
C 3.611591 -0.616378 2.238027
H 4.641785 -0.904948 2.470827
H 3.293720 0.192772 2.903731
H 2.941491 -1.472069 2.386123
C 1.805834 0.130983 0.150739
H 1.189974 -1.062681 0.403264
Ge 0.438004 0.505347 1.621244
O 0.354445 -1.739732 1.037857
C -0.694200 -2.207016 0.346521
C -1.875304 -2.554900 1.014615
C -0.646547 -2.335151 -1.048958
C -2.974843 -3.016331 0.304348
H -1.909256 -2.445932 2.096473
C -1.751963 -2.802276 -1.749825
H 0.263541 -2.054844 -1.577009
C -2.924385 -3.144491 -1.081813
H -3.886450 -3.274844 0.841082
H -1.695487 -2.891224 -2.833706
H -3.788435 -3.507515 -1.634325

II
E = -3953.31439801
C 0.181404 1.797302 2.564939
C 0.160575 0.685881 1.728212
C -1.001902 0.266463 1.060986
C -2.169638 1.010291 1.301057
C -2.157439 2.106700 2.144161
C -0.983123 2.516201 2.777422
H 1.122883 2.065813 3.038964
H -3.082359 0.723837 0.784585
H -3.077264 2.669748 2.291163
H -0.980373 3.385102 3.431334
N -1.002722 -0.795852 0.159726
S 1.637075 -0.258747 1.551967
O 2.751953 0.476401 2.186879
O 1.406271 -1.627555 2.025018
C -2.164594 -1.601161 0.118925
C -2.652177 -2.170463 1.302042
C -2.821938 -1.876174 -1.082326
C -3.774890 -2.983462 1.279123
H -2.123036 -1.971511 2.231730
C -3.935180 -2.710811 -1.102114
H -2.460621 -1.422268 -2.002492
C -4.421268 -3.262547 0.076356
H -4.140677 -3.418152 2.207755
H -4.433395 -2.915481 -2.048057
H -5.297943 -3.906751 0.060714
C 1.852379 -0.255454 -0.168612
Ge 0.315812 -1.200736 -1.167933
O 0.450168 0.936438 -1.809746
C -0.514519 1.861864 -1.633675
C -1.784121 1.665453 -2.181485
C -0.262693 3.009880 -0.876571
C -2.789159 2.597859 -1.957819
H -1.966847 0.774707 -2.778668
C -1.273056 3.937943 -0.664602

H 0.725127 3.151002 -0.441372
C -2.542860 3.737581 -1.197835
H -3.777069 2.430061 -2.383751
H -1.066416 4.822164 -0.064097
H -3.333577 4.464227 -1.024393
H 1.249859 0.761089 -0.928413
P 3.508686 -0.507444 -0.610164
C 3.562719 -0.891642 -2.373079
H 4.596730 -0.904806 -2.733145
H 3.094810 -1.865253 -2.553631
H 2.990296 -0.124350 -2.908361
C 4.278392 -1.853339 0.315294
H 3.693539 -2.766221 0.162943
H 5.320448 -2.008178 0.014955
H 4.222447 -1.584433 1.376616
C 4.519442 0.964657 -0.348749
H 5.573495 0.766817 -0.572954
H 4.143682 1.767392 -0.992049
H 4.390574 1.251149 0.700283

III
E = -4715.38070458
C -2.810672 0.734030 -0.375220
C -2.114640 1.506145 0.558950
C -2.745852 2.607437 1.135624
C -4.046272 2.947428 0.784067
C -4.734437 2.180486 -0.145719
C -4.113261 1.081155 -0.724429
H -2.192706 3.188494 1.868686
H -4.519385 3.811297 1.246566
H -5.754397 2.435386 -0.426470
H -4.624377 0.461683 -1.457015
S -2.106799 -0.649060 -1.310048
S -0.489787 1.145784 1.298200
O -3.291781 -1.356659 -1.815744
O -1.210680 0.050109 -2.296079
O -0.713177 0.003937 2.192545
O -0.125407 2.431655 1.931421
C -1.050598 -1.553038 -0.416784
P 1.491973 2.202235 -0.525793
P -1.623573 -2.700019 0.705419
C 2.655081 1.707099 -1.815654
H 3.168550 2.601318 -2.186323
H 3.389884 1.007971 -1.396813
H 2.123788 1.215911 -2.636654
C 0.253471 3.267232 -1.301333
H -0.372066 3.693626 -0.509525
H 0.720083 4.072307 -1.879119
H -0.368132 2.634313 -1.947369
C 2.452861 3.226515 0.612141
H 3.307600 2.640886 0.964930
H 2.810505 4.121368 0.089786
H 1.811027 3.493759 1.455571
C -0.330233 -3.142572 1.870851
H -0.697520 -3.903560 2.567656
H 0.538839 -3.515327 1.320165
H -0.031870 -2.229122 2.394629
C -3.056285 -2.090976 1.626063
H -3.833538 -1.824585 0.899831
H -3.437703 -2.857251 2.309778

H -2.740596 -1.199670 2.176664
C -2.199914 -4.220031 -0.099982
H -2.652935 -4.921808 0.609868
H -2.932507 -3.913796 -0.855928
H -1.354221 -4.695668 -0.607845
C 0.726725 0.777986 0.133485
H 1.442392 -0.188963 0.765059
O 1.974017 -1.313125 0.818440
C 3.267522 -1.077917 0.594540
C 3.938107 -0.082310 1.323783
C 3.969700 -1.750040 -0.417697
C 5.259252 0.240753 1.033325
H 3.399180 0.418195 2.126926
C 5.290447 -1.424275 -0.695521
H 3.450938 -2.517600 -0.988336
C 5.943163 -0.420223 0.017148
H 5.761501 1.014784 1.612795
H 5.815830 -1.956241 -1.487417
H 6.976331 -0.165818 -0.209206
Ge 0.685192 -0.906855 -1.161132

IV
E = -3923.36338742
C 2.943463 2.461451 1.026938
C 1.769955 1.751620 0.831770
C 0.655558 2.189325 0.105364
C 0.785016 3.441333 -0.508043
C 1.950687 4.182572 -0.343614
C 3.022464 3.711269 0.422758
H 3.750234 2.064525 1.641261
H -0.041039 3.846084 -1.091461
H 2.027724 5.164349 -0.808403
H 3.911415 4.324818 0.551926
B -0.478753 1.070903 0.106956
S 1.412659 0.110817 1.350360
O 2.103086 -0.309537 2.565178
O -0.125667 0.267325 1.422760
C 1.555204 -0.933341 0.002668
C -2.015667 1.390169 0.027157
C -2.883158 1.107570 1.088883
C -2.576206 1.937933 -1.136018
C -4.247135 1.358833 0.994024
H -2.479635 0.664957 1.998164
C -3.938420 2.195060 -1.236313
H -1.933715 2.147968 -1.993663
C -4.782710 1.903289 -0.168723
H -4.899674 1.117411 1.832043
H -4.346007 2.617097 -2.154180
H -5.851784 2.094974 -0.245526
P 3.063406 -1.688580 -0.358627
C 4.331118 -0.546029 -0.956720
H 5.244609 -1.080545 -1.240084
H 3.926689 -0.008928 -1.821940
H 4.562456 0.182842 -0.172380
C 2.746239 -2.881092 -1.671218
H 2.440178 -2.334122 -2.569737
H 3.638955 -3.483357 -1.871176
H 1.911534 -3.520224 -1.362576
C 3.775305 -2.576138 1.045815
H 3.884864 -1.875281 1.879820

H 3.074669 -3.360225 1.351702
H 4.741704 -3.020267 0.783097
C -2.908765 -1.773341 -1.001749
C -1.802425 -2.055126 -0.197068
C -1.992662 -2.402562 1.140796
C -3.279736 -2.475015 1.660670
C -4.383106 -2.198620 0.861342
C -4.188214 -1.842343 -0.470540
H -2.747739 -1.485085 -2.038897
H -1.124483 -2.594725 1.768073
H -3.417739 -2.742796 2.707050
H -5.387936 -2.247754 1.275254
H -5.042000 -1.604305 -1.102157
O -0.557511 -1.989354 -0.722945
H 0.458639 -1.735278 -0.053293
Ge 0.279941 -0.198731 -1.539159

V
E = -3374.14018420
C 2.466731 2.274568 0.687193
C 0.257295 1.778298 -0.196330
C -0.101209 3.113510 0.042324
C 0.794767 4.024715 0.583491
C 2.080756 3.591196 0.902252
H 3.484335 1.986216 0.947269
H -1.100786 3.444733 -0.237782
H 0.508137 5.062037 0.743270
H 2.800248 4.294151 1.321038
B -0.631062 0.718953 -0.876047
C -2.173160 0.739880 -0.993713
C -2.980553 1.355381 -0.020112
C -2.827413 0.043819 -2.026889
C -4.366173 1.280180 -0.075268
H -2.505830 1.864644 0.816919
C -4.211956 -0.013768 -2.102338
H -2.227385 -0.464497 -2.781721
C -4.985015 0.601092 -1.120488
H -4.968247 1.746087 0.703128
H -4.693383 -0.551529 -2.917510
H -6.071494 0.543420 -1.166195
C 1.572139 1.334888 0.144664
C 1.868110 -0.079977 -0.110807
P 3.453167 -0.738867 0.022294
C 4.140207 -0.723562 1.708875
H 3.456084 -1.280279 2.358373
H 5.129986 -1.193643 1.730920
H 4.216639 0.299916 2.086980
C 3.398539 -2.487765 -0.426142
H 4.349542 -2.974223 -0.183844
H 2.576380 -2.959978 0.124325
H 3.184280 -2.580206 -1.495629
C 4.739387 0.011004 -1.023877
H 5.714566 -0.465322 -0.871800
H 4.433355 -0.100034 -2.069820
H 4.814747 1.080165 -0.799078
O 0.155037 -1.873963 0.429251
C -0.986960 -1.645201 1.114023
C -2.168130 -2.280427 0.724323
C -1.004731 -0.761224 2.196752
C -3.347986 -2.033838 1.411948

H -2.142848 -2.952300 -0.130710
C -2.191194 -0.529061 2.883102
H -0.084791 -0.254113 2.483852
C -3.367537 -1.162285 2.498180
H -4.265642 -2.523876 1.091097
H -2.193477 0.162128 3.724516
H -4.295377 -0.972113 3.033222
H 0.986809 -1.050897 0.536476
Ge 0.605506 -0.883408 -1.538827

Pathway C

IV
E = -3923.31478853
C -1.688250 1.689206 2.582772
C -1.128583 1.188928 1.412748
C 0.187885 0.705907 1.329666
C 0.902478 0.704142 2.534612
C 0.362601 1.189424 3.721741
C -0.930897 1.696619 3.747895
H -2.712179 2.054782 2.566075
H 1.916130 0.307201 2.541967
H 0.959366 1.171490 4.632269
H -1.355575 2.085316 4.671419
B 0.877761 0.167161 -0.024259
S -2.203316 1.188040 -0.027260
O -3.573825 1.494428 0.448550
O -1.604743 2.051850 -1.039649
C -2.241526 -0.478442 -0.580034
C 1.693776 1.177429 -0.928614
C 1.585086 2.563202 -0.764493
C 2.540554 0.715808 -1.948140
C 2.288177 3.447326 -1.576413
H 0.922196 2.956295 0.004829
C 3.252291 1.591365 -2.758275
H 2.652424 -0.358132 -2.107139
C 3.128983 2.965775 -2.573895
H 2.178381 4.520732 -1.428611
H 3.905522 1.201760 -3.537919
H 3.683549 3.656634 -3.206916
P -3.782138 -1.254792 -0.516079
C -4.450542 -1.411039 1.156307
H -5.426943 -1.907954 1.142416
H -3.744518 -1.984083 1.766191
H -4.542374 -0.398369 1.560797
C -3.593090 -2.927777 -1.168163
H -2.851290 -3.468316 -0.570778
H -4.552459 -3.454985 -1.132398
H -3.240471 -2.875511 -2.203415
C -5.045843 -0.432693 -1.511294
H -5.129203 0.592227 -1.135321
H -4.711722 -0.408266 -2.553696
H -6.008000 -0.951387 -1.435101
C 2.963131 -1.447380 0.449666
C 3.395862 -2.762530 0.294241
C 4.735907 -3.074796 0.482895
C 5.649199 -2.082823 0.824868
C 5.206875 -0.773062 0.975839
C 3.869385 -0.443806 0.788876
H 2.672783 -3.531078 0.027115

H 5.066543 -4.104553 0.360332
H 6.698691 -2.328572 0.970319
H 5.911480 0.014023 1.237659
H 3.527366 0.582334 0.887342
O 1.635720 -1.180403 0.281495
H 0.854735 -2.048828 -0.442767
Ge -0.526127 -1.064907 -0.962376

V
E = -3374.11136171
C -1.329794 2.081465 -1.776248
C 0.679276 0.965360 -0.967043
C 1.313571 1.328568 -2.157903
C 0.642629 2.025749 -3.159115
C -0.687409 2.391228 -2.968180
H -2.352671 2.427867 -1.626567
H 2.350290 1.029415 -2.313637
H 1.150492 2.275821 -4.089048
H -1.220117 2.942488 -3.741697
B 1.384230 0.138567 0.185485
C 2.945300 -0.013996 0.284350
C 3.586795 -1.234839 0.535172
C 3.766784 1.113982 0.123336
C 4.972366 -1.328218 0.622197
H 2.985986 -2.134057 0.663775
C 5.150401 1.029084 0.205022
H 3.302960 2.082687 -0.065547
C 5.762615 -0.196466 0.456459
H 5.438596 -2.293183 0.817945
H 5.757515 1.924255 0.075735
H 6.847086 -0.266824 0.523087
C -0.661545 1.388350 -0.753588
C -1.211862 1.066086 0.564058
P -2.899900 0.821768 0.784823
C -3.911940 2.333005 0.915309
H -3.801164 2.932026 0.005943
H -4.970873 2.092432 1.065733
H -3.542483 2.922586 1.761213
C -3.654615 -0.141957 -0.553283
H -4.723878 -0.297657 -0.371712
H -3.513531 0.383644 -1.502459
H -3.137990 -1.105718 -0.615832
C -3.246740 -0.079811 2.311545
H -4.328898 -0.149721 2.467608
H -2.807231 -1.078849 2.236893
H -2.780742 0.438694 3.155991
H 0.185597 -1.139519 1.646083
C -1.095619 -2.806206 0.461255
C -2.001583 -3.583027 -0.250132
C -2.044529 -3.511484 -1.639588
C -1.162724 -2.668153 -2.307666
C -0.245689 -1.888807 -1.609059
C -0.218797 -1.964538 -0.221750
H -1.042170 -2.859197 1.547546
H -2.673699 -4.249558 0.286956
H -2.755036 -4.116403 -2.198598
H -1.179476 -2.612472 -3.394220
H 0.444738 -1.235186 -2.130362
O 0.704476 -1.266630 0.521546
Ge 0.100841 0.667746 1.882255

5.2.7 Phenol activated products

Pathway A

I
E = -3404.14795825
C 0.989528 -2.819190 1.059302
C -1.001394 -1.624916 0.380691
C -1.747359 -2.394535 1.269286
C -1.129518 -3.360431 2.061784
C 0.239563 -3.565776 1.964588
H 2.058808 -3.014100 0.978834
H -2.821446 -2.242124 1.338311
H -1.728996 -3.951725 2.750920
H 0.730094 -4.315365 2.582530
N -1.533528 -0.639030 -0.462598
C -2.820567 -0.090650 -0.321485
C -3.324688 0.297834 0.925832
C -3.601578 0.132931 -1.459421
C -4.579393 0.883214 1.025621
H -2.718099 0.142641 1.815516
C -4.849384 0.734793 -1.355219
H -3.216923 -0.176986 -2.429290
C -5.348742 1.107703 -0.112315
H -4.953946 1.180123 2.003742
H -5.440293 0.902620 -2.253922
H -6.329383 1.571599 -0.029937
C 0.402009 -1.845505 0.243888
C 1.076778 -1.018518 -0.749393
P 2.754352 -0.773850 -0.839457
C 3.548650 -0.250937 0.708478
H 3.213349 -0.911464 1.515489
H 4.641277 -0.286258 0.631953
H 3.219262 0.767855 0.941591
C 3.714127 -2.221438 -1.388422
H 4.783910 -1.989805 -1.445690
H 3.562101 -3.051455 -0.691042
H 3.343633 -2.528901 -2.371803
C 3.110736 0.519219 -2.050563
H 4.191559 0.673548 -2.131229
H 2.711922 0.222151 -3.026551
H 2.631927 1.450815 -1.729216
Ge -0.186169 0.173084 -1.444895
H -0.554214 0.457123 -2.898616
O 0.128736 1.905998 -0.927677
C 0.734158 2.189762 0.238905
C 0.450647 1.500563 1.425430
C 1.700778 3.202522 0.260396
C 1.121195 1.823386 2.598641
H -0.297626 0.711201 1.422206
C 2.365356 3.518172 1.439805
H 1.904223 3.739791 -0.664175
C 2.084598 2.828257 2.616208
H 0.887230 1.274885 3.509464
H 3.109626 4.313092 1.438564
H 2.605945 3.076283 3.538203

II
E = -3953.34571228

C 3.697582 1.317779 -0.916446
C 2.498427 1.035225 -0.274460
C 1.303797 1.706143 -0.603520
C 1.381543 2.686028 -1.604696
C 2.582759 2.977300 -2.233668
C 3.748925 2.292288 -1.903218
H 4.583983 0.767338 -0.607648
H 0.475095 3.216973 -1.885257
H 2.602920 3.745672 -3.004562
H 4.688154 2.521818 -2.401019
N 0.086717 1.411179 0.010886
S 2.501229 -0.149356 1.052456
O 3.793374 -0.859506 0.995118
O 2.128519 0.585626 2.262025
C 1.238732 -1.232171 0.644812
C -0.884743 2.433250 0.135658
C -0.584627 3.602768 0.842537
C -2.161021 2.267127 -0.403710
C -1.550242 4.585177 1.002496
H 0.409633 3.715319 1.270080
C -3.132313 3.247423 -0.219875
H -2.380560 1.370895 -0.979653
C -2.830245 4.409444 0.478525
H -1.308591 5.490379 1.556650
H -4.126076 3.101215 -0.639073
H -3.587244 5.178879 0.615876
P 1.689455 -2.725201 -0.054485
C 2.724922 -2.564486 -1.532659
H 3.027070 -3.545332 -1.916509
H 2.166203 -2.014870 -2.297556
H 3.609952 -1.985518 -1.248070
C 0.229917 -3.661813 -0.546817
H -0.306370 -3.109796 -1.322575
H 0.542781 -4.643187 -0.919020
H -0.437600 -3.789598 0.311655
C 2.627133 -3.765898 1.088264
H 3.485464 -3.174641 1.423834
H 1.992936 -3.998488 1.949900
H 2.960850 -4.689802 0.602491
Ge -0.373859 -0.271492 0.663609
O -1.479129 -1.076197 -0.534654
H -1.181994 -0.060294 1.939407
C -2.785519 -1.396928 -0.422640
C -3.280890 -2.395696 -1.268568
C -3.663477 -0.769813 0.468043
C -4.617958 -2.764655 -1.215886
H -2.599852 -2.865087 -1.975086
C -5.000027 -1.150838 0.514424
H -3.306075 0.024042 1.118202
C -5.487933 -2.148680 -0.321009
H -4.983597 -3.542043 -1.884770
H -5.667371 -0.650709 1.214256
H -6.535283 -2.439182 -0.280692

III
E = -2620.28203911
C -1.932726 -1.292285 -0.937995
C -2.686396 -0.644349 0.048243
C -4.050901 -0.914270 0.148720
C -4.671842 -1.798267 -0.723747

C -3.927325 -2.425994 -1.713421
C -2.566499 -2.170650 -1.816422
H -4.615635 -0.399078 0.921520
H -5.738283 -1.992042 -0.627160
H -4.401165 -3.116509 -2.408271
H -1.959250 -2.659026 -2.574357
S -0.157784 -1.062679 -1.283165
S -2.066034 0.505182 1.324931
O 0.203906 -2.308693 -1.988009
O -0.076975 0.208219 -2.026026
O -1.428690 -0.365738 2.333498
O -3.276564 1.261198 1.691649
C 0.709185 -0.969785 0.155266
C -0.900640 1.535182 0.685396
P -1.438032 2.792876 -0.351765
P 1.034470 -2.455447 0.943100
C -0.078203 3.457244 -1.319499
H -0.458369 4.259712 -1.960897
H 0.707409 3.831547 -0.657835
H 0.336011 2.636807 -1.914733
C -2.691607 2.240113 -1.532128
H -3.552753 1.855412 -0.975339
H -3.005075 3.073460 -2.170601
H -2.246931 1.439755 -2.133365
C -2.202710 4.149150 0.570429
H -1.446981 4.605328 1.218334
H -2.634181 4.904850 -0.095744
H -2.979249 3.697489 1.197969
C 1.563273 -2.122874 2.633103
H 1.760277 -3.069085 3.147912
H 2.472123 -1.512788 2.626468
H 0.756437 -1.576452 3.135823
C -0.427889 -3.510777 1.075181
H -0.767971 -3.759636 0.064292
H -0.203722 -4.428349 1.630183
H -1.199766 -2.924530 1.588571
C 2.324023 -3.425919 0.130539
H 2.499051 -4.382254 0.636193
H 1.989410 -3.585649 -0.900088
H 3.241964 -2.827637 0.110213
H 1.394485 0.763854 2.331775
O 2.002423 1.780034 -0.036299
C 3.212301 1.240087 -0.316294
C 3.366296 0.364114 -1.396124
C 4.317931 1.557192 0.476466
C 4.614524 -0.180579 -1.671252
H 2.492293 0.122877 -1.995907
C 5.563135 1.007720 0.190590
H 4.181181 2.242831 1.310412
C 5.717977 0.134363 -0.881191
H 4.725272 -0.859028 -2.515928
H 6.419410 1.265860 0.811910
H 6.693315 -0.293887 -1.103732
Ge 0.820968 0.767751 0.910326

IV
E = -3923.42013075
C -0.311994 0.529992 3.097770
C 0.401306 0.649454 1.916831
C 1.310124 -0.285497 1.419482

C 1.488117 -1.441011 2.179959
C 0.793709 -1.600010 3.377271
C -0.090584 -0.623996 3.843124
H -1.002153 1.304556 3.427294
H 2.179624 -2.212503 1.844914
H 0.947175 -2.500620 3.969115
H -0.607996 -0.767676 4.789418
B 1.910312 0.214178 0.007880
S 0.157923 1.846862 0.641528
O -0.272527 3.163111 1.107958
O 1.614581 1.743112 0.074069
C -0.791160 1.154659 -0.553290
C 3.400252 -0.107130 -0.387668
C 4.378388 0.890825 -0.445174
C 3.795777 -1.422992 -0.663467
C 5.699388 0.588686 -0.761939
H 4.093832 1.921511 -0.237243
C 5.114535 -1.732990 -0.973878
H 3.051185 -2.221039 -0.647019
C 6.073234 -0.724902 -1.024555
H 6.442720 1.383812 -0.802568
H 5.395914 -2.763776 -1.184640
H 7.106452 -0.962728 -1.272102
P -2.365699 1.784798 -0.764932
C -3.305829 1.925018 0.776188
H -4.314102 2.309455 0.586438
H -3.363271 0.932739 1.235651
H -2.768116 2.611828 1.438863
C -3.307163 0.725783 -1.874364
H -3.545080 -0.215813 -1.368357
H -4.232194 1.234581 -2.166479
H -2.707164 0.512515 -2.765467
C -2.362927 3.452520 -1.471380
H -1.749504 4.076027 -0.811410
H -1.893582 3.416569 -2.460090
H -3.375602 3.864224 -1.549629
C -3.912101 -2.975552 -1.101151
C -4.394244 -2.533846 0.129038
C -3.520070 -1.904997 1.012972
C -2.189582 -1.696028 0.668125
C -1.708533 -2.116069 -0.579869
C -2.583719 -2.771749 -1.455613
H -4.579574 -3.484548 -1.794957
H -5.434265 -2.696399 0.403772
H -3.875382 -1.581854 1.991169
H -1.494595 -1.222156 1.357730
H -2.196153 -3.108937 -2.414586
O -0.437284 -1.911749 -0.933748
H 0.164759 -0.237587 -2.861004
Ge 0.314517 -0.275676 -1.321731

V
E = -1585.97664167
C -0.029099 3.295888 -0.584974
C 1.630159 1.548518 -0.201454
C 2.618245 2.468298 -0.608253
C 2.316929 3.757883 -1.000229
C 0.973759 4.152561 -0.995881
H -1.053867 3.661757 -0.605678
H 3.658782 2.147863 -0.589509

H 3.097850 4.451384 -1.302991
H 0.707485 5.159675 -1.316404
B 1.948177 0.123675 0.236168
C 3.276970 -0.659915 0.130888
C 4.173920 -0.466635 -0.935397
C 3.621464 -1.624501 1.093786
C 5.359154 -1.184960 -1.024949
H 3.920312 0.240536 -1.722965
C 4.819127 -2.323897 1.027000
H 2.932511 -1.822655 1.914783
C 5.690981 -2.106284 -0.036157
H 6.028467 -1.027394 -1.869000
H 5.067788 -3.053883 1.795365
H 6.623182 -2.665070 -0.100605
C 0.246505 1.974858 -0.149621
C -0.752901 1.060818 0.346488
P -2.365994 1.507977 0.664878
C -2.547207 2.917532 1.811436
H -1.962422 3.769354 1.451002
H -3.596799 3.213864 1.919746
H -2.145672 2.616283 2.784819
C -3.382524 1.967476 -0.776950
H -4.378368 2.301007 -0.463151
H -2.894977 2.763437 -1.347697
H -3.474585 1.083603 -1.415660
C -3.283891 0.166716 1.450108
H -4.268334 0.536889 1.755893
H -3.410652 -0.663509 0.748285
H -2.735903 -0.186543 2.329271
H -0.351018 -1.159570 2.192880
O -0.353502 -1.945128 -0.332553
C -1.636457 -2.186048 -0.627508
C -2.269346 -1.496081 -1.670339
C -2.371203 -3.127781 0.104418
C -3.608747 -1.733948 -1.957322
H -1.684400 -0.773347 -2.235529
C -3.710587 -3.356227 -0.188136
H -1.868836 -3.666429 0.905217
C -4.341388 -2.656568 -1.213802
H -4.086849 -1.194338 -2.774404
H -4.268623 -4.089462 0.392291
H -5.389596 -2.838669 -1.441146
Ge 0.104637 -0.587750 0.829459

Pathway B

I
E = -3404.15650094
C 2.070018 0.740203 1.695814
C -0.248158 0.226813 1.144998
C -0.633237 0.872130 2.333339
C 0.316555 1.483824 3.142500
C 1.669100 1.458145 2.819091
H 3.126805 0.678676 1.434102
H -1.681580 0.916881 2.611175
H -0.015302 2.002703 4.040796
H 2.400322 1.974336 3.436379
N -1.086237 -0.330899 0.205583
C -2.482684 -0.233709 0.247919
C -3.243227 -1.354048 -0.118978

C -3.171380 0.947450 0.565568
C -4.629743 -1.299659 -0.165516
H -2.720298 -2.274261 -0.374143
C -4.558941 0.992725 0.531048
H -2.609968 1.840205 0.826695
C -5.299703 -0.127226 0.166778
H -5.191182 -2.185782 -0.457156
H -5.066500 1.923561 0.779244
H -6.386423 -0.084340 0.137514
C 1.139931 0.102072 0.883573
P 1.864768 -2.398219 0.268799
C 3.235416 -2.490433 1.446319
H 2.998166 -1.852323 2.304612
H 3.391975 -3.521578 1.782551
H 4.149113 -2.116472 0.971540
C 0.429807 -3.100866 1.101924
H 0.669184 -4.090094 1.505864
H 0.136098 -2.422470 1.911236
H -0.401061 -3.169748 0.391348
C 2.285421 -3.459742 -1.127991
H 2.489167 -4.482408 -0.793751
H 1.443692 -3.453852 -1.830155
H 3.169921 -3.057956 -1.634341
C 1.498827 -0.763406 -0.288033
H 2.342789 -0.391482 -0.883940
Ge -0.249552 -0.885433 -1.517597
O 0.041090 0.797988 -2.288827
C 0.649276 1.828709 -1.691136
C -0.051965 2.682804 -0.830315
C 2.013198 2.069847 -1.907139
C 0.605592 3.702870 -0.157876
H -1.114844 2.503230 -0.689957
C 2.663391 3.099578 -1.236765
H 2.544535 1.429900 -2.610088
C 1.969140 3.914064 -0.347685
H 0.046982 4.339846 0.526397
H 3.725443 3.267785 -1.412464
H 2.482242 4.712302 0.184593

II
E = -3953.35564742
C 1.799828 3.135741 1.054022
C 1.193063 1.961611 0.619665
C 0.032267 1.953699 -0.180251
C -0.487814 3.220488 -0.515752
C 0.104835 4.389451 -0.071684
C 1.255928 4.363243 0.715422
H 2.696455 3.051804 1.664191
H -1.372076 3.269060 -1.145539
H -0.334179 5.343568 -0.358843
H 1.718199 5.285503 1.058821
N -0.544663 0.787646 -0.661902
S 1.893364 0.437179 1.141581
O 3.235527 0.695367 1.703786
O 0.923188 -0.177059 2.092951
C 1.987506 -0.495790 -0.271905
C -1.925626 0.826876 -0.982441
C -2.857483 1.223305 -0.015143
C -2.391707 0.418245 -2.233989
C -4.214974 1.193344 -0.290542

H -2.497527 1.531375 0.964003
C -3.757299 0.372696 -2.499420
H -1.675213 0.135008 -3.001896
C -4.675297 0.758754 -1.531854
H -4.922919 1.488453 0.481648
H -4.100529 0.044122 -3.478907
H -5.742541 0.724320 -1.740866
P 3.494986 -1.250490 -0.550980
C 4.841085 -0.095510 -0.909389
H 5.794451 -0.619042 -1.042216
H 4.589312 0.467993 -1.813665
H 4.899556 0.593363 -0.060803
C 3.344524 -2.338239 -1.986054
H 3.032860 -1.754272 -2.858356
H 4.308359 -2.816371 -2.190080
H 2.588006 -3.104907 -1.788606
C 4.079696 -2.275453 0.822782
H 4.143269 -1.623487 1.700776
H 3.339667 -3.059722 1.013769
H 5.057869 -2.720756 0.608819
C -1.780432 -1.818724 1.081242
C -2.185479 -1.244060 2.283992
C -2.719978 -2.332306 0.190168
C -3.540074 -1.200216 2.594797
H -1.435296 -0.825933 2.952185
C -4.069109 -2.270763 0.509446
H -2.379684 -2.757889 -0.751604
C -4.487022 -1.711056 1.714054
H -3.855366 -0.753742 3.536345
H -4.802099 -2.660063 -0.194576
H -5.545775 -1.667652 1.960178
Ge 0.333087 -0.852879 -1.224381
O -0.462508 -1.880461 0.744043
H 0.082184 -1.344433 1.388878

III
E = -4715.41592234
C 2.659147 -0.598664 0.022859
C 1.895017 -1.260646 0.985444
C 2.515242 -2.119542 1.894713
C 3.885009 -2.335906 1.840421
C 4.644856 -1.697561 0.868550
C 4.030639 -0.840662 -0.034757
H 1.898424 -2.620263 2.636607
H 4.352794 -3.006335 2.558252
H 5.718506 -1.865535 0.809836
H 4.598897 -0.322251 -0.803179
S 2.007352 0.502500 -1.277430
S 0.138090 -1.040326 1.365896
O 3.224756 1.199826 -1.731308
O 1.358416 -0.459542 -2.208335
O 0.006268 0.209235 2.114581
O -0.234087 -2.290952 2.057513
C 0.841457 1.498900 -0.653229
P -0.852405 -2.488349 -1.018173
P 1.364088 2.791782 0.333690
C -1.342365 -2.162044 -2.716376
H -1.417644 -3.109334 -3.261950
H -2.303224 -1.637886 -2.728021
H -0.577866 -1.512774 -3.158375

C 0.724969 -3.350012 -1.066610
H 1.006308 -3.645436 -0.051456
H 0.607067 -4.239044 -1.696061
H 1.470572 -2.673793 -1.497637
C -2.056627 -3.614399 -0.282426
H -3.045474 -3.142169 -0.306478
H -2.089428 -4.570361 -0.816530
H -1.758985 -3.761251 0.761895
C 0.016527 3.495914 1.290529
H 0.404754 4.322231 1.896111
H -0.776058 3.840604 0.621462
H -0.400490 2.698814 1.912611
C 2.630955 2.302099 1.535327
H 3.500091 1.917101 0.990780
H 2.930817 3.160134 2.147405
H 2.207708 1.513141 2.166499
C 2.126164 4.134339 -0.618789
H 2.547300 4.913690 0.026817
H 2.909293 3.674674 -1.232381
H 1.369984 4.566583 -1.282733
C -0.833963 -0.964606 -0.105535
H -1.836889 -0.879859 0.347785
O -1.943833 1.707793 0.147891
C -3.143012 1.192894 0.412166
C -3.380220 0.585244 1.657844
C -4.180780 1.195112 -0.535722
C -4.614915 0.014368 1.942640
H -2.564951 0.566488 2.378412
C -5.409804 0.616694 -0.240526
H -4.003002 1.669300 -1.499270
C -5.637890 0.021666 0.997417
H -4.777583 -0.446237 2.916357
H -6.202739 0.636995 -0.987425
H -6.603415 -0.425529 1.225084
Ge -0.958802 0.900688 -1.294656

IV
E = -3923.38751983
C 3.179061 2.057410 1.109319
C 1.951655 1.433816 0.913488
C 0.932397 1.852714 0.040710
C 1.256627 2.967496 -0.754373
C 2.481412 3.601464 -0.608083
C 3.438748 3.173270 0.328563
H 3.884698 1.697609 1.856799
H 0.528185 3.354425 -1.464745
H 2.700875 4.477536 -1.216599
H 4.370054 3.722198 0.446998
B -0.339953 0.924137 0.135206
S 1.412418 -0.080278 1.562540
O 1.999672 -0.526431 2.816110
O -0.100445 0.181921 1.528616
C 1.586933 -1.230700 0.217098
C -1.805292 1.475952 0.021091
C -2.723389 1.359499 1.072001
C -2.253628 2.072595 -1.166166
C -4.029136 1.816974 0.943099
H -2.411550 0.878385 1.997470
C -3.556265 2.538908 -1.298210
H -1.577438 2.145628 -2.019972

C -4.452723 2.411199 -0.241477
H -4.726130 1.697462 1.771180
H -3.878248 2.993376 -2.234486
H -5.477525 2.764051 -0.344662
P 3.183608 -1.437449 -0.480196
C 3.552192 -0.154272 -1.687350
H 4.542309 -0.328482 -2.123053
H 2.778037 -0.203137 -2.463023
H 3.521633 0.834833 -1.217393
C 3.170424 -3.018364 -1.339484
H 2.357126 -2.991860 -2.073969
H 4.130826 -3.182490 -1.839804
H 2.982404 -3.823619 -0.621821
C 4.512751 -1.466025 0.745420
H 4.543386 -0.499256 1.258117
H 4.291679 -2.238718 1.489301
H 5.481679 -1.660202 0.272239
C -3.153888 -1.396929 -1.048212
C -2.259156 -1.896975 -0.090295
C -2.762516 -2.266860 1.163788
C -4.114859 -2.131093 1.448568
C -4.994846 -1.621889 0.497995
C -4.501418 -1.253839 -0.750591
H -2.771266 -1.099699 -2.024706
H -2.063312 -2.644651 1.906807
H -4.484940 -2.419089 2.431956
H -6.051646 -1.506039 0.729354
H -5.172172 -0.840653 -1.502317
O -0.954340 -2.031227 -0.334047
H 1.154292 -2.178635 0.560843
Ge 0.075138 -0.739490 -1.288597

V
E = -3374.18787417
C 2.466731 2.274568 0.687193
C 0.257295 1.778298 -0.196330
C -0.101209 3.113510 0.042324
C 0.794767 4.024715 0.583491
C 2.080756 3.591196 0.902252
H 3.484335 1.986216 0.947269
H -1.100786 3.444733 -0.237782
H 0.508137 5.062037 0.743270
H 2.800248 4.294151 1.321038
B -0.631062 0.718953 -0.876047
C -2.173160 0.739880 -0.993713
C -2.980553 1.355381 -0.020112
C -2.827413 0.043819 -2.026889
C -4.366173 1.280180 -0.075268
H -2.505830 1.864644 0.816919
C -4.211956 -0.013768 -2.102338
H -2.227385 -0.464497 -2.781721
C -4.985015 0.601092 -1.120488
H -4.968247 1.746087 0.703128
H -4.693383 -0.551529 -2.917510
H -6.071494 0.543420 -1.166195
C 1.572139 1.334888 0.144664
C 1.868110 -0.079977 -0.110807
P 3.453167 -0.738867 0.022294
C 4.140207 -0.723562 1.708875
H 3.456084 -1.280279 2.358373

H 5.129986 -1.193643 1.730920
H 4.216639 0.299916 2.086980
C 3.398539 -2.487765 -0.426142
H 4.349542 -2.974223 -0.183844
H 2.576380 -2.959978 0.124325
H 3.184280 -2.580206 -1.495629
C 4.739387 0.011004 -1.023877
H 5.714566 -0.465322 -0.871800
H 4.433355 -0.100034 -2.069820
H 4.814747 1.080165 -0.799078
O 0.155037 -1.873963 0.429251
C -0.986960 -1.645201 1.114023
C -2.168130 -2.280427 0.724323
C -1.004731 -0.761224 2.196752
C -3.347986 -2.033838 1.411948
H -2.142848 -2.952300 -0.130710
C -2.191194 -0.529061 2.883102
H -0.084791 -0.254113 2.483852
C -3.367537 -1.162285 2.498180
H -4.265642 -2.523876 1.091097
H -2.193477 0.162128 3.724516
H -4.295377 -0.972113 3.033222
H 0.986809 -1.050897 0.536476
Ge 0.605506 -0.883408 -1.538827

Pathway C

IV
E = -3923.39251415
C -1.092792 3.431077 -0.623373
C -0.757095 2.098307 -0.385945
C 0.538747 1.690701 -0.008274
C 1.470517 2.733109 0.124751
C 1.162950 4.061809 -0.136530
C -0.125729 4.418195 -0.512412
H -2.119093 3.675470 -0.885214
H 2.475412 2.484487 0.457524
H 1.933527 4.823402 -0.030086
H -0.384228 5.457203 -0.706003
B 1.088252 0.226595 0.395371
S -2.142921 1.004889 -0.707551
O -3.389040 1.786444 -0.540008
O -1.976559 0.395737 -2.031891
C -2.164927 -0.240078 0.515508
C 1.128438 -0.955148 -0.703969
C 0.755961 -0.787848 -2.044097
C 1.603585 -2.221352 -0.312437
C 0.852617 -1.836638 -2.950502
H 0.386371 0.175862 -2.383129
C 1.701047 -3.273020 -1.214810
H 1.930015 -2.367483 0.719056
C 1.320316 -3.081900 -2.539628
H 0.562128 -1.679425 -3.987871
H 2.081473 -4.239268 -0.887743
H 1.394382 -3.900937 -3.253270
P -3.743405 -0.950649 0.619466
C -4.914733 0.061735 1.550649
H -5.918155 -0.378212 1.535026
H -4.560435 0.154392 2.582570
H -4.914267 1.049495 1.078395

C -3.662670 -2.552979 1.444411
H -3.294890 -2.430797 2.467712
H -4.666063 -2.992018 1.469554
H -2.981742 -3.212714 0.897446
C -4.465140 -1.227513 -1.013314
H -4.576623 -0.253030 -1.500001
H -3.768890 -1.828084 -1.606938
H -5.436168 -1.726952 -0.923041
C 3.499168 0.077070 0.904760
C 4.416806 -0.347856 1.870906
C 5.753192 -0.522517 1.540192
C 6.199987 -0.280342 0.243795
C 5.289115 0.145971 -0.716342
C 3.948740 0.328279 -0.396460
H 4.052260 -0.533057 2.878902
H 6.454045 -0.854214 2.304830
H 7.247480 -0.420908 -0.013960
H 5.622239 0.342698 -1.734129
H 3.244182 0.664883 -1.153583
O 2.222762 0.251736 1.285930
H -0.862521 -2.019458 2.143381
Ge -0.578431 -0.710266 1.345282

V
E = -3374.20212951
C -1.128067 -1.870114 1.979435
C 0.747020 -0.544593 1.220665
C 1.334889 -0.735505 2.477875
C 0.694564 -1.437470 3.491851
C -0.551275 -2.000143 3.234493
H -2.071483 -2.379745 1.787058
H 2.312279 -0.292721 2.671787
H 1.161905 -1.548580 4.468197
H -1.066439 -2.568740 4.007405
B 1.482142 0.417048 0.220209
C 3.009752 0.311942 -0.089973
C 3.706572 1.404455 -0.623736
C 3.728175 -0.864907 0.162014
C 5.067931 1.329758 -0.890187
H 3.160281 2.323433 -0.830886
C 5.088873 -0.948605 -0.103879
H 3.201110 -1.731137 0.560797
C 5.761598 0.151361 -0.629300
H 5.592617 2.189873 -1.303183
H 5.628258 -1.873494 0.093471
H 6.828392 0.088767 -0.838957
C -0.506804 -1.149070 0.943590
C -1.056049 -1.072387 -0.420345
P -2.772850 -0.975150 -0.622946
C -3.639075 -2.579985 -0.668924
H -3.468799 -3.126858 0.263637
H -4.716445 -2.445621 -0.821340
H -3.220378 -3.166817 -1.493728
C -3.587628 -0.017902 0.682427
H -4.672613 -0.008690 0.531239
H -3.352935 -0.436343 1.664633
H -3.198127 1.004318 0.640571
C -3.303599 -0.173054 -2.153745
H -4.395723 -0.081436 -2.139435
H -2.847147 0.818891 -2.209579

H -2.984843 -0.760580 -3.017550
H -0.868496 -1.104354 -3.048620
C -1.116311 2.621838 -0.934827
C -2.268256 3.330939 -0.616020
C -2.616584 3.548087 0.714947
C -1.804581 3.036227 1.723156
C -0.652100 2.321662 1.415102
C -0.296270 2.125741 0.079569
H -0.812868 2.462161 -1.967319
H -2.891937 3.728592 -1.415127
H -3.511155 4.114806 0.963980
H -2.064238 3.199893 2.767483
H -0.010225 1.934841 2.201930
O 0.874508 1.558280 -0.274444
Ge 0.180573 -1.281074 -1.845715

6 Results of the Calculations of all Stannylenes

6.1 Energies

6.1.1 Energies of the Stannylenes

Table S6.1. Energies of the stannylenes (singlet states).

Stannylene	E(SCF)	Corr(H)	Corr(G)	ΔG [hartree]	ΔG [kJ/mol]
$^{Tos}Y_2Sn$	-3796.60560565	0.885672	0.730145	–	–
^{Tos}YSnCl	-2360.61257775	0.446229	0.349777	–	–
^{Tos}YSnHMDS	-2774.39859831	0.690305	0.564848	–	–
$^{Tos}YSnC_6F_5$	-2628.93794305	0.503683	0.390764	–	–
^{Tos}YSnPy	-2148.07780408	0.527350	0.423199	–	–
^{Tos}YSnPyr	-2164.13503634	0.515254	0.411564	–	–
^{Tos}YSnB2	-2307.08730068	0.545554	0.439022	–	–
^{Tos}YSnB1	-2576.27485486	0.692686	0.571462	–	–
$^{Ph}Y_2Sn$	-2619.38479815	0.798397	0.665313	–	–
^{Ph}YSnCl	-1772.01021413	0.403194	0.315768	–	–
^{Ph}YSnHMDS	-2185.79301904	0.646948	0.530772	–	–
$^{Ph}YSnC_6F_5$	-2040.33947504	0.460469	0.356804	–	–
^{Ph}YSnPy	-1559.47095129	0.484077	0.389566	–	–
^{Ph}YSnPyr	-1575.53461591	0.472116	0.378692	–	–
^{Ph}YSnB2	-1718.48752229	0.502015	0.404004	–	–
^{Ph}YSnB1	-1987.67528184	0.649799	0.538052	–	–
$^{F}Y_2Sn$	-3613.20987155	0.726604	0.578267	–	–
^{F}YSnCl	-2268.91939508	0.366893	0.270335	–	–
^{F}YSnHMDS	-2682.70057505	0.610658	0.487429	–	–
$^{F}YSnC_6F_5$	-2537.24629212	0.424019	0.309924	–	–
^{F}YSnPy	-2056.38309255	0.447799	0.343656	–	–
^{F}YSnPyr	-2072.44424700	0.435840	0.332332	–	–
^{F}YSnB2	-2215.39676361	0.466091	0.360027	–	–
^{F}YSnB1	-2484.58534594	0.613571	0.492506	–	–
I	-1021.50458148	0.319035	0.249992	–	–
II	-1570.70479972	0.332775	0.258130	–	–
III	-2332.77588920	0.372956	0.289027	–	–
IV	-1540.75684820	0.331773	0.256570	–	–
V	-991.51969892	0.317750	0.247187	–	–

Table S6.2. Energies of the stannylenes in triplet state, energy of the singlet-triplet gap.

Stannylene	E(SCF)	Corr(H)	Corr(G)	ΔG [hartree]	ΔG [kJ/mol]
$^{Tos}Y_2Sn$	-3796.52462504	0.885826	0.729822	0.080658	211.766555
^{Tos}YSnCl	-2360.52303224	0.445429	0.344105	0.083874	220.209901
^{Tos}YSnHMDS	-2774.31845384	0.690306	0.563925	0.079221	207.995969
$^{Tos}YSnC_6F_5$	-2628.85898412	0.503192	0.385474	0.073669	193.417776
^{Tos}YSnPy	-2148.01079010	0.527169	0.422172	0.065987	173.248816
^{Tos}YSnPyr	-2164.06987180	0.514953	0.407930	0.061531	161.548433
^{Tos}YSnB2	-2307.03326626	0.545600	0.434719	0.049731	130.569843
^{Tos}YSnB1	-2576.21982219	0.692854	0.572893	0.056464	148.245366
$^{Ph}Y_2Sn$	-2619.33185873	0.797918	0.668170	0.055796	146.493501
^{Ph}YSnCl	-1771.95276395	0.402525	0.314282	0.055964	146.933955
^{Ph}YSnHMDS	-2185.73168332	0.645552	0.528427	0.058991	154.880135
$^{Ph}YSnC_6F_5$	-2040.27492747	0.459674	0.355638	0.063382	166.408312
^{Ph}YSnPy	-1559.42247741	0.483747	0.387680	0.046588	122.316479
^{Ph}YSnPyr	-1575.48047566	0.471051	0.373748	0.049196	129.164754
^{Ph}YSnB2	-1718.44247543	0.501942	0.400454	0.041497	108.950006
^{Ph}YSnB1	-1987.63310702	0.649230	0.539259	0.043382	113.898968
$^{F}Y_2Sn$	-3613.13916598	0.724319	0.572791	0.065230	171.260236
^{F}YSnCl	-2268.85323125	0.366241	0.268603	0.064432	169.165770
^{F}YSnHMDS	-2682.63265904	0.609343	0.484403	0.064890	170.368721
$^{F}YSnC_6F_5$	-2537.18658094	0.422925	0.308826	0.058613	153.888904
^{F}YSnPy	-2056.31320271	0.447358	0.342624	0.068858	180.786259
^{F}YSnPyr	-2072.38256343	0.435413	0.328827	0.058179	152.747836
^{F}YSnB2	-2215.34854599	0.465840	0.354662	0.042853	112.509554
^{F}YSnB1	-2484.52838376	0.613292	0.489525	0.053981	141.727588
I	-1021.43970102	0.318490	0.247070	0.061958	162.671937
II	-1570.62964588	0.332131	0.253909	0.070933	186.234171
III	-2332.69444789	0.373223	0.290310	0.082724	217.192676
IV	-1540.73418205	0.331875	0.252849	0.018945	49.740491
V	-991.49553744	0.317101	0.244654	0.021628	56.785564

6.1.2 Energies of the H_2 activation

Pathway A

Table S6.3. Energies of the transition states of the H_2 activation following pathway **A**

Stannylene	E(SCF)	Corr(H)	Corr(G)	ΔG [hartree]	ΔG [kJ/mol]	1M ΔG [kJ/mol]
$^{Tos}Y_2Sn$	-3797.68220505	0.897461	0.744010	0.113342	297.580124	289.654
^{Tos}YSnCl	-2361.68717424	0.457925	0.359170	0.110873	291.097528	283.172
^{Tos}YSnHMDS	-2775.47081470	0.702478	0.580060	0.119072	312.624265	304.698
$^{Tos}YSnC_6F_5$	-2630.02508219	0.516107	0.405024	0.103198	270.945109	263.019
^{Tos}YSnPy	-2149.17493559	0.539547	0.435833	0.091579	240.441078	232.515
^{Tos}YSnPyr	-2165.23134254	0.527142	0.422786	0.090992	238.900724	230.975
^{Tos}YSnB2	n.o.	n.o.	n.o.	–	–	–
^{Tos}YSnB1	-2577.38249734	0.704886	0.585832	0.082804	217.402395	209.476
$^{Ph}Y_2Sn$	-2620.48405265	0.810424	0.678825	0.090334	237.172357	229.246
^{Ph}YSnCl	-1773.09232286	0.414498	0.327307	0.105507	277.008465	269.083
^{Ph}YSnHMDS	-2186.88399426	0.658785	0.543768	0.098097	257.554849	249.629
$^{Ph}YSnC_6F_5$	-2041.43162396	0.472659	0.370274	0.097398	255.717786	247.792
^{Ph}YSnPy	-1560.57715178	0.495887	0.400731	0.081041	212.773612	204.848
^{Ph}YSnPyr	-1576.63642433	0.483929	0.389539	0.085115	223.470083	215.544
^{Ph}YSnB2	-1719.59998698	0.514065	0.412882	0.072490	190.322436	182.397
^{Ph}YSnB1	-1988.78927655	0.661379	0.548735	0.072765	191.044396	183.118
$^{F}Y_2Sn$	-3614.29083094	0.738189	0.588744	0.105594	277.237776	269.312
^{F}YSnCl	-2269.99998836	0.378648	0.283176	0.108324	284.405680	276.480
^{F}YSnHMDS	-2683.78422834	0.623336	0.499101	0.104095	273.302414	265.377
$^{F}YSnC_6F_5$	-2538.32491531	0.435961	0.322077	0.109606	287.771807	279.846
^{F}YSnPy	-2057.46965553	0.459537	0.354925	0.100783	264.604946	256.679
^{F}YSnPyr	-2073.52863446	0.447589	0.343706	0.103063	270.592452	262.667
^{F}YSnB2	-2216.50349980	0.477847	0.369020	0.078333	205.664545	197.739
^{F}YSnB1	-2485.69725006	0.625560	0.504776	0.076443	200.699909	192.774
I	-1022.56486991	0.329747	0.259556	0.125063	329.112355	321.186
II	-1571.77998350	0.344096	0.269090	0.111595	293.669812	285.744
III	-2333.86304916	0.385261	0.302916	0.102568	269.916441	261.991
IV	n.o.	n.o.	n.o.	–	–	–
V	n.o.	n.o.	n.o.	–	–	–

Table S6.4. Energies of the H_2 activated products following pathway **A**

Stannylene	E(SCF)	Corr(H)	Corr(G)	ΔG [hartree]	ΔG [kJ/mol]	1M ΔG [kJ/mol]
**$^{Tos}Y_2Sn$**	-3797.77917506	0.902175	0.749892	0.022254	58.428553	50.503
**$^{Tos}YSnCl$**	-2361.77899927	0.461862	0.364658	0.024536	64.419656	56.494
**$^{Tos}YSnHMDS$**	-2775.57458786	0.706788	0.582090	0.017329	45.497598	37.572
**$^{Tos}YSnC_6F_5$**	-2630.11579236	0.519967	0.406166	0.013629	35.783878	27.858
**$^{Tos}YSnPy$**	-2149.25791222	0.542983	0.438035	0.010805	28.367287	20.441
**$^{Tos}YSnPyr$**	-2165.31623436	0.530797	0.426213	0.009528	25.014839	17.089
**$^{Tos}YSnB2$**	-2308.27645164	0.560815	0.451134	-0.000962	-2.526499	-10.452
**$^{Tos}YSnB1$**	-2577.45693301	0.708249	0.583635	0.006172	16.203320	8.277
**$^{Ph}Y_2Sn$**	-2620.57497065	0.813836	0.682108	0.002699	7.086665	-0.839
**$^{Ph}YSnCl$**	-1773.18870127	0.418383	0.332384	0.014206	37.296613	29.371
**$^{Ph}YSnHMDS$**	-2186.97193692	0.662394	0.545340	0.011727	30.788681	22.863
**$^{Ph}YSnC_6F_5$**	-2041.51632429	0.475468	0.370713	0.013136	34.489664	26.564
**$^{Ph}YSnPy$**	-1560.65390547	0.499014	0.402109	0.005665	14.874738	6.949
**$^{Ph}YSnPyr$**	-1576.72065164	0.487386	0.393661	0.005010	13.153591	5.228
**$^{Ph}YSnB2$**	-1719.67372922	0.517379	0.417846	0.003712	9.745167	1.819
**$^{Ph}YSnB1$**	-1988.86624564	0.664662	0.551147	-0.001792	-4.705244	-12.631
**$^{F}Y_2Sn$**	-3614.39676385	0.742686	0.596278	0.007195	18.891438	10.966
**$^{F}YSnCl$**	-2270.09241554	0.382126	0.287470	0.020191	53.012016	45.086
**$^{F}YSnHMDS$**	-2683.88011039	0.626521	0.500716	0.009828	25.804274	17.878
**$^{F}YSnC_6F_5$**	-2538.42233005	0.439963	0.327919	0.018034	47.347578	39.422
**$^{F}YSnPy$**	-2057.56659845	0.462980	0.356644	0.005559	14.594544	6.669
**$^{F}YSnPyr$**	-2073.62521702	0.449947	0.347901	0.010676	28.028913	20.103
**$^{F}YSnB2$**	-2216.58280477	0.480863	0.372091	0.002100	5.512257	-2.414
**$^{F}YSnB1$**	-2485.77711052	0.628970	0.508353	0.000159	0.417685	-7.508
**I**	-1022.65604664	0.333336	0.262203	0.036738	96.677549	88.752
**II**	-1571.87088588	0.348468	0.272594	0.024398	64.205365	56.279
**III**	-2333.95836046	0.388943	0.305636	0.010191	26.817983	18.892
**IV**	-1541.94749500	0.346416	0.268209	-0.002924	-7.695633	-15.622
**V**	-992.71561093	0.332317	0.260538	-0.006484	-17.024599	-24.950

Pathway B

Table S6.5. Energies of the transition states of the H_2 activation following pathway **B**

Stannylene	E(SCF)	Corr(H)	Corr(G)	ΔG [hartree]	ΔG [kJ/mol]	1M ΔG [kJ/mol]
**$^{Tos}Y_2Sn$**	-3797.71955061	0.897874	0.743979	0.075966	199.447965	191.522
**$^{Tos}YSnCl$**	n.o.	n.o.	n.o.	–	–	–
**$^{Tos}YSnHMDS$**	-2361.74633085	0.459510	0.362395	0.054942	144.249086	136.323
**$^{Tos}YSnB1$**	n.o.	n.o.	n.o.	–	–	–
**$^{Ph}Y_2Sn$**	-2620.52892475	0.812317	0.680373	0.047010	123.424933	115.499
**$^{Ph}YSnCl$**	n.o.	n.o.	n.o.	–	–	–
**$^{Ph}YSnHMDS$**	-2186.92670619	0.660769	0.545090	0.056708	148.885588	140.960
**$^{Ph}YSnB1$**	-1988.81751873	0.663026	0.551140	0.046928	123.208880	115.283
**I**	-1022.62302873	0.331720	0.261782	0.069259	182.260736	174.335
**II**	n.o.	n.o.	n.o.	–	–	–
**III**	-2333.91204800	0.386460	0.303704	0.054469	143.338880	135.413
**IV**	-1541.89807114	0.344972	0.268504	0.046680	122.841234	114.915
**V**	-992.66893030	0.330931	0.259448	0.039106	102.673600	94.748

Table S6.6. Energies of the H_2 activated products following pathway **B**

Stannylene	E(SCF)	Corr(H)	Corr(G)	ΔG [hartree]	ΔG [kJ/mol]	1M ΔG [kJ/mol]
**$^{Tos}Y_2Sn$**	-3797.74178854	0.903412	0.750108	0.059857	157.153970	149.228
**$^{Tos}YSnCl$**	-2361.79594561	0.464515	0.365917	0.008849	23.232544	15.307
**$^{Tos}YSnHMDS$**	-2775.57104813	0.709068	0.583446	0.022225	58.351337	50.425
**$^{Tos}YSnB1$**	-2577.44789013	0.711697	0.586848	0.018427	48.381132	40.455
**$^{Ph}Y_2Sn$**	-2620.58994123	0.817045	0.683788	-0.010591	-27.807753	-35.734
**$^{Ph}YSnCl$**	-2186.98598799	0.665867	0.550652	-0.009563	-25.108818	-33.035
**$^{Ph}YSnHMDS$**	-1773.21499324	0.421749	0.334907	0.002988	7.844253	-0.082
**$^{Ph}YSnB1$**	-1988.87846260	0.668636	0.556498	-0.008658	-22.731822	-30.658
**I**	-1022.69769888	0.337176	0.268619	0.001583	4.164801	-3.761
**II**	-1571.89629800	0.351327	0.274771	0.001217	3.201558	-4.724
**III**	-2333.95557594	0.391230	0.306899	0.014229	37.444746	29.519
**IV**	-1541.93264299	0.349394	0.271380	0.015057	39.623780	31.698
**V**	-992.72470864	0.335532	0.263086	-0.013034	-34.220852	-42.147

Pathway C

Table S6.7. Energies of the transition states of the H_2 activation following pathway **C**

Stannylene	E(SCF)	Corr(H)	Corr(G)	ΔG [hartree]	ΔG [kJ/mol]	1M ΔG [kJ/mol]
**IV**	-1541.89766699	0.344035	0.267564	0.046145	121.434360	113.508
**V**	-992.68335500	0.329783	0.258895	0.024129	63.349652	55.424

Table S6.8. Energies of the H_2 activated products following pathway **C**

Stannylene	E(SCF)	Corr(H)	Corr(G)	ΔG [hartree]	ΔG [kJ/mol]	1M ΔG [kJ/mol]
**IV**	-1541.95645177	0.348178	0.271419	-0.008658	-22.783777	-30.710
**V**	-992.73003731	0.333927	0.262447	-0.019002	-49.888975	-57.815

6.1.3 Energies of the phenol activation

Pathway A

Table S6.9. Energies of the coordination of phenol following pathway **A**

Stannylene	E(SCF)	Corr(H)	Corr(G)	ΔG [hartree]	ΔG [kJ/mol]	1M ΔG [kJ/mol]
**I**	n.o.	n.o.	n.o.	–	–	–
**II**	n.o.	n.o.	n.o.	–	–	–
**III**	-2640.77340287	0.487827	0.387481	0.005521	14.527889	6.602
**IV**	-1848.75866193	0.446071	0.354871	0.001078	2.836380	-5.090
**V**	-1299.51972656	0.431924	0.345651	0.003030	7.956565	0.031

Table S6.10. Energies of the transition states of the phenol activation following pathway **A**

Stannylene	E(SCF)	Corr(H)	Corr(G)	ΔG [hartree]	ΔG [kJ/mol]	1M ΔG [kJ/mol]
**I**	n.o.	n.o.	n.o.	–	–	–
**II**	n.o.	n.o.	n.o.	–	–	–
**III**	-2640.71103440	0.480778	0.381357	0.061635	162.197745	154.272
**IV**	-1848.71184647	0.439159	0.349940	0.042866	112.804030	104.878
**V**	-1299.46790223	0.425025	0.340360	0.049564	130.129823	122.204

Table S6.11. Energies of the phenol activated products following pathway **A**

Stannylene	E(SCF)	Corr(H)	Corr(G)	ΔG [hartree]	ΔG [kJ/mol]	1M ΔG [kJ/mol]
**I**	-1329.48363273	0.428773	0.345683	0.021184	55.746716	47.821
**II**	-1878.69097650	0.443294	0.353226	0.013481	35.476465	27.551
**III**	-2640.77661450	0.483953	0.385429	0.708229	0.000269	-7.218
**IV**	-1848.77868194	0.441847	0.353700	-0.020064	-52.800617	-60.727
**V**	-1299.54007155	0.427556	0.343707	-0.019258	-50.563179	-58.489

Pathway B

Table S6.12. Energies of the coordination of phenol following pathway **B**

Stannylene	E(SCF)	Corr(H)	Corr(G)	ΔG [hartree]	ΔG [kJ/mol]	1M ΔG [kJ/mol]
**I**	-1329.50756795	0.433679	0.348474	0.000088	0.232567	-7.693
**II**	-1878.70646852	0.447574	0.355339	0.000133	0.349848	-7.576
**III**	-2640.78975236	0.487430	0.387391	-0.010881	-28.633992	-36.560
**IV**	-1848.75893407	0.445490	0.354639	0.000575	1.512761	-6.413
**V**	-1299.52451581	0.431533	0.345634	-0.001776	-4.662245	-12.588

Table S6.13. Energies of the transition states of the phenol activation following pathway **B**

Stannylene	E(SCF)	Corr(H)	Corr(G)	ΔG [hartree]	ΔG [kJ/mol]	1M ΔG [kJ/mol]
**I**	-1329.48945057	0.427565	0.343667	0.013368	35.178969	27.253
**II**	-1878.68527743	0.441268	0.349096	0.015047	39.596058	31.670
**III**	-2640.76422607	0.481478	0.382018	0.009226	24.278471	16.353
**IV**	-1848.74953999	0.440036	0.350690	0.006007	15.808818	7.883
**V**	-1299.51986875	0.426842	0.342520	-0.000243	-0.637196	-8.563

Table S6.14. Energies of the phenol activated products following pathway **B**

Stannylene	E(SCF)	Corr(H)	Corr(G)	ΔG [hartree]	ΔG [kJ/mol]	1M ΔG [kJ/mol]
**I**	-1329.52832617	0.431982	0.349258	-0.019840	-52.209748	-60.136
**II**	-1878.73604993	0.446485	0.356996	-0.027727	-72.965691	-80.892
**III**	-2640.79461493	0.486647	0.389198	-0.013929	-36.656391	-44.582
**IV**	-1848.77287817	0.444210	0.354088	-0.013887	-36.544124	-44.470
**V**	-1299.55838981	0.431518	0.345451	-0.035833	-94.078898	-102.005

Pathway C

Table S6.15. Energies of the coordination of phenol following pathway **C**

Stannylene	E(SCF)	Corr(H)	Corr(G)	ΔG [hartree]	ΔG [kJ/mol]	1M ΔG [kJ/mol]
**IV**	-1848.72208617	0.446464	0.354454	0.037153	97.771205	89.845
**V**	n.o.	n.o.	n.o.	–	–	–

Table S6.16. Energies of the transition states of the phenol activation following pathway **C**

Stannylene	E(SCF)	Corr(H)	Corr(G)	ΔG [hartree]	ΔG [kJ/mol]	1M ΔG [kJ/mol]
**IV**	-1848.67221227	0.439117	0.348066	0.080539	211.943435	204.018
**V**	-1299.47606938	0.425552	0.343604	0.044641	117.204092	109.278

Table S6.17. Energies of the phenol activated products following pathway **C**

Stannylene	E(SCF)	Corr(H)	Corr(G)	ΔG [hartree]	ΔG [kJ/mol]	1M ΔG [kJ/mol]
**IV**[a)]	-1848.80905576	0.441764	0.352404	-0.051661	-135.949729	-143.876
**V**	-1299.57385574	0.428127	0.343003	-0.053747	-141.111921	-149.038

a) Decomposition was observed.

6.2 Coordinates of the Structures

6.2.1 Singlet state structures

$^{Tos}Y_2Sn$

E = -3796.60560565
C 4.537103 2.550899 -0.221864
H 4.437006 2.798845 0.831875
C 3.498330 1.888199 -0.854263
C -3.630084 -1.787957 -0.983899
O 0.890560 2.107372 -0.602656
C -3.703080 -1.333571 -2.297973
H -2.842401 -0.838679 -2.748830
C 2.768315 -2.649324 3.065715
H 3.836078 -2.776051 2.893313
C 5.685070 2.862388 -0.944771
H 6.507287 3.375820 -0.447543
C -1.857387 5.208492 -0.640013
H -1.597950 6.169289 -0.200224
C -2.038443 4.102537 0.182282
H -1.911530 4.198007 1.258061
C 2.538278 -2.667970 -1.976593
H 2.606177 -1.649682 -2.356757
C 2.502451 -2.882043 -0.595690

C 5.247191 -1.177292 -0.285773
H 4.951406 -1.704607 -1.190483
C 6.540880 -0.688294 -0.160932
H 7.253766 -0.835325 -0.969821
C -1.988024 5.084976 -2.018515
H -1.836007 5.951759 -2.658951
C -1.730702 0.128349 -0.175612
Sn -0.034658 0.012286 -1.634031
O -2.443372 -1.986968 1.314812
S -2.143115 -1.478078 -0.034818
P -2.542498 1.369445 0.630021
C -4.875844 -1.505747 -3.013469
H -4.934912 -1.148661 -4.041159
C -4.718021 0.240118 1.940171
H -3.995335 -0.045626 2.701235
C -0.588019 1.461440 2.531506
H -0.013474 0.940349 1.771803
C 6.003952 0.185777 2.019215
H 6.295371 0.726574 2.917463
C 4.704667 -0.292997 1.897101
H 3.977473 -0.108027 2.684232
C -2.476596 2.741665 -1.764963
H -2.697117 1.766087 -2.196667
C 1.968859 -2.014629 2.110736
C 3.587012 1.548185 -2.201291
H 2.758833 1.029172 -2.684538
C 2.391964 -4.180623 -0.098778
H 2.338893 -4.351413 0.974002
C 4.325914 -0.992576 0.748069
C -2.092967 2.792163 4.457836
H -2.680350 3.307296 5.215746
C -1.917426 1.785040 2.278063
C -2.675196 2.457704 3.241553
H -3.715699 2.711792 3.043391
C -2.355403 2.865915 -0.377431
C -2.298621 3.850987 -2.581095
H -2.389660 3.748357 -3.660514
C 7.059843 2.820188 -3.057570
H 6.834363 3.282333 -4.026287
H 7.625748 1.901459 -3.264392
H 7.717774 3.496745 -2.502346
C -0.758440 2.472883 4.705585
H -0.306933 2.744784 5.658225
C -5.988934 -2.131871 -2.436742
C 6.922615 -0.013511 0.994733
H 7.937605 0.367348 1.091923
C -6.549400 0.906915 -0.050234
H -7.261644 1.162439 -0.832271
C 1.702998 -0.193118 -0.218182
P 2.581729 -1.441109 0.504215
O 2.345014 1.797762 1.465570
S 2.043436 1.411413 0.076003
O -1.031061 -2.150929 -0.798554
C 5.807992 2.518541 -2.289633
C 4.735616 1.862055 -2.908022
H 4.807514 1.594048 -3.961656
C 2.473411 -3.746634 -2.849446
H 2.495067 -3.574374 -3.923594
C 2.366959 -5.040710 -2.349969
H 2.305971 -5.884541 -3.034654

C 2.325747 -5.255719 -0.976804
H 2.228966 -6.266134 -0.584919
C -5.230205 1.327950 -0.156414
H -4.915470 1.911340 -1.019058
C -0.003951 1.814528 3.741681
H 1.044692 1.579265 3.906166
C -4.310463 1.005918 0.844241
C -6.957943 0.163272 1.052663
H -7.992793 -0.163887 1.134908
C -5.882196 -2.592248 -1.126018
H -6.736141 -3.084087 -0.661553
C -6.041702 -0.171525 2.043554
H -6.355060 -0.764516 2.900535
C 0.822842 -2.961807 4.457776
H 0.377604 -3.333383 5.379181
C 2.191492 -3.113415 4.242024
H 2.811617 -3.599122 4.993265
C -4.709750 -2.423892 -0.394369
H -4.621083 -2.763831 0.634406
C -7.264186 -2.279527 -3.211169
H -7.969913 -2.941915 -2.699272
H -7.079977 -2.686340 -4.212922
H -7.757933 -1.307334 -3.345677
C 0.026043 -2.343447 3.500423
H -1.047254 -2.234842 3.636612
C 0.602142 -1.862814 2.331650
H -0.008639 -1.374279 1.578900

^{Tos}YSnCl
E = -2360.61257775
C 3.305917 -0.260172 -1.489673
H 2.974507 0.146956 -2.441718
C 2.404025 -0.962571 -0.708286
O 0.478749 -2.642912 -1.286312
C -2.021413 3.081591 -1.058605
H -1.439113 3.747936 -0.422460
C 4.606716 -0.082798 -1.027742
H 5.318093 0.474482 -1.635758
C -0.891830 0.285309 2.873827
H -0.140203 -0.461267 2.623212
C -1.374683 1.128866 1.868038
C 1.723061 1.979479 1.148436
H 1.457439 1.508485 2.093079
C 2.955087 2.603484 1.009068
H 3.649432 2.621424 1.846637
Sn -1.142599 -2.749971 0.393117
C 2.418384 3.161767 -1.273040
H 2.693138 3.613278 -2.224204
C 1.186819 2.528604 -1.144152
H 0.515008 2.460076 -1.996760
C -1.849040 1.695023 -0.972523
C 2.786112 -1.502097 0.516532
H 2.061275 -2.052329 1.115233
C -2.359721 2.069974 2.171796
H -2.763400 2.711926 1.392951
C 0.827453 1.952681 0.076640
C 6.405570 -0.378516 0.713183
H 6.862460 -1.319694 1.042266
H 6.404833 0.297591 1.578892
H 7.050176 0.062807 -0.053687

C 3.299231 3.204168 -0.198922
H 4.264444 3.695604 -0.305556
C -0.332340 -0.692690 -0.026153
P -0.684761 0.949975 0.198239
O 0.624476 -0.410604 -2.523981
S 0.713196 -1.137483 -1.255098
C 5.013611 -0.596932 0.201892
C 4.082439 -1.315786 0.963960
H 4.384891 -1.734818 1.922956
C -1.380901 0.393130 4.169884
H -1.002901 -0.268353 4.946755
C -2.356655 1.338104 4.469407
H -2.743597 1.418879 5.483347
C -2.846059 2.172388 3.469940
H -3.619129 2.903074 3.698515
C -3.644481 2.757084 -2.807034
H -4.347583 3.172907 -3.526487
C -2.921607 3.609192 -1.973946
H -3.055464 4.686878 -2.044401
C -3.462582 1.381785 -2.728848
H -4.019936 0.714223 -3.382417
C -2.560693 0.844085 -1.815980
H -2.413352 -0.233216 -1.755383
Cl -3.117714 -2.627118 -1.093336

^{Tos}YSnHMDS
E = -2774.39859831
S -0.765049 -1.396195 -0.698345
P -0.899923 1.411961 -0.068207
Si 3.910810 0.475986 0.538449
Si 3.505432 -2.193609 -0.915707
O 0.167605 -2.500253 -0.207670
O -0.814341 -1.178032 -2.150572
N 2.991346 -0.956357 0.204330
C -0.304659 -0.131066 0.279772
C -2.394681 -1.971126 -0.243055
C -2.687379 -2.193634 1.099451
H -1.916914 -2.040269 1.854709
C -3.963978 -2.591743 1.457873
H -4.196788 -2.766487 2.507640
C -4.962788 -2.771118 0.491640
C -4.635992 -2.556199 -0.846070
H -5.398027 -2.696755 -1.611544
C -3.356819 -2.157202 -1.221622
H -3.093705 -1.979009 -2.261253
C -6.351279 -3.163172 0.898035
H -6.339452 -3.988715 1.619574
H -6.950153 -3.472145 0.035224
H -6.870642 -2.323121 1.379475
C -2.662250 1.374067 -0.523044
C -3.630865 1.243733 0.475295
H -3.342985 1.291223 1.523990
C -4.961807 1.049491 0.131739
H -5.712040 0.949282 0.913528
C -5.333967 0.984260 -1.208176
H -6.378367 0.834504 -1.475234
C -4.370278 1.094583 -2.203459
H -4.655369 1.025528 -3.251332
C -3.034037 1.279014 -1.866342
H -2.277166 1.327756 -2.645605

C -0.103108 2.260084 -1.461527
C -0.616846 3.448169 -1.993246
H -1.496808 3.912170 -1.548259
C -0.025413 4.018963 -3.111397
H -0.424003 4.943225 -3.524968
C 1.066400 3.395882 -3.712665
H 1.522047 3.836759 -4.597307
C 1.565632 2.206762 -3.195300
H 2.407448 1.712162 -3.675366
C 0.985488 1.634459 -2.067012
H 1.360604 0.696655 -1.655215
C -0.710699 2.421369 1.427812
C -0.861205 1.783242 2.662731
H -1.087978 0.718685 2.685513
C -0.695960 2.499149 3.842035
H -0.809361 1.995531 4.799870
C -0.373743 3.851586 3.796400
H -0.235270 4.410057 4.720122
C -0.217187 4.487516 2.569433
H 0.048144 5.541994 2.530887
C -0.383798 3.777103 1.386519
H -0.230135 4.276713 0.433549
C 4.606993 1.306913 -1.001736
H 5.191196 2.192656 -0.717156
H 3.798576 1.640141 -1.661650
H 5.264247 0.649242 -1.581548
C 2.766911 1.745952 1.342214
H 3.336624 2.628826 1.661602
H 2.234951 1.378540 2.233889
H 2.013928 2.083532 0.619895
C 5.333775 0.146695 1.726684
H 6.057989 -0.551663 1.289234
H 4.961793 -0.303281 2.656812
H 5.868895 1.070145 1.986084
C 2.831482 -1.821730 -2.628544
H 3.209687 -0.849110 -2.974334
H 1.735509 -1.768126 -2.601076
H 3.121158 -2.579269 -3.368681
C 2.948533 -3.900462 -0.350992
H 3.384944 -4.660604 -1.013425
H 1.860412 -4.020189 -0.378597
H 3.307046 -4.112222 0.666194
C 5.388919 -2.311900 -1.002592
H 5.793492 -2.648482 -0.039052
H 5.887875 -1.371919 -1.264368
H 5.673158 -3.056508 -1.758659
Sn 1.287795 -1.304762 1.453911

$^{Tos}YSnC_6F_5$
E = -2628.93794305
C 3.427937 -1.639666 -1.783116
H 2.953577 -1.413948 -2.734892
C 2.641006 -1.752955 -0.649235
O 0.246979 -2.708550 -0.153877
C -0.413664 3.486694 -1.409918
H 0.348140 4.105718 -0.936566
C 4.805033 -1.806842 -1.670457
H 5.428828 -1.712359 -2.558266
C 1.087486 1.560266 2.783817
H 1.658902 0.645027 2.637864

C 0.447434 2.151676 1.688549
C 3.409128 1.617134 0.198039
H 3.302252 1.515286 1.275452
C 4.676749 1.695235 -0.361034
H 5.553467 1.656651 0.282306
C 3.702905 1.854678 -2.558172
H 3.814738 1.938098 -3.637329
C 2.429585 1.772509 -2.004316
H 1.554697 1.774260 -2.650453
C -0.503313 2.123964 -1.106257
C 3.207375 -2.041560 0.589674
H 2.572299 -2.127287 1.470823
C -0.347062 3.281438 1.885363
H -0.899666 3.715040 1.056783
C 2.277180 1.668770 -0.619655
C 6.886948 -2.241266 -0.317067
H 7.148018 -3.187128 0.173383
H 7.318353 -1.435174 0.291390
H 7.376495 -2.220282 -1.295937
C 4.825114 1.820627 -1.739711
H 5.820196 1.881497 -2.176228
C 0.417462 -0.315797 0.361426
P 0.626490 1.337318 0.075829
O 0.614895 -1.188515 -2.174214
S 0.883616 -1.460856 -0.757994
C 5.401550 -2.084743 -0.442188
C 4.578217 -2.205060 0.685883
H 5.025073 -2.431884 1.653154
C 0.966119 2.118293 4.049958
H 1.467863 1.652418 4.895601
C 0.191985 3.259108 4.236282
H 0.090723 3.692554 5.229333
C -0.469449 3.832086 3.156108
H -1.098055 4.707910 3.302777
C -2.248666 3.252496 -2.952219
H -2.939586 3.696489 -3.666241
C -1.290518 4.048675 -2.327670
H -1.226421 5.109894 -2.559756
C -2.313996 1.892353 -2.677430
H -3.051610 1.266750 -3.176086
C -1.434825 1.323160 -1.761759
H -1.445807 0.253891 -1.565299
Sn -0.679624 -1.721686 1.745673
C -2.640282 -1.325072 0.651871
C -4.469809 -1.991126 -0.801330
C -4.199497 0.331528 -0.213750
C -4.884231 -0.667056 -0.896438
C -3.094165 -0.025826 0.544671
C -3.353896 -2.294122 -0.027869
F -2.980306 -3.572304 0.037785
F -2.422207 0.963908 1.157801
F -5.136474 -2.932698 -1.452477
F -5.933681 -0.355094 -1.640854
F -4.584722 1.595490 -0.330320

^{Tos}YSnPy
E = -2148.07780408
C 3.598572 -0.347981 -1.640540
H 3.235682 0.165625 -2.527297
C 2.706855 -1.085284 -0.879901

O 0.607125 -2.575012 -1.418975
C -1.527236 3.280963 -0.647654
H -1.010748 3.834853 0.136135
C 4.929429 -0.270952 -1.239558
H 5.632560 0.315044 -1.830127
C -0.369341 0.213042 2.968477
H 0.419447 -0.480645 2.682348
C -0.897128 1.080637 2.007112
C 2.299900 1.775835 1.253773
H 2.049181 1.225867 2.158646
C 3.566103 2.325054 1.109489
H 4.299505 2.207494 1.904730
C 2.964265 3.149625 -1.072185
H 3.225402 3.675507 -1.988434
C 1.695298 2.595704 -0.936163
H 0.979327 2.668223 -1.751551
C -1.326640 1.902451 -0.782897
C 3.127225 -1.754884 0.266425
H 2.408888 -2.326650 0.852937
C -1.946522 1.931920 2.353267
H -2.394672 2.582829 1.607824
C 1.351769 1.921672 0.237493
C 6.805352 -0.822299 0.352085
H 7.264068 -1.815579 0.437099
H 6.882680 -0.348764 1.339669
H 7.403577 -0.233496 -0.350745
C 3.897053 3.020321 -0.050884
H 4.890822 3.449917 -0.162427
C 0.065206 -0.639105 0.001045
P -0.212540 0.991404 0.327120
O 0.867420 -0.294864 -2.535884
S 0.975309 -1.106890 -1.315690
C 5.376360 -0.919752 -0.090538
C 4.453149 -1.667741 0.653294
H 4.785671 -2.188479 1.550684
C -0.870909 0.215856 4.263737
H -0.456345 -0.465236 5.004163
C -1.910056 1.074482 4.606929
H -2.308565 1.070746 5.619641
C -2.448413 1.927092 3.649880
H -3.272522 2.588563 3.909713
C -3.002952 3.245360 -2.549768
H -3.662657 3.770028 -3.238562
C -2.369904 3.948118 -1.525941
H -2.528256 5.019497 -1.418727
C -2.780315 1.882464 -2.702251
H -3.260838 1.333441 -3.509414
C -1.936943 1.209117 -1.824570
H -1.730881 0.149143 -1.950455
Sn -0.920523 -2.615614 0.448971
C -2.801214 -1.857667 -0.512082
N -3.177937 -0.985352 0.439349
C -4.701910 -1.256341 -1.854830
C -4.277408 -0.257872 0.271603
C -5.074691 -0.353946 -0.863792
H -5.302292 -1.359110 -2.758724
H -4.532871 0.438402 1.074232
H -5.961832 0.267130 -0.967876
C -3.554627 -2.021755 -1.675532
H -3.237786 -2.732783 -2.437513

^{Tos}YSnPyr
E = -2164.13503634
C 3.628531 -0.310313 -1.593425
H 3.275469 0.223307 -2.472214
C 2.729631 -1.068311 -0.861903
O 0.631269 -2.536479 -1.477001
C -1.592608 3.222913 -0.712168
H -1.099912 3.804094 0.067009
C 4.953412 -0.237107 -1.172659
H 5.661690 0.365533 -1.739831
C -0.390545 0.222728 2.961261
H 0.387230 -0.483560 2.675760
C -0.909845 1.090907 1.996381
C 2.256452 1.828896 1.249955
H 2.006142 1.290400 2.161908
C 3.512503 2.401980 1.109850
H 4.238688 2.315153 1.915514
C 2.917681 3.176530 -1.092387
H 3.178252 3.691916 -2.014725
C 1.659485 2.597440 -0.961378
H 0.952524 2.635310 -1.786846
C -1.356327 1.847711 -0.819011
C 3.137849 -1.764412 0.273044
H 2.414837 -2.352686 0.837015
C -1.942211 1.963047 2.341915
H -2.383620 2.615932 1.594234
C 1.317764 1.936738 0.220232
C 6.810582 -0.819969 0.429905
H 7.275298 -1.812675 0.484019
H 6.872751 -0.382599 1.434994
H 7.412920 -0.202091 -0.243822
C 3.841340 3.084423 -0.058693
H 4.826750 3.533766 -0.166614
C 0.077247 -0.649995 0.006243
P -0.229869 0.980824 0.315988
O 0.908389 -0.214426 -2.504341
S 1.003504 -1.074960 -1.316712
C 5.387812 -0.911267 -0.033507
C 4.458194 -1.681323 0.679263
H 4.781002 -2.222318 1.568149
C -0.884934 0.243843 4.259322
H -0.477053 -0.437702 5.003062
C -1.907567 1.122163 4.601715
H -2.299985 1.133256 5.616737
C -2.436584 1.977001 3.641171
H -3.246883 2.655379 3.900982
C -3.033931 3.120330 -2.638331
H -3.692800 3.617354 -3.348074
C -2.436753 3.854544 -1.615619
H -2.621797 4.923541 -1.529593
C -2.773295 1.761342 -2.765287
H -3.219881 1.189098 -3.575812
C -1.929978 1.123398 -1.861525
H -1.692193 0.068486 -1.974235
Sn -0.889308 -2.654011 0.388090
C -2.820725 -1.803320 -0.420634
N -3.520173 -2.034534 -1.534967
N -3.184960 -0.917621 0.524628
C -4.627576 -1.320943 -1.716527

C -4.294982 -0.216170 0.339375
C -5.073671 -0.376601 -0.799677
H -5.188574 -1.509833 -2.635980
H -4.567931 0.500579 1.118709
H -5.978593 0.203011 -0.965566

^{Tos}YSnB2
E = -2307.08730068
C 3.299142 -1.378198 -1.943517
H 2.728869 -1.210480 -2.853857
C 2.619742 -1.579731 -0.753841
O 0.403428 -2.821044 -0.110595
C -1.054055 3.240999 -1.261301
H -0.284432 3.936491 -0.927067
C 4.691047 -1.382344 -1.937560
H 5.230198 -1.217040 -2.869483
C 0.949204 1.468965 2.835605
H 1.529838 0.566856 2.649461
C 0.225507 2.043486 1.784118
C 3.059633 1.841003 0.040375
H 3.055247 1.716185 1.121342
C 4.256172 2.074944 -0.622441
H 5.183625 2.137099 -0.056754
Sn -0.518138 -1.997401 1.895426
C 3.085328 2.125604 -2.727585
H 3.094434 2.225015 -3.811160
C 1.883045 1.887902 -2.069629
H 0.962045 1.783623 -2.638540
C -0.992521 1.892055 -0.892918
C 3.308706 -1.796452 0.437019
H 2.755153 -1.953611 1.362361
C -0.562595 3.169058 2.027913
H -1.161277 3.603529 1.232406
C 1.864438 1.761796 -0.679021
C 6.906683 -1.565657 -0.748860
H 7.312910 -2.485710 -0.310403
H 7.287379 -0.729441 -0.147352
H 7.314098 -1.463960 -1.759869
C 4.269072 2.224340 -2.006890
H 5.208750 2.406569 -2.524899
C 0.337556 -0.424529 0.453429
P 0.336495 1.236552 0.159460
O 0.432246 -1.227857 -2.109402
S 0.835572 -1.497466 -0.723812
C 5.407756 -1.583653 -0.759734
C 4.692835 -1.796250 0.427060
H 5.236115 -1.964693 1.356267
C 0.902898 2.030663 4.105266
H 1.467101 1.575843 4.916881
C 0.126343 3.160965 4.339208
H 0.084424 3.596729 5.335586
C -0.608535 3.724140 3.302028
H -1.231706 4.597041 3.485427
C -3.055077 2.793406 -2.526061
H -3.867471 3.147869 -3.157768
C -2.088114 3.689005 -2.072040
H -2.138682 4.738391 -2.356194
C -2.976645 1.447803 -2.189533
H -3.721201 0.742400 -2.553036
C -1.939359 0.994742 -1.379841

H -1.832327 -0.062161 -1.147805
C -4.423670 -0.722157 0.111251
C -4.079586 -1.699999 -0.818204
C -5.532152 0.085301 -0.055915
C -4.827918 -1.919617 -1.958998
C -6.296042 -0.127755 -1.205858
H -5.784904 0.850434 0.673144
C -5.952310 -1.109274 -2.136820
H -4.543389 -2.684212 -2.676782
H -7.178145 0.485319 -1.378706
H -6.572190 -1.246891 -3.020361
B -2.558163 -1.704311 0.783547
O -3.499046 -0.722507 1.108961
O -2.940692 -2.317562 -0.406841

^{Tos}YSnB1
E = -2576.27485486
C 4.037392 0.657534 0.763716
H 3.756605 0.936675 1.776201
C 3.075520 0.690858 -0.232279
O 1.075909 2.293112 -0.754259
C -1.049223 -2.504475 2.797215
H -0.585587 -3.459562 2.555429
C 5.329760 0.253319 0.443650
H 6.087731 0.217053 1.225233
C -0.266485 -2.562255 -2.063214
H 0.309142 -1.698464 -2.390196
C -0.569458 -2.704613 -0.704667
C 2.590712 -2.764540 0.121032
H 2.262059 -2.948306 -0.900602
C 3.868299 -3.135043 0.517194
H 4.536669 -3.615644 -0.194602
Sn -0.614002 1.255645 -2.063476
C 3.445317 -2.255179 2.720041
H 3.781766 -2.044050 3.733194
C 2.165547 -1.876659 2.327722
H 1.514092 -1.355071 3.025016
C -0.867319 -1.398039 1.958997
C 3.389929 0.344805 -1.543660
H 2.618188 0.379907 -2.312065
C -1.317474 -3.804137 -0.282824
H -1.597902 -3.913598 0.759855
C 1.727214 -2.147911 1.030330
C 7.049359 -0.600214 -1.188014
H 7.399008 -0.193988 -2.144291
H 7.068552 -1.695602 -1.277123
H 7.770453 -0.320701 -0.412792
C 4.293919 -2.887755 1.819854
H 5.296188 -3.179115 2.128451
C 0.350056 -0.043746 -0.412627
P 0.136058 -1.481851 0.441011
O 1.385633 1.395263 1.612690
S 1.389483 1.126518 0.166188
C 5.669613 -0.116960 -0.856375
C 4.680428 -0.055089 -1.846717
H 4.931102 -0.327982 -2.871219
C -0.698900 -3.512486 -2.979919
H -0.462569 -3.388148 -4.034844
C -1.424419 -4.618396 -2.549497
H -1.755834 -5.367027 -3.266733

C -1.732693 -4.760630 -1.201871
H -2.315087 -5.614472 -0.861390
C -2.316757 -1.140991 4.323598
H -2.886011 -1.040631 5.246045
C -1.784972 -2.378939 3.967256
H -1.929460 -3.243191 4.612740
C -2.097797 -0.031550 3.516754
H -2.489486 0.942920 3.798403
C -1.373541 -0.155544 2.334184
H -1.177958 0.713373 1.709138
N -3.328637 1.096608 0.009269
C -3.141185 2.848872 1.386817
C -3.837245 1.715168 1.147943
H -3.300856 3.595210 2.157430
H -4.695837 1.326777 1.683003
B -2.230694 1.895022 -0.504972
C -1.195500 4.023350 0.418650
C -0.921029 4.725604 -0.753773
C -0.454151 4.280748 1.569686
C 0.107248 5.657806 -0.780844
H -1.519889 4.525970 -1.640799
C 0.562883 5.225243 1.539720
H -0.640754 3.696094 2.467732
C 0.852128 5.912003 0.365483
H 0.325244 6.192025 -1.703691
H 1.152872 5.405052 2.436366
H 1.660708 6.639749 0.342457
C -3.702310 -0.187507 -0.404169
C -3.588439 -0.551009 -1.750807
C -4.184355 -1.134252 0.507154
C -3.943525 -1.825485 -2.172340
H -3.262628 0.191058 -2.477923
C -4.564352 -2.396949 0.072469
H -4.241699 -0.883711 1.563399
C -4.448434 -2.751885 -1.267055
H -3.841886 -2.087359 -3.223870
H -4.942204 -3.116016 0.797419
H -4.737081 -3.745708 -1.601495
N -2.170452 3.005839 0.405057

$^{Ph}Y_2Sn$
E = -2619.38479815
P 3.163701 0.240127 0.002554
P -3.163835 0.344390 -0.014992
C 1.718834 -0.360411 -0.669916
C 1.629782 -1.791833 -0.949426
C 2.514771 -2.758517 -0.425251
H 3.304113 -2.453200 0.259196
C 2.402114 -4.106702 -0.737060
H 3.110999 -4.813139 -0.306823
C 1.386217 -4.558727 -1.570763
H 1.287005 -5.616807 -1.804089
C 0.490034 -3.628927 -2.093066
H -0.323453 -3.959122 -2.737353
C 0.618234 -2.282013 -1.800297
H -0.089992 -1.569093 -2.222122
C 3.459141 -0.219035 1.759736
C 4.525967 -1.001813 2.202178
H 5.305421 -1.307404 1.507469
C 4.588203 -1.415324 3.530043

H 5.419964 -2.033617 3.862341
C 3.591751 -1.047851 4.426036
H 3.641817 -1.375183 5.462740
C 2.520098 -0.272107 3.990212
H 1.729107 0.008376 4.683005
C 2.449399 0.126595 2.663080
H 1.592522 0.704269 2.309472
C 3.197016 2.060594 -0.021918
C 3.507697 2.838862 1.091978
H 3.691659 2.369115 2.055169
C 3.576727 4.224598 0.974958
H 3.810667 4.827437 1.850314
C 3.348625 4.834717 -0.251922
H 3.402033 5.918315 -0.339581
C 3.058646 4.058624 -1.371471
H 2.884469 4.532558 -2.335251
C 2.987394 2.678413 -1.259689
H 2.756211 2.062722 -2.127734
C 4.707877 -0.177419 -0.881185
C 4.650888 -0.886109 -2.081057
H 3.691658 -1.248629 -2.443007
C 5.816551 -1.118141 -2.803920
H 5.769070 -1.678742 -3.735381
C 7.035680 -0.634567 -2.341691
H 7.945137 -0.816822 -2.911371
C 7.092738 0.093304 -1.156210
H 8.042866 0.487922 -0.801217
C 5.931687 0.324969 -0.429934
H 5.972291 0.910552 0.488599
C -1.591292 -0.298165 0.166520
C -1.481968 -1.729183 0.469802
C -0.472392 -2.192513 1.332892
H 0.219128 -1.466790 1.758119
C -0.322792 -3.539085 1.621944
H 0.486436 -3.856549 2.277759
C -1.193304 -4.482784 1.081221
H -1.075263 -5.539692 1.312018
C -2.203089 -4.053171 0.229062
H -2.885140 -4.775132 -0.218587
C -2.335034 -2.704917 -0.078841
H -3.096173 -2.400996 -0.796360
C -4.372373 -0.338777 1.180114
C -4.361457 0.127224 2.496868
H -3.705059 0.945991 2.779488
C -5.189320 -0.443630 3.454736
H -5.173668 -0.065504 4.475020
C -6.032917 -1.494912 3.111270
H -6.678888 -1.944902 3.862777
C -6.045676 -1.970400 1.805537
H -6.697140 -2.797458 1.530400
C -5.220970 -1.396989 0.844523
H -5.241339 -1.779025 -0.173001
C -3.146360 2.153631 0.241922
C -3.825788 3.040748 -0.593087
H -4.368406 2.671679 -1.458937
C -3.793994 4.408289 -0.338333
H -4.319061 5.090682 -1.003829
C -3.089172 4.901615 0.752556
H -3.065296 5.972044 0.948142
C -2.404412 4.024180 1.587524

H -1.837220 4.404379 2.434720
C -2.427103 2.660645 1.330742
H -1.863251 1.972359 1.957630
C -4.003883 0.120835 -1.635348
C -5.368494 0.362500 -1.825203
H -5.986440 0.678702 -0.985341
C -5.937667 0.195605 -3.081433
H -6.999615 0.386106 -3.225381
C -5.151029 -0.218564 -4.154164
H -5.600301 -0.352869 -5.136465
C -3.795883 -0.466885 -3.969003
H -3.180792 -0.798258 -4.803309
C -3.224848 -0.297893 -2.711713
H -2.166728 -0.499331 -2.541936
Sn 0.028071 1.046915 -0.444041

^{Ph}YSnCl
E = -1772.01021413
P -0.796094 0.005927 -0.037313
C 0.912514 -0.001044 -0.064913
C 1.553586 1.333131 -0.001147
C 1.600496 2.076944 1.187311
H 1.198355 1.639305 2.099186
C 2.143001 3.356312 1.216891
H 2.162011 3.911832 2.153302
C 2.667559 3.922503 0.059297
H 3.095101 4.923192 0.081285
C 2.654110 3.189121 -1.122851
H 3.073855 3.614842 -2.032896
C 2.106324 1.913040 -1.151493
H 2.088857 1.344832 -2.079362
C -1.568606 0.385249 1.578097
C -2.773608 1.081272 1.689499
H -3.274311 1.451075 0.796829
C -3.331090 1.306745 2.943769
H -4.266934 1.855738 3.028255
C -2.693270 0.835638 4.086778
H -3.130020 1.017882 5.066882
C -1.493949 0.137167 3.978769
H -0.991558 -0.228838 4.871978
C -0.931662 -0.083913 2.728040
H 0.016304 -0.613085 2.627065
C -1.419852 -1.637639 -0.489023
C -2.324576 -2.339004 0.308549
H -2.687134 -1.900549 1.235547
C -2.754641 -3.604841 -0.076394
H -3.451814 -4.151597 0.555127
C -2.293407 -4.170832 -1.259453
H -2.631277 -5.161985 -1.555798
C -1.399733 -3.471275 -2.064920
H -1.038189 -3.911219 -2.991952
C -0.962100 -2.209921 -1.682739
H -0.259590 -1.658202 -2.305381
C -1.555989 1.210286 -1.174006
C -1.258168 2.568869 -1.004266
H -0.624494 2.894781 -0.181761
C -1.772117 3.507968 -1.888612
H -1.529092 4.559805 -1.753149
C -2.589155 3.107189 -2.941538
H -2.989019 3.846426 -3.633007

C -2.897183 1.761826 -3.106445
H -3.542667 1.444170 -3.922973
C -2.382785 0.814256 -2.227471
H -2.633039 -0.235420 -2.363399
Sn 1.979670 -1.841795 0.046783
Cl 4.174022 -0.739790 0.082024

^{Ph}YSnHMDS
E = -2185.79301904
P 1.945694 0.047339 -0.040414
C 0.283740 -0.249504 -0.324274
C -0.142801 -1.616460 -0.652910
C 0.453366 -2.769515 -0.105503
H 1.283768 -2.668280 0.590042
C -0.017881 -4.042146 -0.400220
H 0.465512 -4.907776 0.050294
C -1.108195 -4.214517 -1.247409
H -1.485778 -5.210904 -1.468120
C -1.698461 -3.090493 -1.819764
H -2.541479 -3.204280 -2.500157
C -1.215873 -1.820320 -1.538968
H -1.666538 -0.950903 -2.006514
C 2.656844 -0.695966 1.479816
C 3.867409 -1.388420 1.520953
H 4.467650 -1.499142 0.621222
C 4.302313 -1.958256 2.713944
H 5.243679 -2.503799 2.737488
C 3.537472 -1.838039 3.868697
H 3.879995 -2.288575 4.798236
C 2.326107 -1.152932 3.831149
H 1.716578 -1.066717 4.728394
C 1.883759 -0.594741 2.639835
H 0.920172 -0.085137 2.595456
C 2.218751 1.835693 0.135128
C 2.689634 2.428592 1.304985
H 2.933238 1.816163 2.169817
C 2.838549 3.811204 1.369715
H 3.196863 4.271504 2.288161
C 2.527046 4.600180 0.269279
H 2.641762 5.680960 0.325358
C 2.071980 4.009380 -0.906831
H 1.832908 4.624944 -1.771509
C 1.919107 2.632168 -0.976489
H 1.560070 2.163304 -1.891687
C 3.071825 -0.424328 -1.393400
C 2.636546 -1.250450 -2.430403
H 1.623093 -1.642661 -2.422849
C 3.501363 -1.561824 -3.474744
H 3.158399 -2.208141 -4.280058
C 4.792686 -1.047046 -3.494457
H 5.463883 -1.291546 -4.315634
C 5.224320 -0.208369 -2.470346
H 6.229203 0.208815 -2.491037
C 4.366095 0.105651 -1.424868
H 4.697233 0.777097 -0.632850
Sn -1.038580 1.306995 0.388386
N -2.911908 0.277371 0.057081
Si -3.889715 0.890925 -1.236423
Si -3.515067 -0.699806 1.361500
C -2.806679 2.001279 -2.321821

H -2.470451 2.908456 -1.799822
H -1.915917 1.465232 -2.683055
H -3.368461 2.329180 -3.206902
C -4.593650 -0.426201 -2.385923
H -5.276714 -1.110385 -1.871190
H -5.158007 0.061952 -3.192713
H -3.803358 -1.027796 -2.850533
C -5.334610 1.921410 -0.602893
H -5.920787 2.359024 -1.421890
H -6.015737 1.309784 0.004105
H -4.972041 2.738383 0.034842
C -4.990751 -1.750878 0.856359
H -5.335267 -2.339965 1.716782
H -5.839525 -1.151512 0.503386
H -4.707125 -2.450359 0.059602
C -4.036988 0.409729 2.797972
H -4.416821 -0.164558 3.653653
H -3.187507 1.011914 3.151096
H -4.820852 1.110624 2.481726
C -2.197377 -1.878172 2.003236
H -1.222310 -1.393836 2.155561
H -2.510099 -2.308407 2.964313
H -2.039201 -2.699974 1.294190

$^{Ph}YSnC_6F_5$
E = -2040.33947504
P -1.884877 0.175653 0.044675
C -0.262070 -0.091506 -0.421988
C 0.640181 1.078580 -0.288583
C 1.270149 1.404321 0.922763
H 1.081871 0.783257 1.797036
C 2.152019 2.475057 1.012644
H 2.638836 2.693056 1.961974
C 2.428334 3.252345 -0.108399
H 3.129904 4.081585 -0.042687
C 1.805894 2.951520 -1.315698
H 2.018235 3.547666 -2.201487
C 0.919597 1.884616 -1.401859
H 0.443649 1.642995 -2.350063
C -2.146573 0.261472 1.854108
C -3.185799 1.009184 2.414093
H -3.844234 1.592836 1.773174
C -3.376442 1.011541 3.791059
H -4.184782 1.597605 4.223995
C -2.533009 0.270591 4.613682
H -2.680858 0.278916 5.691933
C -1.497244 -0.474686 4.059555
H -0.832176 -1.049991 4.700415
C -1.302870 -0.478585 2.683285
H -0.483313 -1.046243 2.242379
C -2.950309 -1.167850 -0.543309
C -3.844908 -1.826098 0.301319
H -3.912417 -1.544493 1.349706
C -4.643069 -2.851090 -0.195315
H -5.332371 -3.367339 0.469597
C -4.556819 -3.218447 -1.533346
H -5.181448 -4.022453 -1.917779
C -3.669895 -2.561430 -2.381210
H -3.598591 -2.849111 -3.428031
C -2.867071 -1.540357 -1.890247

H -2.163734 -1.027944 -2.544635
C -2.584545 1.742943 -0.561476
C -2.020725 2.938286 -0.098522
H -1.225586 2.915727 0.645079
C -2.473549 4.155529 -0.588921
H -2.026014 5.079695 -0.229275
C -3.491893 4.192406 -1.537144
H -3.845072 5.148164 -1.919569
C -4.060092 3.008378 -1.992908
H -4.861620 3.034011 -2.728475
C -3.608466 1.784847 -1.509042
H -4.060883 0.863022 -1.867788
C 2.543670 -1.279483 -0.425462
C 4.225729 -0.673484 1.222389
C 4.356246 0.228600 -1.009026
C 4.868670 0.118775 0.278961
Sn 0.435890 -2.074665 -0.782821
C 3.210501 -0.484413 -1.335916
C 3.072216 -1.348852 0.846577
F 2.419470 -2.038328 1.800469
F 2.721698 -0.336161 -2.573206
F 4.958204 1.014608 -1.889972
F 5.962763 0.785775 0.612563
F 4.703654 -0.746864 2.458261

^{Ph}YSnPy
E = -1559.47095129
P -1.303940 0.074371 -0.013070
C 0.376834 -0.150413 -0.197882
C 1.188718 1.089060 -0.145129
C 1.791300 1.534126 1.040634
C 2.595524 2.668019 1.062173
C 2.809508 3.397996 -0.102622
C 2.210552 2.980023 -1.287363
C 1.415972 1.841700 -1.307274
C -1.855197 0.376612 1.711133
C -3.003895 1.114964 2.008134
H -3.586696 1.562646 1.205078
C -3.402084 1.280905 3.329583
H -4.296029 1.858663 3.556697
C -2.657883 0.712852 4.359655
H -2.969399 0.848125 5.393748
C -1.514076 -0.022956 4.067903
H -0.928984 -0.465663 4.871701
C -1.113189 -0.188316 2.747209
H -0.215434 -0.755105 2.500562
C -2.244861 -1.374958 -0.566085
C -3.274266 -1.921446 0.200978
H -3.515824 -1.495495 1.171933
C -3.986745 -3.019039 -0.269982
H -4.782581 -3.445682 0.337102
C -3.680413 -3.571740 -1.508155
H -4.238689 -4.431573 -1.873624
C -2.657122 -3.028444 -2.279241
H -2.411829 -3.461455 -3.246794
C -1.938773 -1.935835 -1.811336
H -1.129928 -1.511441 -2.403506
C -1.964065 1.522151 -0.901568
C -1.566634 2.794024 -0.472313
H -0.926657 2.897934 0.402814

C -1.981118 3.923488 -1.164857
H -1.662031 4.907866 -0.828665
C -2.795848 3.795540 -2.286160
H -3.120106 4.682405 -2.827399
C -3.196941 2.534793 -2.713280
H -3.838926 2.431713 -3.585924
C -2.781958 1.398999 -2.025309
H -3.102875 0.415988 -2.363116
C 3.296479 -1.194767 -0.034108
C 5.293448 -0.465238 1.103476
C 4.805624 0.134959 -1.152008
C 5.649670 0.223154 -0.049492
Sn 1.244553 -2.108933 -0.201246
N 3.667970 -0.550804 -1.150655
H 5.929494 -0.432401 1.987930
H 6.562211 0.813830 -0.099256
H 5.060621 0.654229 -2.079315
H 1.636612 0.960568 1.953495
H 3.062248 2.979550 1.995415
H 3.442205 4.283638 -0.087761
H 2.373509 3.539309 -2.207478
H 0.963663 1.504759 -2.238135
C 4.106615 -1.187406 1.106192
H 3.800010 -1.732526 2.000004

^{Ph}YSnPyr
E = -1575.53461591
P -1.295841 0.090842 0.002146
C 0.363394 -0.156425 -0.302858
C 1.204406 1.064932 -0.282793
C 1.861960 1.494686 0.880582
C 2.688604 2.612319 0.872964
C 2.872856 3.341475 -0.297899
C 2.224196 2.935706 -1.460432
C 1.404979 1.814158 -1.451828
C -1.724099 0.398973 1.758631
C -2.835027 1.158882 2.134503
H -3.465225 1.615971 1.373556
C -3.133308 1.335868 3.480695
H -3.997516 1.931045 3.769582
C -2.326497 0.757333 4.456401
H -2.559356 0.902029 5.509759
C -1.220378 -0.000335 4.085910
H -0.585833 -0.450864 4.846591
C -0.918634 -0.176925 2.740547
H -0.049359 -0.759431 2.435323
C -2.279814 -1.350694 -0.491401
C -3.267985 -1.891385 0.332105
H -3.455041 -1.461236 1.313409
C -4.006665 -2.989196 -0.096394
H -4.769456 -3.412235 0.554092
C -3.767747 -3.547476 -1.346985
H -4.346052 -4.408033 -1.677950
C -2.786793 -3.008886 -2.174314
H -2.594918 -3.446025 -3.152016
C -2.042994 -1.915955 -1.749596
H -1.266241 -1.494892 -2.385349
C -1.993764 1.544396 -0.846442
C -1.549438 2.812341 -0.453190
H -0.854986 2.911644 0.379954

C -1.988349 3.943755 -1.127476
H -1.632742 4.925056 -0.820142
C -2.874662 3.821691 -2.193801
H -3.217826 4.710013 -2.720799
C -3.323367 2.564947 -2.583889
H -4.021538 2.466629 -3.412863
C -2.883940 1.427256 -1.914657
H -3.241429 0.447274 -2.223378
C 3.241625 -1.195286 -0.156900
C 4.903980 -0.563082 1.275340
C 4.902312 0.135072 -0.975314
C 5.513988 0.177278 0.272599
Sn 1.203196 -2.128610 -0.333657
N 3.786307 -0.546829 -1.194963
N 3.780871 -1.243230 1.069786
H 5.331771 -0.604021 2.280778
H 6.420783 0.750825 0.450011
H 5.327479 0.676202 -1.824817
H 1.739263 0.915881 1.794586
H 3.196496 2.911928 1.788573
H 3.521690 4.215511 -0.305526
H 2.364293 3.492818 -2.385640
H 0.910262 1.489681 -2.365483

^{Ph}YSnB2
E = -1718.48752229
P -1.697235 0.217833 -0.011987
C -0.105360 -0.367396 -0.250181
C 0.930469 0.698713 -0.248909
C 1.507422 1.174495 0.939473
C 2.475117 2.173978 0.926094
C 2.892253 2.731571 -0.277459
C 2.330679 2.275947 -1.467108
C 1.364910 1.278086 -1.452031
C -2.092148 0.693295 1.714037
C -3.013014 1.698887 2.018602
H -3.497671 2.254933 1.218205
C -3.308529 1.992474 3.345089
H -4.023417 2.779242 3.578313
C -2.689738 1.285045 4.371496
H -2.919662 1.520181 5.409046
C -1.774179 0.281250 4.071862
H -1.286940 -0.271814 4.872468
C -1.474817 -0.011918 2.746802
H -0.751487 -0.787792 2.495549
C -2.948206 -1.012229 -0.467817
C -4.034098 -1.297932 0.360054
H -4.136742 -0.794484 1.318503
C -4.981479 -2.236984 -0.033838
H -5.820849 -2.462530 0.620738
C -4.853690 -2.888249 -1.255088
H -5.595403 -3.624254 -1.559536
C -3.775249 -2.602756 -2.087278
H -3.670035 -3.114366 -3.041709
C -2.822985 -1.671040 -1.696078
H -1.969707 -1.451382 -2.335057
C -2.060206 1.736554 -0.951948
C -1.372101 2.907116 -0.609321
H -0.687385 2.910342 0.237538
C -1.555957 4.065536 -1.351852

H -1.011190 4.968056 -1.082643
C -2.427716 4.069710 -2.436825
H -2.570284 4.979150 -3.017504
C -3.118507 2.912108 -2.776530
H -3.806515 2.913258 -3.619697
C -2.935934 1.746984 -2.038678
H -3.482911 0.846415 -2.308942
H 1.199230 0.731175 1.884682
H 2.915229 2.509196 1.863794
H 3.658937 3.503675 -0.289626
H 2.652983 2.697895 -2.417819
H 0.930617 0.921184 -2.384061
Sn 0.324974 -2.469516 -0.193441
C 4.383100 -0.659975 -0.685095
C 4.346765 -0.761258 0.702169
C 5.368224 0.053148 -1.338956
C 5.293037 -0.153534 1.503338
C 6.325982 0.678911 -0.538180
H 5.377926 0.132169 -2.422710
C 6.289312 0.577883 0.852698
H 5.248081 -0.234269 2.586233
H 7.116133 1.259669 -1.009755
H 7.051832 1.080706 1.444120
B 2.564893 -1.774074 -0.118681
O 3.249303 -1.481031 1.062200
O 3.311254 -1.318746 -1.202964

^{Ph}YSnB1
E = -1987.67528184
P 1.831286 -0.667456 0.059668
C 0.159088 -0.327411 -0.040595
C -0.522561 -0.194398 1.270527
C -0.346716 0.924905 2.101672
H 0.292688 1.736164 1.761861
C -0.976069 1.018554 3.337460
H -0.817958 1.902200 3.955211
C -1.802739 -0.007097 3.785958
H -2.295930 0.064515 4.753613
C -1.994300 -1.122281 2.976522
H -2.639471 -1.934771 3.308315
C -1.361847 -1.215057 1.742308
H -1.516955 -2.090443 1.113780
C 2.893082 0.742190 0.554765
C 4.101737 0.554415 1.229660
H 4.417673 -0.448766 1.510561
C 4.899202 1.648168 1.546641
H 5.838235 1.498233 2.076125
C 4.494485 2.931353 1.190971
H 5.117978 3.787082 1.443824
C 3.291263 3.122054 0.518957
H 2.965770 4.122585 0.244433
C 2.489766 2.031154 0.202334
H 1.535616 2.172195 -0.307664
C 2.493332 -1.228285 -1.532628
C 3.635308 -0.664649 -2.100631
H 4.149876 0.149849 -1.596080
C 4.111372 -1.136617 -3.319890
H 4.997382 -0.686321 -3.762851
C 3.456779 -2.175600 -3.970537
H 3.831747 -2.542385 -4.924218

C 2.319073 -2.744702 -3.405220
H 1.801124 -3.554391 -3.915039
C 1.834468 -2.271060 -2.193946
H 0.937393 -2.700779 -1.751670
C 2.251715 -1.952161 1.288238
C 1.991047 -1.690239 2.639255
H 1.605110 -0.719336 2.943385
C 2.222596 -2.668006 3.597379
H 2.009248 -2.455263 4.642955
C 2.722303 -3.911672 3.222765
H 2.903619 -4.676267 3.975869
C 2.993987 -4.173367 1.885025
H 3.393728 -5.140768 1.587015
C 2.759291 -3.199568 0.919534
H 2.980501 -3.414065 -0.123284
Sn -0.758157 0.140002 -1.927165
N -3.714430 0.308297 -0.104901
C -3.583181 2.303926 0.902098
C -4.260754 1.138288 0.871142
H -3.788285 3.185449 1.498476
H -5.143107 0.863584 1.436707
B -2.582249 0.978583 -0.704510
N -2.573841 2.272845 -0.056141
C -1.601078 3.267257 -0.193606
C -1.147212 3.999255 0.908729
C -1.053015 3.538946 -1.453311
C -0.163687 4.967478 0.752313
H -1.549634 3.778245 1.894544
C -0.058034 4.497253 -1.599504
H -1.441799 3.012797 -2.323251
C 0.389580 5.221880 -0.499351
H 0.180592 5.522783 1.623079
H 0.353017 4.692819 -2.588143
H 1.156236 5.985105 -0.617689
C -4.107154 -1.021942 -0.294327
C -3.931918 -1.631053 -1.541631
C -4.657405 -1.773264 0.748697
C -4.271913 -2.963834 -1.729594
H -3.544300 -1.040860 -2.369431
C -5.011627 -3.101575 0.547350
H -4.771566 -1.321491 1.730757
C -4.815632 -3.709253 -0.688005
H -4.125054 -3.418196 -2.707873
H -5.433139 -3.671189 1.373905
H -5.088429 -4.751429 -0.839278

$^{F}Y_2Sn$
E = -3613.20987155
P -3.111259 -0.785516 -0.060094
P 3.032551 -1.041451 0.086804
C -1.776655 -0.039292 -0.814077
C -1.612328 1.393655 -0.883284
C -2.317631 2.351372 -0.132600
C -2.130194 3.719953 -0.247772
C -1.151958 4.212163 -1.100218
C -0.401478 3.309372 -1.844372
C -0.644413 1.952173 -1.740659
C -3.044416 -0.897672 1.768667
C -3.866443 -0.163756 2.622456
H -4.665475 0.450997 2.215308

C -3.648214 -0.193829 3.996559
H -4.289019 0.389476 4.654749
C -2.611617 -0.952135 4.526530
H -2.440313 -0.966640 5.601105
C -1.783966 -1.683278 3.677858
H -0.963499 -2.271387 4.085815
C -1.997284 -1.649806 2.308326
H -1.335269 -2.210588 1.643782
C -3.208909 -2.520804 -0.619094
C -3.572246 -3.566582 0.229552
H -3.724082 -3.383546 1.290829
C -3.733703 -4.852347 -0.278420
H -4.007060 -5.665205 0.391494
C -3.546079 -5.097351 -1.633045
H -3.670675 -6.104310 -2.026858
C -3.204695 -4.052073 -2.487184
H -3.063167 -4.238253 -3.549824
C -3.041870 -2.769172 -1.985226
H -2.771343 -1.944361 -2.642780
C -4.762696 -0.154420 -0.494750
C -4.888979 0.784910 -1.516997
H -3.996897 1.167105 -2.008679
C -6.150582 1.220222 -1.906852
H -6.245073 1.958970 -2.700146
C -7.286911 0.712325 -1.286821
H -8.273380 1.054826 -1.593486
C -7.164273 -0.240191 -0.279240
H -8.052800 -0.647704 0.198960
C -5.905661 -0.676533 0.114351
H -5.811315 -1.430788 0.895093
C 1.536635 -0.223961 -0.010871
C 1.523190 1.198882 0.292204
C 0.605504 1.743822 1.202130
C 0.532809 3.101685 1.466301
C 1.411622 3.983250 0.847594
C 2.351629 3.487618 -0.044796
C 2.377563 2.128079 -0.316366
C 4.163480 -0.236022 1.268045
C 3.950852 -0.395580 2.639548
H 3.180300 -1.074504 2.996835
C 4.722871 0.308862 3.554105
H 4.552218 0.174823 4.620352
C 5.707584 1.184638 3.107825
H 6.308609 1.740112 3.825175
C 5.919344 1.352103 1.744240
H 6.683146 2.041206 1.389692
C 5.153133 0.644969 0.824597
H 5.314574 0.791033 -0.240209
C 2.703282 -2.735603 0.697807
C 3.328773 -3.873104 0.188695
H 4.049402 -3.787023 -0.619935
C 3.008473 -5.131342 0.689679
H 3.490475 -6.013629 0.273362
C 2.068604 -5.263968 1.704311
H 1.817108 -6.250331 2.089352
C 1.441403 -4.133009 2.219157
H 0.697279 -4.232848 3.007383
C 1.750991 -2.876713 1.716192
H 1.235858 -1.989357 2.083547
C 4.039668 -1.266168 -1.423150

C 5.350682 -1.749705 -1.366670
H 5.807652 -1.975277 -0.403333
C 6.076150 -1.932335 -2.537161
H 7.096451 -2.308170 -2.490155
C 5.498907 -1.627894 -3.767613
H 6.069357 -1.768163 -4.683903
C 4.201527 -1.131842 -3.825456
H 3.756098 -0.875799 -4.784581
C 3.473241 -0.947311 -2.655030
H 2.469574 -0.523444 -2.682418
Sn -0.047384 -1.432470 -0.905216
F 3.263476 1.699126 -1.219944
F 3.190814 4.312696 -0.652414
F 1.335274 5.281583 1.094659
F -0.395098 3.572321 2.288897
F -0.259734 0.949404 1.824515
F 0.129177 1.136612 -2.468129
F 0.579057 3.750941 -2.619915
F -0.911089 5.512418 -1.180619
F -2.846038 4.554193 0.489926
F -3.234417 1.939957 0.750757

^{F}YSnCl
E = -2268.91939508
P -1.131545 0.565904 0.054602
C 0.050453 -0.654955 -0.130549
C 1.458224 -0.244575 -0.073969
C 2.270751 -0.506796 1.033556
C 3.610332 -0.149168 1.075796
C 4.187809 0.479625 -0.019833
C 3.417999 0.746413 -1.144805
C 2.082374 0.372898 -1.163720
C -1.528899 1.032757 1.774338
C -2.204873 2.223792 2.059460
H -2.478959 2.901982 1.252146
C -2.517978 2.544001 3.373647
H -3.044749 3.470034 3.595579
C -2.149369 1.684645 4.406697
H -2.388443 1.941952 5.436854
C -1.467688 0.506320 4.125558
H -1.167328 -0.158206 4.933066
C -1.157425 0.177732 2.809436
H -0.604054 -0.729475 2.574074
C -2.683307 0.000161 -0.692873
C -3.898394 0.016677 -0.007582
H -3.944358 0.388888 1.013272
C -5.049327 -0.460963 -0.625808
H -5.992276 -0.459198 -0.083085
C -4.994300 -0.946380 -1.927412
H -5.896944 -1.321262 -2.405935
C -3.785409 -0.959568 -2.617412
H -3.739978 -1.341717 -3.635031
C -2.630631 -0.493382 -2.002994
H -1.675782 -0.514998 -2.526889
C -0.656488 2.154581 -0.688406
C 0.364026 2.870720 -0.053851
H 0.766788 2.518932 0.895424
C 0.868191 4.026313 -0.634505
H 1.668447 4.574506 -0.141782
C 0.349224 4.480388 -1.843304

H 0.744565 5.386227 -2.298537
C -0.675756 3.779038 -2.468575
H -1.086769 4.137141 -3.410230
C -1.176177 2.613986 -1.898064
H -1.972427 2.065914 -2.396854
Sn -0.544040 -2.702565 -0.267391
Cl 1.778483 -3.500719 -0.260770
F 1.384855 0.623924 -2.266377
F 3.965988 1.346043 -2.191745
F 5.463460 0.829553 0.010259
F 4.343404 -0.405846 2.148272
F 1.759303 -1.113047 2.098769

^{F}YSnHMDS
E = -2682.70057505
P -2.039850 -0.228740 -0.074030
C -0.435341 0.096468 -0.555822
C 0.191269 1.397756 -0.442697
C -0.218783 2.437407 0.403693
C 0.403774 3.673584 0.464347
C 1.499162 3.933777 -0.348179
C 1.936118 2.943534 -1.220527
C 1.292130 1.719291 -1.256366
C -2.273120 -0.587755 1.706999
C -2.863124 0.301710 2.603870
H -3.325654 1.216586 2.242038
C -2.827079 0.037991 3.970033
H -3.279684 0.743051 4.664295
C -2.204571 -1.108355 4.448586
H -2.171475 -1.304648 5.518392
C -1.611555 -1.999994 3.557845
H -1.111261 -2.893998 3.924567
C -1.642127 -1.734874 2.197313
H -1.153484 -2.423441 1.503505
C -2.604304 -1.742103 -0.921919
C -3.442146 -2.673709 -0.306165
H -3.714789 -2.558362 0.740443
C -3.926104 -3.759936 -1.027640
H -4.570217 -4.487430 -0.537923
C -3.586323 -3.917292 -2.366296
H -3.963180 -4.770567 -2.926892
C -2.768480 -2.979982 -2.990329
H -2.506366 -3.095050 -4.039984
C -2.284431 -1.893483 -2.274822
H -1.647179 -1.152996 -2.755483
C -3.289491 1.005122 -0.542975
C -2.946048 2.009982 -1.446892
H -1.927555 2.071407 -1.823260
C -3.908337 2.919706 -1.871047
H -3.635163 3.706595 -2.571036
C -5.214665 2.821717 -1.405470
H -5.966087 3.534891 -1.738734
C -5.565212 1.806211 -0.520218
H -6.590218 1.720100 -0.165272
C -4.607039 0.896781 -0.091448
H -4.885718 0.096221 0.592904
Sn 0.824112 -1.683509 -0.782004
N 2.577280 -0.868477 0.148398
Si 4.055014 -1.114458 -0.753783
Si 2.599165 -0.650017 1.881397

C 3.662960 -1.704375 -2.506989
H 3.208792 -2.704117 -2.527923
H 3.014917 -1.012872 -3.060641
H 4.607931 -1.771906 -3.064132
C 5.050121 0.468779 -0.914256
H 5.417982 0.828859 0.052735
H 5.920615 0.309104 -1.564860
H 4.436795 1.260461 -1.360145
C 5.130522 -2.474522 -0.009113
H 6.047525 -2.617776 -0.596277
H 5.427623 -2.267830 1.025816
H 4.580503 -3.425385 -0.009786
C 4.215093 0.095236 2.499398
H 4.192613 0.123320 3.597228
H 5.112364 -0.457146 2.199740
H 4.319221 1.128608 2.145217
C 2.353481 -2.330246 2.712499
H 2.333884 -2.253434 3.807859
H 1.404029 -2.787263 2.396469
H 3.155780 -3.025125 2.431512
C 1.269743 0.498015 2.547628
H 0.262125 0.284661 2.178647
H 1.249328 0.406963 3.642568
H 1.507852 1.540873 2.306818
F 1.733043 0.821442 -2.142025
F -1.248146 2.229227 1.231403
F -0.032391 4.600939 1.304065
F 2.109862 5.107643 -0.302242
F 2.962553 3.183806 -2.024688

$^{F}YSnC_6F_5$
E = -2537.24629212
P -2.168401 0.122425 0.137789
C -0.538978 -0.329214 -0.135002
C 0.490247 0.688703 0.105257
C 1.360309 0.640963 1.198906
C 2.435137 1.507673 1.334149
C 2.643929 2.497598 0.381479
C 1.789223 2.591504 -0.710717
C 0.733053 1.700356 -0.831109
C -2.793514 -0.199230 1.821033
C -3.995469 0.368386 2.257501
H -4.561416 1.022636 1.594871
C -4.459684 0.104861 3.538895
H -5.395558 0.544478 3.878162
C -3.721870 -0.713806 4.391895
H -4.084148 -0.913303 5.398621
C -2.519587 -1.264986 3.964809
H -1.935693 -1.891383 4.636020
C -2.052835 -1.009005 2.678972
H -1.100135 -1.411743 2.339041
C -3.252849 -0.768568 -1.008778
C -4.382001 -1.475266 -0.596371
H -4.647834 -1.515718 0.457368
C -5.159232 -2.146130 -1.535447
H -6.031986 -2.708114 -1.209614
C -4.818154 -2.106230 -2.882349
H -5.428452 -2.633627 -3.612911
C -3.693143 -1.398743 -3.298210
H -3.422969 -1.369753 -4.351716

C -2.906396 -0.736473 -2.365842
H -2.013411 -0.193760 -2.675328
C -2.453506 1.905425 -0.064642
C -1.903779 2.754495 0.901827
H -1.400684 2.334394 1.772107
C -1.996908 4.131478 0.752192
H -1.561565 4.787538 1.503035
C -2.645998 4.668107 -0.355864
H -2.718498 5.747520 -0.473018
C -3.203653 3.826402 -1.311667
H -3.715738 4.244984 -2.175542
C -3.105326 2.446488 -1.172012
H -3.538136 1.794471 -1.927109
C 2.135479 -1.775800 -0.613588
C 4.137444 -1.631248 0.757889
C 3.931479 -0.248135 -1.206476
C 4.658189 -0.665505 -0.095894
Sn -0.072718 -2.314723 -0.787036
C 2.692275 -0.824275 -1.446895
C 2.886359 -2.169223 0.476284
F 2.387308 -3.058443 1.342200
F 1.990368 -0.370705 -2.495014
F 4.411832 0.711084 -1.985228
F 5.840490 -0.130849 0.154840
F 4.831508 -2.013631 1.818457
F -0.036587 1.791670 -1.909261
F 1.991836 3.525367 -1.625636
F 3.661236 3.332082 0.504210
F 3.270976 1.386662 2.354168

^{F}YSnPy
E = -2056.38309255
P -1.685734 0.194083 0.072856
C -0.103213 -0.432838 -0.029140
C 1.025213 0.496908 0.058464
C 1.900807 0.506412 1.149133
C 3.087833 1.222852 1.147305
C 3.404011 2.022656 0.056817
C 2.544965 2.066768 -1.033945
C 1.383690 1.309754 -1.022922
C -2.415698 0.208268 1.749082
C -3.573681 0.943361 2.022889
H -4.046212 1.528041 1.233993
C -4.113932 0.934646 3.301789
H -5.016104 1.505142 3.513964
C -3.495131 0.203304 4.313772
H -3.916527 0.202845 5.317317
C -2.334925 -0.514143 4.047144
H -1.843080 -1.072504 4.841159
C -1.793125 -0.512111 2.765234
H -0.870078 -1.045856 2.543075
C -2.818139 -0.760821 -0.973873
C -4.032305 -1.273202 -0.518790
H -4.339148 -1.119271 0.513014
C -4.844583 -2.000640 -1.383218
H -5.785257 -2.410728 -1.021505
C -4.452765 -2.209989 -2.700104
H -5.090465 -2.780598 -3.372591
C -3.241729 -1.697598 -3.158671
H -2.931091 -1.865024 -4.187894

C -2.421157 -0.981035 -2.298557
H -1.462184 -0.590497 -2.638217
C -1.803768 1.941971 -0.415353
C -1.184548 2.881371 0.415790
H -0.722813 2.560410 1.348898
C -1.156117 4.220955 0.052407
H -0.667041 4.946343 0.699307
C -1.752305 4.632170 -1.136097
H -1.729989 5.682354 -1.420471
C -2.377725 3.702260 -1.958889
H -2.848069 4.022931 -2.886184
C -2.400831 2.357789 -1.604608
H -2.885400 1.634686 -2.256574
C 2.460953 -2.178389 -0.169339
C 4.625898 -2.029042 0.878611
C 4.186896 -1.150372 -1.295588
C 5.080119 -1.353451 -0.248022
F 0.620820 1.330721 -2.111313
F 2.855672 2.813661 -2.084555
F 4.529829 2.720671 0.047730
F 3.920729 1.148828 2.175615
F 1.636833 -0.250875 2.212478
Sn 0.239615 -2.539076 -0.303473
N 2.917930 -1.545231 -1.261088
H 5.291545 -2.210337 1.721711
H 6.102210 -0.986948 -0.316311
H 4.511493 -0.630825 -2.200725
C 3.304318 -2.457774 0.910333
H 2.918871 -2.978295 1.786900

^{F}YSnPyr
E = -2072.44424700
P -1.668883 0.215815 0.090886
C -0.105741 -0.440655 -0.092786
C 1.036637 0.474942 -0.015232
C 1.901087 0.509386 1.084671
C 3.081414 1.238223 1.081155
C 3.405596 2.014934 -0.023586
C 2.557048 2.032858 -1.123656
C 1.397906 1.272357 -1.106899
C -2.319263 0.244652 1.797782
C -3.441260 1.011104 2.129806
H -3.933837 1.614001 1.367307
C -3.919426 1.010193 3.433219
H -4.793266 1.605284 3.691439
C -3.274230 0.254888 4.410605
H -3.646768 0.260606 5.433292
C -2.149960 -0.494751 4.085136
H -1.637280 -1.072658 4.851433
C -1.669854 -0.500876 2.778894
H -0.775022 -1.061153 2.511890
C -2.863522 -0.718092 -0.904113
C -4.068970 -1.200544 -0.396216
H -4.330431 -1.031658 0.645752
C -4.930333 -1.917742 -1.220758
H -5.863955 -2.304784 -0.817770
C -4.596355 -2.146737 -2.550406
H -5.272413 -2.709596 -3.191185
C -3.394821 -1.663656 -3.062050
H -3.129828 -1.845786 -4.101493

C -2.525649 -0.957328 -2.241988
H -1.573658 -0.588815 -2.623362
C -1.761856 1.964605 -0.399590
C -1.114257 2.895176 0.419936
H -0.653716 2.569920 1.352193
C -1.056360 4.231120 0.046460
H -0.545442 4.949203 0.684576
C -1.651159 4.647846 -1.140784
H -1.605933 5.695107 -1.433097
C -2.304637 3.727173 -1.952168
H -2.774217 4.052322 -2.878276
C -2.357333 2.386350 -1.587670
H -2.864577 1.670776 -2.230595
C 2.411892 -2.199316 -0.287217
C 4.241226 -2.245316 1.079047
C 4.332613 -1.229930 -1.046749
C 4.983355 -1.537550 0.142918
F 0.633789 1.281701 -2.193787
F 2.872036 2.764217 -2.183278
F 4.527226 2.719917 -0.035491
F 3.905202 1.190278 2.118823
F 1.627059 -0.217025 2.163379
Sn 0.189604 -2.544809 -0.424161
N 3.061923 -1.548293 -1.262619
N 2.974425 -2.586454 0.864988
H 4.681426 -2.545822 2.033013
H 6.014575 -1.245838 0.326715
H 4.852422 -0.696346 -1.847063

^{F}YSnB2
E = -2215.39676361
P -1.972875 0.323784 -0.006786
C -0.522788 -0.590203 0.020630
C 0.701105 0.226101 -0.046924
C 1.362425 0.701501 1.087765
C 2.514638 1.472337 1.011694
C 3.027367 1.817824 -0.230407
C 2.383847 1.387079 -1.384517
C 1.239149 0.612955 -1.280430
C -2.734488 0.692243 1.609980
C -3.713908 1.683753 1.732571
H -4.028199 2.250506 0.856425
C -4.275459 1.953356 2.973388
H -5.037006 2.724943 3.068343
C -3.857097 1.241619 4.095870
H -4.294754 1.457843 5.068656
C -2.873622 0.266625 3.977914
H -2.536087 -0.278650 4.856908
C -2.308284 -0.006684 2.736331
H -1.515421 -0.745543 2.630929
C -3.246565 -0.494949 -1.008051
C -4.579670 -0.601528 -0.611536
H -4.900245 -0.202606 0.347664
C -5.499098 -1.241562 -1.435999
H -6.535194 -1.334205 -1.116900
C -5.095149 -1.769721 -2.656856
H -5.817618 -2.271734 -3.297547
C -3.765648 -1.666075 -3.055884
H -3.445406 -2.085023 -4.007617
C -2.840447 -1.038397 -2.232697

H -1.792047 -0.970179 -2.521214
C -1.623699 1.965654 -0.701946
C -1.015680 2.907367 0.136136
H -0.935770 2.711008 1.204686
C -0.490379 4.076882 -0.396531
H -0.006974 4.799576 0.257573
C -0.575466 4.316575 -1.765179
H -0.157520 5.229884 -2.183927
C -1.194472 3.390808 -2.597923
H -1.264810 3.579630 -3.667036
C -1.713456 2.213378 -2.071094
H -2.174125 1.481716 -2.730822
Sn -0.558227 -2.734773 0.174385
C 3.691672 -1.639594 -0.476042
C 3.668124 -1.586204 0.913730
C 4.721602 -1.086428 -1.212228
C 4.670895 -0.972512 1.638906
C 5.735975 -0.454830 -0.489989
H 4.716370 -1.116828 -2.298532
C 5.709126 -0.396343 0.904227
H 4.626398 -0.914234 2.722916
H 6.557229 0.014648 -1.026874
H 6.510846 0.116505 1.430915
B 1.771292 -2.476235 0.219762
O 2.505571 -2.142556 1.356246
O 2.545011 -2.231231 -0.913445
F 0.644481 0.226461 -2.405435
F 0.887818 0.408557 2.294227
F 3.137010 1.864015 2.114390
F 2.886224 1.698780 -2.570649
F 4.141180 2.526623 -0.317273

^{F}*YSnB1*
E = -2484.58534594
P 2.105885 0.685751 0.223883
C 0.482353 0.279366 -0.100063
C -0.259127 -0.519579 0.870856
C -1.358718 0.002996 1.565151
C -2.193707 -0.781052 2.343986
C -1.907251 -2.130127 2.513758
C -0.796931 -2.675478 1.884339
C 0.004536 -1.877065 1.082386
C 2.379125 2.396115 0.809990
C 3.628814 2.799819 1.292040
H 4.458227 2.093050 1.314923
C 3.808836 4.097183 1.752459
H 4.782150 4.410701 2.125051
C 2.740975 4.992547 1.746176
H 2.883010 6.007842 2.111854
C 1.493225 4.586704 1.288392
H 0.653764 5.278996 1.299674
C 1.309600 3.287985 0.822779
H 0.329987 2.944746 0.492179
C 3.135298 0.459057 -1.254377
C 4.023575 1.415165 -1.742961
H 4.131108 2.372887 -1.239782
C 4.756053 1.153297 -2.897028
H 5.437003 1.907735 -3.285741
C 4.611650 -0.061638 -3.556335
H 5.186338 -0.261799 -4.458707

C 3.724638 -1.018951 -3.069276
H 3.605805 -1.969306 -3.586334
C 2.978812 -0.758983 -1.928111
H 2.258579 -1.486254 -1.553496
C 2.827017 -0.309319 1.562823
C 2.306346 -0.119953 2.847127
H 1.549688 0.645235 3.017882
C 2.750761 -0.906161 3.901232
H 2.338398 -0.758907 4.897303
C 3.721197 -1.879713 3.681518
H 4.069596 -2.495992 4.508062
C 4.245898 -2.064648 2.407476
H 5.005959 -2.823940 2.235048
C 3.798384 -1.285342 1.346102
H 4.204117 -1.441498 0.349161
Sn -0.424421 0.968506 -1.935783
N -2.531434 -1.643634 -1.284919
C -4.366650 -0.775409 -0.344570
C -3.735461 -1.929783 -0.645760
H -5.342386 -0.638119 0.106990
H -4.091795 -2.943002 -0.503568
B -2.380581 -0.203702 -1.371439
N -3.598534 0.298945 -0.783803
C -3.942589 1.639705 -0.543316
C -4.567068 2.017267 0.647507
C -3.640071 2.616876 -1.494883
C -4.878665 3.350529 0.878097
H -4.760796 1.267066 1.410360
C -3.937534 3.951427 -1.247583
H -3.189381 2.316880 -2.438846
C -4.562156 4.325702 -0.062674
H -5.357985 3.631693 1.814078
H -3.694158 4.701001 -1.998429
H -4.802792 5.369856 0.125883
C -1.584776 -2.616761 -1.631747
C -0.666865 -2.365428 -2.657273
C -1.508337 -3.837294 -0.953322
C 0.332851 -3.282339 -2.954536
H -0.766250 -1.460516 -3.254826
C -0.506902 -4.749696 -1.255526
H -2.204040 -4.052684 -0.146522
C 0.426796 -4.476908 -2.249594
H 1.033195 -3.061684 -3.758573
H -0.448900 -5.677308 -0.689778
H 1.212218 -5.193623 -2.479014
F -1.652527 1.294594 1.442329
F -3.265722 -0.256550 2.926978
F -2.704452 -2.897347 3.243418
F -0.549949 -3.974649 2.000433
F 1.008338 -2.462045 0.433818

I
E = -1021.50458148
C -1.573170 2.503066 0.091050
C 0.544081 1.314450 0.147114
C 1.190967 2.553772 0.296699
C 0.473396 3.737509 0.324677
C -0.916999 3.716797 0.210456
H -2.660529 2.509061 0.019293
H 2.272028 2.577169 0.405205

H 1.000380 4.682633 0.442554
H -1.486359 4.644205 0.225121
N 1.194069 0.100676 0.129289
C 2.588679 -0.005139 0.032741
C 3.316040 0.664772 -0.962673
C 3.283599 -0.857301 0.899157
C 4.687414 0.489382 -1.076937
H 2.783579 1.316035 -1.652812
C 4.655505 -1.041148 0.770898
H 2.727748 -1.369224 1.683125
C 5.367729 -0.365564 -0.213076
H 5.230473 1.017420 -1.859298
H 5.172258 -1.711035 1.456216
H 6.442833 -0.503260 -0.308228
C -0.885205 1.273940 0.067288
C -1.487273 -0.039307 -0.013390
P -3.172681 -0.279700 -0.098572
C -4.131979 0.329966 1.326553
H -3.946498 1.399830 1.462401
H -5.205875 0.156550 1.192366
H -3.779091 -0.192669 2.221796
C -3.999498 0.432947 -1.558362
H -5.079959 0.249789 -1.537480
H -3.810470 1.510057 -1.598057
H -3.561423 -0.022660 -2.452588
C -3.565209 -2.046554 -0.180109
H -4.649685 -2.188619 -0.234226
H -3.096666 -2.485825 -1.067109
H -3.177716 -2.549714 0.712084
Sn -0.064635 -1.630370 0.009814

II
E = -1570.70479972
C -1.446694 2.755422 -0.675121
C -0.748009 1.552188 -0.712410
C 0.515411 1.384460 -0.081051
C 0.997393 2.521058 0.608846
C 0.294300 3.712518 0.648584
C -0.934041 3.851522 0.002191
H -2.400257 2.801784 -1.197860
H 1.944895 2.442128 1.135470
H 0.714168 4.551272 1.202049
H -1.474978 4.794363 0.021473
N 1.186934 0.189614 -0.077196
S -1.595209 0.148276 -1.380712
O -2.838625 0.616992 -1.985944
O -0.596826 -0.538467 -2.306948
C -1.697764 -0.983212 -0.140697
C 2.557298 0.154010 0.210881
C 3.475928 0.982083 -0.454980
C 3.059112 -0.781793 1.124692
C 4.835837 0.872662 -0.210227
H 3.101371 1.705088 -1.177084
C 4.426432 -0.901183 1.352902
H 2.358211 -1.412805 1.670915
C 5.323043 -0.072110 0.691832
H 5.528262 1.522956 -0.742450
H 4.788254 -1.640737 2.065219
H 6.391923 -0.158674 0.874833
P -2.543125 -0.561420 1.270800

C -1.685681 0.534463 2.447850
H -2.234977 0.619032 3.392919
H -0.683396 0.131514 2.633595
H -1.571195 1.528395 1.999115
C -2.913838 -2.062673 2.208677
H -1.973727 -2.571345 2.448950
H -3.443906 -1.825583 3.137325
H -3.520618 -2.725430 1.584216
C -4.107636 0.272476 0.908597
H -3.879954 1.249511 0.467997
H -4.655781 -0.312800 0.164294
H -4.701904 0.404721 1.819371
Sn 0.337047 -1.795227 -0.623416

III
E = -2332.77588920
C -0.536913 1.751137 -0.807932
C 0.218395 1.917818 0.359715
C 0.534599 3.211167 0.775645
C 0.137811 4.320980 0.040845
C -0.583814 4.149692 -1.132992
C -0.921484 2.868610 -1.548321
H 1.120911 3.321027 1.684506
H 0.398361 5.318847 0.388632
H -0.891903 5.009298 -1.724933
H -1.511880 2.705144 -2.445938
S -1.160066 0.167617 -1.455190
S 0.878257 0.609073 1.468846
O -2.257823 0.582802 -2.341095
O 0.019866 -0.515843 -2.111553
O -0.247539 0.209524 2.329045
O 1.998361 1.319263 2.132135
C -1.564373 -0.851556 -0.220975
C 1.489221 -0.657306 0.553584
P 3.047086 -0.337180 -0.076402
P -2.936087 -0.550290 0.731775
C 3.418862 -1.576117 -1.342470
H 4.425287 -1.414722 -1.743459
H 3.356898 -2.582759 -0.914596
H 2.681837 -1.476834 -2.148995
C 3.199729 1.252715 -0.936319
H 3.040483 2.054198 -0.207764
H 4.185797 1.359080 -1.402413
H 2.409241 1.291593 -1.695312
C 4.406232 -0.389886 1.120179
H 4.446774 -1.392173 1.559487
H 5.367524 -0.141625 0.655830
H 4.153873 0.330938 1.904467
C -2.861124 -1.600825 2.193023
H -3.745920 -1.458931 2.822282
H -2.798921 -2.648280 1.878248
H -1.951276 -1.316215 2.733273
C -3.103800 1.158752 1.307192
H -3.155671 1.816669 0.431859
H -4.011507 1.277177 1.909597
H -2.211067 1.395716 1.895261
C -4.483039 -0.898810 -0.149358
H -5.370127 -0.618604 0.430206
H -4.438433 -0.327838 -1.084524
H -4.516524 -1.964110 -0.400392

Sn 0.193109 -2.207136 -0.318271

IV
E = -1540.75684820
C -0.924384 3.204579 -0.135643
C -0.291340 1.987854 -0.319570
C 0.931748 1.608066 0.247058
C 1.538107 2.557789 1.078400
C 0.935089 3.794121 1.285535
C -0.283267 4.127184 0.684601
H -1.871096 3.430523 -0.623551
H 2.498450 2.339651 1.543591
H 1.429407 4.528910 1.919368
H -0.724270 5.107481 0.852070
B 1.320918 0.139252 -0.221432
S -0.923304 0.578590 -1.166932
O -1.786967 0.892810 -2.310291
O 0.472361 -0.024401 -1.516348
C -1.580651 -0.494660 -0.037127
C 2.806252 -0.330901 -0.419099
C 3.277186 -0.776752 -1.661450
C 3.723852 -0.309707 0.642693
C 4.598599 -1.175061 -1.837100
H 2.588217 -0.805417 -2.504634
C 5.046813 -0.698001 0.473158
H 3.388771 0.003591 1.633199
C 5.492119 -1.135002 -0.771779
H 4.935174 -1.516478 -2.815412
H 5.734222 -0.667841 1.317678
H 6.526549 -1.446002 -0.907876
P -3.259878 -0.777396 -0.187672
C -4.278246 0.724576 -0.190531
H -5.345412 0.483449 -0.251224
H -4.074134 1.294209 0.722316
H -3.984620 1.320695 -1.061378
C -3.813989 -1.779922 1.206173
H -3.632842 -1.232472 2.136677
H -4.880497 -2.009909 1.107077
H -3.230606 -2.706097 1.233257
C -3.730127 -1.665698 -1.695733
H -3.364628 -1.071338 -2.540288
H -3.225713 -2.637576 -1.701911
H -4.814943 -1.803952 -1.767593
Sn -0.021106 -1.313645 1.272348

V
E = -991.519698916
C -1.613869 2.500597 0.149186
C 0.532996 1.381816 0.252317
C 1.128740 2.631850 0.466375
C 0.389402 3.808171 0.525821
C -0.987251 3.726099 0.351396
H -2.696314 2.502189 0.034712
H 2.208435 2.675035 0.606402
H 0.872555 4.766588 0.702754
H -1.593934 4.630921 0.382794
B 1.335894 0.068873 0.195637
C 2.864789 -0.035808 -0.029212
C 3.567285 0.862565 -0.856011
C 3.607021 -1.073386 0.564586

C 4.934104 0.736003 -1.068862
H 3.020782 1.662278 -1.353934
C 4.980254 -1.179823 0.390838
H 3.085305 -1.808027 1.178061
C 5.646976 -0.277099 -0.433566
H 5.450008 1.434173 -1.726261
H 5.532133 -1.980639 0.880258
H 6.720414 -0.370573 -0.590342
C -0.892379 1.291225 0.113888
C -1.498597 -0.033549 -0.030126
P -3.181568 -0.219652 -0.270763
C -4.275120 0.358535 1.077108
H -4.090765 1.415826 1.288347
H -5.332332 0.215081 0.825184
H -4.029351 -0.215857 1.976745
C -3.850537 0.573873 -1.773177
H -4.934451 0.431779 -1.852352
H -3.621469 1.643655 -1.766953
H -3.354094 0.124491 -2.639729
C -3.647122 -1.958367 -0.460594
H -4.730240 -2.031299 -0.609747
H -3.122520 -2.389132 -1.318888
H -3.355697 -2.518103 0.433777
Sn -0.206091 -1.742284 0.223853

6.2.2 Triplet state structures

$^{Tos}Y_2Sn$

E = -3796.52462504
C 4.934491 2.230854 0.459432
H 4.826384 2.229248 1.541237
C 3.839816 1.902335 -0.325146
C -3.956083 -1.839844 -0.397649
O 1.324352 2.513528 0.074510
C -4.081239 -1.755727 -1.780357
H -3.209650 -1.508618 -2.384741
C 2.593615 -3.256580 2.426008
H 3.598445 -3.529968 2.106978
C 6.145765 2.528112 -0.154987
H 7.009723 2.777422 0.460196
C -1.368471 4.783706 -1.745570
H -0.853502 5.717159 -1.529169
C -1.604075 3.877921 -0.716088
H -1.252115 4.094350 0.288903
C 2.702941 -2.248011 -2.385325
H 3.079748 -1.239333 -2.552803
C 2.358062 -2.649204 -1.088054
C 5.291838 -1.366169 -0.399420
H 4.996858 -1.648412 -1.406254
C 6.631129 -1.119945 -0.124431
H 7.368057 -1.204268 -0.920681
C -1.768305 4.491012 -3.042813
H -1.580564 5.203284 -3.844119
C -1.805240 -0.035531 -0.264624
Sn 0.000092 -0.032803 -1.420037
O -2.657566 -1.367336 1.810204
S -2.370359 -1.518373 0.365930
P -2.509495 1.424663 0.300193
C -5.320483 -1.969583 -2.364594
H -5.423806 -1.897785 -3.446838
C -4.719539 0.901430 1.904247

H -3.983132 0.726892 2.684319
C -0.618577 1.638797 2.307272
H -0.107409 0.843644 1.774808
C 6.078761 -0.662342 2.174065
H 6.382159 -0.382096 3.180838
C 4.736595 -0.898638 1.903877
H 3.991850 -0.780327 2.686519
C -2.613380 2.361486 -2.306073
H -3.051050 1.388433 -2.526934
C 1.886586 -2.256034 1.749372
C 3.936953 1.889761 -1.712911
H 3.061359 1.639025 -2.310201
C 1.808588 -3.912189 -0.876529
H 1.491558 -4.210759 0.119183
C 4.336797 -1.263437 0.614686
C -1.924227 3.705377 3.638760
H -2.434134 4.512389 4.161531
C -1.835991 2.112429 1.832984
C -2.492856 3.152616 2.500239
H -3.447690 3.524467 2.130686
C -2.238807 2.668337 -0.991407
C -2.391659 3.276760 -3.323751
H -2.687541 3.035805 -4.342843
C 7.604254 2.757765 -2.195527
H 7.484097 3.217570 -3.182819
H 8.160478 1.820416 -2.339050
H 8.230578 3.416485 -1.584111
C -0.700108 3.229612 4.107723
H -0.254012 3.671744 4.996946
C -6.442847 -2.276454 -1.586920
C 7.027064 -0.773210 1.163227
H 8.077401 -0.584419 1.377590
C -6.595006 1.268002 -0.119883
H -7.324658 1.408584 -0.914938
C 1.786183 -0.011534 -0.231995
P 2.557929 -1.460175 0.267782
O 2.607177 1.279381 1.882175
S 2.286577 1.470384 0.450810
O -1.470727 -2.595121 -0.064078
C 6.279897 2.496224 -1.543162
C 5.153008 2.180879 -2.311550
H 5.234101 2.166002 -3.397994
C 2.531220 -3.118399 -3.451015
H 2.802953 -2.803493 -4.456570
C 1.988580 -4.383515 -3.234940
H 1.838811 -5.060843 -4.073611
C 1.622577 -4.773588 -1.953275
H 1.174150 -5.750511 -1.786110
C -5.245484 1.466443 -0.383483
H -4.935976 1.769194 -1.379810
C -0.045595 2.201293 3.442252
H 0.925143 1.840513 3.771416
C -4.299373 1.290965 0.628750
C -7.010382 0.897047 1.154825
H -8.068585 0.746015 1.360300
C -6.281493 -2.380815 -0.204928
H -7.142253 -2.623538 0.417348
C -6.071441 0.713603 2.163684
H -6.389935 0.414004 3.160191
C 0.718819 -3.551282 3.912741

H 0.262461 -4.063014 4.758358
C 2.008460 -3.896564 3.509968
H 2.557996 -4.671382 4.041289
C -5.046939 -2.162505 0.395012
H -4.916387 -2.215165 1.473145
C -7.791350 -2.449799 -2.218757
H -8.413746 -3.151561 -1.652654
H -7.711065 -2.815500 -3.248354
H -8.331040 -1.492558 -2.253872
C 0.013675 -2.563618 3.236725
H -1.003925 -2.297941 3.511576
C 0.602861 -1.912831 2.159305
H 0.053872 -1.146643 1.623119

^{Tos}*YSnCl*
E = -2360.52303224
C 3.200800 -0.475580 -1.636355
H 2.978208 0.150979 -2.496485
C 2.194415 -1.265354 -1.096606
O 0.220595 -2.583468 -2.179228
C -1.505228 3.355049 -0.158645
H -0.652477 3.743772 0.396467
C 4.458419 -0.495243 -1.051520
H 5.249692 0.127019 -1.467890
C 0.183244 -0.387630 2.606361
H 0.812117 -1.048939 2.011820
C -0.536421 0.639872 1.981469
C 2.241578 1.703275 0.903176
H 2.084993 1.196839 1.852276
C 3.451512 2.339731 0.654309
H 4.233900 2.322352 1.410320
Sn -2.080281 -2.148373 0.338002
C 2.656401 3.014523 -1.519499
H 2.818544 3.524142 -2.467017
C 1.444792 2.379695 -1.281272
H 0.669332 2.372717 -2.043735
C -1.611186 1.984366 -0.423236
C 2.439449 -2.098305 -0.009834
H 1.647222 -2.745868 0.365790
C -1.343732 1.486876 2.742284
H -1.912124 2.279712 2.261666
C 1.232996 1.725777 -0.062758
C 6.075557 -1.256509 0.717497
H 6.176964 -2.039423 1.475761
H 6.254125 -0.290173 1.208693
H 6.873767 -1.391918 -0.022070
C 3.658663 2.996732 -0.553648
H 4.605757 3.497959 -0.744550
C -0.523339 -0.719208 -0.530981
P -0.344677 0.860945 0.196710
O 0.536891 -0.126718 -2.779153
S 0.559632 -1.221998 -1.795515
C 4.723623 -1.284934 0.071303
C 3.698020 -2.089922 0.576429
H 3.895769 -2.731196 1.434235
C 0.077522 -0.571967 3.976550
H 0.630066 -1.376993 4.456336
C -0.742178 0.263718 4.732257
H -0.831322 0.111597 5.805902
C -1.447234 1.290686 4.114436

H -2.091367 1.941163 4.702327
C -3.565669 3.722322 -1.349120
H -4.332623 4.403482 -1.713033
C -2.487942 4.220012 -0.619866
H -2.409741 5.285863 -0.415633
C -3.660618 2.361855 -1.618701
H -4.497699 1.967511 -2.190413
C -2.685223 1.483848 -1.158729
H -2.771584 0.419421 -1.371758
Cl -3.825332 -1.877893 -1.408014

^{Tos}YSnHMDS
E = -2774.31845384
S 1.027575 -1.613207 0.811421
P 0.865817 1.203407 0.095807
Si -4.172586 0.495081 -0.383974
Si -3.508148 -2.381378 0.606689
O 0.261664 -2.851220 0.640447
O 1.173427 -1.049188 2.168737
N -3.125450 -0.909769 -0.295135
C 0.391168 -0.411251 -0.232836
C 2.691070 -1.958285 0.255782
C 2.920810 -2.244586 -1.086759
H 2.084608 -2.264449 -1.783816
C 4.215496 -2.483188 -1.517587
H 4.399668 -2.701520 -2.569047
C 5.292803 -2.451819 -0.622792
C 5.029318 -2.185685 0.720085
H 5.853935 -2.160795 1.431455
C 3.735332 -1.936588 1.166241
H 3.523463 -1.706086 2.207490
C 6.692455 -2.680851 -1.108818
H 6.755164 -3.573203 -1.743270
H 7.390988 -2.806333 -0.275137
H 7.043529 -1.832640 -1.712417
C 2.632798 1.366712 0.515071
C 3.595485 1.363623 -0.497507
H 3.293772 1.343898 -1.541944
C 4.945795 1.391587 -0.174940
H 5.689614 1.390408 -0.969015
C 5.343444 1.422821 1.158245
H 6.402171 1.448134 1.409399
C 4.388474 1.409438 2.168317
H 4.695768 1.416877 3.212142
C 3.035545 1.372049 1.852575
H 2.291099 1.319715 2.642446
C 0.004151 1.983454 1.489871
C 0.397588 3.235203 1.974791
H 1.216357 3.773818 1.498631
C -0.236193 3.779662 3.083469
H 0.069014 4.754277 3.458976
C -1.247727 3.067367 3.723459
H -1.734596 3.487553 4.601479
C -1.626394 1.815548 3.252355
H -2.400853 1.248376 3.764663
C -1.006974 1.270999 2.132414
H -1.291637 0.288495 1.760448
C 0.577165 2.195584 -1.397992
C 0.922666 1.641921 -2.637436
H 1.321048 0.629151 -2.677121

C 0.740776 2.371415 -3.803682
H 1.011707 1.933528 -4.762100
C 0.195635 3.651881 -3.747131
H 0.041097 4.218694 -4.663168
C -0.158932 4.200620 -2.521547
H -0.598389 5.194697 -2.473698
C 0.035190 3.479028 -1.347491
H -0.269360 3.910055 -0.397783
C -4.881533 0.877954 1.319102
H -5.591063 1.712927 1.244481
H -4.075765 1.184419 1.995951
H -5.405228 0.033480 1.779026
C -3.216016 2.029239 -0.897848
H -3.887291 2.899545 -0.870692
H -2.789108 1.962146 -1.906601
H -2.392796 2.222496 -0.198320
C -5.571879 0.229587 -1.608941
H -6.206646 -0.615306 -1.316414
H -5.166303 0.012667 -2.605910
H -6.208225 1.120942 -1.688803
C -2.853290 -2.152688 2.351354
H -3.256757 -1.236519 2.803008
H -1.758290 -2.074782 2.320248
H -3.111695 -2.998005 3.002342
C -2.737256 -3.894839 -0.179415
H -3.069052 -4.789213 0.366690
H -1.643133 -3.857501 -0.137411
H -3.055369 -4.001736 -1.224915
C -5.367265 -2.696373 0.624703
H -5.721843 -2.905708 -0.393032
H -5.967148 -1.872421 1.026100
H -5.576016 -3.584835 1.235857
Sn -1.333708 -0.902849 -1.404525

$^{Tos}YSnC_6F_5$
E = -2628.85898412
C 3.936047 -1.273018 -1.798297
H 3.679869 -0.780946 -2.733518
C 2.916838 -1.679266 -0.949060
O 0.605812 -2.693365 -1.669219
C 0.040466 3.502958 -0.894676
H 0.810161 3.937016 -0.257075
C 5.256485 -1.478209 -1.419060
H 6.058280 -1.151151 -2.080105
C 1.029171 0.605574 2.879351
H 1.587823 -0.282092 2.585947
C 0.492581 1.443734 1.893896
C 3.548708 1.297731 0.754171
H 3.305685 0.921027 1.743781
C 4.870556 1.586571 0.442707
H 5.646056 1.427545 1.189203
C 4.204243 2.269068 -1.768338
H 4.457721 2.641807 -2.758659
C 2.880303 1.974454 -1.465972
H 2.108384 2.089861 -2.221838
C -0.165849 2.120533 -0.906477
C 3.203814 -2.307371 0.257347
H 2.391784 -2.630418 0.907077
C -0.271945 2.546794 2.269558
H -0.742838 3.168857 1.513428

C 2.543824 1.499065 -0.195616
C 7.003557 -2.240651 0.224650
H 7.113643 -3.024874 0.981081
H 7.388783 -1.307636 0.660022
H 7.649130 -2.490051 -0.624936
C 5.199358 2.078651 -0.816666
H 6.235603 2.308075 -1.057862
C 0.457434 -0.644563 -0.064338
P 0.816916 1.031983 0.158254
O 1.274011 -0.391309 -2.507209
S 1.215833 -1.391131 -1.421995
C 5.574929 -2.079259 -0.199755
C 4.527338 -2.497649 0.627037
H 4.755858 -2.983123 1.575036
C 0.835331 0.895307 4.222648
H 1.258328 0.241832 4.982634
C 0.083457 2.006698 4.592719
H -0.077789 2.227870 5.645985
C -0.475715 2.823377 3.617602
H -1.084187 3.678678 3.903459
C -1.698093 3.764023 -2.539520
H -2.303124 4.407672 -3.175129
C -0.731629 4.322826 -1.707208
H -0.576226 5.399667 -1.694958
C -1.883545 2.386240 -2.570352
H -2.626272 1.948736 -3.234060
C -1.114141 1.560734 -1.759606
H -1.233214 0.479461 -1.785868
Sn -0.994966 -1.760524 1.033693
C -3.010901 -1.146003 0.380195
C -5.189482 -1.599848 -0.587770
C -4.529543 0.676863 -0.152871
C -5.464713 -0.237236 -0.624786
C -3.330587 0.203108 0.360362
C -3.969276 -2.035192 -0.083160
F -3.738564 -3.342405 -0.075466
F -2.470981 1.104078 0.842219
F -6.088066 -2.460543 -1.037436
F -6.618689 0.189315 -1.106829
F -4.796472 1.973762 -0.185939

^{Tos}YSnPy
E = -2148.01079010
C 3.938511 -0.454982 -1.122464
H 3.920592 0.162177 -2.017480
C 2.791279 -1.138967 -0.757915
O 0.999249 -2.361776 -2.222844
C -1.063000 3.492552 -0.420208
H -0.403762 3.817413 0.384093
C 5.071569 -0.558398 -0.321661
H 5.973523 -0.013943 -0.598802
C -0.477667 -0.547981 2.388592
H 0.064380 -1.321741 1.848686
C -0.739826 0.667116 1.753562
C 2.289635 1.451356 1.387991
H 1.895132 0.848884 2.203471
C 3.562625 1.997688 1.482306
H 4.160371 1.821078 2.374263
C 3.310041 2.977259 -0.705583
H 3.710949 3.565743 -1.528328

C 2.036046 2.431829 -0.808851
H 1.450413 2.575807 -1.713521
C -1.119145 2.143279 -0.780778
C 2.767687 -1.943453 0.377415
H 1.858087 -2.486436 0.631511
C -1.442316 1.664492 2.430194
H -1.665064 2.609077 1.940383
C 1.517562 1.675344 0.245121
C 6.276543 -1.404404 1.723481
H 6.464812 -2.428978 2.065206
H 6.140214 -0.785895 2.621707
H 7.174092 -1.045218 1.209314
C 4.070887 2.765354 0.438810
H 5.068504 3.193951 0.515552
C 0.020436 -0.621196 -0.716536
P -0.110220 0.885134 0.062370
O 1.621049 0.042093 -2.759873
S 1.313548 -0.981572 -1.754088
C 5.069816 -1.333878 0.836590
C 3.901337 -2.032149 1.168275
H 3.886370 -2.656629 2.060957
C -0.924644 -0.764480 3.687414
H -0.725288 -1.717322 4.173656
C -1.631346 0.227793 4.357074
H -1.986506 0.054781 5.371176
C -1.886835 1.442534 3.727966
H -2.441321 2.221015 4.248318
C -2.635316 4.021179 -2.165724
H -3.228874 4.754943 -2.708010
C -1.826982 4.427926 -1.108220
H -1.781968 5.477951 -0.825890
C -2.671668 2.682232 -2.542265
H -3.289039 2.366712 -3.380772
C -1.912543 1.743198 -1.854707
H -1.917811 0.693606 -2.143770
Sn -1.330935 -2.300381 -0.937951
C -3.214595 -1.540420 -0.029123
N -3.108029 -0.246556 0.188597
C -5.455270 -1.552751 0.784465
C -4.131571 0.434733 0.694040
C -5.335837 -0.178908 1.010550
H -6.384571 -2.068900 1.022653
H -3.979081 1.505700 0.845152
H -6.159700 0.400348 1.420469
C -4.385420 -2.255324 0.254144
H -4.447301 -3.325050 0.065242

^{Tos}YSnPyr
E = -2164.06987180
C 3.999874 -0.470021 -1.116844
H 3.966254 0.179189 -1.988414
C 2.847562 -1.134017 -0.730766
O 1.033234 -2.269050 -2.235650
C -0.896996 3.527963 -0.565236
H -0.291957 3.871798 0.273141
C 5.160746 -0.636418 -0.367790
H 6.067615 -0.109776 -0.662605
C -0.802838 -0.449055 2.407310
H -0.222271 -1.259238 1.969938
C -0.915978 0.759563 1.717512

C 2.221107 1.326864 1.622565
H 1.743681 0.691969 2.366355
C 3.503955 1.809359 1.843452
H 4.027436 1.551086 2.761669
C 3.451400 2.933760 -0.288158
H 3.935238 3.553129 -1.040668
C 2.169308 2.448693 -0.519607
H 1.661027 2.665373 -1.455822
C -0.966068 2.164904 -0.868829
C 2.843400 -1.974729 0.378320
H 1.925579 -2.489669 0.658432
C -1.667761 1.800761 2.262275
H -1.777213 2.739106 1.724688
C 1.544399 1.655641 0.445474
C 6.423174 -1.604732 1.587191
H 6.635032 -2.658482 1.804908
H 6.316220 -1.092613 2.553313
H 7.295987 -1.179393 1.081197
C 4.116385 2.619477 0.891529
H 5.121423 2.998757 1.066854
C 0.076861 -0.623753 -0.598043
P -0.081435 0.915825 0.112097
O 1.621967 0.176872 -2.617870
S 1.348578 -0.924180 -1.683624
C 5.181837 -1.455511 0.759833
C 4.005065 -2.126864 1.116893
H 4.005234 -2.779734 1.989150
C -1.452441 -0.616465 3.625370
H -1.372967 -1.565493 4.151643
C -2.208571 0.419404 4.162070
H -2.721731 0.283963 5.112208
C -2.309582 1.629822 3.482400
H -2.899796 2.443161 3.900083
C -2.319789 4.014511 -2.445664
H -2.848886 4.737463 -3.063860
C -1.578680 4.449536 -1.350333
H -1.522747 5.510251 -1.113622
C -2.371241 2.660857 -2.761192
H -2.935963 2.322311 -3.627322
C -1.692932 1.735220 -1.977357
H -1.710330 0.674519 -2.220542
Sn -1.320608 -2.246196 -0.895071
C -3.249777 -1.546721 -0.102411
N -4.273301 -2.366929 0.124787
N -3.243677 -0.231605 0.096492
C -5.373254 -1.810209 0.612619
C -4.342388 0.318064 0.594411
C -5.468519 -0.445632 0.882059
H -6.218842 -2.476140 0.801210
H -4.321056 1.396795 0.765456
H -6.374617 -0.003496 1.287606

^{Tos}YSnB2
E = -2307.03326626
C 2.858100 -2.497691 -1.432310
H 2.529133 -2.231710 -2.433923
C 1.997203 -2.292120 -0.366026
O -0.603885 -2.607714 -0.274019
C 0.585902 3.287103 -1.950277
H 1.583069 3.627822 -1.672390

C 4.127133 -3.013955 -1.190967
H 4.808933 -3.169257 -2.026377
C 1.603552 1.654322 2.544142
H 1.811583 0.585372 2.555916
C 1.146819 2.253210 1.363020
C 3.726495 0.780638 0.007724
H 3.618055 0.845449 1.087398
C 4.974108 0.512544 -0.539154
H 5.830156 0.367194 0.116477
Sn -1.292113 -0.411569 1.913381
C 4.026078 0.603384 -2.751832
H 4.138489 0.523996 -3.831239
C 2.772662 0.864497 -2.210708
H 1.904485 0.958533 -2.857977
C 0.044882 2.140027 -1.360076
C 2.380499 -2.616696 0.931949
H 1.684525 -2.458892 1.754574
C 0.839787 3.613942 1.359502
H 0.454153 4.087050 0.460567
C 2.621730 0.970043 -0.826056
C 5.933141 -3.833332 0.364634
H 5.925653 -4.664081 1.080112
H 6.568436 -3.045575 0.793078
H 6.414030 -4.180042 -0.556006
C 5.126405 0.430900 -1.919888
H 6.105417 0.223038 -2.347659
C 0.221516 -0.254803 0.403389
P 0.963715 1.200751 -0.108773
O 0.413929 -1.100935 -2.052658
S 0.377374 -1.590806 -0.660146
C 4.548402 -3.322015 0.101873
C 3.650795 -3.121264 1.158328
H 3.956452 -3.370095 2.174157
C 1.768953 2.412897 3.694895
H 2.124102 1.938491 4.607364
C 1.466405 3.771356 3.683361
H 1.587159 4.364180 4.587932
C 0.998503 4.367050 2.518304
H 0.745876 5.425335 2.509173
C -1.405615 3.528523 -3.281328
H -1.975022 4.072955 -4.032451
C -0.142860 3.982160 -2.906030
H 0.276682 4.875096 -3.365226
C -1.932349 2.375823 -2.710442
H -2.911170 2.009888 -3.013592
C -1.207898 1.673880 -1.752632
H -1.599982 0.752973 -1.327385
C -5.388765 -1.108408 0.493660
C -4.737752 -0.877682 -0.715389
C -6.738770 -1.391600 0.553209
C -5.402324 -0.922946 -1.924469
C -7.422149 -1.436235 -0.664194
H -7.234352 -1.571091 1.503376
C -6.768910 -1.207888 -1.875182
H -4.878203 -0.753076 -2.861128
H -8.487309 -1.657370 -0.666459
H -7.334451 -1.256492 -2.803230
B -3.275565 -0.713412 0.908283
O -4.485762 -1.003642 1.511450
O -3.422557 -0.621028 -0.467000

^{Tos}YSnB1
E = -2576.21982219
C 4.258038 0.973620 0.640759
H 3.982758 1.367708 1.616104
C 3.303854 0.949703 -0.365096
O 1.443938 2.726928 -0.882285
C -0.913529 -2.232969 2.532236
H -0.478595 -3.190544 2.249150
C 5.524660 0.461026 0.388149
H 6.270720 0.465387 1.182241
C 0.753448 -2.606673 -1.948113
H 1.580198 -1.923484 -2.140319
C -0.007113 -2.452591 -0.780509
C 3.020418 -2.398622 0.499091
H 2.834332 -2.769516 -0.504546
C 4.226290 -2.696417 1.120699
H 4.970654 -3.289451 0.592749
Sn -1.005774 0.724823 -1.937360
C 3.528456 -1.472967 3.072634
H 3.725915 -1.102962 4.076699
C 2.321315 -1.168676 2.455636
H 1.590130 -0.547626 2.966565
C -0.659409 -1.090776 1.765000
C 3.612123 0.447827 -1.624840
H 2.851653 0.450210 -2.404258
C -1.120299 -3.265555 -0.572899
H -1.765736 -3.114010 0.288008
C 2.056073 -1.639605 1.166379
C 7.198678 -0.692575 -1.095436
H 7.459300 -0.694452 -2.159343
H 7.216712 -1.737122 -0.752010
H 7.986270 -0.160628 -0.550001
C 4.480425 -2.238529 2.409282
H 5.425481 -2.474819 2.894884
C 0.594892 0.309067 -0.557776
P 0.496052 -1.133253 0.360233
O 1.585856 1.704606 1.435419
S 1.648704 1.552279 -0.034835
C 5.852001 -0.078638 -0.856525
C 4.878635 -0.066500 -1.861417
H 5.121439 -0.467658 -2.844926
C 0.426106 -3.588943 -2.870093
H 1.021040 -3.700993 -3.774268
C -0.672667 -4.415830 -2.646475
H -0.933360 -5.183164 -3.372893
C -1.446225 -4.244938 -1.506066
H -2.325286 -4.863562 -1.341248
C -2.210055 -0.905459 4.067433
H -2.813484 -0.832254 4.970537
C -1.701056 -2.141305 3.671263
H -1.903466 -3.031540 4.263624
C -1.935569 0.233944 3.320453
H -2.318485 1.202696 3.632382
C -1.163751 0.144360 2.164799
H -0.904454 1.031690 1.591164
N -3.736052 0.697625 0.046904
C -3.564637 2.622262 1.180735
C -4.232515 1.448420 1.107678
H -3.723639 3.455822 1.855761

H -5.065415 1.102176 1.708467
B -2.681432 1.458177 -0.572433
C -1.642239 3.706621 0.092016
C -1.436146 4.383018 -1.108029
C -0.874868 4.022631 1.210568
C -0.465179 5.371581 -1.183563
H -2.044448 4.126742 -1.972270
C 0.091954 5.015826 1.128399
H -1.017315 3.466039 2.134894
C 0.297823 5.693083 -0.066626
H -0.300924 5.891784 -2.125001
H 0.704270 5.239737 1.999142
H 1.065688 6.460890 -0.133541
C -4.084360 -0.649399 -0.153066
C -4.207739 -1.162463 -1.446401
C -4.301609 -1.490684 0.940558
C -4.555851 -2.493167 -1.637242
H -4.050672 -0.502678 -2.295895
C -4.661028 -2.818559 0.741178
H -4.161216 -1.104318 1.947248
C -4.793816 -3.325498 -0.547381
H -4.652231 -2.878963 -2.650276
H -4.828991 -3.461407 1.603429
H -5.081390 -4.363702 -0.702434
N -2.614400 2.679245 0.170922

$^{Ph}Y_2Sn$
E = -2619.33185873
P 2.521447 0.467738 0.277689
P -2.776852 0.140985 -0.135035
C 2.129242 -0.321891 -1.179904
C 2.910602 -1.428134 -1.717268
C 3.656685 -2.306465 -0.903227
H 3.640868 -2.171445 0.175982
C 4.383769 -3.361365 -1.439733
H 4.941750 -4.016109 -0.771530
C 4.389790 -3.595201 -2.810357
H 4.956144 -4.424202 -3.229603
C 3.650335 -2.750600 -3.635061
H 3.639286 -2.913951 -4.711879
C 2.931238 -1.690884 -3.103346
H 2.375171 -1.028378 -3.765645
C 1.857620 -0.270784 1.810710
C 2.566114 -1.258544 2.504879
H 3.604235 -1.467660 2.251458
C 1.950160 -1.976089 3.521319
H 2.507076 -2.747143 4.050016
C 0.622657 -1.720442 3.854338
H 0.135792 -2.300129 4.635907
C -0.083390 -0.730957 3.181465
H -1.122634 -0.526627 3.430680
C 0.531284 -0.012839 2.163610
H -0.036532 0.728719 1.612019
C 1.917897 2.187454 0.250889
C 1.574411 2.872270 1.417945
H 1.571584 2.355160 2.374563
C 1.228065 4.220582 1.360992
H 0.944198 4.743130 2.272555
C 1.263770 4.899075 0.149272
H 1.002780 5.955051 0.108482

C 1.648704 4.228759 -1.010976
H 1.697040 4.760370 -1.959385
C 1.963773 2.877505 -0.964731
H 2.253238 2.338950 -1.864968
C 4.313510 0.661515 0.577290
C 5.190840 0.593366 -0.506130
H 4.806196 0.346608 -1.493212
C 6.546484 0.834899 -0.316864
H 7.228083 0.769605 -1.162549
C 7.030015 1.156329 0.947121
H 8.092252 1.344523 1.092137
C 6.155571 1.241738 2.026206
H 6.529914 1.501817 3.014469
C 4.800065 0.995937 1.843158
H 4.116024 1.064674 2.687864
C -1.311028 -0.737692 -0.342725
C -1.134862 -2.030691 0.302525
C 0.088610 -2.734716 0.193988
H 0.882556 -2.323067 -0.421154
C 0.313337 -3.927872 0.858748
H 1.277561 -4.419849 0.737973
C -0.667479 -4.490107 1.673013
H -0.487100 -5.425233 2.199446
C -1.889750 -3.836609 1.783073
H -2.684442 -4.261347 2.395278
C -2.122498 -2.648973 1.103399
H -3.104354 -2.198463 1.194802
C -3.067132 0.681936 1.596983
C -2.393702 1.808066 2.085550
H -1.787861 2.417223 1.420807
C -2.482559 2.163665 3.424296
H -1.947431 3.041163 3.781816
C -3.249265 1.403367 4.302744
H -3.313526 1.678478 5.353534
C -3.937393 0.293661 3.826329
H -4.548563 -0.302627 4.500984
C -3.849798 -0.064688 2.485407
H -4.407607 -0.928644 2.133426
C -2.787489 1.643022 -1.150889
C -3.845215 1.897469 -2.024929
H -4.669560 1.195002 -2.104274
C -3.850890 3.043209 -2.814565
H -4.679569 3.223249 -3.495893
C -2.802030 3.951530 -2.725023
H -2.810068 4.855557 -3.331376
C -1.741225 3.705728 -1.863302
H -0.918885 4.410797 -1.791608
C -1.711557 2.544888 -1.092396
H -0.866426 2.363930 -0.430761
C -4.304843 -0.746613 -0.601446
C -5.576551 -0.317582 -0.211046
H -5.683676 0.557731 0.428315
C -6.703968 -1.003232 -0.646426
H -7.693810 -0.666929 -0.343809
C -6.565690 -2.117849 -1.469956
H -7.449946 -2.655502 -1.807247
C -5.301425 -2.545858 -1.860364
H -5.192949 -3.418217 -2.501448
C -4.170236 -1.862022 -1.428210
H -3.171844 -2.188091 -1.719164

Sn 0.076374 -0.098052 -1.952901

[Ph]YSnCl
E = -1771.95276395
P -0.753674 -0.367719 -0.054429
C 0.606514 0.717553 0.154401
C 0.435260 2.144325 0.131865
C -0.756474 2.806067 0.509939
H -1.603021 2.221104 0.862387
C -0.844857 4.188098 0.499868
H -1.766867 4.672496 0.816426
C 0.242655 4.956411 0.089051
H 0.168204 6.042117 0.071858
C 1.428244 4.327748 -0.293101
H 2.279838 4.922965 -0.617101
C 1.534240 2.948835 -0.258794
H 2.451624 2.448685 -0.566623
C -1.576592 -0.743243 1.518693
C -2.810744 -1.401584 1.561153
H -3.318097 -1.672986 0.636602
C -3.398224 -1.696190 2.784787
H -4.358189 -2.207534 2.814093
C -2.761352 -1.334775 3.968694
H -3.224467 -1.565500 4.926037
C -1.538309 -0.674618 3.929958
H -1.041277 -0.386598 4.853671
C -0.944475 -0.376670 2.708814
H 0.009870 0.147872 2.678938
C -0.142017 -1.908319 -0.779361
C -0.747617 -3.138083 -0.520341
H -1.565951 -3.215439 0.190719
C -0.277779 -4.284666 -1.152467
H -0.741825 -5.245177 -0.938124
C 0.785288 -4.205175 -2.045055
H 1.153141 -5.106354 -2.532040
C 1.387182 -2.977432 -2.305893
H 2.237112 -2.906211 -2.980820
C 0.930119 -1.828515 -1.674752
H 1.441906 -0.881811 -1.848554
C -2.043442 0.227146 -1.196620
C -3.221624 0.832448 -0.750261
H -3.436147 0.899044 0.314478
C -4.128995 1.352516 -1.664810
H -5.045595 1.817921 -1.308235
C -3.865302 1.281671 -3.028443
H -4.576531 1.691667 -3.742675
C -2.690969 0.686785 -3.477866
H -2.478928 0.629632 -4.543357
C -1.783347 0.159807 -2.568504
H -0.870059 -0.309929 -2.927918
Sn 2.593519 -0.234191 0.986151
Cl 3.706476 0.168736 -1.264507

[Ph]YSnHMDS
E = -2185.73168332
P -2.000190 -0.070876 0.016350
C -0.485267 0.346525 -0.746637
C -0.093187 1.677973 -1.053029
C -0.769698 2.843536 -0.596401
H -1.665077 2.738790 0.011166

C -0.294541 4.110244 -0.873813
H -0.825049 4.980003 -0.490639
C 0.861781 4.278613 -1.638804
H 1.232122 5.277957 -1.858423
C 1.541682 3.156364 -2.112439
H 2.443647 3.278263 -2.709089
C 1.089686 1.884920 -1.813124
H 1.635562 1.012310 -2.158361
C -1.864530 0.083099 1.808568
C -2.248596 1.265780 2.449026
H -2.805977 2.027617 1.906810
C -1.910378 1.475105 3.778790
H -2.208535 2.397154 4.272871
C -1.176676 0.513500 4.471022
H -0.900053 0.686613 5.509140
C -0.797720 -0.665010 3.840410
H -0.219674 -1.413039 4.378360
C -1.140204 -0.890038 2.510833
H -0.825307 -1.803860 2.008869
C -2.366568 -1.789775 -0.391999
C -3.074041 -2.612770 0.489401
H -3.354094 -2.246502 1.475644
C -3.403758 -3.906856 0.108945
H -3.942985 -4.551425 0.800068
C -3.042728 -4.379458 -1.150194
H -3.298945 -5.395867 -1.442280
C -2.357164 -3.553817 -2.036138
H -2.074466 -3.921655 -3.020058
C -2.022249 -2.258628 -1.665013
H -1.475496 -1.612129 -2.350441
C -3.493820 0.826085 -0.511172
C -3.502068 1.479339 -1.744965
H -2.592828 1.523724 -2.341266
C -4.669830 2.079561 -2.201685
H -4.672382 2.592119 -3.161335
C -5.828180 2.027573 -1.433685
H -6.739889 2.501016 -1.792754
C -5.824533 1.369086 -0.207376
H -6.731668 1.324103 0.391794
C -4.661948 0.764698 0.253421
H -4.660884 0.244505 1.210469
Sn 1.105966 -1.540811 -0.532246
N 2.815897 -0.239582 -0.019512
Si 4.211646 -0.325466 -1.050479
Si 2.878869 0.406833 1.592844
C 3.766417 -0.981859 -2.765756
H 3.389383 -2.011895 -2.737080
H 3.011697 -0.371370 -3.279450
H 4.673394 -0.975906 -3.387050
C 5.029185 1.353617 -1.342040
H 5.697572 1.643722 -0.524046
H 5.627158 1.325040 -2.263116
H 4.271619 2.140046 -1.450745
C 5.516896 -1.509038 -0.372506
H 6.406098 -1.551988 -1.016064
H 5.844632 -1.217398 0.633539
H 5.102039 -2.523472 -0.300041
C 4.479469 1.331232 1.980865
H 4.484837 1.582904 3.050408
H 5.384588 0.747306 1.774148

H 4.545023 2.270694 1.419003
C 2.743104 -0.981213 2.868245
H 2.665006 -0.590365 3.892225
H 1.864631 -1.610138 2.673403
H 3.628471 -1.629328 2.814719
C 1.515747 1.659539 1.926043
H 0.514029 1.243363 1.784654
H 1.582334 2.022916 2.961033
H 1.617844 2.520257 1.251678

$^{Ph}YSnC_6F_5$
E = -2040.27492747
P -1.589135 0.170764 0.289056
C -0.386057 0.325011 -0.979148
C 0.448414 1.517244 -1.063722
C 1.194432 2.017547 0.022761
H 1.125850 1.532224 0.992419
C 2.110325 3.046808 -0.153684
H 2.714739 3.371875 0.691229
C 2.269184 3.647428 -1.396870
H 2.986848 4.454434 -1.528728
C 1.515320 3.192615 -2.477332
H 1.635881 3.649014 -3.457962
C 0.641420 2.129888 -2.321213
H 0.095790 1.739800 -3.178548
C -1.067058 0.676608 1.948449
C -1.408831 1.940777 2.436027
H -2.108911 2.567791 1.888660
C -0.839911 2.405332 3.615957
H -1.108577 3.390857 3.990530
C 0.077019 1.620875 4.307710
H 0.530803 1.994177 5.223597
C 0.416779 0.358654 3.827994
H 1.139618 -0.259658 4.355132
C -0.152468 -0.116047 2.654013
H 0.122466 -1.100024 2.285037
C -2.223743 -1.511521 0.358064
C -2.408866 -2.204158 1.555353
H -2.149356 -1.743416 2.504582
C -2.934207 -3.490942 1.537263
H -3.068887 -4.030368 2.472139
C -3.288291 -4.083502 0.328072
H -3.699836 -5.090942 0.317605
C -3.125340 -3.389707 -0.864218
H -3.409373 -3.847274 -1.809208
C -2.589988 -2.106027 -0.856523
H -2.495278 -1.551485 -1.787194
C -3.018333 1.220595 -0.118102
C -2.860048 2.352914 -0.918888
H -1.885220 2.596983 -1.333839
C -3.956018 3.166463 -1.186286
H -3.830937 4.048114 -1.811350
C -5.204792 2.852204 -0.661690
H -6.060647 3.489271 -0.876256
C -5.363930 1.720006 0.132532
H -6.341961 1.469703 0.538353
C -4.274677 0.902399 0.404599
H -4.399684 0.011362 1.017837
C 2.093401 -1.175876 -0.793014
C 2.711565 -1.390718 1.555156

C 3.973851 0.071465 0.130359
C 3.750351 -0.485378 1.383072
Sn 0.437670 -1.386992 -2.411763
C 3.144398 -0.284426 -0.927572
C 1.905979 -1.705031 0.468524
F 0.862756 -2.517694 0.716583
F 3.358690 0.343267 -2.084647
F 4.934084 0.974125 -0.022089
F 4.514911 -0.148309 2.411646
F 2.469663 -1.899533 2.764330

^{Ph}YSnPy
E = -1559.42247741
P -1.167920 -0.401333 -0.171307
C 0.034292 0.682442 0.435855
C -0.249073 2.103847 0.543447
C -1.516321 2.640702 0.846078
C -1.721759 4.011285 0.915480
C -0.667269 4.890612 0.687435
C 0.597911 4.382862 0.392508
C 0.806593 3.015425 0.325867
C -2.482132 -0.948790 0.970942
C -3.654675 -1.561814 0.516375
H -3.819047 -1.700762 -0.551476
C -4.614656 -1.985239 1.426861
H -5.526041 -2.461493 1.070683
C -4.411357 -1.797359 2.791698
H -5.166238 -2.127534 3.502823
C -3.249047 -1.184763 3.246263
H -3.090311 -1.033823 4.311985
C -2.284994 -0.759256 2.338608
H -1.371057 -0.275561 2.684323
C -0.340654 -1.897428 -0.779038
C -0.922633 -3.162469 -0.703446
H -1.886447 -3.299169 -0.219823
C -0.251528 -4.265014 -1.222945
H -0.702591 -5.252637 -1.150719
C 0.992731 -4.107219 -1.822650
H 1.515272 -4.973324 -2.224754
C 1.573459 -2.844550 -1.900987
H 2.553445 -2.714787 -2.355865
C 0.915620 -1.739117 -1.377385
H 1.387112 -0.754553 -1.414302
C -2.036716 0.330839 -1.596832
C -3.277214 0.959689 -1.463443
H -3.797440 0.949472 -0.508377
C -3.855072 1.602314 -2.551877
H -4.821633 2.088957 -2.438234
C -3.198923 1.627989 -3.777403
H -3.652769 2.133166 -4.627789
C -1.960369 1.009684 -3.913624
H -1.440956 1.029091 -4.869427
C -1.379201 0.365684 -2.829182
H -0.408913 -0.112332 -2.941930
C 3.462571 0.013692 0.330668
C 5.845609 -0.158911 0.006901
C 4.374639 0.643584 -1.688014
C 5.667842 0.339203 -1.283269
Sn 1.665643 -0.211936 1.635321
N 3.305827 0.484256 -0.910886

H 6.840671 -0.414196 0.370130
H 6.508289 0.487689 -1.957711
H 4.189985 1.035721 -2.691179
H -2.340958 1.963427 1.064675
H -2.710545 4.397021 1.158804
H -0.828361 5.965584 0.742891
H 1.427384 5.063545 0.208094
H 1.785258 2.610121 0.071461
C 4.736403 -0.320396 0.820181
H 4.847312 -0.701786 1.835064

^{Ph}*YSnPyr*
E = -1575.48047566
P -1.191639 -0.331502 0.169242
C 0.086851 0.741941 -0.311696
C -0.028121 2.173970 -0.389524
C 1.154443 2.940788 -0.247499
C 1.129300 4.321890 -0.328994
C -0.068043 4.993323 -0.575814
C -1.243944 4.262816 -0.734301
C -1.229756 2.880740 -0.636887
C -0.622382 -1.404690 1.511743
C -1.519620 -2.292068 2.115244
H -2.550755 -2.350600 1.767718
C -1.096241 -3.090443 3.170127
H -1.790787 -3.787332 3.634892
C 0.211503 -2.986973 3.637479
H 0.540592 -3.610507 4.466802
C 1.090427 -2.078034 3.058567
H 2.106855 -1.979661 3.434389
C 0.680359 -1.282183 1.993680
H 1.362663 -0.549987 1.561634
C -1.735345 -1.334501 -1.238342
C -1.854356 -2.724071 -1.199545
H -1.595193 -3.272157 -0.297314
C -2.271742 -3.412405 -2.332443
H -2.350615 -4.497175 -2.303421
C -2.572763 -2.721696 -3.502089
H -2.892856 -3.266307 -4.388114
C -2.452029 -1.335661 -3.543751
H -2.672768 -0.793981 -4.461060
C -2.030642 -0.641152 -2.418476
H -1.904303 0.440776 -2.458756
C -2.692199 0.427138 0.870932
C -2.524234 1.252328 1.987868
H -1.531122 1.392547 2.413994
C -3.618874 1.897912 2.543517
H -3.484357 2.542637 3.409561
C -4.885903 1.723099 1.991199
H -5.743586 2.231040 2.427927
C -5.055962 0.900320 0.884174
H -6.045918 0.759930 0.454681
C -3.960967 0.252160 0.319212
H -4.095934 -0.387866 -0.550304
C 3.422430 -0.111354 -0.105570
C 5.664274 -0.488026 0.182455
C 4.410948 0.645986 1.821030
C 5.645089 0.154855 1.418277
Sn 1.717900 -0.403585 -1.537609
N 3.316780 0.518099 1.080223

N 4.582178 -0.620060 -0.566454
H 6.593127 -0.910012 -0.212020
H 6.538385 0.263942 2.028386
H 4.299204 1.163128 2.778311
H 2.079254 2.409414 -0.033059
H 2.052308 4.884575 -0.200754
H -0.084063 6.079122 -0.648841
H -2.181164 4.776758 -0.940948
H -2.157080 2.331179 -0.781700

^{Ph}YSnB2
E = -1718.44247543
P 1.457546 -0.567780 0.143769
C 0.629378 0.883917 -0.265483
C 1.339125 2.159990 -0.047905
C 1.895676 2.897681 -1.105646
C 2.544746 4.105903 -0.881306
C 2.664849 4.611510 0.408836
C 2.117524 3.897624 1.470746
C 1.461571 2.694655 1.246350
C 2.725057 -1.154915 -1.039645
C 3.732741 -2.039027 -0.640200
H 3.781429 -2.379111 0.393348
C 4.677567 -2.477381 -1.559474
H 5.459874 -3.165650 -1.245320
C 4.626711 -2.031496 -2.877669
H 5.371181 -2.371941 -3.594941
C 3.630811 -1.146915 -3.275767
H 3.592585 -0.792318 -4.303743
C 2.681650 -0.707547 -2.359047
H 1.899969 -0.012011 -2.662700
C 0.271926 -1.920122 0.349470
C 0.503803 -3.209889 -0.129334
H 1.420771 -3.438692 -0.667483
C -0.455462 -4.198679 0.060326
H -0.279191 -5.200873 -0.325046
C -1.641073 -3.905943 0.726500
H -2.392432 -4.681455 0.863106
C -1.871530 -2.621316 1.210687
H -2.803393 -2.381592 1.719570
C -0.917781 -1.629320 1.025444
H -1.104502 -0.615960 1.374652
C 2.395940 -0.408525 1.692119
C 3.573512 0.346293 1.673723
H 3.932210 0.770610 0.736926
C 4.275490 0.566426 2.850535
H 5.187289 1.159683 2.831734
C 3.809188 0.035595 4.050053
H 4.361081 0.208644 4.971996
C 2.639441 -0.715924 4.071485
H 2.275746 -1.134804 5.007598
C 1.929540 -0.937290 2.895789
H 1.014761 -1.525889 2.915590
H 1.810852 2.505611 -2.118485
H 2.962897 4.654493 -1.723864
H 3.174294 5.556998 0.584734
H 2.192654 4.285609 2.485469
H 1.020853 2.150759 2.079799
Sn -0.949464 0.726574 -1.762809
C -4.530843 0.381657 0.805632

C -5.124010 0.361411 -0.454421
C -5.271398 0.250134 1.964204
C -6.487317 0.210386 -0.615271
C -6.651842 0.094914 1.811703
H -4.796009 0.272635 2.941394
C -7.245919 0.076086 0.550350
H -6.937641 0.197099 -1.604126
H -7.275386 -0.010836 2.696949
H -8.324151 -0.045184 0.470703
B -2.949588 0.605301 -0.716087
O -4.158147 0.503143 -1.400457
O -3.187130 0.538899 0.657932

^{Ph}YSnB1
E = -1987.63310702
P -0.653296 1.486838 0.037329
C 0.694614 0.694012 -0.709340
C 2.046369 1.125784 -0.397408
C 2.402591 1.740479 0.825989
H 1.644999 1.897187 1.592122
C 3.704102 2.141002 1.090394
H 3.933772 2.605817 2.048143
C 4.707099 1.957453 0.144768
H 5.726194 2.280104 0.348583
C 4.386102 1.342323 -1.064166
H 5.159530 1.175741 -1.812172
C 3.091945 0.927580 -1.326258
H 2.853908 0.442156 -2.272239
C -1.162973 0.743869 1.619988
C -2.403542 1.028421 2.201550
H -3.131423 1.633740 1.663126
C -2.706501 0.536273 3.464774
H -3.671215 0.760660 3.915696
C -1.776771 -0.241563 4.150938
H -2.013637 -0.620290 5.143580
C -0.555784 -0.551692 3.561356
H 0.161159 -1.184779 4.078682
C -0.249397 -0.067657 2.293802
H 0.696287 -0.323809 1.816320
C -2.130328 1.486966 -1.016824
C -3.058944 0.444303 -0.946588
H -2.939549 -0.357564 -0.221213
C -4.154008 0.438349 -1.803509
H -4.875714 -0.371093 -1.735489
C -4.315146 1.449988 -2.743672
H -5.169534 1.434620 -3.417857
C -3.388448 2.483911 -2.819689
H -3.512055 3.279441 -3.551543
C -2.303090 2.508874 -1.952470
H -1.588753 3.326464 -2.008807
C -0.325170 3.251650 0.389627
C -0.815180 3.914028 1.515302
H -1.414238 3.381148 2.249298
C -0.525540 5.260009 1.712944
H -0.910336 5.769390 2.594272
C 0.255770 5.950222 0.793135
H 0.483633 7.002374 0.952916
C 0.755927 5.291187 -0.325626
H 1.381029 5.822299 -1.040449
C 0.473646 3.946455 -0.525193

H 0.888454 3.420997 -1.384011
Sn 0.219530 -1.029305 -2.033561
N 1.578132 -2.692996 0.590081
C -0.116614 -3.490003 1.814729
C 1.222425 -3.316911 1.781194
H -0.717696 -3.991018 2.564853
H 1.968757 -3.652587 2.492604
B 0.382386 -2.431055 -0.173215
N -0.674036 -2.985206 0.644642
C -2.059446 -2.907762 0.441052
C -2.913433 -2.569520 1.492694
C -2.597802 -3.151001 -0.826069
C -4.283143 -2.466626 1.278623
H -2.492403 -2.353738 2.471028
C -3.966976 -3.045788 -1.032339
H -1.932673 -3.434339 -1.637376
C -4.817120 -2.702902 0.015990
H -4.934476 -2.190379 2.105890
H -4.372781 -3.240272 -2.023416
H -5.889919 -2.624941 -0.149652
C 2.915484 -2.359197 0.294869
C 3.455164 -2.667069 -0.954505
C 3.715315 -1.737379 1.254562
C 4.782282 -2.364421 -1.231850
H 2.827451 -3.158701 -1.693324
C 5.043768 -1.445403 0.973170
H 3.283314 -1.468403 2.216898
C 5.583933 -1.761974 -0.268387
H 5.194772 -2.613992 -2.207717
H 5.654387 -0.949372 1.724857
H 6.623989 -1.529114 -0.487834

$^{F}Y_2Sn$
E = -3613.13916598
P -3.038406 -0.926759 0.037592
P 3.060407 -0.891292 0.106741
C -1.780254 -0.013695 -0.774658
C -1.739242 1.386088 -0.972769
C -2.527603 2.342460 -0.279351
C -2.382430 3.707420 -0.424649
C -1.380249 4.209694 -1.250694
C -0.556113 3.321836 -1.939426
C -0.733563 1.961293 -1.798832
C -2.712369 -1.052003 1.811392
C -3.297889 -0.160421 2.712065
H -4.068099 0.529034 2.377893
C -2.878069 -0.141082 4.036405
H -3.329215 0.561694 4.733389
C -1.873681 -1.003740 4.463031
H -1.538265 -0.976349 5.498082
C -1.300606 -1.903870 3.571325
H -0.520833 -2.584373 3.908390
C -1.716980 -1.935983 2.245885
H -1.258820 -2.626760 1.537942
C -3.044800 -2.584450 -0.658909
C -3.430473 -3.686866 0.120433
H -3.612056 -3.567692 1.186608
C -3.559135 -4.937129 -0.466321
H -3.835720 -5.791745 0.148268
C -3.341230 -5.102133 -1.832690

H -3.443271 -6.085264 -2.287123
C -3.012057 -3.998223 -2.619585
H -2.858641 -4.117200 -3.690206
C -2.876356 -2.744964 -2.046713
H -2.616293 -1.886484 -2.664302
C -4.761096 -0.384051 -0.192591
C -5.089947 0.411829 -1.291032
H -4.304369 0.794476 -1.940756
C -6.419328 0.720021 -1.551274
H -6.671063 1.346539 -2.404513
C -7.424282 0.232567 -0.721871
H -8.464621 0.478220 -0.925419
C -7.099575 -0.570611 0.367038
H -7.884250 -0.955640 1.015206
C -5.772041 -0.884200 0.630804
H -5.520828 -1.517180 1.480789
C 1.517468 -0.183962 0.115780
C 1.371903 1.225768 0.377306
C 0.344898 1.714306 1.208458
C 0.143746 3.060595 1.457769
C 0.990092 4.011515 0.900958
C 2.034947 3.585373 0.090650
C 2.186826 2.233502 -0.174842
C 4.148962 -0.172262 1.382390
C 3.998975 -0.563780 2.715124
H 3.301494 -1.357563 2.972389
C 4.741049 0.052635 3.713959
H 4.622132 -0.264232 4.748243
C 5.632256 1.072533 3.393088
H 6.210234 1.558127 4.177134
C 5.781161 1.470634 2.069861
H 6.472993 2.270675 1.813812
C 5.045814 0.851049 1.064782
H 5.163939 1.168920 0.032132
C 2.826959 -2.658891 0.508706
C 3.531229 -3.692645 -0.108786
H 4.263347 -3.470765 -0.880699
C 3.282412 -5.015779 0.242434
H 3.830624 -5.815622 -0.251217
C 2.330595 -5.316521 1.211365
H 2.136489 -6.353230 1.480330
C 1.619276 -4.292288 1.826788
H 0.863266 -4.526179 2.574752
C 1.857145 -2.969181 1.472671
H 1.292595 -2.153508 1.922512
C 4.093178 -0.901268 -1.408445
C 5.443226 -1.262622 -1.385015
H 5.921878 -1.520259 -0.440428
C 6.178991 -1.284908 -2.564282
H 7.230932 -1.563624 -2.542606
C 5.570497 -0.945083 -3.769502
H 6.147845 -0.961041 -4.692179
C 4.230485 -0.572961 -3.793771
H 3.757752 -0.292630 -4.732923
C 3.493346 -0.546452 -2.615358
H 2.451218 -0.226974 -2.614721
Sn 0.046389 -1.510529 -0.997687
F 3.162236 1.888184 -1.019471
F 2.847097 4.474373 -0.462251
F 0.787968 5.302269 1.125904

F -0.881203 3.452745 2.207671
F -0.503866 0.859696 1.770246
F 0.095756 1.167402 -2.460803
F 0.425421 3.791717 -2.689623
F -1.205492 5.509758 -1.375022
F -3.143827 4.538780 0.263516
F -3.418121 1.922768 0.613613

^{F}YSnCl
E = -2268.85323125
P -1.096665 0.651189 0.103077
C 0.123552 -0.585548 0.370066
C 1.519378 -0.237768 0.282941
C 2.448468 -0.707882 1.226984
C 3.803582 -0.440542 1.121699
C 4.281419 0.303690 0.047353
C 3.396417 0.777086 -0.915464
C 2.045816 0.511421 -0.783336
C -2.335536 0.489681 1.419872
C -2.461243 1.430490 2.442410
H -1.832098 2.316577 2.457930
C -3.407174 1.240749 3.444392
H -3.502535 1.976620 4.239990
C -4.227192 0.118353 3.427705
H -4.965172 -0.027570 4.214096
C -4.111670 -0.814663 2.401257
H -4.758115 -1.689532 2.379766
C -3.173455 -0.630716 1.394341
H -3.081910 -1.361868 0.589845
C -2.013827 0.477297 -1.438448
C -3.258103 1.108068 -1.552288
H -3.678293 1.651650 -0.707015
C -3.960542 1.027290 -2.747138
H -4.927234 1.517637 -2.841086
C -3.430862 0.308898 -3.816347
H -3.987252 0.239744 -4.749279
C -2.201129 -0.328266 -3.692902
H -1.793372 -0.904635 -4.520365
C -1.481810 -0.246730 -2.505582
H -0.528549 -0.765450 -2.407504
C -0.411861 2.323013 0.213271
C 0.453133 2.606366 1.277289
H 0.660544 1.845584 2.028888
C 1.053854 3.854735 1.369896
H 1.724912 4.071316 2.198357
C 0.804692 4.819549 0.398409
H 1.281127 5.795363 0.467738
C -0.044547 4.535357 -0.665968
H -0.231042 5.286329 -1.430679
C -0.651657 3.289001 -0.764356
H -1.301069 3.062797 -1.607112
Sn -0.416311 -2.898071 0.264651
Cl 0.926095 -2.686344 -1.888272
F 1.224217 0.949884 -1.731866
F 3.851969 1.470962 -1.944060
F 5.571346 0.562297 -0.055607
F 4.646566 -0.874255 2.043228
F 2.026316 -1.396065 2.274082

^{F}YSnHMDS
E = -2682.63265904
P -2.122507 -0.233396 -0.066856
C -0.507892 0.183775 -0.599400
C 0.091408 1.466057 -0.542596
C -0.299718 2.512421 0.327663
C 0.360014 3.720892 0.409678
C 1.454768 3.961001 -0.418531
C 1.866254 2.979438 -1.316771
C 1.215370 1.760781 -1.358839
C -2.199777 -0.712098 1.673758
C -2.624450 0.187985 2.655438
H -3.042740 1.150224 2.372568
C -2.492251 -0.142116 3.997307
H -2.821276 0.561011 4.759214
C -1.923447 -1.359864 4.365069
H -1.807397 -1.607598 5.418440
C -1.509209 -2.260729 3.391796
H -1.066941 -3.212829 3.677070
C -1.652255 -1.948486 2.044075
H -1.319669 -2.649870 1.280777
C -2.604568 -1.676222 -1.040871
C -3.514910 -2.609484 -0.533948
H -3.887115 -2.514579 0.484640
C -3.929344 -3.670968 -1.326600
H -4.628757 -4.402071 -0.926461
C -3.447314 -3.803039 -2.626538
H -3.770074 -4.639524 -3.243082
C -2.553803 -2.867124 -3.137822
H -2.174436 -2.969492 -4.152187
C -2.134472 -1.800409 -2.353541
H -1.428786 -1.070623 -2.748065
C -3.433249 0.985433 -0.379868
C -3.205527 2.051319 -1.250861
H -2.224277 2.190298 -1.699350
C -4.236115 2.936036 -1.546821
H -4.054004 3.768666 -2.222982
C -5.493439 2.757865 -0.980723
H -6.297388 3.453736 -1.211989
C -5.725955 1.688392 -0.120636
H -6.710430 1.544983 0.319834
C -4.701041 0.800385 0.177537
H -4.885252 -0.036572 0.849785
Sn 0.886634 -1.880382 -0.742391
N 2.598731 -0.843344 0.162885
Si 4.103463 -0.925093 -0.717976
Si 2.590863 -0.633674 1.890871
C 3.859700 -1.500562 -2.497204
H 3.475018 -2.526616 -2.561145
H 3.180037 -0.849934 -3.059457
H 4.837164 -1.482348 -3.000263
C 4.961034 0.747103 -0.822830
H 5.222443 1.150965 0.161096
H 5.886705 0.666305 -1.408755
H 4.311462 1.472045 -1.327183
C 5.288534 -2.184482 0.045934
H 6.230225 -2.236777 -0.517034
H 5.536764 -1.962861 1.090787
H 4.829283 -3.182419 0.023961
C 4.110139 0.274017 2.549922

H 4.057133 0.296762 3.647080
H 5.066030 -0.184579 2.273404
H 4.122079 1.313944 2.199320
C 2.484869 -2.303323 2.765284
H 2.464284 -2.196498 3.858277
H 1.574656 -2.836805 2.457828
H 3.342969 -2.934396 2.498233
C 1.150139 0.417167 2.498807
H 0.190341 0.135121 2.057052
H 1.052119 0.317607 3.588590
H 1.328588 1.477224 2.277804
F 1.621547 0.880951 -2.253404
F -1.314513 2.308420 1.160924
F -0.029897 4.647540 1.268530
F 2.078752 5.120295 -0.370697
F 2.875720 3.231321 -2.132391

$^{F}YSnC_6F_5$
E = -2537.18658094
P -0.521141 1.362731 0.523718
C -0.749870 0.099237 -0.665573
C -1.728355 -0.943264 -0.510228
C -2.614972 -1.255321 -1.553995
C -3.547488 -2.275090 -1.450137
C -3.602520 -3.043891 -0.291932
C -2.732125 -2.775464 0.760163
C -1.823228 -1.739676 0.642255
C -0.916146 2.940115 -0.272353
C -0.659577 4.130033 0.414441
H -0.165195 4.107147 1.384959
C -1.034424 5.344747 -0.145880
H -0.823814 6.271803 0.383108
C -1.682842 5.372933 -1.376716
H -1.977639 6.325359 -1.812734
C -1.961442 4.186273 -2.048125
H -2.475099 4.206634 -3.006711
C -1.577988 2.967790 -1.501392
H -1.795334 2.038101 -2.025234
C 1.174369 1.412208 1.135113
C 2.090241 2.339094 0.634263
H 1.781314 3.066484 -0.112868
C 3.408255 2.311712 1.077514
H 4.124458 3.024528 0.676215
C 3.814485 1.358196 2.003176
H 4.851436 1.326261 2.330012
C 2.907483 0.415488 2.480933
H 3.234816 -0.352455 3.178735
C 1.589757 0.431642 2.044431
H 0.888158 -0.319860 2.399267
C -1.651553 1.308189 1.946569
C -3.007124 1.107740 1.663774
H -3.340035 1.000248 0.632299
C -3.933246 1.057246 2.696254
H -4.985377 0.896578 2.470949
C -3.515385 1.215506 4.014411
H -4.241571 1.173418 4.823622
C -2.172269 1.435599 4.296669
H -1.845584 1.571842 5.325490
C -1.237749 1.482591 3.267698
H -0.190307 1.660464 3.497694

C 2.177278 -0.889608 -1.127932
C 4.358534 -1.015563 -0.044506
C 2.701988 -2.686387 0.438412
C 3.972000 -2.159918 0.638891
Sn 0.741901 0.288110 -2.495883
C 1.838920 -2.044110 -0.441214
C 3.455769 -0.406409 -0.905944
F 3.869423 0.714227 -1.510147
F 0.635360 -2.607924 -0.607798
F 2.334408 -3.781943 1.090988
F 4.802938 -2.733800 1.497957
F 5.552398 -0.481727 0.190811
F -0.975723 -1.528052 1.644042
F -2.775817 -3.514319 1.854434
F -4.482525 -4.021463 -0.188455
F -4.389514 -2.518441 -2.438988
F -2.611264 -0.519095 -2.657359

^{F}YSnPy
E = -2056.31320271
P -1.770954 0.181082 0.032291
C -0.162560 -0.290717 -0.147779
C 0.971374 0.599218 0.075039
C 1.862887 0.436502 1.147382
C 3.068411 1.115660 1.238260
C 3.391385 2.069262 0.283843
C 2.521478 2.292194 -0.777306
C 1.346233 1.563143 -0.876258
C -2.438891 0.235141 1.737023
C -3.611399 0.931588 2.045624
H -4.148429 1.466690 1.262745
C -4.083478 0.950816 3.352108
H -4.995382 1.494282 3.592338
C -3.383707 0.282700 4.354318
H -3.751963 0.303491 5.378429
C -2.210103 -0.398976 4.051517
H -1.656456 -0.909608 4.836990
C -1.734267 -0.422357 2.744200
H -0.804200 -0.932513 2.494815
C -2.825651 -0.970352 -0.886455
C -4.019137 -1.482237 -0.375367
H -4.351074 -1.202291 0.621706
C -4.778511 -2.362755 -1.137932
H -5.706170 -2.763826 -0.734857
C -4.350338 -2.734192 -2.408939
H -4.947052 -3.424785 -3.002057
C -3.158644 -2.231045 -2.920030
H -2.818622 -2.527430 -3.910129
C -2.390305 -1.354781 -2.161403
H -1.448975 -0.959016 -2.540673
C -2.060533 1.868415 -0.568589
C -1.578261 2.926369 0.207575
H -1.157049 2.730018 1.193055
C -1.632307 4.226000 -0.279787
H -1.247854 5.046327 0.322990
C -2.177889 4.476399 -1.534831
H -2.221122 5.495013 -1.915397
C -2.669321 3.425801 -2.303223
H -3.099831 3.621455 -3.283244
C -2.605202 2.122110 -1.827204

H -2.975750 1.301089 -2.437434
C 2.629020 -2.137385 -0.449545
C 4.796459 -2.043150 0.553922
C 4.237214 -0.838259 -1.430839
C 5.173701 -1.162825 -0.456302
F 0.591773 1.762391 -1.947564
F 2.840008 3.183269 -1.706414
F 4.529929 2.741238 0.367099
F 3.906115 0.867099 2.235300
F 1.606319 -0.469466 2.090064
Sn 0.410687 -2.395855 -0.252025
N 2.998980 -1.326428 -1.421324
H 5.499688 -2.319170 1.338250
H 6.172157 -0.732620 -0.487862
H 4.489909 -0.155799 -2.244613
C 3.502103 -2.546766 0.563463
H 3.159397 -3.203704 1.359799

^{F}*YSnPyr*
E = -2072.38256343
P 0.353327 1.307976 0.311843
C -0.053129 -0.164798 -0.464157
C 0.974330 -1.209506 -0.448689
C 0.950856 -2.239677 0.498189
C 1.971201 -3.176366 0.598065
C 3.059220 -3.103554 -0.262761
C 3.117600 -2.098074 -1.221092
C 2.085616 -1.175822 -1.299911
C -0.424279 1.533682 1.941157
C 0.049748 2.504290 2.830916
H 0.886771 3.142573 2.548253
C -0.537499 2.641686 4.081334
H -0.168122 3.394400 4.775131
C -1.592520 1.808130 4.448048
H -2.049374 1.914081 5.430407
C -2.051612 0.835486 3.568021
H -2.867014 0.175410 3.857585
C -1.467996 0.687907 2.312671
H -1.814270 -0.096972 1.639109
C -0.061583 2.762705 -0.691688
C -0.673475 3.899559 -0.163205
H -0.947426 3.936682 0.888176
C -0.957948 4.980726 -0.990723
H -1.447131 5.860506 -0.577638
C -0.628615 4.934403 -2.340491
H -0.855671 5.781749 -2.984637
C -0.018584 3.800962 -2.871203
H 0.231646 3.758396 -3.929191
C 0.257730 2.713672 -2.053764
H 0.710254 1.811209 -2.462028
C 2.134193 1.325793 0.671435
C 2.583342 0.518540 1.723185
H 1.861862 0.048144 2.390662
C 3.942668 0.299475 1.900650
H 4.286841 -0.337491 2.712889
C 4.860027 0.889299 1.035109
H 5.925318 0.712555 1.169653
C 4.417379 1.706062 0.000255
H 5.134618 2.171321 -0.672621
C 3.056565 1.922906 -0.186792

H 2.713825 2.543501 -1.011773
C -3.486504 -1.352540 -0.574640
C -5.550808 -2.293238 -0.692552
C -4.344840 -2.245167 1.333450
C -5.496990 -2.627047 0.657953
F 2.186918 -0.209371 -2.208477
F 4.157538 -2.025946 -2.037727
F 4.036057 -3.990508 -0.173346
F 1.927721 -4.126246 1.520491
F -0.038828 -2.307002 1.375581
Sn -1.833798 -0.358102 -1.659268
N -3.349605 -1.609847 0.727251
N -4.559877 -1.665279 -1.308154
H -6.426157 -2.544218 -1.296903
H -6.309878 -3.149909 1.155317
H -4.218961 -2.455895 2.398340

^{F}YSnB2
E = -2215.34854599
P -1.005171 1.236960 0.327628
C -0.498929 -0.141397 -0.554658
C -1.304777 -1.359720 -0.461510
C -2.179525 -1.781490 -1.468087
C -2.932336 -2.943104 -1.360786
C -2.841463 -3.717463 -0.210738
C -1.987708 -3.329450 0.814699
C -1.233755 -2.173566 0.676164
C -2.031142 2.455806 -0.563367
C -2.701214 3.464423 0.137287
H -2.611597 3.526795 1.221576
C -3.490245 4.378134 -0.548495
H -4.010414 5.162932 -0.002776
C -3.621659 4.283020 -1.932229
H -4.244057 4.997405 -2.467828
C -2.970881 3.270531 -2.627372
H -3.085168 3.186602 -3.706116
C -2.176984 2.353703 -1.945050
H -1.684052 1.541332 -2.476776
C 0.439576 2.119416 0.972890
C 0.594594 3.504575 0.914131
H -0.173168 4.125263 0.458624
C 1.752806 4.089832 1.414141
H 1.879484 5.168927 1.355612
C 2.752058 3.299326 1.972227
H 3.660368 3.762071 2.353525
C 2.598292 1.917006 2.033006
H 3.384946 1.294072 2.454595
C 1.446036 1.324845 1.533564
H 1.330337 0.242054 1.542176
C -2.062207 0.750224 1.721600
C -3.362815 0.326456 1.429238
H -3.736005 0.385069 0.407186
C -4.174594 -0.171554 2.439262
H -5.183381 -0.506506 2.206995
C -3.695914 -0.241487 3.744724
H -4.332292 -0.632804 4.535997
C -2.406746 0.188291 4.038837
H -2.033315 0.135778 5.059359
C -1.585891 0.679388 3.029913
H -0.572973 1.001166 3.260908

Sn 1.081492 0.087514 -2.007532
C 4.557708 -1.055565 0.445385
C 5.217648 -0.653437 -0.712967
C 5.241274 -1.488680 1.564619
C 6.595238 -0.666730 -0.808591
C 6.635642 -1.504225 1.478415
H 4.712164 -1.805263 2.459703
C 7.297005 -1.102770 0.317926
H 7.098609 -0.351358 -1.718456
H 7.216843 -1.839743 2.334667
H 8.384125 -1.130206 0.287697
B 3.053638 -0.438973 -1.036841
O 4.294105 -0.274842 -1.639308
O 3.215099 -0.934443 0.253237
F -0.420061 -1.840729 1.673080
F -2.318720 -1.048366 -2.568649
F -3.753239 -3.306490 -2.334699
F -1.896192 -4.068954 1.909366
F -3.562216 -4.821084 -0.094297

^{F}YSnB1
E = -2484.52838376
P 1.951344 -1.262957 -0.007097
C 0.602534 -0.253727 -0.186874
C 0.644174 1.077132 -0.764609
C -0.223078 1.487493 -1.792412
C -0.249600 2.782532 -2.288839
C 0.633436 3.732960 -1.795754
C 1.517841 3.371163 -0.789470
C 1.498947 2.084048 -0.280049
C 2.014661 -2.687141 -1.156732
C 3.122422 -3.541485 -1.172747
H 3.946257 -3.378591 -0.477873
C 3.181912 -4.586240 -2.084812
H 4.044288 -5.250085 -2.094412
C 2.143551 -4.775527 -2.994784
H 2.193407 -5.592762 -3.711968
C 1.054482 -3.912680 -2.998545
H 0.251540 -4.047258 -3.720520
C 0.989930 -2.864903 -2.083959
H 0.155579 -2.163799 -2.095782
C 2.009840 -1.951099 1.677000
C 2.139809 -3.303676 1.987692
H 2.225772 -4.044162 1.196504
C 2.131531 -3.712000 3.318807
H 2.220391 -4.769819 3.558085
C 2.001705 -2.775459 4.337275
H 1.995141 -3.100115 5.376114
C 1.866695 -1.423375 4.029437
H 1.753251 -0.690030 4.825554
C 1.858651 -1.012153 2.704690
H 1.719593 0.035882 2.440252
C 3.536572 -0.448429 -0.342536
C 3.717020 0.097048 -1.616928
H 2.937822 -0.013941 -2.370208
C 4.886354 0.782294 -1.917635
H 5.020855 1.213896 -2.907337
C 5.882871 0.915011 -0.954925
H 6.799150 1.452115 -1.192084
C 5.709593 0.360575 0.308257

H 6.489606 0.461423 1.060246
C 4.535933 -0.316940 0.619928
H 4.395956 -0.734835 1.614519
Sn -1.318544 -1.100829 0.361478
N -2.755729 1.838720 1.008336
C -4.910662 1.520718 0.469994
C -3.994403 2.447221 0.819189
H -5.973831 1.652111 0.307040
H -4.143314 3.503401 1.009177
B -2.910381 0.433720 0.724541
N -4.310173 0.267605 0.415635
C -4.989506 -0.889927 0.008169
C -5.963184 -0.834569 -0.992521
C -4.686536 -2.121340 0.595527
C -6.616231 -1.991292 -1.396573
H -6.184888 0.116077 -1.472197
C -5.332772 -3.276687 0.173606
H -3.965819 -2.157822 1.410610
C -6.302110 -3.218831 -0.821238
H -7.368406 -1.933414 -2.181174
H -5.084997 -4.227897 0.640930
H -6.811812 -4.123602 -1.145400
C -1.629764 2.566166 1.438144
C -0.741329 2.010987 2.363344
C -1.412422 3.867386 0.979972
C 0.336104 2.754161 2.823754
H -0.915036 1.000065 2.727313
C -0.332597 4.604920 1.450478
H -2.080933 4.292651 0.234426
C 0.549153 4.053056 2.372221
H 1.019264 2.312461 3.547146
H -0.163542 5.609401 1.066982
H 1.402811 4.625873 2.726411
F -1.066557 0.610068 -2.332419
F -1.099419 3.111072 -3.252327
F 0.610600 4.978324 -2.252725
F 2.322366 4.285471 -0.264021
F 2.311123 1.828552 0.745543

I
E = -1021.43970102
C 1.613774 2.514697 -0.142016
C -0.542553 1.327291 -0.209549
C -1.155427 2.587753 -0.447048
C -0.420514 3.747462 -0.502305
C 0.978537 3.716372 -0.330205
H 2.697791 2.510586 -0.037904
H -2.228991 2.619842 -0.609402
H -0.921755 4.693851 -0.696978
H 1.554155 4.639102 -0.367468
N -1.220316 0.172863 -0.137265
C -2.596996 0.083330 0.021952
C -3.309254 0.888012 0.928520
C -3.302368 -0.913703 -0.670107
C -4.672915 0.712164 1.109987
H -2.771750 1.635679 1.507390
C -4.667326 -1.082700 -0.482935
H -2.760258 -1.545341 -1.373392
C -5.364729 -0.268888 0.403525
H -5.200859 1.342013 1.824409

H -5.190845 -1.858779 -1.038767
H -6.434141 -0.403343 0.551539
C 0.914702 1.270296 -0.088476
C 1.514149 0.009718 0.072876
P 3.199043 -0.228256 0.231316
C 4.236671 0.296900 -1.175396
H 4.129576 1.373823 -1.337208
H 5.291553 0.054096 -1.002350
H 3.880549 -0.221851 -2.071970
C 3.965140 0.529183 1.701477
H 5.034635 0.294777 1.753258
H 3.828293 1.614549 1.675343
H 3.456773 0.139719 2.589730
C 3.517597 -2.000153 0.395807
H 4.586283 -2.199482 0.527261
H 2.958736 -2.382684 1.257884
H 3.158306 -2.510572 -0.506184
Sn 0.024752 -1.761772 -0.173414

//
E = -1570.62964588
C -0.668280 3.080533 0.069676
C -0.209929 1.788358 -0.139719
C 0.967087 1.336120 0.493587
C 1.633950 2.217513 1.368930
C 1.147007 3.493007 1.599443
C -0.002057 3.932376 0.944096
H -1.546385 3.402481 -0.486417
H 2.523961 1.861055 1.883269
H 1.668269 4.150349 2.292355
H -0.371853 4.942885 1.105963
N 1.431417 0.052109 0.309291
S -1.061056 0.723141 -1.311235
O -2.211811 1.537870 -1.771539
O -0.036088 0.335531 -2.277495
C -1.590701 -0.566442 -0.357940
C 2.750680 -0.149559 0.068554
C 3.562394 0.836987 -0.550716
C 3.335749 -1.402924 0.378457
C 4.889923 0.578427 -0.826420
H 3.104411 1.773322 -0.857614
C 4.670746 -1.638491 0.111902
H 2.727141 -2.160443 0.865953
C 5.455194 -0.654128 -0.490438
H 5.492210 1.334408 -1.325962
H 5.109274 -2.599014 0.373971
H 6.502590 -0.850816 -0.709724
P -3.195587 -0.407048 0.202457
C -3.599467 1.121030 1.103759
H -4.633796 1.110398 1.466065
H -2.907497 1.231003 1.945857
H -3.457304 1.955530 0.409969
C -3.558305 -1.751521 1.357582
H -2.843619 -1.718553 2.187319
H -4.581101 -1.653899 1.738095
H -3.452044 -2.713190 0.844271
C -4.447633 -0.515767 -1.103660
H -4.214788 0.275509 -1.823615
H -4.350757 -1.487805 -1.598178
H -5.460974 -0.392932 -0.704058

Sn -0.049038 -1.990198 0.328001

III
E = -2332.69444789
C -0.406135 1.858060 -0.571661
C 0.405537 1.858234 0.571310
C 0.776214 3.078550 1.138970
C 0.386028 4.285571 0.575912
C -0.387590 4.285392 -0.576355
C -0.777303 3.078206 -1.139369
H 1.401479 3.053790 2.027523
H 0.693096 5.221492 1.038538
H -0.695037 5.221165 -1.039032
H -1.402554 3.053198 -2.027928
S -1.017458 0.419778 -1.530548
S 1.017153 0.420181 1.530342
O -2.194892 0.985738 -2.221340
O 0.134070 -0.008755 -2.339254
O -0.134356 -0.008363 2.338986
O 2.194423 0.986495 2.221121
C -1.514785 -0.794823 -0.467903
C 1.514915 -0.794448 0.468059
P 2.953150 -0.502499 -0.417751
P -2.952787 -0.503150 0.418347
C 3.022674 -1.607890 -1.838510
H 3.928428 -1.405682 -2.421170
H 3.020100 -2.651337 -1.507920
H 2.123059 -1.409687 -2.435432
C 3.060160 1.169608 -1.104765
H 3.046507 1.891328 -0.281580
H 3.985559 1.279690 -1.681238
H 2.183662 1.314903 -1.747047
C 4.457057 -0.723209 0.566381
H 4.513601 -1.764881 0.899299
H 5.361101 -0.455317 0.007880
H 4.339867 -0.080233 1.446207
C -3.021615 -1.608521 1.839148
H -3.926896 -1.406187 2.422502
H -3.019629 -2.651920 1.508379
H -2.121497 -1.410683 2.435409
C -3.060224 1.169057 1.105028
H -3.046341 1.890587 0.281687
H -3.985965 1.279052 1.680975
H -2.184111 1.314708 1.747765
C -4.456828 -0.724548 -0.565445
H -5.360977 -0.457214 -0.006846
H -4.340139 -0.081463 -1.445272
H -4.512785 -1.766235 -0.898413
Sn 0.000326 -2.223001 -0.000217

IV
E = -1540.73418205
C 0.781800 3.350629 0.551331
C 0.223656 2.091462 0.659050
C -1.076341 1.742585 0.266429
C -1.865044 2.785483 -0.244191
C -1.342213 4.067120 -0.348784
C -0.030770 4.358497 0.041728
H 1.807293 3.541401 0.860300
H -2.893530 2.599162 -0.544940

H -1.968986 4.867940 -0.737380
H 0.351520 5.372889 -0.047563
B -1.344125 0.237966 0.626774
S 1.092768 0.634495 1.138457
O 1.876139 0.727811 2.374840
O -0.280127 -0.202662 1.477937
C 1.778524 -0.029106 -0.186971
C -2.633583 -0.614223 0.587748
C -2.770475 -1.706730 1.460025
C -3.702503 -0.339850 -0.281844
C -3.919892 -2.485345 1.467866
H -1.953183 -1.936033 2.142064
C -4.852334 -1.116942 -0.279871
H -3.619576 0.481486 -0.992513
C -4.966467 -2.193158 0.597530
H -4.003629 -3.325244 2.155937
H -5.664292 -0.887723 -0.968143
H -5.868022 -2.803404 0.599839
P 2.997812 -1.172939 0.183962
C 4.473757 -0.436862 0.930929
H 5.226023 -1.200774 1.157490
H 4.885639 0.300949 0.234753
H 4.159223 0.073215 1.846998
C 3.536699 -1.969376 -1.342434
H 3.886792 -1.200419 -2.038900
H 4.343319 -2.681251 -1.135652
H 2.683193 -2.490746 -1.790892
C 2.472736 -2.492630 1.311870
H 2.168329 -2.016907 2.251587
H 1.600484 -2.990155 0.873338
H 3.270175 -3.219973 1.501209
Sn 0.128900 -0.410052 -1.828816

V
E = -991.495537440
C 1.738035 2.446534 -0.211971
C -0.447358 1.362096 -0.203448
C -1.016061 2.636999 -0.436538
C -0.251397 3.783470 -0.516620
C 1.139684 3.678530 -0.391582
H 2.825496 2.409520 -0.159429
H -2.097277 2.709149 -0.546212
H -0.720644 4.752482 -0.673766
H 1.760849 4.571228 -0.453788
B -1.378707 0.163836 -0.112419
C -2.895913 -0.013478 0.066544
C -3.619262 0.875296 0.887449
C -3.620695 -1.050822 -0.549567
C -4.989410 0.740853 1.069800
H -3.087654 1.677779 1.396756
C -4.990245 -1.183129 -0.376137
H -3.088455 -1.762918 -1.180050
C -5.682040 -0.286403 0.435423
H -5.521443 1.440615 1.712500
H -5.524526 -1.991429 -0.872804
H -6.756384 -0.391573 0.576302
C 0.990979 1.246971 -0.122898
C 1.576272 -0.061059 0.012088
P 3.232367 -0.326347 0.300044
C 4.401185 0.143036 -1.024217

H 4.316186 1.213312 -1.235544
H 5.435643 -0.093544 -0.748560
H 4.123970 -0.405982 -1.930472
C 3.887064 0.478743 1.798010
H 4.961900 0.299133 1.914221
H 3.698051 1.555439 1.746197
H 3.346120 0.079395 2.662182
C 3.555987 -2.090599 0.549025
H 4.618510 -2.255147 0.757660
H 2.955649 -2.453180 1.390061
H 3.273791 -2.644628 -0.352815
Sn 0.104546 -1.617517 -0.277063

6.2.3 H_2 activation transition states

Pathway A

$^{Tos}Y_2Sn$
E = -3797.68220505
S -2.291390 1.305580 0.449441
S 2.032856 -1.273997 0.329879
P -2.340082 -1.594449 0.148973
P 2.540509 1.581137 0.267158
O -1.363632 2.400370 0.075815
O -2.512477 1.039225 1.885422
O 0.971484 -2.238715 0.017683
O 2.373343 -1.081460 1.760690
C -1.756415 -0.074832 -0.387016
C -4.143799 -1.498125 0.442881
C -4.618415 -1.280048 1.738929
H -3.918235 -1.223093 2.567839
C -5.979842 -1.108366 1.963987
H -6.338500 -0.942906 2.978051
C -6.874709 -1.135062 0.901527
H -7.939494 -0.996752 1.080149
C -6.404513 -1.326677 -0.394041
H -7.097780 -1.338488 -1.232727
C -5.047095 -1.506721 -0.622830
H -4.696514 -1.660344 -1.639041
C -2.017261 -2.805328 -1.155793
C -2.391117 -2.494846 -2.467603
H -2.847166 -1.530188 -2.684591
C -2.133146 -3.389075 -3.496226
H -2.428677 -3.143426 -4.514290
C -1.470678 -4.583398 -3.227391
H -1.255380 -5.279613 -4.035934
C -1.060210 -4.874025 -1.932326
H -0.508872 -5.788941 -1.726042
C -1.330253 -3.989015 -0.894648
H -0.961056 -4.194078 0.106105
C -1.714017 -2.265847 1.710248
C -2.267779 -3.423973 2.265228
H -3.091904 -3.932831 1.765538
C -1.766994 -3.919533 3.461333
H -2.194657 -4.821134 3.895804
C -0.716578 -3.263105 4.101924
H -0.322514 -3.659326 5.036323
C -0.169729 -2.111232 3.550427
H 0.666658 -1.600878 4.022269
C -0.676469 -1.610274 2.358175
H -0.247740 -0.722726 1.909329

C -3.890920 1.774442 -0.196739
C -4.063664 1.893533 -1.572563
H -3.223553 1.707153 -2.239982
C -5.310903 2.229470 -2.072639
H -5.450989 2.319097 -3.149428
C -6.396533 2.455028 -1.216800
C -6.188832 2.347228 0.157363
H -7.020688 2.521305 0.838890
C -4.942716 2.007026 0.674188
H -4.774701 1.899734 1.742927
C -7.751663 2.774898 -1.772753
H -8.237441 1.870695 -2.165427
H -7.687985 3.490712 -2.600723
H -8.410909 3.195923 -1.006432
C 1.713929 0.231908 -0.408306
C 2.098679 3.015758 -0.745707
C 1.501766 4.139499 -0.176899
H 1.315913 4.176077 0.893226
C 1.112219 5.201124 -0.986546
H 0.631605 6.068127 -0.538896
C 1.320164 5.148310 -2.358835
H 1.011341 5.980797 -2.988233
C 1.907741 4.024339 -2.933154
H 2.056097 3.972641 -4.009731
C 2.284761 2.955499 -2.132860
H 2.711995 2.059802 -2.581326
C 4.354713 1.400005 0.254374
C 5.147261 1.986970 -0.734442
H 4.709787 2.670457 -1.456979
C 6.507916 1.708674 -0.795854
H 7.116795 2.171877 -1.569831
C 7.087972 0.847431 0.128868
H 8.152391 0.625921 0.074755
C 6.306398 0.276570 1.127657
H 6.753818 -0.397885 1.854864
C 4.946447 0.550648 1.197120
H 4.328449 0.081943 1.959992
C 2.110180 2.005853 1.976015
C 2.992523 2.657630 2.839096
H 4.004731 2.894301 2.516506
C 2.572042 2.996683 4.120668
H 3.260453 3.497115 4.799075
C 1.276925 2.697110 4.536082
H 0.955620 2.966612 5.540745
C 0.393766 2.059778 3.669957
H -0.628350 1.830622 3.964296
C 0.815290 1.711889 2.394695
H 0.127361 1.228354 1.711148
C 3.553299 -1.909540 -0.378771
C 4.008190 -1.495073 -1.622801
H 3.415168 -0.784394 -2.194734
C 5.223998 -1.968382 -2.096986
H 5.587873 -1.631828 -3.067169
C 5.995548 -2.858484 -1.345439
C 5.502949 -3.279285 -0.107537
H 6.087272 -3.978546 0.489725
C 4.292297 -2.807917 0.381800
H 3.924199 -3.104363 1.361589
C 7.333886 -3.317162 -1.841464
H 7.659811 -4.231611 -1.334536

H 8.099075 -2.549137 -1.660644
H 7.319152 -3.509521 -2.920480
H 0.035832 -0.152464 -3.167108
H 0.210482 -1.295084 -2.405152
Sn -0.062761 0.463349 -1.562262

^{Tos}YSnCl
E = -2361.68717424
S -0.644702 -1.457964 -1.264304
P 0.510283 0.927283 0.113661
O -0.320830 -2.901850 -1.198983
O -0.592118 -0.760358 -2.552795
C 0.371439 -0.766182 -0.075068
C 0.796562 1.293618 1.868724
C 1.621990 2.335046 2.291287
H 2.147239 2.949208 1.564320
C 1.800377 2.569267 3.650078
H 2.454983 3.375193 3.975302
C 1.156929 1.771174 4.589195
H 1.303883 1.955000 5.651681
C 0.332387 0.730966 4.170182
H -0.167310 0.100229 4.902670
C 0.154068 0.488360 2.814771
H -0.472094 -0.335133 2.473664
C -1.044649 1.739578 -0.354761
C -2.069265 1.900392 0.581329
H -1.918623 1.602089 1.616123
C -3.284488 2.446452 0.190750
H -4.080274 2.569061 0.922385
C -3.481212 2.833999 -1.131075
H -4.433628 3.264630 -1.434635
C -2.466528 2.664321 -2.066452
H -2.622991 2.955236 -3.103085
C -1.249327 2.112575 -1.685494
H -0.466260 1.951216 -2.422101
C 1.798924 1.754586 -0.852641
C 1.803230 3.145768 -1.003961
H 1.016831 3.748731 -0.550907
C 2.801249 3.755857 -1.751874
H 2.803029 4.837208 -1.873491
C 3.791085 2.981428 -2.352915
H 4.570496 3.461230 -2.941946
C 3.778393 1.598790 -2.212347
H 4.542794 0.988646 -2.688462
C 2.781477 0.979858 -1.465945
H 2.768750 -0.103690 -1.372094
C -2.326792 -1.284588 -0.687077
C -2.663649 -1.706667 0.595863
H -1.904382 -2.161662 1.229800
C -3.960933 -1.531536 1.047383
H -4.228106 -1.855467 2.052698
C -4.938638 -0.949266 0.229106
C -4.576617 -0.558998 -1.058791
H -5.324223 -0.108422 -1.710310
C -3.275177 -0.721414 -1.523820
H -2.977160 -0.402772 -2.519652
C -6.334094 -0.749547 0.739085
H -6.761907 -1.690253 1.107410
H -6.994798 -0.360604 -0.042295
H -6.351140 -0.040464 1.577491

Sn 1.696055 -2.267110 0.566142
H 2.695498 -0.805039 1.485405
H 2.372796 -2.096821 2.133281
Cl 3.487526 -2.631121 -1.004432

^{Tos}YSnHMDS
E = -2775.47081470
S 1.293664 -1.923235 0.212997
P 0.766193 0.946297 0.314656
O 0.807543 -3.163683 -0.389628
O 1.336786 -1.823560 1.689029
C 0.462235 -0.567541 -0.447289
C 0.044430 2.257056 -0.714720
C -0.648370 3.328948 -0.151157
H -0.823168 3.362444 0.921247
C -1.120388 4.356733 -0.962017
H -1.667560 5.184444 -0.516161
C -0.899882 4.322080 -2.333599
H -1.272410 5.125990 -2.965262
C -0.212381 3.252946 -2.902349
H -0.046798 3.217675 -3.976870
C 0.256773 2.222122 -2.099133
H 0.792397 1.381326 -2.537057
C 2.531209 1.364783 0.477709
C 3.166999 2.221094 -0.423046
H 2.598357 2.725361 -1.199978
C 4.536565 2.438120 -0.327885
H 5.025281 3.110218 -1.030369
C 5.277108 1.797630 0.659079
H 6.351391 1.960533 0.726303
C 4.643868 0.951294 1.563736
H 5.220515 0.443515 2.333887
C 3.273714 0.740134 1.485229
H 2.778738 0.059442 2.176172
C 0.112168 1.183419 1.991298
C 0.614443 2.192373 2.819215
H 1.428274 2.828646 2.474462
C 0.079878 2.375728 4.087589
H 0.470800 3.160008 4.732700
C -0.946304 1.547516 4.534549
H -1.358287 1.684886 5.532530
C -1.439160 0.539439 3.713770
H -2.231192 -0.115069 4.070421
C -0.913205 0.354921 2.440147
H -1.287325 -0.429681 1.787648
C 3.014773 -1.794015 -0.275841
C 3.419768 -1.117333 -1.417109
H 2.674526 -0.627302 -2.040618
C 4.772604 -1.037474 -1.717593
H 5.092625 -0.484953 -2.600323
C 5.731952 -1.641886 -0.901269
C 5.294194 -2.349434 0.220843
H 6.025705 -2.837451 0.863951
C 3.946086 -2.423145 0.542222
H 3.605470 -2.938957 1.437180
C 7.192675 -1.492461 -1.201157
H 7.789162 -2.258734 -0.695018
H 7.557706 -0.512257 -0.862085
H 7.392663 -1.556130 -2.276912
Sn -1.339227 -0.780104 -1.586711

H -1.422453 -1.937869 -2.851832
H -1.330512 -2.721367 -1.734969
N -3.029611 -0.775591 -0.365214
Si -3.449261 -2.213691 0.565429
Si -4.125994 0.581932 -0.523355
C -4.088055 1.672820 1.008049
H -3.076890 2.069879 1.161968
H -4.775100 2.523111 0.901815
H -4.365653 1.124157 1.915215
C -5.891528 0.020451 -0.859498
H -6.303387 -0.624628 -0.075393
H -6.552867 0.892177 -0.953751
H -5.933645 -0.536525 -1.804598
C -3.639042 1.660057 -1.985580
H -2.627757 2.073273 -1.892444
H -3.691779 1.109915 -2.934112
H -4.333752 2.509151 -2.050223
C -4.442420 -3.395775 -0.505734
H -4.698893 -4.311563 0.043094
H -5.372242 -2.941357 -0.868204
H -3.843918 -3.688670 -1.378949
C -1.966498 -3.145501 1.220350
H -1.431405 -3.687376 0.433844
H -1.217878 -2.527827 1.730360
H -2.333926 -3.882641 1.948902
C -4.477907 -1.716681 2.068081
H -5.385879 -1.152489 1.828227
H -4.782758 -2.622475 2.608684
H -3.877911 -1.107121 2.755177

$^{Tos}YSnC_6F_5$
E = -2630.02508219
S -1.951098 -0.142696 -1.904565
P -0.426875 0.611942 0.514020
O -1.782333 -1.172779 -2.944021
O -1.981891 1.276694 -2.292721
C -0.745577 -0.487080 -0.751333
C 0.298697 -0.348908 1.873619
C 1.414922 0.062206 2.603701
H 1.919482 0.992978 2.360518
C 1.915878 -0.743375 3.619736
H 2.802901 -0.430410 4.165274
C 1.298714 -1.951288 3.924418
H 1.696715 -2.578820 4.719229
C 0.188786 -2.368333 3.197967
H -0.284841 -3.322163 3.420469
C -0.302431 -1.579011 2.166319
H -1.145364 -1.913647 1.562823
C -1.947507 1.407582 1.106094
C -2.770465 0.787243 2.048578
H -2.478950 -0.160129 2.495016
C -3.965730 1.386313 2.423746
H -4.603379 0.901451 3.159944
C -4.344820 2.600474 1.859048
H -5.281961 3.067773 2.155650
C -3.532663 3.212263 0.911124
H -3.832882 4.155318 0.459246
C -2.335890 2.618608 0.528633
H -1.713946 3.081079 -0.233765
C 0.680170 1.994637 0.121178

C 1.053309 2.951184 1.073179
H 0.661456 2.898374 2.088286
C 1.918191 3.976286 0.717407
H 2.218400 4.714141 1.458739
C 2.398077 4.061758 -0.589054
H 3.084895 4.860637 -0.861788
C 1.989987 3.140872 -1.545383
H 2.352383 3.209361 -2.568345
C 1.120907 2.112161 -1.194403
H 0.785146 1.393069 -1.939241
C -3.526193 -0.451856 -1.115699
C -3.734926 -1.652647 -0.443360
H -2.934469 -2.389450 -0.399413
C -4.955375 -1.883488 0.169360
H -5.121229 -2.819995 0.700949
C -5.983727 -0.933123 0.112956
C -5.752499 0.251383 -0.584241
H -6.541288 1.000504 -0.640579
C -4.529042 0.499830 -1.199167
H -4.331458 1.427869 -1.730074
C -7.293059 -1.190426 0.796298
H -8.016348 -0.395002 0.589927
H -7.165258 -1.253964 1.885173
H -7.732494 -2.141242 0.469932
Sn 0.689861 -1.864934 -1.536943
H 0.784863 -2.389810 -3.157814
H 1.019385 -0.991753 -3.238756
C 2.638912 -1.086532 -0.848813
C 3.363112 -0.064149 -1.450411
C 3.108813 -1.535857 0.376462
C 4.451143 0.539200 -0.830597
C 4.206985 -0.977304 1.016737
C 4.872077 0.080209 0.410712
F 2.475690 -2.521428 1.010646
F 3.033510 0.416875 -2.642833
F 4.603472 -1.416836 2.202803
F 5.907125 0.640991 1.009374
F 5.080986 1.550421 -1.407372

^{Tos}YSnPy
E = -2149.17493559
S 0.343799 -1.761623 0.149363
P 0.335071 1.190057 0.222607
O -0.438476 -2.776406 -0.595370
O 0.242425 -1.792750 1.624324
C -0.111880 -0.276546 -0.527484
C -0.039547 2.532784 -0.934212
C -0.542116 3.760573 -0.502842
H -0.743014 3.929364 0.552319
C -0.813420 4.761201 -1.428465
H -1.215452 5.713532 -1.089037
C -0.584771 4.542831 -2.782822
H -0.804946 5.326658 -3.504942
C -0.082682 3.319452 -3.215795
H 0.089799 3.142898 -4.275469
C 0.189946 2.314197 -2.296517
H 0.563955 1.346318 -2.626886
C 2.117516 1.260644 0.564626
C 2.996553 1.939922 -0.280394
H 2.612031 2.538669 -1.103152

C 4.367887 1.847727 -0.072516
H 5.050252 2.382247 -0.730408
C 4.864626 1.071175 0.968807
H 5.939354 0.990527 1.122812
C 3.989167 0.397241 1.814641
H 4.375561 -0.217416 2.624867
C 2.617020 0.496203 1.624130
H 1.930177 -0.051818 2.268042
C -0.450249 1.578369 1.810930
C 0.081023 2.554436 2.658720
H 0.992586 3.082591 2.380650
C -0.547456 2.837791 3.864536
H -0.134048 3.595470 4.527260
C -1.699073 2.143703 4.227173
H -2.187941 2.363799 5.174459
C -2.218002 1.161828 3.389981
H -3.109626 0.608504 3.677371
C -1.593596 0.874434 2.182373
H -1.976428 0.093333 1.531266
C 2.077778 -2.056721 -0.171726
C 2.721311 -1.474221 -1.255504
H 2.153800 -0.841027 -1.934482
C 4.084473 -1.669600 -1.421428
H 4.595127 -1.194097 -2.257943
C 4.817195 -2.450890 -0.523380
C 4.138550 -3.051899 0.539047
H 4.691452 -3.671679 1.243927
C 2.776961 -2.854323 0.725573
H 2.252458 -3.283294 1.575999
C 6.300542 -2.598437 -0.677536
H 6.684789 -3.443613 -0.097091
H 6.816239 -1.693284 -0.326337
H 6.584313 -2.744063 -1.726226
Sn -1.928587 -0.645458 -1.640736
H -2.568461 1.156851 -1.153900
H -2.447600 0.769915 -2.502261
C -3.539183 -1.417381 -0.394666
C -5.800553 -1.655759 -0.178670
C -4.392910 -2.569183 1.518802
C -5.672051 -2.339420 1.025985
H -6.788936 -1.463957 -0.601992
H -4.244418 -3.108423 2.453428
H -6.555306 -2.687369 1.557565
N -4.760662 -1.206346 -0.873712
C -3.299303 -2.106402 0.796437
H -2.282702 -2.277330 1.145569

^{Tos}YSnPyr
E = -2165.23134254
S 0.304358 -1.727575 0.329907
P 0.314266 1.230980 0.201566
O -0.496962 -2.775390 -0.344070
O 0.273771 -1.658184 1.801065
C -0.153953 -0.280718 -0.434615
C -0.104112 2.482092 -1.040665
C -0.702028 3.696413 -0.704682
H -0.937837 3.921310 0.332543
C -1.024870 4.608793 -1.702846
H -1.501762 5.549990 -1.437285
C -0.753112 4.315713 -3.034513

H -1.014004 5.029977 -3.813045
C -0.154865 3.105458 -3.373063
H 0.052658 2.869842 -4.414825
C 0.168476 2.188942 -2.381430
H 0.616035 1.230245 -2.639569
C 2.107124 1.321137 0.483393
C 2.965539 1.942368 -0.425366
H 2.562269 2.499745 -1.267719
C 4.341862 1.845964 -0.256307
H 5.007647 2.335080 -0.964654
C 4.864552 1.122294 0.810101
H 5.942863 1.036103 0.932994
C 4.010099 0.508946 1.720736
H 4.416178 -0.063908 2.551681
C 2.633466 0.614030 1.569367
H 1.962766 0.110542 2.264537
C -0.427618 1.716112 1.781776
C 0.055624 2.810038 2.506927
H 0.906790 3.380583 2.135504
C -0.545087 3.158345 3.709268
H -0.172464 4.008901 4.276740
C -1.617376 2.410013 4.191085
H -2.084088 2.681633 5.136328
C -2.080828 1.310982 3.477606
H -2.906177 0.716640 3.864596
C -1.486940 0.954817 2.271286
H -1.820235 0.071971 1.726965
C 2.028094 -2.054761 -0.039003
C 2.633181 -1.551578 -1.183106
H 2.042254 -0.964832 -1.883663
C 3.989307 -1.761290 -1.385692
H 4.468717 -1.345725 -2.271306
C 4.754532 -2.481971 -0.464407
C 4.114925 -3.005733 0.660983
H 4.693039 -3.577505 1.386081
C 2.761153 -2.790607 0.883583
H 2.268933 -3.157357 1.781137
C 6.230704 -2.646841 -0.664567
H 6.637667 -3.441343 -0.030438
H 6.760891 -1.717265 -0.412997
H 6.472113 -2.882551 -1.707613
Sn -1.872123 -0.757217 -1.625801
H -2.642394 1.054654 -1.418400
H -2.382913 0.514618 -2.690879
C -3.513101 -1.421362 -0.360607
C -5.658208 -2.123832 -0.156705
C -4.192453 -2.112156 1.691630
C -5.458383 -2.389979 1.192594
H -6.627575 -2.303536 -0.627393
H -3.944822 -2.283878 2.741559
H -6.248916 -2.791159 1.822002
N -3.226645 -1.625746 0.921204
N -4.692478 -1.649483 -0.935113

^{Tos}YSnB2
Not determined.

^{Tos}YSnB1
E = -2577.38249734
S -1.684904 1.332499 -0.116416

P -0.384070 -1.320724 0.250229
O -1.464205 2.484761 -1.011373
O -1.611732 1.590574 1.340613
C -0.628188 0.112128 -0.646521
C 0.397762 -2.528198 -0.853451
C 1.563648 -3.221320 -0.532561
H 2.060572 -3.059798 0.419830
C 2.127585 -4.090444 -1.460680
H 3.043916 -4.618139 -1.207704
C 1.540396 -4.265074 -2.707522
H 1.990929 -4.940474 -3.432386
C 0.381865 -3.565032 -3.034756
H -0.073605 -3.687209 -4.015384
C -0.186892 -2.697271 -2.113763
H -1.072275 -2.118504 -2.372422
C -1.945218 -2.070962 0.816644
C -2.493343 -3.205223 0.214513
H -1.941049 -3.742650 -0.551901
C -3.753776 -3.653519 0.594006
H -4.174033 -4.539476 0.121936
C -4.474784 -2.970295 1.566574
H -5.467517 -3.314485 1.851747
C -3.923327 -1.849872 2.179743
H -4.484063 -1.310735 2.940361
C -2.658374 -1.405463 1.819589
H -2.240408 -0.512566 2.281730
C 0.597558 -1.193515 1.777622
C 0.848318 -2.323202 2.564409
H 0.497211 -3.302576 2.240778
C 1.527430 -2.194144 3.767579
H 1.727232 -3.074463 4.375439
C 1.929707 -0.933353 4.206349
H 2.447020 -0.830977 5.158726
C 1.657585 0.192364 3.438610
H 1.958455 1.179399 3.781488
C 0.997696 0.065825 2.218767
H 0.749924 0.943082 1.625825
C -3.374077 0.777573 -0.345553
C -3.704552 -0.189454 -1.284940
H -2.924576 -0.597152 -1.924975
C -5.010040 -0.654334 -1.355022
H -5.263038 -1.432736 -2.073998
C -6.001987 -0.153556 -0.507765
C -5.652362 0.848372 0.400249
H -6.414154 1.258858 1.062192
C -4.346317 1.309729 0.492698
H -4.058989 2.053299 1.232169
C -7.391298 -0.714410 -0.545579
H -8.112256 -0.039858 -0.071866
H -7.434165 -1.673519 -0.009762
H -7.722153 -0.904616 -1.573116
H 1.945940 -0.611061 -1.805032
H 1.437701 -0.172923 -3.034003
N 3.581526 0.973847 0.288386
C 3.194534 2.860083 1.436414
C 3.949484 1.740232 1.390006
H 3.246642 3.690961 2.130832
H 4.768374 1.445021 2.035463
N 2.317569 2.864955 0.362260
C 4.121602 -0.308144 0.082501

C 4.496794 -0.721930 -1.197062
C 4.304986 -1.173890 1.163224
C 5.056629 -1.978366 -1.387335
H 4.367428 -0.037777 -2.032048
C 4.877322 -2.425397 0.967444
H 3.979959 -0.865828 2.154131
C 5.258964 -2.831929 -0.306796
H 5.349193 -2.286837 -2.389126
H 5.018098 -3.087717 1.819794
H 5.713136 -3.809399 -0.458318
C 1.297345 3.835101 0.211750
C 1.154407 4.511234 -0.998211
C 0.405887 4.075363 1.254249
C 0.105785 5.404656 -1.169407
H 1.867145 4.323746 -1.798332
C -0.634115 4.977465 1.078619
H 0.500949 3.515662 2.182076
C -0.790135 5.639491 -0.132953
H -0.012071 5.919234 -2.120872
H -1.344161 5.138266 1.886980
H -1.617804 6.331414 -0.274261
B 2.512979 1.662326 -0.395629
Sn 0.924159 1.025791 -1.872543

$^{Ph}Y_2Sn$
E = -2620.48405265
P -3.155991 0.182471 -0.024132
P 3.095413 0.393376 0.082457
C -1.575618 -0.343686 0.279567
C -1.304954 -1.703920 0.762430
C -2.090682 -2.819202 0.419173
H -2.926506 -2.687847 -0.265592
C -1.803367 -4.089845 0.901980
H -2.434560 -4.928168 0.610373
C -0.707153 -4.299028 1.731263
H -0.471694 -5.296367 2.096840
C 0.085310 -3.210274 2.085049
H 0.940662 -3.351295 2.744734
C -0.213143 -1.938043 1.619720
H 0.406058 -1.092072 1.914276
C -3.957321 -0.333839 -1.593862
C -4.844069 -1.411333 -1.671177
H -5.206956 -1.884619 -0.760293
C -5.262906 -1.889199 -2.907802
H -5.956375 -2.726732 -2.955101
C -4.797238 -1.301865 -4.079491
H -5.124932 -1.678834 -5.046369
C -3.903818 -0.237294 -4.012392
H -3.527462 0.220013 -4.925158
C -3.480704 0.238929 -2.778432
H -2.779153 1.071624 -2.730611
C -3.221534 2.005573 -0.071264
C -4.190465 2.687907 -0.808464
H -4.875586 2.138030 -1.450779
C -4.278277 4.073864 -0.732531
H -5.031729 4.599598 -1.315751
C -3.406278 4.783836 0.085004
H -3.475625 5.868571 0.142663
C -2.449897 4.105383 0.834362
H -1.771685 4.658532 1.480786

C -2.359224 2.721672 0.762151
H -1.622260 2.174225 1.347570
C -4.335260 -0.276120 1.286802
C -3.866787 -0.767318 2.505780
H -2.802443 -0.939964 2.642046
C -4.762650 -1.031955 3.535694
H -4.393534 -1.426144 4.480385
C -6.121668 -0.793468 3.361974
H -6.819225 -1.000883 4.171418
C -6.589576 -0.281994 2.155151
H -7.650688 -0.082081 2.019618
C -5.699423 -0.022378 1.120808
H -6.065902 0.382652 0.178140
C 1.825966 -0.462416 -0.660864
C 2.026277 -1.851379 -1.080196
C 0.959762 -2.653856 -1.534584
H -0.033426 -2.217508 -1.604519
C 1.135061 -3.982149 -1.887083
H 0.268984 -4.556489 -2.213108
C 2.390341 -4.581895 -1.825515
H 2.526139 -5.624843 -2.104288
C 3.466204 -3.812178 -1.400627
H 4.463821 -4.246555 -1.346989
C 3.290498 -2.482675 -1.038166
H 4.167343 -1.929986 -0.703518
C 4.583542 0.604107 -0.970441
C 4.365770 0.557589 -2.349478
H 3.357341 0.359142 -2.712963
C 5.425995 0.736794 -3.230292
H 5.250347 0.693424 -4.303368
C 6.709317 0.954386 -2.739838
H 7.540704 1.089534 -3.429254
C 6.933317 0.983542 -1.366807
H 7.938973 1.139564 -0.981002
C 5.875834 0.805196 -0.482205
H 6.067085 0.808072 0.587611
C 2.547255 2.071715 0.498917
C 1.747096 2.250225 1.633044
H 1.545240 1.406151 2.292047
C 1.207645 3.499318 1.910746
H 0.580918 3.633744 2.790532
C 1.463545 4.571989 1.060781
H 1.034458 5.549209 1.274945
C 2.269537 4.399409 -0.058617
H 2.471552 5.238943 -0.720453
C 2.815444 3.151454 -0.340301
H 3.437238 3.011864 -1.222247
C 3.697349 -0.246117 1.689062
C 4.431195 0.567552 2.558783
H 4.677706 1.589141 2.271265
C 4.819398 0.090969 3.805154
H 5.385502 0.732204 4.478113
C 4.472013 -1.199079 4.194814
H 4.774637 -1.572273 5.171446
C 3.724244 -2.004325 3.342133
H 3.438708 -3.008897 3.649018
C 3.329480 -1.530218 2.095771
H 2.722350 -2.148529 1.439022
Sn -0.011435 0.611910 -0.848669
H -0.366945 1.153913 -2.460001

H -0.430960 -0.177427 -2.667010

^{Ph}YSnCl
E = -1773.09232286
P -0.834506 -0.160387 0.024796
C 0.726052 0.492420 -0.219892
C 0.970627 1.929460 -0.090303
C -0.045145 2.832786 0.296958
H -1.037879 2.465965 0.552100
C 0.175285 4.201405 0.365612
H -0.645282 4.854706 0.658770
C 1.424865 4.735501 0.073508
H 1.599850 5.807551 0.133880
C 2.450683 3.864303 -0.279172
H 3.445679 4.250857 -0.494032
C 2.233025 2.497098 -0.356191
H 3.066949 1.846197 -0.607997
C -1.482749 -0.142007 1.730875
C -2.612694 -0.876301 2.107299
H -3.149036 -1.472364 1.369599
C -3.033970 -0.875502 3.431002
H -3.909648 -1.452606 3.721539
C -2.327737 -0.147615 4.385197
H -2.658282 -0.149147 5.422142
C -1.191519 0.565255 4.018995
H -0.628443 1.119787 4.766696
C -0.761802 0.562602 2.696120
H 0.142553 1.094553 2.404703
C -0.851262 -1.913086 -0.437545
C -0.533617 -2.886528 0.513863
H -0.371717 -2.601657 1.551804
C -0.414967 -4.216999 0.130306
H -0.159936 -4.971743 0.871084
C -0.612765 -4.578959 -1.198001
H -0.515688 -5.620818 -1.496920
C -0.935840 -3.612446 -2.145512
H -1.094227 -3.896444 -3.183723
C -1.055883 -2.280387 -1.768880
H -1.304510 -1.522826 -2.509665
C -2.068680 0.672071 -1.034642
C -3.418600 0.796238 -0.703408
H -3.790856 0.424517 0.247240
C -4.294695 1.420985 -1.583807
H -5.346066 1.516676 -1.320042
C -3.828573 1.929660 -2.791547
H -4.516778 2.422253 -3.475793
C -2.480345 1.822626 -3.116968
H -2.108113 2.237672 -4.051333
C -1.599857 1.200400 -2.240885
H -0.536334 1.135221 -2.467811
Sn 2.268057 -0.916748 -0.499435
H 3.075204 0.031849 -2.050784
H 2.895359 -1.374038 -2.028466
Cl 4.122524 -0.344754 0.934643

^{Ph}YSnHMDS
E = -2186.88399426
P -1.907377 -0.121157 -0.042741
C -0.357821 0.513920 -0.357199
C -0.041719 1.908491 -0.058614

C -0.884091 2.731078 0.723612
H -1.808634 2.329337 1.135746
C -0.572961 4.056381 0.996773
H -1.260493 4.646416 1.601211
C 0.601544 4.626206 0.518657
H 0.850483 5.661114 0.742961
C 1.451875 3.837401 -0.250746
H 2.379938 4.253890 -0.640920
C 1.138528 2.517102 -0.534533
H 1.825678 1.929796 -1.137608
C -2.365021 -0.454502 1.697503
C -3.466068 -1.246916 2.039975
H -4.090595 -1.680862 1.259632
C -3.749776 -1.506840 3.375078
H -4.603768 -2.128739 3.636435
C -2.935728 -0.981294 4.375203
H -3.158736 -1.187697 5.420304
C -1.830006 -0.208283 4.039208
H -1.181626 0.189785 4.817019
C -1.539816 0.048847 2.703856
H -0.655482 0.621572 2.430973
C -2.082473 -1.732001 -0.858255
C -1.736144 -2.906438 -0.185287
H -1.458959 -2.872217 0.866600
C -1.730884 -4.119042 -0.865302
H -1.450722 -5.030053 -0.340716
C -2.074115 -4.164339 -2.211857
H -2.066307 -5.114212 -2.742909
C -2.428667 -2.997066 -2.881860
H -2.701077 -3.032430 -3.934594
C -2.432829 -1.781886 -2.208804
H -2.701962 -0.867268 -2.733785
C -3.215809 0.962696 -0.717832
C -4.501601 1.056495 -0.183708
H -4.770357 0.497895 0.708822
C -5.443971 1.885586 -0.781935
H -6.444903 1.955353 -0.360629
C -5.106982 2.629229 -1.908020
H -5.846075 3.280605 -2.370652
C -3.821217 2.551300 -2.433168
H -3.547708 3.146379 -3.302069
C -2.875431 1.724994 -1.839341
H -1.857144 1.674659 -2.223082
Sn 1.108776 -0.825094 -1.114805
H 1.468673 0.303501 -2.727693
H 1.309156 -1.069120 -2.804964
Si 2.743217 -0.656453 1.641358
Si 4.370015 0.206302 -0.786811
C 1.418481 -1.958112 1.985786
H 0.412617 -1.617768 1.711105
H 1.628082 -2.893951 1.450003
H 1.398575 -2.183137 3.060891
C 4.329611 -1.330153 2.398547
H 5.185290 -0.655040 2.280082
H 4.176006 -1.478711 3.476007
H 4.598407 -2.298667 1.958803
C 2.282478 0.913976 2.564356
H 1.407220 1.396998 2.114785
H 2.063386 0.698891 3.619491
H 3.099275 1.645234 2.533170

C 4.174179 0.801227 -2.558422
H 3.869484 -0.000020 -3.242847
H 3.458222 1.623744 -2.669079
H 5.157547 1.162322 -2.891445
C 5.702242 -1.123738 -0.851420
H 6.605099 -0.741454 -1.346705
H 5.992122 -1.483234 0.141628
H 5.344452 -1.987724 -1.427064
C 4.993258 1.673489 0.217024
H 5.251576 1.402825 1.248047
H 5.889357 2.107873 -0.245388
H 4.217611 2.449566 0.262024
N 2.886933 -0.406877 -0.087363

$^{Ph}YSnC_6F_5$
E = -2041.43162396
P -1.896213 0.004149 0.129103
C -0.493290 0.565363 -0.667820
C 0.090298 1.865328 -0.325574
C 1.171536 2.408145 -1.050676
H 1.591860 1.841289 -1.878728
C 1.714972 3.647187 -0.746053
H 2.551051 4.015054 -1.339165
C 1.207229 4.414450 0.297336
H 1.636114 5.385048 0.536981
C 0.142510 3.905654 1.032650
H -0.274723 4.478512 1.859628
C -0.396526 2.661135 0.736997
H -1.215644 2.301938 1.357976
C -3.241173 1.234773 0.037371
C -3.235061 2.091686 -1.066857
H -2.422003 2.016969 -1.787778
C -4.240728 3.038150 -1.218851
H -4.226477 3.707224 -2.076791
C -5.252152 3.139615 -0.269278
H -6.037056 3.884356 -0.386707
C -5.252544 2.298391 0.838440
H -6.034955 2.384702 1.589773
C -4.248495 1.349905 0.996376
H -4.246406 0.715320 1.878388
C -2.502580 -1.504915 -0.673086
C -2.051533 -2.755871 -0.241765
C -2.427877 -3.903077 -0.930360
C -3.252596 -3.806511 -2.045922
C -3.704356 -2.562316 -2.475728
C -3.330700 -1.411465 -1.792529
C -1.717393 -0.478113 1.883646
C -2.693832 -1.210656 2.567705
H -3.622026 -1.486011 2.067909
C -2.466295 -1.623429 3.874435
H -3.225608 -2.198264 4.401084
C -1.260906 -1.317496 4.501534
H -1.081460 -1.649217 5.522450
C -0.280305 -0.606527 3.819408
H 0.672211 -0.387192 4.297120
C -0.505285 -0.190560 2.511826
H 0.266192 0.343337 1.960006
Sn 0.645212 -0.914523 -1.664194
H 0.735777 -1.120264 -3.368305
H 1.204363 0.166131 -3.208418

C 2.620939 -0.685804 -0.739141
C 3.801598 -0.919927 1.380661
C 4.826507 0.285410 -0.433391
C 4.871667 -0.195609 0.869247
H -1.398320 -2.829572 0.626281
H -2.069209 -4.874480 -0.596830
H -3.545209 -4.705847 -2.584408
H -4.350893 -2.487073 -3.347550
H -3.680521 -0.437427 -2.129331
C 3.705508 0.032350 -1.217251
C 2.706181 -1.155055 0.562594
F 1.688352 -1.851142 1.085363
F 3.706764 0.534021 -2.447685
F 5.847619 0.979193 -0.909874
F 5.931725 0.036237 1.622631
F 3.839278 -1.371853 2.625558

^{Ph}YSnPy
E = -1560.57715178
P -1.353297 -0.146911 0.090360
C 0.177345 0.393314 -0.426432
C 0.493184 1.823228 -0.409415
C 1.576350 2.347429 -1.146181
H 2.196263 1.662946 -1.723466
C 1.868302 3.702950 -1.159411
H 2.716869 4.054368 -1.744858
C 1.092806 4.609969 -0.442405
H 1.323658 5.673114 -0.454546
C 0.019558 4.123353 0.295391
H -0.603537 4.806720 0.870871
C -0.267428 2.765248 0.320505
H -1.107449 2.431869 0.928278
C -2.686240 0.876749 -0.628389
C -2.433014 1.431659 -1.886820
H -1.461566 1.266936 -2.350806
C -3.400131 2.204640 -2.516110
H -3.192596 2.638202 -3.492259
C -4.621332 2.437412 -1.892122
H -5.376980 3.047882 -2.382932
C -4.870754 1.901524 -0.633268
H -5.819578 2.093308 -0.136051
C -3.906249 1.125894 0.000902
H -4.105146 0.733062 0.994252
C -1.639182 -1.852015 -0.463585
C -1.321489 -2.933294 0.361545
H -0.987483 -2.761090 1.382633
C -1.419139 -4.231661 -0.125896
H -1.161181 -5.070232 0.517450
C -1.833852 -4.455418 -1.433629
H -1.906112 -5.472243 -1.814676
C -2.155841 -3.380285 -2.256940
H -2.482470 -3.554051 -3.280118
C -2.058295 -2.081252 -1.775650
H -2.302741 -1.240358 -2.421776
C -1.665314 -0.217477 1.894478
C -2.767859 -0.876084 2.449008
H -3.495945 -1.362838 1.800737
C -2.921625 -0.938304 3.828442
H -3.777700 -1.456923 4.255798
C -1.975046 -0.347877 4.662086

H -2.096547 -0.399159 5.742458
C -0.868064 0.292355 4.116360
H -0.120360 0.741641 4.766760
C -0.708288 0.350491 2.735797
H 0.167111 0.825800 2.293382
Sn 1.637804 -1.107144 -0.820893
H 1.810521 -1.755047 -2.420473
H 2.192017 -0.468426 -2.605624
C 3.566261 -0.463866 -0.043043
N 4.636035 -1.136224 -0.464835
C 4.903725 0.917899 1.391183
C 5.828714 -0.794821 0.010620
C 6.019503 0.224100 0.938893
H 5.005275 1.723677 2.116948
H 6.682563 -1.364465 -0.363350
H 7.018936 0.462379 1.296865
C 3.654055 0.570123 0.893630
H 2.757197 1.101451 1.210352

^{Ph}YSnPyr
E = -1576.63642433
P -1.329780 -0.064770 0.087594
C 0.128022 0.312257 -0.707877
C 0.649749 1.682008 -0.680686
C 1.708215 2.075752 -1.524494
H 2.152580 1.331672 -2.184055
C 2.194066 3.373657 -1.537402
H 3.013038 3.626122 -2.209841
C 1.648017 4.349270 -0.707284
H 2.030918 5.367756 -0.717078
C 0.608593 3.988678 0.142315
H 0.167983 4.726739 0.811369
C 0.128441 2.685934 0.164228
H -0.667170 2.446153 0.867845
C -2.638894 1.126465 -0.366430
C -2.555203 1.697826 -1.639978
H -1.707623 1.445973 -2.275940
C -3.526677 2.593929 -2.067607
H -3.450390 3.039341 -3.057461
C -4.582603 2.932328 -1.227629
H -5.341071 3.637633 -1.562108
C -4.659904 2.379660 0.045940
H -5.476009 2.653144 0.711854
C -3.689895 1.482467 0.479165
H -3.749073 1.078859 1.485771
C -1.911530 -1.707392 -0.427233
C -1.687437 -2.840530 0.357093
H -1.221628 -2.744061 1.335405
C -2.047543 -4.097240 -0.117921
H -1.862887 -4.977819 0.493852
C -2.631484 -4.227514 -1.372427
H -2.911012 -5.212030 -1.742662
C -2.856683 -3.099795 -2.156971
H -3.313166 -3.199689 -3.139570
C -2.496510 -1.843496 -1.688310
H -2.665084 -0.962360 -2.304373
C -1.287550 -0.150494 1.916178
C -2.343788 -0.641769 2.691422
H -3.258256 -0.990054 2.212170
C -2.217107 -0.720592 4.072473

H -3.038832 -1.108163 4.671765
C -1.034185 -0.315201 4.686175
H -0.936083 -0.378313 5.768429
C 0.025911 0.150994 3.917191
H 0.957920 0.448863 4.393379
C -0.092627 0.227853 2.532608
H 0.750935 0.555082 1.921181
Sn 1.450082 -1.326568 -0.977976
H 1.668394 -2.111765 -2.507696
H 2.129157 -0.862138 -2.764110
C 3.353964 -0.678097 -0.132547
N 3.235767 0.181711 0.882056
N 4.507271 -1.152891 -0.603489
C 4.356206 0.635973 1.431327
C 5.621655 -0.698878 -0.043684
C 5.608357 0.229417 0.991120
H 4.249515 1.348687 2.252139
H 6.564360 -1.089580 -0.434430
H 6.525360 0.607334 1.436530

^{Ph}YSnB2
E = -1719.59998698
P -1.917834 -0.074659 0.105313
C -0.349167 0.310031 -0.434210
C 0.084285 1.707922 -0.489607
C 1.199770 2.102724 -1.257093
H 1.752603 1.343133 -1.805877
C 1.600416 3.427128 -1.346503
H 2.470074 3.676583 -1.953042
C 0.904063 4.431577 -0.680456
H 1.219464 5.470428 -0.751769
C -0.202148 4.074576 0.082306
H -0.766300 4.836950 0.617995
C -0.596464 2.747549 0.183713
H -1.458603 2.517693 0.808167
C -3.167033 1.015958 -0.663930
C -2.878140 1.496914 -1.944670
H -1.922735 1.242271 -2.401253
C -3.789339 2.310409 -2.605969
H -3.554024 2.686453 -3.599537
C -4.989528 2.656137 -1.992999
H -5.700717 3.297593 -2.510056
C -5.273264 2.193323 -0.712948
H -6.204664 2.473082 -0.224575
C -4.364462 1.378857 -0.046396
H -4.588042 1.044520 0.962818
C -2.343278 -1.776514 -0.366121
C -2.129230 -2.839446 0.514342
C -2.335265 -4.147252 0.089178
C -2.755642 -4.398720 -1.211471
C -2.973854 -3.341512 -2.090455
C -2.767313 -2.033810 -1.671375
C -2.237624 -0.030996 1.909948
C -3.386393 -0.576871 2.492769
H -4.146475 -1.044026 1.867180
C -3.548156 -0.551628 3.872426
H -4.440930 -0.982406 4.321788
C -2.563765 0.014709 4.678610
H -2.692004 0.033087 5.759290
C -1.411865 0.541949 4.105759

H -0.635517 0.972198 4.735174
C -1.243737 0.511810 2.725075
H -0.334530 0.898281 2.264747
Sn 0.998115 -1.326878 -0.666894
H 0.958910 -2.127470 -2.224209
H 1.608971 -0.977437 -2.495927
H -1.792308 -2.647084 1.530810
H -2.158623 -4.972006 0.776432
H -2.913791 -5.422992 -1.543537
H -3.304521 -3.536606 -3.108491
H -2.929852 -1.207858 -2.361099
C 5.324237 -0.743874 -0.216435
C 4.853589 0.320832 0.546036
C 6.673805 -0.993727 -0.367768
C 5.707295 1.192027 1.192632
C 7.547016 -0.119940 0.283837
H 7.028156 -1.828755 -0.965968
C 7.074728 0.948885 1.046024
H 5.323719 2.021898 1.779814
H 8.619760 -0.276588 0.192978
H 7.786756 1.609221 1.536455
B 3.132147 -0.751945 -0.288593
O 3.490254 0.316093 0.514407
O 4.259126 -1.422446 -0.732630

^{Ph}YSnB1
E = -1988.78927655
P 2.047609 -0.741316 0.105243
C 0.486630 -0.143098 0.395585
C 0.249791 0.827362 1.461687
C 1.249877 1.706797 1.934406
H 2.236576 1.694166 1.472700
C 1.014991 2.604166 2.966624
H 1.821977 3.258056 3.295196
C -0.237067 2.684123 3.569899
H -0.423920 3.395716 4.371670
C -1.241197 1.824303 3.129818
H -2.226646 1.852019 3.593122
C -1.001876 0.913800 2.110431
H -1.794896 0.230335 1.812489
C 3.204859 0.236793 -0.923758
C 4.399939 -0.286422 -1.429793
H 4.681706 -1.315525 -1.207872
C 5.217310 0.495843 -2.235298
H 6.144122 0.084694 -2.631095
C 4.844089 1.802420 -2.542662
H 5.484604 2.414687 -3.174901
C 3.649784 2.319666 -2.054962
H 3.349637 3.334489 -2.304051
C 2.825017 1.537102 -1.252267
H 1.872029 1.920571 -0.885188
C 1.927055 -2.327236 -0.776673
C 2.037556 -2.390847 -2.166847
H 2.311767 -1.503737 -2.733555
C 1.777357 -3.584233 -2.832086
H 1.855141 -3.625133 -3.916611
C 1.406371 -4.715212 -2.115136
H 1.198298 -5.647023 -2.637603
C 1.298402 -4.656554 -0.728164
H 1.008282 -5.540858 -0.164472

C 1.555215 -3.466942 -0.060209
H 1.455525 -3.417721 1.022303
C 2.920453 -1.048742 1.681756
C 4.296405 -0.891622 1.851980
H 4.917042 -0.546824 1.029522
C 4.881169 -1.153738 3.086352
H 5.954766 -1.026440 3.211205
C 4.097675 -1.566178 4.158207
H 4.557868 -1.765342 5.124260
C 2.723062 -1.709588 3.997895
H 2.102532 -2.013001 4.838639
C 2.134223 -1.447988 2.767909
H 1.055540 -1.524928 2.640417
Sn -1.078119 -0.694423 -0.962701
N -4.089443 0.597745 -0.123902
C -3.621759 2.782710 0.027702
C -4.562023 1.846256 0.274183
H -3.676104 3.856062 0.168323
H -5.569095 1.985170 0.648387
B -2.749669 0.768435 -0.623415
N -2.508866 2.180332 -0.549436
C -1.361929 2.918759 -0.906067
C -0.757708 3.774017 0.015711
C -0.850380 2.826702 -2.202059
C 0.319420 4.560965 -0.372107
H -1.120205 3.794845 1.041572
C 0.229401 3.614504 -2.582251
H -1.332504 2.159950 -2.914192
C 0.805757 4.497661 -1.673930
H 0.785550 5.222232 0.355609
H 0.610935 3.547649 -3.599498
H 1.636962 5.131580 -1.977555
C -4.814182 -0.590934 0.050478
C -4.654671 -1.638775 -0.859962
C -5.690816 -0.753526 1.125613
C -5.351260 -2.826976 -0.691551
H -3.988287 -1.505968 -1.710951
C -6.395660 -1.940937 1.279412
H -5.799572 0.043510 1.857381
C -6.229701 -2.984481 0.375400
H -5.212612 -3.633374 -1.409246
H -7.072283 -2.053784 2.124564
H -6.780405 -3.914105 0.502205
H -2.056749 -1.865243 0.275749
H -1.548723 -2.374054 -0.863267

$^{F}Y_2Sn$
E = -3614.29083094
P -3.320396 -0.528397 0.049491
P 2.970994 -1.137277 -0.034846
C -1.716310 -0.121163 -0.319378
C -1.292363 1.175814 -0.820088
C -1.834019 2.401561 -0.407828
C -1.360747 3.629560 -0.840300
C -0.297078 3.678750 -1.732440
C 0.247574 2.490014 -2.199700
C -0.249366 1.273320 -1.756055
C -3.907658 -0.276193 1.763086
C -4.581472 0.877681 2.175506
H -4.885084 1.623253 1.445530

C -4.856488 1.082943 3.521115
H -5.386372 1.981924 3.829601
C -4.450923 0.150567 4.471379
H -4.665309 0.317789 5.525165
C -3.761505 -0.988118 4.072814
H -3.427587 -1.714199 4.810954
C -3.487093 -1.198776 2.727212
H -2.950945 -2.096927 2.422255
C -3.565112 -2.320674 -0.222844
C -4.644106 -2.991122 0.357918
H -5.302629 -2.467862 1.049117
C -4.874698 -4.329654 0.065003
H -5.715010 -4.846555 0.524134
C -4.031349 -5.006155 -0.810874
H -4.210264 -6.055859 -1.036434
C -2.964798 -4.338020 -1.402571
H -2.307983 -4.862953 -2.093479
C -2.734488 -2.997146 -1.117240
H -1.911243 -2.457685 -1.582687
C -4.519512 0.265131 -1.061460
C -4.088306 0.699271 -2.316882
H -3.041446 0.596164 -2.590278
C -4.998437 1.243105 -3.215396
H -4.653958 1.587998 -4.188194
C -6.343406 1.339817 -2.875917
H -7.054149 1.766693 -3.580923
C -6.782143 0.879211 -1.638684
H -7.836341 0.938258 -1.375595
C -5.875690 0.340036 -0.734195
H -6.227494 -0.026090 0.228271
C 1.681783 -0.181652 0.585675
C 1.857373 1.212883 0.953145
C 0.829510 2.010955 1.503225
C 0.939858 3.374359 1.728753
C 2.134918 4.039708 1.499025
C 3.203335 3.294264 1.024887
C 3.058090 1.935619 0.794494
C 4.332218 -1.370770 1.159953
C 3.970124 -1.396351 2.509110
H 2.924242 -1.251323 2.779676
C 4.939592 -1.579170 3.488391
H 4.650959 -1.597627 4.537458
C 6.275295 -1.719845 3.127319
H 7.036024 -1.853875 3.894028
C 6.640983 -1.670447 1.785822
H 7.687834 -1.759696 1.502265
C 5.674963 -1.494775 0.802241
H 5.974955 -1.431539 -0.240357
C 2.322767 -2.798579 -0.388464
C 1.601401 -2.980314 -1.575389
H 1.532528 -2.168005 -2.297429
C 0.955564 -4.185490 -1.814782
H 0.387318 -4.319899 -2.733313
C 1.027837 -5.212514 -0.877649
H 0.510504 -6.152542 -1.060200
C 1.767676 -5.043227 0.287160
H 1.833647 -5.850456 1.013584
C 2.420274 -3.839567 0.532588
H 2.986552 -3.702696 1.451359
C 3.702570 -0.700797 -1.648334

C 4.496512 -1.621978 -2.338888
H 4.716668 -2.593170 -1.896918
C 4.985236 -1.310748 -3.601102
H 5.605863 -2.028597 -4.133873
C 4.667313 -0.088557 -4.187710
H 5.044467 0.151566 -5.180047
C 3.855947 0.817853 -3.514756
H 3.583963 1.764750 -3.974623
C 3.374552 0.512371 -2.247543
H 2.727293 1.211084 -1.725722
Sn -0.184413 -1.258499 0.665829
H -0.501118 -2.164220 2.092920
H -0.562098 -0.932340 2.676575
F -2.829020 2.401932 0.475153
F -1.894593 4.753143 -0.390147
F 0.173315 4.841360 -2.155922
F -0.111695 4.040683 2.183478
F 1.225520 2.534429 -3.100141
F -0.347355 1.475279 1.814352
F 0.277059 0.161068 -2.273601
F 2.251432 5.340612 1.722406
F 4.150641 1.301432 0.341120
F 4.373664 3.875254 0.803295

^{F}YSnCl
E = -2269.99998836
P 0.764757 0.763860 0.243915
C -0.202118 -0.626215 0.027531
C -1.631824 -0.556312 -0.191283
C -2.280662 0.489654 -0.863217
C -3.658213 0.557320 -1.008623
C -4.460442 -0.449172 -0.487538
C -3.862147 -1.513501 0.177664
C -2.486374 -1.541840 0.327585
C 1.549609 1.504971 -1.220080
C 2.525440 2.498813 -1.090353
H 2.814366 2.857061 -0.102291
C 3.139937 3.016982 -2.222130
H 3.897920 3.791353 -2.122425
C 2.793397 2.534398 -3.482398
H 3.281068 2.936260 -4.368557
C 1.839503 1.531715 -3.610198
H 1.583890 1.141476 -4.592868
C 1.217909 1.013163 -2.479764
H 0.488530 0.209630 -2.560694
C 2.181521 0.333090 1.301871
C 3.365266 -0.130334 0.718710
H 3.468325 -0.166405 -0.363843
C 4.407055 -0.575357 1.526893
H 5.318950 -0.949131 1.066310
C 4.281148 -0.543761 2.910056
H 5.099828 -0.887442 3.539344
C 3.107340 -0.072327 3.492633
H 3.008567 -0.041809 4.575744
C 2.057375 0.358883 2.693613
H 1.141492 0.722366 3.153868
C -0.219651 2.045864 1.073667
C -0.174648 3.391110 0.707004
H 0.468656 3.716812 -0.106080
C -0.976972 4.315594 1.365170

H -0.941020 5.363049 1.073133
C -1.832364 3.903601 2.381494
H -2.463146 4.630551 2.889376
C -1.894180 2.560280 2.737337
H -2.575426 2.231274 3.519279
C -1.096290 1.631034 2.082063
H -1.158406 0.573104 2.332820
Sn 0.835080 -2.470798 -0.029277
H -0.575058 -3.336799 -1.103572
H 0.106322 -4.017757 -0.059711
Cl 2.389313 -2.305695 -1.896954
F -1.953674 -2.550171 1.025751
F -4.612758 -2.473849 0.693896
F -5.776037 -0.401846 -0.629994
F -4.209770 1.567982 -1.662165
F -1.552897 1.462251 -1.418899

^{F}YSnHMDS
E = -2683.78422834
P -2.082632 -0.338051 -0.140182
C -0.423931 -0.013291 -0.333538
C 0.099501 1.336799 -0.267319
C -0.325501 2.286506 0.672483
C 0.170229 3.577551 0.739401
C 1.170607 3.972266 -0.139186
C 1.634701 3.063534 -1.082774
C 1.103657 1.784334 -1.139182
C -2.754141 -0.691676 1.522323
C -4.127865 -0.792943 1.762420
H -4.838220 -0.625932 0.953044
C -4.589489 -1.096229 3.037303
H -5.659182 -1.171251 3.222875
C -3.684284 -1.298393 4.076384
H -4.048428 -1.534796 5.074377
C -2.318070 -1.188048 3.842881
H -1.608709 -1.333717 4.654954
C -1.854896 -0.880772 2.568593
H -0.789662 -0.763431 2.375103
C -2.448459 -1.828214 -1.128355
C -3.280634 -2.860252 -0.687647
H -3.742403 -2.812213 0.294569
C -3.498516 -3.971248 -1.493353
H -4.139109 -4.775571 -1.137459
C -2.890564 -4.063496 -2.741062
H -3.059643 -4.938705 -3.365447
C -2.057357 -3.041909 -3.184296
H -1.566092 -3.113741 -4.152412
C -1.834514 -1.928977 -2.383541
H -1.147746 -1.147680 -2.708424
C -3.093480 1.038280 -0.775367
C -3.697957 1.957013 0.085816
H -3.611777 1.834015 1.162012
C -4.388907 3.046341 -0.433029
H -4.851161 3.761131 0.244630
C -4.482916 3.225056 -1.808093
H -5.024576 4.078694 -2.210889
C -3.878673 2.314314 -2.669500
H -3.946489 2.451458 -3.746674
C -3.183344 1.227610 -2.156876
H -2.709618 0.523168 -2.836026

Sn 0.867696 -1.719285 -0.301073
H 1.143996 -1.944667 -2.284033
H 0.788577 -2.973413 -1.468864
Si 2.908322 -0.787238 1.962673
Si 4.146411 -0.840956 -0.844434
C 2.038882 -2.146452 2.941036
H 0.967038 -2.241302 2.723257
H 2.500007 -3.121548 2.736911
H 2.135679 -1.944861 4.016805
C 4.676669 -0.757926 2.596978
H 5.324732 -0.047434 2.071877
H 4.646791 -0.459894 3.653697
H 5.140231 -1.749543 2.542311
C 2.106367 0.853429 2.411812
H 1.011803 0.794352 2.362484
H 2.375537 1.159764 3.431257
H 2.425434 1.643256 1.717626
C 3.787055 -1.239530 -2.641355
H 3.537479 -2.297845 -2.787775
H 2.982966 -0.636126 -3.071082
H 4.709455 -1.035975 -3.204192
C 5.587554 -1.974791 -0.397561
H 6.312261 -1.979229 -1.223081
H 6.123809 -1.675383 0.507944
H 5.235395 -3.005521 -0.258756
C 4.715074 0.949399 -0.737434
H 4.818074 1.289156 0.300792
H 5.687986 1.083272 -1.228819
H 3.999768 1.613429 -1.234364
N 2.782657 -1.101499 0.238938
F 1.562145 0.967577 -2.081595
F 2.565711 3.437352 -1.949415
F 1.657409 5.202522 -0.096806
F -0.272196 4.419370 1.661790
F -1.199197 1.916359 1.616304

$^{F}YSnC_6F_5$
E = -2538.32491531
P -1.584799 0.727594 0.135091
C -0.042179 0.605239 -0.564724
C 0.747488 1.807471 -0.856014
C 1.825101 1.766278 -1.762201
H 2.071391 0.821294 -2.244028
C 2.569046 2.896920 -2.066729
H 3.393551 2.814591 -2.773313
C 2.268190 4.124943 -1.486164
H 2.854086 5.010374 -1.723229
C 1.207547 4.195067 -0.589937
H 0.952894 5.142223 -0.116988
C 0.472736 3.061603 -0.271481
H -0.335557 3.159721 0.451325
C -2.558164 2.038625 -0.676799
C -2.271163 2.306686 -2.018565
H -1.513187 1.715311 -2.528001
C -2.941957 3.323138 -2.681317
H -2.709461 3.531566 -3.723411
C -3.902201 4.078236 -2.013371
H -4.426424 4.876564 -2.535262
C -4.183740 3.820205 -0.677425
H -4.925152 4.415738 -0.148800

C -3.508553 2.807011 -0.004480
H -3.714908 2.636901 1.048086
C -2.452403 -0.879426 -0.033741
C -2.262498 -1.895178 0.907406
C -2.878552 -3.133005 0.789985
C -3.694498 -3.388915 -0.304960
C -3.890996 -2.406729 -1.266837
C -3.259650 -1.176638 -1.132238
C -1.741848 1.050965 1.929315
C -2.938044 0.867680 2.628919
H -3.835198 0.534444 2.106727
C -2.981861 1.090703 3.998675
H -3.912653 0.943812 4.543038
C -1.832117 1.491310 4.675350
H -1.867893 1.663293 5.749353
C -0.637016 1.654200 3.984536
H 0.266370 1.948408 4.514657
C -0.586291 1.425660 2.613197
H 0.354376 1.512702 2.071366
Sn 0.902710 -1.283805 -0.521065
H 0.740550 -2.449119 -1.778385
H 1.377642 -1.406963 -2.429979
C 3.015748 -0.964168 -0.106602
C 4.760571 0.295349 1.024668
C 5.359929 -1.459931 -0.512199
C 5.731861 -0.454375 0.372985
F -1.453270 -1.721956 1.939522
F -2.672504 -4.071843 1.692343
F -4.278601 -4.560118 -0.431629
F -4.659749 -2.651444 -2.310249
F -3.454721 -0.295488 -2.095197
C 4.009577 -1.699584 -0.732616
C 3.421002 0.022914 0.777318
F 3.683093 -2.665715 -1.587205
F 2.517816 0.752178 1.432799
F 5.118528 1.245204 1.872688
F 7.011161 -0.216959 0.600259
F 6.289991 -2.172531 -1.126667

^{F}YSnPy
E = -2057.46965553
P -0.895369 0.748051 0.226111
C 0.677723 0.632951 -0.401390
C 1.495620 1.844742 -0.533702
C 2.618040 1.883049 -1.386173
H 2.885194 0.985869 -1.942428
C 3.384652 3.028661 -1.540055
H 4.242464 3.003519 -2.210546
C 3.067590 4.198925 -0.856761
H 3.669909 5.096574 -0.979352
C 1.966352 4.191831 -0.008270
H 1.694520 5.090489 0.543614
C 1.206511 3.042131 0.158305
H 0.364061 3.083864 0.846909
C -1.804095 2.162923 -0.487194
C -1.438025 2.554685 -1.778039
H -0.659982 2.000645 -2.299676
C -2.054286 3.644556 -2.373709
H -1.760046 3.948728 -3.375927
C -3.038367 4.351381 -1.687920

H -3.519961 5.207698 -2.156290
C -3.398144 3.970623 -0.400863
H -4.158803 4.527527 0.142503
C -2.778113 2.882368 0.204783
H -3.046548 2.614543 1.222585
C -1.822084 -0.794499 -0.118386
C -1.650446 -1.908040 0.708078
C -2.306868 -3.107218 0.475893
C -3.147358 -3.221751 -0.624464
C -3.328531 -2.138307 -1.473823
C -2.657555 -0.947251 -1.224160
C -1.128990 0.901166 2.038874
C -2.361935 0.678599 2.657558
H -3.237367 0.423580 2.060133
C -2.472353 0.760442 4.039739
H -3.432977 0.582047 4.518991
C -1.351848 1.059415 4.810388
H -1.438996 1.121014 5.893407
C -0.119569 1.261490 4.199204
H 0.760318 1.477294 4.801808
C -0.004461 1.172531 2.816026
H 0.962300 1.293527 2.328264
Sn 1.465127 -1.316001 -0.756997
H 1.198635 -2.060649 -2.301483
H 2.018067 -1.025819 -2.617499
C 3.555113 -1.417798 -0.179388
N 4.237619 -2.471188 -0.622271
C 5.459256 -0.580362 1.010453
C 5.514702 -2.590432 -0.275735
C 6.174139 -1.673887 0.537694
H 5.935278 0.162294 1.649179
H 6.044003 -3.464175 -0.662150
H 7.221587 -1.819445 0.793210
F -0.818663 -1.863136 1.736140
F -2.116944 -4.141614 1.271235
F -3.771406 -4.355161 -0.861489
F -4.123289 -2.248613 -2.521285
F -2.842356 0.035645 -2.085808
C 4.124785 -0.446321 0.647315
H 3.534354 0.404755 0.983987

^{F}YSnPyr
E = -2073.52863446
P -0.743125 0.796386 0.234903
C 0.646447 0.485781 -0.687773
C 1.691154 1.515168 -0.792833
C 2.710987 1.419735 -1.759596
H 2.724120 0.554870 -2.421072
C 3.692281 2.390311 -1.890776
H 4.458753 2.268765 -2.655016
C 3.704333 3.509529 -1.062993
H 4.475909 4.269886 -1.165182
C 2.712979 3.628594 -0.096175
H 2.700827 4.488031 0.572694
C 1.737210 2.650844 0.043337
H 0.999695 2.777341 0.833816
C -1.453169 2.429041 -0.170980
C -1.232857 2.903121 -1.467327
H -0.684694 2.280214 -2.171452
C -1.698059 4.155847 -1.837230

H -1.517015 4.521607 -2.845726
C -2.385107 4.945159 -0.919038
H -2.748250 5.928905 -1.210572
C -2.596724 4.481822 0.373777
H -3.121903 5.100784 1.098391
C -2.125828 3.228745 0.752784
H -2.268522 2.893759 1.775753
C -2.000506 -0.502203 -0.078007
C -1.984583 -1.701595 0.639401
C -2.905113 -2.713237 0.406023
C -3.864882 -2.549111 -0.584136
C -3.898632 -1.376214 -1.326661
C -2.963204 -0.379059 -1.080496
C -0.604381 0.756245 2.061134
C -1.710366 0.747665 2.915194
H -2.720616 0.791282 2.507754
C -1.526724 0.655268 4.288664
H -2.389007 0.643732 4.952567
C -0.239479 0.564385 4.812550
H -0.096954 0.489721 5.888990
C 0.860169 0.547107 3.962238
H 1.864610 0.451637 4.369684
C 0.682773 0.631882 2.584916
H 1.537665 0.565468 1.909030
Sn 1.149324 -1.555358 -0.989249
H 0.841611 -2.356092 -2.495547
H 1.763970 -1.422765 -2.841900
C 3.211655 -1.757768 -0.327050
N 3.557003 -0.912051 0.644752
N 4.007888 -2.681275 -0.863369
C 4.810353 -0.966561 1.081250
C 5.256860 -2.732901 -0.416686
C 5.728835 -1.871667 0.567183
H 5.089293 -0.263659 1.869179
H 5.908021 -3.490164 -0.859567
H 6.756239 -1.911164 0.920589
F -1.062557 -1.935254 1.558788
F -2.856729 -3.835226 1.097787
F -4.739735 -3.503107 -0.819654
F -4.805398 -1.221550 -2.272905
F -3.022898 0.697099 -1.842609

^{F}YSnB2
E = -2216.50349980
P -1.678443 -0.803453 0.194482
C -0.427393 0.237807 -0.307538
C -0.522212 1.685436 -0.280319
C 0.612235 2.485923 -0.046762
C 0.574375 3.870427 -0.023090
C -0.636749 4.537008 -0.171777
C -1.789243 3.791461 -0.375794
C -1.716244 2.407688 -0.424919
C -2.942455 -1.331256 -1.024316
C -2.554469 -2.238302 -2.017267
H -1.564266 -2.692376 -1.982624
C -3.422585 -2.566662 -3.050396
H -3.108536 -3.275717 -3.813714
C -4.686665 -1.988278 -3.108192
H -5.367540 -2.244842 -3.917502
C -5.075143 -1.076627 -2.132718

H -6.059007 -0.613892 -2.178216
C -4.208441 -0.744491 -1.098177
H -4.514438 -0.014907 -0.352889
C -0.910903 -2.363997 0.756027
C 0.354255 -2.299474 1.349793
C 0.949570 -3.449475 1.853212
C 0.278892 -4.666370 1.790450
C -0.993097 -4.729884 1.230338
C -1.589312 -3.584084 0.716010
C -2.585314 -0.221544 1.658328
C -3.750569 -0.874812 2.068174
H -4.154670 -1.694121 1.476013
C -4.394571 -0.487252 3.235720
H -5.302489 -0.999423 3.547689
C -3.876995 0.550781 4.004419
H -4.384696 0.857908 4.916703
C -2.704252 1.186590 3.613073
H -2.288605 1.988442 4.219777
C -2.051195 0.797405 2.448574
H -1.119546 1.275368 2.157569
Sn 1.041420 -0.763608 -1.532719
H 0.902490 -0.308173 -3.224632
H 1.368255 0.769813 -2.641089
H 0.869214 -1.341607 1.414093
H 1.941368 -3.388006 2.296390
H 0.745508 -5.567640 2.183509
H -1.524065 -5.678764 1.187673
H -2.582477 -3.643933 0.275671
C 5.292372 -0.307081 -0.557842
C 4.751270 -0.491168 0.710464
C 6.647783 -0.138639 -0.757603
C 5.537880 -0.515100 1.844673
C 7.453519 -0.162201 0.382664
H 7.056224 0.005616 -1.754021
C 6.911086 -0.345990 1.654666
H 5.102093 -0.655425 2.830153
H 8.528234 -0.032441 0.275464
H 7.571303 -0.355910 2.519181
B 3.121309 -0.511663 -0.756607
O 3.397792 -0.631942 0.594625
O 4.283177 -0.327714 -1.477520
F 1.791796 1.903707 0.160636
F 1.682802 4.563654 0.190640
F -0.687362 5.860315 -0.125076
F -2.856398 1.743625 -0.642408
F -2.954795 4.403230 -0.529299

^{F}YSnB1
E = -2485.69725006
P 1.950077 1.244261 -0.137903
C 0.506537 0.356642 -0.213796
C 0.500344 -1.005049 -0.719083
C 1.463958 -1.962153 -0.366219
C 1.478471 -3.252407 -0.866514
C 0.473326 -3.667060 -1.727522
C -0.528536 -2.770810 -2.075562
C -0.494756 -1.469459 -1.597504
C 2.957634 1.227997 1.391453
C 4.246455 1.766161 1.453222
H 4.702051 2.196352 0.561600

C 4.954870 1.740130 2.648589
H 5.959894 2.155432 2.692391
C 4.381242 1.178423 3.786412
H 4.938177 1.158273 4.721290
C 3.102400 0.635550 3.726208
H 2.656679 0.188059 4.612814
C 2.394926 0.656438 2.529755
H 1.405575 0.207856 2.452233
C 1.539517 3.001848 -0.413015
C 2.143198 4.054459 0.277586
H 2.887611 3.854269 1.043162
C 1.775262 5.367817 0.007909
H 2.244524 6.181203 0.557772
C 0.804818 5.641336 -0.949615
H 0.516889 6.670789 -1.154477
C 0.193253 4.596782 -1.635388
H -0.579023 4.802198 -2.373854
C 0.554130 3.282881 -1.367040
H 0.049141 2.456957 -1.867752
C 3.094996 0.724365 -1.458560
C 4.193969 -0.097322 -1.199449
H 4.419165 -0.399437 -0.180251
C 4.984735 -0.553795 -2.248297
H 5.835134 -1.199333 -2.038280
C 4.686482 -0.194780 -3.557398
H 5.307069 -0.554242 -4.376013
C 3.588927 0.619277 -3.820773
H 3.347952 0.899263 -4.844192
C 2.794051 1.074002 -2.777459
H 1.933389 1.703622 -2.990209
Sn -1.223589 1.102775 0.816553
N -4.197342 -0.380662 0.509807
C -3.814875 -2.540439 0.955024
C -4.750833 -1.656478 0.556331
H -3.927559 -3.599068 1.154249
H -5.800349 -1.830278 0.350589
B -2.812512 -0.480818 0.879963
N -2.611243 -1.874451 1.184892
C -1.451690 -2.556308 1.599339
C -1.187823 -3.847920 1.138046
C -0.553558 -1.959313 2.488845
C -0.043832 -4.520775 1.548188
H -1.863838 -4.311296 0.422949
C 0.599448 -2.629523 2.875782
H -0.781724 -0.977530 2.899697
C 0.863863 -3.911869 2.407046
H 0.158642 -5.515738 1.156221
H 1.296726 -2.146259 3.557345
H 1.772083 -4.430269 2.704632
C -4.920154 0.743888 0.075606
C -4.727601 1.979518 0.695252
C -5.834166 0.642348 -0.975058
C -5.426472 3.097484 0.259909
H -4.037209 2.053400 1.534325
C -6.541765 1.761367 -1.394704
H -5.967212 -0.312478 -1.478694
C -6.340106 2.994479 -0.783507
H -5.263102 4.054690 0.751481
H -7.247660 1.669415 -2.218056
H -6.892374 3.869883 -1.118593

H -2.169126 1.952272 -0.683932
H -1.741066 2.691484 0.337370
F -1.451635 -0.643857 -2.008338
F 2.401689 -1.644498 0.532795
F 2.393908 -4.120371 -0.455503
F -1.502165 -3.159134 -2.888371
F 0.438938 -4.919765 -2.164120

I
E = -1022.56486991
C 1.597128 2.505454 0.195586
C -0.514914 1.343281 -0.064459
C -1.110082 2.575361 -0.352906
C -0.374707 3.755445 -0.354333
C 0.982913 3.724753 -0.064808
H 2.661701 2.494979 0.428187
H -2.167919 2.604741 -0.599021
H -0.871682 4.696474 -0.581678
H 1.567719 4.642228 -0.042209
N -1.213988 0.138547 -0.052605
C -2.602778 0.011307 -0.053365
C -3.444762 0.828233 0.717638
C -3.186232 -1.023434 -0.798128
C -4.815934 0.615965 0.726896
H -3.008554 1.617880 1.324428
C -4.558070 -1.239322 -0.771208
H -2.543388 -1.648996 -1.418928
C -5.384841 -0.416767 -0.014478
H -5.448839 1.259253 1.336364
H -4.983691 -2.050650 -1.359369
H -6.460437 -0.578988 0.001274
C 0.897024 1.289372 0.173705
C 1.537454 -0.009060 0.341494
P 3.158895 -0.325957 -0.025262
C 3.694851 0.333317 -1.633932
H 3.473939 1.405951 -1.660480
H 4.763619 0.170245 -1.811725
H 3.101309 -0.158200 -2.412628
C 4.411654 0.273441 1.156924
H 5.420564 -0.024511 0.848068
H 4.365220 1.364904 1.226092
H 4.183319 -0.143356 2.143410
C 3.411271 -2.115077 -0.109106
H 4.454105 -2.351480 -0.344854
H 3.150178 -2.565689 0.855004
H 2.761201 -2.519623 -0.897541
H -0.164552 -2.962664 -0.559411
H 0.393064 -2.218698 -1.751433
Sn 0.064555 -1.476558 0.229010

II
E = -1571.77998350
C 1.327305 2.857317 0.341540
C 0.682275 1.639576 0.539645
C -0.563669 1.342055 -0.075539
C -1.084080 2.350794 -0.914197
C -0.434380 3.557505 -1.108716
C 0.779244 3.829900 -0.480410
H 2.269839 3.015794 0.862221
H -2.020803 2.157093 -1.430249

H -0.882673 4.295104 -1.772441
H 1.284130 4.781948 -0.624592
N -1.231352 0.138067 0.059030
S 1.549507 0.407799 1.489901
O 2.817238 0.990696 1.926271
O 0.586296 -0.095599 2.498242
C 1.732749 -0.900939 0.393992
C -2.620109 0.069125 -0.135747
C -3.489554 0.970538 0.496387
C -3.168375 -0.950513 -0.920412
C -4.861360 0.849232 0.340257
H -3.069569 1.758613 1.118067
C -4.547248 -1.077236 -1.057754
H -2.499701 -1.631765 -1.446844
C -5.401046 -0.177584 -0.433101
H -5.520379 1.555406 0.842545
H -4.952151 -1.880124 -1.671311
H -6.478982 -0.273378 -0.544107
P 2.609536 -0.656120 -1.061551
C 4.092704 0.325398 -0.745280
H 4.740032 0.319139 -1.628769
H 3.791324 1.350985 -0.509336
H 4.610702 -0.081596 0.127770
C 1.736078 0.191918 -2.411146
H 1.555299 1.235429 -2.127953
H 2.316835 0.155913 -3.340050
H 0.770972 -0.307730 -2.558818
C 3.120927 -2.249634 -1.735515
H 3.717798 -2.776629 -0.984778
H 2.213905 -2.827139 -1.949687
H 3.697884 -2.113305 -2.656845
Sn -0.219329 -1.677751 0.515367
H -0.814224 -3.046753 -0.308321
H -0.116522 -2.374419 -1.453333

III
E = -2333.86304916
C 0.331955 1.901499 0.501286
C -0.487538 1.848938 -0.633145
C -0.887445 3.037921 -1.244817
C -0.511896 4.271254 -0.731430
C 0.273327 4.326181 0.412155
C 0.688110 3.148440 1.018254
H -1.519269 2.966340 -2.126123
H -0.838101 5.184478 -1.225238
H 0.568302 5.283970 0.836397
H 1.318037 3.168255 1.903553
S 0.965383 0.503425 1.508608
S -1.079474 0.351525 -1.494346
O 2.134517 1.118504 2.174310
O -0.178805 0.096306 2.333575
O 0.098322 -0.137381 -2.249361
O -2.246111 0.856052 -2.241766
C 1.529201 -0.746052 0.521888
C -1.529277 -0.829084 -0.388153
P -2.952332 -0.542029 0.518703
P 2.998346 -0.409163 -0.296541
C -3.002764 -1.605714 1.967444
H -3.910978 -1.397575 2.543606
H -2.986155 -2.657404 1.666281

H -2.104246 -1.383702 2.554761
C -3.094643 1.151446 1.141259
H -3.111553 1.839284 0.289192
H -4.015789 1.262783 1.724032
H -2.214963 1.347860 1.764478
C -4.456060 -0.821086 -0.452725
H -4.497603 -1.875010 -0.747108
H -5.362521 -0.547139 0.099128
H -4.353072 -0.209831 -1.356714
C 3.197582 -1.582213 -1.654076
H 4.148140 -1.398729 -2.166580
H 3.179428 -2.608880 -1.273534
H 2.362220 -1.431751 -2.349193
C 3.074987 1.223397 -1.075557
H 3.004943 1.989582 -0.296828
H 4.013676 1.340315 -1.628602
H 2.215148 1.302332 -1.751489
C 4.462758 -0.526794 0.759850
H 5.376861 -0.245635 0.224705
H 4.283860 0.146104 1.605419
H 4.543594 -1.551612 1.136875
H -0.049483 -3.695112 0.565465
H -0.412421 -2.851875 1.629597
Sn 0.110081 -2.152900 -0.163403

IV
Not observed.

V
Not observed.

Pathway B

${}^{Tos}Y_2Sn$
E = -3797.71955061
C 3.582518 2.937439 -0.035086
H 3.004750 3.242717 0.833601
C 3.061835 1.968040 -0.878045
C -4.284027 -1.485434 -0.966229
O 0.578144 1.569133 -1.651957
C -4.595484 -0.943368 -2.210227
H -3.808339 -0.530236 -2.841286
C 2.583776 -2.984479 2.562577
H 3.594093 -2.640116 2.780184
C 4.836255 3.472185 -0.310475
H 5.253791 4.226384 0.355664
C -0.828129 5.035808 0.370083
H -0.307643 5.744132 1.011523
C -1.140342 3.773039 0.855760
H -0.829562 3.488038 1.856193
C 3.527116 -2.288980 -2.260899
H 2.846578 -1.529440 -2.640742
C 3.640034 -2.460768 -0.883299
C 5.222035 -0.272268 0.732447
H 5.562806 -0.796111 -0.158098
C 6.090108 0.567104 1.421376
H 7.112460 0.691454 1.069525
C -1.156368 5.384579 -0.935323
H -0.901230 6.372085 -1.315696
C -1.911186 0.051831 -0.603475

Sn -0.112784 -0.568806 -2.301125
O -2.598899 -2.001395 0.963463
S -2.583071 -1.496074 -0.422047
P -2.234960 1.206788 0.635057
C -5.918526 -0.919584 -2.620277
H -6.168973 -0.492291 -3.590619
C -4.502953 0.447561 2.100980
H -3.807567 -0.058886 2.765162
C -0.546997 -0.107856 2.377014
H -0.261127 -0.660941 1.488608
C 4.337216 1.105477 2.986795
H 3.985829 1.651170 3.860156
C 3.462661 0.271865 2.302679
H 2.426145 0.185257 2.622512
C -2.106819 3.198933 -1.282357
H -2.549493 2.466898 -1.954534
C 1.918632 -2.534311 1.418574
C 3.766240 1.546390 -2.000335
H 3.335680 0.788378 -2.651602
C 4.495019 -3.443037 -0.375832
H 4.579318 -3.592162 0.699455
C 3.909452 -0.432415 1.179915
C -1.295018 1.379599 4.610849
H -1.586982 1.961120 5.483144
C -1.468207 0.924141 2.250153
C -1.848702 1.668321 3.369819
H -2.590963 2.460458 3.276659
C -1.800393 2.856288 0.037303
C -1.788363 4.462610 -1.761745
H -2.018946 4.717576 -2.793896
C 6.950041 3.590311 -1.676058
H 7.143863 3.699031 -2.749179
H 7.717429 2.912393 -1.276055
H 7.096049 4.565826 -1.200135
C -0.386154 0.332097 4.738435
H 0.029453 0.091681 5.715548
C -6.938859 -1.432242 -1.809752
C 5.651172 1.250004 2.550658
H 6.333078 1.906850 3.087654
C -6.293474 1.640341 0.335405
H -6.990299 2.096496 -0.364997
C 1.584539 -0.434130 -0.606698
P 2.684940 -1.383180 0.235217
O 1.090242 1.814658 0.797794
S 1.489735 1.209828 -0.485532
O -1.855048 -2.314552 -1.440876
C 5.575606 3.051202 -1.415844
C 5.015034 2.086982 -2.262034
H 5.570390 1.755466 -3.138663
C 4.264946 -3.089732 -3.127445
H 4.165308 -2.956716 -4.202849
C 5.122831 -4.057615 -2.619199
H 5.701223 -4.682986 -3.296712
C 5.237898 -4.233647 -1.242006
H 5.905090 -4.995054 -0.842825
C -4.926928 1.777366 0.132536
H -4.563970 2.340220 -0.724604
C -0.018909 -0.415938 3.624665
H 0.673820 -1.248997 3.723854
C -4.021495 1.193767 1.022026

C -6.768676 0.925252 1.430888
H -7.840446 0.822707 1.590644
C -6.591939 -1.983962 -0.577374
H -7.371849 -2.391008 0.065141
C -5.872569 0.329001 2.309224
H -6.238424 -0.247973 3.156432
C 0.632199 -4.255313 3.187826
H 0.124919 -4.915918 3.888601
C 1.940313 -3.849476 3.440933
H 2.456397 -4.194728 4.334913
C -5.269241 -2.013192 -0.148200
H -4.987523 -2.426838 0.817034
C -8.370526 -1.357425 -2.248036
H -8.478405 -1.598881 -3.311869
H -8.769981 -0.343448 -2.105828
H -9.002977 -2.043498 -1.674851
C -0.032722 -3.805908 2.051196
H -1.066087 -4.080626 1.854257
C 0.613884 -2.956763 1.161874
H 0.078814 -2.587268 0.288223
H -2.059571 0.488024 -1.888971
H -1.859702 0.645013 -2.959063

^{Tos}YSnCl
E = -2361.74633085
C 2.470772 -1.209298 -0.677272
C 2.729866 -1.739661 0.582743
H 1.947647 -2.275438 1.119325
C -2.473625 1.699634 3.637945
H -3.335458 2.286701 3.947933
C -2.111746 1.669603 2.297442
H -2.709242 2.205336 1.565564
C -1.755324 0.965718 4.575611
H -2.047716 0.987492 5.623636
C -0.280368 -0.877056 -0.202931
Sn -1.989929 -2.517944 -0.340368
O 0.830959 -0.542301 -2.598444
S 0.838566 -1.373167 -1.383531
P -0.476031 0.787001 0.165414
C 3.987522 -1.572644 1.141309
H 4.194592 -1.981631 2.129479
C 1.417830 2.238732 -1.248539
H 0.733757 2.123255 -2.085508
C -2.154283 1.054568 -2.016576
H -1.897608 0.014778 -2.205376
C -0.312123 0.150017 2.831180
H 0.514319 -0.480779 2.505350
C -2.678539 3.756788 -1.581108
H -2.875041 4.814524 -1.418147
C -1.609290 1.702451 -0.911048
C -1.853061 3.063539 -0.706508
H -1.386068 3.584021 0.128856
C -1.016593 0.908050 1.890780
C -0.677294 0.186129 4.170813
H -0.126056 -0.407661 4.896865
C -3.243857 3.099803 -2.671443
H -3.888307 3.645375 -3.358096
C 4.998353 -0.889677 0.454416
C 3.201327 2.476787 0.876531
H 3.896995 2.565908 1.708545

O 0.571289 -2.823010 -1.524314
C 1.987288 1.827842 1.063773
H 1.742053 1.424366 2.042214
C -2.972803 1.754943 -2.894935
H -3.394705 1.247604 -3.759693
C 1.086441 1.711140 0.003164
C 3.522822 3.012787 -0.366156
H 4.473046 3.523956 -0.509027
C 4.717011 -0.395345 -0.819452
H 5.493823 0.131246 -1.372484
C 2.631664 2.890744 -1.426367
H 2.882076 3.301117 -2.402474
C 3.458321 -0.547804 -1.389594
H 3.219450 -0.146522 -2.371621
C 6.338257 -0.666632 1.088198
H 6.614883 -1.495145 1.749520
H 6.332002 0.247986 1.698085
H 7.124949 -0.550167 0.335137
H -0.408546 -1.884954 0.961440
H -0.795795 -2.644824 1.430486
Cl -3.573799 -0.875187 0.651993

^{Tos}YSnHMDS

Not observed.

^{Tos}YSnB1

Not observed.

$^{Ph}Y_2Sn$
E = -2620.52892475
P 3.115203 0.215335 0.028417
P -3.173986 0.277747 0.087268
C 1.648535 -0.027753 -0.778797
C 1.291461 -1.331665 -1.321174
C 1.783466 -2.559793 -0.834712
H 2.424610 -2.561636 0.044608
C 1.434012 -3.768701 -1.416484
H 1.834783 -4.692416 -1.001581
C 0.550599 -3.815729 -2.491889
H 0.268132 -4.767349 -2.937368
C 0.038985 -2.620663 -2.987830
H -0.642831 -2.630458 -3.838103
C 0.416389 -1.408552 -2.426244
H 0.069041 -0.478014 -2.881482
C 3.309543 -0.389184 1.754344
C 3.972021 -1.583102 2.050550
H 4.484770 -2.130783 1.261920
C 3.982550 -2.080745 3.349244
H 4.505104 -3.010659 3.565233
C 3.326428 -1.395639 4.365610
H 3.335823 -1.784882 5.381931
C 2.646124 -0.215849 4.075958
H 2.116539 0.317420 4.863028
C 2.632660 0.281017 2.779252
H 2.076785 1.190987 2.554180

C 3.474198 2.003218 0.116605
C 4.137387 2.596598 1.192650
H 4.406563 2.004803 2.064071
C 4.459053 3.949135 1.157158
H 4.969366 4.402859 2.004536
C 4.131263 4.718275 0.046158
H 4.380991 5.777415 0.023300
C 3.495393 4.127719 -1.040713
H 3.249866 4.719681 -1.920065
C 3.177439 2.775772 -1.010964
H 2.705003 2.298433 -1.867816
C 4.579896 -0.457691 -0.837395
C 4.454139 -0.944056 -2.138474
H 3.471041 -0.988015 -2.600781
C 5.583334 -1.365773 -2.832899
H 5.478504 -1.755360 -3.843565
C 6.839507 -1.288455 -2.241566
H 7.720860 -1.616954 -2.789472
C 6.971351 -0.783354 -0.951036
H 7.954350 -0.709294 -0.489825
C 5.845503 -0.368967 -0.251344
H 5.950005 0.029669 0.757506
C -1.515648 -0.010266 0.336786
C -1.095007 -1.354686 0.770586
C -0.144435 -1.487173 1.797571
H 0.259693 -0.587447 2.250802
C 0.273547 -2.728133 2.253836
H 1.011174 -2.780504 3.053408
C -0.253873 -3.894535 1.704835
H 0.068346 -4.870472 2.063621
C -1.190604 -3.791859 0.682945
H -1.598280 -4.690991 0.222905
C -1.591072 -2.545962 0.214804
H -2.285310 -2.493177 -0.622517
C -4.216469 -0.355632 1.445522
C -4.511415 0.440918 2.553940
H -4.209926 1.485594 2.570576
C -5.199224 -0.091525 3.637445
H -5.431304 0.541044 4.492075
C -5.589207 -1.426967 3.629773
H -6.123269 -1.844080 4.481492
C -5.297488 -2.226656 2.530596
H -5.598150 -3.272391 2.519008
C -4.618782 -1.695029 1.440446
H -4.403979 -2.324067 0.580053
C -3.456343 2.072829 -0.028099
C -4.263048 2.644677 -1.013341
H -4.755267 2.015109 -1.749970
C -4.429587 4.024531 -1.068092
H -5.056428 4.459665 -1.843982
C -3.794338 4.844283 -0.142888
H -3.924449 5.923825 -0.190653
C -2.985501 4.281990 0.839339
H -2.476802 4.917692 1.561120
C -2.813099 2.905012 0.896124
H -2.166701 2.472846 1.656879
C -4.005914 -0.400660 -1.405478
C -5.394805 -0.524773 -1.502534
H -6.026796 -0.240601 -0.661955
C -5.968618 -1.016683 -2.668832

H -7.050207 -1.115547 -2.741092
C -5.160997 -1.388865 -3.741100
H -5.613162 -1.778739 -4.651255
C -3.778627 -1.271933 -3.645421
H -3.144277 -1.572460 -4.477310
C -3.203172 -0.779769 -2.478838
H -2.121860 -0.710451 -2.371553
Sn 0.034234 1.464352 -0.704607
H -0.752349 0.994923 1.283226
H -0.055012 1.625744 1.524387

PhYSnCl
Not observed.

PhYSnHMDS
E = -2186.92670619
P -1.954194 0.132567 -0.006917
C -0.351144 -0.201840 -0.453767
C 0.061788 -1.609217 -0.214317
C -0.189698 -2.610622 -1.167454
H -0.655599 -2.331216 -2.111392
C 0.124596 -3.943599 -0.922626
H -0.091981 -4.696813 -1.678616
C 0.715146 -4.313584 0.281485
H 0.963367 -5.355417 0.475079
C 0.992571 -3.335522 1.231698
H 1.458271 -3.610510 2.177203
C 0.673570 -2.006884 0.983329
H 0.871128 -1.251401 1.738857
C -3.231030 -0.508603 -1.156415
C -4.515700 -0.856046 -0.731862
H -4.776826 -0.786727 0.322586
C -5.459203 -1.295425 -1.653715
H -6.456923 -1.571068 -1.317395
C -5.127707 -1.386486 -3.002306
H -5.866818 -1.734631 -3.721470
C -3.851004 -1.036996 -3.430750
H -3.589043 -1.108532 -4.484546
C -2.904607 -0.601568 -2.510197
H -1.899677 -0.334288 -2.835321
C -2.269452 1.918161 0.081363
C -3.299004 2.532154 -0.632034
H -3.952701 1.937852 -1.266204
C -3.486798 3.907796 -0.538431
H -4.287869 4.380923 -1.102710
C -2.655230 4.675316 0.268628
H -2.805273 5.750984 0.338049
C -1.628771 4.067874 0.986110
H -0.973731 4.663525 1.618594
C -1.434337 2.696628 0.889938
H -0.631690 2.217573 1.448019
C -2.458431 -0.587337 1.594487
C -2.510808 -1.984242 1.687142
H -2.331828 -2.597296 0.804500
C -2.783772 -2.594063 2.904158
H -2.814963 -3.679991 2.965344
C -3.010670 -1.820828 4.038786
H -3.224064 -2.300690 4.992049
C -2.967957 -0.433948 3.951357
H -3.154286 0.175454 4.833582

C -2.692364 0.182908 2.735263
H -2.670957 1.268583 2.678189
Sn 1.243019 1.453393 -0.939484
N 2.963724 0.401485 -0.212447
Si 3.362104 0.687245 1.457776
Si 4.005956 -0.546214 -1.247504
C 1.848273 1.413915 2.337041
H 1.633500 2.432132 1.977389
H 0.942197 0.804727 2.219425
H 2.052444 1.503480 3.412367
C 3.909628 -0.866579 2.366921
H 4.094497 -0.644984 3.426629
H 3.159466 -1.662398 2.303811
H 4.842583 -1.261835 1.946028
C 4.711702 1.984678 1.674122
H 4.858691 2.224814 2.736145
H 5.673854 1.650007 1.270214
H 4.437830 2.911792 1.153085
C 5.827074 -0.239074 -0.857507
H 6.451661 -0.841418 -1.530955
H 6.090093 0.815729 -1.008576
H 6.100307 -0.509882 0.169830
C 3.778737 -0.057708 -3.052658
H 4.549141 -0.552488 -3.659648
H 2.801800 -0.355997 -3.451006
H 3.891415 1.025712 -3.197625
C 3.674839 -2.384135 -1.077930
H 2.635529 -2.618325 -1.338178
H 4.335614 -2.969994 -1.731324
H 3.833426 -2.717703 -0.044693
H 0.733934 0.138203 -2.627951
H 0.095107 -0.081608 -1.937223

^{Ph}YSnB1
E = -1988.81751873
P 1.898695 0.726124 0.155615
C 0.297287 0.192871 0.382778
C -0.002613 -0.727374 1.483608
C -1.263605 -0.699864 2.114791
H -2.012819 0.002606 1.754376
C -1.565343 -1.525080 3.188046
H -2.556339 -1.470297 3.636473
C -0.611697 -2.402485 3.703420
H -0.845541 -3.040554 4.553427
C 0.646618 -2.439306 3.111760
H 1.411801 -3.113148 3.495304
C 0.938087 -1.631965 2.019493
H 1.926452 -1.705446 1.566666
C 1.904386 2.354741 -0.668238
C 1.674458 3.497609 0.100630
H 1.571996 3.413738 1.180569
C 1.576543 4.742425 -0.507164
H 1.398965 5.627300 0.100556
C 1.703982 4.855674 -1.888160
H 1.626330 5.831479 -2.363998
C 1.925689 3.721058 -2.660007
H 2.015325 3.802974 -3.741306
C 2.023088 2.473050 -2.054596
H 2.179869 1.586875 -2.665674
C 2.989345 -0.301423 -0.893251

C 4.197151 0.165445 -1.421858
H 4.520925 1.186109 -1.219797
C 4.976269 -0.663590 -2.218472
H 5.915454 -0.297304 -2.628806
C 4.549317 -1.959630 -2.497329
H 5.158755 -2.608383 -3.123855
C 3.341709 -2.422059 -1.986927
H 3.000810 -3.429554 -2.211369
C 2.557206 -1.594695 -1.189094
H 1.599947 -1.943292 -0.795966
C 2.772672 0.958524 1.741278
C 2.007439 1.369263 2.838351
H 0.933870 1.504518 2.718842
C 2.609138 1.572533 4.072713
H 2.004606 1.885373 4.921575
C 3.975934 1.360495 4.226551
H 4.445934 1.514803 5.196052
C 4.738204 0.939737 3.143013
H 5.805306 0.761885 3.261479
C 4.140294 0.736022 1.903619
H 4.743348 0.388117 1.069016
Sn -0.927666 0.361545 -1.651980
N -2.608789 -2.187944 -0.381085
C -4.445512 -1.468777 0.660856
C -3.662690 -2.548860 0.447115
H -5.384336 -1.412372 1.199440
H -3.805132 -3.569204 0.784768
B -2.696953 -0.780824 -0.671567
N -3.917599 -0.372380 -0.016913
C -4.434875 0.926524 0.109383
C -4.995437 1.363056 1.312194
C -4.377586 1.809442 -0.972312
C -5.488543 2.657410 1.425997
H -5.020243 0.691095 2.167309
C -4.858623 3.104767 -0.846826
H -3.959971 1.462351 -1.915234
C -5.420551 3.536308 0.351044
H -5.918261 2.983874 2.371353
H -4.803021 3.780197 -1.698463
H -5.802756 4.550527 0.445059
C -1.539018 -3.039161 -0.698806
C -1.059751 -3.095029 -2.011352
C -0.938925 -3.826090 0.285088
C -0.000388 -3.935753 -2.333902
H -1.556113 -2.507164 -2.782072
C 0.113905 -4.668450 -0.046138
H -1.279510 -3.737832 1.314644
C 0.583294 -4.733733 -1.355078
H 0.356732 -3.977423 -3.361318
H 0.580628 -5.268726 0.732482
H 1.402250 -5.404074 -1.609521
H -0.716777 1.456338 0.159536
H -1.327259 1.990126 -0.336236

I
E = -1022.62302873
C 1.613134 2.517621 -0.071080
C -0.494192 1.347089 -0.257819
C -1.121032 2.582211 -0.448851
C -0.400994 3.773059 -0.415476

C 0.968870 3.745595 -0.200389
H 2.694552 2.514131 0.064829
H -2.194656 2.609453 -0.616004
H -0.920935 4.719013 -0.554868
H 1.543920 4.668305 -0.152886
N -1.186045 0.137469 -0.205786
C -2.571436 0.063235 -0.037879
C -3.261668 0.834490 0.913618
C -3.305545 -0.877983 -0.774375
C -4.623635 0.664813 1.109961
H -2.707971 1.558048 1.507806
C -4.667927 -1.055297 -0.559908
H -2.791104 -1.460499 -1.538137
C -5.339109 -0.281763 0.378271
H -5.132911 1.271118 1.857712
H -5.209355 -1.795891 -1.146322
H -6.407102 -0.411568 0.540027
C 0.926963 1.298843 -0.137662
C 1.555522 -0.024887 -0.139685
P 3.199708 -0.286360 0.185941
C 4.367962 0.418998 -1.027402
H 4.237410 1.503455 -1.087536
H 5.406202 0.191400 -0.759399
H 4.137858 -0.006410 -2.010222
C 3.838463 0.279258 1.798248
H 4.911960 0.083291 1.904060
H 3.651877 1.354202 1.895817
H 3.286008 -0.235938 2.591085
C 3.523908 -2.067883 0.123191
H 4.598633 -2.270227 0.181807
H 3.015792 -2.568721 0.953976
H 3.134096 -2.463973 -0.822740
Sn -0.061012 -1.660627 -0.043711
H 0.793891 -1.443937 -2.037498
H 1.253405 -0.755208 -1.451141

II
Not observed.

III
E = -2333.91204800
C 0.315657 1.979858 0.347385
C -0.516326 1.838886 -0.768326
C -0.914199 2.972360 -1.477266
C -0.522460 4.241697 -1.075882
C 0.278480 4.386875 0.048776
C 0.692594 3.262541 0.749904
H -1.556820 2.826200 -2.341450
H -0.848107 5.112851 -1.640960
H 0.586155 5.374767 0.385942
H 1.333019 3.353928 1.622969
S 0.943363 0.677753 1.472462
S -1.137252 0.273526 -1.451125
O 2.111448 1.348087 2.088311
O -0.177938 0.340499 2.359258
O 0.041246 -0.327817 -2.149296
O -2.275339 0.708849 -2.279134
C -1.535315 -0.781741 -0.233179
P -2.906011 -0.487001 0.726207
P 3.031917 -0.422227 -0.127286

C -2.807508 -1.507653 2.206312
H -3.677856 -1.343580 2.850452
H -2.756606 -2.562701 1.916543
H -1.879473 -1.227114 2.718001
C -3.077659 1.232861 1.265770
H -3.146913 1.868458 0.375434
H -3.979313 1.356395 1.875967
H -2.181665 1.492253 1.839225
C -4.463683 -0.857837 -0.128599
H -4.492777 -1.925971 -0.368484
H -5.345322 -0.580572 0.460751
H -4.435881 -0.294111 -1.068802
C 3.319614 -1.708897 -1.361111
H 4.299752 -1.563507 -1.828629
H 3.275402 -2.700795 -0.899881
H 2.529222 -1.625840 -2.117538
C 3.117597 1.125890 -1.062211
H 3.035451 1.965500 -0.365252
H 4.061647 1.188160 -1.614984
H 2.265487 1.133580 -1.753207
C 4.446660 -0.431562 1.001423
H 5.383706 -0.215118 0.475918
H 4.241458 0.327380 1.763189
H 4.506130 -1.413017 1.483915
C 1.501808 -0.689415 0.625260
H 0.995769 -1.723013 1.487118
Sn 0.042190 -2.297561 -0.345223
H 0.284765 -2.468279 1.697067

IV
E = -1541.89807114
C -0.757062 3.382019 -0.164073
C -0.215438 2.115497 -0.326014
C 0.993466 1.664837 0.218091
C 1.704150 2.607247 0.975607
C 1.205611 3.891646 1.142188
C -0.014886 4.288145 0.579380
H -1.709364 3.651582 -0.616704
H 2.661887 2.337206 1.417703
H 1.780544 4.615805 1.717516
H -0.375570 5.304678 0.720264
B 1.262979 0.155149 -0.188668
S -0.990954 0.735745 -1.071384
O -1.834041 1.001255 -2.242072
O 0.329132 -0.033720 -1.415700
C 2.705428 -0.407319 -0.471357
C 3.698408 -0.404304 0.520675
C 3.057431 -0.925558 -1.724930
C 4.980975 -0.877096 0.272473
H 3.457040 -0.033077 1.518607
C 4.336692 -1.410211 -1.978270
H 2.308668 -0.941124 -2.515650
C 5.307135 -1.385397 -0.982259
H 5.729612 -0.857157 1.063483
H 4.579820 -1.806640 -2.963478
H 6.309099 -1.762971 -1.179131
C -1.749042 -0.138731 0.159038
H -1.360421 0.327546 1.508121
P -3.270382 -0.810558 -0.252643
C -4.538001 0.413542 -0.672815

H -4.676248 1.083218 0.182449
H -4.169664 0.990502 -1.527099
H -5.488184 -0.070538 -0.924859
C -3.882824 -1.724904 1.176850
H -4.858823 -2.170114 0.954699
H -3.158891 -2.505495 1.434761
H -3.973255 -1.039903 2.026539
C -3.216409 -1.962370 -1.649622
H -4.204994 -2.378271 -1.874383
H -2.838440 -1.402540 -2.512544
H -2.515351 -2.770026 -1.413694
H -0.791210 0.332275 2.341919
Sn 0.044706 -1.275936 1.386580

V
E = -992.668930301
C 1.645438 2.536064 -0.055488
C -0.493476 1.359603 -0.118661
C -1.117775 2.626159 -0.212928
C -0.408807 3.807689 -0.204388
C 0.989008 3.746403 -0.105089
H 2.732535 2.546679 0.004862
H -2.203200 2.657228 -0.293879
H -0.915995 4.767888 -0.269872
H 1.570449 4.667829 -0.077951
B -1.310209 0.069470 -0.076886
C -2.866577 -0.005282 -0.049893
C -3.628411 0.769821 0.842577
C -3.571090 -0.886841 -0.887447
C -5.014614 0.672683 0.893501
H -3.118671 1.453184 1.521171
C -4.957589 -0.963431 -0.866499
H -3.013953 -1.522326 -1.576730
C -5.686282 -0.186411 0.030447
H -5.574772 1.277046 1.605629
H -5.474317 -1.643196 -1.542420
H -6.772283 -0.256725 0.060644
C 0.945766 1.297871 -0.083249
C 1.593652 0.015766 -0.117408
P 3.283526 -0.190279 0.001752
C 4.297332 0.567974 -1.316287
H 4.129383 1.647824 -1.357157
H 5.363654 0.371129 -1.155509
H 3.985452 0.137935 -2.274230
C 4.052815 0.379187 1.554812
H 5.134162 0.199543 1.558655
H 3.859136 1.448240 1.689706
H 3.585753 -0.160614 2.385397
C 3.700567 -1.947463 -0.103448
H 4.788483 -2.076946 -0.112317
H 3.265971 -2.481041 0.747574
H 3.272834 -2.364977 -1.021678
Sn 0.085075 -1.774994 0.171270
H 1.057339 -0.776817 -1.429861
H 0.492688 -1.374132 -1.870703

Pathway C

IV
E = -1541.89766699

C 0.994756 3.227486 -0.049454
C 0.319085 2.044429 0.196492
C -0.947462 1.727541 -0.301109
C -1.553924 2.682221 -1.122072
C -0.903106 3.881835 -1.392399
C 0.358686 4.160907 -0.861635
H 1.968706 3.427250 0.393427
H -2.546811 2.499065 -1.529909
H -1.391738 4.626824 -2.018209
H 0.839110 5.113938 -1.073299
B -1.377028 0.288165 0.227988
S 0.923802 0.648201 1.104917
O 1.740119 1.062826 2.250930
O -0.511790 0.095863 1.455374
C 1.589061 -0.497213 0.110597
C -2.862131 -0.174980 0.394268
C -3.761293 -0.128867 -0.681091
C -3.351292 -0.625532 1.626802
C -5.088922 -0.511360 -0.534593
H -3.409363 0.195679 -1.661554
C -4.678380 -1.011613 1.779930
H -2.672113 -0.670986 2.477095
C -5.554161 -0.955086 0.700222
H -5.764475 -0.468102 -1.387828
H -5.033680 -1.357633 2.749739
H -6.593227 -1.257680 0.818708
P 3.285556 -0.619900 0.174130
C 4.173824 0.954222 0.000874
H 5.258909 0.802063 -0.014749
H 3.849353 1.448240 -0.921235
H 3.904296 1.578250 0.860371
C 3.844129 -1.682178 -1.175041
H 3.524800 -1.245546 -2.127037
H 4.934435 -1.784805 -1.154521
H 3.382675 -2.670220 -1.071475
C 3.928200 -1.337544 1.710038
H 3.549312 -0.713480 2.527102
H 3.522476 -2.348340 1.823494
H 5.023726 -1.366938 1.726829
Sn 0.047385 -1.756093 -1.004215
H -0.088980 -0.286216 -2.293321
H -0.674754 -0.025370 -1.404808

V
E = -992.683355000
C 1.626408 2.491038 -0.352953
C -0.522692 1.332698 -0.194508
C -1.144569 2.587226 -0.412112
C -0.428982 3.751966 -0.567462
C 0.974787 3.690278 -0.520157
H 2.715215 2.490386 -0.371074
H -2.232126 2.618579 -0.459352
H -0.932721 4.702471 -0.729633
H 1.559574 4.601968 -0.640683
B -1.343438 0.068073 0.015282
C -2.894729 -0.032482 0.137368
C -3.628618 0.852033 0.947799
C -3.616218 -1.041685 -0.521914
C -5.007494 0.742446 1.082483
H -3.100953 1.633257 1.494035

C -4.997579 -1.142443 -0.410712
H -3.076306 -1.756536 -1.143083
C -5.698740 -0.251059 0.395752
H -5.547228 1.437047 1.724483
H -5.530049 -1.926268 -0.947197
H -6.779757 -0.334820 0.494510
C 0.921106 1.261099 -0.199934
C 1.563409 -0.008851 -0.057445
P 3.212604 -0.176404 0.333418
C 4.452618 0.282767 -0.931680
H 4.354175 1.340714 -1.193164
H 5.472406 0.090809 -0.577567
H 4.257377 -0.310429 -1.831596
C 3.728415 0.738305 1.822651
H 4.803327 0.633510 2.008175
H 3.477368 1.797123 1.704314
H 3.163001 0.346290 2.674509
C 3.614440 -1.904676 0.695261
H 4.667075 -1.991992 0.985144
H 2.972123 -2.261565 1.506967
H 3.431277 -2.521406 -0.191197
Sn 0.242793 -1.716312 -0.355795
H -0.762757 -0.907698 1.136659
H -0.171926 -1.652098 1.593223

6.2.4 H_2 activated products

Pathway A

$^{Tos}Y_2Sn$
E = -3797.77917506
C -4.748563 -2.186115 0.563915
H -4.615272 -2.098582 1.639385
C -3.691900 -1.861047 -0.270138
C 3.691884 1.861097 -0.270030
O -1.128573 -2.292899 0.031406
C 3.818742 1.954522 -1.652813
H 2.971281 1.701891 -2.288511
C -2.520422 3.342746 2.429514
H -3.434819 3.754050 2.002494
C -5.956422 -2.589986 0.003275
H -6.793235 -2.836460 0.655873
C 1.314224 -4.979479 -1.745090
H 0.801149 -5.909263 -1.508999
C 1.560578 -4.057557 -0.733506
H 1.221002 -4.258367 0.278852
C -2.553635 2.568624 -2.353934
H -2.975502 1.595585 -2.600320
C -2.201138 2.855569 -1.030471
C -5.166441 1.368370 -0.534876
H -4.834604 1.605435 -1.541461
C -6.505818 1.078190 -0.312171
H -7.204641 1.087870 -1.146210
C 1.702231 -4.707902 -3.051182
H 1.505980 -5.432432 -3.839364
C 1.741173 -0.158863 -0.345802
O 2.420562 1.097097 1.867095
S 2.144165 1.296460 0.428323
P 2.469556 -1.588681 0.238199
C 5.028522 2.352653 -2.197064
H 5.133762 2.421903 -3.279390

C 4.705308 -1.038754 1.810343
H 3.995323 -0.984086 2.631441
C 0.707880 -1.749156 2.355877
H 0.201197 -0.935510 1.852415
C -6.049386 0.757018 2.028850
H -6.388592 0.508689 3.032623
C -4.705475 1.038570 1.810121
H -3.995643 0.983894 2.631350
C 2.554011 -2.568312 -2.354145
H 2.976042 -1.595293 -2.600329
C -1.866655 2.276330 1.802467
C -3.818794 -1.954414 -1.652873
H -2.971369 -1.701738 -2.288602
C -1.560637 4.057773 -0.732947
H -1.221219 4.258485 0.279484
C -4.258128 1.362371 0.526426
C 2.000657 -3.872172 3.602593
H 2.506955 -4.701073 4.093689
C 1.866626 -2.276555 1.802250
C 2.520268 -3.343127 2.429160
H 3.434621 -3.754479 2.002092
C 2.201296 -2.855406 -1.030768
C 2.318248 -3.497610 -3.356832
H 2.596981 -3.268587 -4.383418
C -7.436689 -3.059706 -1.981600
H -7.305707 -3.763512 -2.812104
H -7.959852 -2.181061 -2.384058
H -8.095586 -3.524494 -1.240685
C 0.830097 -3.344616 4.147699
H 0.422588 -3.769287 5.063610
C 6.120468 2.669735 -1.378511
C -6.950988 0.779045 0.971758
H -8.001653 0.553764 1.145302
C 6.506021 -1.078316 -0.311638
H 7.204987 -1.087917 -1.145557
C -1.741148 0.158880 -0.345868
P -2.469483 1.588660 0.238293
O -2.420620 -1.097096 1.867004
S -2.144180 -1.296454 0.428240
O 1.128553 2.292884 0.031462
C -6.120446 -2.669872 -1.378561
C -5.028593 -2.352679 -2.197109
H -5.133846 -2.422002 -3.279424
C -2.317886 3.498108 -3.356454
H -2.596454 3.269189 -4.383108
C -1.702104 4.708451 -3.050543
H -1.505872 5.433139 -3.838586
C -1.314303 4.979888 -1.744358
H -0.801405 5.909717 -1.508061
C 5.166674 -1.368438 -0.534598
H 4.835005 -1.605437 -1.541253
C 0.181579 -2.286340 3.524287
H -0.747443 -1.879933 3.916409
C 4.258174 -1.362448 0.526542
C 6.950975 -0.779272 0.972386
H 8.001618 -0.554041 1.146126
C 5.956534 2.589679 0.003274
H 6.793472 2.835844 0.655819
C 6.049192 -0.757281 2.029326
H 6.388240 -0.509041 3.033174

C -0.830269 3.344212 4.148067
H -0.422816 3.768811 5.064035
C -2.000889 3.871695 3.603025
H -2.507295 4.700465 4.094230
C 4.748602 2.185927 0.563970
H 4.615415 2.098236 1.639439
C 7.436299 3.060469 -1.981870
H 8.100850 3.511513 -1.237551
H 7.305556 3.776541 -2.801942
H 7.952744 2.184760 -2.399013
C -0.181615 2.286098 3.524516
H 0.747462 1.879752 3.916576
C -0.707846 1.748999 2.356035
H -0.201060 0.935470 1.852488
Sn 0.000018 0.000007 -1.571427
H 0.179817 1.375386 -2.567639
H -0.179760 -1.375356 -2.567664

^{Tos}YSnCl
E = -2361.77899927
C 3.365502 -0.408696 -1.459406
H 3.081885 0.117438 -2.367697
C 2.402565 -1.130652 -0.773727
O 0.438071 -2.586515 -1.731922
C -1.689783 3.182287 -0.904228
H -1.117991 3.753026 -0.172718
C 4.661518 -0.357203 -0.955814
H 5.419620 0.216042 -1.488049
C -0.701661 0.262826 2.890380
H 0.101406 -0.437596 2.665559
C -1.226770 1.056586 1.863968
C 1.953637 1.708991 1.251774
H 1.662283 1.180597 2.156063
C 3.203930 2.307895 1.181851
H 3.881396 2.242334 2.030619
C 2.723285 3.056412 -1.057154
H 3.026508 3.572732 -1.965647
C 1.473403 2.451189 -0.997693
H 0.816341 2.465763 -1.863313
C -1.577290 1.787945 -0.952644
C 2.722683 -1.817992 0.392940
H 1.952987 -2.388840 0.909305
C -2.294126 1.913036 2.135889
H -2.733240 2.511548 1.342152
C 1.077326 1.789323 0.166665
C 6.391456 -0.925518 0.787763
H 6.774786 -1.914815 1.065232
H 6.408623 -0.308236 1.696434
H 7.087968 -0.479500 0.070429
C 3.586401 2.989388 0.030219
H 4.566126 3.460508 -0.022165
C -0.294340 -0.777889 -0.089749
P -0.506566 0.895743 0.204459
O 0.677967 -0.157853 -2.458720
S 0.729015 -1.184464 -1.404270
C 5.004862 -1.014081 0.224694
C 4.014640 -1.751060 0.887389
H 4.267184 -2.282762 1.804237
C -1.223116 0.344489 4.173985
H -0.810444 -0.277475 4.965598

C -2.281530 1.207509 4.441803
H -2.696588 1.264702 5.446130
C -2.817840 1.985042 3.422402
H -3.656778 2.647719 3.624144
C -3.229639 3.109232 -2.752923
H -3.877282 3.626281 -3.458573
C -2.518788 3.839621 -1.802155
H -2.606915 4.923640 -1.766442
C -3.106285 1.726433 -2.809082
H -3.652754 1.154819 -3.555896
C -2.275953 1.060189 -1.913489
H -2.168263 -0.021073 -1.964694
Cl -3.345456 -2.449626 -1.309001
Sn -1.648848 -2.315024 0.388661
H -2.542525 -1.894658 1.777613
H -0.927763 -3.838920 0.516289

^{Tos}YSnHMDS
E = -2775.57458786
S 0.926416 -1.460415 0.835570
P 0.881949 1.311902 0.066364
Si -4.069196 0.424313 -0.337610
Si -3.447118 -2.389906 0.687790
O 0.057132 -2.638422 0.659207
O 1.106366 -0.911277 2.192173
N -3.037637 -0.989133 -0.299701
C 0.372546 -0.283128 -0.268609
C 2.561811 -1.953932 0.305444
C 2.776153 -2.307940 -1.023352
H 1.946307 -2.287530 -1.727850
C 4.049495 -2.666101 -1.433043
H 4.221739 -2.938702 -2.473751
C 5.120576 -2.686778 -0.529987
C 4.871600 -2.348468 0.798963
H 5.691237 -2.362435 1.516315
C 3.598398 -1.980803 1.223724
H 3.397677 -1.697565 2.254024
C 6.498501 -3.052318 -0.993984
H 6.496276 -4.002322 -1.542361
H 7.192755 -3.147702 -0.152864
H 6.900758 -2.290160 -1.674983
C 2.665265 1.384179 0.434639
C 3.585666 1.260118 -0.609343
H 3.242359 1.208160 -1.639787
C 4.945253 1.200754 -0.337074
H 5.655587 1.103757 -1.155461
C 5.395294 1.264495 0.978506
H 6.461791 1.219965 1.191037
C 4.481810 1.368598 2.020589
H 4.828715 1.398359 3.051506
C 3.117926 1.418544 1.754922
H 2.403268 1.454946 2.572496
C 0.092334 2.119282 1.488772
C 0.547920 3.352047 1.968177
H 1.378644 3.857040 1.475786
C -0.041411 3.921517 3.088676
H 0.312621 4.881126 3.459970
C -1.073322 3.253719 3.743982
H -1.526904 3.693716 4.630089
C -1.516237 2.021753 3.277158

H -2.308989 1.491210 3.800354
C -0.938809 1.451559 2.147740
H -1.274491 0.482091 1.780880
C 0.574967 2.327080 -1.407526
C 0.864262 1.779501 -2.661497
H 1.236232 0.758286 -2.725408
C 0.649257 2.521066 -3.815624
H 0.872445 2.084601 -4.786860
C 0.134338 3.810288 -3.728873
H -0.042371 4.388062 -4.633993
C -0.167864 4.354625 -2.485762
H -0.587241 5.355914 -2.414490
C 0.050962 3.618319 -1.327601
H -0.215106 4.043786 -0.363757
C -4.742096 0.861821 1.366708
H -5.439256 1.705851 1.276821
H -3.923369 1.178126 2.023252
H -5.272801 0.043074 1.863273
C -3.108091 1.951259 -0.881301
H -3.739790 2.837310 -0.730898
H -2.797153 1.938305 -1.932021
H -2.213056 2.086998 -0.261584
C -5.502695 0.186080 -1.529879
H -6.142034 -0.650554 -1.223954
H -5.123946 -0.035150 -2.536469
H -6.127376 1.087042 -1.593225
C -2.832983 -2.132723 2.443947
H -3.253942 -1.217097 2.879765
H -1.739289 -2.044725 2.439688
H -3.101393 -2.972866 3.097980
C -2.736157 -3.973201 -0.022204
H -3.122231 -4.819769 0.562069
H -1.643397 -3.999090 0.027155
H -3.052614 -4.117416 -1.063795
C -5.316051 -2.660277 0.690151
H -5.657644 -2.915355 -0.321690
H -5.907494 -1.805963 1.036602
H -5.553200 -3.511154 1.343093
Sn -1.293493 -0.976716 -1.412422
H -0.970741 -2.518108 -2.046114
H -1.567614 0.114063 -2.698613

$^{Tos}YSnC_6F_5$
E = -2630.11579236
C 3.530240 -1.743821 -1.647967
H 3.142066 -1.408859 -2.606529
C 2.654486 -1.899157 -0.585907
O 0.156894 -2.738393 -0.514404
C -0.164831 3.356315 -1.594796
H 0.636806 3.954747 -1.161950
C 4.884759 -1.997377 -1.454025
H 5.577415 -1.865790 -2.284378
C 1.252002 1.767131 2.691402
H 1.903892 0.897726 2.630196
C 0.549865 2.188183 1.555822
C 3.565824 1.481952 0.191069
H 3.441963 1.380459 1.265203
C 4.845720 1.566746 -0.338694
H 5.706866 1.532158 0.325286
C 3.920633 1.718654 -2.556327

H 4.055585 1.798607 -3.633054
C 2.635940 1.631007 -2.031581
H 1.775667 1.613997 -2.695376
C -0.317397 2.015586 -1.227473
C 3.111081 -2.324800 0.658244
H 2.407513 -2.449609 1.479600
C -0.366182 3.234785 1.666736
H -0.965068 3.531569 0.810251
C 2.451620 1.527340 -0.651042
C 6.834279 -2.664269 -0.000147
H 7.011871 -3.705963 0.296660
H 7.238309 -2.030581 0.799893
H 7.413184 -2.470814 -0.908953
C 5.024734 1.690989 -1.713274
H 6.029103 1.756034 -2.127472
C 0.504571 -0.382167 0.394333
P 0.775093 1.260254 0.011134
O 0.840684 -0.923238 -2.160205
S 0.922185 -1.518109 -0.813327
C 5.373443 -2.403709 -0.213926
C 4.462271 -2.568566 0.837935
H 4.823664 -2.895690 1.812358
C 1.077094 2.417584 3.904352
H 1.628281 2.083448 4.780876
C 0.180819 3.477747 4.002775
H 0.035955 3.981601 4.956409
C -0.546718 3.875369 2.888071
H -1.270871 4.683403 2.967268
C -2.055609 3.156613 -3.072944
H -2.741896 3.605264 -3.788535
C -1.035635 3.924391 -2.515242
H -0.920004 4.968634 -2.798557
C -2.192376 1.817790 -2.726092
H -2.982548 1.214390 -3.168165
C -1.316831 1.242855 -1.811891
H -1.388595 0.188485 -1.558613
Sn -0.875168 -1.159740 1.804460
H -1.083581 -0.078867 3.108851
H -0.470635 -2.704951 2.364944
C -2.781648 -1.152841 0.749870
C -4.498634 -2.173724 -0.627664
C -4.390913 0.228431 -0.443786
C -4.986170 -0.915627 -0.963086
C -3.305433 0.082310 0.407037
C -3.400315 -2.273662 0.220751
F -2.954837 -3.488406 0.508027
F -2.724583 1.196464 0.869256
F -5.079845 -3.252938 -1.123978
F -6.019371 -0.805446 -1.779268
F -4.844991 1.425243 -0.785292

^{Tos}YSnPy
E = -2149.25791222
C 3.736435 -0.247422 -1.691341
H 3.410917 0.363677 -2.529630
C 2.807609 -1.045515 -1.042653
O 0.845249 -2.379929 -2.179229
C -1.484600 3.111488 -0.233819
H -0.981215 3.550502 0.627014
C 5.051374 -0.226611 -1.237119

H 5.780596 0.408161 -1.739232
C 0.083549 -0.220763 2.899169
H 0.875467 -0.844011 2.485968
C -0.596244 0.667292 2.059126
C 2.470574 1.539497 1.282193
H 2.276242 0.896568 2.136245
C 3.687101 2.202481 1.190756
H 4.432794 2.069330 1.971848
C 2.997239 3.186708 -0.895961
H 3.203841 3.822331 -1.754633
C 1.779674 2.521628 -0.813012
H 1.051051 2.611903 -1.614128
C -1.213432 1.792625 -0.608996
C 3.182786 -1.833170 0.041268
H 2.438402 -2.451730 0.539461
C -1.666267 1.404873 2.565398
H -2.242475 2.052144 1.911296
C 1.506028 1.703269 0.285309
C 6.858726 -0.940382 0.370012
H 7.275087 -1.947484 0.494952
H 6.905224 -0.450152 1.351983
H 7.512534 -0.384813 -0.310167
C 3.949686 3.031194 0.104186
H 4.903678 3.550573 0.034280
C 0.138198 -0.856299 -0.223082
P -0.050359 0.754048 0.327796
O 1.010589 0.126595 -2.498807
S 1.108208 -1.052899 -1.614901
C 5.450115 -0.990375 -0.141335
C 4.494268 -1.798858 0.486828
H 4.787860 -2.409829 1.339991
C -0.276480 -0.336903 4.235373
H 0.256756 -1.032850 4.879597
C -1.328348 0.420126 4.739774
H -1.616648 0.324132 5.784848
C -2.025572 1.283053 3.901801
H -2.866765 1.856202 4.286199
C -3.002493 3.322216 -2.091688
H -3.707724 3.918498 -2.667865
C -2.386907 3.870124 -0.969058
H -2.603176 4.894362 -0.671519
C -2.703003 2.023588 -2.487945
H -3.166531 1.600075 -3.376370
C -1.807254 1.258653 -1.750606
H -1.546749 0.250734 -2.062690
C -3.201760 -1.385067 -0.287422
N -3.463750 -0.342502 0.512281
C -5.118089 -0.879563 -1.650505
C -4.518648 0.421103 0.255699
C -5.379204 0.198009 -0.813779
H -5.763171 -1.089125 -2.502840
H -4.685454 1.265425 0.929839
H -6.226698 0.857844 -0.986521
C -4.015227 -1.683248 -1.383608
H -3.780561 -2.529887 -2.027400
Sn -1.317234 -2.356178 0.208717
H -1.370172 -2.674290 1.888740
H -1.086821 -3.792057 -0.665088

^{Tos}YSnPyr
E = -2165.31623436
C 3.758051 -0.218816 -1.662084
H 3.445929 0.393751 -2.504461
C 2.820293 -1.024131 -1.034720
O 0.901763 -2.367869 -2.226922
C -1.472809 3.099902 -0.236410
H -0.961094 3.533602 0.622084
C 5.063609 -0.194886 -1.183214
H 5.799761 0.444351 -1.669403
C 0.042300 -0.323353 2.853608
H 0.804392 -0.968905 2.419113
C -0.600711 0.615302 2.040599
C 2.445916 1.513848 1.310597
H 2.239936 0.858982 2.153178
C 3.658032 2.188347 1.249047
H 4.390436 2.053010 2.042267
C 2.998203 3.189981 -0.839787
H 3.215556 3.836939 -1.687245
C 1.785623 2.513095 -0.787592
H 1.071677 2.604515 -1.601747
C -1.215646 1.778446 -0.613368
C 3.178662 -1.817541 0.050100
H 2.428389 -2.444930 0.528146
C -1.626424 1.395238 2.574732
H -2.173596 2.087396 1.941780
C 1.499653 1.680685 0.296881
C 6.839988 -0.895557 0.461784
H 7.201748 -1.885188 0.764126
H 6.883407 -0.250769 1.350858
H 7.539258 -0.486008 -0.274773
C 3.932840 3.031773 0.176842
H 4.883005 3.560734 0.130846
C 0.132808 -0.872494 -0.270554
P -0.052620 0.726265 0.311073
O 1.039547 0.145609 -2.515609
S 1.133965 -1.043976 -1.644898
C 5.444760 -0.962471 -0.083091
C 4.481806 -1.780040 0.520797
H 4.762801 -2.396576 1.374128
C -0.313859 -0.453889 4.189943
H 0.189692 -1.189985 4.813042
C -1.322733 0.341830 4.721281
H -1.607471 0.235028 5.766270
C -1.981104 1.260038 3.910663
H -2.787530 1.867043 4.317110
C -2.988745 3.331158 -2.093628
H -3.685898 3.936975 -2.669669
C -2.367164 3.869656 -0.969678
H -2.570881 4.896101 -0.670864
C -2.702231 2.030629 -2.493165
H -3.165129 1.615865 -3.386169
C -1.813713 1.254272 -1.757825
H -1.559842 0.246421 -2.077626
Sn -1.342266 -2.370740 0.103464
H -1.323946 -2.811494 1.758340
H -1.202085 -3.726849 -0.895898
C -3.221655 -1.311788 -0.244392
N -3.931261 -1.563071 -1.348215
N -3.513621 -0.351259 0.639967

C -4.994024 -0.799775 -1.577826
C -4.572912 0.410560 0.399250
C -5.369692 0.228586 -0.722674
H -5.566493 -1.012880 -2.483872
H -4.789886 1.199448 1.124424
H -6.234039 0.856854 -0.923590

^{Tos}YSnB2
E = -2308.27645164
C 1.403191 3.226276 1.086912
H 1.035151 3.003422 2.085517
C 0.891231 2.520464 0.010868
O -1.579935 1.764619 -0.379471
C 1.456126 -2.890518 2.455411
H 2.536014 -2.857633 2.311857
C 2.390672 4.181302 0.864339
H 2.802568 4.731794 1.709337
C 2.303782 -1.671474 -2.216313
H 2.076511 -0.623833 -2.409787
C 2.006266 -2.215450 -0.961221
C 3.642109 0.362045 0.260052
H 3.695828 0.112400 -0.797213
C 4.601585 1.194451 0.819636
H 5.401442 1.590655 0.197484
C 3.503451 1.027081 2.957843
H 3.440105 1.296162 4.010279
C 2.534874 0.199121 2.401962
H 1.702154 -0.152789 3.005478
C 0.614486 -2.140476 1.626794
C 1.341259 2.766239 -1.283083
H 0.926046 2.200115 -2.115249
C 2.252725 -3.567588 -0.723440
H 1.996125 -4.012341 0.234215
C 2.611837 -0.150350 1.052322
C 3.958470 5.443574 -0.654412
H 3.671846 6.165064 -1.429486
H 4.881822 4.956529 -0.995987
H 4.193422 6.001524 0.257805
C 4.537189 1.521359 2.171145
H 5.290505 2.173806 2.608715
C 0.149232 -0.127731 -0.537543
P 1.271631 -1.111009 0.281622
O -0.382108 1.069156 1.752447
S -0.358289 1.269949 0.292317
C 2.873170 4.436501 -0.418062
C 2.327834 3.716287 -1.488968
H 2.686127 3.907658 -2.500016
C 2.857854 -2.467337 -3.210169
H 3.083059 -2.036418 -4.183514
C 3.109956 -3.813430 -2.964482
H 3.536701 -4.439415 -3.745734
C 2.803551 -4.361071 -1.724141
H 2.984287 -5.416977 -1.533627
C -0.462278 -3.677943 3.678338
H -0.884365 -4.280392 4.480650
C 0.916163 -3.661987 3.475525
H 1.570750 -4.245872 4.119637
C -1.296044 -2.915030 2.869547
H -2.370899 -2.911440 3.038128
C -0.761802 -2.139623 1.845483

H -1.407878 -1.511115 1.235907
C -4.499876 0.206445 0.560714
C -4.950498 0.931933 -0.538504
C -5.152907 0.239498 1.775978
C -6.081057 1.721564 -0.476775
C -6.296077 1.038435 1.850461
H -4.783459 -0.323063 2.629090
C -6.750568 1.762152 0.748302
H -6.420635 2.284425 -1.341924
H -6.840431 1.100058 2.790415
H -7.643583 2.376029 0.844926
B -3.115208 -0.107071 -1.106945
O -3.380944 -0.484987 0.202919
O -4.114743 0.709468 -1.592916
Sn -1.209423 -0.767527 -2.075759
H -0.997532 -2.456574 -2.276301
H -0.809960 -0.008498 -3.560299

^{Tos}YSnB1
E = -2577.45693301
C 4.247123 1.214870 -0.896997
H 4.150044 2.042107 -0.197874
C 3.098094 0.645944 -1.421027
O 0.895026 1.813580 -2.187882
C 0.386266 -0.791102 3.729483
H 1.243744 -1.453312 3.845784
C 5.487129 0.697840 -1.257483
H 6.391973 1.136325 -0.837870
C 0.844326 -3.204075 -0.490712
H 1.330798 -2.589875 -1.247540
C 0.486488 -2.619862 0.728806
C 3.573899 -1.727055 0.973558
H 3.239343 -2.568834 0.372156
C 4.919018 -1.601311 1.295338
H 5.629356 -2.345576 0.941133
C 4.444786 0.427106 2.507197
H 4.784796 1.274549 3.098971
C 3.098016 0.311066 2.186053
H 2.390501 1.073710 2.502090
C 0.024161 -0.329161 2.459550
C 3.175354 -0.409978 -2.323688
H 2.259330 -0.824155 -2.742392
C -0.180381 -3.380745 1.688005
H -0.491228 -2.931063 2.627323
C 2.657768 -0.775152 1.425285
C 6.928985 -0.970003 -2.477511
H 6.979312 -1.267089 -3.531407
H 7.126765 -1.870671 -1.879007
H 7.741844 -0.262517 -2.282222
C 5.354054 -0.528145 2.066495
H 6.408566 -0.432666 2.318848
C 0.545927 -0.045447 -0.491476
P 0.903781 -0.863813 0.957190
O 1.767491 2.214934 0.168099
S 1.493404 1.291519 -0.945833
C 5.593481 -0.381222 -2.134107
C 4.416878 -0.920447 -2.669034
H 4.480567 -1.750927 -3.371588
C 0.558173 -4.539802 -0.735371
H 0.838750 -4.987507 -1.686560

C -0.102810 -5.297554 0.227223
H -0.338430 -6.341754 0.030691
C -0.474490 -4.716370 1.433667
H -1.005840 -5.301870 2.181209
C -1.433818 0.459107 4.695691
H -2.006266 0.762364 5.570387
C -0.342798 -0.395774 4.843224
H -0.059213 -0.753043 5.831330
C -1.782959 0.933322 3.437042
H -2.629437 1.606462 3.313032
C -1.046377 0.548894 2.320689
H -1.287526 0.941611 1.336640
N -4.207138 0.714913 -0.370440
C -3.927902 2.685443 0.641153
C -4.786375 1.655529 0.472601
H -4.078230 3.615030 1.178307
H -5.800780 1.548315 0.838830
B -2.886698 1.171806 -0.735286
C -1.684939 3.361391 -0.060036
C -1.073960 3.744082 -1.249082
C -1.245018 3.903545 1.149629
C -0.026449 4.655393 -1.229236
H -1.413775 3.313309 -2.187003
C -0.206738 4.823496 1.160461
H -1.709430 3.587596 2.081358
C 0.404419 5.203970 -0.029385
H 0.462040 4.921648 -2.163385
H 0.137356 5.232846 2.108288
H 1.229147 5.913358 -0.016992
C -4.838154 -0.496944 -0.702556
C -4.746070 -0.998852 -2.002258
C -5.556562 -1.213566 0.256654
C -5.342694 -2.209550 -2.328585
H -4.216709 -0.420979 -2.758021
C -6.166082 -2.414887 -0.081348
H -5.611948 -0.833968 1.274727
C -6.058224 -2.921948 -1.372244
H -5.258076 -2.590741 -3.344345
H -6.719362 -2.966257 0.676718
H -6.531612 -3.866395 -1.632011
N -2.764722 2.445852 -0.079888
Sn -1.312524 -0.216387 -1.570738
H -1.861109 -1.800434 -1.170508
H -1.023125 -0.202128 -3.253735

$^{Ph}Y_2Sn$
E = -2620.57497065
P 3.139620 0.218713 -0.044724
P -3.196663 0.297430 0.020199
C 1.646447 -0.047985 -0.797252
C 1.287517 -1.377972 -1.291400
C 1.810406 -2.574774 -0.767252
H 2.479631 -2.531052 0.089388
C 1.448299 -3.814898 -1.274550
H 1.867667 -4.715023 -0.827601
C 0.531671 -3.916496 -2.315499
H 0.236293 -4.889652 -2.702317
C -0.003332 -2.748004 -2.851153
H -0.714617 -2.803880 -3.674988
C 0.373321 -1.507683 -2.356098

H -0.027268 -0.599901 -2.809322
C 3.399794 -0.320842 1.698017
C 4.036158 -1.520422 2.025218
H 4.507287 -2.116377 1.245410
C 4.076671 -1.961144 3.344092
H 4.580096 -2.895662 3.584279
C 3.474500 -1.213864 4.349802
H 3.506693 -1.559052 5.381395
C 2.819003 -0.027292 4.030599
H 2.334233 0.556245 4.810854
C 2.778325 0.413271 2.714434
H 2.253747 1.336901 2.467795
C 3.503991 2.005661 -0.014679
C 4.329835 2.580223 0.953416
H 4.722270 1.973621 1.767036
C 4.647782 3.932131 0.887702
H 5.286061 4.373252 1.650834
C 4.149780 4.718061 -0.145949
H 4.396059 5.777269 -0.192119
C 3.344009 4.145792 -1.124769
H 2.960650 4.752972 -1.942386
C 3.030177 2.793768 -1.066587
H 2.416295 2.332454 -1.838742
C 4.549105 -0.499628 -0.956027
C 4.363914 -0.967103 -2.257412
H 3.365928 -0.969779 -2.688032
C 5.453116 -1.422782 -2.991628
H 5.301012 -1.797602 -4.001805
C 6.729902 -1.397810 -2.440985
H 7.580346 -1.753346 -3.019930
C 6.921878 -0.909739 -1.151853
H 7.921215 -0.875666 -0.722260
C 5.835351 -0.460776 -0.411635
H 5.988248 -0.074118 0.595377
C -1.567268 -0.039055 0.301589
C -1.147377 -1.319663 0.883717
C -0.144357 -1.339926 1.869468
H 0.261287 -0.390267 2.213860
C 0.323848 -2.523913 2.417977
H 1.111522 -2.488019 3.169169
C -0.218409 -3.743582 2.018658
H 0.141511 -4.676252 2.449186
C -1.219905 -3.752131 1.053831
H -1.641157 -4.697326 0.714086
C -1.665248 -2.564066 0.485657
H -2.397784 -2.599850 -0.320121
C -4.273800 -0.238479 1.397242
C -4.505306 0.599036 2.490344
H -4.124676 1.617508 2.487460
C -5.230689 0.142632 3.583607
H -5.414474 0.808366 4.424694
C -5.718836 -1.160331 3.603748
H -6.281509 -1.518755 4.463726
C -5.487405 -2.002051 2.522405
H -5.863387 -3.023163 2.533033
C -4.774104 -1.543478 1.420547
H -4.610951 -2.203808 0.572185
C -3.390124 2.097739 -0.190787
C -4.122665 2.663816 -1.235043
H -4.620236 2.026323 -1.961142

C -4.200981 4.046374 -1.365002
H -4.766327 4.477278 -2.188999
C -3.552009 4.874561 -0.456503
H -3.612260 5.955864 -0.565037
C -2.818519 4.318190 0.586360
H -2.300552 4.959573 1.296496
C -2.735862 2.938038 0.718403
H -2.143759 2.497460 1.519153
C -4.063542 -0.428192 -1.431533
C -5.456090 -0.418877 -1.558713
H -6.071461 0.013028 -0.769784
C -6.054397 -0.966496 -2.686398
H -7.138489 -0.958446 -2.784355
C -5.267423 -1.532475 -3.687814
H -5.739050 -1.965945 -4.567939
C -3.883531 -1.553151 -3.558757
H -3.268943 -2.005647 -4.334766
C -3.282475 -1.001965 -2.430888
H -2.202217 -1.029327 -2.294695
Sn 0.001652 1.252808 -0.345756
H 0.498648 2.374192 0.875468
H -0.491427 2.209553 -1.692921

^{Ph}YSnCl
E = -1773.18870127
P -0.773220 -0.309819 0.109710
C 0.441587 0.499343 -0.775180
C 0.362558 1.957928 -0.909743
C -0.864998 2.652605 -0.905381
H -1.798882 2.099640 -0.815444
C -0.928159 4.035481 -1.012086
H -1.901350 4.524263 -1.000052
C 0.230841 4.792057 -1.140058
H 0.181265 5.875079 -1.230021
C 1.456941 4.133700 -1.135381
H 2.382555 4.702491 -1.209898
C 1.521834 2.753903 -1.011054
H 2.504539 2.287826 -0.947816
C -1.204261 0.632328 1.611665
C -2.498131 0.775625 2.113180
H -3.342433 0.321555 1.601448
C -2.718428 1.527130 3.262298
H -3.730328 1.639575 3.646581
C -1.652780 2.140969 3.911160
H -1.829836 2.731719 4.807959
C -0.363178 2.013750 3.404598
H 0.471245 2.507821 3.897810
C -0.136650 1.270243 2.253994
H 0.867301 1.194564 1.833975
C -0.157647 -1.940801 0.612485
C 0.766311 -2.020089 1.658005
H 1.018927 -1.134262 2.236241
C 1.402794 -3.223728 1.931038
H 2.135541 -3.272943 2.733153
C 1.116657 -4.352100 1.169034
H 1.622920 -5.292204 1.379467
C 0.188561 -4.279591 0.135741
H -0.032183 -5.160516 -0.463565
C -0.448224 -3.076656 -0.146392
H -1.155524 -3.017749 -0.970480

C -2.331060 -0.676440 -0.766863
C -2.483070 -0.199087 -2.068111
H -1.683040 0.397624 -2.506157
C -3.635190 -0.497333 -2.788934
H -3.752472 -0.118525 -3.802206
C -4.629176 -1.284073 -2.217959
H -5.528037 -1.520657 -2.784216
C -4.471863 -1.781946 -0.926523
H -5.242334 -2.412089 -0.486547
C -3.325130 -1.482619 -0.202300
H -3.191492 -1.894307 0.797737
Cl 3.625044 -0.003464 0.846945
Sn 2.255678 -0.479991 -1.110276
H 3.134276 0.200317 -2.399102
H 2.217183 -2.177093 -1.192200

^{Ph}YSnHMDS
E = -2186.97193692
P 2.015452 0.082837 -0.040123
C 0.396828 -0.311954 -0.358980
C -0.011035 -1.697045 -0.625916
C 0.647363 -2.817040 -0.081301
H 1.499894 -2.670813 0.579116
C 0.217982 -4.112855 -0.340557
H 0.753024 -4.950225 0.105222
C -0.894347 -4.345044 -1.142803
H -1.237797 -5.359412 -1.334971
C -1.551220 -3.254208 -1.707417
H -2.409799 -3.414315 -2.358726
C -1.110531 -1.961324 -1.465161
H -1.614185 -1.122185 -1.936604
C 2.771234 -0.578236 1.503134
C 4.102407 -0.976009 1.637070
H 4.784005 -0.910113 0.792912
C 4.560452 -1.481950 2.849372
H 5.598208 -1.796050 2.943978
C 3.696939 -1.594515 3.933842
H 4.059236 -1.994026 4.879172
C 2.365585 -1.211319 3.803358
H 1.680341 -1.312396 4.642565
C 1.903306 -0.715148 2.591260
H 0.854303 -0.443898 2.471262
C 2.099615 1.888425 0.125215
C 2.084556 2.520994 1.368475
H 2.144695 1.932809 2.281803
C 1.978779 3.907243 1.438401
H 1.955744 4.397602 2.409275
C 1.899987 4.659950 0.272899
H 1.814760 5.743339 0.331274
C 1.928799 4.031412 -0.969651
H 1.865550 4.621086 -1.881783
C 2.022976 2.649306 -1.045823
H 2.021020 2.148728 -2.012720
C 3.189249 -0.325679 -1.370553
C 2.818535 -1.220415 -2.375533
H 1.837677 -1.687911 -2.348008
C 3.700584 -1.499977 -3.413554
H 3.408622 -2.202741 -4.191232
C 4.943580 -0.878143 -3.464986
H 5.628029 -1.095404 -4.282867

C 5.307135 0.033736 -2.478604
H 6.271029 0.536704 -2.526236
C 4.433120 0.310978 -1.434431
H 4.711088 1.039883 -0.673583
N -2.945568 0.312113 0.048544
Si -3.871113 0.788749 -1.353555
Si -3.571593 -0.657562 1.366781
C -2.755765 1.710190 -2.563021
H -2.468304 2.703761 -2.200212
H -1.832648 1.153392 -2.781658
H -3.289082 1.844227 -3.513285
C -4.606940 -0.661543 -2.308246
H -5.525409 -1.043145 -1.850019
H -4.859172 -0.332191 -3.325305
H -3.901616 -1.496861 -2.389602
C -5.286392 1.921435 -0.849822
H -5.890976 2.235374 -1.710978
H -5.953564 1.411736 -0.141231
H -4.903653 2.822086 -0.352489
C -5.057061 -1.682411 0.846489
H -5.404836 -2.263037 1.711369
H -5.897640 -1.066559 0.502669
H -4.795335 -2.388184 0.048990
C -4.112589 0.462086 2.780527
H -4.512516 -0.112159 3.626866
H -3.273586 1.066113 3.149056
H -4.894454 1.153223 2.438169
C -2.262138 -1.854149 1.993331
H -1.291404 -1.376191 2.181665
H -2.591949 -2.308076 2.937605
H -2.091197 -2.659566 1.267558
Sn -1.045815 1.090231 0.358915
H -0.959648 2.655579 -0.314198
H -0.938561 1.263530 2.074197

$^{Ph}YSnC_6F_5$
E = -2041.51632429
P -1.869681 0.220390 0.041877
C -0.378826 0.057907 -0.731644
C 0.586179 1.179910 -0.788661
C 1.073580 1.822999 0.362789
H 0.690892 1.520205 1.337128
C 2.053371 2.806462 0.288375
H 2.413807 3.277283 1.201902
C 2.591873 3.169453 -0.942660
H 3.374324 3.923382 -1.001101
C 2.121910 2.550715 -2.096721
H 2.534132 2.822478 -3.066927
C 1.127136 1.583000 -2.019539
H 0.765528 1.101143 -2.926450
C -1.958125 0.273440 1.878168
C -3.019557 0.865092 2.568647
H -3.826517 1.347371 2.018681
C -3.042627 0.843738 3.958603
H -3.868457 1.308767 4.493835
C -2.008704 0.234669 4.665230
H -2.027208 0.224892 5.753479
C -0.949429 -0.353354 3.981869
H -0.134359 -0.824892 4.527225
C -0.924653 -0.331955 2.591921

H -0.089136 -0.775091 2.052697
C -2.903035 -1.202306 -0.421805
C -3.689968 -1.891049 0.502019
H -3.709779 -1.577121 1.542910
C -4.435268 -2.993113 0.096691
H -5.038311 -3.531976 0.824671
C -4.402731 -3.412325 -1.228612
H -4.983634 -4.278407 -1.539900
C -3.624398 -2.726136 -2.155992
H -3.596631 -3.050252 -3.194373
C -2.877192 -1.624837 -1.756751
H -2.258967 -1.083082 -2.471639
C -2.726453 1.750935 -0.443122
C -2.285524 2.965070 0.093089
H -1.504528 2.974578 0.851172
C -2.844025 4.161347 -0.339584
H -2.490085 5.102651 0.075939
C -3.852356 4.154735 -1.297973
H -4.292048 5.092535 -1.632127
C -4.300339 2.948730 -1.826652
H -5.093530 2.939771 -2.571653
C -3.736617 1.749539 -1.406074
H -4.088432 0.809734 -1.826619
C 2.482250 -1.343686 -0.295496
C 3.799181 -0.557808 1.594021
C 4.394718 0.121738 -0.637397
C 4.625039 0.153565 0.731976
Sn 0.481087 -1.860286 -1.018328
H 0.613087 -2.383728 -2.639231
H -0.187685 -3.108464 -0.064479
C 3.340778 -0.639594 -1.124315
C 2.746121 -1.289011 1.061750
F 1.936310 -1.912413 1.928088
F 3.138806 -0.632017 -2.441765
F 5.160782 0.827914 -1.451807
F 5.623727 0.869769 1.217479
F 4.017545 -0.518868 2.900250

^{Ph}YSnPy
E = -1560.65390547
P -1.362034 0.065490 0.002074
C 0.274563 -0.014709 -0.362775
C 1.089711 1.227038 -0.311929
C 1.792060 1.606718 0.842650
C 2.615314 2.726569 0.858776
C 2.742642 3.515095 -0.280478
C 2.037230 3.171786 -1.429784
C 1.227036 2.042886 -1.443763
C -1.859105 0.230536 1.769766
C -3.057224 0.826518 2.171444
H -3.737611 1.235382 1.425757
C -3.378931 0.905393 3.521901
H -4.311066 1.375112 3.830714
C -2.507808 0.390040 4.477711
H -2.759487 0.457096 5.534598
C -1.313472 -0.204199 4.082946
H -0.629872 -0.604713 4.829184
C -0.989556 -0.279824 2.733063
H -0.052825 -0.732736 2.408063
C -2.196661 -1.444341 -0.566140

C -3.133795 -2.128269 0.210379
H -3.389046 -1.763655 1.202714
C -3.731934 -3.284013 -0.279639
H -4.455684 -3.817513 0.333313
C -3.401695 -3.761426 -1.543514
H -3.869390 -4.668728 -1.921328
C -2.470384 -3.082254 -2.322885
H -2.206603 -3.454672 -3.310623
C -1.868627 -1.927520 -1.838049
H -1.130640 -1.391560 -2.433255
C -2.186703 1.501025 -0.758282
C -1.854831 2.776062 -0.287702
H -1.175789 2.884043 0.557225
C -2.381691 3.904402 -0.902435
H -2.111586 4.893079 -0.536873
C -3.248873 3.770104 -1.982663
H -3.663143 4.655332 -2.461613
C -3.587109 2.504319 -2.448894
H -4.269418 2.396086 -3.289854
C -3.055431 1.371066 -1.842357
H -3.321230 0.383146 -2.213341
C 3.358455 -1.121897 -0.050791
C 5.224682 -0.508983 1.334374
C 4.944747 0.308994 -0.890185
C 5.681570 0.284482 0.290746
N 3.820229 -0.374184 -1.061138
H 5.774943 -0.563155 2.272978
H 6.591194 0.874140 0.383972
H 5.277589 0.916978 -1.734250
H 1.701323 0.989062 1.735465
H 3.161215 2.983264 1.765445
H 3.388179 4.391728 -0.272825
H 2.128578 3.781627 -2.327442
H 0.691072 1.761611 -2.348549
C 4.044484 -1.223175 1.161513
H 3.650889 -1.842724 1.966654
Sn 1.348029 -1.847275 -0.407751
H 1.290158 -2.673154 -1.909498
H 0.863745 -2.958921 0.819758

^{Ph}YSnPyr
E = -1576.72065164
P -1.339012 0.047870 0.016719
C 0.186691 0.051249 -0.718780
C 0.937496 1.291094 -0.909335
C 0.680367 2.478755 -0.195871
C 1.430708 3.630792 -0.392758
C 2.492541 3.643669 -1.290382
C 2.780157 2.478934 -1.998228
C 2.014825 1.336744 -1.819803
C -1.433337 0.338195 1.825137
C -2.636442 0.522113 2.515620
H -3.582041 0.522519 1.973598
C -2.628269 0.712527 3.891454
H -3.565765 0.852818 4.426411
C -1.418966 0.727739 4.583511
H -1.413676 0.879637 5.661439
C -0.221726 0.556588 3.898692
H 0.724264 0.575993 4.436329
C -0.223173 0.362987 2.519993

H 0.718986 0.242245 1.979187
C -2.075383 -1.598904 -0.255439
C -2.777832 -2.290310 0.732131
H -2.906564 -1.855285 1.719249
C -3.291860 -3.555698 0.469278
H -3.829298 -4.089464 1.250573
C -3.109117 -4.142725 -0.777287
H -3.508592 -5.135230 -0.976744
C -2.405378 -3.462176 -1.765567
H -2.249748 -3.918790 -2.740904
C -1.890294 -2.198159 -1.507605
H -1.316939 -1.670205 -2.268612
C -2.478907 1.289239 -0.696949
C -2.813367 2.474319 -0.039409
H -2.443508 2.665370 0.965526
C -3.613787 3.422864 -0.667217
H -3.861970 4.346337 -0.147819
C -4.087916 3.195626 -1.953565
H -4.714931 3.938528 -2.442905
C -3.752987 2.019409 -2.617393
H -4.116444 1.838362 -3.626912
C -2.947774 1.075098 -1.995252
H -2.678300 0.164193 -2.524762
C 3.225531 -0.903203 0.160856
C 4.168120 0.577040 1.614789
C 5.441688 -0.454209 -0.080069
C 5.395862 0.441546 0.979295
N 4.368781 -1.121513 -0.491461
N 3.093767 -0.090932 1.216424
H 4.046208 1.248049 2.467971
H 6.271910 1.001279 1.297735
H 6.373439 -0.636367 -0.621010
H -0.102806 2.491992 0.559484
H 1.192113 4.525014 0.181919
H 3.087771 4.542999 -1.434702
H 3.605418 2.461580 -2.708939
H 2.249598 0.446227 -2.404447
Sn 1.356301 -1.728327 -0.596150
H 1.658864 -2.501563 -2.096172
H 0.709453 -2.877675 0.497376

^{Ph}YSnB2
E = -1719.67372922
P -1.724234 0.099218 0.133038
C -0.396055 -0.016966 -0.891374
C 0.435622 1.202205 -1.084176
C 1.455598 1.562251 -0.187214
C 2.242515 2.690914 -0.389850
C 2.015266 3.509138 -1.491704
C 0.997397 3.184543 -2.383496
C 0.224181 2.046457 -2.183944
C -1.440754 0.138260 1.955451
C -2.339309 0.727638 2.849181
H -3.245997 1.201248 2.474692
C -2.075428 0.717981 4.214165
H -2.777304 1.181035 4.905444
C -0.912109 0.123292 4.695439
H -0.704803 0.121462 5.764027
C -0.012085 -0.460379 3.810250
H 0.902125 -0.920627 4.180308

C -0.274476 -0.450000 2.444774
H 0.438762 -0.891138 1.748728
C -2.844433 -1.306317 -0.130297
C -3.500415 -1.954276 0.916821
H -3.342565 -1.630158 1.942845
C -4.344008 -3.027456 0.650815
H -4.846411 -3.534451 1.472230
C -4.537631 -3.457932 -0.657178
H -5.195062 -4.301174 -0.860713
C -3.886818 -2.813771 -1.705234
H -4.033495 -3.150185 -2.729687
C -3.042937 -1.741310 -1.445083
H -2.518482 -1.232049 -2.252726
C -2.660089 1.636650 -0.144232
C -2.080457 2.847275 0.249635
H -1.127721 2.847993 0.777731
C -2.713335 4.049125 -0.040245
H -2.251866 4.987480 0.260748
C -3.930694 4.052036 -0.714494
H -4.427081 4.994590 -0.937818
C -4.513442 2.850148 -1.101987
H -5.467720 2.849323 -1.625269
C -3.879090 1.644254 -0.822908
H -4.336070 0.706426 -1.132895
H 1.621936 0.941896 0.692182
H 3.033688 2.930631 0.319308
H 2.627272 4.394513 -1.654555
H 0.807292 3.820969 -3.246562
H -0.568531 1.788986 -2.884171
Sn 0.552652 -1.898452 -1.237857
H 0.474742 -2.383503 -2.884120
H -0.153376 -3.144490 -0.292727
C 4.604821 -0.409861 -0.482099
C 4.210827 -0.705710 0.819013
C 5.783276 0.256322 -0.755093
C 4.974885 -0.351972 1.913862
C 6.563234 0.619871 0.344144
H 6.075133 0.486367 -1.776005
C 6.168333 0.322593 1.649354
H 4.653423 -0.586750 2.925075
H 7.498967 1.149566 0.178857
H 6.802787 0.624574 2.479878
B 2.650896 -1.410552 -0.564949
O 3.007071 -1.345804 0.777768
O 3.653236 -0.863628 -1.344841

^{Ph}*YSnB1*
E = -1988.86624564
P 2.004101 -0.634482 0.156958
C 0.371717 -0.225415 0.365033
C -0.077397 0.495513 1.555379
C 0.753005 1.364171 2.300215
H 1.784846 1.516325 1.992441
C 0.289643 2.053167 3.413224
H 0.973109 2.709952 3.950367
C -1.031416 1.928821 3.834121
H -1.396303 2.479950 4.698356
C -1.867134 1.060553 3.135057
H -2.898454 0.914955 3.454841
C -1.395661 0.347915 2.042024

H -2.057841 -0.369257 1.560311
C 3.209421 0.742675 0.003931
C 4.558104 0.640198 0.349783
H 4.944490 -0.275574 0.789855
C 5.413622 1.716890 0.145758
H 6.463675 1.632165 0.419321
C 4.927044 2.899239 -0.402391
H 5.597932 3.741412 -0.562241
C 3.580084 3.010367 -0.731865
H 3.192290 3.938033 -1.144243
C 2.718371 1.939726 -0.523025
H 1.653130 2.025795 -0.746458
C 2.199808 -1.570745 -1.384929
C 2.819432 -1.027257 -2.509300
H 3.254361 -0.031483 -2.464192
C 2.866127 -1.754662 -3.693582
H 3.343571 -1.324955 -4.571861
C 2.299491 -3.022651 -3.757408
H 2.334041 -3.587256 -4.687275
C 1.689130 -3.572069 -2.633501
H 1.245698 -4.564447 -2.681012
C 1.640366 -2.850828 -1.448057
H 1.157367 -3.272272 -0.567368
C 2.703027 -1.741759 1.435025
C 2.076536 -1.841787 2.678966
H 1.190780 -1.247119 2.886750
C 2.576456 -2.711386 3.641599
H 2.084936 -2.779571 4.610058
C 3.689628 -3.498710 3.366316
H 4.073581 -4.183375 4.120329
C 4.302433 -3.422291 2.119663
H 5.161131 -4.051188 1.892226
C 3.810873 -2.548874 1.156373
H 4.280219 -2.508437 0.173975
N -4.028419 0.391244 -0.169869
C -3.729756 2.562954 0.271020
C -4.568085 1.517569 0.441721
H -3.860463 3.596604 0.570125
H -5.548653 1.502272 0.903211
B -2.750829 0.746795 -0.734339
N -2.622242 2.149404 -0.456764
C -1.522621 2.997490 -0.689614
C -0.976867 3.735195 0.361863
C -0.989473 3.120942 -1.973290
C 0.055091 4.631511 0.116068
H -1.358805 3.580829 1.369445
C 0.048049 4.014845 -2.210491
H -1.417822 2.534820 -2.783571
C 0.556618 4.789801 -1.172445
H 0.473171 5.205279 0.940791
H 0.445480 4.120447 -3.218202
H 1.346688 5.512895 -1.366042
C -4.592509 -0.888932 -0.032009
C -4.583850 -1.778912 -1.107398
C -5.151861 -1.290727 1.183651
C -5.115366 -3.053276 -0.963329
H -4.164090 -1.455550 -2.057134
C -5.696051 -2.562162 1.315501
H -5.135319 -0.606753 2.030072
C -5.678074 -3.450367 0.245472

H -5.098870 -3.738122 -1.808956
H -6.125271 -2.864427 2.268945
H -6.100037 -4.447348 0.353011
Sn -1.017919 -0.594732 -1.240201
H -1.489215 -2.250019 -1.325800
H -0.273856 -0.259697 -2.757039

$^{F}Y_2Sn$
E = -3614.39676385
P 3.175888 0.668661 -0.094724
P -3.154590 0.851761 0.062710
C 1.654747 0.303708 -0.756743
C 1.234765 -1.014430 -1.182151
C 1.622912 -2.233524 -0.606589
C 1.112062 -3.460884 -0.998162
C 0.159563 -3.534609 -2.006878
C -0.244258 -2.357183 -2.623502
C 0.305201 -1.149817 -2.228725
C 3.322830 0.777000 1.725057
C 3.539454 -0.367243 2.498346
H 3.765276 -1.315968 2.020225
C 3.454103 -0.302280 3.882829
H 3.617706 -1.202302 4.471172
C 3.132961 0.895236 4.510874
H 3.054260 0.938363 5.595469
C 2.907480 2.035743 3.748831
H 2.651833 2.976422 4.232660
C 3.003925 1.979938 2.364661
H 2.818952 2.877113 1.779106
C 3.716297 2.319241 -0.665664
C 4.806130 2.952728 -0.061576
H 5.288619 2.498826 0.802893
C 5.272991 4.162371 -0.556824
H 6.120718 4.651002 -0.080591
C 4.656448 4.748994 -1.659250
H 5.020668 5.699774 -2.043952
C 3.581813 4.115814 -2.271929
H 3.103397 4.565447 -3.139691
C 3.117666 2.897907 -1.783925
H 2.296377 2.378274 -2.275361
C 4.496624 -0.432545 -0.681646
C 4.372482 -1.004411 -1.949997
H 3.478430 -0.816622 -2.540009
C 5.393197 -1.799988 -2.455446
H 5.287733 -2.249822 -3.440584
C 6.545890 -2.017881 -1.707725
H 7.342861 -2.643237 -2.105408
C 6.682318 -1.429738 -0.455140
H 7.587003 -1.588982 0.128165
C 5.662653 -0.637006 0.058338
H 5.777082 -0.178137 1.038048
C -1.522643 0.447089 0.290411
C -1.152129 -0.823383 0.896670
C -0.203630 -0.891731 1.932145
C 0.197754 -2.088901 2.503551
C -0.331538 -3.292115 2.050691
C -1.281331 -3.271711 1.040882
C -1.656940 -2.061284 0.479892
C -4.182873 0.218939 1.431057
C -4.349268 0.963145 2.600845

H -3.949094 1.971852 2.668427
C -5.034462 0.422961 3.681778
H -5.166931 1.014130 4.585744
C -5.547948 -0.868211 3.609216
H -6.079712 -1.291704 4.459127
C -5.381168 -1.615664 2.449094
H -5.778174 -2.626940 2.387402
C -4.705840 -1.075736 1.360029
H -4.583480 -1.662772 0.452731
C -3.276556 2.670391 0.039510
C -4.047281 3.362066 -0.896467
H -4.608692 2.817024 -1.650862
C -4.082392 4.752060 -0.880028
H -4.677783 5.283225 -1.619915
C -3.352626 5.461085 0.067538
H -3.379454 6.549115 0.073111
C -2.581531 4.778520 1.002823
H -2.002038 5.327542 1.742147
C -2.540639 3.390071 0.988983
H -1.922649 2.849261 1.704577
C -4.068681 0.308847 -1.426684
C -5.465185 0.341684 -1.485004
H -6.040523 0.679240 -0.623390
C -6.120051 -0.068874 -2.639709
H -7.207386 -0.046477 -2.683123
C -5.386117 -0.520648 -3.734416
H -5.902768 -0.852778 -4.633112
C -3.997697 -0.559901 -3.675142
H -3.415555 -0.930311 -4.516194
C -3.340769 -0.141993 -2.523505
H -2.258011 -0.187262 -2.459724
Sn 0.058979 1.706160 -0.404694
H 0.613078 2.869338 0.733920
H -0.453004 2.593362 -1.781499
F 2.471946 -2.228300 0.426235
F 1.471827 -4.561269 -0.353221
F -0.355875 -4.700157 -2.365910
F 0.059504 -4.437571 2.586866
F -1.765248 -4.407685 0.559587
F -1.144821 -2.389811 -3.597957
F -2.512290 -2.107065 -0.547427
F -0.094296 -0.048917 -2.877770
F 0.321732 0.237316 2.407523
F 1.074248 -2.108027 3.498300

^{F}YSnCl
E = -2270.09241554
P -0.766978 0.826170 0.182752
C 0.105514 -0.495197 -0.463293
C 1.559530 -0.480140 -0.464935
C 2.325464 0.666689 -0.723320
C 3.710545 0.690663 -0.672370
C 4.409624 -0.467466 -0.360650
C 3.698555 -1.634412 -0.106793
C 2.313170 -1.620444 -0.138976
C 0.229474 1.647806 1.459752
C 0.344020 3.034251 1.559210
H -0.179882 3.679432 0.858657
C 1.152281 3.593910 2.542069
H 1.241578 4.675888 2.613582

C 1.853658 2.776390 3.421552
H 2.489386 3.219206 4.185789
C 1.755387 1.392596 3.315162
H 2.315631 0.749468 3.990594
C 0.952353 0.827253 2.333267
H 0.891936 -0.254884 2.223931
C -2.299506 0.214953 0.943266
C -2.303939 -0.238663 2.263823
H -1.420631 -0.121836 2.886354
C -3.435689 -0.849522 2.784364
H -3.431215 -1.203372 3.812948
C -4.563316 -1.025611 1.989659
H -5.442695 -1.521435 2.395488
C -4.565918 -0.572430 0.676103
H -5.445925 -0.708767 0.051186
C -3.442215 0.056913 0.153816
H -3.451465 0.414726 -0.873951
C -1.361753 2.106971 -0.971200
C -0.927614 2.080223 -2.294024
H -0.212763 1.316951 -2.596784
C -1.404300 3.019761 -3.201568
H -1.062279 2.997367 -4.234244
C -2.317309 3.984068 -2.789550
H -2.691095 4.718163 -3.500827
C -2.762714 4.007729 -1.469915
H -3.485352 4.756021 -1.150517
C -2.292186 3.067910 -0.562289
H -2.657565 3.071691 0.464475
Cl -1.936662 -3.430680 0.625374
Sn -0.953431 -2.170572 -1.168172
H 0.071132 -3.215569 -2.027465
H -2.306606 -1.719046 -2.102735
F 4.368275 1.809944 -0.933695
F 5.733169 -0.464003 -0.317173
F 4.347502 -2.744081 0.209029
F 1.677461 -2.748764 0.179024
F 1.704546 1.802282 -1.059353

^{F}*YSnHMDS*
E = -2683.88011039
P -2.132121 -0.393743 -0.114253
C -0.471343 -0.081544 -0.008687
C 0.038617 1.278314 -0.160137
C -0.375310 2.363510 0.619136
C 0.153625 3.638731 0.485262
C 1.162202 3.866818 -0.442349
C 1.603528 2.817906 -1.241294
C 1.022017 1.565436 -1.116634
C -3.151062 -0.397486 1.405919
C -4.548525 -0.378496 1.362895
H -5.063919 -0.335602 0.403605
C -5.280144 -0.401459 2.543701
H -6.367735 -0.382323 2.508616
C -4.621373 -0.441375 3.770433
H -5.196509 -0.455981 4.694336
C -3.231882 -0.450032 3.816464
H -2.716182 -0.467498 4.774365
C -2.497401 -0.423204 2.636091
H -1.408783 -0.398363 2.658688
C -2.353188 -2.053769 -0.835133

C -3.292395 -2.978613 -0.376392
H -3.944393 -2.730948 0.457516
C -3.381812 -4.232043 -0.973321
H -4.109182 -4.952543 -0.604767
C -2.539474 -4.568449 -2.027420
H -2.610065 -5.552712 -2.486554
C -1.598467 -3.651612 -2.486333
H -0.925591 -3.913917 -3.300280
C -1.502536 -2.401837 -1.890546
H -0.747564 -1.686015 -2.213609
C -2.950751 0.826723 -1.186522
C -3.454991 2.007231 -0.633948
H -3.436051 2.155024 0.443449
C -3.970331 2.997528 -1.462524
H -4.354873 3.917431 -1.026799
C -3.992146 2.813798 -2.840568
H -4.398280 3.589447 -3.486944
C -3.493549 1.638255 -3.393379
H -3.511217 1.490295 -4.471190
C -2.969266 0.648755 -2.571197
H -2.578343 -0.266684 -3.009341
N 2.808780 -0.888747 0.347926
Si 3.685682 -1.467317 -1.056364
Si 3.575986 -0.034957 1.671948
C 2.515907 -2.386964 -2.213364
H 2.159188 -3.334925 -1.793348
H 1.645629 -1.772273 -2.475848
H 3.049956 -2.616747 -3.144843
C 4.513676 -0.098358 -2.047323
H 5.466279 0.213612 -1.605834
H 4.729247 -0.470105 -3.058374
H 3.874983 0.785547 -2.146367
C 5.026558 -2.675686 -0.517037
H 5.576269 -3.081115 -1.376939
H 5.757494 -2.187845 0.141788
H 4.591884 -3.517716 0.037525
C 5.107678 0.892971 1.108621
H 5.516485 1.452717 1.960436
H 5.898680 0.228579 0.739925
H 4.866537 1.611428 0.315389
C 4.072114 -1.235301 3.032830
H 4.560973 -0.719929 3.870197
H 3.197522 -1.766312 3.430015
H 4.769795 -1.987975 2.642861
C 2.397482 1.251010 2.378062
H 1.386446 0.868529 2.572782
H 2.787670 1.644928 3.325784
H 2.306258 2.091703 1.678977
Sn 0.882785 -1.555260 0.721359
H 0.625430 -3.106941 0.067567
H 0.792734 -1.689793 2.435637
F 1.404147 0.619970 -1.967972
F 2.546014 3.034392 -2.149167
F 1.688573 5.073610 -0.574820
F -0.272522 4.629961 1.254364
F -1.288188 2.171250 1.573363

$^{F}YSnC_6F_5$
E = -2538.42233005
P -2.020513 0.038533 0.313493

C -0.683644 0.353391 -0.670400
C 0.349307 1.312579 -0.258382
C 1.260233 1.056055 0.770137
C 2.353532 1.868075 1.029093
C 2.540105 3.019172 0.276679
C 1.634771 3.336140 -0.729267
C 0.558358 2.496075 -0.976636
C -1.942556 -1.278016 1.583666
C -2.845545 -1.341385 2.649339
H -3.618293 -0.580600 2.757147
C -2.747482 -2.368963 3.579475
H -3.448163 -2.415530 4.410987
C -1.749344 -3.333406 3.454109
H -1.673317 -4.133108 4.188464
C -0.844555 -3.268161 2.400497
H -0.051329 -4.006156 2.297612
C -0.942424 -2.238586 1.472104
H -0.220881 -2.158791 0.665684
C -3.430850 -0.407103 -0.745762
C -4.357637 -1.395601 -0.411164
H -4.253331 -1.952500 0.516769
C -5.406222 -1.685173 -1.277188
H -6.120210 -2.463985 -1.017086
C -5.536974 -0.990869 -2.475200
H -6.357592 -1.223447 -3.151257
C -4.614590 -0.005288 -2.813685
H -4.712144 0.536272 -3.752488
C -3.560898 0.284736 -1.955610
H -2.818274 1.040401 -2.210492
C -2.412921 1.520536 1.282033
C -1.663264 1.760248 2.437595
H -0.946021 1.015926 2.782089
C -1.835098 2.945142 3.142147
H -1.245780 3.131889 4.037518
C -2.758982 3.887985 2.702410
H -2.895787 4.814301 3.256992
C -3.508252 3.649090 1.554829
H -4.231965 4.385794 1.212185
C -3.332820 2.470114 0.838767
H -3.913086 2.288460 -0.063832
C 1.942057 -1.499451 -1.262690
C 3.210243 -2.672718 0.453826
C 3.975228 -0.531250 -0.327412
C 4.127928 -1.628891 0.509395
C 2.896845 -0.497537 -1.201386
C 2.147532 -2.591844 -0.435535
F 1.268345 -3.598867 -0.421736
F 2.759006 0.603610 -1.945143
F 4.821980 0.482684 -0.262338
F 5.138361 -1.683435 1.355196
F 3.350687 -3.718623 1.253580
F -0.258991 2.814528 -1.971229
F 1.808143 4.438178 -1.442052
F 3.575131 3.805926 0.509043
F 3.226862 1.531507 1.966826
F 1.139880 -0.059177 1.490830
Sn -0.017491 -1.019540 -2.141897
H 0.255536 -0.344986 -3.681582
H -0.964259 -2.432471 -2.227743

^{F}YSnPy
E = -2057.56659845
P -1.749631 0.164256 0.037169
C -0.138340 -0.316410 -0.032298
C 0.976969 0.616576 0.108521
C 1.890310 0.501029 1.164596
C 3.081701 1.207579 1.218253
C 3.363193 2.139859 0.228811
C 2.469138 2.314504 -0.821039
C 1.306209 1.560309 -0.873434
C -2.579140 -0.001683 1.664159
C -3.851087 0.528406 1.903484
H -4.381434 1.052024 1.107915
C -4.432046 0.399313 3.157936
H -5.421680 0.812522 3.343780
C -3.742829 -0.249807 4.180745
H -4.198358 -0.346283 5.164601
C -2.470774 -0.760516 3.950653
H -1.926077 -1.253313 4.753566
C -1.886233 -0.634609 2.693734
H -0.877862 -1.001294 2.503377
C -2.740870 -0.809241 -1.133617
C -3.926040 -1.464742 -0.799576
H -4.317418 -1.407851 0.213218
C -4.600263 -2.210526 -1.761552
H -5.518866 -2.729458 -1.495204
C -4.098708 -2.299225 -3.055025
H -4.628388 -2.885167 -3.803879
C -2.913161 -1.650514 -3.390754
H -2.512771 -1.729086 -4.399380
C -2.229607 -0.914898 -2.432479
H -1.283844 -0.426262 -2.667564
C -1.977641 1.927909 -0.330170
C -1.473359 2.846107 0.595718
H -1.031628 2.491958 1.526565
C -1.533345 4.207340 0.328136
H -1.132254 4.918323 1.047574
C -2.107571 4.658820 -0.856434
H -2.155939 5.725853 -1.064835
C -2.621489 3.747759 -1.772485
H -3.074293 4.099905 -2.697129
C -2.553247 2.383089 -1.515489
H -2.945961 1.673882 -2.240673
C 2.593748 -2.098157 -0.318790
C 4.808351 -1.962974 0.604144
C 4.219430 -0.979061 -1.489886
C 5.179185 -1.223675 -0.511455
F 0.526883 1.706441 -1.938965
F 2.751515 3.184337 -1.781324
F 4.486558 2.840857 0.271682
F 3.941704 1.009000 2.207249
F 1.657741 -0.391803 2.130739
N 2.961558 -1.395698 -1.397762
H 5.526997 -2.174451 1.394395
H 6.189652 -0.836687 -0.623876
H 4.477976 -0.405698 -2.382973
C 3.496963 -2.414176 0.698639
H 3.168016 -2.977878 1.570090
Sn 0.447308 -2.365438 -0.162965
H -0.210478 -3.153815 -1.530147

H 0.066469 -3.299066 1.232929

^{F}YSnPyr
E = -2073.62521702
P -1.737666 0.179459 0.040337
C -0.139720 -0.315869 -0.153498
C 0.979937 0.610478 0.029645
C 1.871357 0.469982 1.102347
C 3.064161 1.170972 1.194562
C 3.371663 2.118088 0.227527
C 2.498664 2.319237 -0.835094
C 1.331669 1.573266 -0.925827
C -2.426306 0.163255 1.740323
C -3.615112 0.826421 2.060981
H -4.152680 1.380996 1.291960
C -4.103310 0.788033 3.360569
H -5.027906 1.305638 3.609316
C -3.403609 0.095107 4.346497
H -3.784873 0.070452 5.365730
C -2.214462 -0.553163 4.033493
H -1.660601 -1.083157 4.805816
C -1.723444 -0.518413 2.731679
H -0.779668 -0.999055 2.478192
C -2.827510 -0.890818 -0.940187
C -4.007620 -1.445250 -0.441838
H -4.311642 -1.253057 0.584274
C -4.789270 -2.258413 -1.255311
H -5.703853 -2.696083 -0.860384
C -4.400490 -2.517232 -2.565150
H -5.014184 -3.155879 -3.197734
C -3.222587 -1.969505 -3.064952
H -2.911170 -2.179612 -4.086044
C -2.433551 -1.163099 -2.255186
H -1.495387 -0.746333 -2.620644
C -2.001962 1.905903 -0.461741
C -1.495289 2.908282 0.370489
H -1.054702 2.645420 1.331683
C -1.551257 4.237736 -0.027979
H -1.147601 5.013989 0.618966
C -2.124475 4.573921 -1.250422
H -2.169907 5.615830 -1.561354
C -2.641563 3.579539 -2.073804
H -3.094600 3.841823 -3.027752
C -2.575862 2.246473 -1.686009
H -2.969889 1.471299 -2.339581
C 2.594032 -2.095386 -0.368918
C 4.617495 -2.090292 0.667345
C 4.288662 -0.951453 -1.372327
C 5.146135 -1.280507 -0.329481
F 0.571860 1.752922 -1.997167
F 2.803818 3.207445 -1.771162
F 4.499047 2.809595 0.304435
F 3.907206 0.945076 2.192558
F 1.617926 -0.437917 2.044890
Sn 0.436784 -2.371006 -0.285170
H -0.145268 -3.126412 -1.706065
H -0.011165 -3.328355 1.064297
N 3.023195 -1.352433 -1.395865
N 3.352774 -2.498878 0.651529
H 5.230024 -2.414122 1.511671

H 6.173767 -0.926929 -0.297683
H 4.628344 -0.336715 -2.209229

^{F}YSnB2
E = -2216.58280477
P 2.075255 0.217445 0.020358
C 0.510148 -0.382255 -0.155044
C -0.658270 0.475778 -0.314007
C -1.519663 0.331522 -1.411963
C -2.712162 1.029882 -1.544140
C -3.080387 1.945171 -0.568779
C -2.261583 2.119617 0.540505
C -1.084929 1.394947 0.657774
C 3.153804 0.042791 -1.450116
C 4.427601 0.618813 -1.495188
H 4.807367 1.169160 -0.634248
C 5.201789 0.502998 -2.641698
H 6.191972 0.953448 -2.676415
C 4.705904 -0.180515 -3.750487
H 5.313277 -0.267546 -4.649518
C 3.432961 -0.737173 -3.715016
H 3.038820 -1.257113 -4.585900
C 2.653515 -0.622035 -2.567518
H 1.640870 -1.022947 -2.534280
C 2.949468 -0.610213 1.384117
C 4.191482 -1.232176 1.264824
H 4.722041 -1.222113 0.315923
C 4.745345 -1.890102 2.359500
H 5.709123 -2.385316 2.259466
C 4.066682 -1.923053 3.571663
H 4.502349 -2.440497 4.424356
C 2.823075 -1.307131 3.692635
H 2.284798 -1.342612 4.637614
C 2.259373 -0.662561 2.600874
H 1.271248 -0.207063 2.666784
C 2.111995 2.007253 0.318123
C 1.589199 2.830128 -0.684217
H 1.227887 2.389975 -1.612954
C 1.528981 4.203902 -0.493656
H 1.114399 4.840537 -1.272577
C 1.999692 4.763295 0.690914
H 1.952702 5.840351 0.839509
C 2.531870 3.947683 1.683006
H 2.903302 4.384475 2.607820
C 2.585434 2.569777 1.502254
H 2.991059 1.934068 2.286148
C -3.747331 -1.042866 1.093405
C -4.135181 -1.506297 -0.158705
C -4.531157 -0.179562 1.833424
C -5.325850 -1.122657 -0.743712
C -5.736116 0.215557 1.251669
H -4.203978 0.190579 2.801177
C -6.122564 -0.243337 -0.009131
H -5.603812 -1.468182 -1.735269
H -6.381074 0.909603 1.785762
H -7.062489 0.100853 -0.434580
B -2.104305 -2.229460 0.250389
O -3.130680 -2.276388 -0.673963
O -2.501413 -1.521897 1.372878
F -0.383366 1.545963 1.779066

F -1.214584 -0.555846 -2.359547
F -3.501498 0.821282 -2.587634
F -2.639391 2.945016 1.507213
F -4.216755 2.617006 -0.671992
Sn 0.096892 -2.495303 -0.132196
H 0.980655 -3.265092 1.117047
H 0.485945 -3.301384 -1.596548

^{F}YSnB1
E = -2485.77711052
P -2.120695 0.829630 0.285035
C -0.484484 0.438925 0.124533
C 0.131180 -0.602155 0.931570
C -0.370729 -1.900668 1.069379
C 0.301141 -2.901813 1.753551
C 1.534614 -2.640663 2.331955
C 2.068846 -1.361421 2.232241
C 1.363557 -0.372521 1.568603
C -3.389118 -0.024713 -0.721064
C -4.754034 0.082169 -0.436629
H -5.088284 0.666914 0.420146
C -5.684234 -0.566614 -1.238736
H -6.746095 -0.484885 -1.014342
C -5.255151 -1.327147 -2.324002
H -5.984253 -1.838300 -2.950080
C -3.897653 -1.444533 -2.600053
H -3.561332 -2.052910 -3.436834
C -2.964039 -0.796266 -1.798512
H -1.895929 -0.905390 -1.980376
C -2.328892 2.597051 -0.117687
C -3.402226 3.093376 -0.860512
H -4.157951 2.417243 -1.250892
C -3.499020 4.455435 -1.122012
H -4.334687 4.832347 -1.708325
C -2.529123 5.330997 -0.646294
H -2.607293 6.396205 -0.855839
C -1.452193 4.842369 0.086059
H -0.681968 5.520239 0.448078
C -1.347896 3.481654 0.345267
H -0.493300 3.083201 0.890582
C -2.687688 0.558010 1.997047
C -3.273414 -0.657752 2.359015
H -3.473565 -1.411140 1.601056
C -3.591801 -0.909065 3.689148
H -4.041790 -1.861012 3.963871
C -3.335633 0.049806 4.662212
H -3.588599 -0.147741 5.702164
C -2.753413 1.262577 4.305477
H -2.552284 2.016557 5.063842
C -2.424605 1.514616 2.979802
H -1.964879 2.462127 2.708231
N 3.733955 -0.099221 -0.781520
C 3.437025 -2.310008 -0.935106
C 4.282430 -1.346694 -0.509235
H 3.582132 -3.383792 -0.935454
H 5.268787 -1.453447 -0.073181
B 2.447755 -0.286920 -1.396097
N 2.310106 -1.719655 -1.496776
C 1.213970 -2.469256 -1.960578
C 0.828977 -3.647558 -1.315903

C 0.496352 -2.041002 -3.079083
C -0.282001 -4.354774 -1.752919
H 1.383189 -3.995019 -0.448076
C -0.603106 -2.765269 -3.522730
H 0.820362 -1.148990 -3.609563
C -1.007071 -3.917022 -2.856297
H -0.586167 -5.250013 -1.214690
H -1.144844 -2.421757 -4.402487
H -1.876595 -4.474588 -3.198025
C 4.354721 1.102512 -0.386220
C 4.367752 2.197830 -1.249289
C 4.955129 1.207740 0.869269
C 4.963398 3.389104 -0.854500
H 3.917627 2.101616 -2.234948
C 5.563703 2.395972 1.249956
H 4.901339 0.366558 1.555478
C 5.568600 3.492379 0.393089
H 4.964720 4.238442 -1.534930
H 6.023281 2.469869 2.233765
H 6.041454 4.423801 0.697532
Sn 0.763878 1.215815 -1.452898
H 1.268429 2.829008 -1.155485
H -0.154423 1.274373 -2.910357
F -1.495750 -2.240655 0.437954
F -0.182311 -4.139260 1.765114
F 2.206052 -3.604666 2.946888
F 3.249657 -1.098592 2.781146
F 1.905572 0.844104 1.509145

I
E = -1022.65604664
C 1.612922 2.479888 0.019864
C -0.513294 1.350470 -0.217694
C -1.102309 2.593521 -0.460990
C -0.358659 3.769372 -0.437093
C 1.003629 3.714337 -0.179930
H 2.686200 2.460459 0.208054
H -2.165693 2.636412 -0.681942
H -0.851138 4.721789 -0.622990
H 1.599880 4.624137 -0.143113
N -1.230564 0.150954 -0.232728
C -2.606068 0.038197 -0.049707
C -3.343300 0.884669 0.796203
C -3.290921 -1.017757 -0.671959
C -4.701407 0.679917 0.993091
H -2.833917 1.692274 1.315156
C -4.647157 -1.224165 -0.458615
H -2.741668 -1.674483 -1.347940
C -5.368103 -0.372098 0.370280
H -5.245444 1.348153 1.659188
H -5.145764 -2.053594 -0.957677
H -6.432692 -0.526007 0.532168
C 0.901592 1.270877 -0.014435
C 1.514250 -0.051361 0.107701
P 3.175116 -0.332440 0.169877
C 4.156400 0.304651 -1.232616
H 3.947887 1.372118 -1.357788
H 5.232028 0.156827 -1.082415
H 3.830737 -0.211958 -2.141596
C 4.056649 0.304527 1.637741

H 5.130521 0.088895 1.591023
H 3.907314 1.386370 1.712454
H 3.618892 -0.159974 2.527521
C 3.494800 -2.116233 0.191214
H 4.571588 -2.312316 0.227293
H 3.021665 -2.561716 1.073373
H 3.079949 -2.573356 -0.714458
Sn 0.026828 -1.502797 -0.096110
H -0.471153 -2.481406 1.218209
H 0.052466 -2.515702 -1.486430

II
E = -1571.87088588
C 0.831207 3.074972 0.005755
C 0.351966 1.785886 0.220873
C -0.860571 1.341337 -0.354413
C -1.538913 2.265842 -1.171416
C -1.062004 3.549485 -1.376028
C 0.130353 3.968044 -0.789728
H 1.759764 3.358150 0.497125
H -2.463994 1.948008 -1.647026
H -1.625394 4.229099 -2.013357
H 0.504220 4.977371 -0.945493
N -1.366375 0.057487 -0.181828
S 1.305239 0.723389 1.294463
O 2.561773 1.443389 1.586591
O 0.416969 0.328558 2.388691
C 1.655411 -0.651539 0.337851
C -2.749569 -0.147587 -0.110458
C -3.587006 0.737749 0.587488
C -3.327545 -1.289060 -0.680534
C -4.944222 0.483027 0.701465
H -3.149973 1.617866 1.053506
C -4.687258 -1.548355 -0.545224
H -2.705676 -1.973281 -1.259170
C -5.507230 -0.662386 0.140877
H -5.570822 1.181321 1.254004
H -5.106625 -2.446022 -0.996323
H -6.572049 -0.860130 0.241901
P 3.152755 -0.644054 -0.473996
C 3.472276 0.830435 -1.480400
H 4.454039 0.781211 -1.964645
H 2.681972 0.930253 -2.232048
H 3.431087 1.694380 -0.808268
C 3.227375 -2.059387 -1.596831
H 2.425590 -1.984955 -2.338950
H 4.195797 -2.081351 -2.107605
H 3.099214 -2.984929 -1.025699
C 4.571641 -0.776354 0.640032
H 4.476994 0.048646 1.354661
H 4.506047 -1.725335 1.181990
H 5.521216 -0.711688 0.096764
Sn -0.144221 -1.655639 0.011041
H -0.834839 -2.573116 1.253970
H -0.111442 -2.547620 -1.454173

III
E = -2333.95836046
C -0.401063 1.875369 -0.574745
C 0.400169 1.875392 0.574464

C 0.768416 3.094449 1.145666
C 0.382391 4.301208 0.578795
C -0.382665 4.301184 -0.579623
C -0.769100 3.094396 -1.146156
H 1.385453 3.068093 2.039898
H 0.684848 5.237575 1.043660
H -0.684890 5.237532 -1.044677
H -1.386306 3.068059 -2.040278
S -1.012629 0.423278 -1.509952
S 1.011633 0.423264 1.509685
O -2.160390 0.991252 -2.244321
O 0.155602 -0.053021 -2.277031
O -0.156698 -0.053539 2.276379
O 2.158957 0.991380 2.244693
C -1.532443 -0.781158 -0.462855
C 1.532312 -0.780664 0.462464
P 2.972537 -0.466304 -0.404227
P -2.971825 -0.467110 0.405492
C 3.088568 -1.595698 -1.804488
H 4.013175 -1.404898 -2.359371
H 3.078676 -2.633112 -1.455496
H 2.215342 -1.414338 -2.443223
C 3.074257 1.196814 -1.109713
H 3.043328 1.926256 -0.293398
H 4.003855 1.314696 -1.677613
H 2.202190 1.329403 -1.759955
C 4.463072 -0.654304 0.606857
H 4.534664 -1.693770 0.943584
H 5.371317 -0.367927 0.064473
H 4.315912 -0.012806 1.483241
C -3.085487 -1.595214 1.806962
H -4.009530 -1.404322 2.362758
H -3.075542 -2.632976 1.459019
H -2.211433 -1.412541 2.444257
C -3.074531 1.196663 1.109286
H -3.044975 1.925240 0.292128
H -4.003995 1.314021 1.677504
H -2.202336 1.331019 1.758913
C -4.463156 -0.656810 -0.604117
H -5.371292 -0.371808 -0.060835
H -4.317639 -0.014875 -1.480465
H -4.533652 -1.696215 -0.941268
Sn 0.000252 -2.163154 -0.000806
H -0.448364 -3.158540 1.311949
H 0.449618 -3.157867 -1.313761

IV
E = -1541.94749500
C 0.693377 3.264343 -0.016953
C 0.151402 2.008913 0.201106
C -1.041037 1.539706 -0.350667
C -1.723973 2.418850 -1.194225
C -1.217681 3.694017 -1.430267
C -0.022771 4.121190 -0.845631
H 1.627136 3.568170 0.452528
H -2.663001 2.110659 -1.652931
H -1.766065 4.377807 -2.076176
H 0.345695 5.127220 -1.035758
B -1.336691 0.043743 0.141393
S 0.904314 0.674628 1.088532

O 1.672586 1.097902 2.262459
O -0.460752 -0.046294 1.407757
C 1.698718 -0.322549 0.025674
C -2.815269 -0.462087 0.386319
C -3.795429 0.451571 0.798702
C -3.207331 -1.799025 0.250880
C -5.102259 0.052979 1.059776
H -3.526217 1.500669 0.920977
C -4.509556 -2.210350 0.512253
H -2.472511 -2.538488 -0.069402
C -5.463815 -1.282386 0.916483
H -5.841731 0.786739 1.377471
H -4.782416 -3.258275 0.396942
H -6.486055 -1.598945 1.117583
P 3.329769 -0.661737 0.347323
C 4.395062 0.803489 0.436054
H 5.436452 0.532792 0.643292
H 4.328993 1.347317 -0.511949
H 4.009892 1.436966 1.242756
C 3.960460 -1.702415 -0.985061
H 3.856059 -1.167204 -1.934512
H 5.011585 -1.956483 -0.811744
H 3.360216 -2.617714 -1.030839
C 3.633783 -1.548476 1.899807
H 3.217373 -0.931024 2.703562
H 3.094458 -2.501133 1.871561
H 4.701840 -1.724548 2.071632
Sn 0.125930 -1.246378 -1.247559
H 0.437709 -2.947379 -1.101775
H 0.585193 -0.894422 -2.875794

V
E = -992.715610931
C 1.638634 2.492562 -0.114545
C -0.513855 1.333555 -0.147442
C -1.120471 2.601662 -0.316076
C -0.402131 3.775375 -0.372856
C 0.994659 3.700994 -0.256229
H 2.725417 2.499256 -0.046156
H -2.203495 2.638305 -0.423210
H -0.897345 4.733703 -0.510673
H 1.588154 4.614670 -0.290728
B -1.362600 0.073511 -0.088049
C -2.907137 -0.035748 0.014988
C -3.660271 0.834756 0.823385
C -3.612011 -1.035514 -0.677279
C -5.041467 0.718816 0.925987
H -3.146470 1.603401 1.398716
C -4.994965 -1.135388 -0.606683
H -3.056961 -1.746527 -1.289754
C -5.714885 -0.259026 0.200943
H -5.596674 1.396964 1.572114
H -5.514399 -1.909229 -1.169504
H -6.797766 -0.345664 0.272977
C 0.936243 1.253209 -0.071237
C 1.593581 -0.012397 0.033425
P 3.272443 -0.222496 0.130290
C 4.266249 0.367326 -1.286678
H 4.090937 1.436269 -1.443136
H 5.337093 0.193099 -1.129468

H 3.931054 -0.166618 -2.182210
C 4.096938 0.515473 1.583584
H 5.179182 0.342319 1.564213
H 3.901343 1.591962 1.612813
H 3.664274 0.066636 2.483861
C 3.660515 -1.986716 0.238686
H 4.742797 -2.138854 0.308708
H 3.170548 -2.409450 1.122329
H 3.275827 -2.494953 -0.652398
Sn 0.145175 -1.608366 -0.049263
H 0.300889 -2.649845 1.316071
H 0.455426 -2.640880 -1.397343

Pathway B

$^{Tos}Y_2Sn$
E = -3797.74178854
C -4.273828 -2.539955 -0.752835
H -4.020625 -3.195291 0.077017
C -3.368765 -1.557571 -1.125262
C 3.775573 2.081371 -0.970527
O -0.781524 -1.925683 -1.205658
C 3.850656 2.235617 -2.352247
H 2.992234 1.988523 -2.976086
C -2.640069 2.167601 3.385136
H -3.673850 1.831674 3.430280
C -5.484397 -2.641185 -1.429487
H -6.203013 -3.403543 -1.129493
C 1.889222 -4.920172 -1.554647
H 1.042039 -5.435427 -2.001859
C 1.683959 -3.694446 -0.934140
H 0.683246 -3.263230 -0.890646
C -2.177007 2.816015 -1.602596
H -1.483968 2.025394 -1.889757
C -2.845815 2.713257 -0.385562
C -5.319807 0.846208 0.396466
H -5.304655 1.585395 -0.401366
C -6.494934 0.160881 0.684208
H -7.397468 0.376905 0.115323
C 3.161371 -5.478719 -1.610953
H 3.313965 -6.438187 -2.102041
C 2.152502 -0.217574 -0.784750
Sn 0.266957 -0.408238 -2.398050
O 2.440176 1.520485 1.217308
S 2.264956 1.477513 -0.244642
P 2.463040 -1.447665 0.470416
C 5.023321 2.716473 -2.911507
H 5.093225 2.831627 -3.992270
C 4.039218 -1.118293 2.772617
H 3.154660 -1.405137 3.336448
C 0.543286 -0.637911 2.305838
H 0.721169 0.365717 1.946143
C -5.352423 -1.092978 2.394825
H -5.358630 -1.860459 3.166294
C -4.173786 -0.418207 2.106684
H -3.253555 -0.676108 2.628666
C 4.047068 -3.593806 -0.400789
H 4.891357 -3.096948 0.068499
C -1.910557 2.047243 2.202191
C -3.657324 -0.687758 -2.171846

H -2.946105 0.095659 -2.433801
C -3.706469 3.732604 0.030403
H -4.208084 3.660067 0.995691
C -4.155493 0.570223 1.115241
C 0.106958 -3.220737 3.251526
H -0.074956 -4.231768 3.609117
C 1.184380 -1.721123 1.703102
C 0.964314 -3.018299 2.179556
H 1.458079 -3.869296 1.717958
C 2.771022 -3.025641 -0.358870
C 4.239070 -4.816498 -1.033136
H 5.234400 -5.254838 -1.064493
C -7.132873 -1.856040 -3.165755
H -7.856424 -1.170971 -2.701063
H -7.556521 -2.864609 -3.106802
H -7.056985 -1.576883 -4.222763
C -0.516089 -2.137571 3.861976
H -1.181705 -2.301799 4.707617
C 6.114614 3.072900 -2.108260
C -6.514898 -0.802320 1.686952
H -7.435843 -1.338743 1.908383
C 6.297816 -0.355577 1.327705
H 7.174023 -0.049600 0.759516
C -1.447589 0.203358 -0.031658
P -2.531347 1.265977 0.671179
O -1.963537 -2.286877 0.934898
S -1.833899 -1.357370 -0.203317
O 1.192293 2.262585 -0.852607
C -5.803209 -1.775850 -2.477009
C -4.865486 -0.804633 -2.842907
H -5.092119 -0.121068 -3.660609
C -2.385015 3.929562 -2.411315
H -1.855569 4.012952 -3.358536
C -3.258688 4.932734 -2.008257
H -3.421686 5.801172 -2.644343
C -3.919700 4.835467 -0.785007
H -4.596520 5.625743 -0.465387
C 5.116809 -0.655828 0.661521
H 5.076797 -0.575324 -0.424266
C -0.295939 -0.848982 3.391907
H -0.773669 0.006548 3.865617
C 3.984563 -1.044317 1.381434
C 6.350207 -0.430513 2.716041
H 7.273193 -0.188488 3.239464
C 5.995658 2.934456 -0.724847
H 6.830433 3.219121 -0.085758
C 5.221364 -0.808711 3.435165
H 5.257799 -0.859871 4.521355
C -0.710704 3.123790 4.476217
H -0.239061 3.528627 5.370016
C -2.036580 2.705225 4.519612
H -2.605461 2.789784 5.443965
C 4.830586 2.440479 -0.147223
H 4.727979 2.321428 0.928774
C 7.369029 3.608060 -2.729830
H 7.185706 4.578182 -3.209833
H 7.746911 2.932514 -3.507076
H 8.159098 3.747065 -1.985147
C 0.009657 3.030841 3.287298
H 1.047972 3.352399 3.237182

C -0.592355 2.506027 2.152522
H -0.034053 2.424511 1.222202
H 2.918148 -0.380230 -1.554728
H 1.234136 -1.827384 -2.982524

TosYSnCl
E = -2361.79594561
C 2.472728 -1.488662 -0.331437
C 2.839834 -1.790694 0.975634
H 2.126600 -2.257000 1.653953
C -2.307179 2.687894 3.124977
H -3.162414 3.348549 3.247761
C -1.962417 2.246922 1.854353
H -2.568553 2.529775 0.998912
C -1.586253 2.264200 4.235620
H -1.867468 2.605619 5.229816
C -0.266609 -0.962694 0.203381
Sn -2.375923 -2.390625 0.131921
O 0.671113 -1.188374 -2.207419
S 0.803650 -1.801220 -0.879757
P -0.373389 0.788145 0.061096
C 4.127998 -1.498158 1.400109
H 4.420048 -1.726843 2.424070
C 1.525124 1.805366 -1.698126
H 0.810025 1.533024 -2.470111
C -2.063190 0.476749 -2.131077
H -1.886395 -0.590947 -2.037424
C -0.168103 0.942031 2.811155
H 0.654718 0.236504 2.701174
C -2.438645 3.226363 -2.431058
H -2.579882 4.298248 -2.552022
C -1.466139 1.368083 -1.243631
C -1.634607 2.746954 -1.406637
H -1.130830 3.445010 -0.738908
C -0.875947 1.387743 1.690547
C -0.521294 1.384065 4.079489
H 0.030470 1.029295 4.947202
C -3.053104 2.333893 -3.305490
H -3.681454 2.710447 -4.110343
C 5.062860 -0.926276 0.531044
C 3.384093 2.447859 0.271963
H 4.107501 2.697587 1.045460
O 0.547398 -3.227184 -0.678493
C 2.160168 1.897872 0.630540
H 1.938561 1.736649 1.681364
C -2.855929 0.965579 -3.161854
H -3.322036 0.267800 -3.853552
C 1.219692 1.579254 -0.352378
C 3.678147 2.686424 -1.066617
H 4.635028 3.124401 -1.344357
C 4.674679 -0.670122 -0.785574
H 5.391977 -0.235868 -1.480817
C 2.748924 2.362921 -2.048405
H 2.975644 2.541150 -3.097558
C 3.385928 -0.944696 -1.222484
H 3.067834 -0.728144 -2.239173
C 6.439966 -0.572169 1.004377
H 7.195770 -0.798130 0.243771
H 6.702879 -1.110737 1.920645
H 6.511651 0.502932 1.221283

H -0.076438 -1.253298 1.238842
H -1.919976 -2.769865 1.817715
Cl -3.629830 -0.295490 0.790158

[Tos]YSnHMDS
E = -2775.57104813
C -2.800649 -2.024351 0.150200
C -3.093006 -2.588512 -1.089492
H -2.285192 -2.852012 -1.771065
C 0.060405 4.336001 -2.410034
H 0.637373 5.255894 -2.346081
C -0.074203 3.534790 -1.282157
H 0.416232 3.826131 -0.357935
C -0.520468 3.956909 -3.613423
H -0.406362 4.583465 -4.495621
C -0.624495 -0.361894 -0.505968
Sn 1.617392 -1.001374 -1.751353
O -1.083051 -1.151993 1.936823
S -1.102670 -1.665394 0.558963
P -0.965745 1.253583 0.084387
C -4.415981 -2.819879 -1.428430
H -4.651683 -3.258144 -2.397450
C -2.872746 1.366802 2.118684
H -2.037154 1.176579 2.787232
C 1.090616 0.910700 1.921488
H 1.271920 -0.052570 1.456059
C -1.372757 1.961663 -2.571113
H -1.924345 1.027134 -2.655786
C 0.689293 3.426015 3.077322
H 0.533055 4.403944 3.527543
C 0.115729 1.769692 1.420685
C -0.095477 3.023984 2.007376
H -0.885394 3.677634 1.638771
C -0.794802 2.342251 -1.354540
C -1.238385 2.767797 -3.692244
H -1.686878 2.459371 -4.634085
C 1.660528 2.564489 3.584173
H 2.267350 2.872206 4.433561
C -5.455567 -2.512313 -0.540606
C -5.024094 1.769279 0.396039
H -5.862083 1.926290 -0.279779
O -0.332052 -2.855573 0.209437
C -3.734448 1.694021 -0.114318
H -3.577694 1.803399 -1.184529
C 1.851829 1.312991 3.013881
H 2.604414 0.639115 3.413710
C -2.650549 1.492678 0.744116
C -5.240759 1.651083 1.765195
H -6.251420 1.715771 2.163859
C -5.127395 -1.978973 0.705404
H -5.920648 -1.746497 1.414614
C -4.164644 1.453297 2.623475
H -4.329231 1.357184 3.694797
C -3.804551 -1.736800 1.060397
H -3.541787 -1.328007 2.031936
C -6.881326 -2.768696 -0.925356
H -7.054524 -3.836926 -1.108342
H -7.144280 -2.239064 -1.849779
H -7.573049 -2.445593 -0.140974
H -1.088204 -0.508532 -1.485136

H 1.702593 0.742938 -2.173211
Si 4.100735 0.596864 -0.169880
Si 3.414816 -2.293724 0.713415
C 2.850341 -3.807729 -0.249973
H 1.762963 -3.840503 -0.377483
H 3.322044 -3.862822 -1.240018
H 3.149009 -4.702467 0.314444
C 5.262607 -2.556522 1.017709
H 5.634037 -2.018013 1.896304
H 5.440782 -3.626835 1.188389
H 5.867179 -2.254036 0.152144
C 2.558192 -2.345393 2.389467
H 2.780782 -3.290069 2.903805
H 2.899943 -1.529759 3.040224
H 1.468307 -2.273803 2.284643
C 3.197944 2.259075 -0.172649
H 3.822557 2.991937 -0.702189
H 2.243360 2.192860 -0.702635
H 3.019465 2.640609 0.839487
C 5.146950 0.554105 -1.739449
H 4.501371 0.602536 -2.627932
H 5.850307 1.396111 -1.792987
H 5.724709 -0.378051 -1.794874
N 3.060308 -0.807056 -0.144098
C 5.279298 0.682693 1.306926
H 6.187829 0.090349 1.154436
H 5.582420 1.727524 1.457790
H 4.805606 0.336368 2.235001

^{Tos}*YSnB1*
E = -2577.44789013
C 3.372817 -1.376674 -1.231276
C 2.975199 -2.360951 -2.130810
H 2.107413 -2.195225 -2.764684
C -2.887230 0.149802 2.719509
H -3.915396 0.428112 2.502985
C -1.869712 0.690232 1.946242
H -2.118926 1.367057 1.132490
C -2.594040 -0.749568 3.737575
H -3.399246 -1.191904 4.320733
C 1.084038 -0.248020 -0.063469
Sn -0.662854 -1.667723 -1.128153
O 3.304719 1.140372 -0.474909
S 2.452096 0.138759 -1.138549
P 0.795689 0.983580 1.181280
C 3.704383 -3.538733 -2.192738
H 3.397851 -4.317977 -2.889010
C 2.507361 2.402951 2.909131
H 1.881295 3.270908 2.718257
C 0.876739 3.162635 -0.568680
H 1.440128 2.547154 -1.261000
C -0.244658 -0.520160 3.265797
H 0.787498 -0.772405 3.497254
C -0.602829 4.788191 1.153178
H -1.185932 5.416410 1.823295
C 0.353162 2.634601 0.613606
C -0.389633 3.454257 1.473781
H -0.793953 3.053750 2.400654
C -0.540044 0.363631 2.226309
C -1.272614 -1.085268 4.009281

H -1.037745 -1.786452 4.806964
C -0.061402 5.316171 -0.013620
H -0.219221 6.364197 -0.261021
C 4.828853 -3.739891 -1.384953
C 4.154672 0.208207 3.374847
H 4.800097 -0.650826 3.546996
O 1.958025 0.442667 -2.481957
C 3.070966 0.092219 2.514913
H 2.889381 -0.855892 2.011789
C 0.673917 4.503915 -0.867495
H 1.086674 4.906659 -1.788166
C 2.234904 1.187061 2.283724
C 4.419311 1.421405 4.002405
H 5.273193 1.514718 4.670436
C 5.217033 -2.719096 -0.513825
H 6.104905 -2.851922 0.102691
C 3.596738 2.516296 3.765591
H 3.806335 3.470804 4.243930
C 4.497237 -1.533315 -0.431270
H 4.806126 -0.720846 0.223093
C 5.598304 -5.023581 -1.464186
H 5.914902 -5.232047 -2.493568
H 4.980304 -5.871335 -1.141160
H 6.491679 -4.997710 -0.832222
H 1.367779 -1.136883 0.515549
H -0.891930 -2.273198 0.536114
N -3.801874 -0.746464 -0.381326
C -4.143663 1.379653 -0.978238
C -4.705345 0.308705 -0.383945
H -4.620498 2.322545 -1.208967
H -5.724315 0.200664 -0.030627
C -2.079002 1.970608 -2.143899
C -0.977995 1.522837 -2.879057
C -2.424403 3.325769 -2.229867
C -0.270064 2.389612 -3.699540
H -0.669678 0.483518 -2.828966
C -1.730819 4.180531 -3.077232
H -3.241454 3.724512 -1.636003
C -0.654485 3.719896 -3.827014
H 0.592772 2.000737 -4.235591
H -2.033493 5.224840 -3.138105
H -0.113524 4.392399 -4.489954
C -4.125951 -1.995276 0.169499
C -3.609834 -3.168585 -0.386804
C -4.988382 -2.101937 1.265884
C -3.907524 -4.405926 0.165960
H -2.996785 -3.111002 -1.285101
C -5.296702 -3.344190 1.805783
H -5.411759 -1.203531 1.707834
C -4.750588 -4.503811 1.267707
H -3.486286 -5.303416 -0.282823
H -5.967527 -3.402329 2.661357
H -4.988620 -5.475359 1.695464
N -2.845672 1.069739 -1.381115
B -2.547924 -0.299777 -0.974095

$^{Ph}Y_2Sn$
E = -2620.58994123
P 2.839727 -0.477517 0.160229
P -2.806351 -0.289613 0.057323

C 1.466714 0.557745 -0.156544
C 1.716936 2.021163 -0.168164
C 2.386851 2.643085 -1.229868
H 2.709976 2.045145 -2.079588
C 2.616920 4.013924 -1.223936
H 3.138501 4.474048 -2.061723
C 2.162415 4.799813 -0.169926
H 2.326020 5.875672 -0.175999
C 1.474248 4.199773 0.879889
H 1.086485 4.804429 1.697228
C 1.265954 2.827856 0.884825
H 0.726046 2.367805 1.710868
C 4.040053 -0.581156 -1.188570
C 5.389844 -0.818646 -0.911938
H 5.733240 -0.879275 0.118642
C 6.295620 -0.969193 -1.954675
H 7.346032 -1.148727 -1.734998
C 5.861232 -0.885234 -3.273941
H 6.573867 -0.999547 -4.088653
C 4.518525 -0.651508 -3.551851
H 4.173954 -0.581769 -4.581472
C 3.606230 -0.500966 -2.514375
H 2.555455 -0.310990 -2.737783
C 2.191409 -2.134281 0.469171
C 2.481138 -3.204859 -0.373863
H 3.175384 -3.077810 -1.201587
C 1.857132 -4.430921 -0.170080
H 2.074456 -5.262438 -0.837093
C 0.953551 -4.591387 0.873815
H 0.464993 -5.552111 1.026434
C 0.673886 -3.526364 1.726815
H -0.031844 -3.642897 2.546580
C 1.287115 -2.298897 1.522744
H 1.056180 -1.467911 2.185480
C 3.819937 0.003061 1.617087
C 4.339320 1.304779 1.652803
H 4.192921 1.978256 0.810647
C 5.045673 1.739993 2.766004
H 5.440770 2.753486 2.787012
C 5.244441 0.887364 3.848113
H 5.797517 1.232953 4.719385
C 4.740024 -0.407684 3.812838
H 4.901846 -1.080824 4.652484
C 4.029730 -0.851816 2.702372
H 3.645233 -1.868758 2.679886
C -1.745264 0.742890 -0.751246
C -1.792233 2.184668 -0.517811
C -1.262132 3.087324 -1.470584
H -0.822478 2.684851 -2.382734
C -1.272642 4.457994 -1.279866
H -0.840455 5.102007 -2.045058
C -1.823472 5.017931 -0.127260
H -1.829477 6.095724 0.022879
C -2.358653 4.161106 0.826691
H -2.795810 4.565034 1.739659
C -2.340065 2.783923 0.640375
H -2.761129 2.153950 1.423168
C -4.481330 0.438036 0.251667
C -4.902609 1.324566 -0.744773
H -4.218711 1.568727 -1.555997

C -6.165756 1.898642 -0.683295
H -6.480393 2.592831 -1.460100
C -7.018326 1.599624 0.374896
H -8.006514 2.053405 0.425124
C -6.600989 0.727659 1.374046
H -7.260622 0.496938 2.208632
C -5.336261 0.150649 1.315841
H -5.016949 -0.515955 2.112788
C -3.103308 -1.858878 -0.828430
C -2.371506 -3.012476 -0.542434
H -1.619615 -2.994273 0.240504
C -2.578465 -4.174779 -1.274624
H -1.993448 -5.064256 -1.047149
C -3.513529 -4.194376 -2.303661
H -3.673816 -5.104591 -2.878881
C -4.240662 -3.045853 -2.600337
H -4.971425 -3.053671 -3.406658
C -4.036639 -1.883090 -1.866529
H -4.607965 -0.987178 -2.101122
C -2.369743 -0.850159 1.766507
C -2.931044 -1.965090 2.397006
H -3.668690 -2.570821 1.872276
C -2.545178 -2.312267 3.687233
H -2.984771 -3.184426 4.168445
C -1.597602 -1.546663 4.362668
H -1.297907 -1.818337 5.373341
C -1.037963 -0.432877 3.744451
H -0.299394 0.170992 4.269282
C -1.419225 -0.091958 2.450035
H -0.982393 0.769798 1.946055
Sn -0.114928 -0.075649 -2.026703
H -0.164250 -1.713877 -1.310512
H 0.733107 0.270660 0.606322

^{Ph}YSnCl
E = -1773.21499324
P 0.946247 0.016148 -0.026111
C -0.575174 0.203818 -0.900116
C -1.342515 1.459000 -0.707966
C -2.143756 1.930070 -1.757772
H -2.174005 1.371769 -2.693376
C -2.909373 3.078904 -1.617348
H -3.526486 3.416168 -2.448387
C -2.893741 3.794370 -0.424243
H -3.495988 4.693523 -0.312517
C -2.103204 3.341222 0.624653
H -2.080692 3.885485 1.566953
C -1.339321 2.189930 0.485507
H -0.729413 1.862471 1.326016
C 1.985047 -1.117060 -0.981026
C 3.059095 -0.658225 -1.745060
H 3.320822 0.397620 -1.745601
C 3.800463 -1.558391 -2.502589
H 4.639282 -1.200155 -3.095720
C 3.472720 -2.909789 -2.499637
H 4.055384 -3.611006 -3.093937
C 2.404448 -3.367693 -1.733925
H 2.149097 -4.425264 -1.726994
C 1.660380 -2.477351 -0.970613
H 0.825426 -2.830451 -0.364629

C 0.848698 -0.674744 1.635169
C 1.997531 -1.231870 2.208645
H 2.927626 -1.275346 1.642886
C 1.944657 -1.745129 3.497718
H 2.836596 -2.180211 3.943835
C 0.747773 -1.713647 4.209414
H 0.706835 -2.123625 5.216884
C -0.396457 -1.178757 3.629594
H -1.342920 -1.178144 4.165546
C -0.353717 -0.659644 2.340672
H -1.273468 -0.291925 1.885202
C 1.847926 1.592001 0.097315
C 1.739085 2.486029 -0.973201
H 1.108995 2.238668 -1.825976
C 2.409637 3.700918 -0.935630
H 2.316728 4.394191 -1.768811
C 3.180497 4.037015 0.173328
H 3.698082 4.993749 0.205517
C 3.277400 3.158057 1.246164
H 3.868788 3.424724 2.119621
C 2.612100 1.937476 1.212496
H 2.678865 1.262647 2.062242
Cl -3.517195 -0.552168 1.004431
Sn -2.220024 -1.657146 -0.835472
H -1.191454 -2.583218 0.314323
H -0.299481 0.061193 -1.952450

^{Ph}YSnHMDS
E = -2186.98598799
P -2.002670 0.145822 -0.056653
C -0.268898 -0.015823 -0.315757
C 0.189820 -1.434412 -0.308791
C 0.392531 -2.137394 -1.503723
H 0.267933 -1.620230 -2.454598
C 0.712930 -3.491095 -1.494977
H 0.859303 -4.015695 -2.437627
C 0.840036 -4.173587 -0.289199
H 1.088525 -5.232935 -0.281504
C 0.677113 -3.479619 0.906013
H 0.798718 -3.994947 1.857138
C 0.366502 -2.126195 0.895466
H 0.240054 -1.583450 1.830611
C -2.963504 0.018392 -1.599951
C -4.315333 -0.339572 -1.557183
H -4.787500 -0.567417 -0.603009
C -5.054477 -0.413362 -2.730530
H -6.104419 -0.696163 -2.690615
C -4.451842 -0.131442 -3.953244
H -5.031007 -0.194218 -4.872393
C -3.109329 0.226034 -4.000622
H -2.634025 0.445198 -4.954328
C -2.364197 0.300807 -2.828742
H -1.313631 0.579360 -2.882532
C -2.447000 1.737184 0.677993
C -3.346868 2.614147 0.072028
H -3.812592 2.353403 -0.875568
C -3.642508 3.829832 0.679722
H -4.339571 4.515058 0.202059
C -3.047996 4.168882 1.889796
H -3.281227 5.121647 2.361079

C -2.148626 3.296750 2.495812
H -1.672902 3.564364 3.436652
C -1.839603 2.086536 1.889916
H -1.128715 1.405040 2.360770
C -2.684335 -1.160302 0.997245
C -2.702819 -2.462558 0.482413
H -2.304666 -2.664134 -0.511158
C -3.208712 -3.502795 1.246950
H -3.210130 -4.513342 0.844644
C -3.699566 -3.254433 2.526127
H -4.092016 -4.073190 3.126136
C -3.691040 -1.962338 3.036270
H -4.079366 -1.764030 4.032931
C -3.187619 -0.912731 2.273804
H -3.196653 0.096308 2.676799
N 2.939123 0.398251 -0.020870
Si 3.974790 -0.674389 0.879686
Si 3.421728 0.930163 -1.603265
C 3.454950 -0.895001 2.674497
H 3.408947 0.056021 3.218758
H 2.479363 -1.382462 2.775453
H 4.206786 -1.530295 3.163739
C 4.075862 -2.398925 0.114955
H 4.890217 -2.477687 -0.615007
H 4.245809 -3.160571 0.887684
H 3.138712 -2.641119 -0.397966
C 5.738354 0.009511 0.953714
H 6.390561 -0.648255 1.544474
H 6.185553 0.105419 -0.044294
H 5.750728 1.005006 1.417577
C 4.373095 -0.385396 -2.560051
H 4.577391 -0.024651 -3.577319
H 5.334199 -0.642986 -2.099533
H 3.779291 -1.305786 -2.634755
C 4.461703 2.500407 -1.521872
H 4.766637 2.850403 -2.517343
H 3.886948 3.303409 -1.040470
H 5.366985 2.339361 -0.923272
C 1.936912 1.341187 -2.708532
H 1.257388 2.074433 -2.253454
H 2.300113 1.770868 -3.651973
H 1.369331 0.434510 -2.956926
H -0.096028 0.445184 -1.293130
Sn 1.301062 1.567236 0.819594
H 1.083610 0.634721 2.341266

^{Ph}*YSnB1*
E = -1988.87846260
P -1.948555 -0.314138 0.027752
C -0.729272 0.830173 -0.563191
C -0.307892 1.769743 0.515617
C 0.478158 1.361943 1.603023
H 0.900021 0.360996 1.611629
C 0.736801 2.222612 2.661864
H 1.344631 1.874363 3.495194
C 0.236034 3.522143 2.654568
H 0.439340 4.195143 3.485491
C -0.504419 3.957375 1.561243
H -0.881985 4.977752 1.526524
C -0.770770 3.092439 0.507432

H -1.368816 3.438012 -0.335012
C -1.451755 -1.415596 1.374951
C -2.415172 -1.802698 2.314344
H -3.417040 -1.381210 2.278195
C -2.096137 -2.729792 3.299246
H -2.850991 -3.023921 4.025596
C -0.818882 -3.276562 3.352003
H -0.570789 -4.005895 4.121024
C 0.141850 -2.893065 2.422015
H 1.141930 -3.317710 2.449725
C -0.168743 -1.967378 1.434641
H 0.579131 -1.686024 0.696790
C -2.565005 -1.323989 -1.336537
C -3.031418 -2.617976 -1.096819
H -2.985720 -3.036918 -0.093926
C -3.535004 -3.378813 -2.143676
H -3.889681 -4.389528 -1.953299
C -3.574090 -2.853879 -3.431776
H -3.962951 -3.453980 -4.252138
C -3.108765 -1.566553 -3.673750
H -3.125734 -1.157604 -4.681507
C -2.604307 -0.797621 -2.631268
H -2.228305 0.201317 -2.838030
C -3.379057 0.603387 0.676830
C -3.218857 1.343748 1.856052
H -2.285759 1.289289 2.415745
C -4.253258 2.147775 2.314820
H -4.120607 2.722100 3.229270
C -5.449266 2.222453 1.606485
H -6.257779 2.854290 1.969215
C -5.611878 1.488967 0.436636
H -6.547445 1.541581 -0.116547
C -4.580082 0.681670 -0.030762
H -4.711760 0.108268 -0.946150
N 3.157805 1.432347 -0.225756
C 4.352248 0.008752 1.019327
C 4.168946 1.308297 0.719157
H 5.112686 -0.430584 1.653440
H 4.740559 2.161036 1.065467
B 2.632725 0.109121 -0.541737
N 3.459910 -0.766600 0.281764
C 3.396175 -2.157234 0.446546
C 3.719952 -2.749296 1.673758
C 3.013154 -2.984474 -0.613960
C 3.660179 -4.128931 1.832153
H 3.998630 -2.120645 2.515925
C 2.928202 -4.358819 -0.439708
H 2.802939 -2.543079 -1.583421
C 3.252602 -4.943042 0.780966
H 3.920864 -4.567179 2.794245
H 2.619655 -4.980831 -1.277744
H 3.197056 -6.022114 0.907856
C 2.694779 2.698647 -0.626094
C 2.193265 2.905103 -1.914253
C 2.729404 3.779239 0.259759
C 1.701968 4.148699 -2.287492
H 2.201514 2.088210 -2.630412
C 2.260804 5.027116 -0.129331
H 3.088502 3.630124 1.274566
C 1.731376 5.219620 -1.399781

H 1.310410 4.282700 -3.294506
H 2.288403 5.850729 0.582116
H 1.353625 6.195357 -1.698589
H -1.290875 1.399261 -1.316772
Sn 0.870283 -0.320511 -2.037222
H 0.351707 -1.886637 -1.348771

I
E = -1022.69769888
C 2.192492 2.260159 -0.829242
C -0.057383 1.345891 -0.635071
C -0.550619 2.650525 -0.824859
C 0.307214 3.723751 -1.013289
C 1.687363 3.541617 -1.021911
H 3.271003 2.096455 -0.836602
H -1.626412 2.804130 -0.856682
H -0.111625 4.715798 -1.176157
H 2.359363 4.381344 -1.184235
N -0.853718 0.226843 -0.519542
C -2.105994 0.245078 0.058113
C -2.485248 1.166448 1.057941
C -3.050280 -0.742932 -0.283538
C -3.730064 1.095590 1.667818
H -1.787176 1.945110 1.358822
C -4.288097 -0.811321 0.337225
H -2.795309 -1.465039 -1.058626
C -4.646538 0.108635 1.318734
H -3.983503 1.824765 2.436669
H -4.987312 -1.591683 0.040040
H -5.621068 0.058792 1.799229
C 1.351221 1.164536 -0.637161
P 2.119466 -0.517086 1.317457
C 3.266586 0.643900 2.106908
H 2.903519 1.661446 1.922646
H 3.333495 0.465416 3.185999
H 4.259519 0.539641 1.655819
C 0.575211 -0.382312 2.234493
H 0.763168 -0.541094 3.301846
H 0.151238 0.614955 2.074480
H -0.143890 -1.119884 1.861007
C 2.778825 -2.176160 1.582101
H 2.935276 -2.367969 2.648786
H 2.061628 -2.897981 1.174350
H 3.730428 -2.280045 1.049359
Sn 0.148404 -1.658605 -1.201566
H 0.466564 -0.986559 -2.815686
C 1.881150 -0.222687 -0.403280
H 2.829897 -0.423258 -0.917698

II
E = -1571.89629800
C -0.959135 3.458242 0.328653
C -0.623708 2.114008 0.206539
C -1.549107 1.131010 -0.186269
C -2.873286 1.554554 -0.376955
C -3.220678 2.889546 -0.239960
C -2.266694 3.853188 0.089362
H -0.190391 4.162998 0.636645
H -3.615466 0.812294 -0.663768
H -4.252818 3.190526 -0.412651

H -2.546281 4.900449 0.176264
N -1.133173 -0.165729 -0.449019
S 0.924216 1.610069 0.890858
O 1.717417 2.808365 1.166825
O 0.600940 0.711289 2.020808
C -1.783466 -1.231056 0.152014
C -2.318550 -1.109465 1.449682
C -1.867831 -2.488611 -0.470733
C -2.914139 -2.192644 2.078523
H -2.222220 -0.156314 1.965567
C -2.452246 -3.570467 0.175529
H -1.477095 -2.608142 -1.480344
C -2.986434 -3.435082 1.452630
H -3.314720 -2.066622 3.083596
H -2.503583 -4.529935 -0.337503
H -3.450976 -4.282071 1.952672
C 1.825026 0.588347 -0.253358
H 2.581619 1.213628 -0.741383
P 2.593129 -0.722698 0.660794
C 3.509152 -0.177864 2.119588
H 4.205427 0.613957 1.823109
H 2.788033 0.231230 2.832845
H 4.060701 -1.015151 2.561775
C 3.797986 -1.471133 -0.458229
H 4.288292 -2.318046 0.033872
H 3.274185 -1.819955 -1.355624
H 4.552363 -0.730214 -0.743488
C 1.427520 -2.007083 1.132231
H 1.954695 -2.794015 1.682748
H 0.646498 -1.554219 1.750005
H 0.969218 -2.429298 0.230344
H 0.129272 1.267743 -2.545121
Sn 0.427155 -0.388791 -2.003372

III
E = -2333.95557594
C -0.452990 1.950305 -0.467809
C 0.346646 1.883994 0.675361
C 0.693785 3.068931 1.323702
C 0.297736 4.303378 0.828574
C -0.468833 4.367089 -0.327942
C -0.846045 3.194780 -0.966950
H 1.303092 2.983976 2.220164
H 0.589970 5.214011 1.348100
H -0.781554 5.326265 -0.735186
H -1.465591 3.221656 -1.859368
S -0.982854 0.611285 -1.574879
S 0.935150 0.356691 1.490660
O -2.188390 1.162609 -2.229220
O 0.146476 0.263990 -2.434068
O -0.306373 -0.167530 2.115219
O 1.982931 0.878777 2.390770
C 1.482879 -0.698919 0.339690
P 2.929735 -0.281194 -0.455098
P -2.923396 -0.504639 0.369235
C 3.155846 -1.311507 -1.914786
H 4.079775 -1.027067 -2.430194
H 3.197089 -2.366570 -1.628280
H 2.281686 -1.157664 -2.556547
C 3.015435 1.425916 -1.071583

H 2.961537 2.116493 -0.223478
H 3.954316 1.586744 -1.613527
H 2.159309 1.586938 -1.736322
C 4.407853 -0.460289 0.584329
H 4.516110 -1.514026 0.862422
H 5.317352 -0.107914 0.084055
H 4.215141 0.119182 1.494497
C -3.006713 -1.727595 1.683531
H -3.910397 -1.559957 2.280415
H -3.015533 -2.735591 1.257631
H -2.104492 -1.604269 2.293434
C -2.983547 1.116736 1.146160
H -3.014846 1.888418 0.371453
H -3.896856 1.156286 1.750472
H -2.101141 1.230949 1.782471
C -4.433021 -0.632251 -0.618761
H -5.323350 -0.414172 -0.018706
H -4.334044 0.087214 -1.439751
H -4.512421 -1.642415 -1.035357
C -1.522807 -0.815252 -0.680384
H -1.906173 -1.407572 -1.526254
Sn 0.133881 -2.462441 0.068343
H 0.570649 -2.653665 -1.664276

IV
E = -1541.93264299
C 0.455093 3.593377 -0.564115
C 0.422971 2.214766 -0.409742
C 1.412770 1.473472 0.255827
C 2.492691 2.217876 0.759198
C 2.543488 3.599140 0.621927
C 1.528926 4.295607 -0.033069
H -0.349891 4.084715 -1.105785
H 3.298724 1.702846 1.279944
H 3.394737 4.144211 1.026855
H 1.582415 5.376617 -0.140404
B 1.243056 -0.040525 0.557524
S -0.877041 1.308388 -1.174567
O -1.765497 2.239602 -1.876076
O -0.245521 0.220376 -1.958670
C 2.126942 -1.136238 -0.076828
C 2.874044 -0.873791 -1.239864
C 2.170602 -2.439745 0.447634
C 3.627008 -1.866790 -1.850732
H 2.836898 0.124705 -1.672926
C 2.938813 -3.432883 -0.145666
H 1.583894 -2.665139 1.338733
C 3.664746 -3.145607 -1.298924
H 4.189657 -1.648687 -2.756802
H 2.968471 -4.433601 0.281920
H 4.260631 -3.924060 -1.773311
C -1.780228 0.445412 0.085350
H -2.545609 1.118674 0.486800
P -2.542178 -0.961819 -0.680332
C -3.263673 -0.595695 -2.297597
H -3.914433 0.279955 -2.204972
H -2.447297 -0.346425 -2.982341
H -3.829764 -1.456627 -2.670343
C -3.891035 -1.479128 0.399868
H -4.377804 -2.370048 -0.011423

H -3.470042 -1.700248 1.387723
H -4.623247 -0.669564 0.488158
C -1.421734 -2.356410 -0.870869
H -1.954017 -3.187429 -1.346675
H -0.574967 -2.032158 -1.483056
H -1.063133 -2.656795 0.120430
H -0.515474 1.278896 2.635845
Sn -0.556402 -0.401683 2.065913

V
E = -992.724708637
C 1.616594 2.565400 -0.130360
C -0.501635 1.375827 -0.190252
C -1.146494 2.614613 -0.036442
C -0.440410 3.798902 0.116842
C 0.950470 3.764485 0.092413
H 2.702281 2.584256 -0.204249
H -2.235004 2.633781 -0.010548
H -0.964413 4.741861 0.261371
H 1.523554 4.681898 0.218976
B -1.275758 0.023223 -0.119579
C -2.832524 -0.059361 -0.143440
C -3.641771 0.569749 0.818307
C -3.485646 -0.812505 -1.133631
C -5.027549 0.454020 0.791947
H -3.175364 1.147840 1.616011
C -4.871096 -0.902519 -1.184127
H -2.887816 -1.339262 -1.877310
C -5.649799 -0.273441 -0.216962
H -5.625864 0.941169 1.560769
H -5.347419 -1.481464 -1.974149
H -6.734907 -0.356707 -0.245119
C 0.917269 1.363929 -0.301464
C 1.579632 0.073104 -0.620631
P 3.243294 -0.225489 -0.202267
C 4.554384 0.704009 -1.070007
H 4.504365 1.771370 -0.837554
H 5.541952 0.322870 -0.785100
H 4.417740 0.576347 -2.149681
C 3.500989 0.053393 1.560642
H 4.535703 -0.163054 1.844832
H 3.258789 1.094818 1.798465
H 2.808742 -0.603993 2.100499
C 3.640114 -1.947323 -0.570498
H 4.700421 -2.143017 -0.378190
H 3.010974 -2.592931 0.052786
H 3.414056 -2.149301 -1.623439
Sn 0.156441 -1.671956 0.406859
H 1.382708 -0.287367 -1.637703
H 0.101684 -2.568770 -1.135706

Pathway C

IV
E = -1541.95645177
C 0.268081 3.226067 0.822221
C -0.153189 1.912333 0.705100
C -1.348336 1.498416 0.129171
C -2.192653 2.499576 -0.346163
C -1.815227 3.835160 -0.236587

C -0.596680 4.201272 0.339533
H 1.222063 3.479494 1.279606
H -3.149632 2.237405 -0.795361
H -2.482745 4.613468 -0.602872
H -0.326977 5.252473 0.417541
B -1.439569 -0.108536 0.155172
S 0.775817 0.494012 1.196667
O 1.375001 0.575365 2.535170
O -0.477785 -0.489232 1.234746
C 1.791835 0.006907 -0.014424
C -2.832953 -0.844378 0.275456
C -3.776472 -0.780729 -0.757179
C -3.180397 -1.552208 1.430690
C -5.021085 -1.388892 -0.640509
H -3.527751 -0.252350 -1.678956
C -4.422568 -2.167125 1.554305
H -2.459044 -1.616299 2.244261
C -5.348131 -2.085698 0.519396
H -5.737784 -1.325317 -1.458111
H -4.670784 -2.712681 2.463800
H -6.320746 -2.565950 0.613837
P 3.102465 -0.929048 0.580672
C 4.311296 0.042001 1.514876
H 5.132285 -0.588523 1.874448
H 4.700299 0.832790 0.865287
H 3.782818 0.496740 2.358453
C 3.997891 -1.646387 -0.813348
H 4.344632 -0.844059 -1.473074
H 4.857402 -2.217315 -0.445863
H 3.330667 -2.303948 -1.380630
C 2.641717 -2.312081 1.660296
H 2.099826 -1.893609 2.515577
H 1.968739 -2.977479 1.109106
H 3.519825 -2.867502 2.008683
Sn 0.828267 -0.327071 -2.047661
H 0.348497 1.368007 -2.193011
H -0.913663 -0.542064 -0.988618

V
E = -992.730037309
C 1.641184 2.468184 0.179202
C -0.491482 1.317282 -0.146902
C -1.112391 2.584548 -0.066046
C -0.396804 3.753049 0.092026
C 0.997961 3.685872 0.208170
H 2.720961 2.454109 0.318898
H -2.193248 2.638271 -0.184940
H -0.905023 4.714977 0.114513
H 1.578885 4.597987 0.339522
B -1.353185 0.096675 -0.534182
C -2.899807 -0.032690 -0.351380
C -3.576266 0.543491 0.736652
C -3.673321 -0.746160 -1.279925
C -4.951797 0.422034 0.885865
H -3.002528 1.085724 1.487905
C -5.051389 -0.865439 -1.144553
H -3.177112 -1.214517 -2.129954
C -5.695540 -0.280173 -0.058839
H -5.448571 0.872923 1.743662
H -5.626396 -1.419330 -1.885135

H -6.774290 -0.375572 0.054433
C 0.937667 1.244313 0.019821
C 1.568679 -0.049241 0.046389
P 3.246387 -0.237143 -0.245195
C 3.828122 0.618062 -1.742072
H 3.553778 1.676023 -1.687769
H 4.913034 0.521699 -1.861014
H 3.318127 0.175821 -2.604216
C 4.385000 0.295148 1.081821
H 5.428310 0.099312 0.807584
H 4.258579 1.361073 1.292016
H 4.131832 -0.264050 1.989027
C 3.700272 -1.969142 -0.498974
H 4.778715 -2.039817 -0.678060
H 3.439515 -2.557562 0.386591
H 3.146721 -2.370421 -1.352454
Sn 0.172262 -1.624176 0.485059
H -0.853505 -0.663026 -1.375029
H 0.908686 -2.945895 -0.447218

6.2.5 Phenol coordinated species

Pathway A

I
Not observed.

II
Not observed.

III
E = -2640.77340287
C -1.535466 1.710576 0.919515
C -2.566426 1.252471 0.088708
C -3.782016 1.934145 0.068107
C -3.988892 3.052863 0.864830
C -2.974665 3.495825 1.702682
C -1.759282 2.823817 1.728555
H -4.566957 1.552652 -0.579504
H -4.944892 3.571708 0.830220
H -3.124476 4.364410 2.341191
H -0.947391 3.153617 2.371963
S 0.126819 0.977730 1.105694
S -2.502242 -0.197453 -1.020387
O 0.900879 2.064840 1.757524
O -0.075061 -0.231834 1.928539
O -1.728358 0.268505 -2.225946
O -3.926764 -0.516875 -1.211850
C 0.772594 0.674794 -0.412249
C -1.574457 -1.413309 -0.398154
P -2.170264 -2.360632 0.877552
P 1.644411 2.000163 -1.047675
C -0.812278 -3.292266 1.604981
H -1.170404 -3.928302 2.421211
H -0.348909 -3.911726 0.829952
H -0.082026 -2.561322 1.966021
C -2.972208 -1.418094 2.199806
H -3.807660 -0.861123 1.759141
H -3.349604 -2.086366 2.981953
H -2.232333 -0.720116 2.603910
C -3.434969 -3.541949 0.330573

H -2.984984 -4.240361 -0.382745
H -3.886476 -4.092187 1.164272
H -4.193821 -2.952015 -0.197186
C 2.010247 1.657649 -2.787681
H 2.575407 2.489350 -3.221920
H 2.602055 0.738819 -2.863807
H 1.069924 1.524562 -3.334761
C 0.732285 3.568839 -1.056606
H 0.531881 3.847798 -0.017399
H 1.303837 4.360417 -1.554290
H -0.219587 3.402638 -1.573696
C 3.243508 2.343102 -0.274768
H 3.739134 3.206197 -0.734374
H 3.050677 2.522786 0.787159
H 3.868283 1.448020 -0.370731
O 2.233722 -2.314749 0.174954
C 3.284525 -1.561744 0.608394
C 4.505073 -1.564696 -0.062744
C 3.111151 -0.781122 1.749685
C 5.567030 -0.809245 0.428577
H 4.628317 -2.169652 -0.961800
C 4.176672 -0.030931 2.225554
H 2.138586 -0.756421 2.232021
C 5.410648 -0.043682 1.577735
H 6.521461 -0.827112 -0.094536
H 4.031603 0.580995 3.113748
H 6.240835 0.544740 1.962099
Sn 0.264091 -1.097989 -1.595331
H 2.533733 -2.881296 -0.543407

IV
E = -1848.75866193
C 0.596326 1.973537 2.385250
C -0.190429 1.075509 1.684944
C -1.203544 1.410293 0.776470
C -1.385808 2.780404 0.546573
C -0.612529 3.714819 1.228999
C 0.366848 3.325688 2.150805
H 1.346906 1.630463 3.095165
H -2.160859 3.118829 -0.139464
H -0.785635 4.776390 1.058776
H 0.938410 4.079061 2.689235
B -1.948435 0.098788 0.284225
S -0.069094 -0.679555 1.709601
O 0.426737 -1.205354 2.986741
O -1.587888 -0.919473 1.410343
C 0.708889 -1.256853 0.338373
C -3.483542 0.081704 -0.051658
C -4.379802 -0.734521 0.651547
C -4.017215 0.893278 -1.064761
C -5.740910 -0.735931 0.363480
H -3.994951 -1.376035 1.443198
C -5.376294 0.903574 -1.352451
H -3.347149 1.521755 -1.654288
C -6.247945 0.085649 -0.637846
H -6.412754 -1.381376 0.928331
H -5.759144 1.547127 -2.143711
H -7.312929 0.086290 -0.864082
P 2.182603 -2.072820 0.605480
C 3.439615 -1.069798 1.437327

H 4.373599 -1.624645 1.580676
H 3.620223 -0.173589 0.833816
H 3.033114 -0.776085 2.411938
C 2.871908 -2.562775 -0.988264
H 3.095202 -1.661458 -1.566705
H 3.787130 -3.146934 -0.842205
H 2.125659 -3.155542 -1.527725
C 2.045671 -3.593462 1.584678
H 1.591808 -3.320307 2.542936
H 1.377266 -4.285719 1.061795
H 3.023246 -4.063240 1.742739
C 4.698930 1.371306 -1.518298
C 4.553520 2.184312 -0.400137
C 3.274136 2.534021 0.028636
C 2.149410 2.080520 -0.645828
C 2.306139 1.269989 -1.765189
C 3.578104 0.909130 -2.203313
H 5.690839 1.094253 -1.870160
H 5.430240 2.547540 0.131130
H 3.139419 3.170696 0.900686
H 1.146999 2.355050 -0.331435
H 3.692425 0.283268 -3.088973
O 1.177399 0.851265 -2.403032
H 1.403197 0.381735 -3.213431
Sn -0.575449 -0.996203 -1.445672

V
E = -1299.51972656
C 1.158617 2.554371 1.470633
C -0.943454 1.574799 0.739562
C -1.581565 2.288266 1.769815
C -0.889755 3.114083 2.641870
C 0.491992 3.231567 2.478778
H 2.231762 2.712740 1.373822
H -2.663339 2.196992 1.866702
H -1.406238 3.666518 3.423752
H 1.061529 3.879191 3.145595
B -1.698958 0.675242 -0.246186
C -3.133546 0.123920 -0.050287
C -3.617821 -0.261721 1.214578
C -3.993523 -0.064173 -1.147706
C -4.889528 -0.798141 1.374206
H -2.971651 -0.151865 2.085095
C -5.280317 -0.560844 -0.989810
H -3.635826 0.190596 -2.145402
C -5.730269 -0.936390 0.273495
H -5.231780 -1.102827 2.362080
H -5.930629 -0.675808 -1.855594
H -6.731399 -1.346030 0.397993
C 0.481804 1.702787 0.563913
C 1.127586 0.970257 -0.504375
P 2.819124 0.842878 -0.623475
C 3.768688 2.348601 -1.060361
H 3.595943 3.127489 -0.310827
H 4.844067 2.146380 -1.131356
H 3.396955 2.715686 -2.023169
C 3.655221 0.200017 0.863174
H 4.745485 0.252025 0.763196
H 3.338104 0.767970 1.742987
H 3.343514 -0.840551 0.999892

C 3.285930 -0.334763 -1.918174
H 4.377005 -0.411816 -1.982500
H 2.859400 -1.311954 -1.667624
H 2.874029 -0.011067 -2.879556
Sn -0.212429 0.094213 -1.982291
H 0.368746 -2.896924 -1.423875
O 0.117697 -2.247622 -0.759189
C 0.831834 -2.432978 0.388819
C 0.489170 -1.664400 1.497214
C 1.876935 -3.349157 0.458579
C 1.187875 -1.834249 2.683543
H -0.315161 -0.937883 1.413743
C 2.567247 -3.514434 1.657126
H 2.141387 -3.942628 -0.417151
C 2.226240 -2.760006 2.773345
H 0.916967 -1.229022 3.546289
H 3.375867 -4.240756 1.711331
H 2.766876 -2.889951 3.708068

Pathway B

I
E = -1329.50756795
C 1.621339 1.587521 1.981836
C -0.208632 0.087189 1.425706
C -1.001874 0.769155 2.364017
C -0.504009 1.850137 3.071177
C 0.812144 2.271360 2.875450
H 2.654754 1.912796 1.865383
H -2.023758 0.440023 2.530350
H -1.142791 2.363167 3.787676
H 1.209677 3.118765 3.430416
N -0.644661 -0.985371 0.681506
C -1.998720 -1.361236 0.628130
C -3.009179 -0.439399 0.318372
C -2.352691 -2.702432 0.810587
C -4.326910 -0.855243 0.197697
H -2.747801 0.602998 0.153767
C -3.673656 -3.114907 0.675283
H -1.573470 -3.418947 1.066541
C -4.669555 -2.194060 0.372508
H -5.092026 -0.121947 -0.052240
H -3.924583 -4.164641 0.818371
H -5.704208 -2.515573 0.271503
C 1.155335 0.485004 1.238234
P 3.497118 0.149188 -0.215187
C 3.626525 1.815395 -0.941217
H 3.334698 2.564465 -0.198224
H 4.644312 2.024690 -1.289456
H 2.921805 1.873994 -1.778652
C 4.798234 0.080641 1.058882
H 5.771116 0.380827 0.652918
H 4.532778 0.736804 1.893498
H 4.853498 -0.945861 1.436441
C 4.080509 -0.964608 -1.517949
H 5.102352 -0.700538 -1.809888
H 4.063425 -1.996844 -1.151913
H 3.421434 -0.883716 -2.388804
C 1.923235 -0.288142 0.278158
H 0.743254 1.060896 -1.010070

Sn 0.801908 -1.894849 -0.600721
O 0.245222 1.215149 -1.829371
C -0.901906 1.882204 -1.566170
C -1.963741 1.736583 -2.459404
C -1.048065 2.699414 -0.443954
C -3.166696 2.386958 -2.218349
H -1.830618 1.091144 -3.324371
C -2.259054 3.339486 -0.209189
H -0.223474 2.806784 0.259389
C -3.326028 3.187210 -1.089864
H -3.992927 2.256949 -2.915242
H -2.365597 3.957498 0.680502
H -4.272795 3.687904 -0.899248

//
E = -1878.70646852
C 1.147856 2.992847 -1.560892
C 1.093490 1.665268 -1.145411
C 2.229530 0.971440 -0.646402
C 3.412750 1.742229 -0.564099
C 3.458516 3.064859 -0.967487
C 2.331869 3.708199 -1.481224
H 0.234185 3.442569 -1.943729
H 4.306357 1.276279 -0.157541
H 4.397496 3.608582 -0.874425
H 2.378556 4.742079 -1.813610
N 2.169176 -0.325044 -0.208988
S -0.502894 0.915602 -1.073705
O -1.472261 1.841257 -1.654487
O -0.367339 -0.440888 -1.761459
C -0.763805 0.414548 0.518034
C 3.348084 -1.065938 -0.030103
C 4.300025 -1.205488 -1.052251
C 3.556173 -1.764461 1.165433
C 5.413065 -2.013023 -0.875753
H 4.143237 -0.677523 -1.991015
C 4.664796 -2.588594 1.331418
H 2.840145 -1.643013 1.978295
C 5.601900 -2.713899 0.314460
H 6.136185 -2.110284 -1.683835
H 4.799658 -3.124758 2.269107
H 6.473108 -3.352343 0.443965
P -0.740143 1.576044 1.778323
C -1.236133 0.741240 3.300245
H -1.279840 1.453379 4.131008
H -2.220195 0.290198 3.132312
H -0.510244 -0.044933 3.534819
C -1.931862 2.896209 1.466241
H -2.921135 2.428707 1.407835
H -1.907591 3.657884 2.252890
H -1.704653 3.341905 0.491509
C 0.857153 2.366274 2.135091
H 1.615232 1.580614 2.233276
H 1.151154 3.017071 1.303791
H 0.801592 2.952101 3.059864
Sn 0.336727 -1.526289 0.150010
O -3.544753 -0.081472 1.167470
C -4.414905 -0.584643 0.268269
C -4.062195 -0.782060 -1.069803
C -5.701981 -0.911875 0.698480

C -4.993185 -1.306771 -1.957862
H -3.066204 -0.509138 -1.412405
C -6.622966 -1.431856 -0.201158
H -5.958628 -0.751537 1.743416
C -6.276543 -1.635907 -1.534005
H -4.705906 -1.453685 -2.997577
H -7.624111 -1.683261 0.145714
H -7.000872 -2.044738 -2.235068
H -2.644636 0.000557 0.768721

III
E = -2640.78975236
C -2.618815 0.940828 0.063458
C -1.756252 1.450897 1.040498
C -2.241134 2.370753 1.969764
C -3.559897 2.803386 1.923367
C -4.408273 2.321066 0.935335
C -3.937235 1.392436 0.016639
H -1.550106 2.753568 2.716138
H -3.918523 3.521043 2.658656
H -5.440688 2.660638 0.879097
H -4.588291 0.976426 -0.747642
S -2.214049 -0.347538 -1.166114
S 0.018320 1.075014 1.223530
O -3.549660 -0.847608 -1.540673
O -1.412676 0.328356 -2.234525
O 0.062074 -0.209487 1.994622
O 0.515028 2.255763 1.968388
C -1.210922 -1.486514 -0.495984
P 1.147401 2.424456 -0.982385
P -1.899245 -2.614614 0.570278
C 1.606160 2.122632 -2.703706
H 1.869500 3.067126 -3.191724
H 2.460803 1.438819 -2.746957
H 0.747724 1.668101 -3.212984
C -0.246675 3.580280 -1.062224
H -0.538112 3.844196 -0.039843
H 0.013800 4.486386 -1.620584
H -1.073579 3.049872 -1.550023
C 2.520906 3.327718 -0.222926
H 3.412143 2.691337 -0.254610
H 2.713293 4.279201 -0.731915
H 2.247089 3.495045 0.823780
C -0.614239 -3.549672 1.423199
H -1.058752 -4.414717 1.926910
H 0.142611 -3.881130 0.705761
H -0.134280 -2.897142 2.158044
C -2.915758 -1.863963 1.869889
H -3.755057 -1.341521 1.396942
H -3.297760 -2.624494 2.560201
H -2.286971 -1.139612 2.400236
C -2.983526 -3.816999 -0.248242
H -3.496469 -4.469822 0.467484
H -3.706547 -3.233136 -0.829201
H -2.384182 -4.416189 -0.941995
C 0.769915 0.928505 -0.249117
H 1.180854 -1.168124 1.351834
O 1.805112 -1.704555 0.781559
C 3.076418 -1.231456 0.888846
C 3.387661 -0.141966 1.703448

C 4.070220 -1.846435 0.130373
C 4.699266 0.313954 1.759047
H 2.601854 0.349396 2.272300
C 5.376369 -1.375792 0.192179
H 3.802159 -2.689440 -0.503009
C 5.699414 -0.294779 1.006280
H 4.937038 1.162408 2.398572
H 6.148116 -1.861874 -0.402101
H 6.723251 0.069774 1.054839
Sn 0.739955 -0.968249 -1.422619

IV
E = -1848.75893407
C 0.311456 1.730087 2.672434
C 0.112612 0.623454 1.861083
C -1.085414 0.279523 1.224751
C -2.156055 1.161226 1.429393
C -2.001564 2.272852 2.247268
C -0.781690 2.564263 2.869755
H 1.270471 1.912871 3.153840
H -3.123203 0.955705 0.974004
H -2.851311 2.932643 2.414158
H -0.692951 3.436150 3.514483
B -0.930014 -1.107273 0.472431
S 1.305211 -0.599086 1.438535
O 2.310234 -0.864470 2.475725
O 0.268454 -1.757473 1.239672
C 1.917570 -0.265916 -0.095773
C -2.133761 -2.103225 0.307052
C -3.293039 -1.719696 -0.385381
C -2.106223 -3.398914 0.839022
C -4.376512 -2.577370 -0.527427
H -3.339584 -0.726344 -0.837452
C -3.183807 -4.267179 0.692764
H -1.219949 -3.724417 1.382361
C -4.326442 -3.860531 0.011951
H -5.263163 -2.248363 -1.068158
H -3.133043 -5.269097 1.117752
H -5.170576 -4.538481 -0.102566
P 3.614045 -0.419377 -0.244359
C 4.558172 0.705864 0.819271
H 5.636938 0.572190 0.681103
H 4.278294 1.737344 0.581093
H 4.283159 0.486295 1.856099
C 4.068924 -0.027212 -1.946262
H 3.741971 0.992228 -2.176405
H 5.152280 -0.113486 -2.083540
H 3.546065 -0.715133 -2.619577
C 4.278483 -2.069434 0.110205
H 3.989524 -2.315316 1.138141
H 3.813406 -2.789430 -0.571618
H 5.368758 -2.105343 0.005431
C -1.809733 2.400110 -1.746240
C -0.553170 2.587748 -1.177294
C -0.293881 3.697522 -0.381866
C -1.309238 4.615225 -0.140786
C -2.572231 4.433116 -0.692618
C -2.815394 3.324104 -1.498541
H -1.989000 1.520239 -2.361731
H 0.692905 3.827699 0.058787

H -1.108865 5.477559 0.492259
H -3.364086 5.152964 -0.498211
H -3.800510 3.171825 -1.935236
O 0.414583 1.663898 -1.440344
H 1.064862 1.535367 -0.714414
Sn 0.302662 -0.814452 -1.625419

V
E = -1299.52451581
C 2.217657 2.549516 -0.174142
C 0.104534 1.579677 -0.877011
C -0.364682 2.882419 -1.113591
C 0.421513 4.004708 -0.897859
C 1.718177 3.820308 -0.420778
H 3.245592 2.466166 0.175775
H -1.373154 3.005547 -1.507409
H 0.043378 5.003250 -1.105972
H 2.360157 4.683936 -0.248136
B -0.727802 0.323600 -1.178754
C -2.272607 0.242657 -1.242304
C -3.089092 1.111301 -0.493241
C -2.919189 -0.770826 -1.974310
C -4.470652 0.972924 -0.473860
H -2.622398 1.883175 0.115304
C -4.301561 -0.893653 -1.987798
H -2.313317 -1.477722 -2.541538
C -5.081206 -0.024010 -1.229058
H -5.075390 1.642874 0.135220
H -4.774965 -1.679724 -2.573815
H -6.164889 -0.130701 -1.219003
C 1.441824 1.388908 -0.387211
C 1.904194 0.027207 -0.136978
P 3.459486 -0.313212 0.477347
C 4.912877 0.141768 -0.536164
H 4.901616 1.217922 -0.736948
H 5.854609 -0.127047 -0.043519
H 4.835344 -0.384183 -1.493957
C 3.814862 0.411382 2.116112
H 4.827527 0.158709 2.450727
H 3.703932 1.499150 2.082859
H 3.084260 0.014124 2.829278
C 3.645674 -2.093722 0.744780
H 4.612326 -2.306968 1.214268
H 2.831687 -2.443057 1.390103
H 3.571666 -2.618837 -0.213159
Sn 0.697581 -1.543568 -1.193668
C -2.235147 -1.580495 1.992122
C -3.363755 -1.004985 2.559536
C -3.327606 0.299134 3.046024
C -2.148557 1.029874 2.950002
C -1.013301 0.468941 2.376827
C -1.056729 -0.842859 1.904940
H -2.250924 -2.595249 1.601725
H -4.284819 -1.582015 2.616611
H -4.215197 0.743887 3.490502
H -2.108197 2.054261 3.316153
H -0.093730 1.044600 2.278641
O 0.036280 -1.423028 1.344040
H 0.758966 -0.750342 1.174692

Pathway C

IV
E = -1848.72208617
C -1.425631 3.330705 0.085216
C -0.947065 2.036611 -0.078026
C 0.303765 1.607405 0.411106
C 1.019853 2.573088 1.137915
C 0.554012 3.871120 1.317731
C -0.665919 4.262760 0.780883
H -2.397578 3.581760 -0.332322
H 1.972715 2.303312 1.587461
H 1.155032 4.581945 1.882890
H -1.030671 5.279657 0.911104
B 0.806848 0.122938 0.194605
S -2.022269 0.911486 -0.961411
O -3.303680 1.631908 -1.187761
O -1.305805 0.464344 -2.157398
C -2.340669 -0.408935 0.111559
C 1.526288 -0.334518 -1.133969
C 1.627654 0.506065 -2.251000
C 2.142866 -1.592305 -1.222055
C 2.318024 0.115548 -3.390864
H 1.137294 1.477336 -2.225212
C 2.846879 -1.987963 -2.353009
H 2.084126 -2.278386 -0.373283
C 2.936918 -1.130483 -3.444605
H 2.375912 0.788151 -4.245269
H 3.321397 -2.967523 -2.385370
H 3.482326 -1.434472 -4.336586
P -4.018501 -0.787097 0.190776
C -5.044349 0.452628 1.026077
H -6.099503 0.156534 1.028273
H -4.684972 0.566997 2.054088
H -4.909074 1.393760 0.485767
C -4.248608 -2.314150 1.132683
H -3.835005 -2.193495 2.139213
H -5.317556 -2.545508 1.197522
H -3.720009 -3.135522 0.638633
C -4.797286 -1.083242 -1.418924
H -4.676658 -0.169285 -2.008427
H -4.265400 -1.901536 -1.915309
H -5.858939 -1.332463 -1.309716
C 3.481938 -0.098718 1.197773
C 4.266626 -1.060013 1.815789
C 5.635322 -1.074130 1.569424
C 6.199864 -0.143470 0.705589
C 5.390444 0.806808 0.089842
C 4.023664 0.840129 0.331853
H 3.813059 -1.795176 2.480285
H 6.257588 -1.824212 2.052824
H 7.268993 -0.161569 0.507377
H 5.823689 1.531254 -0.596227
H 3.376087 1.559991 -0.160191
O 2.115529 -0.067418 1.435593
H 1.853117 -0.765311 2.048186
Sn -0.748046 -1.507771 0.983456

V
Not determined.

6.2.6 Phenol activation transition states

Pathway A

I
Not observed.

II
Not observed.

III
E = -2640.71103440
C -1.958027 1.664402 0.730130
C -2.855623 0.938404 -0.067861
C -4.182143 1.353168 -0.160509
C -4.633401 2.470385 0.530769
C -3.749585 3.186909 1.323497
C -2.424737 2.779787 1.423059
H -4.853873 0.771057 -0.785872
H -5.674186 2.775559 0.444075
H -4.085581 4.062894 1.874788
H -1.719931 3.320752 2.048780
S -0.221738 1.253157 1.120990
S -2.453688 -0.488711 -1.121954
O 0.297365 2.487483 1.755938
O -0.321941 0.064773 1.983640
O -1.701008 0.114541 -2.275225
O -3.759250 -1.111594 -1.378890
C 0.673608 1.005343 -0.290615
C -1.326154 -1.490637 -0.424366
P -1.755937 -2.576878 0.819357
P 1.769684 2.264231 -0.690714
C -0.287433 -3.084486 1.721228
H -0.500355 -3.964543 2.336790
H 0.518817 -3.294382 1.007431
H 0.017693 -2.237644 2.343220
C -2.935062 -1.839125 1.972831
H -3.821287 -1.535835 1.402773
H -3.222353 -2.553239 2.752236
H -2.455206 -0.956890 2.408954
C -2.576799 -4.055300 0.169490
H -1.872678 -4.600432 -0.467926
H -2.945426 -4.710277 0.967260
H -3.408472 -3.701613 -0.451202
C 2.579726 1.812230 -2.243794
H 3.256412 2.616314 -2.552200
H 3.159778 0.895190 -2.086456
H 1.831770 1.642113 -3.025872
C 0.979735 3.869049 -0.976561
H 0.487064 4.161817 -0.044550
H 1.717149 4.626155 -1.265628
H 0.227492 3.753619 -1.763988
C 3.119392 2.544678 0.473544
H 3.731302 3.397797 0.158536
H 2.676264 2.729634 1.456270
H 3.730006 1.635860 0.516523
O 2.160992 -2.169720 -0.394406
C 3.166171 -1.554616 0.219192

C 4.421507 -1.411807 -0.397633
C 2.999727 -1.018225 1.510676
C 5.479204 -0.805714 0.273620
H 4.555752 -1.818879 -1.398983
C 4.068235 -0.440514 2.179521
H 2.011026 -1.046727 1.961954
C 5.318852 -0.329818 1.571472
H 6.445665 -0.719479 -0.221774
H 3.915682 -0.049596 3.184695
H 6.152573 0.128463 2.099182
Sn 0.443743 -0.795317 -1.355001
H 1.865950 -1.751826 -1.817800

IV
E = -1848.71184647
C -0.523769 -1.768509 2.534748
C 0.266327 -0.916404 1.784609
C 1.283101 -1.314057 0.913392
C 1.485595 -2.690043 0.780918
C 0.705637 -3.581271 1.513830
C -0.285030 -3.132374 2.391050
H -1.286572 -1.387580 3.210784
H 2.269557 -3.067528 0.126755
H 0.880115 -4.651056 1.414242
H -0.867290 -3.849533 2.965896
B 2.025102 -0.030771 0.333742
S 0.104045 0.842343 1.673402
O -0.315313 1.451019 2.935370
O 1.622475 1.073111 1.278159
C -0.727842 1.290965 0.307732
C 3.534064 0.002661 -0.083388
C 4.420637 0.902423 0.520996
C 4.056151 -0.878409 -1.040964
C 5.771338 0.919697 0.188237
H 4.038422 1.597054 1.267633
C 5.404261 -0.869442 -1.374877
H 3.388339 -1.573457 -1.552104
C 6.269152 0.032174 -0.759281
H 6.440195 1.629381 0.673113
H 5.783427 -1.563533 -2.123430
H 7.325624 0.044129 -1.021778
P -2.258011 2.028109 0.510330
C -3.481436 0.993683 1.345308
H -4.437866 1.517192 1.454421
H -3.617972 0.075290 0.762729
H -3.089056 0.741478 2.337360
C -2.931675 2.443310 -1.107348
H -3.094308 1.519362 -1.670451
H -3.881910 2.974337 -0.983911
H -2.215731 3.073232 -1.644980
C -2.188133 3.576707 1.448494
H -1.739616 3.357682 2.422666
H -1.541990 4.280544 0.913491
H -3.187658 4.007918 1.575016
C -4.642369 -1.431264 -1.494573
C -4.497020 -2.202717 -0.344037
C -3.212034 -2.493465 0.114066
C -2.095944 -2.014203 -0.555174
C -2.230245 -1.250055 -1.727453
C -3.529006 -0.958956 -2.181351

H -5.638213 -1.201624 -1.871602
H -5.371387 -2.577104 0.183829
H -3.070174 -3.097423 1.009693
H -1.092104 -2.252002 -0.213552
H -3.641015 -0.385532 -3.100503
O -1.153088 -0.838401 -2.385212
H -0.909199 0.526271 -2.706246
Sn 0.445864 0.701374 -1.464066

V
E = -1299.46790223
C 0.806257 3.007540 0.851586
C -1.183016 1.697609 0.352659
C -1.928215 2.645319 1.085609
C -1.345150 3.737586 1.699295
C 0.039507 3.894767 1.583900
H 1.875794 3.196572 0.783506
H -3.006581 2.509143 1.155133
H -1.942245 4.454767 2.257500
H 0.529069 4.739567 2.068086
B -1.862927 0.499706 -0.294197
C -3.262070 -0.094693 -0.037974
C -3.864948 -0.041674 1.233445
C -3.974036 -0.744701 -1.062145
C -5.118271 -0.593507 1.462750
H -3.325678 0.419958 2.058732
C -5.241558 -1.267299 -0.847575
H -3.515449 -0.837066 -2.046540
C -5.815223 -1.195661 0.419034
H -5.556106 -0.554329 2.458639
H -5.777486 -1.750609 -1.662430
H -6.800818 -1.622932 0.596057
C 0.242047 1.890106 0.187131
C 1.006006 0.971775 -0.622536
P 2.706708 1.023141 -0.759153
C 3.406741 2.519993 -1.541736
H 3.112568 3.414175 -0.984141
H 4.500554 2.467173 -1.590603
H 2.995561 2.599219 -2.553808
C 3.622448 0.826391 0.797309
H 4.696169 0.987848 0.648205
H 3.243829 1.528251 1.546715
H 3.443659 -0.191619 1.160601
C 3.313647 -0.324068 -1.800518
H 4.399763 -0.236607 -1.913030
H 3.072226 -1.279665 -1.323046
H 2.834398 -0.280592 -2.783916
Sn -0.218820 -0.475292 -1.580877
H 0.653827 -2.063506 -1.721299
O 0.357061 -2.436159 -0.362212
C 1.213462 -2.345169 0.650761
C 0.922049 -1.530403 1.760381
C 2.417347 -3.069746 0.657384
C 1.764940 -1.506111 2.861699
H 0.012042 -0.930841 1.738632
C 3.267213 -3.020630 1.756966
H 2.647373 -3.698441 -0.201441
C 2.945204 -2.249122 2.870724
H 1.504995 -0.885951 3.718363
H 4.187282 -3.603597 1.747597

H 3.607490 -2.222502 3.733407

Pathway B

I
E = -1329.48945057
C 1.926261 1.501566 2.109298
C -0.116677 0.305012 1.596785
C -0.732488 0.975778 2.662626
C -0.031907 1.891822 3.436221
C 1.305502 2.156589 3.168213
H 2.974087 1.722965 1.907490
H -1.770622 0.754856 2.896057
H -0.536084 2.390366 4.262216
H 1.864942 2.866602 3.773776
N -0.744903 -0.678702 0.849167
C -2.136023 -0.875207 0.902187
C -3.031721 0.179661 0.680264
C -2.654826 -2.158655 1.105545
C -4.399263 -0.049326 0.661798
H -2.634529 1.175465 0.497627
C -4.026608 -2.385407 1.071000
H -1.965104 -2.979842 1.297373
C -4.907300 -1.332455 0.853765
H -5.074852 0.784061 0.475557
H -4.407652 -3.393340 1.227265
H -5.980853 -1.508750 0.831843
C 1.248354 0.584710 1.298657
P 3.483292 -0.085975 -0.247995
C 4.173468 1.540593 -0.693012
H 4.085250 2.237820 0.144962
H 5.226392 1.453547 -0.984670
H 3.589920 1.935370 -1.532079
C 4.588995 -0.740524 1.036312
H 5.642172 -0.667390 0.742806
H 4.425030 -0.178307 1.962008
H 4.326013 -1.787298 1.221372
C 3.753419 -1.088810 -1.730591
H 4.787260 -0.997838 -2.079859
H 3.532856 -2.139896 -1.515817
H 3.069042 -0.734761 -2.511295
C 1.810422 -0.091552 0.115555
H 1.170946 0.414736 -1.040986
Sn 0.404071 -1.725987 -0.600645
O 0.388900 0.321304 -1.966734
C -0.645164 1.178947 -1.935366
C -1.818903 0.885656 -2.639085
C -0.581183 2.364240 -1.190559
C -2.899031 1.756524 -2.597262
H -1.865955 -0.039496 -3.209952
C -1.666319 3.231642 -1.160504
H 0.321772 2.589076 -0.625211
C -2.832774 2.936247 -1.860359
H -3.806802 1.507112 -3.144435
H -1.599226 4.145591 -0.572369
H -3.681235 3.616508 -1.830723

II
E = -1878.68527743

C -1.443248 -2.950798 -1.638495
C -1.161069 -1.661838 -1.186035
C -2.169760 -0.743107 -0.778055
C -3.494270 -1.252689 -0.850750
C -3.766019 -2.534653 -1.285493
C -2.748963 -3.404902 -1.691816
H -0.608065 -3.576042 -1.947742
H -4.311336 -0.612328 -0.530150
H -4.801984 -2.870594 -1.305352
H -2.975466 -4.405561 -2.050225
N -1.871579 0.481690 -0.281438
S 0.530036 -1.246818 -0.983496
O 1.340775 -2.334141 -1.527769
O 0.764876 0.129519 -1.581621
C 0.759441 -0.898155 0.674705
C -2.882634 1.436609 -0.112831
C -3.691931 1.857646 -1.179964
C -3.045960 2.068942 1.126653
C -4.629489 2.864050 -1.004625
H -3.563209 1.385102 -2.152133
C -3.976808 3.089644 1.294522
H -2.438182 1.739220 1.969222
C -4.776854 3.489722 0.232357
H -5.244358 3.175525 -1.847460
H -4.081208 3.566495 2.267584
H -5.508490 4.284164 0.362904
P 0.295785 -2.068269 1.846326
C 1.002422 -1.542031 3.420394
H 0.799590 -2.278548 4.204807
H 2.081774 -1.408464 3.294380
H 0.564550 -0.577924 3.699978
C 0.926619 -3.716222 1.455339
H 2.013479 -3.660697 1.336477
H 0.661316 -4.439456 2.234315
H 0.495215 -4.024954 0.496255
C -1.487978 -2.251764 2.101751
H -1.934914 -1.253330 2.165049
H -1.934676 -2.771306 1.246286
H -1.686749 -2.816168 3.019901
Sn 0.184770 1.397669 0.250808
O 2.543897 0.857231 0.778941
C 3.696549 0.977355 0.116547
C 4.098697 0.029431 -0.838149
C 4.539050 2.069173 0.370417
C 5.306347 0.176221 -1.507311
H 3.447496 -0.813149 -1.060733
C 5.742172 2.207923 -0.309389
H 4.224541 2.799406 1.113803
C 6.137898 1.263271 -1.252265
H 5.596862 -0.569454 -2.246299
H 6.380473 3.065070 -0.097635
H 7.080953 1.374436 -1.783463
H 1.885913 -0.257415 0.779475

III
E = -2640.76422607
C -2.637056 -0.874324 0.392287
C -2.182894 0.051167 1.337054
C -2.991037 0.356492 2.432947
C -4.243698 -0.224119 2.581344

C -4.703281 -1.124278 1.629856
C -3.900224 -1.443966 0.543463
H -2.613753 1.068288 3.162332
H -4.855589 0.030280 3.444440
H -5.684893 -1.583091 1.730538
H -4.225441 -2.157219 -0.209317
S -1.744227 -1.399039 -1.102750
S -0.548960 0.860342 1.411435
O -2.441244 -2.641010 -1.474836
O -1.882009 -0.219978 -2.025957
O 0.404886 -0.170861 1.837049
O -0.786359 1.974245 2.358085
C -0.119857 -1.544427 -0.836879
P -0.894007 3.039600 -0.419476
P 0.465111 -2.867979 0.058544
C -0.579881 3.507366 -2.136157
H -1.008777 4.495994 -2.333669
H 0.500110 3.533637 -2.318761
H -1.038120 2.767381 -2.801766
C -2.688488 2.899466 -0.264019
H -2.937006 2.750198 0.791675
H -3.185217 3.799882 -0.641370
H -2.997762 2.016793 -0.837775
C -0.364158 4.444967 0.587902
H 0.704814 4.611440 0.415228
H -0.928192 5.349457 0.333415
H -0.516157 4.167848 1.635159
C 2.157614 -2.556022 0.575068
H 2.565297 -3.427858 1.097570
H 2.777492 -2.331578 -0.299086
H 2.147667 -1.681478 1.234026
C -0.499187 -3.219456 1.551237
H -1.531012 -3.440485 1.254465
H -0.080371 -4.079676 2.085343
H -0.474518 -2.324120 2.181324
C 0.433819 -4.416032 -0.887146
H 0.721485 -5.282658 -0.280815
H -0.591005 -4.529389 -1.259805
H 1.105931 -4.320693 -1.746505
C -0.078681 1.545638 -0.089930
H 1.279564 1.501917 -0.021815
O 2.353999 1.043977 -0.456944
C 3.392945 0.662315 0.283113
C 3.421689 0.867716 1.670758
C 4.492185 0.040545 -0.326959
C 4.514806 0.446454 2.416479
H 2.562290 1.327572 2.152508
C 5.577785 -0.378846 0.431269
H 4.472134 -0.103568 -1.406142
C 5.598972 -0.182041 1.809155
H 4.514777 0.608463 3.493576
H 6.420288 -0.861551 -0.062659
H 6.450437 -0.510442 2.401346
Sn 0.615350 0.232137 -1.936453

IV
E = -1848.74953999
C 0.594784 1.643341 2.733937
C 0.173116 0.595739 1.927385
C -1.043979 0.521609 1.240831

C -1.890462 1.629204 1.397002
C -1.513378 2.690275 2.206621
C -0.283495 2.706285 2.878369
H 1.557298 1.616183 3.240946
H -2.856814 1.647979 0.897010
H -2.190325 3.534369 2.325092
H -0.019847 3.549963 3.512368
B -1.180357 -0.852784 0.466958
S 1.092477 -0.827500 1.498242
O 2.018749 -1.356936 2.504069
O -0.131366 -1.755610 1.217500
C 1.804238 -0.523126 -0.019480
C -2.564285 -1.579284 0.303995
C -3.612194 -0.962987 -0.398153
C -2.812969 -2.849031 0.840883
C -4.851545 -1.572903 -0.543006
H -3.445524 0.015150 -0.854843
C -4.048929 -3.470664 0.690867
H -2.018565 -3.352121 1.390916
C -5.076260 -2.834925 0.001858
H -5.645762 -1.066785 -1.090416
H -4.213172 -4.458693 1.119535
H -6.043932 -3.319852 -0.115063
P 3.422206 -1.077244 -0.210903
C 4.604372 -0.295630 0.916171
H 5.619818 -0.672679 0.751021
H 4.580086 0.787421 0.755758
H 4.281241 -0.516930 1.938638
C 3.924011 -0.653774 -1.890276
H 3.726682 0.412096 -2.050956
H 4.985638 -0.872561 -2.047208
H 3.309190 -1.223444 -2.595594
C 3.623615 -2.859970 0.010612
H 3.283762 -3.100415 1.024368
H 2.986067 -3.378278 -0.713473
H 4.666676 -3.168660 -0.119800
C -1.079354 2.602683 -1.774639
C 0.194300 2.456369 -1.213846
C 0.713013 3.485840 -0.424765
C -0.036461 4.633022 -0.196904
C -1.305894 4.775350 -0.747995
C -1.820279 3.752670 -1.540240
H -1.480551 1.797994 -2.391006
H 1.702025 3.367464 0.013728
H 0.377059 5.423579 0.427489
H -1.888243 5.675676 -0.564777
H -2.811539 3.849051 -1.980225
O 0.902026 1.337698 -1.443883
H 1.482756 0.724576 -0.586364
Sn 0.053560 -0.855006 -1.640942

V
E = -1299.51986875
C 2.318244 2.525269 0.411591
C 0.151207 1.808280 -0.418896
C -0.259349 3.151193 -0.383995
C 0.588260 4.172159 0.021363
C 1.882060 3.843209 0.419846
H 3.342378 2.323469 0.722679
H -1.266962 3.394427 -0.720039

H 0.257117 5.208478 0.017077
H 2.568694 4.627988 0.736063
B -0.733300 0.679695 -0.980976
C -2.280651 0.671472 -1.001671
C -3.026880 1.371774 -0.035662
C -2.998761 -0.120898 -1.916429
C -4.412094 1.286075 0.013426
H -2.500714 1.961833 0.712461
C -4.384236 -0.191271 -1.890007
H -2.447884 -0.696075 -2.661114
C -5.094413 0.509811 -0.918025
H -4.963263 1.820039 0.785733
H -4.914821 -0.805075 -2.616041
H -6.180693 0.442656 -0.882041
C 1.478547 1.470642 0.002649
C 1.860682 0.056698 -0.005800
P 3.428392 -0.485035 0.428051
C 4.839952 0.083674 -0.576365
H 4.898011 1.176644 -0.549323
H 5.785523 -0.340867 -0.219841
H 4.666168 -0.225247 -1.612861
C 3.904971 -0.125243 2.151328
H 4.900978 -0.522941 2.377032
H 3.893069 0.953081 2.334543
H 3.165503 -0.593733 2.809968
C 3.469131 -2.290300 0.325726
H 4.397690 -2.679063 0.757568
H 2.603140 -2.685720 0.869658
H 3.385986 -2.598897 -0.721738
Sn 0.614813 -1.152592 -1.541184
C -2.147377 -2.104349 1.191032
C -3.320070 -1.792500 1.864238
C -3.340342 -0.784169 2.824659
C -2.171789 -0.080817 3.094879
C -0.992859 -0.378545 2.421325
C -0.972003 -1.401165 1.467748
H -2.123935 -2.885214 0.433641
H -4.231515 -2.340164 1.630707
H -4.262265 -0.543091 3.349287
H -2.174635 0.717869 3.835085
H -0.079939 0.180683 2.620525
O 0.161125 -1.701767 0.801138
H 0.939964 -0.856316 0.747423

Pathway C

IV
E = -1848.67221227
C -1.747331 1.786289 2.480088
C -1.152693 1.292941 1.324260
C 0.153679 0.774904 1.293787
C 0.801285 0.720965 2.536810
C 0.219574 1.190314 3.710651
C -1.054181 1.741775 3.684019
H -2.753696 2.193636 2.418489
H 1.803179 0.299233 2.588340
H 0.770520 1.127798 4.647612
H -1.513224 2.125883 4.592990
B 0.995560 0.279155 0.005023
S -2.188060 1.309291 -0.151762

O -3.527915 1.772875 0.279198
O -1.470020 2.064948 -1.176720
C -2.333962 -0.362335 -0.613213
C 1.919134 1.291717 -0.768048
C 1.759836 2.672636 -0.596809
C 2.902083 0.867463 -1.676459
C 2.544364 3.586053 -1.293963
H 0.989206 3.037464 0.080299
C 3.695221 1.772358 -2.368283
H 3.047791 -0.199806 -1.850655
C 3.520470 3.141293 -2.178139
H 2.390130 4.653811 -1.145196
H 4.451359 1.410374 -3.063683
H 4.138251 3.854636 -2.721150
P -3.820320 -1.110181 -0.196185
C -4.214971 -1.039238 1.570581
H -5.155468 -1.558551 1.786185
H -3.393794 -1.492403 2.135868
H -4.296101 0.018013 1.842073
C -3.722397 -2.861957 -0.632043
H -2.888376 -3.323952 -0.093354
H -4.656921 -3.370042 -0.370847
H -3.544807 -2.957820 -1.708397
C -5.262045 -0.440590 -1.060097
H -5.295127 0.628975 -0.828686
H -5.117356 -0.570231 -2.137657
H -6.185894 -0.935946 -0.740576
C 2.922500 -1.449932 0.591743
C 3.291897 -2.777451 0.398356
C 4.593405 -3.176123 0.675476
C 5.527382 -2.256089 1.139501
C 5.146098 -0.931396 1.324631
C 3.848205 -0.516279 1.052001
H 2.552286 -3.486380 0.029290
H 4.876812 -4.215891 0.523825
H 6.547108 -2.568742 1.352357
H 5.868564 -0.200044 1.681580
H 3.555580 0.522103 1.175955
O 1.620857 -1.091930 0.348650
H 0.948316 -2.077072 -0.349942
Sn -0.458621 -1.014757 -1.273540

V
E = -1299.47606938
C 1.436838 0.690088 2.671313
C -0.628644 0.253670 1.454693
C -1.250389 0.018823 2.689939
C -0.551049 0.084650 3.887765
C 0.806361 0.406739 3.872402
H 2.480947 1.002678 2.691670
H -2.301117 -0.269836 2.700918
H -1.052825 -0.132263 4.829174
H 1.367213 0.462875 4.804236
B -1.391663 0.027581 0.102919
C -2.944441 -0.062827 -0.061640
C -3.565456 -1.004055 -0.897345
C -3.786662 0.815804 0.642458
C -4.949126 -1.068620 -1.023418
H -2.948699 -1.703571 -1.459733
C -5.167856 0.751777 0.528131

H -3.337841 1.573903 1.284737
C -5.758864 -0.192965 -0.309080
H -5.398677 -1.811640 -1.681005
H -5.791173 1.447677 1.088139
H -6.842271 -0.242191 -0.404430
C 0.740772 0.658024 1.445632
C 1.300433 1.053049 0.161977
P 2.975474 0.851893 -0.149351
C 4.090356 2.139912 0.511884
H 4.004634 2.179639 1.602312
H 5.133723 1.946704 0.235657
H 3.774098 3.107928 0.108660
C 3.652544 -0.714193 0.471882
H 4.727932 -0.785981 0.274158
H 3.468135 -0.785899 1.548076
H 3.124343 -1.537041 -0.020726
C 3.325372 0.857933 -1.924935
H 4.403479 0.764554 -2.097275
H 2.790450 0.024438 -2.390336
H 2.962836 1.790922 -2.369875
Sn -0.145509 1.684566 -1.301886
C 0.237731 -2.550903 0.574137
C 1.103395 -3.626412 0.748130
C 1.910587 -4.074232 -0.292390
C 1.842716 -3.442781 -1.530724
C 0.984888 -2.365931 -1.719925
C 0.187485 -1.927144 -0.665505
H -0.392289 -2.204396 1.385839
H 1.139415 -4.119579 1.717316
H 2.582462 -4.916394 -0.142124
H 2.455119 -3.791271 -2.360089
H 0.909834 -1.868195 -2.685484
O -0.697168 -0.898013 -0.923663
H -0.182697 -0.295974 -1.862838

6.2.7 Phenol activated products

Pathway A

I
E = -1329.48363273
C 0.926257 -2.924643 1.041851
C -1.046710 -1.672045 0.408382
C -1.791070 -2.478708 1.272645
C -1.189046 -3.476599 2.031398
C 0.177925 -3.693818 1.925108
H 1.990702 -3.135093 0.943357
H -2.864575 -2.324418 1.344084
H -1.797202 -4.085695 2.697126
H 0.664024 -4.470385 2.512389
N -1.621186 -0.669120 -0.383021
C -2.889974 -0.117502 -0.148087
C -3.339509 0.211723 1.138708
C -3.714801 0.188724 -1.237188
C -4.574148 0.819229 1.320378
H -2.703885 -0.004337 1.994727
C -4.942129 0.811822 -1.050134
H -3.379103 -0.074904 -2.239385
C -5.383807 1.125547 0.230290
H -4.901052 1.069922 2.328201

H -5.563032 1.042088 -1.914207
H -6.348299 1.606391 0.378468
C 0.358187 -1.907628 0.260480
C 1.108936 -1.091076 -0.691247
P 2.793951 -0.880203 -0.657217
C 3.486227 -0.423821 0.958049
H 3.071586 -1.092678 1.719821
H 4.579884 -0.492118 0.960615
H 3.170994 0.599153 1.191776
C 3.780836 -2.314074 -1.202797
H 4.852205 -2.082280 -1.196461
H 3.594240 -3.167710 -0.543635
H 3.462749 -2.587358 -2.214329
C 3.260015 0.449085 -1.792782
H 4.346370 0.585791 -1.789912
H 2.934632 0.192293 -2.807333
H 2.778513 1.380449 -1.472172
Sn -0.179909 0.284353 -1.512118
H -0.651246 0.730335 -3.067370
O 0.313190 2.150556 -0.818918
C 0.881906 2.298220 0.382952
C 0.466549 1.572752 1.511036
C 1.947285 3.199380 0.526899
C 1.093450 1.752057 2.737335
H -0.354786 0.864081 1.415110
C 2.568180 3.372295 1.758369
H 2.260108 3.768204 -0.347068
C 2.150118 2.648689 2.872433
H 0.751501 1.176011 3.595595
H 3.388558 4.083089 1.847889
H 2.638037 2.786309 3.834890

II
E = -1878.69097650
C -3.941487 0.659587 0.709247
C -2.702541 0.648832 0.075568
C -1.620311 1.443546 0.536188
C -1.875677 2.226444 1.679149
C -3.111504 2.231590 2.304754
C -4.161029 1.449447 1.827771
H -4.729154 0.039789 0.284792
H -1.066629 2.832529 2.079097
H -3.252136 2.852646 3.187852
H -5.133379 1.460865 2.314138
N -0.360785 1.433217 -0.042296
S -2.488885 -0.478738 -1.288350
O -3.770610 -1.136300 -1.529494
O -1.843742 0.314830 -2.368330
C -1.230266 -1.526378 -0.802194
C 0.496344 2.541175 0.078449
C 0.051021 3.843679 -0.186215
C 1.843237 2.343677 0.398173
C 0.936704 4.909209 -0.132211
H -0.993425 4.002635 -0.446483
C 2.730955 3.414384 0.426773
H 2.186283 1.342131 0.650948
C 2.282769 4.703008 0.166874
H 0.575549 5.913956 -0.345147
H 3.775733 3.231886 0.671677
H 2.973162 5.543151 0.197089

P -1.397994 -2.538483 0.572527
C -1.049684 -1.748276 2.168110
H -1.120977 -2.462677 2.996436
H -0.033406 -1.339261 2.112448
H -1.760238 -0.927404 2.322995
C -0.249205 -3.921819 0.446524
H 0.758034 -3.497756 0.370187
H -0.315883 -4.566890 1.329359
H -0.475515 -4.490102 -0.460670
C -3.082660 -3.178503 0.670003
H -3.759573 -2.349507 0.902225
H -3.367940 -3.581004 -0.306348
H -3.149562 -3.945430 1.448885
Sn 0.374800 -0.232225 -1.088212
H 1.286910 0.197399 -2.426814
O 1.646984 -1.144003 0.243201
C 2.980585 -1.246442 0.298159
C 3.846862 -0.491033 -0.503344
C 3.539041 -2.151156 1.212469
C 5.224680 -0.644203 -0.393233
H 3.440194 0.225752 -1.214704
C 4.914900 -2.295592 1.315192
H 2.866501 -2.730743 1.842181
C 5.770948 -1.544899 0.513364
H 5.876168 -0.045605 -1.027927
H 5.324480 -3.004453 2.033271
H 6.849209 -1.660003 0.596423

III
E = -2640.77661450
C -2.137643 -1.087311 -1.043454
C -2.846826 -0.324697 -0.104483
C -4.239955 -0.389413 -0.087186
C -4.935099 -1.176729 -0.995192
C -4.235014 -1.913863 -1.939607
C -2.847476 -1.864249 -1.959508
H -4.765166 0.208648 0.652725
H -6.022258 -1.208571 -0.961081
H -4.762798 -2.530047 -2.664799
H -2.278246 -2.440893 -2.683695
S -0.326486 -1.141135 -1.303446
S -2.149135 0.735084 1.210962
O -0.151937 -2.395394 -2.062829
O -0.004242 0.134448 -1.969416
O -1.618024 -0.218277 2.216147
O -3.286309 1.598812 1.568885
C 0.495265 -1.267248 0.158814
C -0.847894 1.627496 0.645908
P -1.134746 2.916705 -0.443587
P 0.481631 -2.813410 0.891912
C 0.385612 3.360464 -1.291013
H 0.201421 4.214657 -1.951277
H 1.168202 3.595757 -0.563473
H 0.708721 2.484893 -1.864980
C -2.352457 2.501915 -1.713777
H -3.296246 2.243489 -1.220847
H -2.507340 3.351446 -2.387970
H -1.971096 1.635315 -2.264757
C -1.789980 4.380497 0.395358
H -1.035312 4.754594 1.095002

H -2.074006 5.169428 -0.310242
H -2.661319 4.042393 0.968251
C 1.020881 -2.640184 2.604552
H 1.045599 -3.620971 3.090723
H 2.019986 -2.192669 2.636796
H 0.310030 -1.983605 3.120418
C -1.166697 -3.554751 0.963478
H -1.516870 -3.720630 -0.060921
H -1.150565 -4.504230 1.509836
H -1.824030 -2.830319 1.459842
C 1.565546 -4.011174 0.081933
H 1.510086 -4.999393 0.552195
H 1.246145 -4.058376 -0.964690
H 2.590938 -3.627379 0.116114
O 2.404651 1.568950 0.210899
C 3.502566 0.978443 -0.294785
C 4.760643 1.308145 0.223919
C 3.424882 0.058107 -1.349137
C 5.911302 0.729333 -0.296931
H 4.809880 2.028655 1.038048
C 4.582356 -0.519096 -1.857540
H 2.448408 -0.185540 -1.760201
C 5.831477 -0.191744 -1.337418
H 6.881504 1.000108 0.117604
H 4.503114 -1.230508 -2.678540
H 6.734008 -0.645032 -1.742424
Sn 0.888318 0.560230 1.104778
H 1.435914 0.452707 2.705235

IV
E = -1848.77868194
C -0.537196 -1.546495 2.697267
C 0.281832 -0.754280 1.910332
C 1.293704 -1.234454 1.077506
C 1.483537 -2.616993 1.048304
C 0.680337 -3.444917 1.827635
C -0.319247 -2.919204 2.649946
H -1.302396 -1.111405 3.337004
H 2.268278 -3.048100 0.429219
H 0.840033 -4.521363 1.807045
H -0.925082 -3.585385 3.260798
B 2.020344 -0.019307 0.362623
S 0.120706 0.996784 1.680586
O -0.200779 1.683436 2.927694
O 1.622258 1.175272 1.163927
C -0.761836 1.373491 0.343628
C 3.507583 -0.016600 -0.124461
C 4.405814 0.953424 0.335544
C 3.997569 -0.996710 -0.998806
C 5.740882 0.942888 -0.056206
H 4.046054 1.724925 1.014791
C 5.329357 -1.014729 -1.391255
H 3.315717 -1.748136 -1.399294
C 6.207785 -0.042581 -0.918780
H 6.421071 1.708037 0.314923
H 5.685223 -1.785039 -2.073629
H 7.251701 -0.051809 -1.227461
P -2.363629 1.945277 0.474718
C -3.559771 0.773705 1.163101
H -4.562116 1.215417 1.199058

H -3.572675 -0.134013 0.550253
H -3.244121 0.516080 2.180673
C -2.925892 2.387332 -1.178428
H -2.829666 1.514953 -1.833713
H -3.967549 2.723285 -1.145572
H -2.283950 3.188764 -1.559059
C -2.495627 3.430653 1.502102
H -2.088105 3.196090 2.490869
H -1.882849 4.219544 1.054049
H -3.536598 3.763085 1.583907
C -4.452056 -1.830700 -1.590258
C -4.131311 -2.662093 -0.518964
C -2.811014 -2.709333 -0.080199
C -1.830103 -1.929708 -0.681409
C -2.143552 -1.076824 -1.753870
C -3.478564 -1.051671 -2.200710
H -5.476420 -1.789289 -1.958466
H -4.895573 -3.272748 -0.043408
H -2.531714 -3.361641 0.745324
H -0.800995 -1.985833 -0.331512
H -3.721194 -0.411632 -3.047407
O -1.257833 -0.290600 -2.346651
H 0.363837 1.929228 -2.560287
Sn 0.372959 0.628915 -1.418865

V
E = -1299.54007155
C 0.880278 2.993620 0.823215
C -1.114937 1.677720 0.301273
C -1.854657 2.618892 1.061035
C -1.272769 3.693308 1.693471
C 0.117646 3.855904 1.576957
H 1.948036 3.189014 0.744907
H -2.932900 2.484041 1.129059
H -1.868360 4.401707 2.264230
H 0.605809 4.691921 2.077618
B -1.810486 0.472967 -0.301500
C -3.248662 -0.054067 -0.089029
C -3.889937 0.018959 1.161286
C -3.958685 -0.662382 -1.139068
C -5.175672 -0.473603 1.346090
H -3.357254 0.445966 2.009130
C -5.256057 -1.125481 -0.969748
H -3.477877 -0.769691 -2.111717
C -5.867808 -1.034928 0.277574
H -5.641392 -0.419834 2.328620
H -5.787046 -1.576133 -1.806351
H -6.878006 -1.415020 0.419484
C 0.317704 1.886262 0.126084
C 1.094917 1.015493 -0.697746
P 2.798484 0.954841 -0.715829
C 3.655453 2.411549 -1.410046
H 3.433869 3.305283 -0.818973
H 4.740062 2.253716 -1.430447
H 3.286138 2.573785 -2.428194
C 3.587435 0.671231 0.897166
H 4.677187 0.764498 0.827283
H 3.201743 1.395229 1.622266
H 3.318784 -0.334363 1.240080
C 3.339467 -0.411461 -1.767230

H 4.433168 -0.455246 -1.794950
H 2.940688 -1.351103 -1.369153
H 2.952933 -0.259228 -2.780993
Sn -0.152416 -0.513745 -1.494606
H 0.068280 -0.943345 -3.132127
O 0.704545 -2.225344 -0.702745
C 1.215111 -2.245096 0.524964
C 0.701224 -1.479968 1.585747
C 2.339344 -3.049339 0.779274
C 1.286757 -1.522066 2.844280
H -0.166531 -0.841358 1.415434
C 2.919657 -3.083764 2.041016
H 2.732213 -3.651128 -0.038390
C 2.403429 -2.318290 3.084150
H 0.865108 -0.915828 3.644522
H 3.788401 -3.718496 2.211065
H 2.861117 -2.346800 4.070534

Pathway B

I
E = -1329.52832617
C 2.071110 1.032817 1.691552
C -0.230418 0.363099 1.242278
C -0.608562 1.098511 2.383344
C 0.323965 1.822129 3.113189
C 1.668412 1.829814 2.757294
H 3.123194 0.996904 1.406296
H -1.650721 1.116448 2.686642
H -0.014398 2.400061 3.972245
H 2.390264 2.431605 3.304003
N -1.095933 -0.307237 0.409594
C -2.487916 -0.214442 0.499975
C -3.185291 0.988471 0.706418
C -3.248539 -1.374374 0.281139
C -4.573324 1.016524 0.703932
H -2.627435 1.909633 0.851559
C -4.636686 -1.338646 0.266745
H -2.723347 -2.315437 0.121781
C -5.311651 -0.143003 0.485537
H -5.085045 1.964872 0.860943
H -5.194620 -2.257149 0.091488
H -6.399130 -0.112680 0.481175
C 1.160322 0.278097 0.956454
P 2.080168 -2.200142 0.614392
C 3.421011 -2.086953 1.826906
H 3.117136 -1.387999 2.613788
H 3.637088 -3.068348 2.264086
H 4.320278 -1.695784 1.338505
C 0.678921 -2.923966 1.488352
H 0.976947 -3.847762 1.995247
H 0.316329 -2.191664 2.218908
H -0.130091 -3.126978 0.778377
C 2.618251 -3.371442 -0.651458
H 2.897930 -4.331590 -0.205449
H 1.798985 -3.518778 -1.364393
H 3.478582 -2.954864 -1.186877
C 1.606285 -0.674846 -0.118249
H 2.438865 -0.293895 -0.725681

Sn -0.278756 -1.040087 -1.515714
O 0.048818 0.815852 -2.398781
C 0.594781 1.863533 -1.784963
C -0.165330 2.696967 -0.949791
C 1.956454 2.162826 -1.953744
C 0.423992 3.755522 -0.274221
H -1.222351 2.468480 -0.832062
C 2.538308 3.230016 -1.279479
H 2.539784 1.536523 -2.627638
C 1.781262 4.028881 -0.427918
H -0.183295 4.373510 0.385924
H 3.597844 3.440918 -1.422748
H 2.241293 4.857090 0.107054

//
E = -1878.73604993
C 3.123781 -1.353708 1.600724
C 2.148360 -1.185118 0.615564
C 0.781651 -1.494176 0.832582
C 0.470734 -1.953969 2.137738
C 1.433155 -2.095384 3.116460
C 2.777949 -1.803703 2.861588
H 4.155321 -1.128106 1.337715
H -0.568784 -2.184060 2.360069
H 1.132016 -2.435893 4.106026
H 3.535159 -1.936813 3.629980
N -0.194442 -1.321123 -0.108036
S 2.700627 -0.540985 -0.931096
O 4.143535 -0.303632 -0.833525
O 2.187771 -1.335509 -2.051765
C 1.810686 0.985746 -1.026577
C -1.285697 -2.202180 -0.121572
C -1.109324 -3.586468 0.044294
C -2.578671 -1.732738 -0.390308
C -2.185302 -4.454685 -0.048138
H -0.108147 -3.970580 0.228914
C -3.650845 -2.612093 -0.502334
H -2.757860 -0.662250 -0.478710
C -3.465054 -3.976541 -0.325864
H -2.019891 -5.523320 0.079540
H -4.642489 -2.214945 -0.711170
H -4.304965 -4.663326 -0.404789
P 1.964830 2.084757 0.362303
C 1.093292 1.584983 1.854170
H 0.992506 2.467199 2.496406
H 0.102951 1.228868 1.561828
H 1.649258 0.802744 2.379839
C 1.346762 3.686681 -0.176510
H 0.299321 3.537223 -0.461077
H 1.424960 4.408523 0.643222
H 1.935670 4.036181 -1.031067
C 3.698839 2.299089 0.829021
H 4.085283 1.343674 1.197148
H 4.285452 2.583070 -0.050388
H 3.784912 3.065723 1.607513
Sn -0.333199 0.200782 -1.729725
O -0.973395 1.741884 -0.361678
C -2.132646 1.991349 0.238485
C -2.944667 3.054790 -0.190132
C -2.562982 1.240190 1.347870

C -4.127900 3.358999 0.469089
H -2.621392 3.632329 -1.054722
C -3.749676 1.551612 2.000111
H -1.957452 0.394066 1.674245
C -4.540365 2.613284 1.570366
H -4.738604 4.189234 0.116467
H -4.061049 0.952032 2.854456
H -5.468969 2.853046 2.083796
H 2.229620 1.500823 -1.903789

III
E = -2640.79461493
C 2.600148 -0.981824 -0.017202
C 1.743600 -1.589816 0.904290
C 2.231659 -2.599249 1.736506
C 3.551534 -3.019311 1.645278
C 4.396681 -2.435177 0.711320
C 3.917317 -1.427292 -0.114094
H 1.549412 -3.054787 2.449307
H 3.912109 -3.805756 2.304921
H 5.431057 -2.761435 0.622089
H 4.554471 -0.943967 -0.850395
S 2.139097 0.322015 -1.204791
S 0.033841 -1.145559 1.329440
O 3.452135 0.798105 -1.674445
O 1.276720 -0.410136 -2.180326
O 0.078663 0.066295 2.145166
O -0.489360 -2.373458 1.962016
C 1.197253 1.462353 -0.469184
P -1.227546 -2.330749 -1.056305
P 1.896846 2.526697 0.657917
C -1.745746 -1.858470 -2.713533
H -1.991045 -2.753561 -3.295968
H -2.620783 -1.202427 -2.650116
H -0.916025 -1.312913 -3.178894
C 0.230599 -3.364743 -1.241322
H 0.520641 -3.758842 -0.262857
H -0.008129 -4.189000 -1.922210
H 1.030435 -2.742616 -1.659113
C -2.544339 -3.315366 -0.314115
H -3.446793 -2.694759 -0.250885
H -2.750622 -4.220254 -0.896094
H -2.220082 -3.566352 0.701737
C 0.609013 3.366967 1.586422
H 1.056664 4.058180 2.308469
H -0.053582 3.903943 0.900821
H 0.005843 2.597038 2.077525
C 2.964271 1.708083 1.873718
H 3.790630 1.219307 1.345659
H 3.366323 2.437857 2.585450
H 2.362166 0.956846 2.395514
C 2.958828 3.780300 -0.113013
H 3.481415 4.398745 0.625830
H 3.676188 3.237096 -0.739267
H 2.343292 4.412739 -0.761502
C -0.948067 -0.867091 -0.105505
H -1.912317 -0.649721 0.386421
O -1.827082 2.001478 0.443838
C -2.960126 1.390588 0.726097
C -3.153327 0.786869 1.987447

C -3.990848 1.234873 -0.227736
C -4.298204 0.051089 2.261412
H -2.356397 0.880464 2.721168
C -5.130718 0.490395 0.057276
H -3.879137 1.721178 -1.196339
C -5.294890 -0.114962 1.300310
H -4.409557 -0.410644 3.241998
H -5.907238 0.393495 -0.701475
H -6.189474 -0.693083 1.522543
Sn -0.823248 1.238663 -1.325183

IV
E = -1848.77287817

C -0.284599 1.935907 2.694274
C -0.058483 0.798143 1.924588
C -1.037225 -0.003597 1.317684
C -2.362597 0.401041 1.551651
C -2.630770 1.514579 2.328608
C -1.606178 2.290351 2.895291
H 0.537403 2.499154 3.130964
H -3.184368 -0.168954 1.125788
H -3.666269 1.803241 2.497846
H -1.851793 3.162603 3.496380
B -0.405357 -1.188896 0.495573
S 1.481538 0.122889 1.521816
O 2.555309 0.166274 2.516418
O 1.031888 -1.317043 1.175131
C 1.977640 0.780519 -0.024451
C -1.084554 -2.596058 0.353831
C -2.356463 -2.718939 -0.228916
C -0.462357 -3.776244 0.782415
C -2.981395 -3.952268 -0.363071
H -2.863339 -1.825182 -0.597305
C -1.078739 -5.015740 0.642860
H 0.523571 -3.716361 1.241905
C -2.343441 -5.111138 0.072114
H -3.970067 -4.012405 -0.816312
H -0.569449 -5.915196 0.987390
H -2.827636 -6.080077 -0.036880
P 3.673118 0.518564 -0.365091
C 4.825435 1.419862 0.697282
H 5.862914 1.175450 0.442572
H 4.660438 2.495209 0.568541
H 4.608059 1.144157 1.733995
C 3.998668 1.027441 -2.060908
H 3.741904 2.086854 -2.171304
H 5.057189 0.878587 -2.301709
H 3.359394 0.441708 -2.731159
C 4.019594 -1.238563 -0.186338
H 3.818065 -1.525735 0.851705
H 3.347046 -1.797994 -0.846401
H 5.063428 -1.452746 -0.437890
C -2.663924 1.217223 -1.581297
C -1.603394 2.059718 -1.193345
C -1.936894 3.286356 -0.588018
C -3.257816 3.619753 -0.336185
C -4.296053 2.755344 -0.683784
C -3.984877 1.557353 -1.319424
H -2.435260 0.278067 -2.090440

H -1.123125 3.949109 -0.299978
H -3.484481 4.569075 0.148522
H -5.331018 3.021463 -0.480074
H -4.780450 0.876296 -1.619905
O -0.339155 1.739705 -1.367136
H 1.598443 1.795893 -0.181659
Sn 0.368020 -0.274004 -1.634593

V
E = -1299.55838981
C -1.981791 3.253175 0.380024
C -2.382429 0.867859 0.116295
C -3.755721 1.145931 0.038121
C -4.240443 2.447175 0.091977
C -3.345845 3.499415 0.257080
H -1.313238 4.097790 0.542289
H -4.449873 0.319691 -0.112521
H -5.306628 2.644051 -0.002054
H -3.711648 4.524108 0.302220
B -1.782211 -0.528565 -0.211720
C -2.498683 -1.866583 0.087956
C -3.432259 -1.981912 1.135491
C -2.213818 -3.030366 -0.648447
C -4.047889 -3.191427 1.430527
H -3.662812 -1.102817 1.736311
C -2.845572 -4.236236 -0.379151
H -1.478906 -2.979637 -1.451653
C -3.761868 -4.319292 0.666229
H -4.756014 -3.257845 2.254947
H -2.614492 -5.119114 -0.972494
H -4.248100 -5.267437 0.890392
C -1.479591 1.949160 0.319932
C -0.051882 1.581705 0.434156
P 1.253395 2.733189 0.359523
C 1.206962 3.674259 -1.181038
H 0.256820 4.211960 -1.262316
H 2.039734 4.383545 -1.227511
H 1.277577 2.956563 -2.006868
C 1.357114 3.946275 1.712771
H 2.239223 4.585579 1.591951
H 0.458672 4.569830 1.743483
H 1.433580 3.400663 2.659754
C 2.813854 1.836965 0.415348
H 3.649385 2.543585 0.462632
H 2.830857 1.177986 1.290572
H 2.908005 1.203757 -0.474647
Sn 0.261487 -0.163090 -1.267354
H 0.183394 0.851069 1.217498
O 1.356748 -1.041796 0.364063
C 2.598552 -1.474645 0.388073
C 3.236171 -1.649616 1.634735
C 3.355710 -1.752031 -0.767249
C 4.554688 -2.072068 1.715006
H 2.649944 -1.452928 2.531160
C 4.677578 -2.171502 -0.674304
H 2.884695 -1.637914 -1.743520
C 5.293451 -2.334581 0.562531
H 5.014172 -2.201349 2.694695
H 5.232823 -2.379431 -1.588620
H 6.326876 -2.667778 0.628860

Pathway C

IV
E = -1848.80905576
C -0.803743 2.789339 -2.653194
C -0.582705 1.826529 -1.674987
C 0.612979 1.155314 -1.448466
C 1.666667 1.505189 -2.303800
C 1.490811 2.461010 -3.298450
C 0.263202 3.102327 -3.480922
H -1.770195 3.280153 -2.746191
H 2.640823 1.036586 -2.187645
H 2.327683 2.719704 -3.945215
H 0.147358 3.850898 -4.262028
B 0.595444 0.066070 -0.234628
S -1.816695 1.350772 -0.520518
O -2.502941 2.540013 -0.001951
O -2.709816 0.309189 -1.156849
C -0.867462 0.412915 0.606690
C 0.513148 -1.458859 -0.746338
C -0.033496 -1.797557 -1.988316
C 0.841451 -2.510065 0.121454
C -0.266894 -3.125612 -2.342786
H -0.302470 -1.006302 -2.688410
C 0.618935 -3.837349 -0.225244
H 1.281327 -2.277469 1.092785
C 0.055117 -4.149414 -1.460423
H -0.704523 -3.359937 -3.312040
H 0.884115 -4.634644 0.467846
H -0.127898 -5.187317 -1.732955
P -0.626553 1.250352 2.138992
C 0.328255 2.775682 1.970936
H 0.441152 3.272507 2.940713
H 1.302567 2.538285 1.536920
H -0.234638 3.423622 1.288158
C 0.169536 0.139723 3.317444
H 1.157395 -0.144105 2.951087
H 0.244939 0.640638 4.288845
H -0.454207 -0.754779 3.423183
C -2.168953 1.741780 2.940999
H -2.729483 2.389112 2.260466
H -2.771016 0.853532 3.152735
H -1.931708 2.272279 3.870409
C 2.970362 0.040462 0.678282
C 3.567323 -0.664435 -0.375083
C 4.936748 -0.904819 -0.364492
C 5.739217 -0.459260 0.680097
C 5.149194 0.240637 1.728403
C 3.784214 0.488508 1.729266
H 2.956280 -1.031221 -1.193969
H 5.380155 -1.455663 -1.192613
H 6.809373 -0.653483 0.677108
H 5.758588 0.601702 2.555492
H 3.320482 1.042708 2.544127
O 1.663894 0.312662 0.764226
H -3.574378 -0.491160 1.508288
Sn -2.481521 -1.431017 0.416192

V

E = -1299.57385574
C -1.183574 -0.521018 2.683876
C 0.665998 0.392491 1.427149
C 1.198568 0.863717 2.635287
C 0.539432 0.696727 3.846401
C -0.667699 0.003902 3.859079
H -2.088928 -1.125680 2.736863
H 2.145660 1.404322 2.614818
H 0.961788 1.093883 4.767118
H -1.196863 -0.156978 4.797447
B 1.400904 0.821231 0.112359
C 2.932685 0.618690 -0.117248
C 3.600692 1.307209 -1.139702
C 3.682309 -0.252335 0.685901
C 4.962975 1.137602 -1.350843
H 3.030497 1.983385 -1.774921
C 5.044685 -0.428334 0.480519
H 3.178924 -0.809576 1.475351
C 5.687144 0.268446 -0.539134
H 5.464954 1.681613 -2.149345
H 5.608402 -1.113398 1.111378
H 6.754849 0.131545 -0.703865
C -0.544068 -0.351138 1.439421
C -1.031961 -0.991496 0.208143
P -2.731471 -1.048850 -0.081455
C -3.581663 -2.546779 0.525517
H -3.428060 -2.645567 1.605146
H -4.656064 -2.521122 0.307866
H -3.126927 -3.412000 0.030045
C -3.638756 0.347465 0.638371
H -4.703418 0.273332 0.391106
H -3.511971 0.376441 1.723423
H -3.224380 1.268296 0.215701
C -3.180306 -0.996479 -1.833535
H -4.267567 -1.090141 -1.934500
H -2.849235 -0.040568 -2.248635
H -2.678085 -1.813758 -2.358762
Sn 0.382721 -2.162483 -0.883732
H -0.849315 -2.781567 -2.055578
O 0.768598 1.546097 -0.875862
C -0.432907 2.161636 -0.844507
C -0.842081 2.935741 0.242691
C -1.227780 2.099858 -1.988689
C -2.028884 3.656292 0.166472
H -0.217859 2.985455 1.130555
C -2.414844 2.820134 -2.052350
H -0.880429 1.500015 -2.826795
C -2.820872 3.606021 -0.977174
H -2.332465 4.269315 1.013086
H -3.021257 2.775890 -2.955623
H -3.744085 4.178730 -1.032212

7 Comparision of Structural Parameters

Table S7.1. Selected bond lengths [Å] and angles [°] of $^{Tos}Y_2Ge$.

	**XRD**[12]	**PW6B95**	**PBE0**	**PB86**
Ge1–C1	2.035(2)	2.03265	2.03020	2.04754
Ge1–C27	2:049(2)	2.03265	2.04392	2.11379
C1–Ge1–C27	105.94(7)	102.703	103.613	99.686

8 References

[1] R. Dennington, T. A. Keith, J. M. Millam, *GaussView, Version 6.0*, Semichem Inc., Shawnee Mission, **2016**.

[2] M. J. Frisch, G. W. Trucks, H. B. Schlegel, G. E. Scuseria, M. A. Robb, J. R. Cheeseman, G. Scalmani, V. Barone, G. A. Petersson, H. Nakatsuji et al., *Gaussian 16, Revision B.01*, Gaussian, Inc., Wallingford CT, **2016**.

[3] M. J. Frisch, G. W. Trucks, H. B. Schlegel, G. E. Scuseria, M. A. Robb, J. R. Cheeseman, G. Scalmani, V. Barone, G. A. Petersson, H. Nakatsuji et al., *Gaussian 16, Revision C.01*, Gaussian, Inc., Wallingford CT, **2016**.

[4] a) P. Hohenberg, W. Kohn, *Phys. Rev.* **1964**, *136*, B864-B871; b) W. Kohn, L. J. Sham, *Phys. Rev.* **1965**, *140*, A1133-A1138.

[5] Y. Zhao, D. G. Truhlar, *J. Phys. Chem. A* **2005**, *109*, 5656.

[6] F. Weigend, R. Ahlrichs, *Phys. Chem. Chem. Phys.* **2005**, *7*, 3297.

[7] A. Bergner, M. Dolg, W. Küchle, H. Stoll, H. Preuß, *Mol. Phys.* **1993**, *80*, 1431.

[8] a) S. Grimme, J. Antony, S. Ehrlich, H. Krieg, *J. Chem. Phys.* **2010**, *132*, 154104; b) S. Grimme, S. Ehrlich, L. Goerigk, *J. Comput. Chem.* **2011**, *32*, 1456; c) D. G. A. Smith, L. A. Burns, K. Patkowski, C. D. Sherrill, *J. Phys. Chem. Lett.* **2016**, *7*, 2197.

[9] P. Deglmann, F. Furche, *J. Am. Chem. Soc.* **2002**, *117*, 9535.

[10] *Chemcraft - graphical software for visualization of quantum chemistry computations*.

[11] The GIMP team, *GIMP 2.10*, **1995-2019**.

[12] C. Mohapatra, L. Scharf, T. Scherpf, B. Mallick, K.-S. Feichtner, C. Schwarz, V. H. Gessner, *Angew. Chem., Int. Ed.* **2019**, *58*, 7459.
